# Enantioconvergent Chan–Evans–Lam C(sp^3^)–O Coupling: Cu-Catalyzed Asymmetric Benzyl- and Allylborane Oxidation

**DOI:** 10.1021/jacs.6c02555

**Published:** 2026-05-14

**Authors:** Tanner J. Schubert, Ambre Carpentier, Yongxian Li, Kolter C. Hubbell, Jeewhan Oh, Shao-Liang Zheng, Robert S. Paton, Yuyang Dong

**Affiliations:** † Department of Chemistry, 3447 Colorado State University, Fort Collins, Colorado 80523, United States; ‡ Department of Chemistry and Chemical Biology, 1812 Harvard University, Cambridge, Massachusetts 02138, United States

## Abstract

While alkylborane oxidation constitutes one of the most widely utilized strategies to construct C–O bonds, asymmetric versions of this transformation remain elusive. Establishing such an asymmetric approach would unlock a strategically distinct disconnection to oxygen-bearing stereogenic centers, which are ubiquitous across biologically active scaffolds. Herein, we employ a Cu-catalyzed single-electron mechanism to achieve enantioconvergent alkylborane oxidation for the first time. The central challenge—suppressing the unselective carbocation pathway from alkyl radical oxidation by the Cu­(II)–carboxylate—is addressed by (1) lowering the reaction temperature to attenuate the undesired radical–polar crossover while (2) leveraging photochemical activation to preserve the single-electron radical functionalization manifold. The reaction exhibits broad functional-group compatibility, engaging benzyl- and allylboronic esters; diverse carboxylic acids, including complex pharmaceutical substructures and heterocycle-containing substrates, are also well tolerated. The reported protocol is scalable to gram quantities and was utilized for the asymmetric synthesis of an immunosuppressant drug candidate. Mechanistic studies, including stoichiometric interrogation of elementary steps, rate law determination, radical trapping experiments, and density functional theory (DFT) computations, indicate that the reaction operates by balancing two light-driven processes: (1) rate-determining N–H bond homolysis followed by N-radical-mediated C–B bond activation and (2) enantioselective Cu-mediated radical functionalization via an inner-sphere pathway. The reactive Cu­(II)–carboxylate intermediate was isolated and structurally characterized, permitting direct examination of its spectroscopic features and radical-trapping reactivity. Electron paramagnetic resonance (EPR) studies identified the Cu­(II)–carboxylate species as the catalyst resting state, and stoichiometric reaction of the Cu­(II)–carboxylate with a persistent trityl radical demonstrated its competency for C–O bond formation. Hammett analysis further revealed that the efficiency of C–O bond formation is governed by the electrophilicity of the Cu­(II)–carboxylate intermediate.

## Graphical abstract

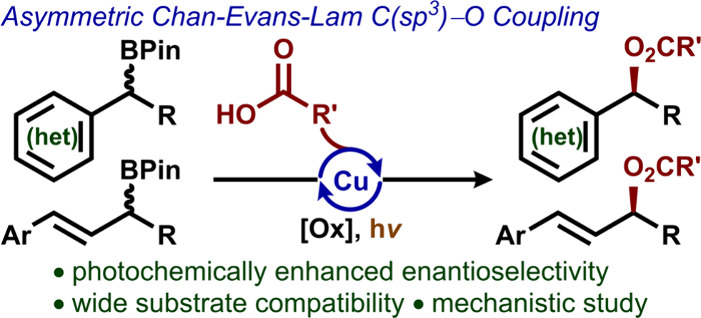

## 1 Introduction

Alkylborane oxidation^1–4^ constitutes one of the most common strategies for constructing C­(sp^3^)–O bonds in natural product synthesis^5–7^ and medicinal^8–11^ and process chemistry,^12,13^ owing to the accessibility and modularity of alkylboron reagents (e.g., Brown hydroboration,^1–4^ Matteson homologation^14–16^ ). Despite the prominence of this transformation, the development of an asymmetric variant has been precluded by the intrinsic stereoretention in the 1,2-metalate rearrangement step (Figure 1A).^1,17–20^ Given that oxygen-bearing stereocenters not only pervade pharmaceutically active agents (Figure 1B)^21–25^ but also serve as synthetically diversifiable substructures,^26–28^ particularly at benzylic and allylic positions, an asymmetric strategy to directly engage racemic alkylboron reagents would offer a strategically distinct retro-synthetic disconnection to these valuable substructures. Accordingly, we report an enantioconvergent Chan–Evans–Lam-type deborylative C­(sp^3^)–O coupling protocol that proceeds through a single-electron pathway.

**Figure 1:**
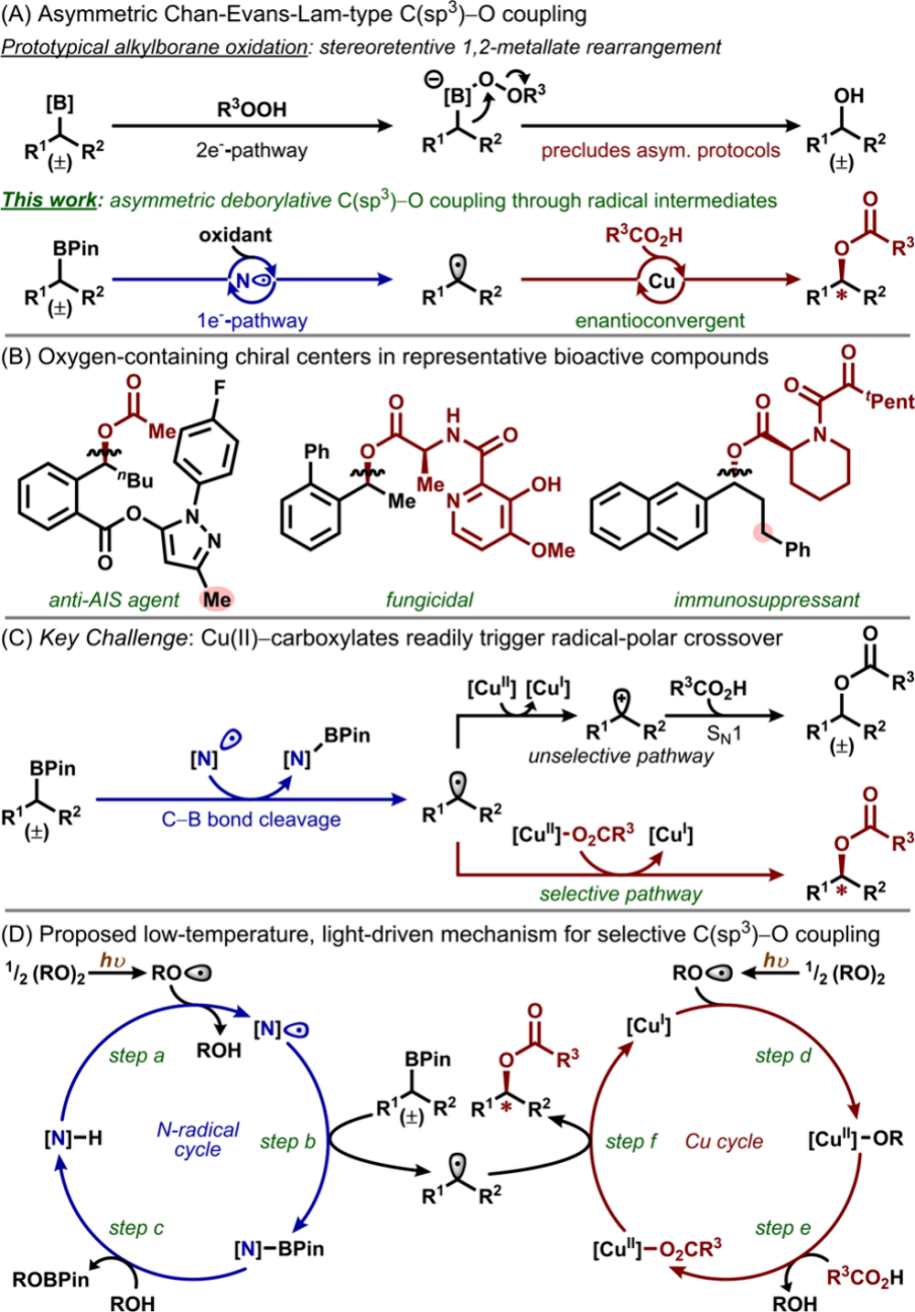
(A) Overcoming the challenge of enantioconvergent alkylboron oxidation via a Chan–Evans–Lam-type deborylative C­(sp^3^)–O coupling protocol that proceeds through a single-electron pathway. (B) Key disconnection for expedited access to oxygen-containing stereocenters as essential pharmaceutical substructures. The reaction orthogonality of the deborylative approach is highlighted by multiple accessible C–H bonds. (C) Key challenge: Cu­(II)–O intermediates can trigger unselective radical–polar crossover, generating carbocations that erode stereocontrol. (D) Proposed low-temperature, light-driven mechanism through N-radical-mediated C–B bond homolysis (steps a–c) followed by Cu­(II) radical functionalization (steps d–f).

We reasoned that the oxidative cross-coupling between alkylboronic pinacol esters and carboxylic acids could benefit from the synthetic advantages of alkylboron reagents while providing an orthogonal alternative to emerging asymmetric methods such as olefin functionalization,^29^ Kharasch-type oxidation,^30–37^ and O-alkylation using organohalides.^38^ We envisioned that a combination of two key single-electron processes could effect this transformation (Figure 1C, bottom): (1) C–B bond homolysis and (2) Cu-mediated asymmetric radical functionalization. Recent studies have demonstrated that N-centered radicals can effectively promote C–B bond homolysis, while bis­(oxazoline) (BOX) ligands enable asymmetric radical functionalization via Cu-mediated inner- or outer-sphere pathways.^39–49^ However, extending this single-electron strategy to asymmetric C­(sp^3^)–O coupling presents a formidable challenge: the high oxidation potential of the Cu­(II)–carboxylate intermediate can divert the reaction toward an oxidative radical–polar crossover mechanism that promotes a racemic carbocation pathway and compromises reaction selectivity (Figure 1C, top).^50–56^

To circumvent this limitation, we devised a photochemical strategy to kinetically suppress the radical–polar crossover pathway while selectively engaging the radical reactivity manifold. Specifically, we envisioned that lowering the reaction temperature would attenuate carbocation formation, whereas photosensitization of peroxides^57–60^ could concurrently drive the N-radical-promoted C–B bond cleavage (steps a–c, Figure 1D) and Cu-mediated radical functionalization (steps d–f). Central to this strategy is the judicious choice of the N-radical precursor to balance the rates between these two light-driven processes. Herein, we report the successful implementation of this photochemical approach to achieve the first asymmetric deborylative C­(sp^3^)–O coupling protocol. Since leveraging photochemical activation to heighten stereocontrol represents an unconventional strategy in asymmetric catalysis, detailed mechanistic investigations were conducted to establish the foundation for future expansion of this asymmetric Chan–Evans–Lam-type cross-coupling platform.

## 2 Results and Discussion

### 2.1 Reaction Development and Optimization

We initiated our investigation by evaluating the deborylative coupling between alkylboronic pinacol ester **1a** and benzoic acid (BzOH, **2a**). Recent work from our laboratory and others established the effectiveness of an in situ generated cationic Cu­(II) species in radical functionalization.^31–35,61–64^ Our initial screening of a CuCl/NaBAr^F^_4_/**L1** catalyst mixture produced the desired product **3a** in moderate yield but low enantioselectivity (Table S1). Notably, formation of vinyl naphthalene was detected, suggesting a competing oxidative pathway that proceeded though a carbocationic intermediate. Guided by this observation, we lowered the reaction temperature to suppress radical–polar crossover and introduced photochemical activation to promote the selective radical pathway. This combination resulted in substantially increased yield and enantioselectivity (up to 97:3 er, Table 1), surpassing the ca. 91:9 er expected from temperature effects alone (see SI for details).^65^ The enhancement in stereocontrol is consistent with the suppression of a competing unselective pathway. Ligand optimization (**L1**–**L6**, Table S2) revealed that **L6** is particularly effective. We attribute this observation to the combined influence of its extended *meso* substituents, which project toward the metal center (*vide infra*), and its distal bulky aryl substituents, which may facilitate favorable noncovalent interactions to further enhance stereoselectivity.^33,42^

**Table 1:**
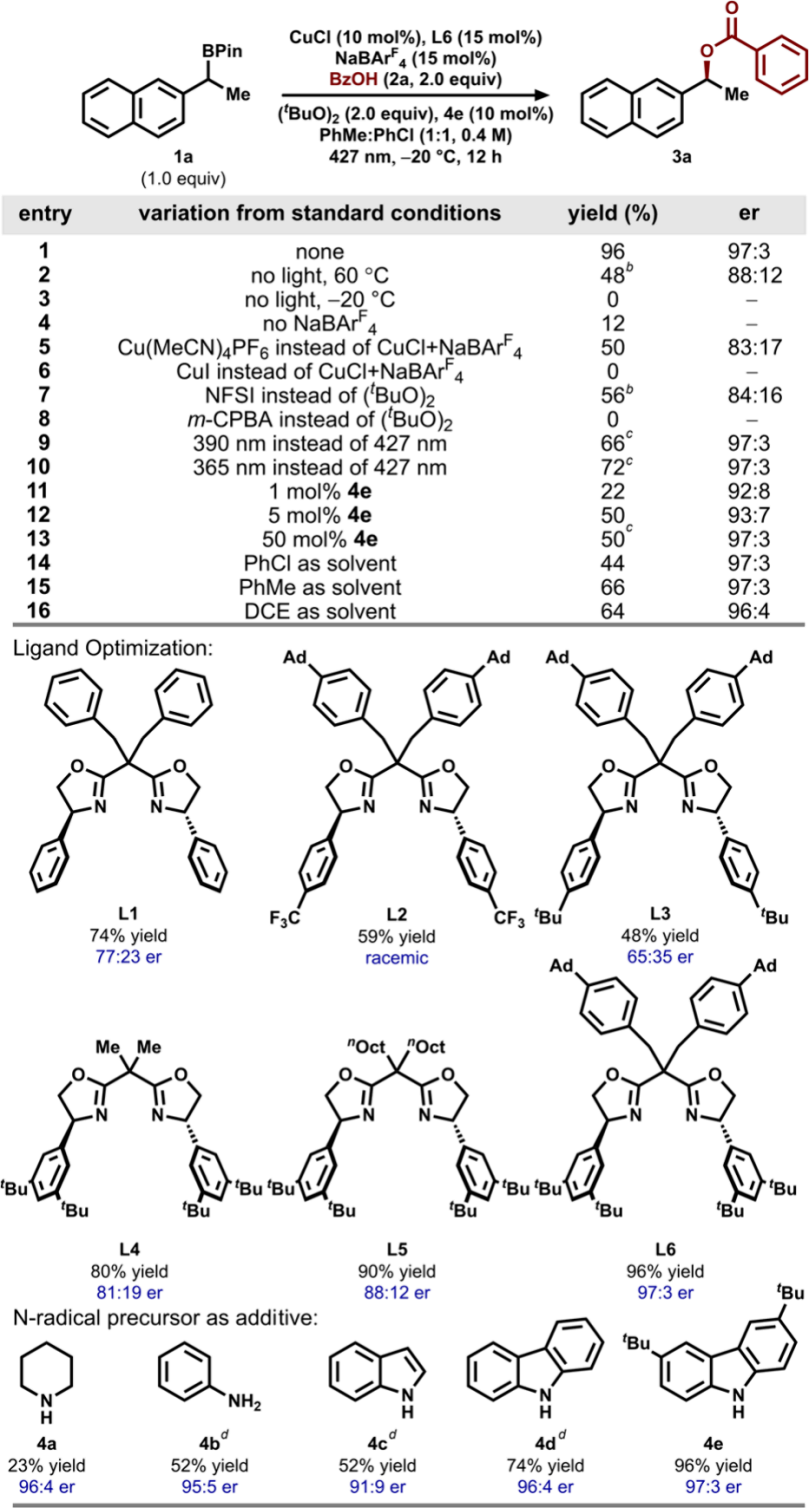
Reaction Optimization Using Alkylboron **1a** ^a^

a Reaction conditions unless otherwise noted: 4,4,5,5-tetramethyl-2-(1-(naphthalen-2-yl)­ethyl)-1,3,2-dioxaborolane (**1a**, 0.10 mmol, 1.0 equiv), BzOH (**2a**, 0.20 mmol, 2.0 equiv), specified Cu, ligand, and additive mixture, di-*tert*-butyl peroxide (0.20 mmol, 2.0 equiv), and a 1:1 mixture of PhCl:PhMe (0.50 mL, 0.2 M); reaction is carried out in a −20 °C freezer under 427 nm LED irradiation; reaction yields were determined by ^1^H NMR spectroscopy of the crude product mixture using 1,1,2,2-tetrachloroethane as an internal standard (see SI for details). The enantiomeric ratio (er) of product **3a** was determined by chiral high-performance liquid chromatography (HPLC) analysis.

b A significant amount (>10%) of 2-vinylnapthalene was observed.

c A significant amount of 2,2′-(butane-2,3-diyl)­dinaphthalene resulting from radical dimerization was observed.

d C–N coupled product with the additive was observed.

Consistent with our proposal that reaction efficiency depends on balancing the N-radical- and Cu-mediated cycles, the reaction proved highly sensitive to the identity and loading of N-radical precursors. 3,6-Di-*tert*-butylcarbazole (**4e**) emerged as the optimal additive (**4a**–**e**), with 10 mol % loading identified as ideal: lower amounts led to incomplete **1a** conversion (entries 11–12), whereas higher loadings promoted benzyl-radical dimerization (entry 13). We hypothesized that the effectiveness of **4e** arises from a combination of facile turnover following N-radical-mediated C–B bond cleavage, favorable energetics for selective HAT activation, weak Cu-binding ability, and enhanced solubility under the reaction conditions (see SI for details). Subsequent screening suggested (^*t*^BuO)_2_ as the most effective oxidant and that a 1:1 PhMe:PhCl solvent system afforded the highest yield (entries 14–16), likely by tuning the overall solvent polarity for stabilizing the cationic Cu and alkyl radical intermediates. We proposed that the light-driven process is initiated through photochemical cleavage of (^*t*^BuO)_2_. Accordingly, the weak visible-light absorption of (^*t*^BuO)_2_ prompted us to evaluate the effect of irradiation wavelengths.^58–60^ UV–Vis spectra of the individual reaction components and their mixtures under the model reaction conditions are provided in the SI. The optimal result was achieved using a 427 nm light source, whereas a substantial amount of alkyl-radical dimerization was observed at shorter wavelengths (entries 9–10).

### 2.2 Substrate Scope of the Enantioconvergent Deborylative C­(sp^3^)–O Coupling

With the optimized reaction conditions in hand, we next explored the functional group tolerance and the scope of compatible alkylboronic pinacol esters (Table 2) and carboxylic acids (Table 3). The reaction accommodated a wide range of substrates. Benzylic and allylic alkylboronic pinacol esters, together with aryl and primary, secondary, and tertiary alkyl carboxylic acids, couple efficiently to provide the desired products in high yields and enantioselectivities.

**Table 2:**
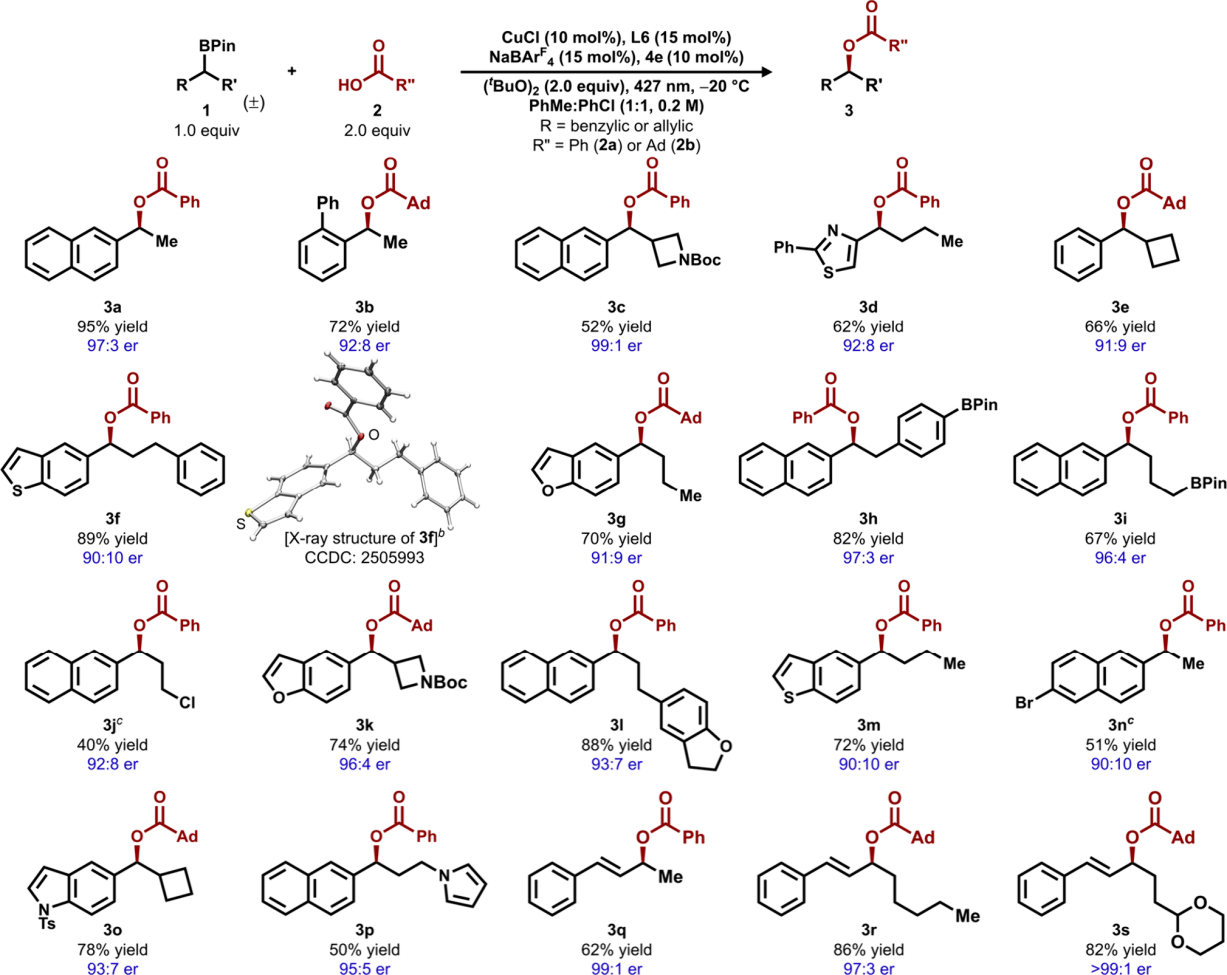
Scope of Alkylboronic Pinacol Esters **1** in Enantioconvergent Deborylative C­(sp^3^)–O Coupling^a^

a Yields were obtained using alkylboronic pinacol ester (**1**, 0.50 mmol), unless otherwise noted. The enantiomeric ratio (er) was determined by chiral HPLC analysis.

b Solid-state molecular structure for **3f** with thermal ellipsoids at the 50% probability level. Color scheme: S, yellow; C, gray; O, red; H, white.

c Reactions carried out at 25 °C for higher conversion.

**Table 3:**
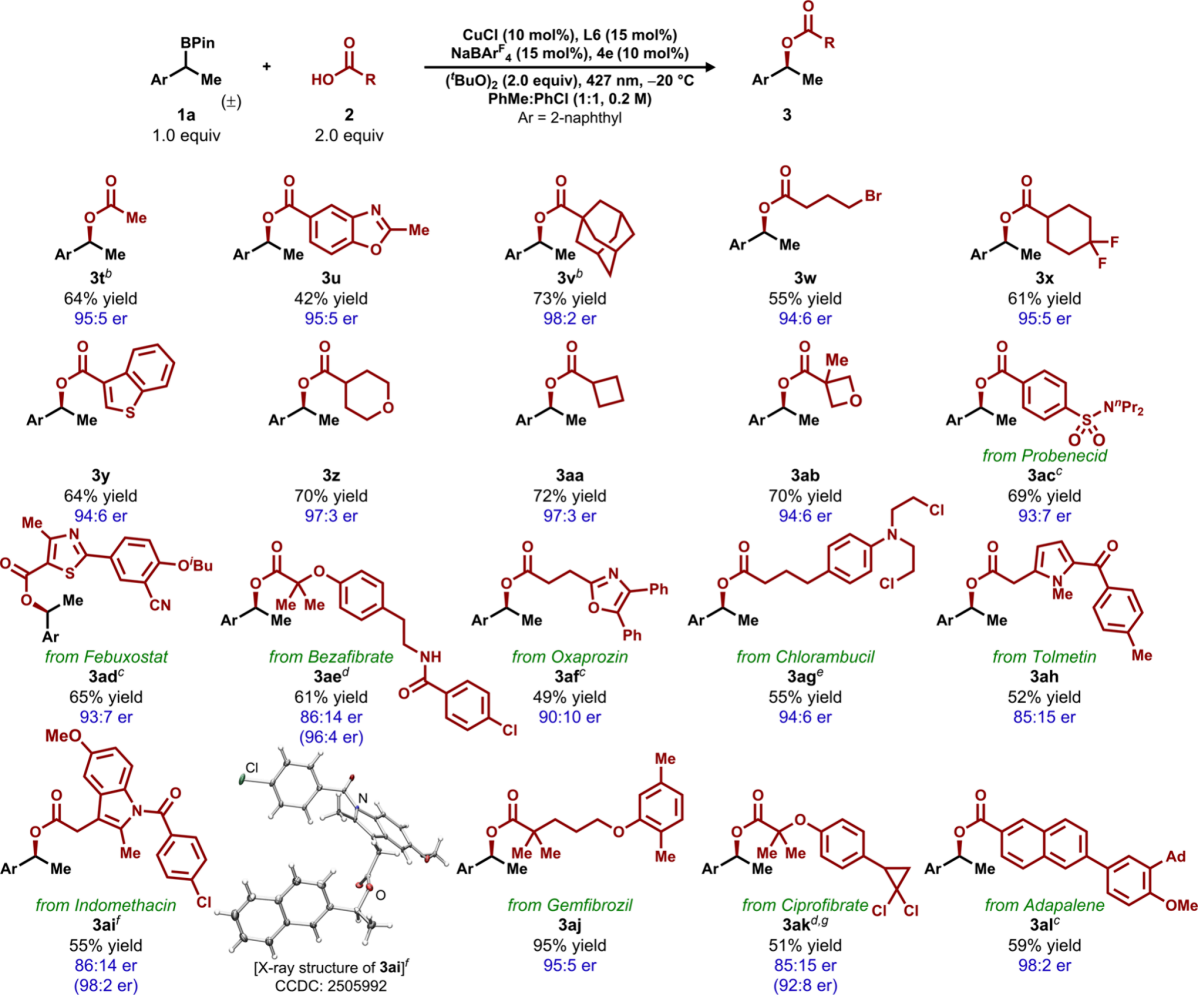
Scope of Carboxylic Acids **2** in Enantioconvergent Deborylative C­(sp^3^)–O Coupling^a^

a Yields were obtained using alkylboronic pinacol ester (**1a**, 0.50 mmol), unless otherwise noted. The enantiomeric ratio (er) was determined by chiral HPLC analysis.

b Reactions carried out at 25 °C for higher conversion.

c Reactions were carried out in DCE (0.2 M) for higher solubility of **2**.

d Enantioselectivity after bulk recrystallization with 65% (**3ae**), 71% (**3ai**), and 53% (**3ak**) recovery yields (see SI for details).

e The reaction was also performed using carboxylic acid **2ag** as the limiting reagent, leading to a similar result (47% yield, 94:6 er) (see the SI for details).

f Solid-state molecular structure for **3ai** with thermal ellipsoids at the 50% probability level. Color scheme: Cl, green; C, gray; O, red; N, blue; H, white.

g A 1:1 dr was determined by ^1^H NMR spectroscopy.

Coordinating functional groups such as aza-nitrogen (**3d**, **3u**, **3ad**, **3af**), amine (**3ag**), amide (**3ae**), sulfonamide (**3ac**), and ketone (**3ah**) are compatible under the optimized conditions. The reaction also tolerates a wide range of medicinally relevant heterocycles, including pyrrole (**3p**, **3ah**), indole (**3o**, **3ai**), oxazole (**3af**), thiazole (**3d**, **3ad**), benzoxazole (**3u**), azetidine (**3c**, **3k**), oxetane (**3ab**), tetrahydropyran (**3z**), benzofuran (**3g**, **3k**), benzothiophene (**3f**, **3m**, **3y**), and dioxolane (**3s**).^66–69^ Substrates featuring alkyl (**3j**, **3w**, **3x**, **3ag**, **3ak**) or aryl halides (**3n**) react smoothly, with no detectable C–halogen bond activation. The reaction also proceeds cleanly with substrates containing weak benzylic (**3f**, **3h**, **3l**, **3af**, **3ag**, **3ah**) and ketal C–H bonds (**3s**) that could be competitive in C–H functionalization protocols.^30–37^ These results highlight the orthogonality of this deborylative coupling platform relative to existing asymmetric radical cross-coupling protocols. Additional selectivity studies revealed that the protocol effectively differentiates distinct boronate sites. In substrates containing additional aryl (**3h**) or primary alkyl boronic esters (**3i**), functionalization occurs exclusively at the benzylic position. The broad applicability of this protocol was further demonstrated through the functionalization of a wide range of pharmaceuticals (**3ac**–**3al**). The enantioselectivity of **3ae**, **3ai**, and **3ak** can be enhanced through bulk recrystallization with 53%–71% recovery yield. The reaction can also be conducted using the carboxylic acid as the limiting reagent (**3ag**), leading to similar result. The absolute configuration of the major enantiomer was assigned through a combination of optical rotation measurements^30^ and single-crystal X-ray diffraction of representative products (**3f** and **3ai**).

### 2.3 Synthetic Applications of Asymmetric Deborylative C­(sp^3^)–O Coupling

To demonstrate the synthetic utility of the enantioconvergent C­(sp^3^)–O coupling strategy, the preparations of **3aj** and **3b** were performed on a 5.0 mmol scale, delivering yield and enantioselectivity comparable to those obtained on a 0.5 mmol scale (Scheme 1A). We further applied the method in a concise asymmetric synthesis of the immunosuppressant **8** (Scheme 1B).^21^ Beginning from commercially available benzylboronic pinacol ester **5**, C–C bond formation via a boron-stabilized α-carbanion provided the requisite alkylboron **6**.^70^ Subsequent C­(sp^3^)–O coupling furnished the enantioenriched **7** that is converted to the target molecule **8** with high selectivity.

**Scheme 1:**
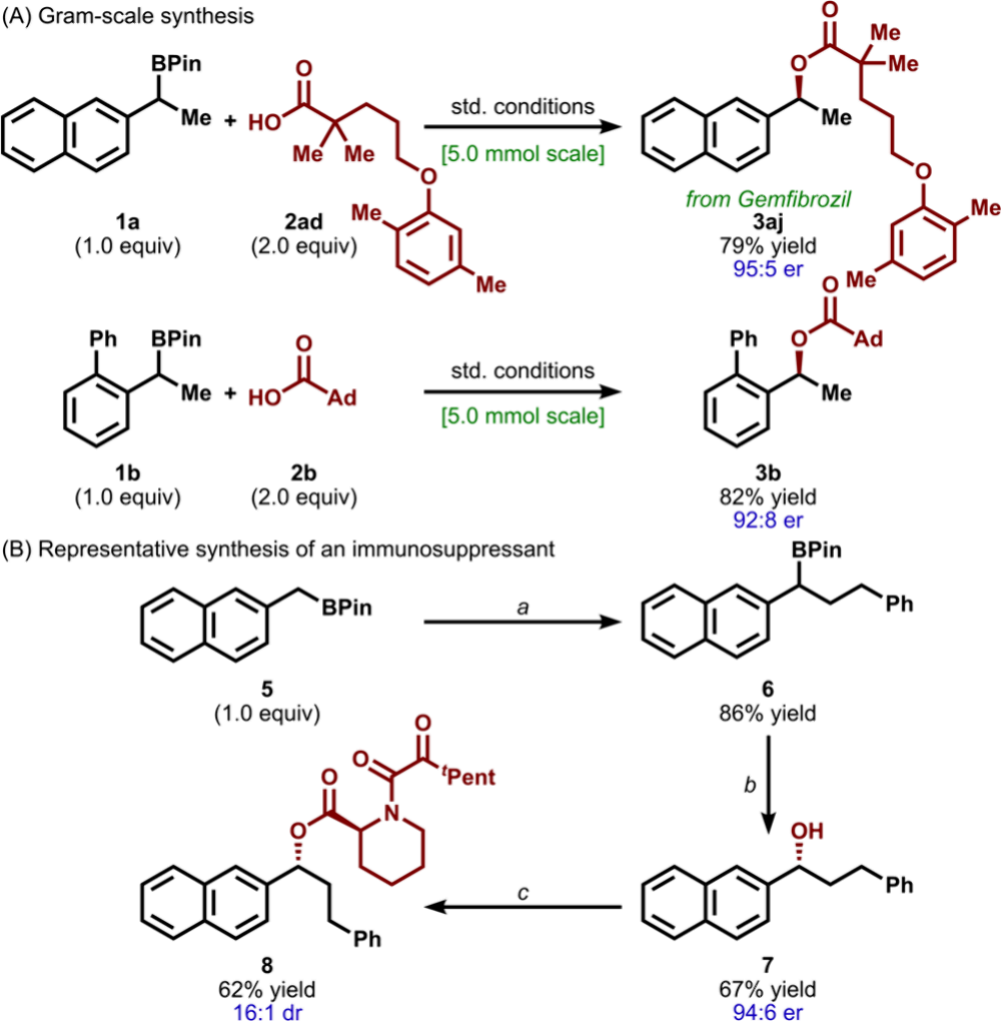
Applications of the Asymmetric Alkylborane Oxidation Protocol

a Reaction conditions: lithium 2,2,6,6-tetramethylpiperidinide (LTMP, 1.0 equiv), (2-bromoethyl)­benzene (1.2 equiv), THF, 0 → 25 °C, 1 h.
b Reaction conditions of a one-pot procedure: C­(sp^3^)–O coupling with **2b** was carried out using **L6′** (the enantiomer of **L6**) under standard conditions; *then* KOH (aq.), MeOH, 40 °C, 24 h.
c Reaction conditions: (*S*)-1-(3,3-dimethyl-2-oxopentanoyl)­piperidine-2-carboxylic acid (1.0 equiv), DCC (1.1 equiv), DMAP (0.5 equiv), DCM, 25 °C, 16 h.

Leveraging photochemical activation to heighten stereocontrol represents an unusual and largely unexplored tactic in asymmetric catalysis. To clarify how visible light affects the selectivity of this transformation, we undertook a comprehensive mechanistic investigation aimed at identifying the key rate- and selectivity-determining steps underlying the deborylative C­(sp^3^)–O coupling. These insights lay a foundation for new mechanistic frameworks in designing asymmetric radical processes.

### 2.4 Mechanistic Investigation into the Photochemical Activation of Alkylboronic Pinacol Esters

Our proposed mechanism comprises two sequential processes: (1) photochemical activation of C–B bonds and (2) light-driven, Cu-mediated asymmetric radical functionalization. We began our investigation by probing the alkyl radical generation cycle. Radical trapping experiments were performed using TEMPO (**9**, see SI for details). In the presence of stoichiometric TEMPO, the C­(sp^3^)–O coupled product **3a** was observed (44%) in addition to the corresponding TEMPO adduct **10** (36%). Complete inhibition of **3a** formation was observed with additional TEMPO (2 equiv) present. Together, these trapping studies suggest the intermediacy of alkyl radicals under the reaction conditions. Accordingly, we proposed the following photochemically initiated sequence for C–B bond activation (Figure 2A). Photolysis of the (^*t*^BuO)_2_ oxidant generates ^*t*^BuO•, which undergoes H atom transfer (HAT) with carbazole **4e** to produce the corresponding N-radical **11** (step a). Subsequent radical homolytic substitution between **11** and alkylboron substrate **1** furnishes the key alkyl radical intermediate **13** that enters the Cu-mediated catalytic cycle (step b), while boryl-group transfer between resulting carbazoleborane **12** and ^*t*^BuOH regenerates the carbazole cocatalyst **4e** (step c).

**Figure 2:**
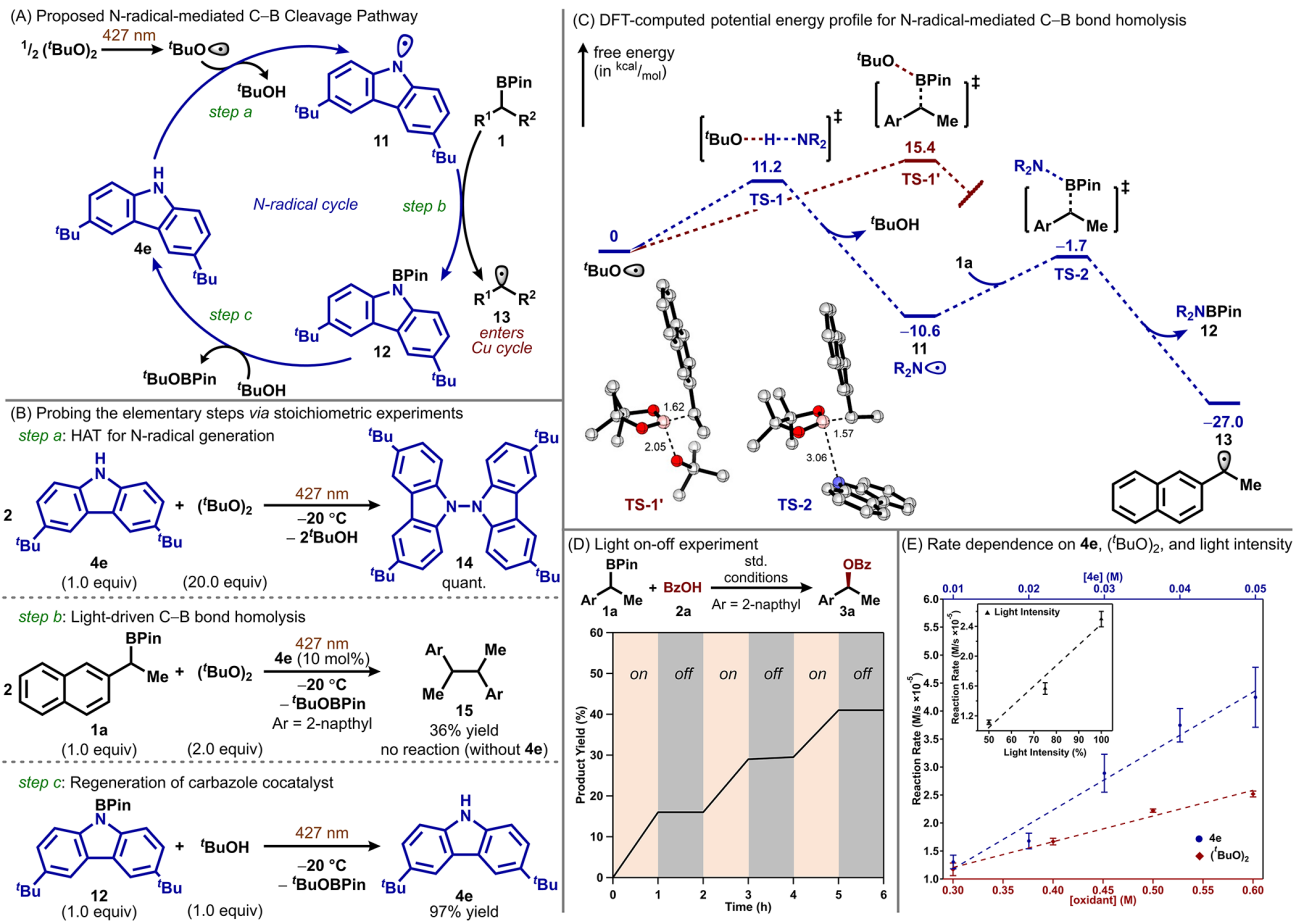
Mechanistic interrogation of the N-radical-mediated alkyl-radical generation pathway. (A) Proposed catalytic cycle featuring N-radical-initiated C–B bond activation. (B) Stoichiometric evaluation of key elementary steps under catalytic conditions: (step a) H atom transfer between cocatalyst **4e** and photochemically generated ^*t*^BuO•; (step b) light-driven dimerization of alkylboron reagent **1a** through C–B bond homolysis requires cocatalyst **4e**; (step c) regeneration of cocatalyst **4e** through BPin-group transfer between **12** and ^*t*^BuOH. (C) Computational comparison of C–B homolysis pathways, highlighting the energetically preferred N-radical-mediated route over direct BPin-group transfer to ^*t*^BuO•. (D) Light on–off experiments establishing the necessity of photochemical activation. (E) Initial-rate kinetic measurement suggests first-order rate dependence on [**4e**], [(^*t*^BuO)_2_], and light intensity (inset: light intensity referenced to standard reaction conditions).

We systematically evaluated each elementary step of the proposed pathway through a series of stoichiometric experiments (Figure 2B). Irradiating a mixture of **4e** and (^*t*^BuO)_2_ under reaction conditions led to quantitative carbazole dimerization to generate **14** (step a), consistent with productive N-radical formation. Subsequent C–B bond homolysis (step b) was probed by subjecting a mixture of alkylboron **1a** and (^*t*^BuO)_2_ to the low-temperature, photochemical conditions. Alkyl-radical dimerization (**15**) was observed only in the presence of 10 mol % **4e**, whereas no reaction occurred in its absence, demonstrating the effectiveness of the in situ generated N-radical in cleaving C–B bond. Carbazole turnover (step c) was evaluated by treating independently prepared carbazoleborane **12** with stoichiometric ^*t*^BuOH, which under the reaction conditions effectively regenerated carbazole cocatalyst **4e** and ^*t*^BuOBPin.

The energetics of the proposed pathway were examined using DFT computations at the PW6B95-D3­(BJ)/def2-TZVP/SMD­(PhCl)//PW6B95-D3­(BJ)/def2-SVP level of theory (Figure 2C).^71–75^ All geometries were optimized using Gaussian 16, and final free energies were obtained using GoodVibes.^76–78^ The single-electron activation of **1a** by either an alkoxy radical ^*t*^BuO• or a carbazole-derived N-centered radical **11** is highly exergonic (Δ*G* = −26.7 and −27.0 kcal/mol, respectively). Despite their comparable thermodynamics, the two pathways display markedly different kinetic profiles. The activation barrier for O-radical-mediated C–B bond homolysis proceeds through a barrier of Δ*G* ^‡^(**TS-1′**) = 15.4 kcal/mol, whereas the N-radical generation via HAT is more kinetically favorable (step a, Δ*G* ^‡^(**TS-1**) = 11.2 kcal/mol), followed by a low barrier for C–B bond cleavage (step b, Δ*G* ^‡^(**TS-2**) = 8.9 kcal/mol). Formation of the N-radical **11** is predicted to be the rate-determining step of the overall transformation, with a kinetic barrier that exceeds those associated with the subsequent Cu-mediated steps (*vide infra*).

A light on–off experiment revealed that photochemical activation is necessary for catalytic turnover (Figure 2D). The light dependence also enabled us to monitor the transformation by ^1^H NMR spectroscopy and extract reaction orders through initial-rate analysis.^65^ In line with the computationally determined rate-determining N-radical generation (step a), the reaction exhibits first-order dependence on light intensity as well as on the concentrations of **4e** and (^*t*^BuO)_2_ (Figure 2E). Zero-order dependence was obtained for the concentrations of coupling partners **1a** and **2a**, likely because these substrates enter the catalytic cycle only after the rate-determining step (steps b and e, Figure 3A, *vide infra*). Measuring the rate dependence on the Cu catalyst concentration proved to be challenging, complicated by the low solubility of isolated cationic Cu species.

**Figure 3:**
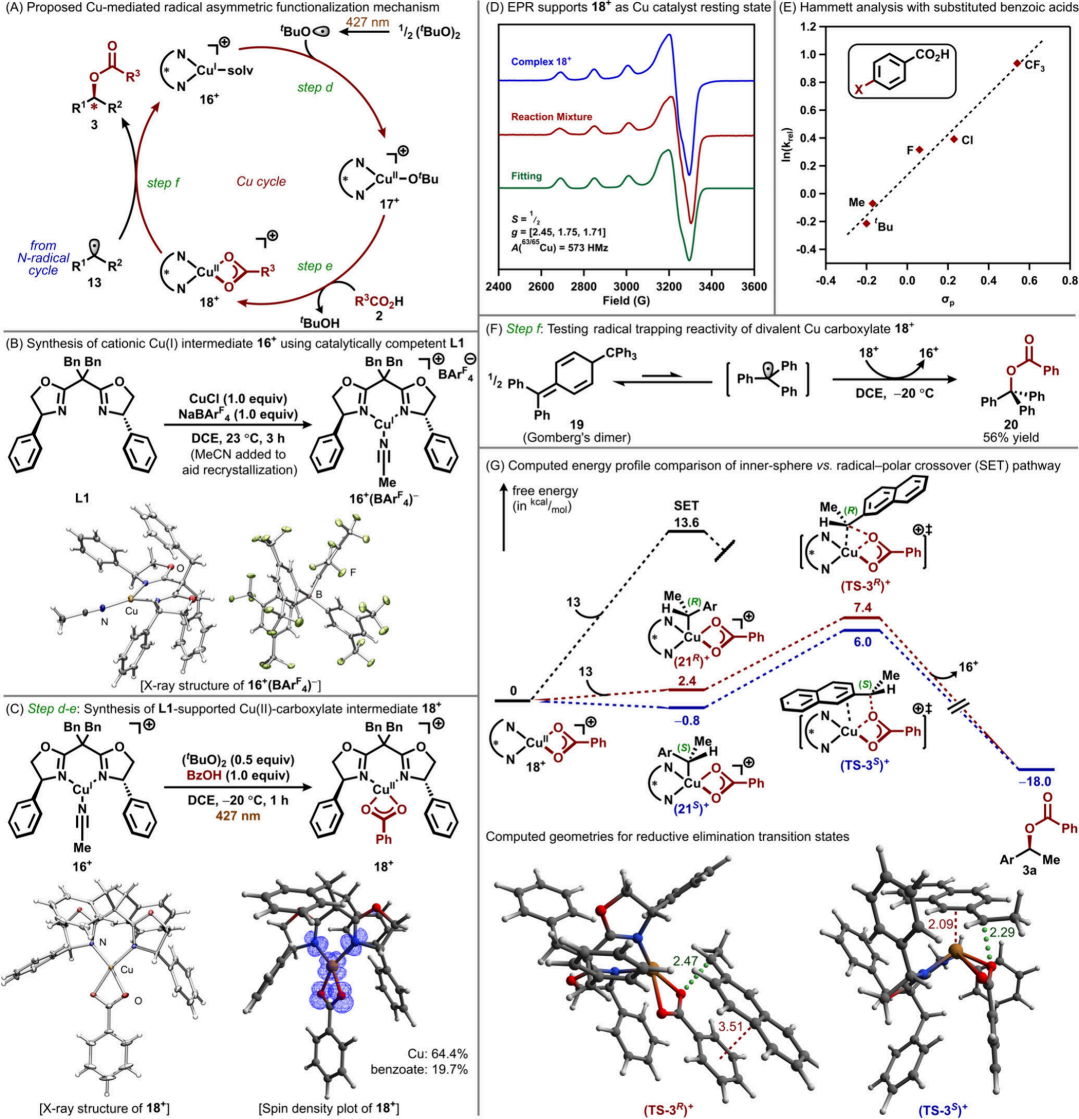
Mechanistic evaluation of the Cu-mediated alkyl-radical functionalization pathway. (A) Proposed catalytic cycle for Cu-mediated C­(sp^3^)–O coupling. (B) Independent synthesis of cationic [(**L1**)­Cu­(MeCN)]­(BAr^F^_4_) through halide abstraction and its solid-state molecular structure with thermal ellipsoids at 50% probability level. Color scheme: Cu, yellow; B, pink; F, yellow-green; C, gray; O, red; N, blue; H, white. (C) (steps d–e) Independent synthesis of reactive Cu­(II) intermediate [(**L1**)­Cu­(OBz)]^+^ (**18** ^**+**^) through sequential oxidation and ligand exchange. Solid-state structure of complex **18** ^**+**^ with thermal ellipsoids at 50% probability level and computed spin density plot of complex **18** ^**+**^ showing 64.4% and 19.7% Cu- and benzoate-based spin density, respectively. (D) Frozen-solution EPR spectra of complex **18** ^**+**^ and the reaction mixture, identifying **18** ^**+**^ as the Cu resting state during the transformation. Spectral simulation affords the following parameters: *S* = ^1^/_2_, *g*_1_ = 2.45, *g*_2_ = 1.75, *g*_3_ = 1.71, *A*(^63/65^Cu, *I* = ^3^/_2_) = 573 MHz. (E) Elucidating the polarity requirements of the deborylative C­(sp^3^)–O coupling through Hammett analysis. (F) (step f) Stoichiometric reaction of complex **18** ^**+**^ with Gomberg’s dimer **19** (trityl radical) to evaluate its competency in C–O bond-forming radical functionalization. (G) Computational analysis comparing inner-sphere and single-electron-transfer pathways identifies a Cu-mediated inner-sphere mechanism as energetically preferred, with reductive elimination TSs favoring formation of the *S*-enantiomer through a stabilizing η^3^-allyl coordination mode in **(TS-3** ^*S*^ **)** ^**+**^.

### 2.5 Cu-Mediated Pathways for C­(sp^3^)–O Bond Formation

We propose that the Cu-mediated radical functionalization proceeds through a radical-relay-type catalytic cycle (Figure 3A):^40^ photochemically generated ^*t*^BuO• oxidizes the cationic monovalent catalyst **16** ^**+**^ to form a Cu­(II)–alkoxide intermediate **17** ^**+**^ (step d). Subsequent transmetalation with carboxylic acid **2** furnishes the reactive Cu­(II)–carboxylate **18** ^**+**^ (step e), which captures alkyl radical **13** to deliver product **3** through either an inner- or outer-sphere coupling pathway (step f). To assess the viability of the proposed mechanism, we undertook the independent synthesis and structural characterization of the putative intermediates and probed their reactivity. We sought to examine two key features of the proposed mechanism: (1) halide abstraction by NaBAr^F^_4_ to form cationic intermediate **16** ^**+**^ and (2) C­(sp^3^)–O bond construction through radical capture by Cu­(II)–carboxylate **18** ^**+**^. The synthesis of these putative intermediates also served to probe the elementary steps d–e in our proposed Cu-mediated catalytic cycle (Figure 3A–C).

We prepared the proposed Cu complexes using catalytically competent ligand **L1** and solvent DCE. Treating **L1** sequentially with stoichiometric CuCl and NaBAr^F^_4_ (Figure 3B) generated a colorless solid. ^1^H and ^19^F NMR analysis of the resulting material indicated the formation of diamagnetic complex **16** ^**+**^ **(BAr** ^**F**^_**4**_ **)** ^–^. Crystals suitable for single-crystal X-ray diffraction analysis were obtained by slow diffusion of pentane into a concentrated DCM/acetonitrile solution of the material at −30 °C. The presence of acetonitrile proved essential for crystal growth, likely due to its stronger coordination to the Cu center relative to other solvents present. The solid-state structure confirms the abstraction of chloride and the noncoordinating nature of the BAr^F^_4_^–^ counterion.

Direct oxidation of **16** ^**+**^ using a mixture of (^*t*^BuO)_2_ and BzOH proved to be sluggish at 23 °C, requiring 6 h for full conversion as indicated by ^1^H NMR analysis. The reaction is inhibited at low temperature (−20 °C), showing <10% conversion over 36 h. However, exposing a mixture of **16** ^**+**^, (^*t*^BuO)_2_ (0.5 equiv), and BzOH (1.0 equiv) to 427 nm irradiation at −20 °C (steps d–e, Figure 3C) led to complete consumption of the starting materials in 1 h, along with the generation of a blue paramagnetic complex (no discernible ^1^H NMR features were observed). Crystals were obtained by slow diffusion of pentane into a concentrated DCM solution of the material at −30 °C. Single-crystal X-ray diffraction analysis revealed the product as the cationic complex [(**L1**)­Cu­(OBz)]^+^ (**18** ^**+**^).^53,54^ The Cu­(II) center adopts a square planar geometry (Σ∠Cu = 359.82°), with the benzoate ligand bound in a κ^2^-fashion. The electron paramagnetic resonance (EPR) spectrum of **18** ^**+**^ (Figure 3D) reveals a rhombic, *S* = 1/2 signal (*g*_1_ = 2.45, *g*_2_ = 1.75, *g*_3_ = 1.71) with hyperfine features (*A*(^63/65^Cu, *I* = 3/2) = 573 MHz) observed with the *g*_1_ signal. Similar EPR features were observed with a flash-frozen reaction mixture. Quantitative analysis of the spectrum using a calibration curve (see SI for details) revealed that complex **18** ^**+**^ accounts for 88% of all Cu species present in the catalytic mixture, suggesting **18** ^**+**^ as the Cu catalyst resting state.^53,54^

Computational analysis of the spin density distribution in **18** ^**+**^ (Figure 3C) revealed a major contribution from Cu (64.4%) and a non-negligible contribution from the benzoate ligand (19.7%). The substantial ligand-based spin density highlights the pronounced electrophilicity of divalent copper carboxylates, consistent with the radical–polar crossover reactivity frequently associated with such intermediates. Accordingly, we evaluated the polarity matching in the reaction through a Hammett analysis. A binary competition protocol was employed to probe the post-rate-determining C­(sp^3^)–O bond-forming step, using the coupling of parent substrates **1a** and **2a** as the internal reference (see SI for details). Correlating the relative rates with Hammett parameters (σ_p_) of substituted benzoic acids revealed a positive ρ value (Figure 3E). This trend reflects the increasing reactivity of electron-deficient benzoic acids and can be ascribed to faster transmetalation resulting from the heightened acidity and/or more efficient trapping of nucleophilic alkyl radicals by electrophilic complex **18** ^**+**^. A complementary Hammett analysis using *para*-substituted benzylboronic pinacol esters (**3e**_**X**_) revealed a negative ρ value (see SI for details). We attributed this observation to more facile C–B bond cleavage by the N-centered radical and/or more efficient trapping of the electron-rich benzyl radicals by electrophilic Cu­(II)–carboxylate intermediates. Additionally, correlation of product enantiomeric ratios with substituent σ_p_ parameters showed modest erosion in stereocontrol for more electron-rich alkylboranes, consistent with an increased propensity for the competing radical–polar crossover pathway.

The alkyl radical trapping ability of **18** ^**+**^ (step f) was independently probed by treating **18** ^**+**^ with Gomberg’s dimer **19** (0.5 equiv, as a source of trityl radical) at −20 °C (Figure 3F). The reaction mixture exhibited a color change from green to light yellow. ^1^H NMR analysis of the resulting mixture revealed the formation of C–O coupled product **20** and the regeneration of the monovalent complex **16** ^**+**^. We subsequently prepared the *para*-substituted benzoate analogues **18**_**CF3**_^**+**^ and **18**_**Me**_^**+**^ and compared their radical-trapping reactivities with that of **18** ^**+**^ using a binary competition protocol. A modest substituent effect was observed (**18**_**CF3**_^**+**^/**18** ^**+**^ = 1.31; **18**_**Me**_^**+**^/**18** ^**+**^ = 0.93). The small magnitude suggests that the overall rate differences observed in the Hammett study (Figure 3E) likely reflect the combined contributions from radical trapping and acid transmetalation steps (see SI for details).

The C­(sp^3^)–O bonding-forming mechanism and the origins of enantioconvergence were investigated through DFT computation. Figure 3G displays the lowest energy pathway identified, proceeding through the most accessible conformers of each stationary point (see SI for other conformers). We modeled the coupling between alkylboronic pinacol ester **1a** and benzoic acid **2a** with truncated but catalytically competent **L1**. Computational results indicate that the formation of divalent copper intermediate **18** ^**+**^ is exergonic, formed by trapping photogenerated ^*t*^BuO• (step d), followed by transmetalation with benzoic acid **2a** (step e, Δ*G* = −14.7 kcal/mol, Scheme S4). The association of alkyl radical **13** to **18** ^**+**^ results in the formation of formal Cu­(III) intermediates **(21** ^***R***^ **)** ^**+**^ (Δ*G* = 2.4 kcal/mol) and **(21** ^***S***^ **)** ^**+**^ (Δ*G* = −0.8 kcal/mol). Despite many attempts, locating transition states (TSs) for the formation of **(21** ^***R***^ **)** ^**+**^ and **(21** ^***S***^ **)** ^**+**^ proved challenging, consistent with reports on related Cu-catalyzed radical cross-coupling reactions.^56,79,80^ Based on scans of the energy surface, we believe this association to be extremely facile. The subsequent reductive elimination step features TSs **(TS-3** ^***R***^ **)** ^**+**^ and **(TS-3** ^***S***^ **)** ^**+**^ with retention of configuration at the benzylic position. Comparison of their lowest-energy conformers (see Schemes S7–S9 for all identified conformers) gives activation free energies of Δ*G* ^‡,*R*^ = 7.4 kcal/mol for **(TS-3** ^***R***^ **)** ^**+**^ and Δ*G* ^‡,*S*^ = 6.0 kcal/mol for **(TS-3** ^***S***^ **)** ^**+**^, corresponding to a ΔΔ*G* ^‡^ of 1.4 kcal/mol in favor of **(TS-3** ^***S***^ **)** ^**+**^ and consistent with the experimental observations. Close inspection of the most energetically accessible reductive elimination TSs (see Scheme S12 for NCI plots)^81–83^ revealed a π–Cu interaction (2.09 Å) in **(TS-3^*S*^)** ^**+**^, whereas the lowest-energy conformer of **(TS-3^*R*^)** ^**+**^ is stabilized through alkyl radical–carboxylate π–π stacking (3.51 Å).^29,33–36,84^

The radical–polar crossover pathway was evaluated by computing the Marcus barriers associated with a single-electron transfer (SET) using a variant of the Nelsen four-point method (see SI for details).^85–87^ The SET manifold features a higher, although thermally accessible, barrier (Δ*G* ^‡,SET^ = 13.6 kcal/mol). This result aligns with the experimentally observed temperature dependence of enantioselectivity. At elevated temperature (60 °C), the accessible SET pathway can competitively divert reactivity from the enantioselective inner-sphere radical functionalization, eroding stereocontrol. In contrast, at low temperature (−20 °C), the relative barrier heights predict an ca. 10^4^-fold increase in kinetic preference (see SI for details) for the enantioselective inner-sphere pathway over SET pathways, consistent with the enhancement in enantioselectivity observed under low-temperature, photochemical conditions.

## 3 Conclusion

In summary, we have developed the first enantioconvergent deborylative C­(sp^3^)–O coupling, enabled by a photochemical strategy that selectively promotes homolytic C–B activation while suppressing competing radical–polar crossover pathways. Synergistic low-temperature control and light-driven N-centered radical initiation unlock a Cu-catalyzed single-electron manifold that delivers oxygenated stereogenic centers with high efficiency and enantioselectivity across a broad substrate scope. Mechanistic interrogation revealed that N–H HAT serves as the rate-determining step, while Cu­(II)–benzoate complexes mediate selective radical capture through an inner-sphere pathway consistent with experimental and computational analyses. The synthetic utility of this method is demonstrated through late-stage functionalization of pharmaceuticals, scalable esterification, and asymmetric synthesis of a bioactive compound. Collectively, these studies establish a mechanistically guided platform for asymmetric alkylboron oxidation and lay the foundation for future expansion of enantioconvergent Chan–Evans–Lam-type C­(sp^3^)–heteroatom cross-coupling reactions.

## Supplementary Material



## Notes

Experimental procedures and characterization data for all new compounds, including NMR spectra, HPLC traces, computational details, and Cartesian coordinates of all computed structures (PDF)
