# Supplementary material for: Enantioconvergent Chan–Evans–Lam C(sp3)–O Coupling: Cu-Catalyzed Asymmetric Benzyl- and Allylborane Oxidation
Source: J Am Chem Soc. 2026 May 14;148(20):20694–704. doi: 10.1021/jacs.6c02555 (PMC13198982; doi:10.1021/jacs.6c02555)
Supplement: Supplementary file 1 [file ja6c02555_si_001.pdf]

***Supporting Information***

# Enantioconvergent Chan-Evans-Lam C(sp$^3$)–O Coupling: Cu-Catalyzed Asymmetric Benzyl- and Allylborane Oxidation

Tanner J. Schubert,[a] Ambre Carpentier,[a] Yongxian Li,[a] Kolter C. Hubbell,[a] Jeewhan Oh,[b] Shao-Liang Zheng,[b] Robert S. Paton,[*,a] Yuyang Dong[*,a]

[a]Department of Chemistry, Colorado State University, Fort Collins, Colorado 80523, United States

[b]Department of Chemistry and Chemical Biology, Harvard University, Cambridge, Massachusetts 02138, United States

## Table of Contents


**1. General Experimental Details** .......... 3

**2. Optimization of Reaction Conditions** .......... 5

**3. Synthesis and Characterization of Starting Materials** .......... 12

**5. Synthesis and Characterization of C($sp^3$)–O Coupled Products** .......... 20

**6. Crystallographic Information** .......... 68

**7. Experimental Procedures for Mechanistic Investigations** .......... 74

**8. Associated Analytical Data** .......... 113

**9. Computational Details** .......... 208

**10. References** .......... 285

## 1. General Experimental Details

*General Experimental Setup*: All reactions were performed in flame-dried or oven-dried glassware fitted with rubber or PTFE/silicone septa under a positive pressure of nitrogen, unless otherwise noted. Standard reactions were performed in glass culture tubes with threaded ends (Fisherbrand, 16 × 125 mm, catalog no. 1495935A; oven-dried at 140 °C) that were sealed with screw-thread caps (Kimble Chase Open Top S/T Closure, catalog no. 73804-15425) fitted with Teflon/SIL septa (Thermo Scientific, catalog no. B7995-15). Air- and moisture-sensitive liquids were transferred via syringe through rubber or PTFE/silicone septa. Solids were added under inert atmosphere or were dissolved in the appropriate solvent prior to addition. Reactions carried out at temperatures above room temperature (25 °C) were conducted in a preheated oil bath. Photochemical reactions carried out at −20 °C were conducted in a −20 °C freezer equipped with a Kessil 45 W blue LED (Model No: KSPR160L-427). All reactions were magnetically stirred and monitored by NMR spectroscopy or analytical thin-layer chromatography (TLC) using glass-backed plates pre-coated with silica gel (250 µm, 60-Å pore size, Extra Hard Layer, SiliaPlate) impregnated with a fluorescent indicator (254 nm). TLC plates were visualized by exposure to ultraviolet light (UV) or stained by submersion in aqueous potassium permanganate solution ($KMnO_4$) followed by heating with a heat gun. Flash column chromatography was performed using SiliCycle SiliaFlash® F60 silica gel (40-63 µm, 230-400 mesh, 60 Å pore diameter) and with the aid of a Teledyne ISCO CombiFlash Automated Flash Chromatography System using SNAP silica cartridges (10–100 g). Reversed-phase column chromatography was performed with the aid of a Teledyne ISCO CombiFlash Automated Flash Chromatography System using prepacked SNAP C18 cartridges (40-100 g).

*Materials*: Unless noted otherwise, all reagents and starting materials were purchased from commercial sources and used as received (Millipore-Sigma, Alfa Aesar, Strem, TCI-America, Combi-Blocks, Ambeed, or Enamine). $CDCl_3$ and $C_6D_6$ was purchased from Millipore-Sigma. Anhydrous benzene, tetrahydrofuran (THF), diethyl ether ($Et_2O$) and dichloromethane ($CH_2Cl_2$) were obtained from J.T. Baker in CYCLE-TAINER® delivery kegs and purified by successive filtrations though packed columns of neutral alumina and CuO under Ar pressure. Solvents for extraction, crystallization, and flash column chromatography were purchased in A.C.S. reagent grade. The yields refer to chromatographically and spectroscopically ($^1H$ and $^{13}C$ NMR) pure material.

*Instrumentation*: NMR spectra were measured on a Bruker Avance NEO 400 spectrometer. Proton chemical shifts are expressed in parts per million (ppm, δ scale) and are referenced to the residual proton in the NMR solvent ($CDCl_3$: δ 7.26). All $^{13}C$ spectra recorded are proton-decoupled. The carbon chemical shifts are expressed in parts per million (ppm, δ scale) and are referenced to the carbon resonance of the NMR solvent ($CDCl_3$: δ 77.16). $^{1}H$ NMR spectroscopic data are reported as follows: Chemical shift in ppm (multiplicity, coupling constants *J* (Hz), integration intensity). The multiplicities are abbreviated with s (singlet), br. s (broad singlet), d (doublet), t (triplet), q (quartet), hept (heptet), m (multiplet), and a (apparent). $^{13}C$ NMR spectroscopic data are reported as follows: Chemical shift in ppm (multiplicity, coupling constants *J* (Hz)). All $^{19}F$ chemical shifts are expressed in parts per million (ppm, δ scale). All raw fid files were processed and the spectra analyzed using the program *MestReNOVA 14.2.1 from Mestrelab Research S. L.* High-resolution mass spectra were recorded on an Agilent 6545 quadrupole time of flight mass spectrometer (TOF-U) interfaced to an Agilent 1290 Infinity UHPLC with electrospray (ESI) and IonSense Direct Analysis in Real Time (DART) ambient ionization source. IR spectra were recorded on a Thermo Nicolet iS-50 FTIR spectrometer equipped with ATR crystals (diamond, ZnSe, and germanium). IR spectra were acquired from neat samples. If required, substances were dissolved in $CH_2Cl_2$ before direct application on the ATR unit. Data are reported as follows: frequency of absorption ($cm^{-1}$).

## 2. Optimization of Reaction Conditions

*a. Reaction optimization procedure under thermo conditions:*

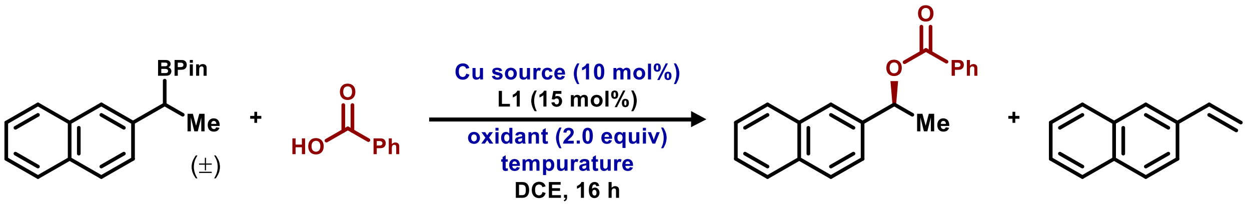


**1a** 1.0 equiv  **2a** 2.0 equiv  **3a**  **3a'**

Inside a nitrogen-filled glovebox, an oven-dried reaction tube (Fisherbrand, 16 × 125 mm, catalog no. 1495935A) equipped with a magnetic stir bar was charged with the corresponding Cu salt (0.010 mmol, 10 mol%) and **L1** (7.3 mg, 0.015 mmol, 15 mol%) followed by the addition of DCE (0.5 mL). The reaction mixture was stirred at room temperature for 10 min before the addition of benzoic acid (24.4 mg, 0.200 mmol, 2.0 equiv) and 4,4,5,5-tetramethyl-2-(1-napthylethyl)-1,3,2-dioxaborolane (**1a**, 28.2 mg, 0.10 mmol, 1.0 equiv). The reaction mixture was stirred for 10 min before the corresponding oxidant (0.20 mmol, 2.0 equiv) was added. The reaction tube was sealed with a screw cap (Kimble Chase Open Top S/T Closure, catalog no. 73804-15425) containing a PTFE septum (Thermo Scientific, catalog no. B7995-15), taken outside of the glovebox, and placed in a preheated oil bath or left stirring at room temperature (25 °C).

After the reaction mixture had vigorously stirred for 16 h at specified temperatures, the screw cap was removed from the reaction tube and acetone (3 mL) was added to the reaction mixture. The resulting reaction mixture was stirred at room temperature (25 °C) for an additional 5 min and concentrated *in vacuo* with the aid of a rotary evaporator. Internal standard 1,1,2,2-tetrachloroethane (11 μL, 0.10 mmol) and $CDCl_3$ (1 mL) were added to the crude residue to determine the crude $^1$H NMR yield. A small portion of the crude reaction mixture was purified by preparative silica thin layer chromatography, and the resulting purified sample was analyzed by chiral HPLC to determine the enantioselectivity (er).

**Table S1.** Optimization of Cu source, additive, oxidant, and temperautre under thermo conditions[a]

| | Cu-source | oxidant | temp (°C) | yield **3a** (%) | yield **3a'** (%) | er |
|---|---|---|---|---|---|---|
| 1 | CuCl+NaBAr$^F_4$ | $(CumylO)_2$ | 60 | 48 | 32 | 62:38 |
| 2 | CuCl | $(CumylO)_2$ | 60 | 18 | 12 | 54:46 |
| 3 | CuOAc | $(CumylO)_2$ | 60 | 44 | 25 | 52:48 |
| 4 | $Cu(CH_3CN)_4PF_6$ | $(CumylO)_2$ | 60 | 18 | 30 | 63:37 |
| 5 | CuCl+NaBAr$^F_4$ | $(^tBuO)_2$ | 60 | 45 | 36 | 65:35 |

| | | | | | | |
|---|---|---|---|---|---|---|
| 6 | CuCl+NaBAr$^F_4$ | BzOO$^t$Bu | 60 | 8 | *N.D.* | – |
| 7 | CuCl+NaBAr$^F_4$ | $^t$BuOOH | 60 | *N.D.* | *N.D.* | – |
| 8 | CuCl+NaBAr$^F_4$ | NFSI | 60 | *N.D.* | *N.D.* | – |
| 9 | CuCl+NaBAr$^F_4$ | (CumylO)$_2$ | 25 | 4 | *N.D.* | – |
| 10 | CuCl+NaBAr$^F_4$ | (CumylO)$_2$ | 80 | 20 | 35 | 54:46 |

[a]*N.D.*: not detected. NaBAr$^F_4$: sodium tetrakis[3,5-bis(trifluoromethyl)phenyl]borate

*b. Reaction optimization procedure under low-temperature, photochemical conditions:*

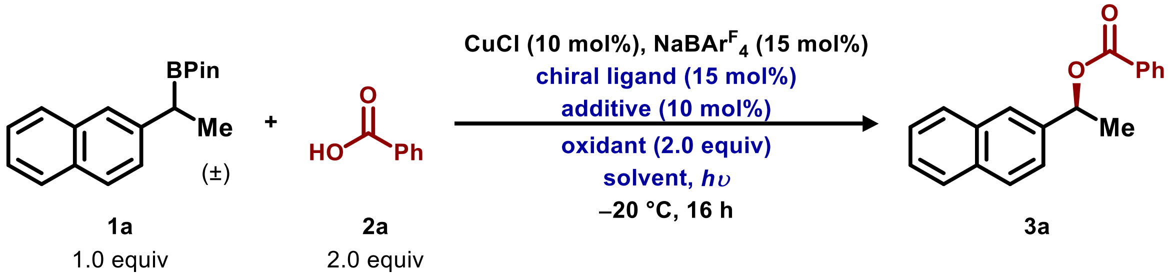


Inside a nitrogen-filled glovebox, an oven-dried reaction tube (Fisherbrand, 16 × 125 mm, catalog no. 1495935A) equipped with a magnetic stir bar was charged with CuCl (1.0 mg, 0.010 mmol, 10 mol%), NaBAr$^F_4$ (13.3 mg, 0.015 mmol, 15 mol%), and chiral ligand (0.015 mmol, 15 mol%), followed by the addition of specified solvent (0.5 mL). The reaction mixture was stirred at room temperature for 10 min before the addition of the additive (0.01 mmol, 10 mol%), benzoic acid (24.4 mg, 0.200 mmol, 2.0 equiv), and 4,4,5,5-tetramethyl-2-(1-(naphthalen-2-yl)ethyl)-1,3,2-dioxaborolane (**1a**, 28.2 mg, 0.10 mmol, 1.0 equiv). The reaction mixture was stirred for 5 min before the corresponding oxidant (0.20 mmol, 2.0 equiv) was added in one portion. The reaction tube was sealed with a screw cap (Kimble Chase Open Top S/T Closure, catalog no. 73804-15425) containing a PTFE septum (Thermo Scientific, catalog no. B7995-15), taken outside of the glovebox, placed in a –20 ºC freezer, and irradiated with the specified wavelength of light.

After the reaction mixture had vigorously stirred for 16 h, the screw cap was removed from the reaction tube and acetone (3 mL) was added to the reaction mixture. The resulting reaction mixture was stirred at room temperature (25 °C) for an additional 5 min and concentrated *in vacuo* with the aid of a rotary evaporator. Internal standard 1,1,2,2-tetrachloroethane (11 µL, 0.10 mmol) and $CDCl_3$ (0.5 mL) were added to the crude residue to determine the crude $^1$H NMR yield. A small portion of the crude reaction mixture was purified by preparative silica thin layer chromatography, and the resulting purified sample was analyzed by chiral HPLC to determine the enantioselectivity (er).

**Table S2.** Optimization of oxidant, solvent, and irradiation wavelength[a]

| | solvent | oxidant | wavelength (nm) | yield **3a** (%) | er |
|---|---|---|---|---|---|
| 1 | DCE | $(^t\text{BuO})_2$ | 427 | 64 | 96:4 |
| 2 | PhCl | $(^t\text{BuO})_2$ | 427 | 44 | 97:3 |
| 3 | PhMe | $(^t\text{BuO})_2$ | 427 | 66 | 97:3 |
| 4 | DCM | $(^t\text{BuO})_2$ | 427 | 74 | 91:9 |
| 5 | dioxane | $(^t\text{BuO})_2$ | 427 | 49 | 97:3 |
| 6 | PhMe:PhCl (1:1) | $(^t\text{BuO})_2$ | 427 | 96 | 97:3 |
| 7 | PhMe:PhCl (1:1) | $(^t\text{BuO})_2$ | 427 | 96 | 97:3 |
| 8 | PhMe:PhCl (1:1) | NFSI | 427 | 56 | 84:16 |
| 9 | PhMe:PhCl (1:1) | $(\text{CumylO})_2$ | 427 | 92 | 97:3 |
| 10 | PhMe:PhCl (1:1) | *m*CPBA | 427 | *N.D.* | – |
| 11 | PhMe:PhCl (1:1) | $(^t\text{BuO})_2$ | 390 | 66 | 97:3 |
| 12 | PhMe:PhCl (1:1) | $(^t\text{BuO})_2$ | 365 | 72 | 97:3 |
| 13 | PhMe:PhCl (1:1) | $(^t\text{BuO})_2$ | none | *N.D.* | – |
| 14[b] | PhMe:PhCl (1:1) | $(^t\text{BuO})_2$ | none | 48 | 88:12 |
| 15[c] | PhMe:PhCl (1:1) | $(^t\text{BuO})_2$ | 427 | 12 | – |
| 16[d] | PhMe:PhCl (1:1) | $(^t\text{BuO})_2$ | 427 | *N.D.* | – |
| 17[e] | PhMe:PhCl (1:1) | $(^t\text{BuO})_2$ | 427 | 50 | 83:17 |
| 18[f] | PhMe:PhCl (1:1) | $(^t\text{BuO})_2$ | 427 | *N.D.* | – |

[a]Reaction carried out with additive **4e** (see Table S3 for additive optimization) and ligand **L6** (see Table S4 for ligand optimization). *N.D.*: not detected. $\text{NaBAr}^F_4$: sodium tetrakis[3,5-bis(trifluoromethyl)phenyl]borate. [b]Reaction carried out at 60 °C. A significant amount of **3a'** (16%) was observed. [c]Reaction carried out in the absence of $\text{NaBAr}^F_4$. [d]Reaction carried out with CuI. [e]Reaction carried out with $\text{Cu(MeCN)}_4\text{PF}_6$. [f]Reaction carried out without Cu.

*Note*: During reaction optimization, we observed similar results with $(^t\text{BuO})_2$ or $(\text{CumylO})_2$ as the oxidant. Since $^t$BuOH as a reaction byproduct is easily removed *in vacuo*, $(^t\text{BuO})_2$ was used in this study unless otherwise noted.

*Note*: Experimentally, we observed an enantiomeric ratio of 88:12 for the formation of **3a** at 60 °C (entry 14), whereas under photochemical conditions at −20 °C the selectivity improved to 97:3 (entry 6). This increase in enantioselectivity may arise from two contributing factors: (1) a thermal effect, in which lowering the temperature increases the ratio $\mathbf{k_S/k_R}$ from inner-sphere pathways and thereby enhances selectivity, and (2) selective suppression at low temperature of the racemic radical–polar crossover (SET) pathway relative to the enantioselective inner-sphere radical

manifold. To assess the relative importance of these contributions, we first estimated the expected temperature effect as follows:

Based on Eyring equation $k = \frac{k_B T}{h} e^{-\Delta G^{\ddagger}/RT}$, the enantioselectivity is related to:

$$\ln\left(\frac{k_S}{k_R}\right) = \frac{\Delta\Delta G^{\ddagger}}{RT}$$

From an enantiomeric ratio of 88:12 at 60 °C, a $\Delta\Delta G^{\ddagger}$ of 1.32 kcal/mol can be estimated.

Accordingly, at −20 °C we would expect an enantiomeric ratio of 93:7, significantly lower than the experimentally observed selectivity of 97:3. Therefore, the additional enhancement in enantioselectivity at low temperature likely also reflects selective suppression of the unselective SET pathway.

**Table S3.** Optimization of additives as *N*-radical precursor[a]

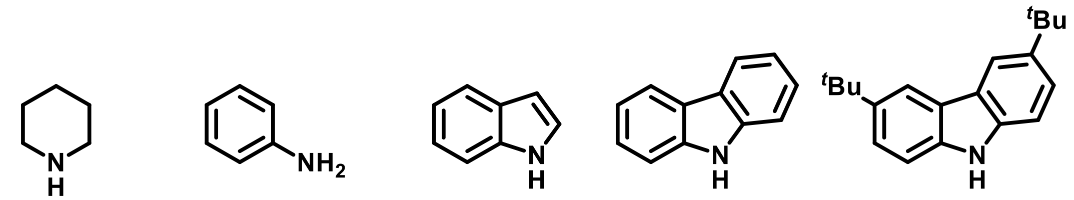

| 4a | 4b | 4c | 4d | 4e |
|---|---|---|---|---|
| 23% yield | 52% yield | 52% yield | 74% yield | 96% yield |
| 96:4 er | 95:5 er | 91:9 er | 96:4 er | 97:3 er |

[a]Reaction conditions: CuCl (0.010 mmol, 10 mol%), $NaBAr^F_4$ (0.015 mmol, 15 mol%), ligand **L6** (0.015 mmol, 15 mol%), specified additive (0.010 mmol, 10 mol%), $(^tBuO)_2$ (0.20 mmol, 2.0 equiv), 427 nm irradiation, –20 °C in a 1:1 mixture of PhMe and PhCl (0.2 M).

*Note*: The following sterically hindered carbazoles were evaluated as reaction additives to suppress undesired dimerization. However, these additives resulted in lower product yields, likely because steric congestion impedes productive interaction of the carbazole N-radical with the alkylboron substrate for C–B bond cleavage.

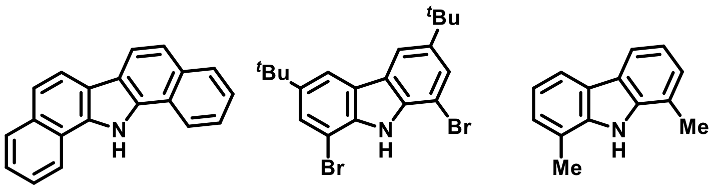

| 4f | 4g | 4h |
|---|---|---|
| 17% yield | 41% yield | 11% yield |
| 90:10 er | 85:15 er | 87:13 er |

*Note*: We propose that additive **4e** plays a beneficial role for three reasons:

(1) In our previous work,[1,2] as well as in related studies from other groups,[3] the N-radical precursor is not efficiently turned over following N-radical-mediated C–B bond cleavage. For example, no reaction is observed between independently prepared aniline–BPin and $^t$BuOH. As a result, amine additives are often required in (super)stoichiometric amounts. We attribute this behavior to conjugation of the aniline nitrogen lone pair with the vacant p orbital on boron, which strengthens the N–B interaction and renders cleavage of this bond difficult. In contrast, in carbazole-derived additives the nitrogen lone pair is incorporated into the aromatic π-system, which weakens this conjugation and facilitates the turnover of **4e**.

(2) Computations further indicate that **4e** is better energetically matched for selective HAT activation than the less effective N–H sources, such as **4c** and **4d**. Specifically, **4e** is computed to have a weaker N–H bond than **4c** and **4d** by 3.0 and 1.7 kcal/mol, respectively (see table below), and the corresponding HAT barriers with $^t$BuO• are lower by similar amounts (2.8 and 0.7 kcal/mol, respectively).

*all M062X/6-31+G**

**4c** **4d** **4e** **substrate**

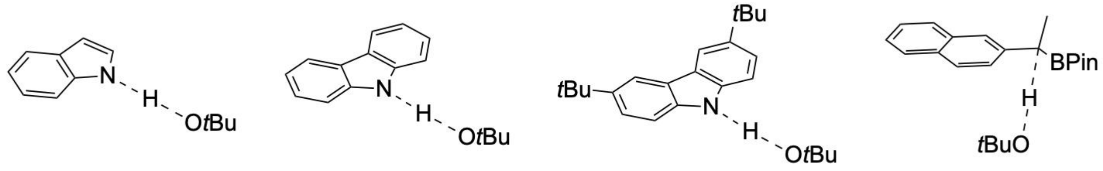


| | 4c | 4d | 4e | substrate |
|---|---|---|---|---|
| BDE (kcal/mol) | 92 | 90.7 | 89.0 | **81.9** |
| $\Delta G^{\ddagger}_{HAT}$ | 15.6 | 13.5 | **12.8** | 15.1 |

(3) The reduced donor ability of the carbazole nitrogen (due to conjugation) makes coordination to Cu less favorable, thereby minimizing unproductive catalyst binding or alternative coordination modes.

(4) The $^t$Bu substituents in **4e** also improve its solubility relative to parent carbazole at −20 °C, which likely further enhances its effectiveness under the reaction conditions.

**Table S4.** Representative Ligand Optimization[a]

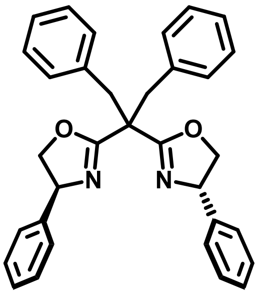

**L1**
74% yield
77:23 er

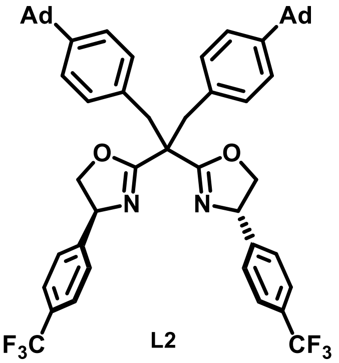


**L2**
59% yield
racemic

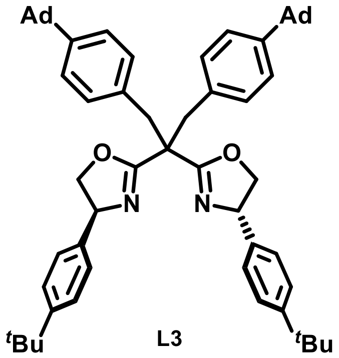


**L3**
48% yield
65:35 er

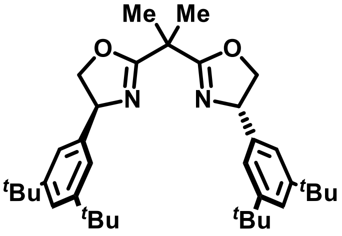


**L4**
80% yield
81:19 er

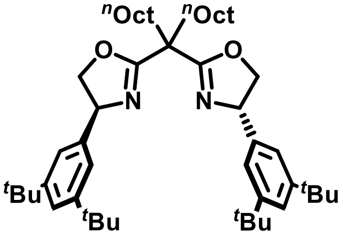


**L5**
90% yield
88:12 er

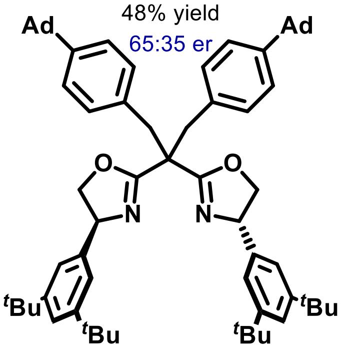


**L6**
96% yield
97:3 er

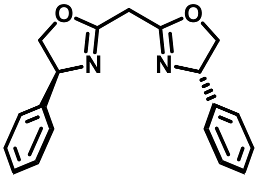

**L7**
72% yield
77:23 er

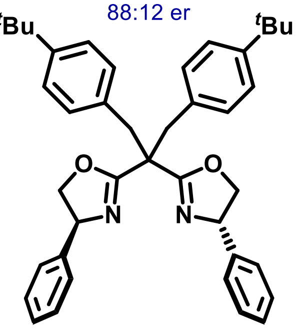


**L8**
80% yield
74:26 er

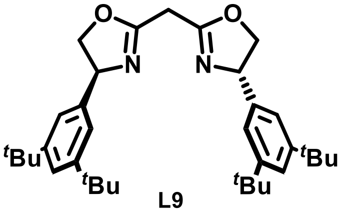


**L9**
33% yield
66:34 er

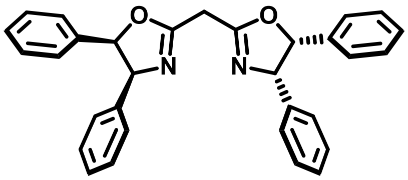

**L10**
38% yield
70:30 er

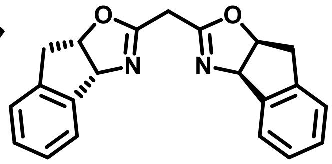

**L11**
56% yield
56:44 er

[a]Reaction conditions: CuCl (0.010 mmol, 10 mol%), $NaBAr^F_4$ (0.015 mmol, 15 mol%), specified ligand (0.015 mmol, 15 mol%), additive **4e** (0.010 mmol, 10 mol%), $(^tBuO)_2$ (0.20 mmol, 2.0 equiv), 427 nm irradiation, –20 °C in a 1:1 mixture of toluene and PhCl (0.2 M).

*Note*: Ligand **L6** was particularly effective in delivering high selectivity, likely owing to two key design features:

(1) The elongated substituents at the meso positions are oriented toward the metal center, as also supported by the X-ray structure (Figure 3B-C); extended groups such as adamantyl may therefore help enforce productive ligand–substrate interactions.

(2) The distal bulky alkyl substituents may promote favorable noncovalent interactions with the incoming radical, thereby further enhancing stereocontrol.

## 3. Synthesis and Characterization of Starting Materials

The following alkylboronic pinacol esters were prepared according to reported protocols:

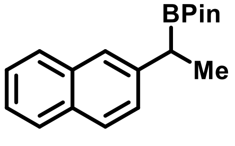


**1a**[1]

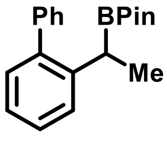


**1b**[1]

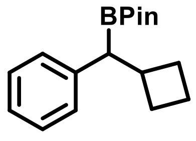


**1e**[2]

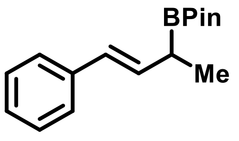


**1q**[4]

*tert*-butyl 3-(naphthalen-2-yl(4,4,5,5-tetramethyl-1,3,2-dioxaborolan-2-yl)methyl)azetidine-1-carboxylate (**1c**)

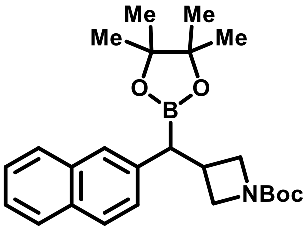


Prepared according to literature procedure on a 10.0 mmol scale.[5] Purification by column chromatography (100 g $SiO_2$) afforded the titled compound (1.59 g, 38%) as a white solid.

**$^{1}$H NMR** (400 MHz; $CDCl_3$) δ 7.85–7.70 (m, 3H), 7.59 (s, 1H), 7.50–7.36 (m, 2H), 7.32 (d, $J$ = 8.6 Hz, 1H), 4.21–4.09 (m, 1H), 3.89–3.80 (m, 1H), 3.70 (dd, $J$ = 8.7, 5.6 Hz, 1H), 3.55 (dd, $J$ = 8.7, 5.6 Hz, 1H), 3.19–2.97 (m, 1H), 2.72 (d, $J$ = 11.6 Hz, 1H), 1.43 (s, 9H), 1.22 (s, 6H), 1.18 (s, 6H).

**$^{13}$C NMR** (101 MHz; $CDCl_3$) δ 156.58, 138.03, 133.85, 132.08, 128.27, 127.67, 127.59, 127.13, 126.55, 126.04, 125.31, 83.86, 79.23, 55.46, 54.16, 37.84, 31.25, 28.54, 24.83, 24.59.

**HRMS** (DART) m/z: $[M+H]^+$ calcd. for $C_{25}H_{35}BNO_4^+$: 424.2654. Found: 424.2668.

2-phenyl-4-(1-(4,4,5,5-tetramethyl-1,3,2-dioxaborolan-2-yl)butyl)thiazole (**1d**)

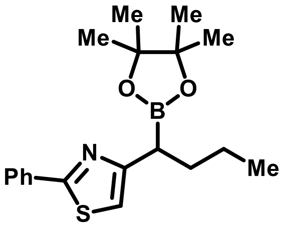


Prepared according to literature procedure on a 10.0 mmol scale.[6] Purification by column chromatography (100 g $SiO_2$) afforded the titled compound (1.83 g, 53%) as a white solid.

**$^{1}$H NMR** (400 MHz; $CDCl_3$) δ 7.95–7.91 (m, 2H), 7.42–7.35 (m, 3H), 6.91 (s, 1H), 2.72–2.68 (m, 1H), 1.90–1.76 (m, 2H), 1.42–1.34 (m, 2H), 1.26 (s, 6H), 1.26 (s, 6H), 0.93 (t, *J* = 7.3 Hz, 3H).
**$^{13}$C NMR** (101 MHz; $CDCl_3$) 166.78, 159.73, 134.33, 129.60, 128.88, 126.61, 112.34, 83.58, 33.56, 24.87, 24.80, 22.49, 14.35.
**HRMS** (DART) m/z: $[M+H]^+$ calcd. for $C_{19}H_{27}BNO_2S^+$: 344.1850. Found: 344.1846.

2-(1-(benzo[*b*]thiophen-5-yl)-3-phenylpropyl)-4,4,5,5-tetramethyl-1,3,2-dioxaborolane (**1f**)

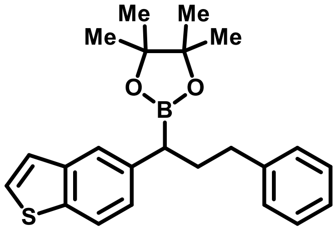


Prepared according to literature procedure on a 10.0 mmol scale.[6] Purification by column chromatography (100 g $SiO_2$) afforded the titled compound (2.89 g, 76%) as a white solid.
**$^{1}$H NMR** (400 MHz, $CDCl_3$) δ 7.72 (d, *J* = 8.3 Hz, 1H), 7.61 (s, 1H), 7.34 (d, *J* = 8.3 Hz, 1H), 7.23–7.15 (m, 4H), 7.14–7.06 (m, 3H), 2.56–2.49 (m, 2H), 2.45–2.38 (m, 1H), 2.21–2.11 (m, 1H), 2.03–1.93 (m, 1H), 1.16 (s, 6H), 1.14 (s, 6H).
**$^{13}$C NMR** (101 MHz; $CDCl_3$) δ 142.67, 140.21, 139.19, 136.99, 128.64, 128.37, 126.24, 125.78, 125.55, 123.94, 123.20, 122.37, 83.53, 35.52, 34.74, 31.95, 24.83, 24.75.
**HRMS** (DART) m/z: $[M+H]^+$ calcd. for $C_{23}H_{28}BO_2S^+$: 379.1898. Found: 379.1886.

2-(1-(benzofuran-5-yl)butyl)-4,4,5,5-tetramethyl-1,3,2-dioxaborolane (**1g**)

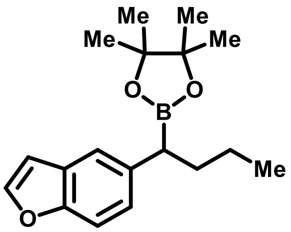


Prepared according to literature procedure on a 10.0 mmol scale.[6] Purification by column chromatography (100 g $SiO_2$) afforded the titled compound (1.46 g, 49%) as a colorless oil.
**$^{1}$H NMR** (400 MHz, $CDCl_3$) δ 7.57–7.55 (m, 1H), 7.41 (s, 1H), 7.38 (d, *J* = 8.5 Hz, 1H), 7.15 (d, *J* = 8.5 Hz, 1H), 6.71–6.68 (m, 1H), 2.43–2.35 (m, 1H), 1.90–1.80 (m, 1H), 1.71–1.61 (m, 1H), 1.32–1.24 (m, 2H), 1.21 (s, 6H), 1.19 (s, 6H), 0.89 (t, *J* = 7.3 Hz, 3H).

**$^{13}$C NMR** (101 MHz; $CDCl_3$) δ 153.44, 144.87, 137.97, 127.61, 125.10, 120.49, 111.02, 106.65, 83.36, 35.49, 24.78, 24.74, 22.47, 14.28.

**HRMS** (DART) m/z: $[M+H]^+$ calcd. for $C_{18}H_{26}BO_3^+$: 301.1970. Found: 301.1976.

4,4,5,5-tetramethyl-2-(4-(2-(naphthalen-2-yl)-2-(4,4,5,5-tetramethyl-1,3,2-dioxaborolan-2-yl)ethyl)phenyl)-1,3,2-dioxaborolane (**1h**)

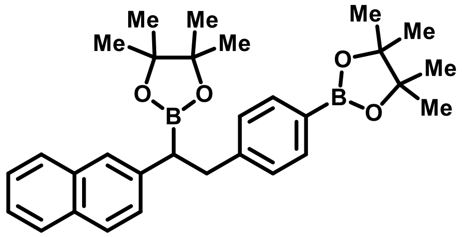


Prepared according to literature procedure on a 10.0 mmol scale.[5] Purification by column chromatography (100 g $SiO_2$) afforded the titled compound (3.17 g, 65%) as a white solid.

**$^1$H NMR** (400 MHz; $CDCl_3$) δ 7.79–7.71 (m, 3H), 7.68–7.63 (m, 3H), 7.44–7.36 (m, 3H), 7.22 (d, $J$ = 8.1 Hz, 2H), 3.29 (dd, $J$ = 13.6, 9.3 Hz, 1H), 3.06 (dd, $J$ = 13.6, 7.0 Hz, 1H), 2.85 (dd, $J$ = 9.3, 7.0 Hz, 1H), 1.33 (s, 12H), 1.12 (s, 6H), 1.11 (s, 6H).

**$^{13}$C NMR** (101 MHz; $CDCl_3$) δ 145.31, 140.23, 134.81, 133.93, 131.98, 128.43, 127.90, 127.68, 127.67, 127.55, 126.45, 125.79, 125.04, 125.01, 83.74, 83.68, 38.90, 25.04, 24.98, 24.73.

**HRMS** (DART) m/z: $[M+H]^+$ calcd. for $C_{30}H_{39}B_2O_4^+$: 485.3029. Found: 485.3036.

2,2'-(1-(naphthalen-2-yl)butane-1,4-diyl)bis(4,4,5,5-tetramethyl-1,3,2-dioxaborolane) (**1i**)

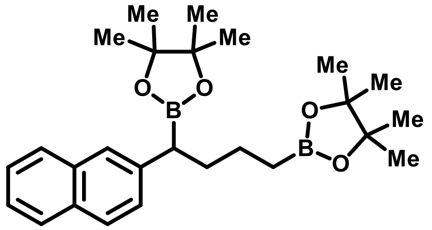


Prepared according to literature procedure on a 10.0 mmol scale.[5] Purification by column chromatography (100 g $SiO_2$) afforded the titled compound (1.83 g, 42%) as a white solid.

**$^1$H NMR** (400 MHz; $CDCl_3$) δ 7.80–7.74 (m, 2H), 7.73 (d, $J$ = 8.5 Hz, 1H), 7.63 (s, 1H), 7.44–7.40 (m, 1H), 7.40–7.35 (m, 2H), 2.54–2.46 (m, 1H), 1.98–1.89 (m, 1H), 1.84–1.75 (m, 1H), 1.46–1.37 (m, 2H), 1.22 (s, 12H), 1.20 (s, 6H), 1.18 (s, 6H), 0.87 – 0.72 (m, 2H).

**$^{13}$C NMR** (101 MHz; $CDCl_3$) δ 141.18, 133.95, 131.86, 127.71, 127.66, 127.63, 127.60, 126.33, 125.65, 124.79, 83.40, 82.96, 35.11, 32.64, 24.95, 24.94, 24.77, 24.73, 23.79, 11.37.

**HRMS** (DART) m/z: $[M+H]^+$ calcd. for $C_{26}H_{39}B_2O_4^+$: 437.3029. Found: 437.3017.

2-(3-chloro-1-(naphthalen-2-yl)propyl)-4,4,5,5-tetramethyl-1,3,2-dioxaborolane (**1j**)

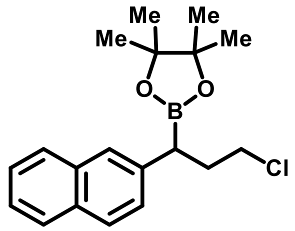


Prepared according to literature procedure on a 10.0 mmol scale.[5] Purification by column chromatography (100 g $SiO_2$) afforded the titled compound (2.05 g, 62%) as a colorless oil.

**$^1$H NMR** (400 MHz; $CDCl_3$) δ 7.84–7.70 (m, 3H), 7.69–7.60 (m, 1H), 7.48–7.38 (m, 2H), 7.36 (d, $J$ = 8.5 Hz, 1H), 3.58–3.48 (m, 1H), 3.46–3.37 (m, 1H), 2.80–2.68 (m, 1H), 2.40–2.31 (m, 1H), 2.31–2.18 (m, 1H), 1.22 (s, 6H), 1.19 (s, 6H).

**$^{13}$C NMR** (101 MHz; $CDCl_3$) δ 139.17, 133.92, 132.08, 128.24, 127.70, 127.63, 127.31, 126.89, 125.99, 125.25, 83.80, 44.48, 35.07, 29.57, 24.79, 24.73.

**HRMS** (DART) m/z: $[M+H]^+$ calcd. for $C_{19}H_{25}BClO_2^+$: 331.1631. Found: 331.1642.

*tert*-butyl 3-(benzofuran-5-yl(4,4,5,5-tetramethyl-1,3,2-dioxaborolan-2-yl)methyl)azetidine-1-carboxylate (**1k**)

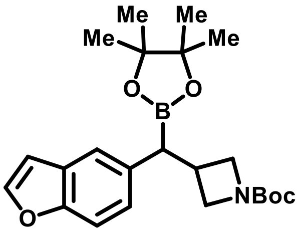


Prepared according to literature procedure on a 10.0 mmol scale.[6] Purification by column chromatography (100 g $SiO_2$) afforded the titled compound (2.11 g, 51%) as a white solid.

**$^1$H NMR** (400 MHz; $CDCl_3$) δ 7.60–7.52 (m, 1H), 7.41–7.33 (m, 2H), 7.09 (d, $J$ = 8.5 Hz, 1H), 6.74–6.65 (m, 1H), 4.17–4.07 (m, 1H), 3.86–3.74 (m, 1H), 3.67–3.61 (m, 1H), 3.55–3.50 (m, 1H), 3.04–2.95 (m, 1H), 2.61 (d, $J$ = 11.6 Hz, 1H), 1.42 (s, 9H), 1.21 (s, 6H), 1.17 (s, 6H).

**$^{13}$C NMR** (101 MHz; $CDCl_3$) δ 156.61, 153.70, 145.19, 134.84, 127.88, 124.88, 120.64, 111.46, 106.59, 83.78, 79.21, 55.35, 54.02, 37.31, 31.86, 28.55, 24.84, 24.60.

**HRMS** (DART) m/z: $[M+H]^+$ calcd. for $C_{23}H_{33}BNO_5^+$: 414.2446. Found: 414.2450.

2-(3-(2,3-dihydrobenzofuran-5-yl)-1-(naphthalen-2-yl)propyl)-4,4,5,5-tetramethyl-1,3,2-dioxaborolane (**1l**)

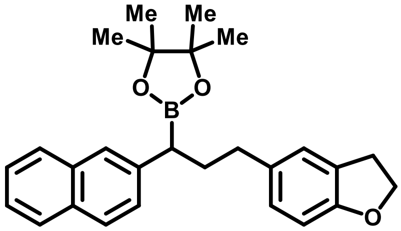


Prepared according to literature procedure on a 10.0 mmol scale.[5] Purification by column chromatography (100 g $SiO_2$) afforded the titled compound (2.50 g, 60%) as a white solid.

**$^1$H NMR** (400 MHz; $CDCl_3$) δ 7.85–7.74 (m, 3H), 7.68 (s, 1H), 7.49–7.38 (m, 3H), 7.01 (s, 1H), 6.91 (d, $J$ = 8.3 Hz, 1H), 6.71 (d, $J$ = 8.3 Hz, 1H), 4.54 (t, $J$ = 8.6 Hz, 2H), 3.17 (t, $J$ = 8.6 Hz, 2H), 2.63–2.51 (m, 3H), 2.29–2.20 (m, 1H), 2.14–2.01 (m, 1H), 1.23 (s, 6H), 1.21 (s, 6H).

**$^{13}$C NMR** (101 MHz; $CDCl_3$) δ 158.24, 140.71, 134.62, 133.93, 131.91, 127.96, 127.88, 127.65, 127.57, 127.51, 126.93, 126.45, 125.78, 125.09, 124.94, 108.94, 83.51, 71.20, 34.97, 34.74, 32.21, 29.93, 24.80, 24.71.

**HRMS** (DART) m/z: $[M+H]^+$ calcd. for $C_{27}H_{32}BO_3^+$: 415.2439. Found: 415.2440.

2-(1-(benzo[*b*]thiophen-5-yl)butyl)-4,4,5,5-tetramethyl-1,3,2-dioxaborolane (**1m**)

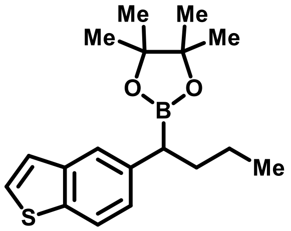


Prepared according to literature procedure on a 10.0 mmol scale.[6] Purification by column chromatography (100 g $SiO_2$) afforded the titled compound (1.66 g, 52%) as a colorless oil.

**$^1$H NMR** (400 MHz; $CDCl_3$) δ 7.75 (d, $J$ = 8.3 Hz, 1H), 7.65 (s, 1H), 7.37 (d, $J$ = 5.4 Hz, 1H), 7.26 (d, $J$ = 5.4 Hz, 1H), 7.22 (d, $J$ = 8.3 Hz, 1H), 2.47–2.38 (m, 1H), 1.92–1.83 (m, 1H), 1.73–1.63 (m, 1H), 1.35–1.26 (m, 2H), 1.21 (s, 6H), 1.19 (s, 6H), 0.93–0.86 (m, 3H).

**$^{13}$C NMR** (101 MHz; $CDCl_3$) δ 140.16, 139.75, 136.82, 126.14, 125.61, 123.94, 123.11, 122.23, 83.43, 35.20, 24.79, 24.76, 22.49, 14.27.

**HRMS** (DART) m/z: $[M+H]^+$ calcd. for $C_{18}H_{26}BO_2S^+$: 317.1741. Found: 317.1432.

2-(1-(6-bromonaphthalen-2-yl)ethyl)-4,4,5,5-tetramethyl-1,3,2-dioxaborolane (**1n**)

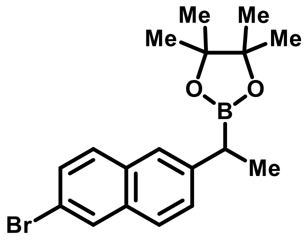


Prepared according to literature procedure on a 10.0 mmol scale.[6] Purification by column chromatography (100 g $SiO_2$) afforded the titled compound (1.35 g, 37%) as a colorless oil.

**$^1$H NMR** (400 MHz; $CDCl_3$) δ 7.87–7.82 (m, 1H), 7.58–7.51 (m, 3H), 7.40 (dd, *J* = 8.7, 2.1 Hz, 1H), 7.32 (dd, *J* = 8.7, 2.1 Hz, 1H), 2.52 (d, *J* = 7.4 Hz, 1H), 1.34 (d, *J* = 7.4 Hz, 3H), 1.12 (s, 6H), 1.11 (s, 6H).

**$^{13}$C NMR** (101 MHz; $CDCl_3$) δ 143.35, 132.94, 132.40, 129.68, 129.31, 129.11, 128.42, 126.91, 125.32, 118.67, 83.60, 24.78, 24.73, 16.77.

**HRMS** (DART) m/z: $[M+H]^+$ calcd. for $C_{18}H_{23}BBrO_2^+$: 361.0969. Found: 361.0970.

5-(cyclobutyl(4,4,5,5-tetramethyl-1,3,2-dioxaborolan-2-yl)methyl)-1-tosyl-1*H*-indole (**1o**)

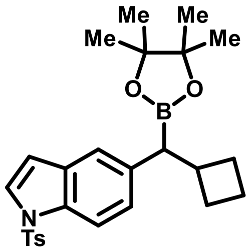


Prepared according to literature procedure on a 10.0 mmol scale.[6] Purification by column chromatography (100 g $SiO_2$) afforded the titled compound (3.25 g, 70%) as a white solid.

**$^1$H NMR** (400 MHz; $CDCl_3$) δ 7.85 (d, *J* = 8.5 Hz, 1H), 7.76 (d, *J* = 6.4 Hz, 2H), 7.50–7.46 (m, 1H), 7.31 (s, 1H), 7.19 (d, *J* = 6.4 Hz, 2H), 7.15 (d, *J* = 8.5 Hz, 1H), 6.60–6.55 (m, 1H), 2.81–2.69 (m, 1H), 2.39 (d, *J* = 13.0 Hz, 1H), 2.31 (s, 3H), 2.19–2.08 (m, 1H), 1.89–1.69 (m, 4H), 1.59–1.49 (m, 1H), 1.20 (s, 6H), 1.17 (s, 6H).

**$^{13}$C NMR** (101 MHz; $CDCl_3$) δ 144.79, 137.01, 135.57, 133.11, 131.06, 129.88, 126.92, 126.07, 125.67, 120.70, 113.19, 109.17, 83.33, 39.89, 38.75, 28.87, 28.64, 24.78, 24.65, 21.61, 18.23.

**HRMS** (DART) m/z: $[M+H]^+$ calcd. for $C_{26}H_{33}BNO_4S^+$: 466.2218. Found: 466.2232.

1-(3-(naphthalen-2-yl)-3-(4,4,5,5-tetramethyl-1,3,2-dioxaborolan-2-yl)propyl)-1*H*-pyrrole (**1p**)

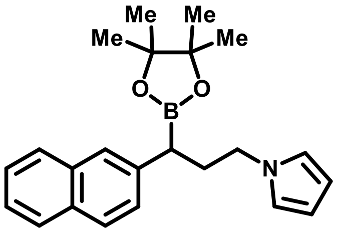


Prepared according to literature procedure on a 10.0 mmol scale.[5] Purification by column chromatography (100 g $SiO_2$) afforded the titled compound (2.20 g, 61%) as a white solid.

**$^1$H NMR** (400 MHz; $CDCl_3$) δ 7.74–7.66 (m, 3H), 7.55 (s, 1H), 7.39–7.31 (m, 2H), 7.28 (d, *J* = 8.5 Hz, 1H), 6.54 (d, *J* = 2.3 Hz, 2H), 6.06 (d, *J* = 2.3 Hz, 2H), 3.81–3.67 (m, 2H), 2.41–2.27 (m, 2H), 2.23–2.12 (m, 1H), 1.12 (s, 6H), 1.09 (s, 6H).

**$^{13}$C NMR** (101 MHz; $CDCl_3$) δ 139.51, 133.87, 132.00, 128.19, 127.67, 127.56, 127.23, 126.60, 125.97, 125.18, 120.64, 107.95, 83.70, 48.69, 33.85, 29.42, 24.75, 24.69.

**HRMS** (DART) m/z: $[M+H]^+$ calcd. for $C_{23}H_{29}BNO_2^+$: 362.2286. Found: 362.2289.

(*E*)-4,4,5,5-tetramethyl-2-(1-phenyloct-1-en-3-yl)-1,3,2-dioxaborolane (**1r**)

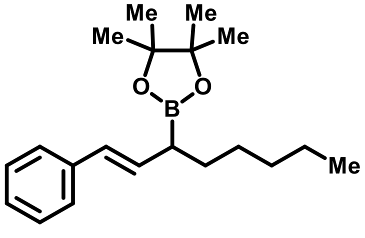


Prepared according to literature procedure on a 10.0 mmol scale.[7] Purification by column chromatography (100 g $SiO_2$) afforded the titled compound (2.06 g, 66%) as a colorless oil.

**$^1$H NMR** (400 MHz; $CDCl_3$) δ 7.33–7.18 (m, 4H), 7.14–7.07 (m, 1H), 6.32 (d, *J* = 15.6 Hz, 1H), 6.17 (dd, *J* = 15.6, 8.9 Hz, 1H), 2.01–1.89 (m, 1H), 1.66–1.52 (m, 2H), 1.52–1.40 (m, 2H), 1.36–1.24 (m, 4H), 1.20 (s, 12H), 0.87–0.79 (m, 3H).

**$^{13}$C NMR** (101 MHz; $CDCl_3$) δ 138.41, 132.21, 129.04, 128.51, 126.60, 126.00, 83.34, 32.00, 30.94, 28.99, 24.88, 24.75, 22.71, 14.21.

**HRMS** (DART) m/z: $[M+H]^+$ calcd. for $C_{20}H_{32}BO_2^+$: 315.2490. Found: 315.2482.

($E$)-2-(5-(1,3-dioxan-2-yl)-1-phenylpent-1-en-3-yl)-4,4,5,5-tetramethyl-1,3,2-dioxaborolane (**1s**)

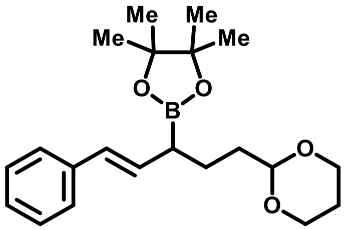


Prepared according to literature procedure on a 10.0 mmol scale.[7] Purification by column chromatography (100 g $SiO_2$) afforded the titled compound (1.44 g, 40%) as a colorless oil.

**$^1$H NMR** (400 MHz; $CDCl_3$) δ 7.32 (d, $J$ = 7.7 Hz, 2H), 7.28–7.24 (m, 2H), 7.15 (t, $J$ = 6.7 Hz, 1H), 6.36 (d, $J$ = 15.8 Hz, 1H), 6.17 (dd, $J$ = 15.8, 9.0 Hz, 1H), 4.55–4.46 (m, 1H), 4.11–4.02 (m, 2H), 3.81–3.69 (m, 2H), 2.10–2.00 (m, 1H), 1.97–1.90 (m, 1H), 1.77–1.65 (m, 2H), 1.65–1.53 (m, 2H), 1.34–1.29 (m, 1H), 1.23 (s, 12H).

**$^{13}$C NMR** (101 MHz; $CDCl_3$) δ 138.28, 131.53, 129.62, 128.48, 126.66, 126.06, 102.52, 83.44, 67.03, 34.79, 25.99, 25.17, 25.10, 24.90, 24.79.

**HRMS** (DART) m/z: $[M+H]^+$ calcd. for $C_{21}H_{32}BO_4^+$: 359.2388. Found: 359.2388.

## 5. Synthesis and Characterization of C(sp$^3$)–O Coupled Products

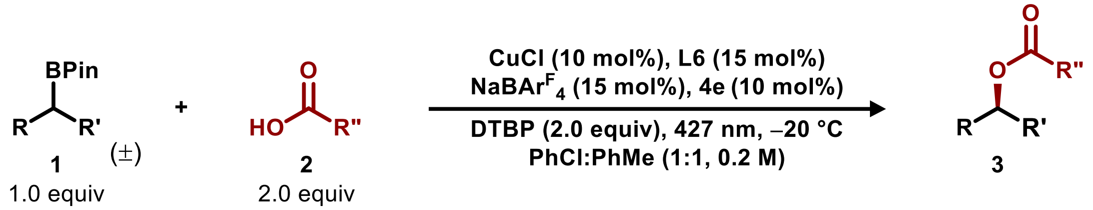


**General Procedure A:**

Inside a nitrogen-filled glovebox, an oven-dried reaction tube (Fisherbrand, 16 × 125 mm, catalog no. 1495935A) equipped with a magnetic stir bar was charged with CuCl (5.0 mg, 0.050 mmol, 10 mol%), $NaBAr^F{}_4$ (66.5 mg, 0.075 mmol, 15 mol%), and **L6** (73.5 mg, 0.075 mmol, 15 mol%) followed by the addition of a 1:1 mixture of PhCl and PhMe (2.5 mL). The reaction mixture was stirred at room temperature for 10 min before the addition of **4e** (14.0 mg ,0.050 mmol, 10 mol%), alkylborane **1** (0.50 mmol, 1.0 equiv), and carboxylic acid **2** (1.00 mmol, 2.0 equiv). The reaction mixture was stirred for 10 min before di-*tert*-butyl peroxide (183 µL, 1.00 mmol, 2.0 equiv) was added in one portion. The reaction tube was sealed with a screw cap (Kimble Chase Open Top S/T Closure, catalog no. 73804-15425) containing a PTFE septum (Thermo Scientific, catalog no. B7995-15), taken outside of the glovebox, placed in a –20 °C freezer and irradiated with 427 nm light (Kessil P160L) with stirring.

After the reaction mixture had stirred for 16 h, the screw cap was removed from the reaction tube, and the reaction mixture was concentrated *in vacuo* with the aid of a rotary evaporator. The resulting crude material was purified by reverse-phase (C18) column chromatography. The enantiomeric ratio was determined by chiral HPLC analysis.

*Note*: General procedures were followed unless otherwise noted. Synthesis of **3ac**, **3ad**, **3af**, and **3al** were carried out DCE (2.5 mL) due to limited solubility of the starting material under standard conditions. Synthesis of **3j**, **3n**, **3t**, and **3v** were carried out at 25 °C for higher conversion.

*Note*: The racemic samples used for HPLC analysis was synthesized using 0.10 mmol of alkylborane **1** and racemic (±)-**L6** ligand (a 1:1 mixture of the two enantiomers of **L6**). The resulting mixture was purified by thin layer chromatography.

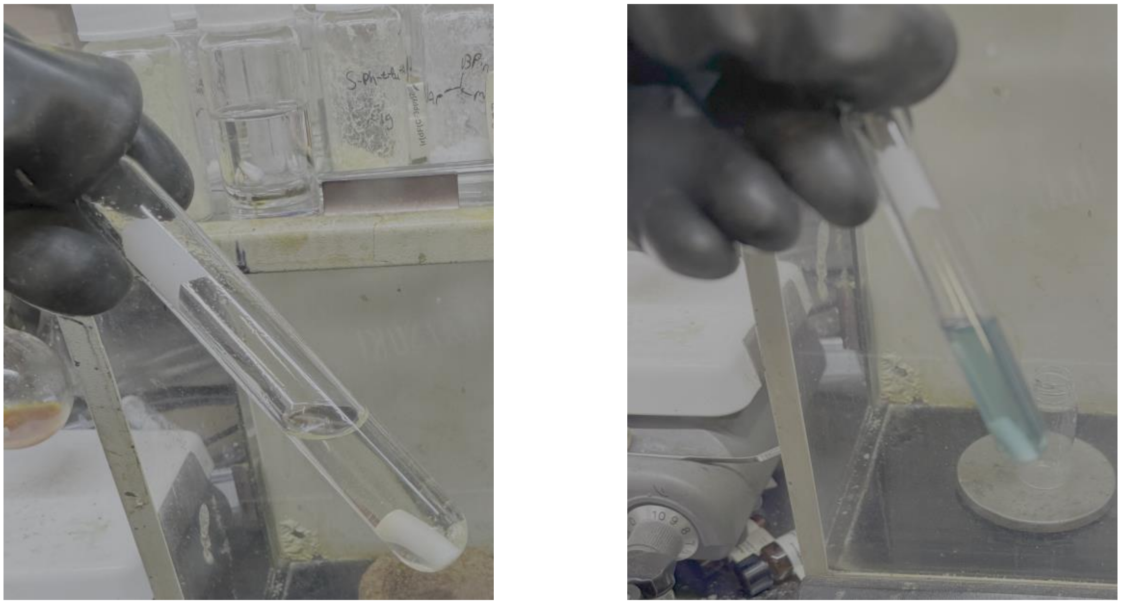

(a) (b)

**Figure S1.** (a) (left) Image of the reaction tube charged with CuCl, **L6**, $NaBAr^F{}_4$, and reaction solvent (1:1 PhMe:PhCl); (b) (right) Image of the reaction tube after the addition of **1a**, **2a**, **4e**, and $(^tBuO)_2$, resulting in a light green solution.

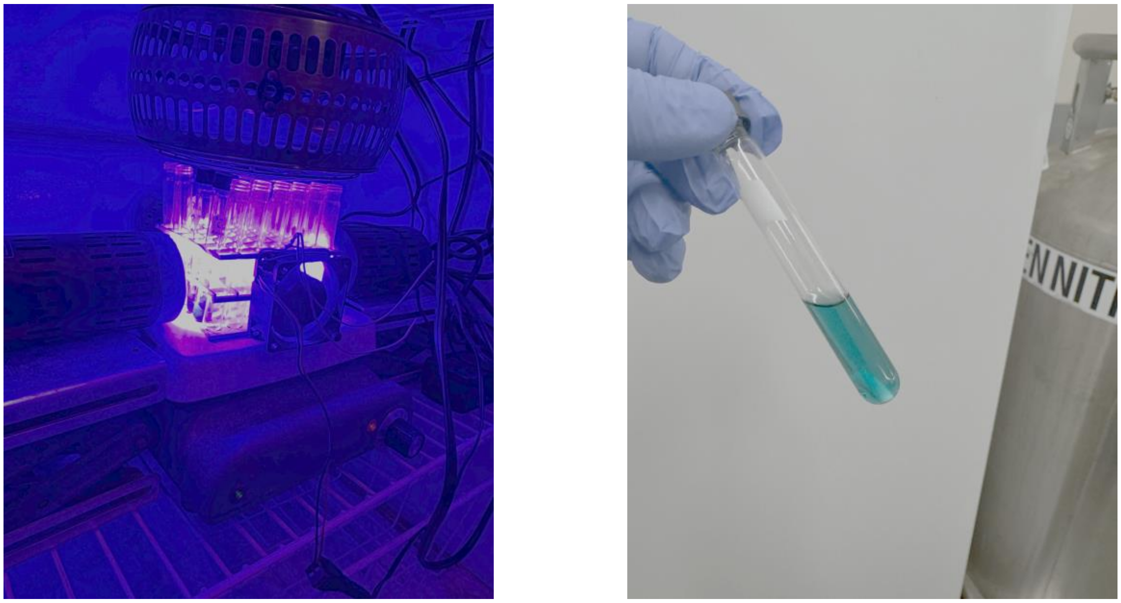

(a) (b)

**Figure S2.** (a) (left) image of the reaction mixture under irradiation in a −20 °C freezer; (b) (right) image of reaction tube after irradiation at −20 °C for 16 h, resulting in a green solution.

(*S*)-1-(naphthalen-2-yl)ethyl benzoate (**3a**)

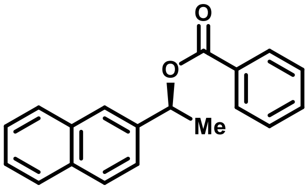


The residue was purified by reverse-phase column chromatography on C18 stationary phase (100 g cartridge, 20% to 100% MeCN in $H_2O$) to yield the titled compound (131 mg, 95%, 97:3 er) as a white solid.

**$^{1}$H NMR** (400 MHz; $CDCl_3$) δ 8.16–8.09 (m, 2H), 7.93–7.80 (m, 4H), 7.63–7.53 (m, 2H), 7.52–7.39 (m, 4H), 6.32 (q, *J* = 6.6 Hz, 1H), 1.78 (d, *J* = 6.6 Hz, 3H).

**$^{13}$C NMR** (101 MHz; CDCl3) δ 165.99, 139.25, 133.35, 133.19, 133.08, 130.67, 129.81, 128.57, 128.50, 128.20, 127.81, 126.37, 126.20, 125.14, 124.21, 73.20, 22.48.

**IR** (Diamond-ATR, neat) $\bar{\nu}$ ($cm^{-1}$) 3057, 2963, 1730, 1602, 1450, 1192, 1174, 1107, 965

**Specific Rotation** $[\alpha]^{27}_{D}$: 34.00 (*c* = 1.0, $CHCl_3$)

**HRMS** (APGC) m/z: $[M]^+$ calcd for $C_{19}H_{16}O_2^+$: 276.1145. Found: 276.1145

**Chiral HPLC (**Chiralcel AD-3, hexane/IPA = 90/10 over 15 min, 1.0 mL/min, 214 nm, 25 °C), 3.83 (minor), 5.15 (major)

The spectroscopic data is consistent with reported values.[8]

($S$)-1-([1,1'-biphenyl]-2-yl)ethyladamantane-1-carboxylate (**3b**)

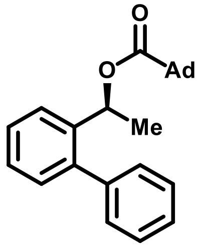


The residue was purified by reverse-phase column chromatography on C18 stationary phase (100 g cartridge, 20% to 100% MeCN in $H_2O$) to yield the titled compound (129 mg, 72%, 92:8 er) as a white solid.

*Gram-Scale Procedure*: Inside a nitrogen-filled glovebox, a 50-mL round-bottom flask equipped with a magnetic stir bar was charged CuCl (50.0 mg, 0.500 mmol, 10 mol%), **L6** (735 mg, 0.750 mmol, 15 mol%), and $NaBAr^F_4$ (665 mg, 0.750 mmol, 15 mol%) followed by the addition of a 1:1 mixture of PhCl and PhMe (25 mL). The reaction mixture was stirred at room temperature for 10 min before the addition of **4e** (140 mg, 0.500 mmol, 10 mol%), alkylborane **1b** (1.54 g, 5.00 mmol, 1.0 equiv), and carboxylic acid **2b** (1.80 g, 10.0 mmol, 2.0 equiv). The reaction mixture was stirred for 10 min before di-*tert*-butyl peroxide (1.83 mL, 10.0 mmol, 2.0 equiv) was added in one portion. The reaction flask was sealed with a rubber septum, taken outside of the glovebox, placed in a –20 °C freezer and irradiated with 427 nm light (Kessil P160L) with stirring.

After the reaction mixture had stirred for 16 h, the septum was removed from the flask, and the reaction mixture was concentrated in vacuo with the aid of a rotary evaporator. The resulting crude material was purified by reverse-phase (C18) column chromatography to yield the titled compound **3b** (1.48 g, 82%, 92:8 er) as a white solid.

**$^1$H NMR** (400 MHz; $CDCl_3$) δ 7.52 (d, $J$ = 7.8 Hz, 1H), 7.46–7.34 (m, 6H), 7.34–7.29 (m, 1H), 7.21 (d, $J$ = 7.4 Hz, 1H), 5.91 (q, $J$ = 6.3 Hz, 1H), 2.08–1.99 (m, 3H), 1.97–1.88 (m, 6H), 1.79–1.69 (m, 6H), 1.35 (d, $J$ = 6.3 Hz, 3H).
**$^{13}$C NMR** (101 MHz; $CDCl_3$) δ 176.63, 140.83, 140.56, 140.51, 130.13, 129.30, 128.36, 127.88, 127.27, 127.26, 125.02, 69.24, 40.66, 38.91, 36.66, 28.09, 22.76.
**IR** (Diamond-ATR, neat) $\bar{\nu}$ (cm$^{-1}$) 3059, 3024, 2905, 2851, 1726, 1479, 1451, 1374, 1231, 1073

**Specific Rotation** $[\alpha]^{27}_{D}$: −12.60 (*c* = 1.0, $CHCl_3$)

**HRMS** (DART) m/z: $[M+Na]^+$ calcd for $C_{25}H_{28}O_2Na^+$: 383.1982. Found: 383.1970

**Chiral HPLC (**Chiralcel AD-3, hexane/IPA = 98/2 over 10 min, 1.0 mL/min, 214 nm, 25 °C), 4.73 (major), 5.01 (minor)

(*S*)-*tert*-butyl 3-((benzoyloxy)(naphthalen-2-yl)methyl)azetidine-1-carboxylate (**3c**)

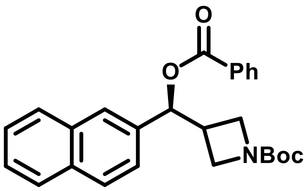


The residue was purified by reverse-phase column chromatography on C18 stationary phase (100 g cartridge, 20% to 100% MeCN in $H_2O$) to yield the titled compound (109 mg, 52%, 99:1 er) as a colorless oil.

**$^1$H NMR** (400 MHz; $CDCl_3$) δ 8.15–8.02 (m, 2H), 7.90–7.76 (m, 4H), 7.62–7.53 (m, 1H), 7.54–7.42 (m, 5H), 6.30 (d, $J$ = 8.0 Hz, 1H), 4.13–4.03 (m, 2H), 3.98–3.88 (m, 1H), 3.83–3.74 (m, 1H), 3.35–3.17 (m, 1H), 1.44 (s, 9H).

**$^{13}$C NMR** (101 MHz; $CDCl_3$) δ 165.96, 156.47, 135.58, 133.42, 133.41, 133.22, 130.02, 129.88, 128.93, 128.63, 128.22, 127.83, 126.61, 126.55, 126.17, 124.06, 79.71, 77.54, 51.74, 50.99, 34.00, 28.51.

**IR** (Diamond-ATR, neat) $\bar{\nu}$ ($cm^{-1}$): 3060, 2972, 2887, 2359, 1719, 1701, 1451, 1392, 1267, 1107

**Specific Rotation** $[\alpha]^{27}_{D}$: 22.10 ($c$ = 1.0, $CHCl_3$)

**HRMS** (DART) m/z: $[M+Na]^+$ calcd for $C_{26}H_{27}NO_4Na^+$: 440.1832. Found: 440.1847

**Chiral HPLC (**Chiralcel AD-3, hexane/IPA = 90/10 over 30 min, 2.0 mL/min, 214 nm, 25 °C), 10.83 (minor), 15.87 (major)

($S$)-1-(2-phenylthiazol-4-yl)butyl benzoate (**3d**)

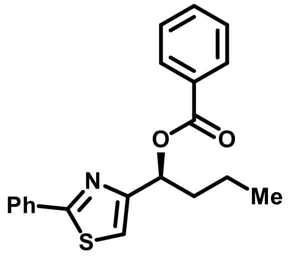


The residue was purified by reverse-phase column chromatography on C18 stationary phase (100 g cartridge, 20% to 100% MeCN in $H_2O$) to yield the titled compound (105 mg, 62%, 92:8 er) as a white solid.

**$^{1}$H NMR** (400 MHz; $CDCl_3$) δ 8.07–7.99 (m, 2H), 7.89–7.82 (m, 2H), 7.50–7.43 (m, 1H), 7.39–7.29 (m, 5H), 7.13 (s, 1H), 6.23–6.09 (m, 1H), 2.13–2.04 (m, 2H), 1.45–1.32 (m, 2H), 0.92–0.86 (m, 3H).

**$^{13}$C NMR** (101 MHz; $CDCl_3$) δ 168.37, 166.08, 156.82, 133.83, 133.17, 130.48, 130.14, 129.89, 129.02, 128.54, 126.78, 115.35, 73.06, 36.70, 18.79, 14.03.

**IR** (Diamond-ATR, neat) $\bar{\nu}$ (cm$^{-1}$): 3410, 3062, 2958, 2871, 1719, 1462, 1266, 1108, 1070, 764

**Specific Rotation** $[\alpha]^{27}_{D}$: −11.50 ($c$ = 1.0, $CHCl_3$)

**HRMS** (ESI) m/z: $[M+Na]^+$ calcd for $C_{20}H_{19}NO_2SNa^+$: 360.1034. Found: 360.1028

**Chiral HPLC (**Chiralcel AD-3, hexane/IPA = 90/10 over 10 min, 1.0 mL/min, 214 nm, 25 °C), 5.68 (minor), 6.16 (major)

(*S*)-cyclobutyl(phenyl)methyladamantane-1-carboxylate (**3e**)

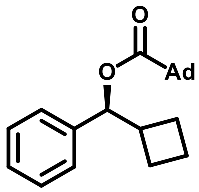


The residue was purified by reverse-phase column chromatography on C18 stationary phase (100 g cartridge, 20% to 100% MeCN in $H_2O$) to yield the titled compound (107 mg, 66%, 91:9 er) as a colorless oil.

**$^{1}$H NMR** (400 MHz; $CDCl_3$) δ 7.28–7.08 (m, 5H), 5.61 (d, *J* = 7.6 Hz, 1H), 2.79–2.55 (m, 1H), 2.02–1.81 (m, 11H), 1.81–1.71 (m, 4H), 1.70–1.55 (m, 6H).

**$^{13}$C NMR** (101 MHz; $CDCl_3$) δ 176.89, 140.02, 128.26, 127.53, 126.30, 77.97, 40.94, 40.75, 38.93, 36.58, 28.04, 24.56, 24.41, 17.88.

**IR** (Diamond-ATR, neat) $\bar{\nu}$ ($cm^{-1}$): 2931, 2905, 2852, 2360, 2341, 1726, 1696, 1452, 1231, 1075

**Specific Rotation** $[\alpha]^{27}_{D}$: –4.00 (*c* = 1.0, $CHCl_3$)

**HRMS** (DART) m/z: $[M+Na]^+$ calcd for $C_{22}H_{28}O_2Na^+$: 347.1982. Found: 347.1974

**Chiral HPLC (**Chiralcel AD-3, hexane/IPA = 98/2 over 15 min, 1.0 mL/min, 214 nm, 25 °C), 7.04 (minor), 11.13 (major)

(*S*)-1-(benzo[*b*]thiophen-5-yl)-3-phenylpropyl benzoate (**3f**)

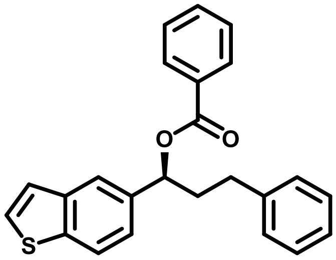


The residue was purified by reverse-phase column chromatography on C18 stationary phase (100 g cartridge, 20% to 100% MeCN in $H_2O$) to yield the titled compound (166 mg, 89%, 90:10 er) as a white solid.

**$^{1}$H NMR** (400 MHz; $CDCl_3$) δ 8.09 (d, *J* = 7.8 Hz, 2H), 7.76 (d, *J* = 7.8 Hz, 1H), 7.70 (d, *J* = 7.6 Hz, 1H), 7.54–7.46 (m, 1H), 7.43–7.35 (m, 2H), 7.34–7.22 (m, 5H), 7.21–7.11 (m, 3H), 6.44–6.32 (m, 1H), 2.83–2.68 (m, 2H), 2.60–2.46 (m, 1H), 2.45–2.31 (m, 1H).

**$^{13}$C NMR** (101 MHz; $CDCl_3$) δ 165.66, 143.84, 140.77, 139.51, 139.21, 133.20, 130.04, 129.78, 128.57, 128.46, 128.44, 126.20, 124.60, 124.45, 123.84, 122.69, 122.45, 71.98, 37.88, 31.79.

**IR** (Diamond-ATR, neat) $\bar{\nu}$ ($cm^{-1}$): 3103, 3061, 3026, 2960, 2927, 2860, 2360, 2341, 1716, 1269

**Specific Rotation** $[\alpha]^{27}_{D}$: 32.10 (*c* = 1.0, $CHCl_3$)

**HRMS** (APGC) m/z: $[M]^+$ calcd for $C_{24}H_{20}O_2S^+$: 372.1179. Found: 372.1187

**Chiral HPLC (**Chiralcel AD-3, hexane/IPA = 90/10 over 25 min, 1.0 mL/min, 228 nm, 25 °C), 7.64 (minor), 9.97 (major)

(*S*)-1-(benzofuran-5-yl)butyladamantane-1-carboxylate (**3g**)

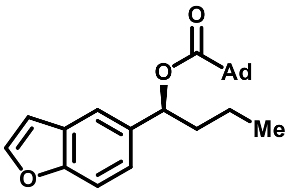


The residue was purified by reverse-phase column chromatography on C18 stationary phase (100 g cartridge, 20% to 100% MeCN in $H_2O$) to yield the titled compound (123 mg, 70%, 91:9 er) as a white solid.

**$^{1}$H NMR** (400 MHz; $CDCl_3$) δ 7.67–7.50 (m, 2H), 7.46 (d, *J* = 8.5 Hz, 1H), 7.27 (d, *J* = 8.5 Hz, 1H), 6.84–6.68 (m, 1H), 5.90–5.68 (m, 1H), 2.06–1.98 (m, 3H), 1.96–1.84 (m, 6H), 1.82–1.61 (m, 8H), 1.48–1.24 (m, 2H), 0.99–0.85 (m, 3H).

**$^{13}$C NMR** (101 MHz; $CDCl_3$) δ 176.98, 154.52, 145.45, 136.25, 127.43, 122.79, 119.21, 111.34, 106.79, 75.49, 40.84, 39.27, 38.91, 36.64, 28.09, 19.02, 13.97.

**IR** (Diamond-ATR, neat) $\bar{\nu}$ ($cm^{-1}$): 2956, 2930, 2905, 2851, 2360, 2342, 1724, 1470, 1452, 1231

**Specific Rotation** $[\alpha]^{27}_{D}$: −34.30 (*c* = 1.0, $CHCl_3$)

**HRMS** (APGC) m/z: $[M]^+$ calcd for $C_{23}H_{28}O_3^+$: 352.2033. Found: 352.2039

**Chiral HPLC (**Chiralcel AD-3, hexane/IPA = 95/5 over 7 min, 1.0 mL/min, 214 nm, 25 °C), 5.06 (minor), 6.29 (major)

(*S*)-1-(naphthalen-2-yl)-2-(4-(4,4,5,5-tetramethyl-1,3,2-dioxaborolan-2-yl)phenyl)ethyl benzoate (**3h**)

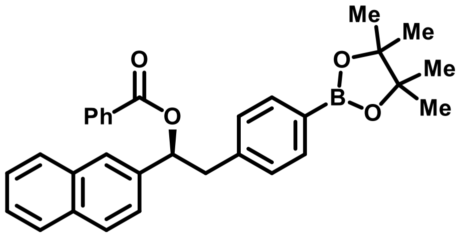


The residue was purified by reverse-phase column chromatography on C18 stationary phase (100 g cartridge, 20% to 100% MeCN in $H_2O$) to yield the titled compound (196 mg, 82%, 97:3 er) as a colorless oil.

**$^{1}$H NMR** (400 MHz; $CDCl_3$) δ 8.07 (d, *J* = 7.0 Hz, 2H), 7.84–7.74 (m, 4H), 7.68 (d, *J* = 8.0 Hz, 2H), 7.59–7.53 (m, 1H), 7.52–7.39 (m, 5H), 7.21 (d, *J* = 8.0 Hz, 2H), 6.36 (dd, *J* = 7.6, 6.0, 1H), 3.47 (dd, *J* = 13.7, 7.6 Hz, 1H), 3.32 (dd, *J* = 13.7, 6.0 Hz, 1H), 1.33 (s, 12H).

**$^{13}$C NMR** (101 MHz; $CDCl_3$) δ 165.78, 140.29, 137.52, 134.94, 133.24, 133.24, 133.12, 130.44, 129.80, 129.20, 128.52, 128.43, 128.25, 127.80, 126.30, 126.21, 125.89, 124.44, 83.85, 77.48, 43.40, 25.03.

**IR** (Diamond-ATR, neat) $\bar{\nu}$ ($cm^{-1}$): 3057, 2978, 2904, 1720, 1612, 1360, 1269, 1108, 1090, 859

**Specific Rotation** $[\alpha]^{27}_{D}$: 15.20 (*c* = 1.0, $CHCl_3$)

**HRMS** (DART) m/z: $[M+Na]^+$ calcd for $C_{31}H_{31}BO_4Na^+$: 501.2208. Found: 501.2192.

**Chiral HPLC (**Chiralcel AD-3, hexane/IPA = 90/10 over 10 min, 2.0 mL/min, 220 nm, 25 °C), 3.50 (minor), 6.18 (major)

(*S*)-1-(naphthalen-2-yl)-4-(4,4,5,5-tetramethyl-1,3,2-dioxaborolan-2-yl)butyl benzoate (**3i**)

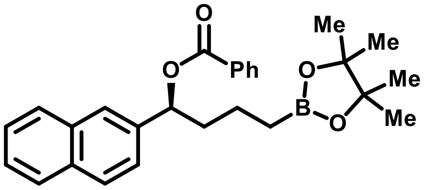

The residue was purified by reverse-phase column chromatography on C18 stationary phase (100 g cartridge, 20% to 100% MeCN in $H_2O$) to yield the titled compound (144 mg, 67%, 96:4 er) as a colorless oil.

**$^1$H NMR** (400 MHz; $CDCl_3$) δ 8.14–8.08 (m, 2H), 7.91–7.78 (m, 4H), 7.59–7.53 (m, 2H), 7.50–7.40 (m, 4H), 6.15 (dd, *J* = 7.8, 5.9 Hz, 1H), 2.27–2.11 (m, 1H), 2.09–1.96 (m, 1H), 1.68–1.42 (m, 2H), 1.23 (s, 12H), 0.92–0.81 (m, 2H).

**$^{13}$C NMR** (101 MHz; $CDCl_3$) δ 166.02, 138.45, 133.32, 133.19, 132.99, 130.75, 129.82, 128.45, 128.42, 128.20, 127.78, 126.23, 126.07, 125.77, 124.48, 83.13, 76.95, 39.11, 24.95, 24.94, 20.26, 11.13.

**IR** (Diamond-ATR, neat) $\bar{\nu}$ ($cm^{-1}$): 3061, 2979, 2905, 1720, 1693, 1451, 1379, 1317, 1272, 1110

**Specific Rotation** $[\alpha]^{27}_D$: 3.85 (*c* = 1.0, $CHCl_3$)

**HRMS** (ESI) m/z: $[M+H]^+$ calcd for $C_{27}H_{32}BO_4^+$: 431.2388. Found: 431.2392.

**Chiral HPLC (**Chiralcel AD-3, hexane/IPA = 90/10 over 15 min, 1.0 mL/min, 214 nm, 25 °C), 6.30 (minor), 10.32 (major)

(*S*)-3-chloro-1-(naphthalen-2-yl)propyl benzoate (**3j**)

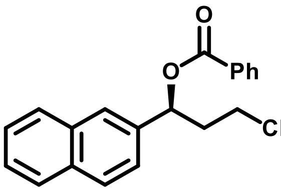


Reaction carried out at room temperature (25 °C). The residue was purified by reverse-phase column chromatography on C18 stationary phase (100 g cartridge, 20% to 100% MeCN in $H_2O$) to yield the titled compound (65 mg, 40%, 92:8 er) as a colorless oil.

**$^1$H NMR** (400 MHz; $CDCl_3$) δ 8.15–8.06 (m, 2H), 7.93–7.89 (m, 1H), 7.89–7.80 (m, 3H), 7.62–7.54 (m, 2H), 7.53–7.43 (m, 4H), 6.37 (dd, *J* = 8.0, 5.7 Hz, 1H), 3.73–3.64 (m, 1H), 3.61–3.51 (m, 1H), 2.76–2.59 (m, 1H), 2.51–2.37 (m, 1H).

**$^{13}$C NMR** (101 MHz; $CDCl_3$) δ 165.75, 136.96, 133.37, 133.32, 133.29, 130.21, 129.84, 128.86, 128.60, 128.24, 127.85, 126.54, 126.45, 125.89, 123.99, 74.13, 40.86, 39.37.

**IR** (Diamond-ATR, neat) $\bar{\nu}$ ($cm^{-1}$): 3059, 2963, 2905, 1720, 1601, 1451, 1266, 1108, 1026, 818

**Specific Rotation** $[\alpha]^{27}_D$: 35.70 (*c* = 1.0, $CHCl_3$)

**HRMS** (APGC) m/z: $[M]^+$ calcd for $C_{20}H_{17}ClO_2^+$: 324.0912. Found: 324.0919

**Chiral HPLC (**Chiralcel AD-3, hexane/IPA = 90/10 over 25 min, 1.0 mL/min, 214 nm, 25 °C), 9.79 (minor), 14.97 (major)

*tert*-butyl 3-((*S*)-(adamantane-1-carbonyl)oxy(benzofuran-5-yl)methyl)azetidine-1-carboxylate (**3k**)

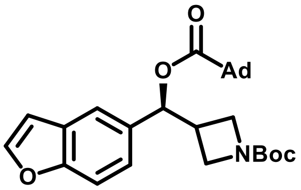


The residue was purified by reverse-phase column chromatography on C18 stationary phase (100 g cartridge, 20% to 100% MeCN in $H_2O$) to yield the titled compound (172 mg, 74%, 96:4 er) as a colorless oil.

**$^1$H NMR** (400 MHz; $CDCl_3$) δ 7.62 (d, *J* = 2.3 Hz, 1H), 7.53 (s, 1H), 7.46 (d, *J* = 8.5 Hz, 1H), 7.22 (d, *J* = 8.5 Hz, 1H), 6.75 (d, *J* = 2.3 Hz, 1H), 5.95 (d, *J* = 7.5 Hz, 1H), 3.97–3.90 (m, 2H), 3.87–3.80 (m, 1H), 3.69–3.60 (m, 1H), 3.09–2.94 (m, 1H), 2.04–1.96 (m, 3H), 1.95–1.82 (m, 6H), 1.78–1.64 (m, 6H), 1.43 (s, 9H).

**$^{13}$C NMR** (101 MHz; $CDCl_3$) δ 176.85, 156.37, 154.79, 145.79, 133.38, 127.73, 122.69, 119.42, 111.77, 106.78, 79.58, 75.88, 51.62, 50.62, 41.03, 38.89, 36.56, 34.31, 28.51, 28.00.

**IR** (Diamond-ATR, neat) $\bar{\nu}$ (cm$^{-1}$): 2972, 2906, 2852, 2360, 2341, 1701, 1452, 1403, 1224, 1129

**Specific Rotation** $[\alpha]^{27}_{D}$: −87.70 (*c* = 1.0, $CHCl_3$)

**HRMS** (DART) m/z: $[M+Na]^+$ calcd for $C_{28}H_{35}NO_5Na^+$: 488.2407. Found: 488.2420.

**Chiral HPLC (**Chiralcel AD-3, hexane/IPA = 90/10 over 10 min, 2.0 mL/min, 214 nm, 25 °C), 5.08 (minor), 8.89 (major)

(*S*)-3-(2,3-dihydrobenzofuran-6-yl)-1-(naphthalen-2-yl)propyl benzoate (**3l**)

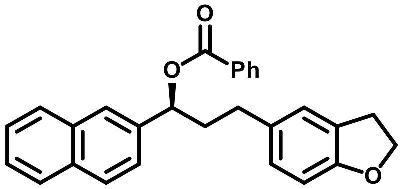


The residue was purified by reverse-phase column chromatography on C18 stationary phase (100 g cartridge, 20% to 100% MeCN in $H_2O$) to yield the titled compound (180 mg, 88%, 93:7 er) as a colorless oil.

**$^1$H NMR** (400 MHz; $CDCl_3$) δ 8.09 (d, *J* = 7.0 Hz, 2H), 7.90–7.77 (m, 4H), 7.62–7.52 (m, 2H), 7.51–7.41 (m, 4H), 7.00 (s, 1H), 6.90 (d, *J* = 9.9 Hz, 1H), 6.69 (d, *J* = 8.0 Hz, 1H), 6.16 (dd, *J* = 8.0, 5.7 Hz, 1H), 4.56–4.37 (m, 2H), 3.23–3.03 (m, 2H), 2.80–2.60 (m, 2H), 2.54–2.39 (m, 1H), 2.37–2.20 (m, 1H).

**$^{13}$C NMR** (101 MHz; $CDCl_3$) δ 165.99, 158.52, 138.05, 133.30, 133.25, 133.13, 130.55, 129.81, 128.58, 128.52, 128.25, 128.20, 127.90, 127.82, 127.25, 126.36, 126.22, 125.87, 125.03, 124.37, 109.20, 76.48, 71.28, 38.61, 31.50, 29.90.

**IR** (Diamond-ATR, neat) $\bar{\nu}$ ($cm^{-1}$): 3057, 2960, 2903, 2849, 1718, 1492, 1269, 1109, 1026, 817

**Specific Rotation** $[\alpha]^{27}_{D}$: –9.90 (*c* = 1.0, $CHCl_3$)

**HRMS** (ESI) m/z: $[M+H]^+$ calcd for $C_{28}H_{25}O_3^+$: 409.1798. Found: 409.1812

**Chiral HPLC (**Chiralcel AD-3, hexane/IPA = 90/10 over 15 min, 1.0 mL/min, 214 nm, 25 °C), 7.99 (minor), 13.07 (major)

($S$)-1-(benzo[$b$]thiophen-5-yl)butyl benzoate (**3m**)

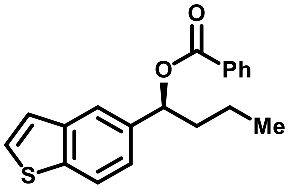


The residue was purified by reverse-phase column chromatography on C18 stationary phase (100 g cartridge, 20% to 100% MeCN in $H_2O$) to yield the titled compound (112 mg, 72%, 90:10 er) as a colorless oil.

**$^1$H NMR** (400 MHz; $CDCl_3$) δ 8.09 (d, $J$ = 7.6 Hz, 2H), 7.92–7.78 (m, 2H), 7.60–7.51 (m, 1H), 7.49–7.38 (m, 4H), 7.32 (d, $J$ = 5.5 Hz, 1H), 6.19–6.03 (m, 1H), 2.22–2.06 (m, 1H), 2.02–1.87 (m, 1H), 1.55–1.31 (m, 2H), 1.06–0.85 (m, 3H).

**$^{13}$C NMR** (101 MHz; $CDCl_3$) δ 166.08, 139.78, 139.39, 137.34, 133.06, 130.69, 129.79, 128.50, 127.10, 124.05, 123.00, 122.73, 121.81, 76.85, 38.97, 19.08, 14.02.

**IR** (Diamond-ATR, neat) $\bar{\nu}$ (cm$^{-1}$): 3069, 2958, 2933, 2871, 2360, 2341, 1716, 1450, 1314, 1272

**Specific Rotation** $[\alpha]^{27}_D$: 49.60 ($c$ = 1.0, $CHCl_3$)

**HRMS** (APGC) m/z: $[M]^+$ calcd for $C_{19}H_{18}O_2S^+$: 310.1022. Found: 310.1027

**Chiral HPLC (**Chiralcel AD-3, hexane/IPA = 90/10 over 25 min, 1.0 mL/min, 214 nm, 25 °C), 7.64 (minor), 9.97 (major)

(*S*)-1-(6-bromonaphthalen-2-yl)ethyl benzoate (**3n**)

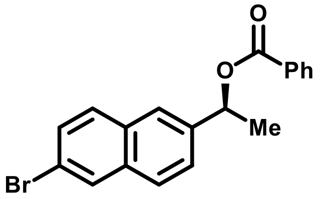


Reaction carried out at room temperature (25 °C). The residue was purified by reverse-phase column chromatography on C18 stationary phase (100 g cartridge, 20% to 100% MeCN in $H_2O$) to yield the titled compound (90 mg, 51%, 90:10 er) as a colorless oil.

**$^1$H NMR** (400 MHz; $CDCl_3$) δ 8.10 (d, *J* = 7.6 Hz, 2H), 8.00 (s, 1H), 7.86 (s, 1H), 7.80–7.67 (m, 2H), 7.63–7.52 (m, 3H), 7.50–7.41 (m, 2H), 6.28 (q, *J* = 6.6 Hz, 1H), 1.75 (d, *J* = 6.6 Hz, 3H).

**$^{13}$C NMR** (101 MHz; $CDCl_3$) δ 165.95, 139.86, 134.22, 133.18, 131.78, 130.53, 129.87, 129.86, 129.82, 129.78, 128.54, 127.71, 125.29, 125.07, 120.18, 72.97, 22.43.

**IR** (Diamond-ATR, neat) $\bar{\nu}$ ($cm^{-1}$): 3060, 2929, 1717, 1601, 1451, 1268, 1111, 1062, 879, 710

**Specific Rotation** $[\alpha]^{27}_D$: 26.10 (*c* = 1.0, $CHCl_3$)

**HRMS** (APGC) m/z: $[M]^+$ calcd for $C_{19}H_{15}BrO_2^+$: 354.0250. Found: 354.0254

**Chiral HPLC (**Chiralcel AD-3, hexane/IPA = 90/10 over 10 min, 1.0 mL/min, 214 nm, 25 °C), 3.82 (minor), 4.76 (major)

(*S*)-cyclobutyl(1-tosyl-1*H*-indol-5-yl)methyladamantane-1-carboxylate (**3o**)

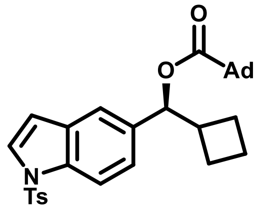


The residue was purified by reverse-phase column chromatography on C18 stationary phase (100 g cartridge, 20% to 100% MeCN in $H_2O$) to yield the titled compound (202 mg, 78%, 93:7 er) as a white solid.

**$^{1}$H NMR** (400 MHz; $CDCl_3$) δ 7.90 (d, *J* = 9.6 Hz, 1H), 7.77 (d, *J* = 8.2 Hz, 2H), 7.56–7.52 (m, 1H), 7.42 (s, 1H), 7.24–7.18 (m, 3H), 6.62 (s, 1H), 5.71 (d, *J* = 8.0 Hz, 1H), 2.79–2.68 (m, 1H), 2.35 (s, 3H), 2.03–1.94 (m, 5H), 1.90 (s, 6H), 1.85–1.75 (m, 4H), 1.76–1.66 (m, 6H).

**$^{13}$C NMR** (101 MHz; $CDCl_3$) δ 177.17, 145.09, 135.49, 135.31, 134.33, 130.73, 130.06, 127.01, 126.65, 123.20, 119.34, 113.40, 109.10, 78.32, 41.05, 40.98, 39.00, 36.65, 28.09, 24.76, 24.56, 21.72, 17.92.

**IR** (Diamond-ATR, neat) $\bar{\nu}$ ($cm^{-1}$): 2932, 2905, 2852, 2360, 2341, 1724, 1456, 1374, 1231, 1174

**Specific Rotation** $[\alpha]^{27}_{D}$: −24.30 (*c* = 1.0, $CHCl_3$)

**HRMS** (DART) m/z: $[M+Na]^+$ calcd for $C_{31}H_{35}NO_4SNa^+$: 540.2179. Found: 540.2174

**Chiral HPLC (**Chiralcel AD-3, hexane/IPA = 95/5 over 60 min, 1.0 mL/min, 214 nm, 25 °C), 36.92 (minor), 39.36 (major)

(*S*)-1-(naphthalen-2-yl)-3-(1*H*-pyrrol-1-yl)propyl benzoate (**3p**)

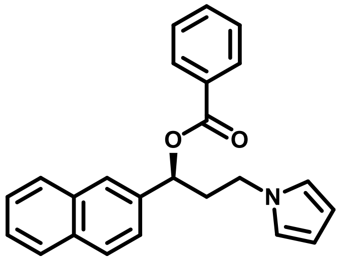

The residue was purified by reverse-phase column chromatography on C18 stationary phase (100 g cartridge, 20% to 100% MeCN in $H_2O$) to yield the titled compound (89 mg, 50%, 95:5 er) as a white solid.

**$^{1}$H NMR** (400 MHz; $CDCl_3$) δ 8.18–8.04 (m, 2H), 7.91–7.78 (m, 4H), 7.65–7.56 (m, 1H), 7.56–7.43 (m, 5H), 6.69–6.64 (m, 2H), 6.20–6.14 (m, 2H), 6.11 (dd, *J* = 8.3, 5.4 Hz, 1H), 4.11–3.91 (m, 2H), 2.76–2.60 (m, 1H), 2.54–2.40 (m, 1H).

**$^{13}$C NMR** (101 MHz; $CDCl_3$) δ 165.82, 137.17, 133.35, 133.28, 130.20, 129.86, 129.85, 128.85, 128.62, 128.22, 127.85, 126.54, 126.45, 125.91, 124.04, 120.62, 108.61, 74.30, 46.05, 38.30.

**IR** (Diamond-ATR, neat) $\bar{\nu}$ ($cm^{-1}$): 3056, 2960, 2903, 2849, 1718, 1492, 1269, 1109, 1026, 817

**Specific Rotation** $[\alpha]^{27}_{D}$: −5.50 (*c* = 1.0, $CHCl_3$)

**HRMS** (ESI) m/z: $[M+H]^+$ calcd for $C_{24}H_{22}NO_2^+$: 356.1645. Found: 356.1635

**Chiral HPLC (**Chiralcel AD-3, hexane/IPA = 90/10 over 30 min, 1.0 mL/min, 220 nm, 25 °C), 21.40 (minor), 26.63 (major)

(*S*,*E*)-4-phenylbut-3-en-2-yl benzoate (**3q**)

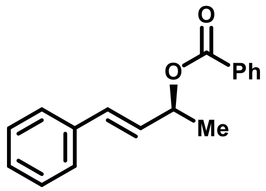

The residue was purified by reverse-phase column chromatography on C18 stationary phase (100 g cartridge, 20% to 100% MeCN in $H_2O$) to yield the titled compound (78 mg, 62%, 99:1 er) as a colorless oil.

**$^{1}$H NMR** (400 MHz; $CDCl_3$) δ 8.19 (d, *J* = 8.4 Hz, 2H), 7.60–7.52 (m, 1H), 7.50–7.42 (m, 4H), 7.39–7.32 (m, 2H), 7.31–7.21 (m, 1H), 6.76 (d, *J* = 16.0 Hz, 1H), 6.38 (dd, *J* = 16.0, 6.6 Hz, 1H), 5.93–5.83 (m, 1H), 1.61 (d, *J* = 6.5 Hz, 3H).

**$^{13}$C NMR** (101 MHz; $CDCl_3$) δ 165.56, 136.22, 132.73, 131.54, 130.55, 129.48, 128.71, 128.45, 128.21, 127.80, 126.51, 71.42, 20.34.

**IR** (Diamond-ATR, neat) $\bar{\nu}$ ($cm^{-1}$): 3061, 3030, 2978, 2931, 2360, 2341, 1716, 1494, 1450, 1269

**Specific Rotation** $[\alpha]^{27}_{D}$: 10.90 (*c* = 1.0, $CHCl_3$)

**HRMS** (APGC) m/z: $[M]^+$ calcd for $C_{17}H_{16}O_2^+$: 252.1145. Found: 252.1144

**Chiral HPLC (**Chiralcel AD-3, hexane/IPA = 95/5 over 10 min, 1.0 mL/min, 214 nm, 25 °C), 2.59 (minor), 3.04 (major)

($S$,$E$)-1-phenyloct-1-en-3-yladamantane-1-carboxylate (**3r**)

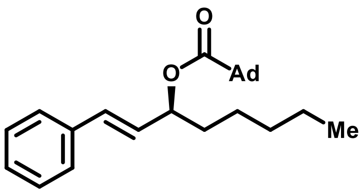


The residue was purified by reverse-phase column chromatography on C18 stationary phase (100 g cartridge, 20% to 100% MeCN in $H_2O$) to yield the titled compound (158 mg, 86%, 97:3 er) as a colorless oil.

**$^{1}$H NMR** (400 MHz; $CDCl_3$) δ 7.43–7.21 (m, 5H), 6.59 (d, $J$ = 16.0 Hz, 1H), 6.15 (dd, $J$ = 16.0, 6.9 Hz, 1H), 5.47–5.34 (m, 1H), 2.11–2.00 (m, 3H), 1.99–1.88 (m, 6H), 1.82–1.65 (m, 7H), 1.45–1.24 (m, 7H), 1.02–0.84 (m, 3H).

**$^{13}$C NMR** (101 MHz; $CDCl_3$) δ 177.07, 136.75, 131.76, 128.66, 128.48, 127.86, 126.68, 73.93, 40.99, 39.01, 36.69, 34.74, 31.71, 28.14, 24.95, 22.67, 14.16.

**IR** (Diamond-ATR, neat) $\bar{\nu}$ (cm$^{-1}$): 3082, 3060, 3026, 2929, 2906, 2852, 1725, 1452, 1232, 1073

**Specific Rotation** $[\alpha]^{27}_{D}$: −6.10 ($c$ = 1.0, $CHCl_3$)

**HRMS** (ESI) m/z: $[M+Na]^+$ calcd for $C_{25}H_{34}O_2Na^+$: 389.2451. Found: 389.2468.

**Chiral HPLC (**Chiralcel AD-3, hexane/IPA = 95/5 over 15 min, 1.0 mL/min, 214 nm, 25 °C), 6.94 (minor), 8.47 (major)

($S$,$E$)-5-(1,3-dioxan-2-yl)-1-phenylpent-1-en-3-yladamantane-1-carboxylate (**3s**)

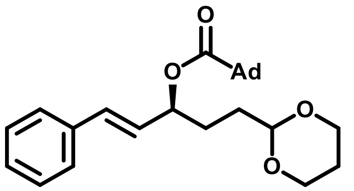


The residue was purified by reverse-phase column chromatography on C18 stationary phase (100 g cartridge, 20% to 100% MeCN in $H_2O$) to yield the titled compound (168 mg, 82%, >99:1 er) as a colorless oil.

**$^1$H NMR** (400 MHz; $CDCl_3$) δ 7.35 (d, $J$ = 7.6 Hz, 2H), 7.32–7.27 (m, 2H), 7.23 (t, $J$ = 7.6 Hz, 1H), 6.56 (d, $J$ = 15.9 Hz, 1H), 6.11 (dd, $J$ = 15.9, 6.8 Hz, 1H), 5.42–5.36 (m, 1H), 4.58–4.51 (m, 1H), 4.14–4.05 (m, 2H), 3.79–3.72 (m, 2H), 2.12–2.03 (m, 1H), 2.04–1.97 (m, 3H), 1.94–1.87 (m, 6H), 1.85–1.78 (m, 2H), 1.76–1.61 (m, 8H), 1.37–1.27 (m, 1H).

**$^{13}$C NMR** (101 MHz; $CDCl_3$) δ 176.99, 136.64, 132.04, 128.63, 128.02, 127.90, 126.71, 101.89, 73.49, 67.05, 41.00, 38.99, 36.67, 30.96, 29.08, 28.11, 25.92.

**IR** (Diamond-ATR, neat) $\bar{\nu}$ (cm$^{-1}$): 2906, 2850, 2360, 2341, 1724, 1451, 1232, 1144, 1074, 748

**Specific Rotation** $[\alpha]^{27}_{D}$: −11.00 ($c$ = 1.0, $CHCl_3$)

**HRMS** (ESI) m/z: $[M+Na]^+$ calcd for $C_{26}H_{34}O_4Na^+$: 433.2349. Found: 433.2329.

**Chiral HPLC (**Chiralcel OD-H, hexane/IPA = 95/5 over 25 min, 1.0 mL/min, 214 nm, 25 °C), 7.43 (major), 8.86 (minor)

(*S*)-1-(naphthalen-2-yl)ethyl acetate (**3t**)

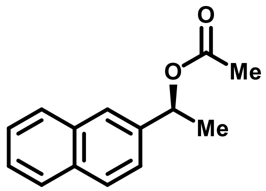

Reaction carried out at room temperature (25 °C). The residue was purified by reverse-phase column chromatography on C18 stationary phase (100 g cartridge, 20% to 100% MeCN in $H_2O$) to yield the titled compound (68 mg, 64%, 95:5 er) as a colorless oil.

**$^1$H NMR** (400 MHz; $CDCl_3$) δ 7.95–7.76 (m, 4H), 7.52–7.39 (m, 3H), 6.07 (q, $J$ = 6.6 Hz, 1H), 2.12 (s, 3H), 1.64 (d, $J$ = 6.6 Hz, 3H).

**$^{13}$C NMR** (101 MHz; $CDCl_3$) δ 170.48, 139.14, 133.31, 133.15, 128.48, 128.15, 127.79, 126.35, 126.18, 125.15, 124.23, 72.56, 22.31, 21.51.

**IR** (Diamond-ATR, neat) $\bar{\nu}$ ($cm^{-1}$): 3057, 2981, 1736, 1509, 1450, 1369, 1237, 1064, 1019, 818

**Specific Rotation** $[\alpha]^{27}_D$: −147.30 ($c$ = 1.0, $CHCl_3$)

**HRMS** (APGC) m/z: $[M]^+$ calcd for $C_{14}H_{14}O_2^+$: 214.0988. Found: 214.0983

**Chiral HPLC (**Chiralcel AD-3, hexane/IPA = 90/10 over 9 min, 1.0 mL/min, 214 nm, 25 °C), 4.50 (minor), 5.34 (major).

(*S*)-1-(naphthalen-2-yl)ethyl 2-methylbenzo[*d*]oxazole-5-carboxylate (**3u**)

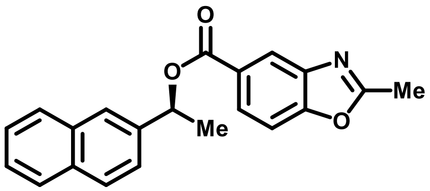


The residue was purified by reverse-phase column chromatography on C18 stationary phase (100 g cartridge, 20% to 100% MeCN in $H_2O$) to yield the titled compound (70 mg, 42%, 95:5 er) as a colorless oil.

**$^1$H NMR** (400 MHz; $CDCl_3$) δ 8.44 (s, 1H), 8.11 (d, $J$ = 8.6 Hz, 1H), 7.91 (s, 1H), 7.89–7.79 (m, 3H), 7.60 (d, $J$ = 8.6 Hz, 1H), 7.55–7.41 (m, 3H), 6.32 (q, $J$ = 6.6 Hz, 1H), 2.66 (s, 3H), 1.78 (d, $J$ = 6.6 Hz, 3H).

**$^{13}$C NMR** (101 MHz; $CDCl_3$) δ 165.67, 139.22, 133.35, 133.21, 128.62, 128.21, 128.14, 127.82, 127.15, 126.79, 126.41, 126.40, 126.24, 126.23, 125.16, 124.18, 121.70, 110.17, 73.49, 22.59, 14.74.

**IR** (Diamond-ATR, neat) $\bar{\nu}$ (cm$^{-1}$): 3056, 2979, 2903, 2848, 1714, 1626, 1434, 1285, 1276, 1077

**Specific Rotation** $[\alpha]^{27}_D$: 77.60 ($c$ = 1.0, $CHCl_3$)

**HRMS** (DART) m/z: $[M+H]^+$ calcd for $C_{21}H_{18}NO_3^+$: 332.1287. Found: 332.1401

**Chiral HPLC (**Chiralcel AD-3, hexane/IPA = 90/10 over 25 min, 1.0 mL/min, 214 nm, 25 °C) 18.34 (minor), 19.64 (major).

($S$)-1-(naphthalen-2-yl)ethyl (1$s$,3$s$)-adamantane-1-carboxylate (**3v**)

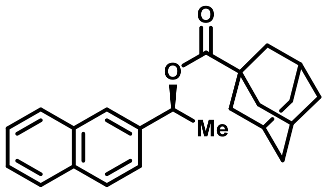

Reaction carried out at room temperature (25 °C). The residue was purified by reverse-phase column chromatography on C18 stationary phase (100 g cartridge, 20% to 100% MeCN in $H_2O$) to yield the titled compound (122 mg, 73%, 98:2 er) as a white solid.

**$^{1}$H NMR** (400 MHz; $CDCl_3$) δ 7.92–7.71 (m, 4H), 7.53–7.42 (m, 3H), 6.02 (q, $J$ = 6.6 Hz, 1H), 2.06–1.99 (m, 3H), 1.98–1.86 (m, 6H), 1.82–1.67 (m, 6H), 1.60 (d, $J$ = 6.6 Hz, 3H).

**$^{13}$C NMR** (101 MHz; $CDCl_3$) δ 176.99, 139.74, 133.35, 133.07, 128.42, 128.17, 127.80, 126.28, 126.07, 124.86, 124.14, 71.84, 40.86, 38.94, 36.68, 28.12, 22.46.

**IR** (Diamond-ATR, neat) $\bar{\nu}$ ($cm^{-1}$): 3056, 2905, 2850, 1725, 1452, 1231, 1183, 1103, 1074, 817

**Specific Rotation** $[\alpha]^{27}_{D}$: –29.60 ($c$ = 1.0, $CHCl_3$)

**HRMS** (ESI) m/z: $[M+Na]^+$ calcd for $C_{23}H_{26}O_2Na^+$: 357.1825. Found: 357.1828

**Chiral HPLC (**Chiralcel AD-3, hexane/IPA = 90/10 over 10 min, 1.0 mL/min, 214 nm, 25 °C), 4.20 (minor), 5.41 (major)

(*S*)-1-(naphthalen-2-yl)ethyl 4-bromobutanoate (**3w**)

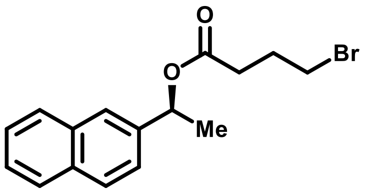


The residue was purified by reverse-phase column chromatography on C18 stationary phase (100 g cartridge, 20% to 100% MeCN in $H_2O$) to yield the titled compound (88 mg, 55%, 94:6 er) as a colorless oil.

**$^1$H NMR** (400 MHz; $CDCl_3$) δ 7.90–7.74 (m, 4H), 7.55–7.39 (m, 3H), 6.07 (q, $J$ = 6.6 Hz, 1H), 3.54–3.37 (m, 2H), 2.68–2.45 (m, 2H), 2.27–2.11 (m, 2H), 1.64 (d, $J$ = 6.6 Hz, 3H).

**$^{13}$C NMR** (101 MHz; $CDCl_3$) δ 171.95, 139.04, 133.31, 133.18, 128.57, 128.17, 127.81, 126.42, 126.25, 125.14, 124.12, 72.86, 32.93, 32.84, 27.89, 22.38.

**IR** (Diamond-ATR, neat) $\bar{\nu}$ ($cm^{-1}$): 3056, 2978, 1733, 1443, 1373, 1307, 1200, 1171, 1127, 1062

**Specific Rotation** $[\alpha]^{27}_D$: –45.90 ($c$ = 1.0, $CHCl_3$)

**HRMS** (APGC) m/z: $[M]^+$ calcd for $C_{16}H_{17}BrO_2^+$: 320.0406. Found: 320.0424

**Chiral HPLC (**Chiralcel AD-3, hexane/IPA = 90/10 over 10 min, 1.0 mL/min, 214 nm, 25 °C), 5.52 (minor), 6.95 (major)

(*S*)-1-(naphthalen-2-yl)ethyl 4,4-difluorocyclohexane-1-carboxylate (**3x**)

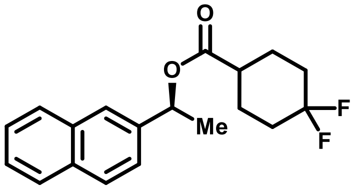


The residue was purified by reverse-phase column chromatography on C18 stationary phase (100 g cartridge, 20% to 100% MeCN in $H_2O$) to yield the titled compound (96 mg, 61%, 95:5 er) as a colorless oil.

**$^1$H NMR (**400 MHz; $CDCl_3$) δ 7.87–7.75 (m, 4H), 7.53–7.42 (m, 3H), 6.07 (q, *J* = 6.6 Hz, 1H), 2.51–2.39 (m, 1H), 2.19–1.95 (m, 4H), 1.95–1.69 (m, 4H), 1.63 (d, *J* = 6.6 Hz, 3H).

**$^{13}$C NMR** (101 MHz; $CDCl_3$) δ 173.57, 138.97, 133.31, 133.19, 128.61, 128.16, 127.83, 126.45, 126.30, 125.16, 124.04, 121.63 (t, *J* = 241.1 Hz), 72.78, 40.85, 32.67 (t, *J* = 24.5 Hz), 25.23, 25.15, 22.29.

**$^{19}$F NMR** (376 MHz; $CDCl_3$) δ –94.29 (d, *J* = 237.9 Hz), –99.55 (d, *J* = 237.9 Hz).

**IR** (Diamond-ATR, neat) $\bar{\nu}$ ($cm^{-1}$): 3057, 2962, 1730, 1450, 1370, 1258, 1192, 1174, 1107, 965

**Specific Rotation** $[\alpha]^{27}_D$: −52.50 (*c* = 1.0, $CHCl_3$)

**HRMS** (ESI) m/z: $[M+H]^+$ calcd for $C_{19}H_{20}F_2O_2^+$: 318.1426. Found: 318.1459

**Chiral HPLC (**Chiralcel AD-3, hexane/IPA = 90/10 over 10 min, 1.0 mL/min, 214 nm, 25 °C), 5.24 (minor), 6.49 (major)

(*S*)-1-(naphthalen-2-yl)ethyl benzo[*b*]thiophene-3-carboxylate (**3y**)

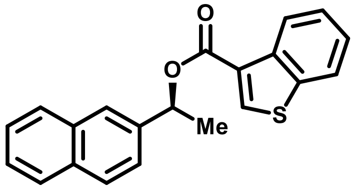


The residue was purified by reverse-phase column chromatography on C18 stationary phase (100 g cartridge, 20% to 100% MeCN in $H_2O$) to yield the titled compound (106 mg, 64%, 94:6 er) as a colorless oil.

**$^1$H NMR** (400 MHz; $CDCl_3$) δ 8.70 (d, *J* = 8.1 Hz, 1H), 8.50 (s, 1H), 8.02–7.83 (m, 5H), 7.65 (d, *J* = 8.1 Hz, 1H), 7.58–7.47 (m, 3H), 7.47–7.38 (m, 1H), 6.41 (q, *J* = 6.6 Hz, 1H), 1.86 (d, *J* = 6.6 Hz, 3H).

**$^{13}$C NMR** (101 MHz; $CDCl_3$) δ 162.19, 140.21, 139.20, 136.91, 136.88, 133.36, 133.21, 128.65, 128.21, 127.82, 127.56, 126.41, 126.24, 125.57, 125.21, 125.15, 124.90, 124.22, 122.64, 72.99, 22.50.

**IR** (Diamond-ATR, neat) $\bar{\nu}$ ($cm^{-1}$): 3056, 2978, 2927, 1710, 1504, 1462, 1217, 1063, 1014, 817

**Specific Rotation** $[\alpha]^{27}_D$: 32.80 (*c* = 0.8, $CHCl_3$)

**HRMS** (ESI) m/z: $[M+H]^+$ calcd for $C_{21}H_{17}O_2S^+$: 333.0944. Found: 333.0956

**Chiral HPLC (**Chiralcel AD-3, hexane/IPA = 95/5 over 10 min, 1.0 mL/min, 214 nm, 25 °C), 4.27 (minor), 7.31 (major)

(*S*)-1-(naphthalen-2-yl)ethyl tetrahydro-2*H*-pyran-4-carboxylate (**3z**)

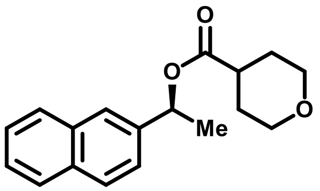

The residue was purified by reverse-phase column chromatography on C18 stationary phase (100 g cartridge, 20% to 100% MeCN in $H_2O$) to yield the titled compound (100 mg, 70%, 97:3 er) as a colorless oil.

**$^{1}$H NMR** (400 MHz; $CDCl_3$) δ 7.88–7.75 (m, 4H), 7.54–7.44 (m, 3H), 6.08 (q, $J$ = 6.6 Hz, 1H), 4.03–3.90 (m, 2H), 3.52–3.36 (m, 2H), 2.66–2.51 (m, 1H), 1.95–1.72 (m, 4H), 1.63 (d, $J$ = 6.6 Hz, 3H).

**$^{13}$C NMR** (101 MHz; $CDCl_3$) δ 173.83, 139.11, 133.30, 133.15, 128.55, 128.15, 127.81, 126.40, 126.23, 125.10, 124.07, 72.58, 67.22, 40.44, 28.77, 22.34.

**IR** (Diamond-ATR, neat) $\bar{\nu}$ ($cm^{-1}$): 3056, 2955, 2929, 2847, 1731, 1446, 1317, 1278, 1169, 1064

**Specific Rotation** $[\alpha]^{27}_{D}$: −62.20 ($c$ = 1.0. $CHCl_3$)

**HRMS** (APGC) m/z: $[M]^+$ calcd for $C_{18}H_{20}O_3^+$: 284.1407. Found: 284.1423

**Chiral HPLC (**Chiralcel AD-3, hexane/IPA = 90/10 over 10 min, 1.0 mL/min, 214 nm, 25 °C), 7.00 (minor), 7.95 (major)

(*S*)-1-(naphthalen-2-yl)ethyl 2, 1-(naphthalen-2-yl)ethyl cyclobutanecarboxylate (**3aa**)

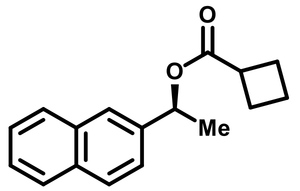

The residue was purified by reverse-phase column chromatography on C18 stationary phase (100 g cartridge, 20% to 100% MeCN in $H_2O$) to yield the titled compound (91 mg, 72%, 97:3 er) as a colorless oil.

**$^1$H NMR** (400 MHz; $CDCl_3$) δ 7.89–7.76 (m, 4H), 7.54–7.42 (m, 3H), 6.06 (q, $J$ = 6.6 Hz, 1H), 3.24–3.13 (m, 1H), 2.39–2.14 (m, 4H), 2.08–1.81 (m, 2H), 1.62 (d, $J$ = 6.6 Hz, 3H).

**$^{13}$C NMR** (101 MHz; $CDCl_3$) δ 174.86, 139.43, 133.34, 133.12, 128.46, 128.16, 127.79, 126.32, 126.13, 125.03, 124.20, 72.19, 38.46, 25.33, 25.29, 22.37, 18.53.

**IR** (Diamond-ATR, neat) $\bar{\nu}$ ($cm^{-1}$): 3056, 2979, 2948, 2867, 1728, 1361, 1250, 1169, 1064, 818

**Specific Rotation** $[\alpha]^{27}_{D}$: −57.70 ($c$ = 1.0, $CHCl_3$)

**HRMS** (APGC) m/z: $[M]^+$ calcd for $C_{17}H_{18}O_2^+$: 254.1301. Found: 254.1295

**Chiral HPLC (**Chiralcel AD-3, hexane/IPA = 95/5 over 10 min, 1.0 mL/min, 214 nm, 25 °C), 5.03 (minor), 6.90 (major)

(*S*)-1-(naphthalen-2-yl)ethyl 3-methyloxetane-3-carboxylate (**3ab**)

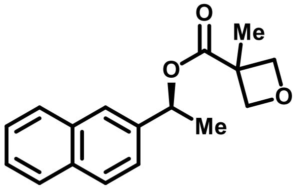


The residue was purified by reverse-phase column chromatography on C18 stationary phase (100 g cartridge, 20% to 100% MeCN in $H_2O$) to yield the titled compound (95 mg, 70%, 94:6 er) as a colorless oil.

**$^1$H NMR** (400 MHz; $CDCl_3$) δ 7.88–7.75 (m, 4H), 7.53–7.43 (m, 3H), 6.11 (q, $J$ = 6.7 Hz, 1H), 5.02–4.92 (m, 2H), 4.49–4.37 (m, 2H), 1.66 (d, $J$ = 6.7 Hz, 3H), 1.62 (s, 3H).

**$^{13}$C NMR** (101 MHz; $CDCl_3$) δ 173.63, 138.60, 133.16, 133.09, 128.55, 128.05, 127.70, 126.36, 126.22, 125.11, 123.83, 79.58, 79.54, 73.19, 44.60, 22.12, 21.83.

**IR** (Diamond-ATR, neat) $\bar{\nu}$ (cm$^{-1}$): 3056, 2972, 2877, 2360, 2341, 1731, 1455, 1297, 1157, 1061

**Specific Rotation** $[\alpha]^{27}_{D}$: −80.00 ($c$ = 0.5, $CHCl_3$)

**HRMS** (APGC) m/z: $[M]^+$ calcd for $C_{17}H_{18}O_3^+$: 270.1250. Found: 270.1293

**Chiral HPLC (**Chiralcel AD-3, hexane/IPA = 90/10 over 10 min, 1.0 mL/min, 214 nm, 25 °C), 6.43 (minor), 7.34 (major)

(*S*)-1-(naphthalen-2-yl)ethyl 4-(*N*,*N*-dipropylsulfamoyl)benzoate (**3ac**)

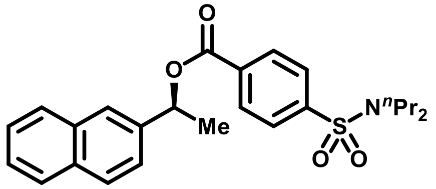


Reaction carried out in DCE (2.5 mL). The residue was purified by reverse-phase column chromatography on C18 stationary phase (100 g cartridge, 20% to 100% MeCN in $H_2O$) to yield the titled compound (152 mg, 69%, 93:7 er) as a yellow oil.

**$^1$H NMR** (400 MHz; $CDCl_3$) δ 8.21 (d, *J* = 8.3 Hz, 2H), 7.98–7.79 (m, 6H), 7.58 (d, *J* = 8.6 Hz, 1H), 7.52–7.44 (m, 2H), 6.32 (q, *J* = 6.7 Hz, 1H), 3.17–2.98 (m, 4H), 1.79 (d, *J* = 6.7 Hz, 3H), 1.62–1.47 (m, 4H), 0.87 (t, *J* = 7.4 Hz, 6H).

**$^{13}$C NMR** (101 MHz; $CDCl_3$) δ 164.66, 144.37, 138.61, 133.92, 133.29, 133.27, 130.41, 128.71, 128.18, 127.83, 127.13, 126.51, 126.39, 125.39, 124.10, 74.09, 50.07, 22.29, 22.06, 11.28.

**IR** (Diamond-ATR, neat) $\bar{\nu}$ ($cm^{-1}$): 2965, 2931, 2875, 1719, 1341, 1268, 1158, 1087, 993, 747

**Specific Rotation** $[\alpha]^{27}_D$: 58.50 (*c* = 1.0, $CHCl_3$)

**HRMS** (DART) m/z: $[M+H]^+$ calcd for $C_{25}H_{30}NO_4S^+$: 440.1890. Found: 440.1888

**Chiral HPLC (**Chiralcel AD-3, hexane/IPA = 90/10 over 25 min, 1.0 mL/min, 214 nm, 25 °C), 17.95 (major), 18.80 (minor)

(*S*)-1-(2-naphthyl)ethyl 2-(3-cyano-4-isobutoxyphenyl)-4-methylthiazole-5-carboxylate (**3ad**)

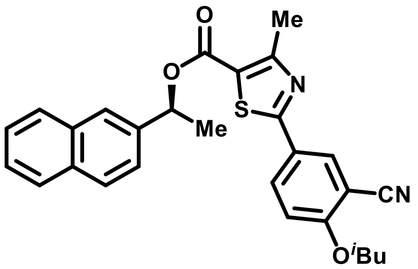


Reaction carried out in DCE (2.5 mL). The residue was purified by reverse-phase column chromatography on C18 stationary phase (100 g cartridge, 20% to 100% MeCN in $H_2O$) to yield the titled compound (153 mg, 65%, 93:7 er) as a colorless oil.

**$^1$H NMR** (400 MHz; $CDCl_3$) δ 8.19 (s, 1H), 8.11 (d, *J* = 11.3 Hz, 1H), 7.91–7.78 (m, 4H), 7.58–7.43 (m, 3H), 7.01 (d, *J* = 8.9 Hz, 1H), 6.25 (q, *J* = 6.6 Hz, 1H), 3.90 (d, *J* = 6.5 Hz, 2H), 2.77 (s, 3H), 2.29–2.11 (m, 1H), 1.76 (d, *J* = 6.6 Hz, 3H), 1.09 (d, *J* = 6.8 Hz, 6H).

**$^{13}$C NMR** (101 MHz; $CDCl_3$) δ 167.42, 162.65, 161.49, 161.44, 138.70, 133.31, 133.26, 132.69, 132.26, 128.73, 128.22, 127.84, 126.49, 126.36, 126.13, 125.29, 124.03, 122.09, 115.52, 112.74, 103.13, 75.84, 73.90, 28.30, 22.53, 19.19, 17.69.

**IR** (Diamond-ATR, neat) $\bar{\nu}$ ($cm^{-1}$): 3057, 2963, 2936, 2874, 2228, 1710, 1259,1091, 1060, 819

**Specific Rotation** $[\alpha]^{27}_D$: 292.10 (*c* = 1.0, $CHCl_3$)

**HRMS** (DART) m/z: $[M+H]^+$ calcd for $C_{28}H_{27}N_2O_3S^+$: 471.1737. Found: 471.1742.

**Chiral HPLC (**Chiralcel AD-3, hexane/IPA = 90/10 over 30 min, 2.0 mL/min, 214 nm, 25 °C), 12.23 (minor), 18.93 (major)

(*S*)-1-(naphthalen-2-yl)ethyl 2-(4-(2-(4-chlorobenzamido)ethyl)phenoxy)-2-methylpropanoate (**3ae**)

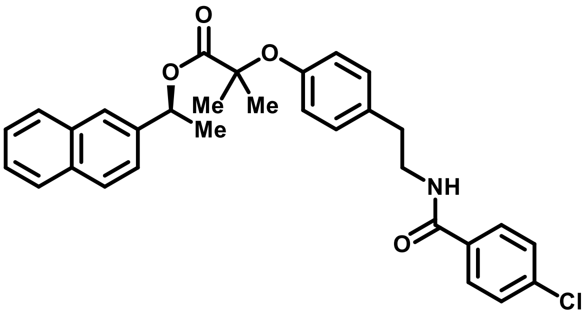


The residue was purified by reverse-phase column chromatography on C18 stationary phase (100 g cartridge, 20% to 100% MeCN in $H_2O$) to yield the titled compound (157 mg, 61%, 86:14 er) as a white solid.

The compound (100 mg) was recrystallized by slow-cooling of a hot, saturated hexane solution (*ca.* 3 mL) to −20 °C over 16 h, yielding further enantiomerically enriched material (65 mg, 65 % recovery yield, 96:4 er).

**$^1$H NMR** (400 MHz; $CDCl_3$) δ 7.84–7.65 (m, 4H), 7.53 (d, *J* = 8.4 Hz, 2H), 7.50–7.42 (m, 2H), 7.42–7.31 (m, 3H), 6.91 (d, *J* = 8.4 Hz, 2H), 6.71 (d, *J* = 8.4 Hz, 2H), 6.12 (q, *J* = 6.6 Hz, 1H), 5.87 (s, 1H), 3.68–3.45 (m, 2H), 2.85–2.69 (m, 2H), 1.66–1.55 (m, 9H).

**$^{13}$C NMR** (101 MHz; $CDCl_3$) δ 173.57, 166.40, 154.30, 138.37, 137.71, 133.18, 133.12, 133.10, 132.18, 129.50, 128.91, 128.41, 128.32, 128.14, 127.76, 126.39, 126.33, 125.34, 124.16, 119.23, 79.23, 73.60, 41.24, 34.75, 25.69, 25.34, 22.06.

**IR** (Diamond-ATR, neat) $\bar{\nu}$ ($cm^{-1}$): 3327, 3059, 2981, 2934, 1731, 1638, 1508, 1486, 1278, 1175

**Specific Rotation** $[\alpha]^{27}_D$: −17.70 (*c* = 1.0, $CHCl_3$)

**HRMS** (DART) m/z: $[M+Na]^+$ calcd for $C_{31}H_{30}ClNO_4Na^+$: 538.1756. Found: 538.1782

**Chiral HPLC (**Chiralcel AD-3, hexane/IPA = 80/20 over 15 min, 1.0 mL/min, 214 nm, 25 °C), 4.63 (minor), 9.73 (major)

(*S*)-1-(naphthalen-2-yl)ethyl 3-(4,5-diphenyloxazol-2-yl)propanoate (**3af**)

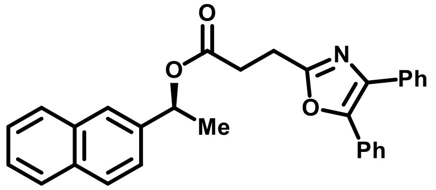


Reaction carried out in DCE (2.5 mL). The residue was purified by reverse-phase column chromatography on C18 stationary phase (100 g cartridge, 20% to 100% MeCN in $H_2O$) to yield the titled compound (110 mg, 49%, 90:10 er) as a colorless oil.

**$^1$H NMR** (400 MHz; $CDCl_3$) δ 7.82–7.76 (m, 4H), 7.64–7.58 (m, 2H), 7.57–7.51 (m, 2H), 7.49–7.44 (m, 3H), 7.38–7.28 (m, 6H), 6.12 (q, *J* = 6.6 Hz, 1H), 3.27–3.16 (m, 2H), 3.04–2.89 (m, 2H), 1.64 (d, *J* = 6.6 Hz, 3H).

**$^{13}$C NMR** (101 MHz; $CDCl_3$) δ 171.37, 161.87, 145.52, 138.95, 135.24, 133.28, 133.15, 132.58, 129.09, 128.73, 128.65, 128.53, 128.18, 128.14, 128.02, 128.01, 127.78, 126.59, 126.34, 126.18, 125.13, 124.12, 73.05, 31.59, 23.68, 22.37.

**IR** (Diamond-ATR, neat) $\bar{\nu}$ ($cm^{-1}$): 3056, 2975, 2929, 1733, 1444, 1363, 1165, 1059, 762, 693

**Specific Rotation** $[\alpha]^{27}_D$: −102.50 (*c* = 1.0, $CHCl_3$)

**HRMS** (DART) m/z: $[M+Na]^+$ calcd for $C_{30}H_{25}NO_3Na^+$: 470.1727. Found: 470.1735.

**Chiral HPLC (**Chiralcel AD-3, hexane/IPA = 90/10 over 7 min, 1.0 mL/min, 214 nm, 25 °C), 4.19 (minor), 5.31 (major)

(*S*)-1-(naphthalen-2-yl)ethyl 4-(4-(bis(2-chloroethyl)amino)phenyl)butanoate (**3ag**)

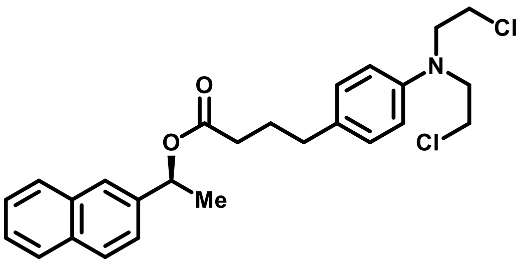


The residue was purified by reverse-phase column chromatography on C18 stationary phase (100 g cartridge, 20% to 100% MeCN in $H_2O$) to yield the titled compound (126 mg, 55%, 94:6 er) as a colorless oil.

*Note*: The preparation of **3ag** was also carried out with the carboxylic acid **2ag** as the limiting reagent (152 mg, 0.50 mmol, 1.0 equiv) and superstoichimetric alkylborane **1a** (282 mg, 1.00 mmol, 2.0 equiv). The reaction resulted in product **3ag** with similar yield and enantioenrichment (108 mg, 47%, 94:6 er).

**$^1$H NMR** (400 MHz; $CDCl_3$) δ 7.89–7.80 (m, 4H), 7.54–7.45 (m, 3H), 7.04 (d, *J* = 8.8 Hz, 2H), 6.61 (d, *J* = 8.8 Hz, 2H), 6.09 (q, *J* = 6.6 Hz, 1H), 3.73–3.66 (m, 4H), 3.64–3.58 (m, 4H), 2.60–2.49 (m, 2H), 2.43–2.34 (m, 2H), 1.98–1.88 (m, 2H), 1.64 (d, *J* = 6.6 Hz, 3H).

**$^{13}$C NMR** (101 MHz; $CDCl_3$) δ 172.93, 144.40, 139.22, 133.31, 133.14, 130.74, 129.82, 128.48, 128.15, 127.80, 126.36, 126.19, 125.16, 124.26, 112.27, 72.38, 53.72, 40.63, 34.06, 34.04, 26.91, 22.33.

**IR** (Diamond-ATR, neat) $\bar{\nu}$ (cm$^{-1}$): 3056, 2961, 2904, 2849, 1731, 1615, 1518, 1360, 1248, 1178

**Specific Rotation** $[\alpha]^{27}_{D}$: −108.10 (*c* = 1.0, $CHCl_3$)

**HRMS** (DART) m/z: $[M+H]^+$ calcd for $C_{26}H_{30}Cl_2NO_2^+$: 458.1648. Found: 458.1648

**Chiral HPLC (**Chiralcel AD-3, hexane/IPA = 90/10 over 25 min, 1.0 mL/min, 214 nm, 25 °C), 10.03 (minor), 16.55 (major)

(*S*)-1-(naphthalen-2-yl)ethyl 2-(1-methyl-5-(4-methylbenzoyl)-1*H*-pyrrol-2-yl)acetate (**3ah**)

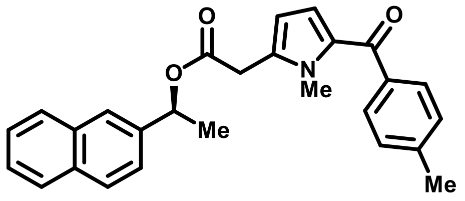


The residue was purified by reverse-phase column chromatography on C18 stationary phase (100 g cartridge, 20% to 100% MeCN in $H_2O$) to yield the titled compound (107 mg, 52%, 85:15 er) as a yellow oil.

**$^1$H NMR** (400 MHz; $CDCl_3$) δ 7.86–7.78 (m, 3H), 7.76–7.67 (m, 3H), 7.52–7.45 (m, 2H), 7.43 (d, $J$ = 9.9 Hz, 1H), 7.25 (d, $J$ = 8.5 Hz, 2H), 6.68 (d, $J$ = 4.2 Hz, 1H), 6.16–6.02 (m, 2H), 3.91 (s, 3H), 3.76 (s, 2H), 2.43 (s, 3H), 1.66 (d, $J$ = 6.7 Hz, 3H).

**$^{13}$C NMR** (101 MHz; $CDCl_3$) δ 186.01, 168.77, 142.01, 138.56, 137.50, 134.63, 133.27, 133.21, 131.55, 129.58, 128.83, 128.61, 128.18, 127.82, 126.48, 126.35, 125.21, 123.98, 122.41, 109.63, 73.76, 33.42, 33.34, 22.28, 21.68.

**IR** (Diamond-ATR, neat) $\bar{\nu}$ ($cm^{-1}$): 3055, 2928, 1737, 1624, 1606, 1455, 1374, 1263, 1172, 749

**Specific Rotation** $[\alpha]^{27}_{D}$: −30.40 ($c$ = 1.0, $CHCl_3$)

**HRMS** (DART) m/z: $[M+H]^+$ calcd for $C_{27}H_{26}NO_3^+$: 412.1907. Found: 412.1929.

**Chiral HPLC (**Chiralcel AD-3, hexane/IPA = 95/5 over 45 min, 2.0 mL/min, 214 nm, 25 °C), 39.38 (major), 42.41 (minor)

(*S*)-1-(naphthalen-2-yl)ethyl 2-(1-(4-chlorobenzoyl)-2-methyl-1*H*-indol-3-yl)acetate (**3ai**)

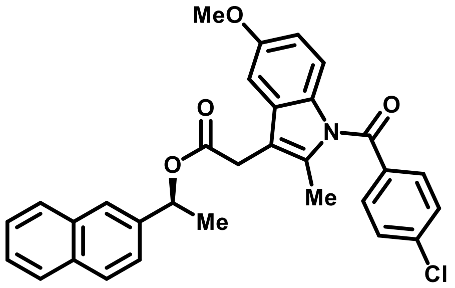


The residue was purified by reverse-phase column chromatography on C18 stationary phase (100 g cartridge, 20% to 100% MeCN in $H_2O$) to yield the titled compound (141 mg, 55%, 86:14 er) as a white solid.

The compound (100 mg) was recrystallized by slow-cooling of a hot, saturated hexane solution (*ca*. 3 mL) to −20 °C over 16 h, yielding further enantiomerically enriched material (71 mg, 71 % recovery yield, 98:2 er).

**$^1$H NMR** (400 MHz; $CDCl_3$) δ 7.84–7.75 (m, 2H), 7.72–7.63 (m, 2H), 7.59 (d, *J* = 8.7 Hz, 2H), 7.50–7.43 (m, 2H), 7.43–7.33 (m, 3H), 6.97–6.88 (m, 2H), 6.68 (dd, *J* = 9.0, 2.6 Hz, 1H), 6.06 (q, *J* = 6.6 Hz, 1H), 3.79–3.65 (m, 5H), 2.35 (s, 3H), 1.62 (d, *J* = 6.6 Hz, 3H).

**$^{13}$C NMR** (101 MHz; $CDCl_3$) δ 170.20, 168.42, 156.19, 139.32, 138.90, 135.92, 134.04, 133.23, 133.09, 131.25, 130.95, 130.75, 129.20, 128.41, 128.12, 127.75, 126.38, 126.25, 125.01, 124.07, 115.10, 112.81, 112.01, 101.35, 73.27, 55.68, 30.91, 22.40, 13.54.

**IR** (Diamond-ATR, neat) $\bar{\nu}$ ($cm^{-1}$): 2962, 2928, 2902, 2848, 1733, 1681, 1591, 1477, 1314, 1164

**Specific Rotation** $[\alpha]^{27}_D$: −44.51 (*c* = 0.9, $CHCl_3$)

**HRMS** (ESI) m/z: $[M+H]^+$ calcd for $C_{31}H_{27}ClNO_4^+$: 512.1623. Found: 512.1630.

**Chiral HPLC (**Chiralcel AD-3, hexane/IPA = 90/10 over 30 min, 2.0 mL/min, 214 nm, 25 °C), 15.20 (minor), 24.55 (major)

($S$)-1-(naphthalen-2-yl)ethyl 5-(2,5-dimethylphenoxy)-2,2-dimethylpentanoate (**3aj**)

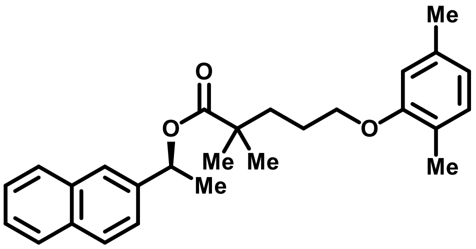


The residue was purified by reverse-phase column chromatography on C18 stationary phase (100 g cartridge, 20% to 100% MeCN in $H_2O$) to yield the titled compound (192 mg, 95%, 95:5 er) as a white solid.

*Gram-Scale Procedure*: Inside a nitrogen-filled glovebox, a 50-mL round-bottom flask equipped with a magnetic stir bar was charged CuCl (50.0 mg, 0.500 mmol, 10 mol%), **L6** (735 mg, 0.750 mmol, 15 mol%), and $NaBAr^F{}_4$ (665 mg, 0.750 mmol, 15 mol%) followed by the addition of a 1:1 mixture of PhCl and PhMe (25 mL). The reaction mixture was stirred at room temperature for 10 min before the addition of **4e** (140 mg, 0.500 mmol, 10 mol%), alkylborane **1a** (1.41 g, 5.00 mmol, 1.0 equiv), and carboxylic acid **2aj** (2.50 g, 10.0 mmol, 2.0 equiv). The reaction mixture was stirred for 10 min before di-*tert*-butyl peroxide (1.83 mL, 10.0 mmol, 2.0 equiv) was added in one portion. The reaction flask was sealed with a rubber septum, taken outside of the glovebox, placed in a –20 °C freezer and irradiated with 427 nm light (Kessil P160L) with stirring.

After the reaction mixture had stirred for 16 h, the septum was removed from the flask, and the reaction mixture was concentrated in vacuo with the aid of a rotary evaporator. The resulting crude material was purified by reverse-phase (C18) column chromatography to yield the titled compound **3aj** (1.60 g, 79%, 95:5 er) as a white solid.

**$^1$H NMR** (400 MHz; $CDCl_3$) δ 7.89–7.75 (m, 4H), 7.52–7.42 (m, 3H), 7.01 (d, $J$ = 7.5 Hz, 1H), 6.67 (d, $J$ = 7.5 Hz, 1H), 6.59 (s, 1H), 6.06 (q, $J$ = 6.6 Hz, 1H), 3.94–3.83 (m, 2H), 2.31 (s, 3H), 2.16 (s, 3H), 1.84–1.66 (m, 4H), 1.63 (d, $J$ = 6.6 Hz, 3H), 1.27 (s, 3H), 1.25 (s, 3H).
**$^{13}$C NMR** (101 MHz; $CDCl_3$) δ 177.07, 157.10, 139.45, 136.57, 133.33, 133.11, 130.41, 128.46, 128.15, 127.80, 126.33, 126.13, 125.00, 124.14, 123.72, 120.79, 112.05, 72.39, 68.08, 42.24, 37.31, 25.30, 25.27, 22.41, 21.54, 15.91.

**IR** (Diamond-ATR, neat) $\bar{\nu}$ ($cm^{-1}$): 3054, 2969, 2927, 2870, 1726, 1585, 1265, 1149, 1128, 1062

**Specific Rotation** $[\alpha]^{27}_{D}$: −29.90 (*c* = 1.0, $CHCl_3$)

**HRMS** (ESI) m/z: $[M+Na]^+$ calcd for $C_{27}H_{32}O_3Na^+$: 427.2244. Found: 427.2244

**Chiral HPLC (**Chiralcel AD-3, hexane/IPA = 90/10 over 10 min, 1.0 mL/min, 214 nm, 25 °C), 1.62 (minor), 2.12 (major)

(*S*)-1-(naphthalen-2-yl)ethyl 2-(4-(2,2-dichlorocyclopropyl)phenoxy)-2-methylpropanoate (**3ak**)

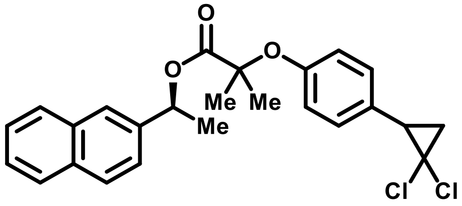

The residue was purified by reverse-phase column chromatography on C18 stationary phase (100 g cartridge, 20% to 100% MeCN in $H_2O$) to yield the titled compound (113 mg, 51%, 85:15 er) as a white solid.

*Note:* The product was isolated as a 1:1 mixture of the two diastereomers as indicated by $^1$H NMR spectroscopy (see below).

The compound (100 mg) was recrystallized by slow-cooling of a hot, saturated hexane solution (*ca.* 3 mL) to −20 °C over 16 h, yielding further enantiomerically enriched material (53 mg, 53 % recovery yield, 92:8 er). The recrystallization did not improve the diastereomeric enrichment. A 1:1 dr was observed by $^1$H NMR spectroscopy.

**$^1$H NMR** (400 MHz; $CDCl_3$) δ (1:1 mixture of two diastereomers) 7.86–7.68 (m, 4H), 7.53–7.45 (m, 2H), 7.41–7.34 (m, 1H), 6.96–6.87 (m, 2H), 6.77–6.66 (m, 2H), 6.18–6.06 (m, 1H), 2.79–2.69 (m, 1H), 1.93–1.84 (m, 1H), 1.73–1.65 (m, 1H), 1.64 (s, 3H), 1.62–1.58 (m, 6H).

**$^{13}$C NMR** (101 MHz; $CDCl_3$) δ (diastereomer 1) 173.57, 155.04, 138.33, 133.21, 133.19, 129.65, 128.46, 128.26, 127.97, 127.85, 127.78, 126.34, 125.50, 124.25, 118.45, 79.23, 73.76, 61.04, 34.91, 25.88, 25.75, 25.40, 21.98. (diastereomer 2) 173.57, 155.04, 138.31, 133.19, 133.17, 129.60, 128.44, 128.26, 127.97, 127.85, 127.76, 126.29, 125.48, 124.23, 118.33, 79.20, 73.72, 60.99, 34.87, 25.86, 25.67, 25.33, 21.96.

**IR** (Diamond-ATR, neat) $\bar{\nu}$ ($cm^{-1}$): 3056, 2964, 2903, 2848, 1731, 1610, 1511, 1279, 1243, 1146

**Specific Rotation** $[\alpha]^{27}_D$: −29.90 ($c$ = 1.0, $CHCl_3$)

**HRMS** (ESI) m/z: $[M+Na]^+$ calcd for $C_{25}H_{24}Cl_2O_3Na^+$: 465.0995. Found: 465.0993

**Chiral HPLC (**Chiralcel AD-3, hexane/IPA = 90/10 over 10 min, 1.0 mL/min, 214 nm, 25 °C), 5.18 (minor), 7.16 (major)

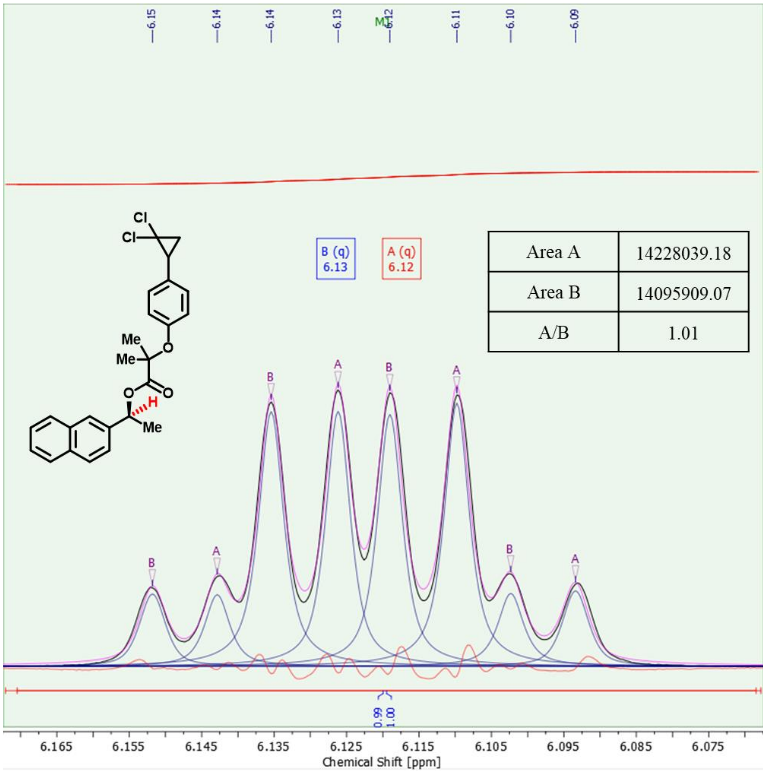


Analysis of $^{1}$H NMR feature of the indicated H, two quartets of equal area were observed.

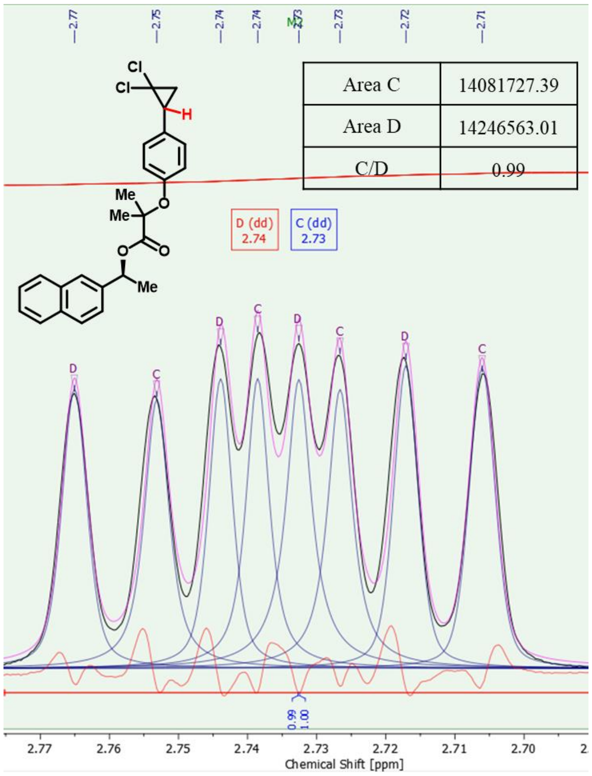


Analysis of $^{1}$H NMR feature of the indicated H, two doublets of doublet of equal area were observed.

(*S*)-1-(naphthalen-2-yl)ethyl 6-3-adamantan-1-yl-4-methoxyphenyl)-2-naphthoate (**3al**)

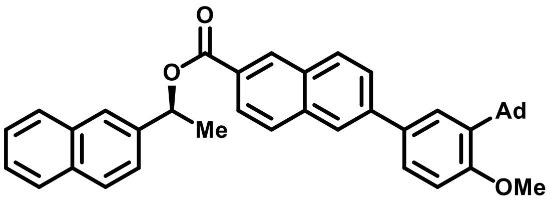


Reaction carried out in DCE (2.5 mL). The residue was purified by reverse-phase column chromatography on C18 stationary phase (100 g cartridge, 20% to 100% MeCN in $H_2O$) to yield the titled compound (167 mg, 59%, 98:2 er) as a colorless oil.

**$^1$H NMR** (400 MHz; $CDCl_3$) δ 8.68 (s, 1H), 8.14 (dd, *J* = 8.6, 1.5 Hz, 1H), 8.05–7.98 (m, 2H), 7.96–7.80 (m, 6H), 7.67–7.62 (m, 2H), 7.56 (dd, *J* = 8.4, 2.2 Hz, 1H), 7.52–7.46 (m, 2H), 7.00 (d, *J* = 8.5 Hz, 1H), 6.39 (q, *J* = 6.6 Hz, 1H), 3.91 (s, 3H), 2.22–2.20 (m, 6H), 2.14–2.10 (m, 3H), 1.85–1.81 (m, 9H).

**$^{13}$C NMR** (101 MHz; CDCl3) δ 166.24, 159.06, 141.51, 139.33, 139.14, 136.13, 133.38, 133.22, 132.69, 131.39, 131.03, 129.86, 128.61, 128.35, 128.23, 127.83, 127.41, 126.60, 126.38, 126.22, 126.11, 125.87, 125.82, 125.22, 124.87, 124.31, 112.25, 73.29, 55.30, 40.75, 37.36, 37.27, 29.25, 22.51.

**IR** (Diamond-ATR, neat) $\bar{\nu}$ ($cm^{-1}$): 2903, 2848, 1712, 1629, 1601, 1474, 1277, 1216, 1179, 1093

**Specific Rotation** $[\alpha]^{27}_D$: 130.20 (*c* = 1.0, $CHCl_3$)

**HRMS** (ESI) m/z: $[M+H]^+$ calcd for $C_{40}H_{39}O_3^+$: 567.2894. Found: 567.2890.

**Chiral HPLC (**Chiralcel AD-3, hexane/IPA = 90/10 over 30 min, 1.0 mL/min, 214 nm, 25 °C), 4.20 (minor), 5.41 (major)

*Synthesis of immunosuppressant **8***

*Step a*:

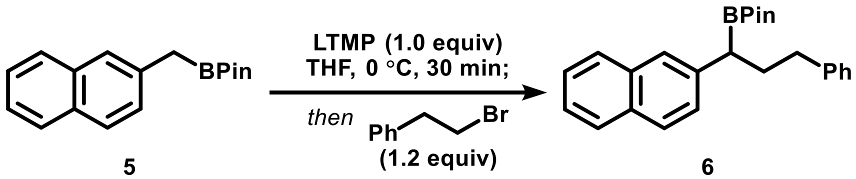


**5**
(1.0 equiv)

**6**
86% yield

Alkylborane **6** is prepared according to literature procedure on a 10.0 mmol scale.[5] Purification by column chromatography (100 g $SiO_2$) afforded the titled compound (3.20 g, 86%) as a white solid.

**$^1$H NMR** (400 MHz; $CDCl_3$) δ 7.83–7.74 (m, 3H), 7.65 (s, 1H), 7.46–7.37 (m, 3H), 7.28–7.24 (m, 2H), 7.19–7.13 (m, 3H), 2.63–2.57 (m, 2H), 2.56–2.49 (m, 1H), 2.30–2.19 (m, 1H), 2.15–2.03 (m, 1H), 1.21 (s, 6H), 1.19 (s, 6H).

**$^{13}$C NMR** (101 MHz; $CDCl_3$) δ 142.66, 140.64, 133.96, 131.96, 128.66, 128.39, 127.95, 127.69, 127.61, 127.52, 126.52, 125.82, 125.80, 124.99, 83.57, 35.57, 34.25, 32.35, 24.83, 24.74.

**HRMS** (DART) m/z: $[M+H]^+$ calcd. for $C_{25}H_{30}BO_2^+$: 373.2333. Found: 373.2345.

*Step b*:

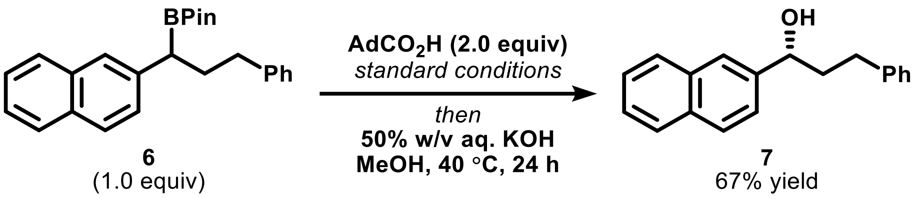


**6**
(1.0 equiv)

**7**
67% yield

Enantiomerically enriched alcohol **7** was prepared using an one-pot procedure. Inside a nitrogen-filled glovebox, an oven-dried reaction tube (Fisherbrand, 16 × 125 mm, catalog no. 1495935A) equipped with a magnetic stir bar was charged with CuCl (5.0 mg, 0.050 mmol, 10 mol%), $NaBAr^F{}_4$ (66.5 mg, 0.075 mmol, 15 mol%), and **L6'** (the enantiomer of **L6**, 73.5 mg, 0.075 mmol, 15 mol%) followed by the addition of a 1:1 mixture of PhCl and PhMe (2.5 mL). The reaction mixture was stirred at room temperature for 10 min before the addition of **4e** (14.0 mg ,0.050 mmol, 10 mol%), alkylborane **6** (186 mg, 0.50 mmol, 1.0 equiv), and 1-adamantanecarboxylic acid **2b** (180 mg, 1.00 mmol, 2.0 equiv). The reaction mixture was stirred for 10 min before di-*tert*-butyl peroxide (183 μL, 1.00 mmol, 2.0 equiv) was added in one portion. The reaction tube was sealed with a screw cap (Kimble Chase Open Top S/T Closure, catalog no. 73804-15425)

containing a PTFE septum (Thermo Scientific, catalog no. B7995-15), taken outside of the glovebox, placed in a −20 °C freezer and irradiated with 427 nm light (Kessil P160L) with stirring.

After the reaction mixture had stirred for 16 h, the screw cap was removed from the reaction tube, and the reaction mixture was concentrated *in vacuo* with the aid of a rotary evaporator. The residue was dissolved in 5 mL of $Et_2O$, and the resulting suspension was filtered. The remaining solid was washed with 3 × 5 mL of $Et_2O$. The organic filtrate was combined in a 50 mL flask and concentrated *in vacuo* with the aid of a rotary evaporator.

The hydrolysis was carried out following an adapted literature procedure.[9] The residue was dissolved in 0.60 mL of MeOH followed by the addition of 50% w/v aq. KOH (0.60 mL, 5.0 mmol). The resulting mixture was left stirring in a preheated 40 °C oil bath for 24 h. The reaction mixture was concentrated *in vacuo* with the aid of a rotary evaporator, diluted with EtOAc (50 mL), and extracted with 5% $NaHCO_3$ (3 × 20 mL). The organic layer was concentrated and purified by reverse-phase (C18) column chromatography to yield alcohol **7** (88 mg, 67%, 94:6 er) as a colorless oil.

**$^1$H NMR** (400 MHz; $CDCl_3$) δ 7.86–7.77 (m, 3H), 7.75 (s, 1H), 7.52–7.41 (m, 3H), 7.32–7.24 (m, 2H), 7.23–7.14 (m, 3H), 4.82 (dd, $J$ = 7.9, 5.4 Hz, 1H), 2.81–2.60 (m, 2H), 2.28–2.03 (m, 3H).

**$^{13}$C NMR** (101 MHz; $CDCl_3$) δ 141.99, 141.86, 133.39, 133.13, 128.58, 128.52, 128.48, 128.05, 127.81, 126.29, 125.99, 125.97, 124.80, 124.17, 74.06, 40.42, 32.15.

**Specific Rotation** $[\alpha]^{27}_{D}$: 5.10 ($c$ = 0.3, $CHCl_3$)

**Chiral HPLC (**Chiralcel AD-3, hexane/IPA = 97/3 over 60 min, 1.0 mL/min, 214 nm, 25 °C), 30.25 (minor), 32.20 (major)

Spectroscopic data is consistent with reported values.[10]

*Step c*:

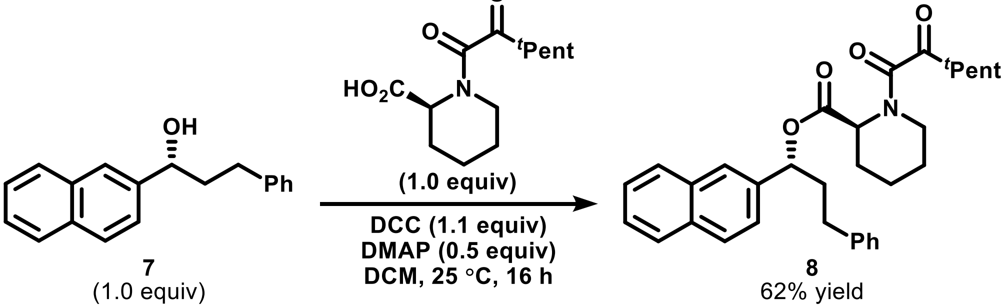


Immunosuppressant **8** was synthesized according to literature procedure on a 0.3 mmol scale.[11, 12] Inside a nitrogen-filled glovebox, an oven-dried reaction tube (Fisherbrand, 16 × 125 mm,

catalog no. 1495935A) equipped with a magnetic stir bar was charged with alcohol **7** (78.7 mg, 0.30 mmol, 1.0 equiv), (*S*)-1-(3,3-dimethyl-2-oxopentanoyl)piperidine-2-carboxylic acid (prepared according to literature procedure[11, 12]) (76.6 mg, 0.30 mmol, 1.0 equiv), DCC (61.9 mg, 0.30 mmol, 1.0 equiv), and DMAP (36.7 mg, 0.15 mmol, 0.5 equiv) before the addition of DCM (1.0 mL). The mixture was left stirring at 25 °C for 16 h. The screw cap was then removed from the reaction tube, and the reaction mixture was concentrated *in vacuo* with the aid of a rotary evaporator. The residue was purified by reverse-phase (C18) column chromatography to yield compound **8** (93 mg, 62%, 16:1 dr) as a light yellow oil.

While the original report did not detail the spectroscopic features of **8**, analogous compounds were reported as a 4:1 mixture of *trans*-*cis* amide rotamers.[11, 12] Similar features were observed with **8**. A *ca*. 4:1 ratio between two rotamer was observed.

**$^1$H NMR** (400 MHz; $CDCl_3$) δ (*major rotamer*) 7.87–7.82 (m, 4H), 7.52–7.49 (m, 2H), 7.48–7.47 (m, 1H), 7.30–7.26 (m, 2H), 7.20–7.14 (m, 3H), 6.00 (dd, *J* = 7.9, 1.9 Hz, 1H), 5.38–5.34 (m, 1H), 3.39–3.31 (m, 1H), 3.16–3.09 (m, 1H), 2.71–2.66 (m, 1H), 2.63–2.57 (m, 1H), 2.41–2.35 (m, 2H), 2.23–2.18 (m, 1H), 1.77–1.72 (m, 2H), 1.72–1.67 (m, 2H), 1.61–1.56 (m, 1H), 1.52–1.43 (m, 1H), 1.36–1.31 (m, 1H), 1.24 (s, 3H), 1.21 (s, 3H), 0.91–0.87 (m, 3H). Due to overlapping signals, only well-isolated peaks are listed below for the minor rotamer: (*minor rotamer*) 7.78 (s, 1H), 7.44 (dd, *J* = 8.5, 1.9 Hz, 1H), 4.48–4.41 (m, 1H), 4.28–4.25 (m, 1H), 3.51–3.46 (m, 1H), 2.90–2.83 (m, 1H), 2.31–2.26 (m, 1H), 1.07 (s, 3H), 1.05 (s, 3H), 0.76–0.69 (m, 3H).

**$^{13}$C NMR** (101 MHz; $CDCl_3$) (*major rotamer*) δ 207.97, 169.90, 167.37, 141.04, 137.07, 133.34, 133.28, 128.74, 128.62, 128.51, 128.27, 127.83, 126.49, 126.42, 126.32, 126.23, 124.26, 77.22, 51.41, 46.86, 44.30, 37.87, 32.62, 31.86, 26.55, 25.10, 23.71, 23.21, 21.31, 8.90. (*minor rotamer*) 207.83, 169.88, 166.76, 140.98, 136.86, 133.37, 133.23, 128.76, 128.66, 128.49, 128.23, 127.83, 126.54, 126.38, 126.32, 126.29, 124.21, 77.55, 56.90, 49.33, 46.76, 38.95, 32.64, 31.92, 27.83, 24.61, 23.69, 23.43, 21.22, 8.84.

**Specific Rotation** $[\alpha]^{27}_{D}$: 48.0 (*c* = 1.0, $CHCl_3$)

**IR** (Diamond-ATR, neat) $\bar{\nu}$ ($cm^{-1}$): 3054, 2930, 1736, 1700, 1633, 1598, 1550, 1465, 1445, 1261, 1245, 1219, 1082, 1016, 944, 731

**HRMS** (DART) m/z: $[M+H]^+$ calcd. for $C_{32}H_{38}NO_4^+$: 500.2795. Found: 500.2797.

**Diastereomeric Excess:** A mixture of two diastereomers **8/8'** was synthesized using racemic mixture of **7** to aid the d.r. determination *via* $^1$H NMR analysis.

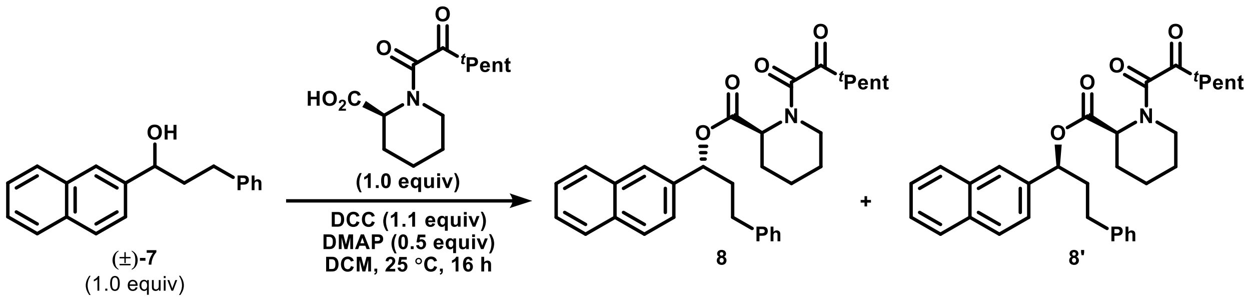


The synthesis of **8**/**8’** diastereomeric mixture was carried out on a 0.1 mmol scale following the same procedure for *step c*. The product was purified by preparative thin-layer chromatography and revealed a ratio **8**/**8’** = 2.6, likely the result of kinetic resolution and incomplete consumption of **(±)-7**.

*Representative $^{1}$H NMR features of **8**/**8’** mixture*

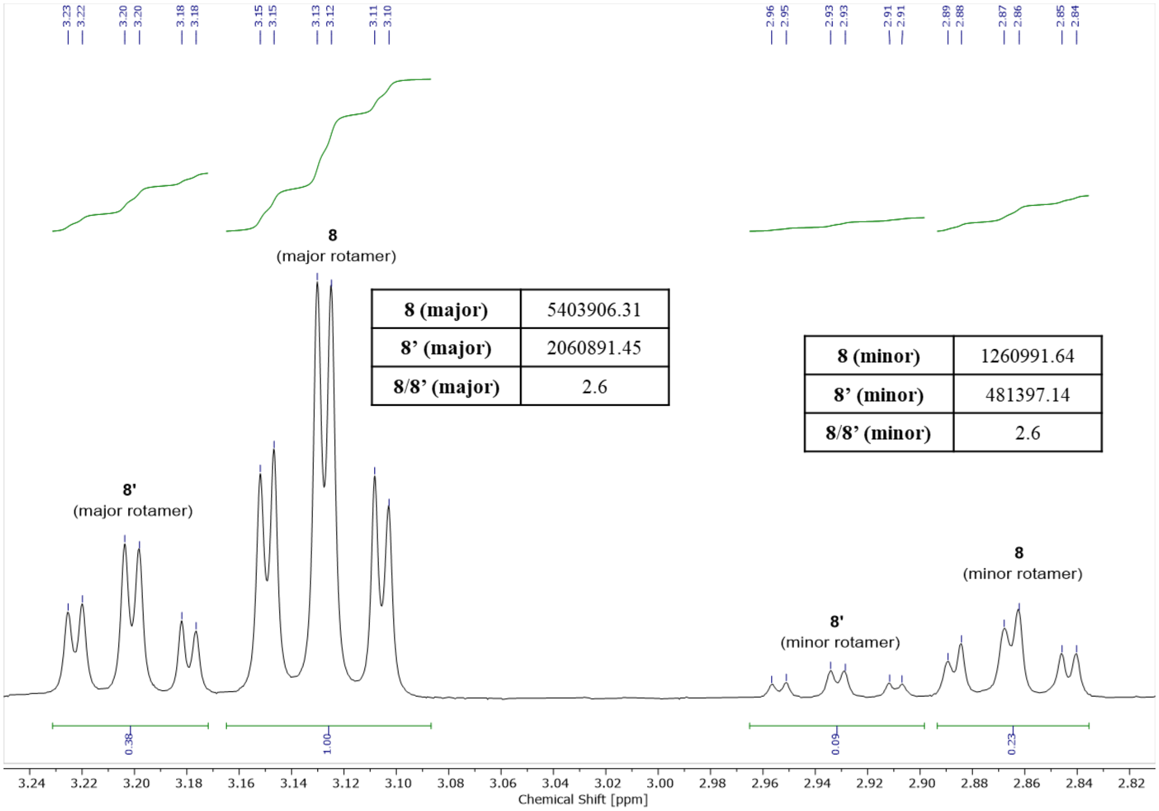

These features were used to analyze the d.r. of **8**:

*Representative $^{1}$H NMR features of sample **8***

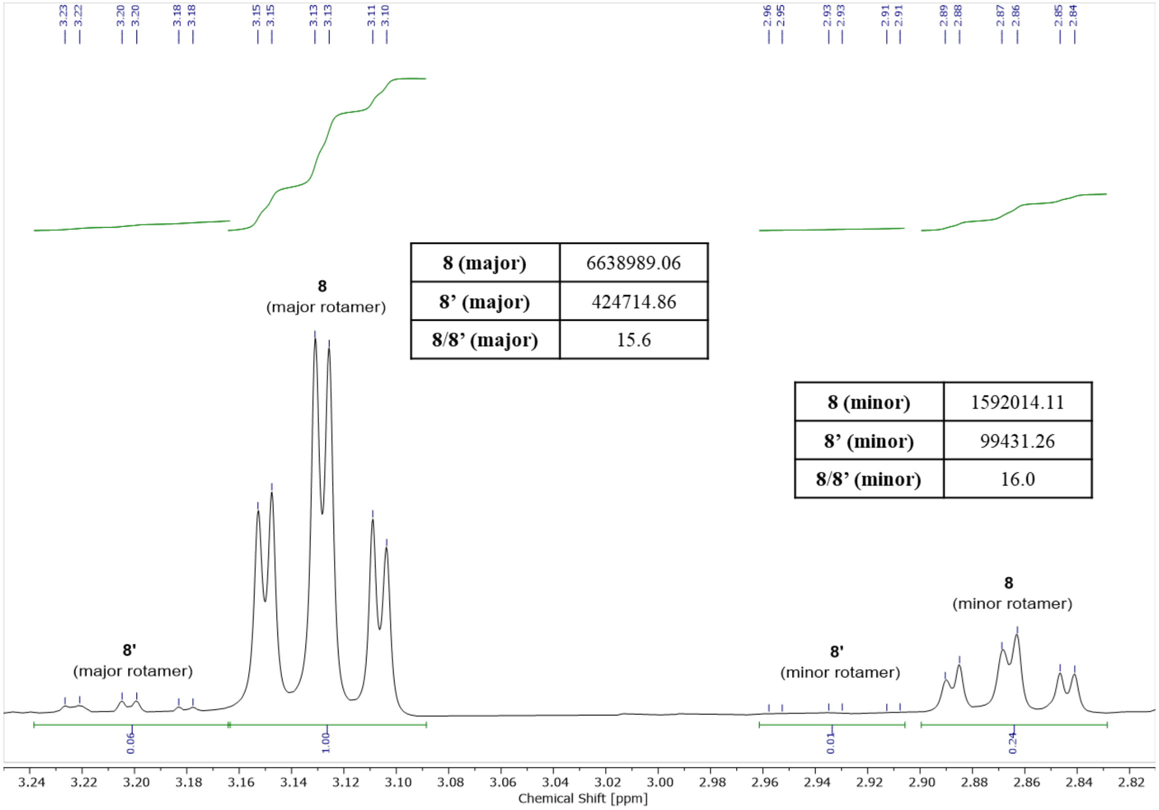


- Based on integration ratio of the major rotamers, a d.r. = 16:1 was determined, consistent with the level of enantioenrichment of starting material **7**.
- The result is consistent with the analysis from other $^{1}$H NMR features.

## 6. Crystallographic Information

X-ray diffraction techniques: Structures of **3f**, **3ai**, **$16^+(BAr^F{}_4)^-$**, and **$18^+(PF_6)^-$** were collected on a Bruker three-circle platform goniometer equipped with a Photon 50™ CMOS detector and an Oxford cryostream cooling device. Radiation was from a graphite fine focus sealed tube Mo Kα (0.71073 Å) source. Crystals were mounted on a cryoloop or glass fiber pin using Paratone N oil. Structures were collected at 100 K. Data were collected as a series of φ and/or ω scans. Data were integrated using SAINT and scaled with multi-scan absorption correction using SADABS.[13, 14] The structures were solved by intrinsic phasing, direct methods or Patterson maps using SHELXS-2014 and refined against $F^2$ on all data by full matrix least squares with SHELXL-2014.[13, 14] All nonhydrogen atoms were refined anisotropically. Hydrogen atoms were placed at idealized positions and refined using a riding model. The isotropic displacement parameters of all hydrogen atoms were constrained to be 1.2 times the parameter of the atoms they were linked to (1.5 times for methyl groups). Further details on particular structures are noted below.

**3f**: The structure was solved in the tetragonal space group $P2_1$ with two molecules per unit cell and one molecule in the asymmetric unit. (CCDC 2505993)

**3ai**: The structure was solved in the monoclinic space group $P2_1$ with two molecules per unit cell and one molecule in the asymmetric unit. (CCDC 2505992)

**$16^+(BAr^F{}_4)^-$**: The structure was solved in the tetragonal space group $P2_1$ with eight molecules per unit cell and four molecules in the asymmetric unit. (CCDC 2505995)

**$18^+(PF_6)^-$**: The structure was solved in the monoclinic space group $P2_12_12_1$ with four molecules per unit cell and one molecule in the asymmetric unit. (CCDC 2505994)

**Table S5. X-ray diffraction experimental details**

| | **3f** (CCDC:2505993) | **3ai** (CCDC: 2505992) | **$16^{+}(BAr^{F}{}_{4})^{-}$** (CCDC:2505995) | **$18^{+}(PF_6)^{-}$** (CCDC:2505994) |
|---|---|---|---|---|
| **Moiety Formula** | $C_{24}H_{20}O_2S$ | $C_{31}H_{26}NO_4Cl$ | $C_{67}H_{45}BCuF_{24}N_3O_2$ | $C_{40}H_{35}CuF_6N_2O_4P$ |
| **FW** | 372.46 | 511.98 | 1454.41 | 816.21 |
| **$\lambda$ (Å)** | 0.71073 | 0.71073 | 0.71073 | 0.71073 |
| **$T$ (K)** | 100(2) | 156(2) | 156(2) | 141(2) |
| **Crystal System** | Monoclinic | Monoclinic | Monoclinic | Orthorhombic |
| **Space Group** | $P2_1$ | $P2_1$ | $P2_1$ | $P2_12_12_1$ |
| **$a$ (Å)** | 9.698(2) | 14.835(14) | 18.141(2) | 14.1556(9) |
| **$b$ (Å)** | 5.7954(14) | 5.226(4) | 39.573(5) | 18.9369(12) |
| **$c$ (Å)** | 16.788(4) | 17.420(13) | 18.231(2) | 13.9517(9) |
| **$\alpha$ (°)** | 90 | 90 | 90 | 90 |
| **$\beta$ (°)** | 94.597(6) | 112.846(19) | 90.939(3) | 90 |
| **$\gamma$ (°)** | 90 | 90 | 90 | 90 |
| **Volume (Å$^3$)** | 940.6(4) | 1244.6(18) | 13086(3) | 3739.9(4) |
| **Calc. $\rho$ (mg/m$^3$)** | 1.315 | 1.366 | 1.318 | 1.450 |
| **$\mu$ (mm$^{-1}$)** | 0.188 | 0.193 | 0.449 | 0.701 |
| **Reflections** | 3374 | 4431 | 47425 | 6704 |
| **Completeness (to 2$\theta$)** | 99% 25.168 | 96% 25.060 | 97% 25.250 | 100% |
| **GOF on F$^2$** | 1.082 | 1.124 | 1.032 | 1.031 |
| **R1, $w$R2$^{a}$ [$I > 2\sigma(I)$]** | 0.0691, 0.1627 | 0.1469, 0.4136 | 0.0723, 0.1983 | 0.0455, 0.1149 |

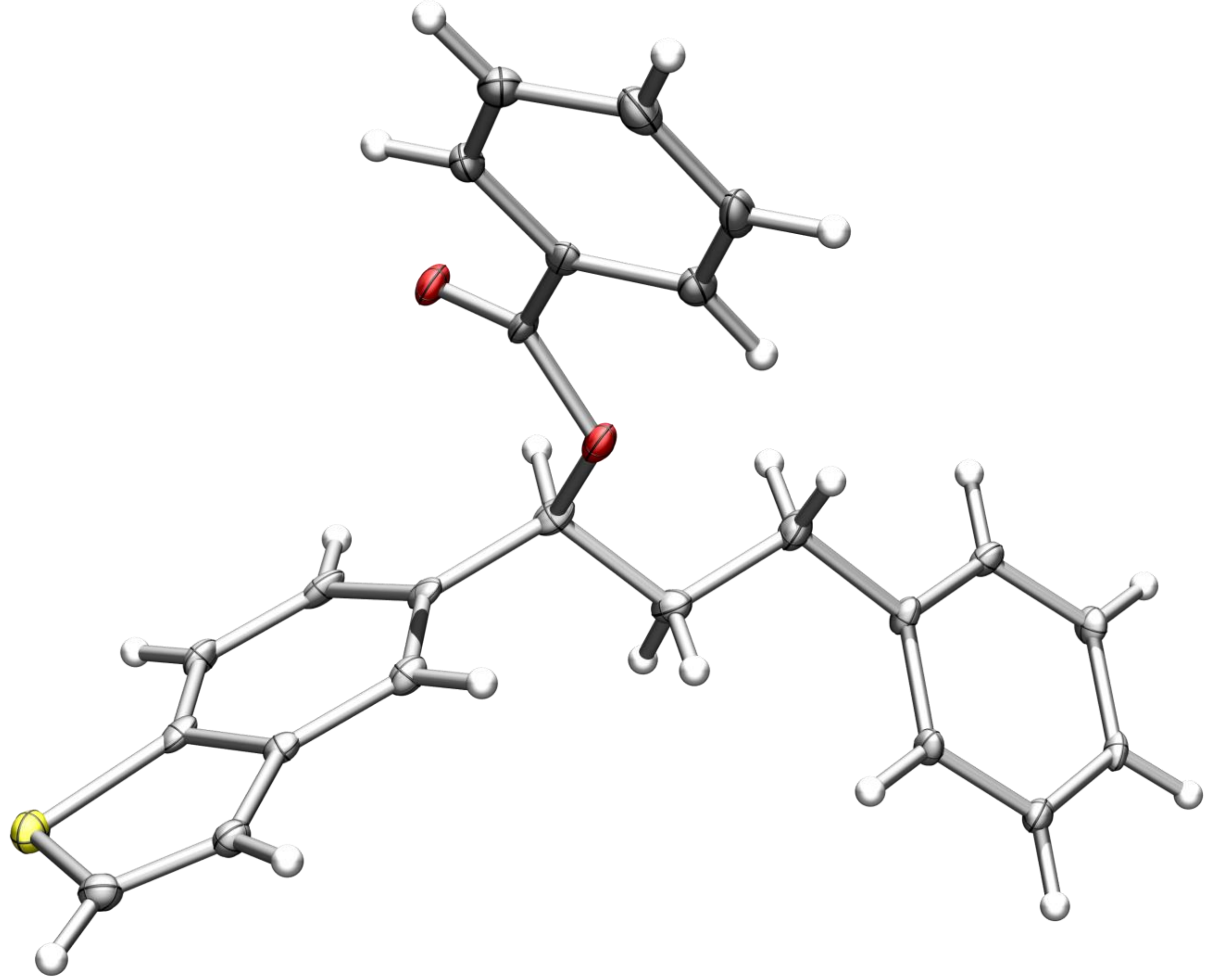

**Figure S3**. Solid-state structure of **3f** with thermal ellipsoids at 50% probability level. carbon = gray, oxygen = red, sulfur = yellow, hydrogen = white.

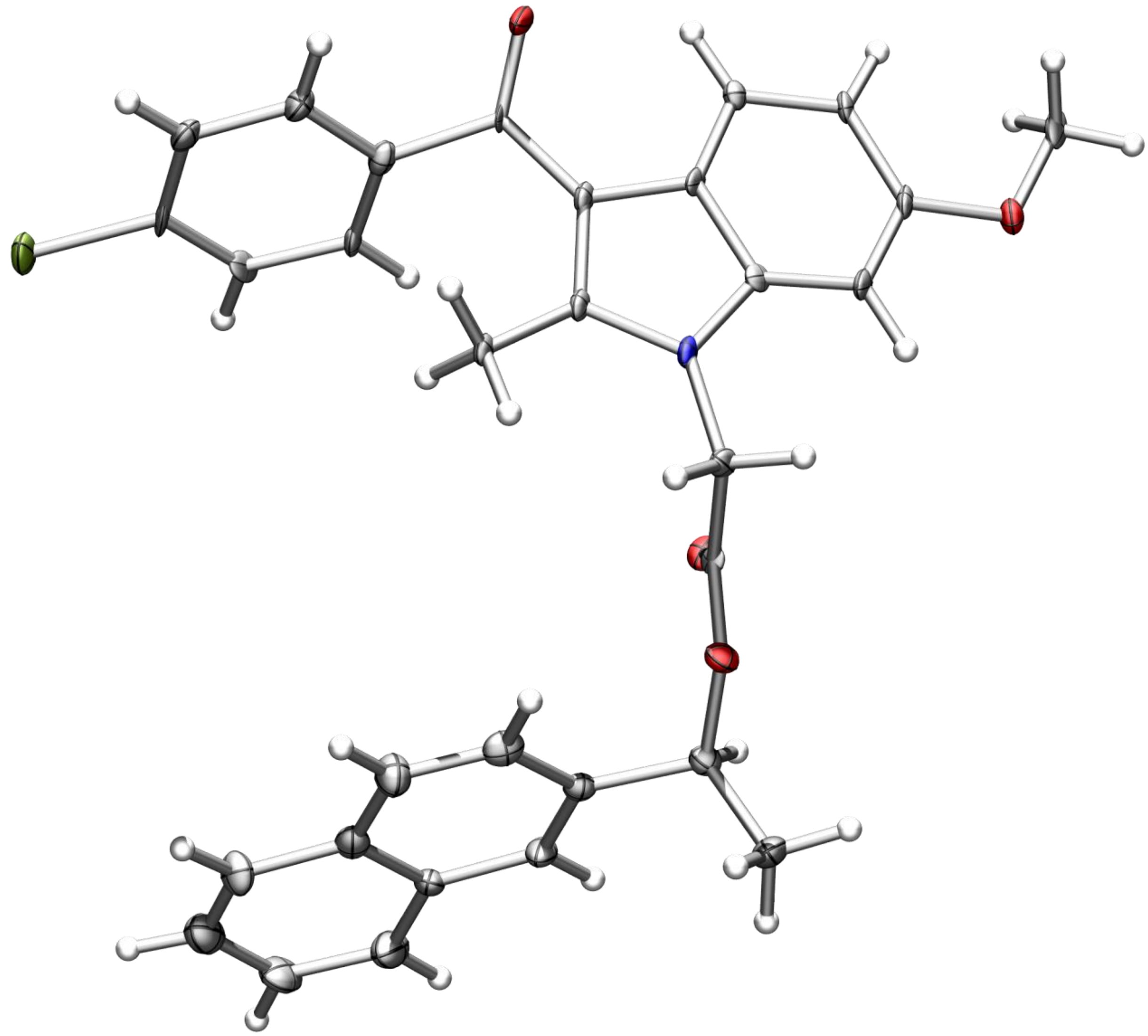

**Figure S4**. Solid-state structure of complex **3ai** with thermal ellipsoids at 50% probability level. carbon = gray, oxygen = red, nitrogen = blue, chlorine = green, hydrogen = white.

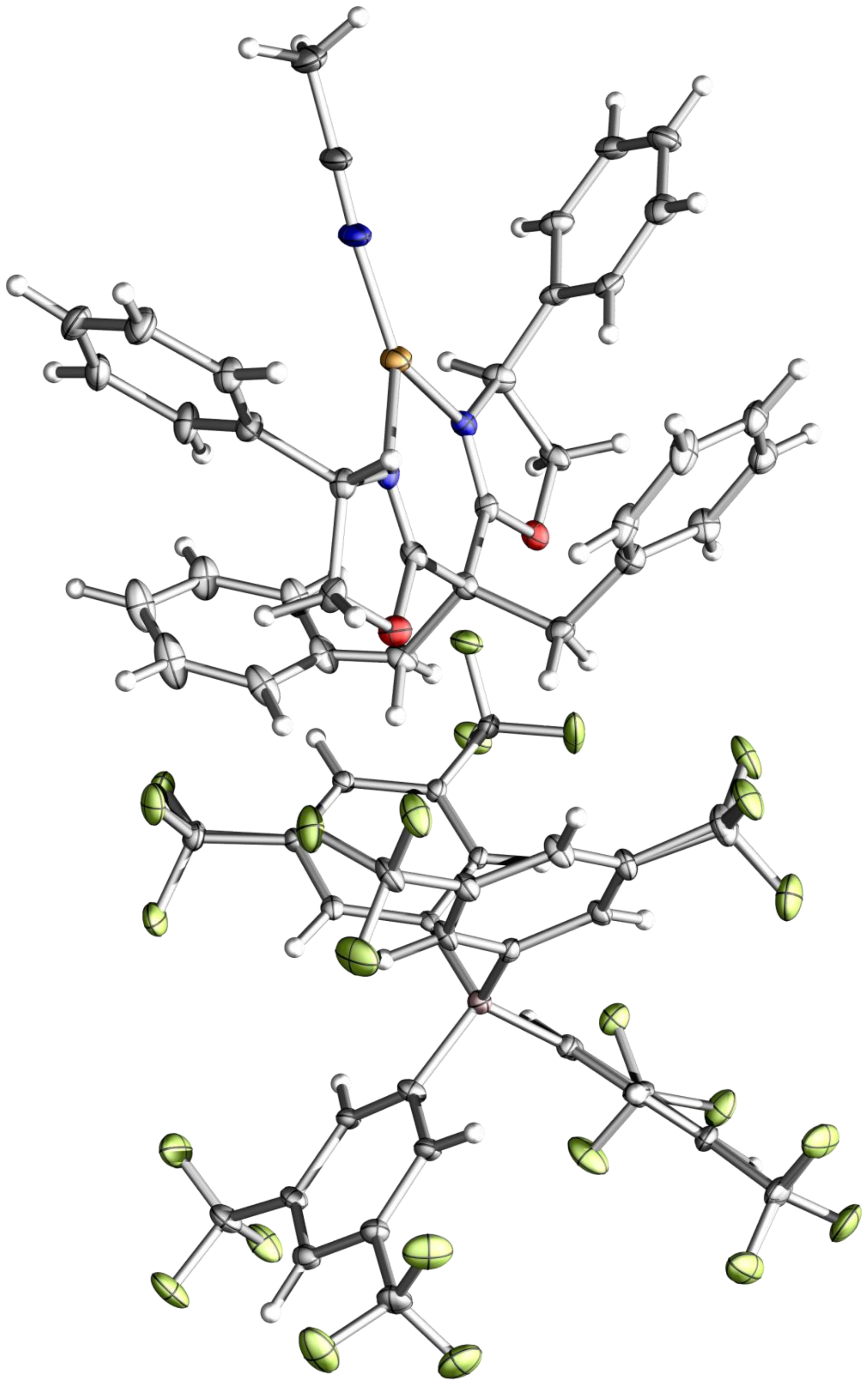

**Figure S5**. Solid-state structure of complex **$16^{+}(BAr^{F}_{4})^{-}$** with thermal ellipsoids at 50% probability level. carbon = gray, oxygen = red, nitrogen = blue, copper = gold, fluorine = yellowgreen, boron = pink, hydrogen = white.

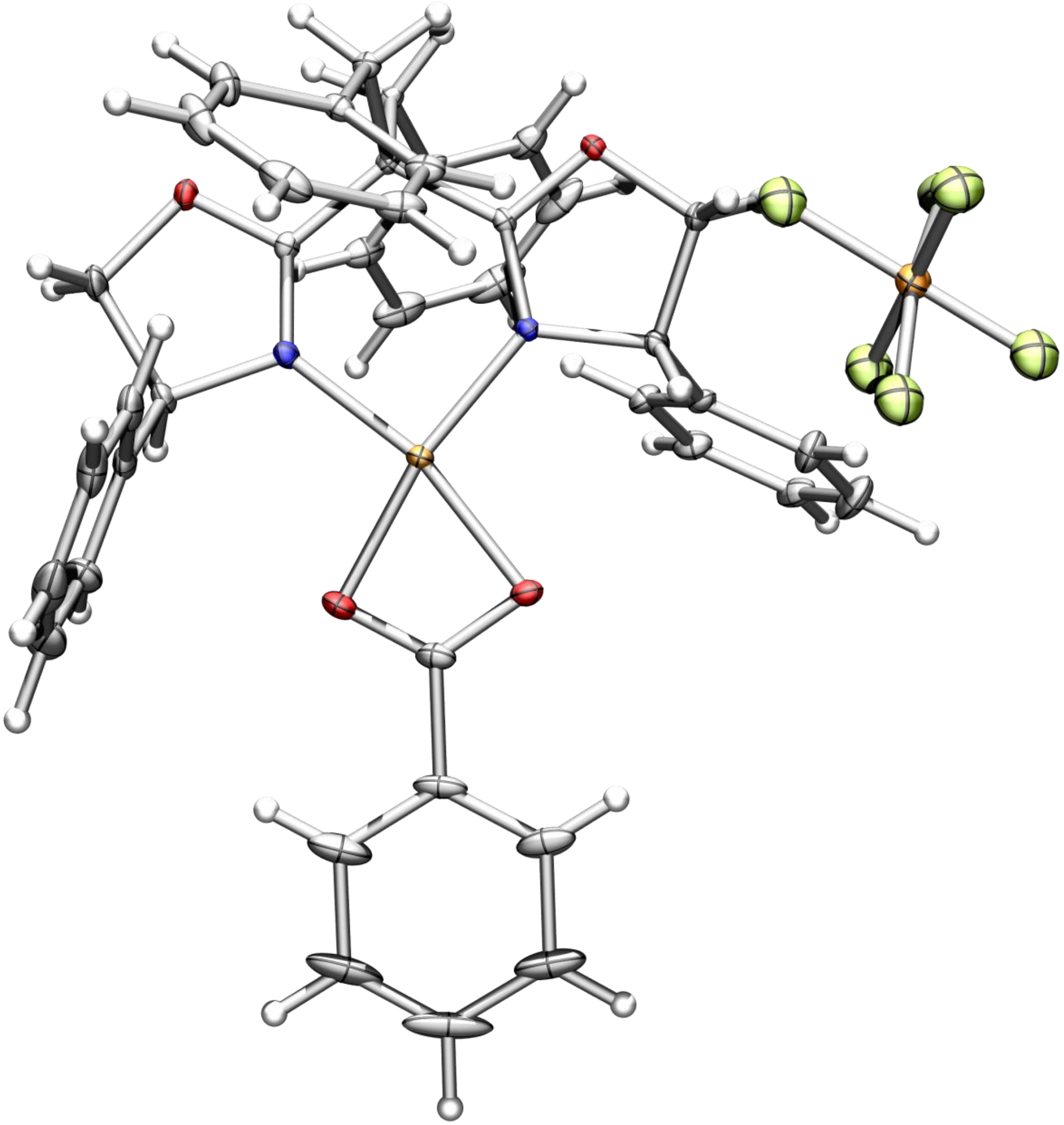

**Figure S6**. Solid-state structure of complex **$18^{+}(PF_6)^{-}$** with thermal ellipsoids at 50% probability level. carbon = gray, oxygen = red, nitrogen = blue, copper = gold, fluorine = yellowgreen, phosphorous = orange, hydrogen = white.

## 7. Experimental Procedures for Mechanistic Investigations

### 1) Radical Trapping Experiments with TEMPO

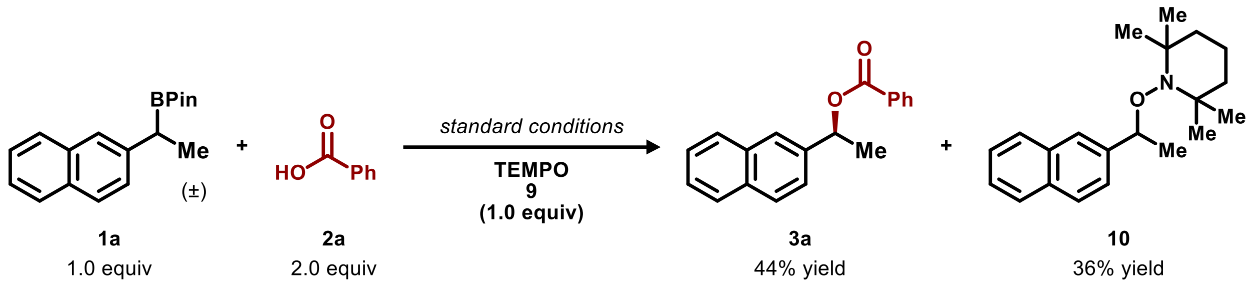


Inside a nitrogen-filled glovebox, an oven-dried reaction tube (Fisherbrand, 16 × 125 mm, catalog no. 1495935A) equipped with a magnetic stir bar was charged with CuCl (1.0 mg, 0.010 mmol, 10 mol%), $NaBAr^F{}_4$ (13.3 mg, 0.015 mmol, 15 mol%), and **L6** (14.7 mg, 0.015 mmol, 15 mol%) followed by the addition of a 1:1 mixture of PhCl and PhMe (0.5 mL). The reaction mixture was stirred at room temperature for 10 min before the addition of **4e** (2.8 mg ,0.010 mmol, 10 mol%), alkylborane **1a** (28.2 mg, 0.10 mmol, 1.0 equiv), and carboxylic acid **2a** (24.4 mg, 0.20 mmol, 2.0 equiv). The reaction mixture was stirred for 10 min before di-*tert*-butyl peroxide (36.6 µL, 0.20 mmol, 2.0 equiv) and TEMPO (**9**, 15.6 mg, 0.10 mmol, 1.0 equiv) was added. The reaction tube was sealed with a screw cap (Kimble Chase Open Top S/T Closure, catalog no. 73804-15425) containing a PTFE septum (Thermo Scientific, catalog no. B7995-15), taken outside of the glovebox, placed in a –20 °C freezer and irradiated with 427 nm light (Kessil P160L) with stirring.

After the reaction mixture had stirred for 16 h, the screw cap was removed from the reaction tube, and the reaction mixture was concentrated *in vacuo* with the aid of a rotary evaporator. The resulting crude material was analyzed by $^1$H NMR spectroscopy using 1,1,2,2-tetrachloroethane as an internal standard. A small portion of the mixture was purified by preparative thin layer chromatography to confirm the product identity. Compound **3a** was formed in 44% yield based on the $^1$H NMR integration. Chiral HPLC analysis of a purified sample of **3a** revealed that the product was formed with 97:3 er. TEMPO-adduct **10** was formed in 36% yield based on the $^1$H NMR integration. The identity of **10** was confirmed by comparing the $^1$H NMR features of a purified sample to reported values:

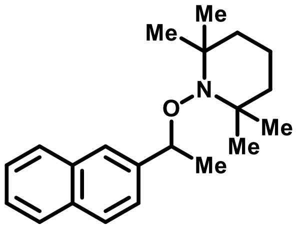


[1]H NMR (400 MHz, $CDCl_3$) δ 7.86–7.78 (m, 3H), 7.72 (s, 1H), 7.51 (dd, $J$ = 8.6, 1.8 Hz, 1H), 7.49–27.39 (m, 1H), 4.94 (q, $J$ = 6.7 Hz, 1H), 1.61–1.48 (m, 6H), 1.34 (s, 3H), 1.21 (s, 3H), 1.04 (s, 3H), 0.62 (s, 3H).

The features are consistent with reported values.[15]

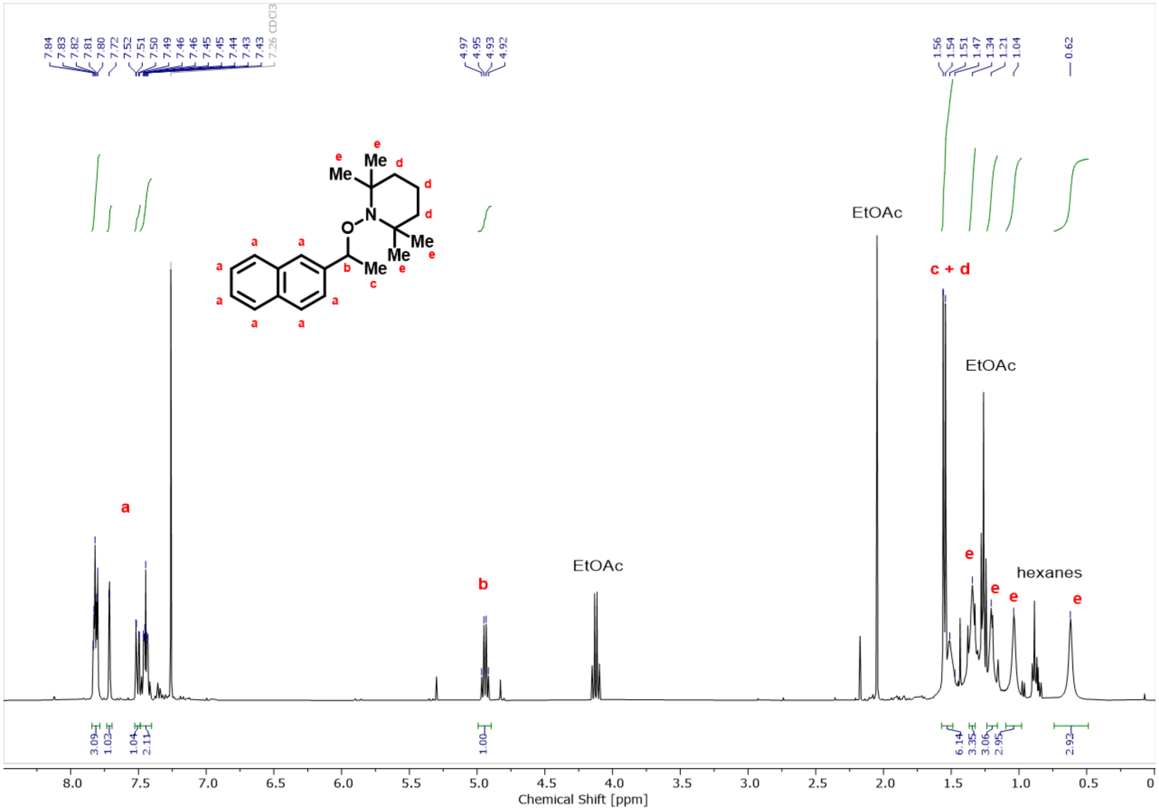


*Note*: With 2.0 equiv of TEMPO, increased yield of **10** (65% yield) was observed. No formation of **3a** was detected. With 3.0 equiv of TEMPO, no further significant increase in yield of **10** (71% yield) was observed. No formation of **3a** was detected.

**2) Reaction Order Determination**

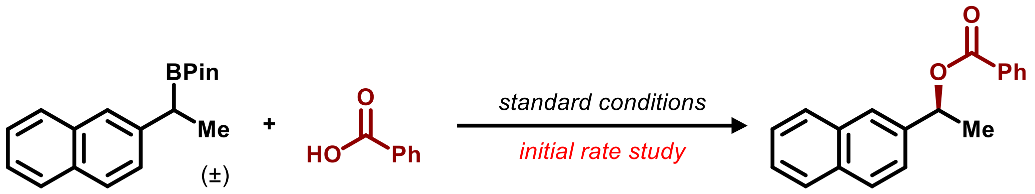


**1a** 0.20 M **2a** 0.40 M **3a**

*General procedure*: Inside a nitrogen-filled glovebox, an oven-dried reaction tube (Fisherbrand, 16 × 125 mm, catalog no. 1495935A) equipped with a magnetic stir bar was charged with specified mixture of starting materials to measure the reaction order in each individual component: (*a*) light intensity, (*b*) [($^t$BuO)$_2$], (*c*) [**4e**], (*d*) [**1a**], (*e*) [**2a**], and (*f*) [Cu]. 1,3,5-trimethoxybenzene (16.8 mg, 0.10 mmol) was added in each reaction as an internal standard. The reaction tube was sealed with a screw cap (Kimble Chase Open Top S/T Closure, catalog no. 73804-15425) containing a PTFE septum (Thermo Scientific, catalog no. B7995-15), taken outside of the glovebox, placed in a −20 °C freezer and irradiated with 427 nm light (Kessil P160L) with stirring.

A 50 μL aliquot was taken using a 100 μL glass microsyringe by piercing through the septum after 3, 6, 9, 15, and 30 min. Solvent was removed from these aliquots *in vacuo*, and the residue was analyzed using $^1$H NMR spectroscopy with 1,3,5-trimethoxybenzene as the internal standard. Initial rates for each reaction (measured using the first 15% of conversion) were plotted against the concentration of each reaction component of interest to derive the reaction order.

*Note*: For reproducibility, a stock solution for Cu catalyst was prepared as the following: Inside a nitrogen-filled glovebox, a 20 mL scintillation vial equipped with a magnetic stir bar was charged with CuCl (24.7 mg, 0.25 mmol, 1.0 equiv) and **L6** (372 mg, 0.38 mmol, 1.5 equiv), followed by 12.5 mL of PhMe:PhCl (1:1) solvent mixture. The mixture was stirred at 25 °C till the complete dissolution of all solids (*ca*. 30 min). This stock solution of (**L6**)CuCl was stored in a −30 °C freezer in the glovebox before use.

*a) Reaction order in light intensity*

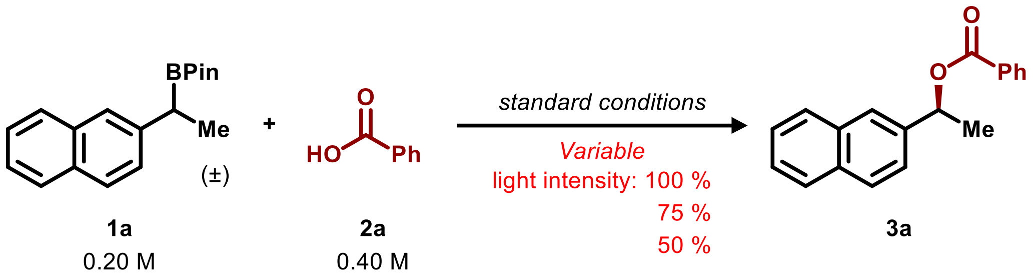


The light intensity on the Kessil blue LED (max 45W) was tuned to 100%, 75%, and 50%. At 25% the light intensity was too low for productive catalysis.

*Result*:

| Intensity | Initial Rate (M/s) | Standard Deviation (M/s) |
|---|---|---|
| 100% | 2.49841e-05 | 1.03447e-06 |
| 75% | 1.56667e-05 | 7.76004e-07 |
| 50% | 1.11429e-05 | 2.87034e-07 |

*Note*: Error bar represents the standard deviation from repeating the experiment in triplicate.

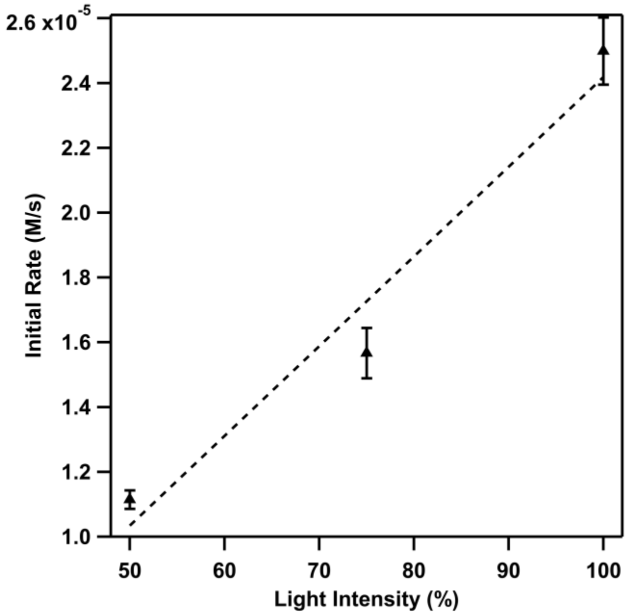


Result suggests the reaction is first order in light intensity.

*b) Reaction order in* [($^t$BuO)$_2$]

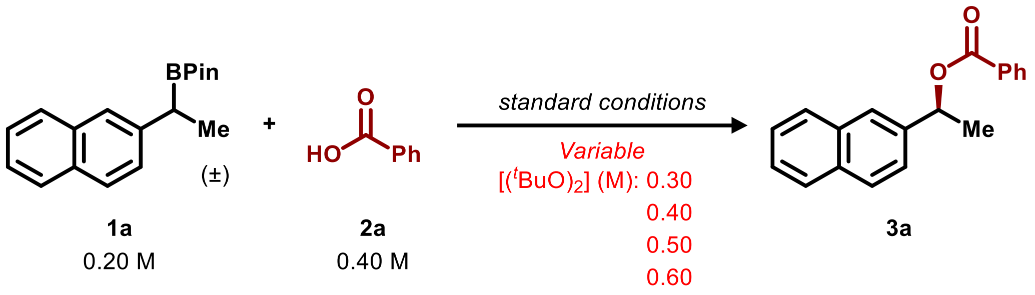


*Result*:

| [($^t$BuO)$_2$] (M) | Initial Rate (M/s) | Standard Deviation (M/s) |
|---|---|---|
| 0.3 | 1.17323e-05 | 1.12545e-06 |
| 0.4 | 1.66835e-05 | 5.87672e-07 |
| 0.5 | 2.22128e-05 | 2.92357e-07 |
| 0.6 | 2.51937e-05 | 5.24244e-07 |

*Note*: Error bar represents the standard deviation from repeating the experiment in triplicate.

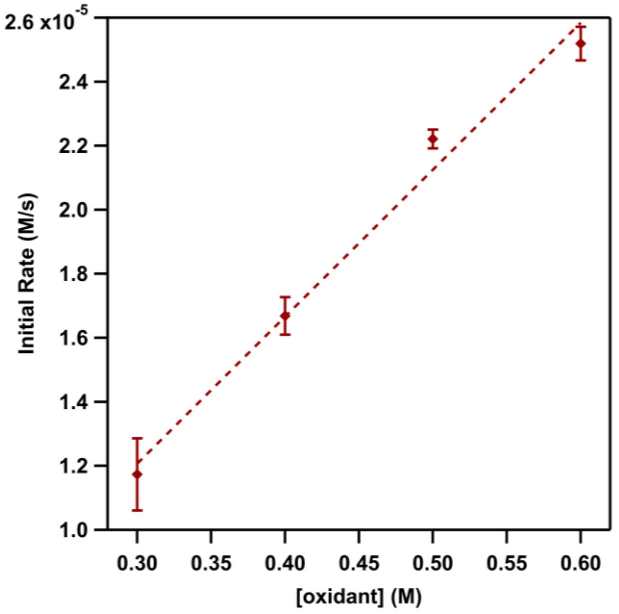


Result suggests the reaction is first order in oxidant concentration.

*c) Reaction order in* [**4e**]

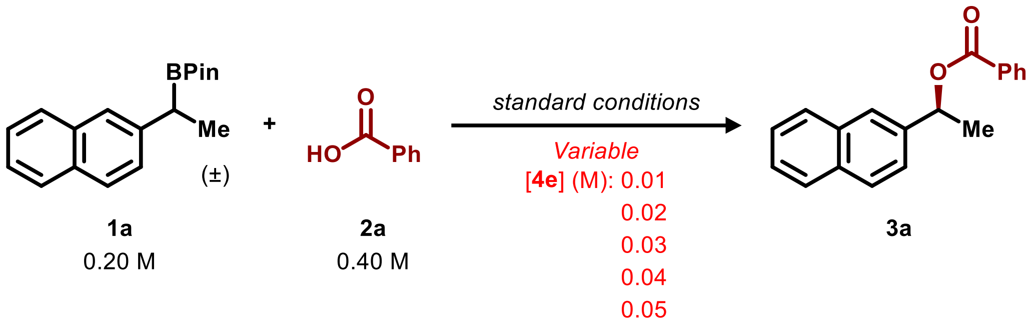


*Result*:

| [**4e**] (M) | Initial Rate (M/s) | Standard Deviation (M/s) |
|---|---|---|
| 0.01 | 1.30379e-05 | 1.22489e-06 |
| 0.02 | 1.67859e-05 | 1.39723e-06 |
| 0.03 | 2.88954e-05 | 3.39253e-06 |
| 0.04 | 3.74647e-05 | 2.99191e-06 |
| 0.05 | 4.24627e-05 | 5.35676e-06 |

*Note*: Error bar represents the standard deviation from repeating the experiment in triplicate.

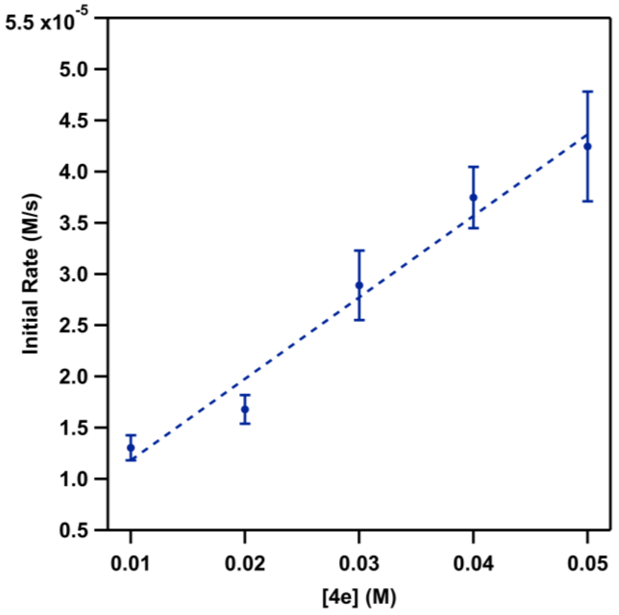


Result suggests the reaction is first order in additive **4e** concentration.

*d) Reaction order in* [**1a**]

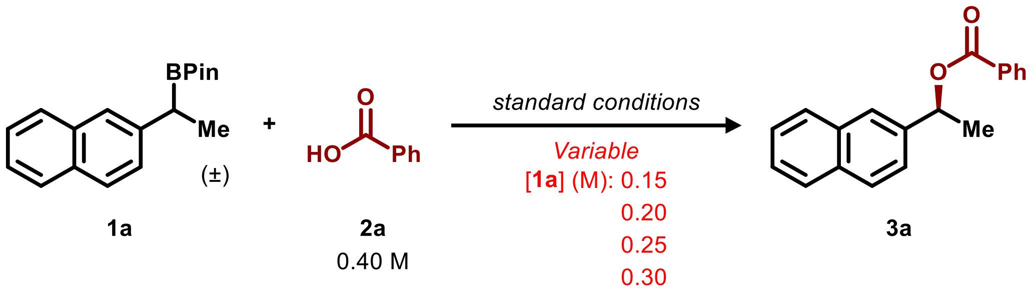


*Result*: Plotting product **3a** formation over time

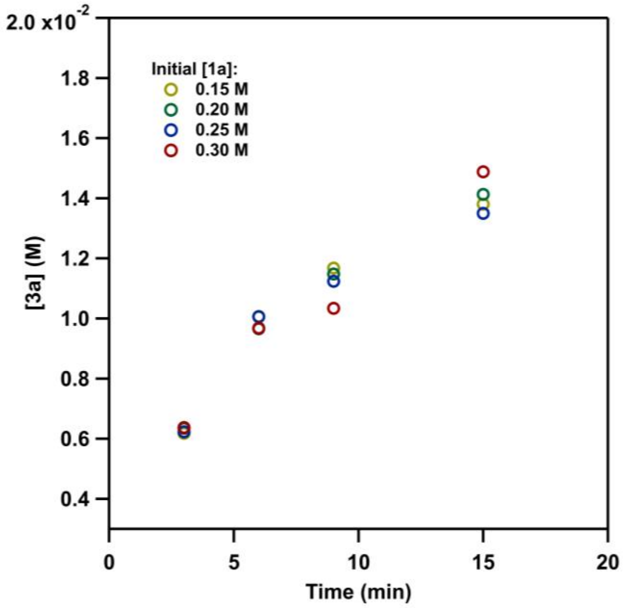


Visual inspection of the data indicates that initial [**1a**] does not significantly influence the initial rate of the transformation, suggesting the reaction is likely zeroth order in alkylborane **1a** concentration.

*e) Reaction order in* [**2a**]

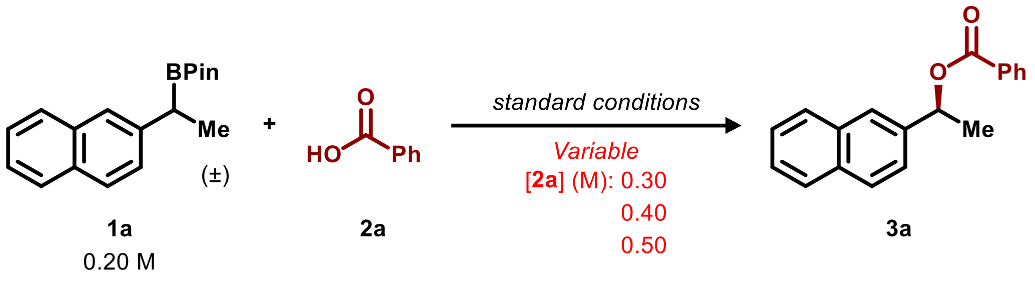


*Result*: Plotting product **3a** formation over time

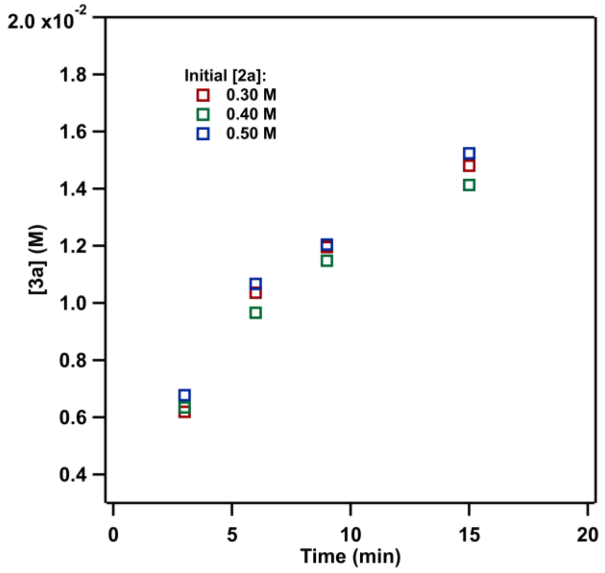


Visual inspection of the data indicates that initial [**2a**] does not significantly influence the initial rate of the transformation, suggesting the reaction is likely zeroth order in carboxylic acid **2a** concentration.

*f) Reaction order in* [Cu]

At catalyst loadings below 10 mol%, the initial-rate measurements showed significant variability, likely due to partial catalyst deactivation. Obtaining reproducible kinetic data therefore required higher Cu concentrations than those used in the standard protocol (> 10 mol%). However, further increasing the catalyst loading was complicated by the limited solubility of the active ionic Cu species, whether generated *in situ* from CuCl/**L6**/$NaBAr^F_4$ or introduced as independently prepared complex. As a result, sufficiently concentrated and homogeneous solutions could not be achieved, preventing a reliable kinetic analysis of the reaction order in [Cu] despite repeated attempts.

### 3) Light On-Off Experiment

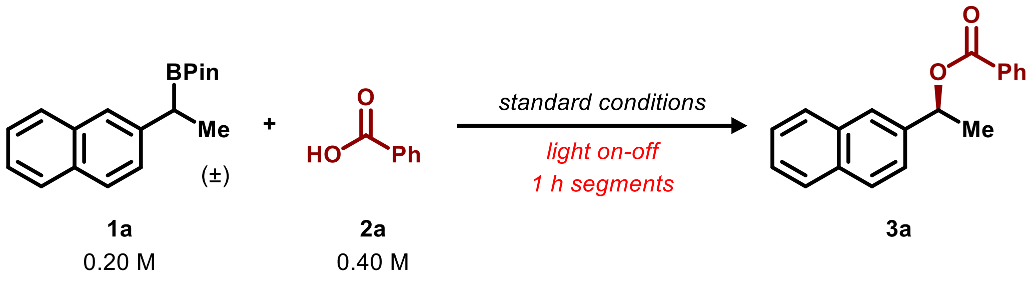


Inside a nitrogen-filled glovebox, an oven-dried reaction tube (Fisherbrand, 16 × 125 mm, catalog no. 1495935A) equipped with a magnetic stir bar was charged with CuCl (5.0 mg, 0.050 mmol, 10 mol%), $NaBAr^F_4$ (66.5 mg, 0.075 mmol, 15 mol%), and **L6** (73.5 mg, 0.075 mmol, 15 mol%) followed by the addition of a 1:1 mixture of PhCl and PhMe (2.5 mL). The reaction mixture was stirred at room temperature for 10 min before the addition of **4e** (14.0 mg ,0.050 mmol, 10 mol%), alkylborane **1a** (141 mg, 0.50 mmol, 1.0 equiv), and carboxylic acid **2a** (122 mg, 1.00 mmol, 2.0 equiv). The reaction mixture was stirred for 10 min before di-*tert*-butyl peroxide (183 µL, 1.00 mmol, 2.0 equiv) was added in one portion. 1,3,5-trimethoxybenzene (84.1 mg, 0.50 mmol) was added to the reaction as an internal standard. The reaction tube was sealed with a screw cap (Kimble Chase Open Top S/T Closure, catalog no. 73804-15425) containing a PTFE septum (Thermo Scientific, catalog no. B7995-15), taken outside of the glovebox, placed in a –20 °C freezer and irradiated with 427 nm light (Kessil P160L) with stirring.

After the reaction mixture had stirred under irradiation for 1 h, a 50 µL aliquot was taken using a 100 µL glass microsyringe by piercing through the septum. The reaction was then kept stirring in the dark at –20 °C for 1 h before another 50 µL aliquot was taken. The procedure was repeated three times to monitor the reaction for a total of 6 h

Solvent was removed from these aliquots *in vacuo*, and the residue was analyzed using $^1H$ NMR spectroscopy with 1,3,5-trimethoxybenzene as the internal standard. Yield of product **3a** (%) was plotted over time.

*Result*:

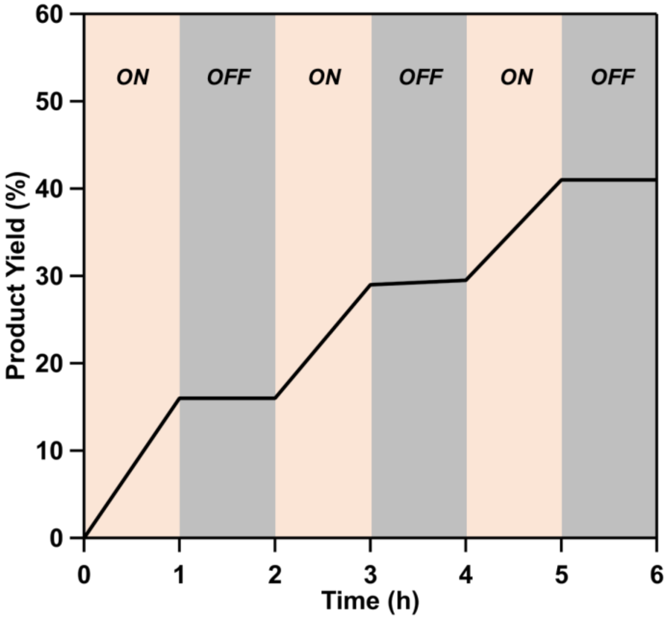


The result indicates that no product formation was observed in the absence of light irradiation, suggesting (1) the lack of a radical-chain mechanism and (2) the necessity of photochemical activation of $(^{t}BuO)_2$.

## 4) Stoichiometric Mechanistic Study

*a) Aminyl radical dimerization*

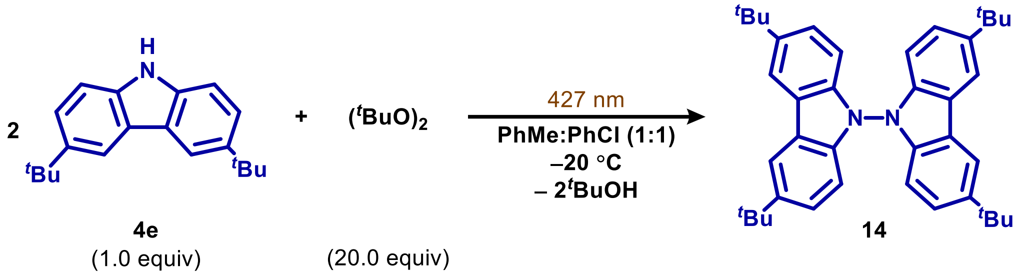


Inside a nitrogen-filled glovebox, an oven-dried reaction tube (Fisherbrand, 16 × 125 mm, catalog no. 1495935A) equipped with a magnetic stir bar was charged with **4e** (14 mg, 0.050 mmol, 1.0 equiv) and a 1:1 mixture of PhCl and PhMe (2.5 mL). The mixture was stirred for 10 min before di-*tert*-butyl peroxide (183 µL, 1.00 mmol, 20.0 equiv) was added in one portion. The reaction tube was sealed with a screw cap (Kimble Chase Open Top S/T Closure, catalog no. 73804-15425) containing a PTFE septum (Thermo Scientific, catalog no. B7995-15), taken outside of the glovebox, placed in a –20 °C freezer and irradiated with 427 nm light (Kessil P160L) with stirring.

After the reaction mixture had stirred under irradiation for 16 h, the screw cap was removed from the reaction tube, and the reaction mixture was concentrated *in vacuo* with the aid of a rotary evaporator. The resulting crude material was analyzed with $^1$H NMR spectroscopy using 1,1,2,2-tetrachloroethane as the internal standard, revealing quantitative formation of the carbazole dimer **14**. A small portion of the reaction mixture was purified by reverse-phase (C18) column chromatography to verify the product identity.

*Note*: The ratio of **4e** to ($^t$BuO)$_2$ (1:20) aims to reproduce the catalytic conditions, where 10 mol% of **4e** was used in combination with 2.0 equiv of ($^t$BuO)$_2$.

*Characterization data*:

3,3',6,6'-tetra-tert-butyl-9,9'-bicarbazole (**14**)

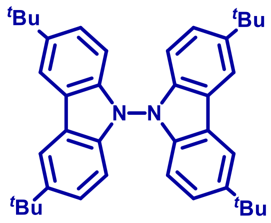

**$^{1}$H NMR** (400 MHz; $CDCl_3$) δ 8.08 (d, $J$ = 2.5 Hz, 2H), 7.24 (dd, $J$ = 8.5, 2.5 Hz, 2H), 6.70 (d, $J$ = 8.5 Hz, 2H), 1.35 (s, 18H).

**$^{13}$C NMR** (101 MHz; $CDCl_3$) δ 143.06, 137.61, 123.22, 120.98, 115.67, 107.75, 33.96, 31.16.

Spectroscopic data is consistent with reported values.[16]

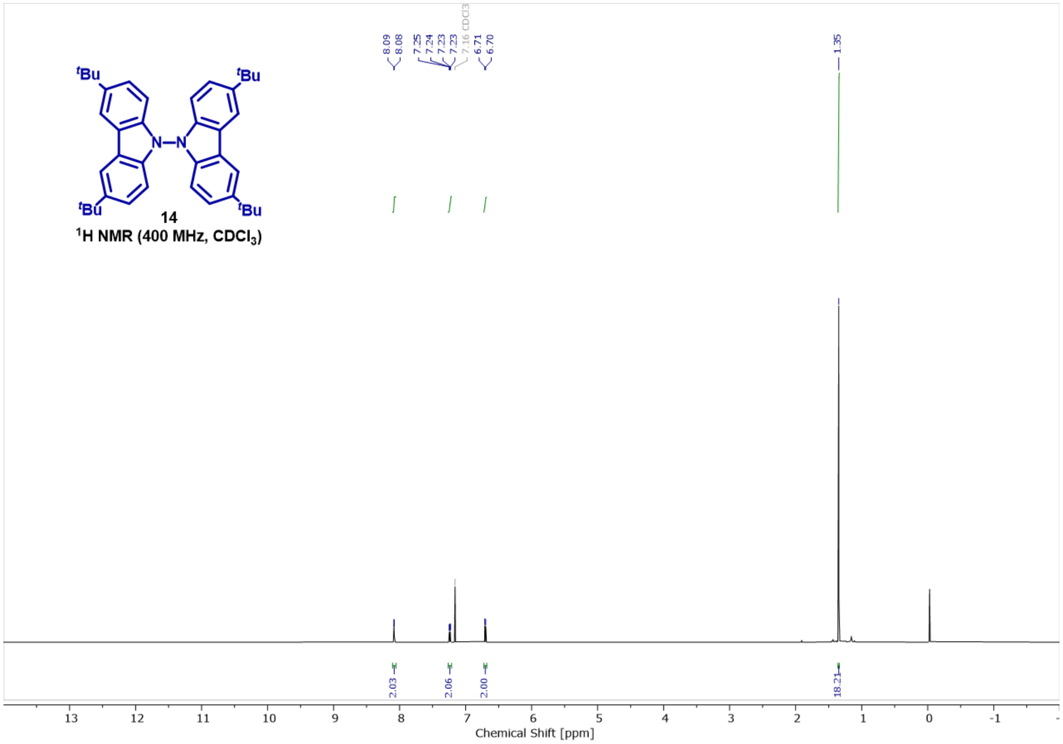


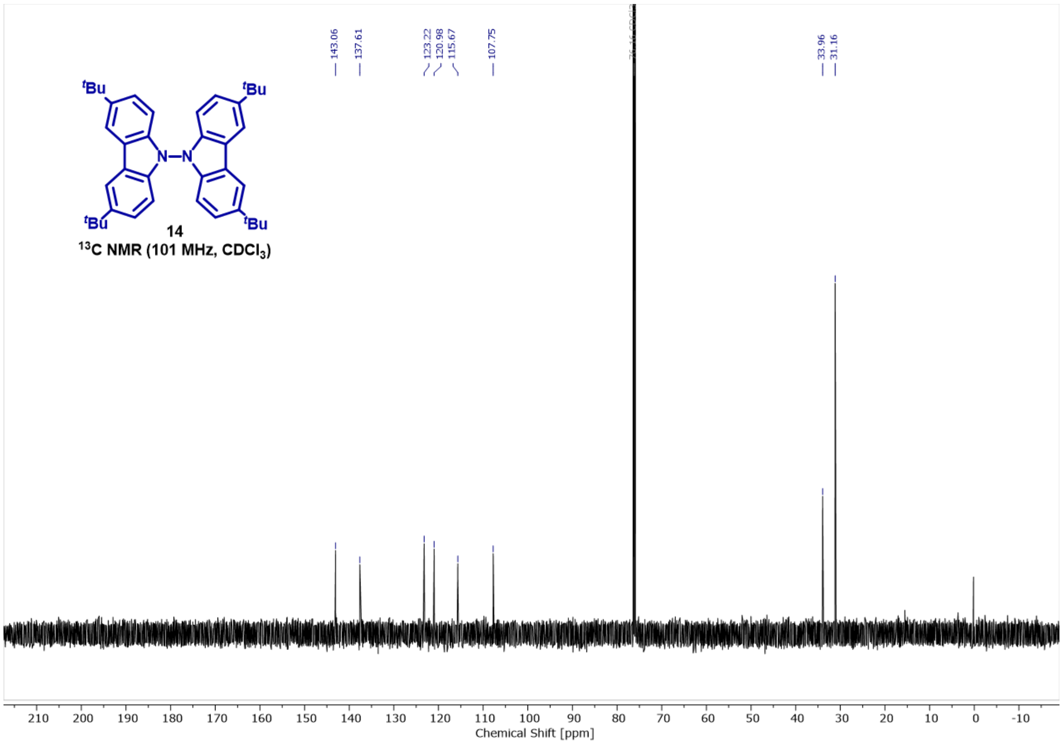

*b) Alkyl radical dimerization*

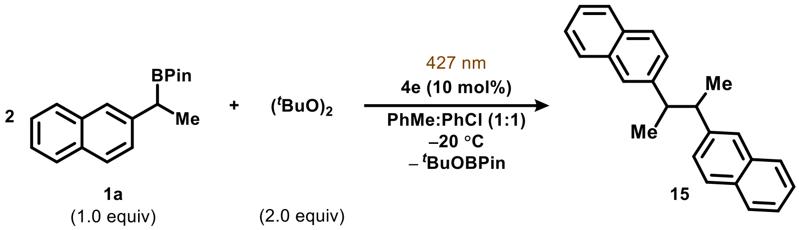


Inside a nitrogen-filled glovebox, an oven-dried reaction tube (Fisherbrand, 16 × 125 mm, catalog no. 1495935A) equipped with a magnetic stir bar was charged with **1a** (141 mg, 0.50 mmol, 1.0 equiv), **4e** (14 mg, 0.050 mmol, 10 mol%) and a 1:1 mixture of PhCl and PhMe (2.5 mL). The mixture was stirred for 10 min before di-*tert*-butyl peroxide (183 µL, 1.00 mmol, 2.0 equiv) was added in one portion. The reaction tube was sealed with a screw cap (Kimble Chase Open Top S/T Closure, catalog no. 73804-15425) containing a PTFE septum (Thermo Scientific, catalog no. B7995-15), taken outside of the glovebox, placed in a –20 °C freezer and irradiated with 427 nm light (Kessil P160L) with stirring.

After the reaction mixture had stirred under irradiation for 16 h, the screw cap was removed from the reaction tube, and the reaction mixture was concentrated *in vacuo* with the aid of a rotary evaporator. The resulting crude material was analyzed with $^1$H NMR spectroscopy using 1,1,2,2-tetrachloroethane as the internal standard, revealing the formation of alkyl dimer **15** (36% yield). A small portion of the reaction mixture was purified by silica thin-layer column chromatography to verify the product identity.

*Note*: The same procedure was repeated in the absence of **4e**. No consumption of **1a** nor formation of **15** was observed.

*Characterization data*:

2,2'-(butane-2,3-diyl)dinaphthalene (**15**)

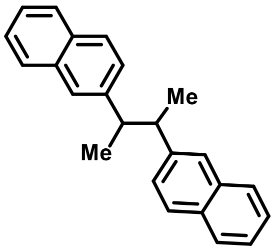


**$^1$H NMR** (400 MHz; $CDCl_3$) δ 7.77–7.71 (m, 3H), 7.66–7.54 (m, 4H), 7.42–7.25 (m, 6H), 7.17–7.12 (m, 1H), 3.25–3.11 (m, 1H), 3.06–2.94 (m, 1H), 1.30 (d, $J$ = 6.9 Hz, 3H), 1.04 (d, $J$ = 6.9 Hz, 3H).

**$^{13}$C NMR** (101 MHz; $CDCl_3$) δ 144.12, 143.46, 133.77, 133.48, 132.49, 132.19, 128.15, 127.78, 127.73, 127.61, 127.46, 126.90, 126.33, 126.22, 126.14, 126.05, 125.75, 125.35, 125.17, 47.44, 46.36, 21.42, 18.13.

**HRMS** (APGC) m/z: $[M]^+$ calcd for $C_{24}H_{22}^+$: 310.1716. Found: 310.1716.

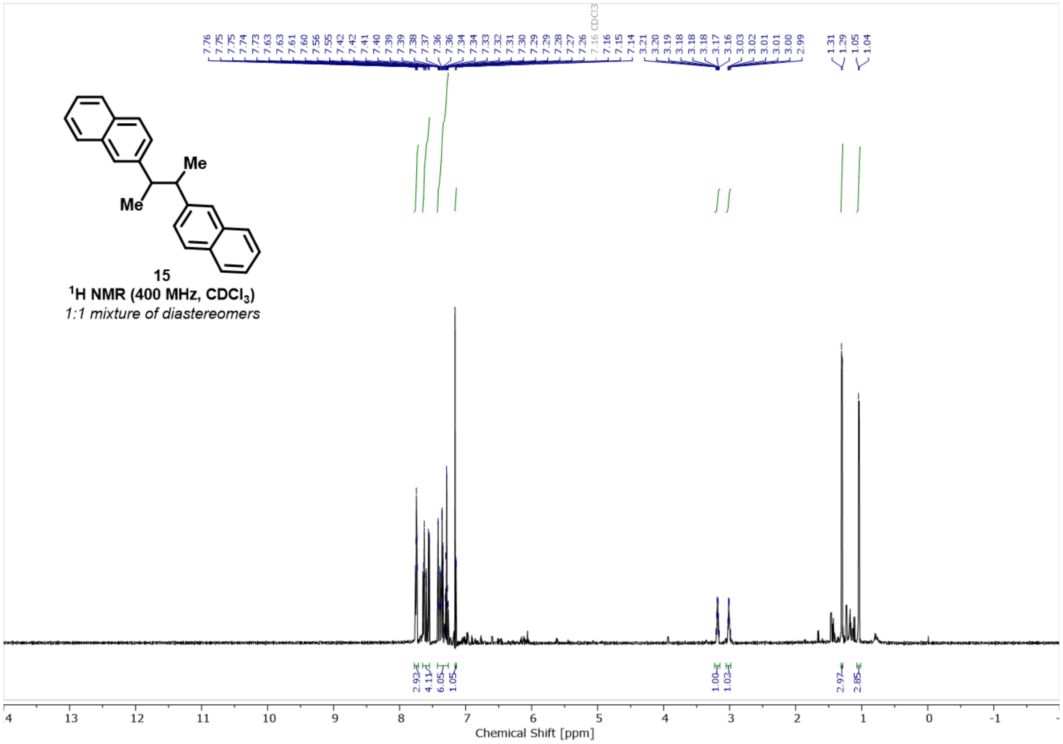


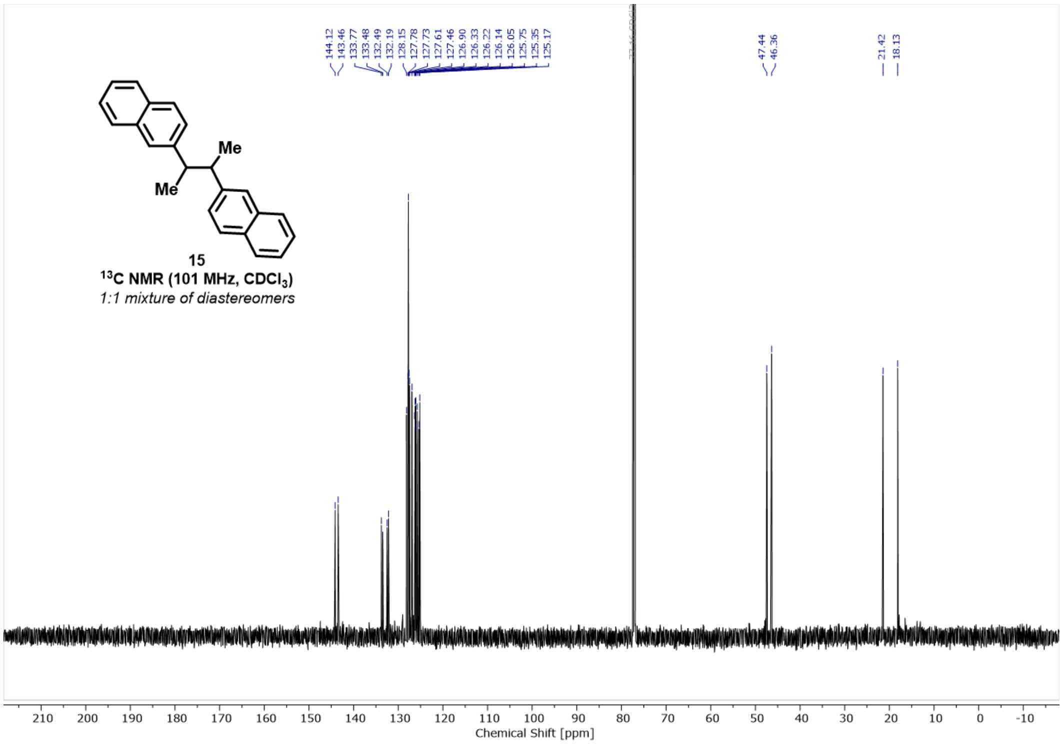

*c) Regeneration of **4e***

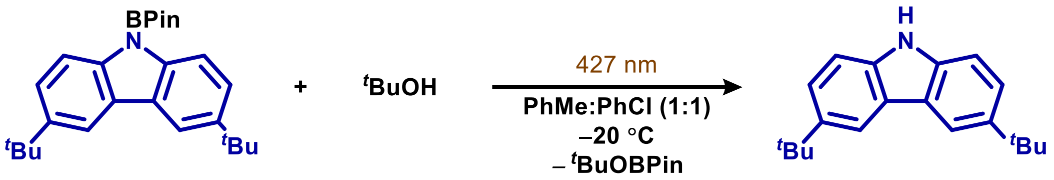


**12** (1.0 equiv) (1.0 equiv) **4e**

The carbazoleborane **12** was synthesized according to reported procedure.[17]

Inside a nitrogen-filled glovebox, an oven-dried reaction tube (Fisherbrand, 16 × 125 mm, catalog no. 1495935A) equipped with a magnetic stir bar was charged with **12** (20 mg, 0.050 mmol, 1.0 equiv), ${}^{t}$BuOH (5 µL, 0.050 mmol, 1.0 equiv) and a 1:1 mixture of PhCl and PhMe (2.5 mL). The reaction tube was sealed with a screw cap (Kimble Chase Open Top S/T Closure, catalog no. 73804-15425) containing a PTFE septum (Thermo Scientific, catalog no. B7995-15), taken outside of the glovebox, placed in a –20 °C freezer and irradiated with 427 nm light (Kessil P160L) with stirring.

After the reaction mixture had stirred under irradiation for 16 h, the screw cap was removed from the reaction tube, and the reaction mixture was concentrated *in vacuo* with the aid of a rotary evaporator. The resulting crude material was analyzed with ${}^{1}$H NMR spectroscopy using 1,1,2,2-tetrachloroethane as the internal standard, revealing the formation of **4e** (97%).

*Result*:

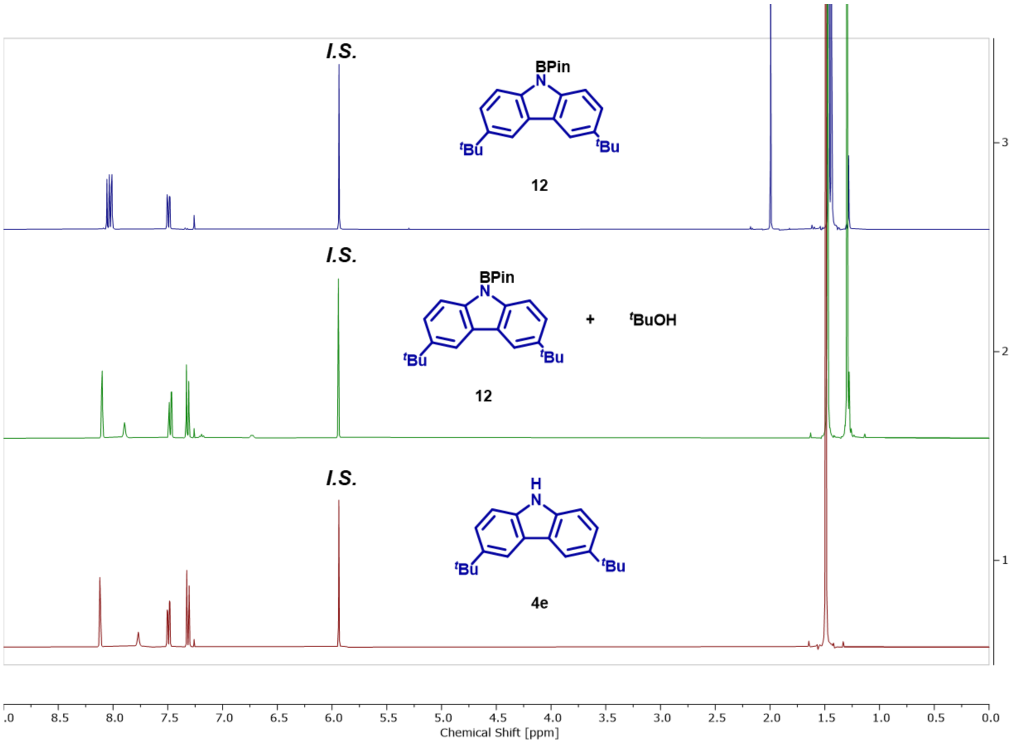


The result suggests that upon reacting carbazoleborane **12** (top) with stoichiometric *ᵗ*BuOH under standard catalytic condition (middle), it is quantitatively converted into carbazole **4e**. $^{1}$H NMR spectrum of **4e** with the same amount of internal standard is shown (bottom) as reference.

## 4) Independent Preparation of Cu Intermediates

*Note*: The synthesis of putative Cu intermediates was performed using the catalytically competent ligand **L1**, as it provides higher crystallinity than **L6**. In contrast, reactions with **L6** frequently yield oily residues highly soluble in nonpolar solvents (e.g., pentane or tetramethylsilane), which precluded structural characterization by single-crystal X-ray diffraction.

*Note*: All syntheses were conducted in the catalytically competent solvent DCE because (1) its lower boiling point relative to PhMe and PhCl facilitates workup, and (2) CuCl and $NaBAr^F_4$ dissolve more rapidly in DCE, expediting our synthetic efforts.

*Synthesis of [(**L1**)Cu](BAr$^F_4$) [(**16**$^+$)(BAr$^F_4$$^-$)]*

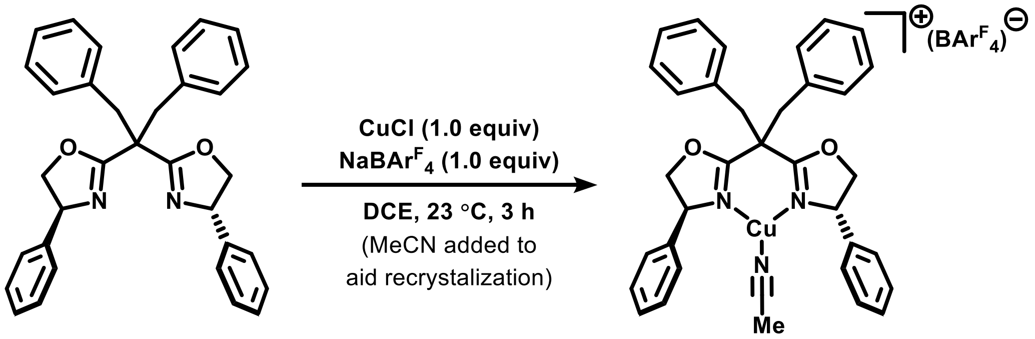


Inside a nitrogen-filled glovebox, a 20 mL scintillation vial equipped with a magnetic stir bar was charged with CuCl (5 mg, 0.05 mmol, 1.0 equiv) and **L1** (24 mg, 0.05 mmol, 1.0 equiv), followed by 2.5 mL of DCE. The mixture was stirred at 25 °C for 10 min before the addition of $NaBAr^F_4$ (44 mg, 0.05 mmol, 1.0 equiv). The mixture was then stirred at 25 °C for 3 h to ensure the complete dissolution of $NaBAr^F_4$. All volatiles were removed *in vacuo*, and 1.0 mL of DCM was added to the residue, resulting in a suspension. The mixture was filtered, and all volatiles were removed *in vacuo*. The residue was dissolved in 1.0 mL of DCM resulting in a clear, colorless solution. One drop of MeCN was added to the solution before 3.0 mL of pentane was layered onto this solution for slow diffusion. The mixture was then stored in a –30 °C freezer for recrystallization. Colorless crystals suitable for X-ray diffraction was obtained (35 mg, 48%) following the complete diffusion of pentane. Structural characterization revealed the product as the ionic complex [(**L1**)Cu](BAr$_4$) ((**16**$^+$)(BAr$^F_4$$^-$), Figure S5).

*Synthesis of [(**L1**)Cu(OBz)](PF$_6$) [(**18**$^+$)(PF$_6^-$)]*

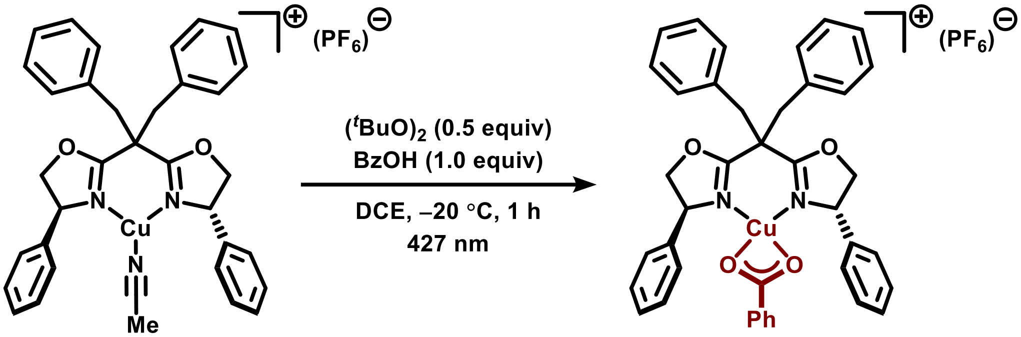


*Note*: Attempts to synthesize (**18**$^+$)(BAr$^F{}_4{}^-$) was complicated by the slow dissolution of (**16**$^+$)(BAr$^F{}_4{}^-$) in DCE (incomplete dissolution after 16 h). To probe the oxidation of cation **16**$^+$, we instead prepared (**16**$^+$)(PF$_6^-$) (complete dissolution in DCE after 30 min) according to reported procedure.[2]

Inside a nitrogen-filled glovebox, an oven-dried reaction tube (Fisherbrand, 16 × 125 mm, catalog no. 1495935A) equipped with magnetic stir bars were charged with (**16**$^+$)(PF$_6^-$) (37 mg, 0.05 mmol) and 2.5 mL DCE. The mixture was stirred for 30 min before the addition of ($^t$BuO)$_2$ (5 μL, 0.025 mmol, 0.5 equiv) and BzOH (6 mg, 0.05 mmol). The reaction mixture was sealed with a screw cap (Fisherbrand, catalog no. 1495936A), taken outside of the glovebox, and placed in a –20 °C freezer with stirring and irradiated with 427 nm light.

After the mixture had stirred for 1 h at –20 °C, the reaction tube was brought inside the glovebox. All volatiles were removed from the deep blue reaction mixture *in vacuo*, and 1.0 ml of DCM was added to the mixture. The mixture was filtered and the 3.0 mL of pentane was layered to the filtrate for slow diffusion. The mixture was then stored in a –30 °C freezer for recrystallization. Blue crystals suitable for X-ray diffraction was obtained (23 mg, 56%) following the complete diffusion of pentane. Structural characterization revealed the product as the ionic complex [(**L1**)Cu(OBz)](PF$_6$) [(**18**$^+$)(PF$_6^-$)].

*Note*: Formation of (**18**$^+$)(PF$_6^-$) at 25 °C in the absence of photochemical conditions proved sluggish. Full conversion of starting material was observed after 6 h. The oxidation was inhibited at –20 °C in the absence of photochemical conditions. No conversion of the starting material was observed after 16 h.

## 5) Quantitative EPR Study

*EPR characterization of ($18^+$)($PF_6^-$)*

The EPR feature of (**18**$^+$)($PF_6^-$) was measured using a solution of (**18**$^+$)($PF_6^-$) (33 mg, 0.04 mmol) dissolved in 2.0 mL of DCM ([Cu] = 0.020 M) at 77K.

*EPR characterization of reaction mixture*

Similarly, a standard reaction mixture for coupling **1a** and **2a** at 0.50 mmol scale was analyzed using EPR spectroscopy (at 10 mol% catalyst loading, [Cu] = 0.020 M). After the reaction had stirred under standard condition for 2 h, the reaction tube was transferred into a glovebox and a 0.5 mL aliquot was transferred to an EPR tube for analysis at 77K.

*Result*

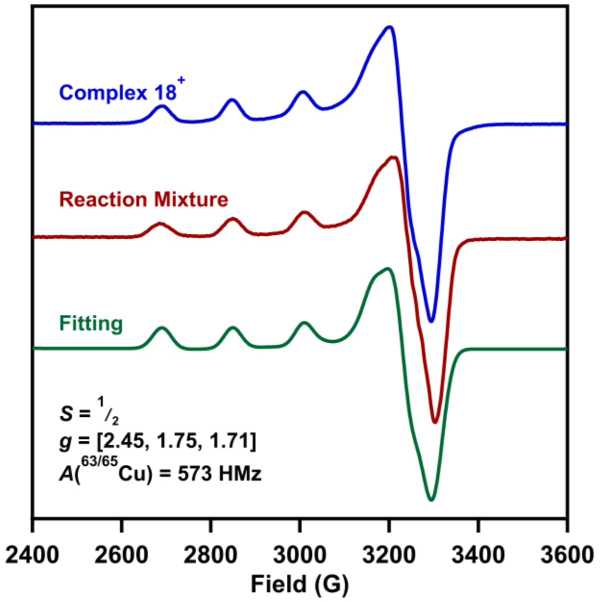


The EPR feature observed from reaction mixture is consistent with that observed using independently prepared **18**$^+$.

*Quantitative analysis*

To quantify the EPR features observed using the reaction mixture, a calibration curve was constructed using **18**$^+$.

The EPR spectra of (**18**$^+$)($PF_6^-$) in DCM with 0.016, 0.024, 0.032, 0.040 M concentrations were acquired. Plotting the double integration of the resulting features against [Cu] yielded a calibration curve. Same data processing procedure was applied to the EPR feature obtained using reaction mixture, and the result was plotted in the figure.

*Calibration Curve*

| Concentration (M) | Double Integration |
|---|---|
| 0.016 | 30463.76211 |
| 0.024 | 44637.57276 |
| 0.032 | 60229.24844 |
| 0.040 | 78009.83307 |

*Reaction Mixture*

| Concentration (M) | Double Integration |
|---|---|
| 0.020 | 33118.76517 |

*Note*: EPR setup: Freq: 9.4 GHz; Temp: 77K; Power: 0.6325 mW; PowerAtten: 25.0 dB; Gain: 60 dB; ModAmp: 4.0 G; ModFreq: 100.00 kHz; Sweep Time: 60 s; ConvTime: 58.59 ms;  results are from 16 scans averaged)

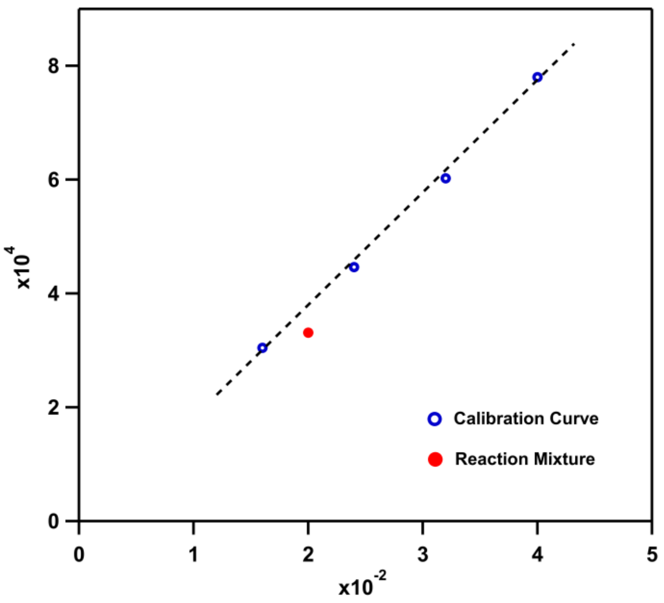


The result suggests that **18**$^{+}$ accounts for 88% of all Cu present in the reaction mixture, suggesting that **18**$^{+}$ is likely the Cu catalyst resting state.

**6) Stoichiometric C(sp$^3$)–O Bond Formation**

Gomberg's dimer was synthesized on a 5.00 mmol scale according to literature procedure.[18]

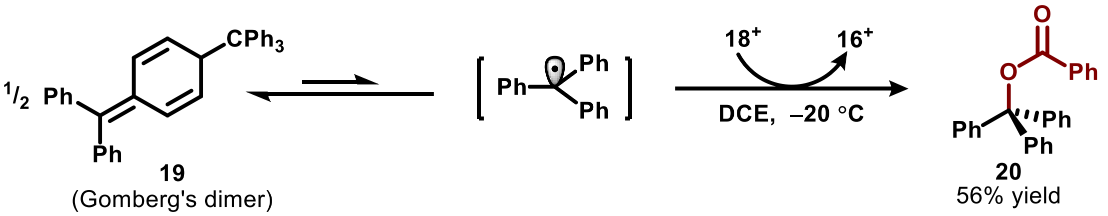


Inside a nitrogen-filled glovebox, an oven-dried reaction tube (Fisherbrand, 16 × 125 mm, catalog no. 1495935A) equipped with a magnetic stir bar was charged with Gomberg's dimer **19** (12 mg, 0.025 mmol, 0.5 equiv) and Cu(II) carboxylate **18**$^+$($PF_6$)$^-$ (41 mg, 0.05 mmol, 1.0 equiv) followed by the addition of 2.5 mL DCE precooled to −30 °C. The reaction tube was sealed with a screw cap (Kimble Chase Open Top S/T Closure, catalog no. 73804-15425) containing a PTFE septum (Thermo Scientific, catalog no. B7995-15), taken outside of the glovebox, placed in a −20 °C freezer. The reaction mixture was stirred for 2 h, during which the solution changed from deep green to light yellow, likely due to the consumption of **18**$^+$. The screw cap was removed from the reaction tube, and the reaction mixture was concentrated *in vacuo* with the aid of a rotary evaporator. The residue was purified by reverse-phase column chromatography on C18 stationary phase (40 g cartridge, 20% to 100% MeCN in $H_2O$) to yield the C(sp$^3$)–O coupled product **20** (10 mg, 56%) as a white solid. Formation of **20** demonstrates the radical trapping ability of electrophilic Cu(II) carboxylate **18**$^+$.

*Characterization data*:

trityl benzoate (**20**)

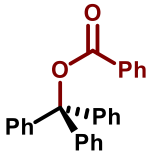


**$^1$H NMR** (400 MHz; $CDCl_3$) δ 8.13 (d, $J$ = 7.0 Hz, 2H), 7.58 (t, $J$ = 7.4 Hz, 1H), 7.50–7.43 (m, 8H), 7.36–7.28 (m, 7H), 7.28–7.22 (m, 2H).

**$^{13}$C NMR** (101 MHz; $CDCl_3$) δ 164.58, 143.54, 133.14, 131.42, 129.98, 128.58, 128.52, 127.97, 127.48, 90.65.

Spectroscopic data is consistent with reported values.[19]

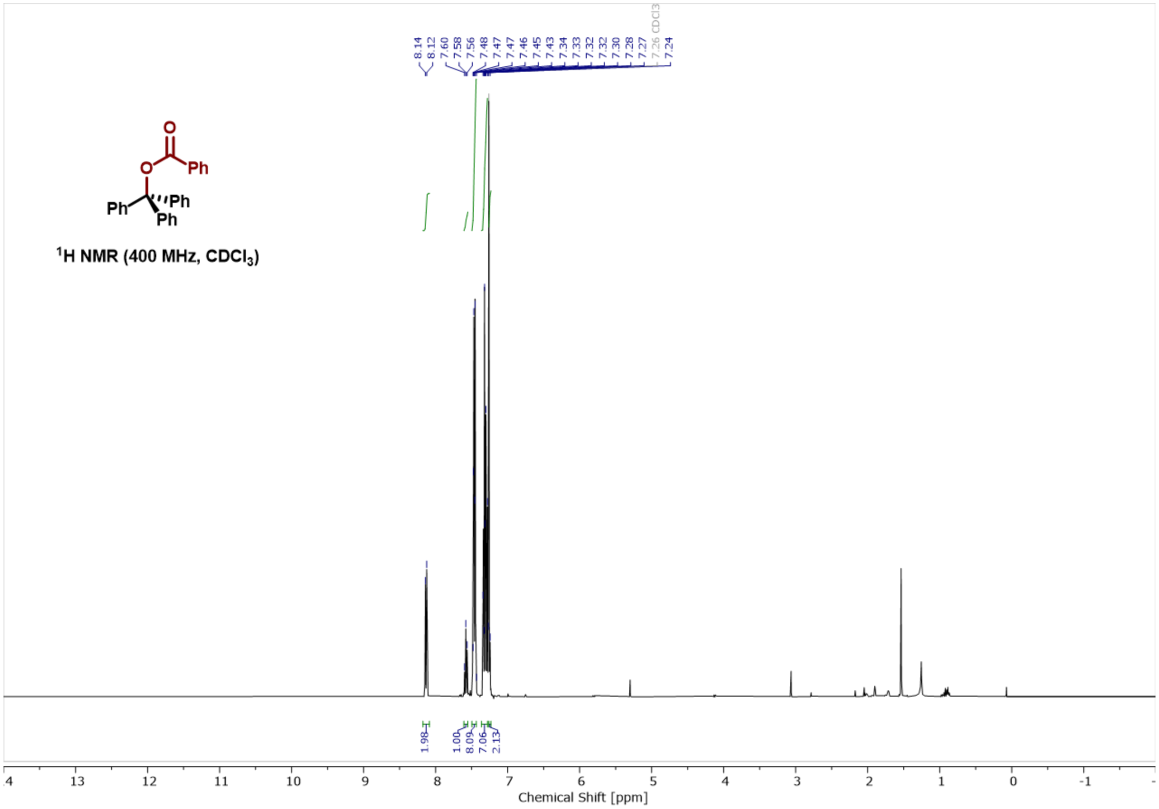

O
Ph
O
Ph
Ph
Ph
¹H NMR (400 MHz, CDCl3)
8.14
8.12
7.60
7.58
7.56
7.48
7.47
7.47
7.46
7.45
7.43
7.34
7.33
7.33
7.32
7.32
7.30
7.28
7.27
7.26 CDCl3
7.24
1.98
1.00
8.09
7.06
2.13
14
13
12
11
10
9
8
7
6
5
4
3
2
1
0
-1
Chemical Shift [ppm]


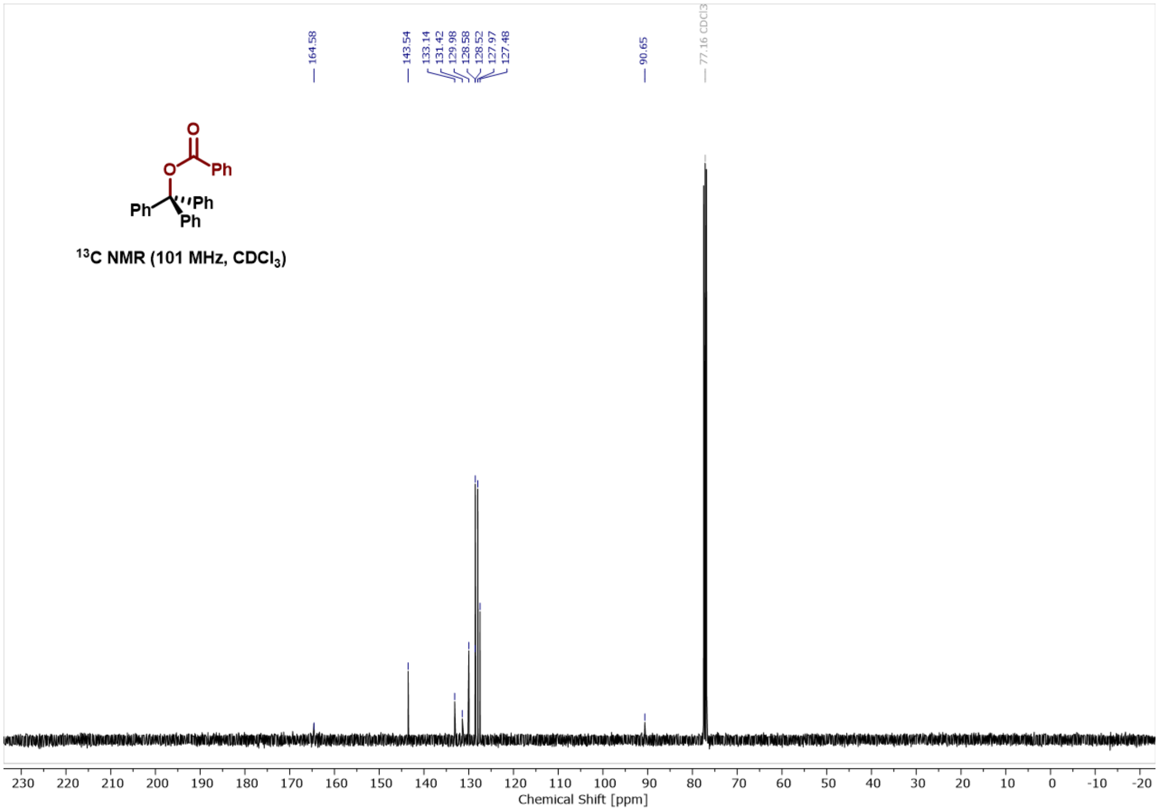

O
Ph
O
Ph
Ph
Ph
¹³C NMR (101 MHz, CDCl3)
164.58
143.54
133.14
131.42
129.98
128.58
128.52
127.97
127.48
90.65
77.16 CDCl3
230
220
210
200
190
180
170
160
150
140
130
120
110
100
90
80
70
60
50
40
30
20
10
0
-10
-20
Chemical Shift [ppm]

To elucidate the substituent effect on radical trapping reactivity of Cu(II) benzoate complexes, we subsequently prepared the *para*-substituted benzoate analogues $\mathbf{18_{CF3}}^+$ and $\mathbf{18_{Me}}^+$ and compared their radical-trapping reactivities with that of $\mathbf{18}^+$ using a binary competition protocol.

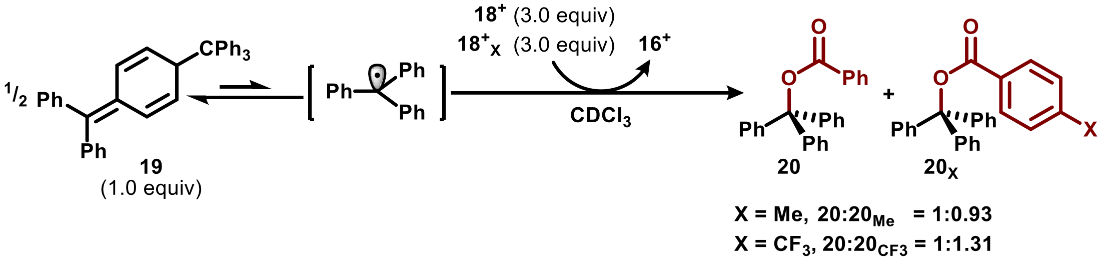


Inside a nitrogen-filled glovebox, an oven-dried NMR tube was charged with $\mathbf{18_X}^+(PF_6)^-$ (0.01 mmol, 3.0 equiv) and $\mathbf{18}^+(PF_6)^-$ (8.2 mg, 0.01 mmol, 3.0 equiv) followed by the addition of 0.5 mL $CDCl_3$. A solution of Gomberg's dimer **19** (1.6 mg, 0.003 mmol, 0.5 equiv; 1.0 equiv of trityl radical) dissolved in 0.1 mL $CDCl_3$ was added to the NMR tube. The mixture was allowed to react for 5 min, and NMR spectra were acquired for analysis.

With $\mathbf{18_{Me}}^+(PF_6)^-$, the corresponding product ratio $\mathbf{20}/\mathbf{20_{Me}}$ = 1:0.93

With $\mathbf{18_{CF3}}^+(PF_6)^-$, the corresponding product ratio $\mathbf{20}/\mathbf{20_{CF3}}$ = 1:1.31

Product $\mathbf{20_{Me}}$ and $\mathbf{20_{CF3}}$ were synthesized independently to aid our analysis.

*Discussion*: We found that trityl-radical trapping is slightly more efficient for the more electron-deficient benzoate complex $\mathbf{18_{CF3}}^+$, whereas the electron-donating Me-substituted analogue $\mathbf{18_{Me}}^+$ exhibits slightly slower reactivity. While this trend is consistent with the Hammett analysis (Figure 3E), the magnitude of this effect appears insufficient by itself to explain the overall rate differences observed in the Hammett study. We therefore reason that the overall rate trend arises from the combined influence of radical capture and transmetalation, with computation indicating that the transmetalation step has a slightly higher barrier than radical trapping and therefore likely contributes more significantly to the overall rate difference.

*Note*: Because trityl radical is electronically distinct from the benzylic radicals involved in catalysis and is more readily oxidized, these competition data may not fully capture the radical-trapping behavior operative under the catalytic conditions.

*Characterization data*:

trityl 4-methylbenzoate (**20**$_{\mathbf{Me}}$)

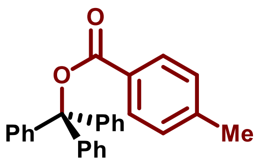

$^{1}$H NMR (400 MHz; CDCl$_3$) δ 7.94 (d, *J* = 7.8 Hz, 2H), 7.38 (d, *J* = 7.7 Hz, 6H), 7.28–7.14 (m, 11H), 2.34 (s, 3H).

$^{13}$C NMR (101 MHz; CDCl$_3$) δ 164.66, 143.67, 130.02, 129.28, 128.53, 128.08, 128.07, 127.94, 127.42, 90.41, 21.82.

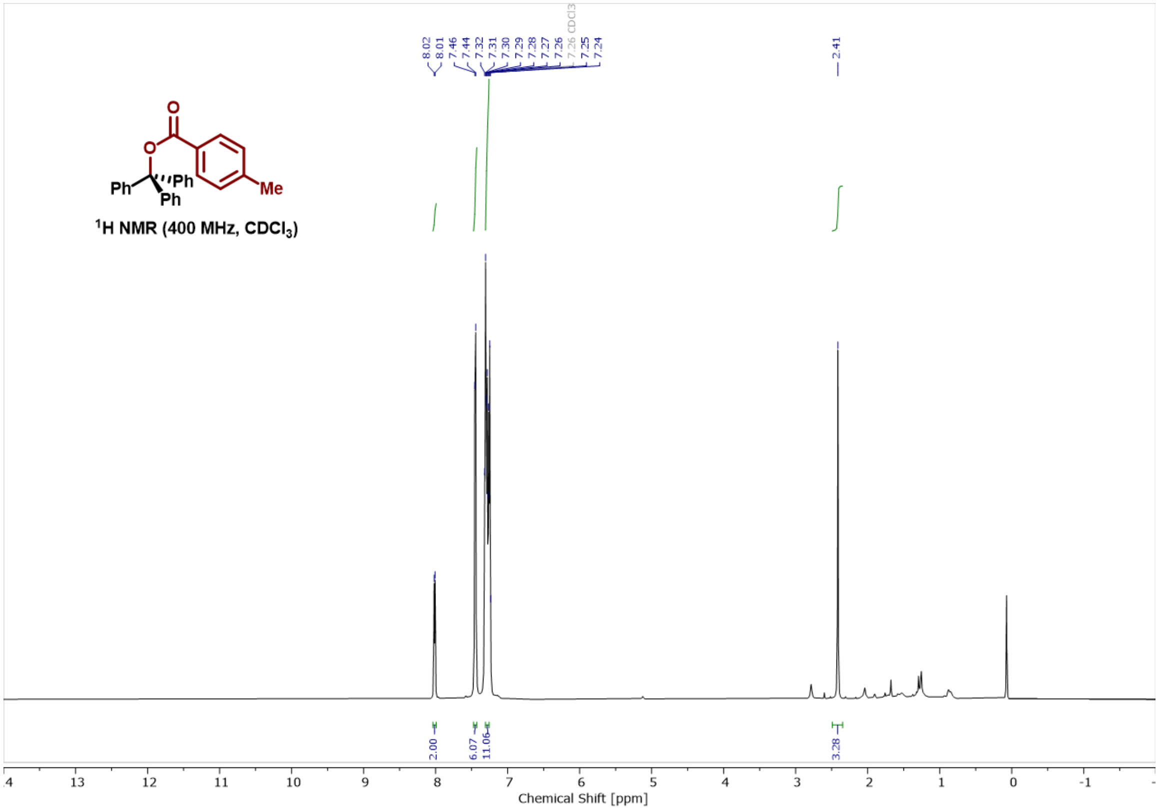

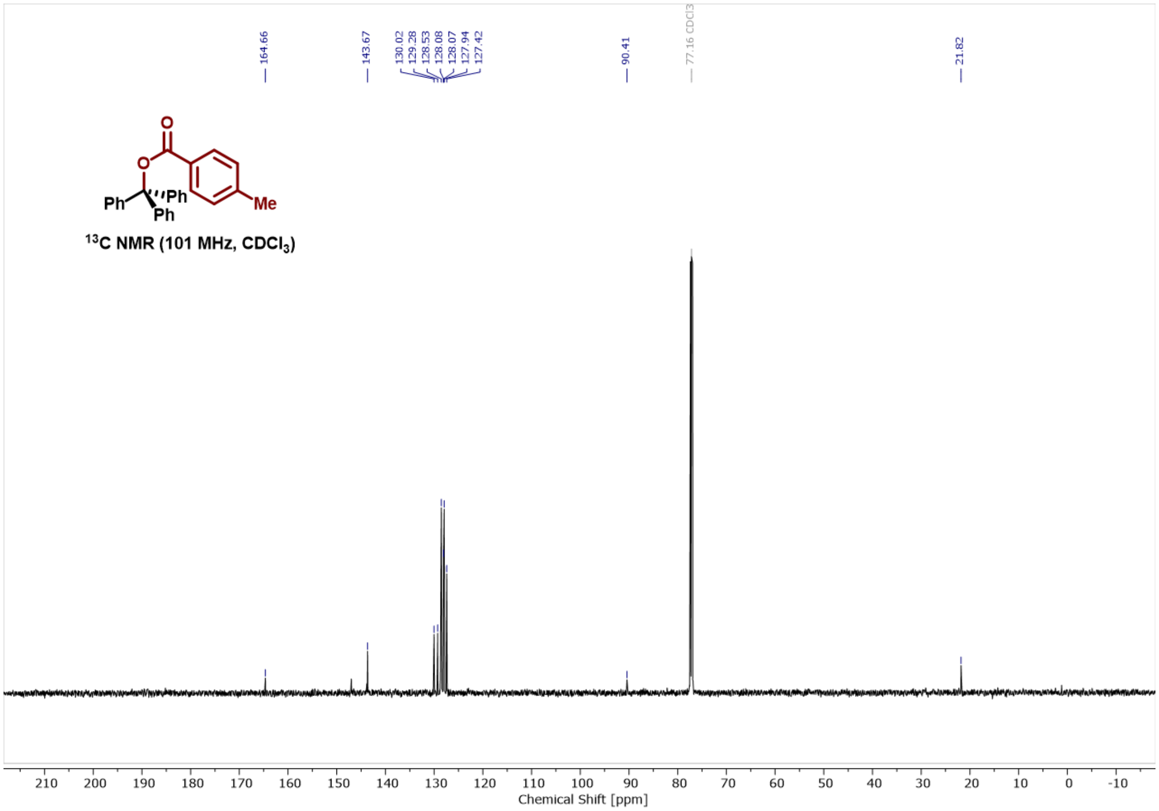


trityl 4-(trifluoromethyl)benzoate (**20**$_{CF3}$)

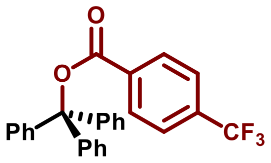


$^{1}$H NMR (400 MHz; $CDCl_3$) δ 8.24 (d, *J* = 8.0 Hz, 2H), 7.74 (d, *J* = 8.0 Hz, 2H), 7.45 (d, *J* = 7.8 Hz, 6H), 7.39–7.27 (m, 9H).

$^{13}$C NMR (101 MHz; $CDCl_3$) δ 163.45, 143.15, 134.69 (d, *J* = 19.1 Hz), 130.35, 128.49, 128.07, 127.69, 127.41, 125.66 (q, *J* = 3.8 Hz), 123.79 (q, *J* = 273.0 Hz), 91.52.

$^{19}$F NMR (376 MHz; $CDCl_3$) δ –63.08.

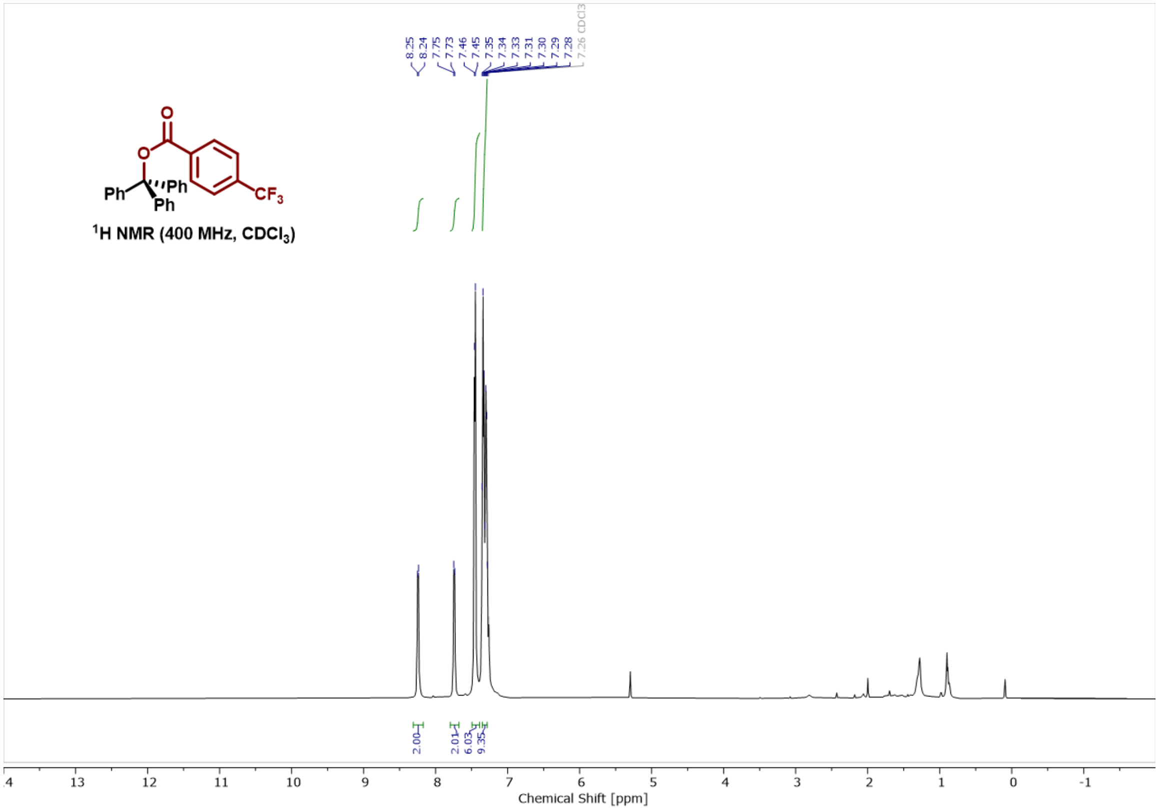
8.25
8.24
7.75
7.73
7.46
7.45
7.35
7.34
7.33
7.31
7.30
7.29
7.28
7.26 CDCl3
O
O
Ph
Ph
Ph
CF3
1H NMR (400 MHz, CDCl3)
2.00
2.01
6.03
9.35
Chemical Shift [ppm]

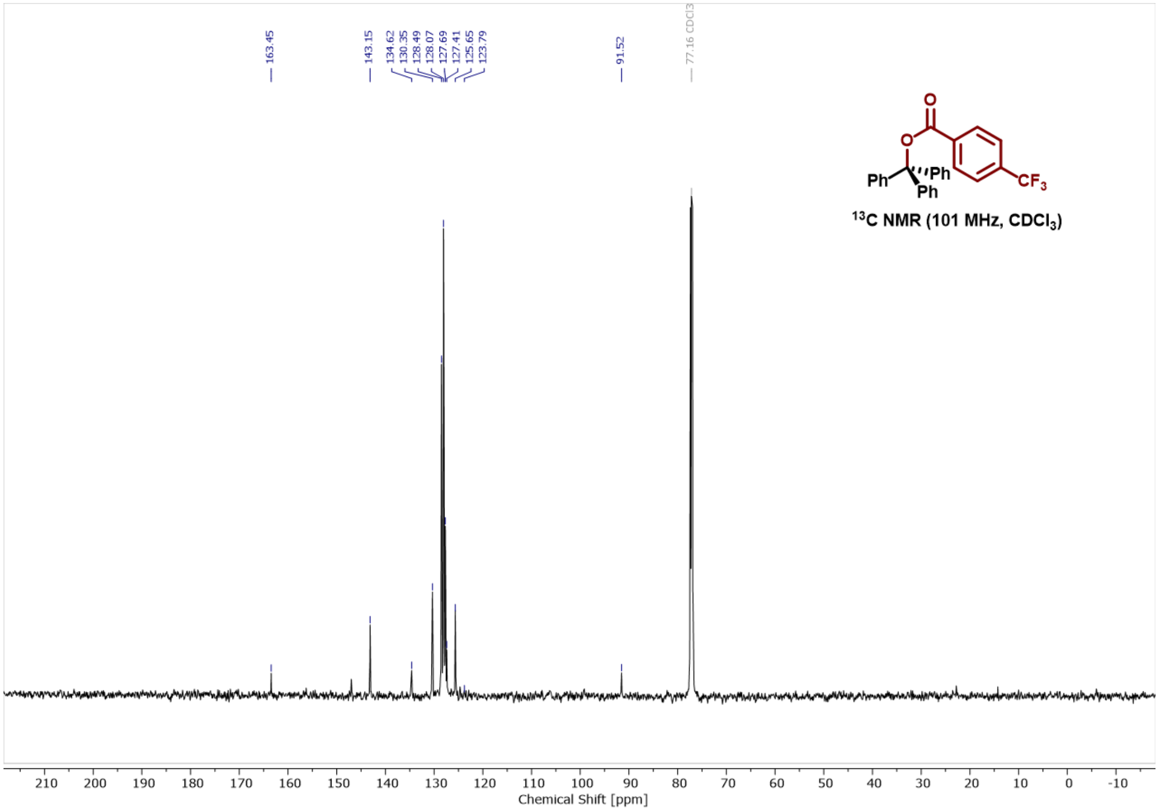
163.45
143.15
134.62
130.35
128.49
128.07
127.69
127.41
125.65
123.79
91.52
77.16 CDCl3
O
O
Ph
Ph
Ph
CF3
13C NMR (101 MHz, CDCl3)
Chemical Shift [ppm]

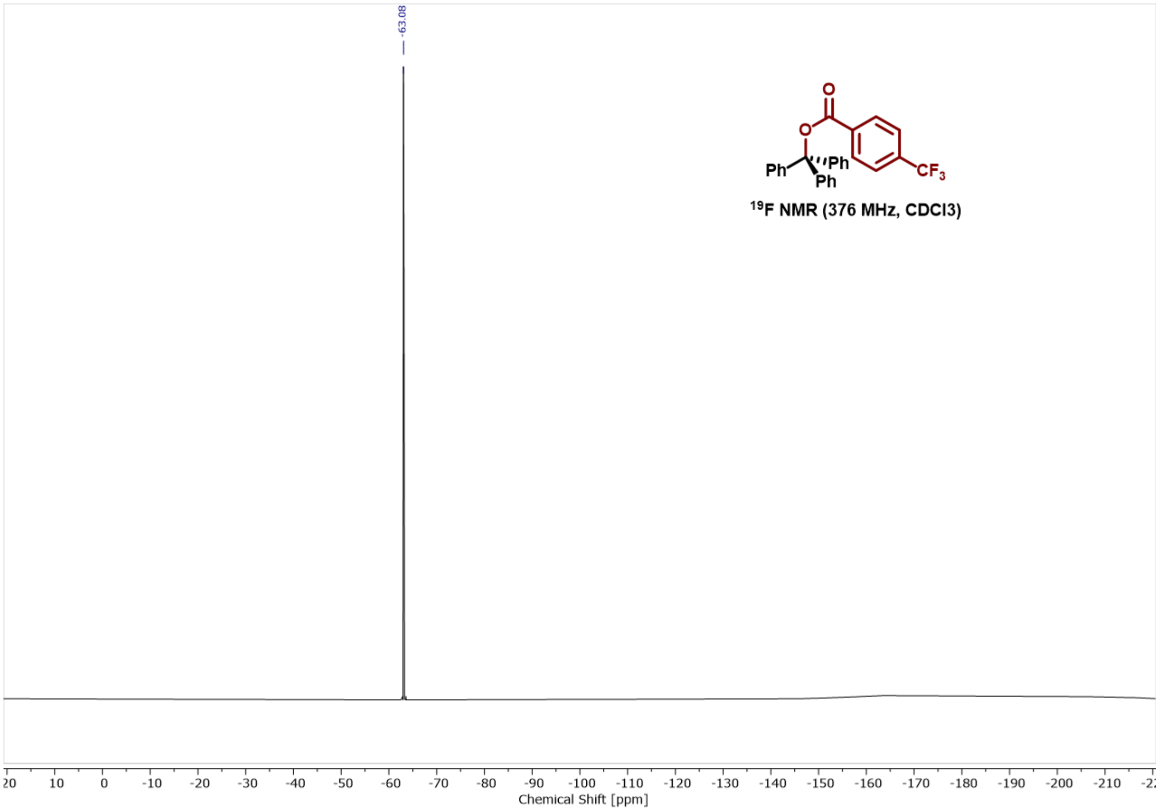
-63.08
O
O
Ph
Ph
Ph
CF3
19F NMR (376 MHz, CDCl3)
20
10
0
-10
-20
-30
-40
-50
-60
-70
-80
-90
-100
-110
-120
-130
-140
-150
-160
-170
-180
-190
-200
-210
Chemical Shift [ppm]

## 7) Hammett Analysis

The Hammett analysis was carried out through a series of binary competition reactions.[20-22]

*a) Substituted carboxylic acids*

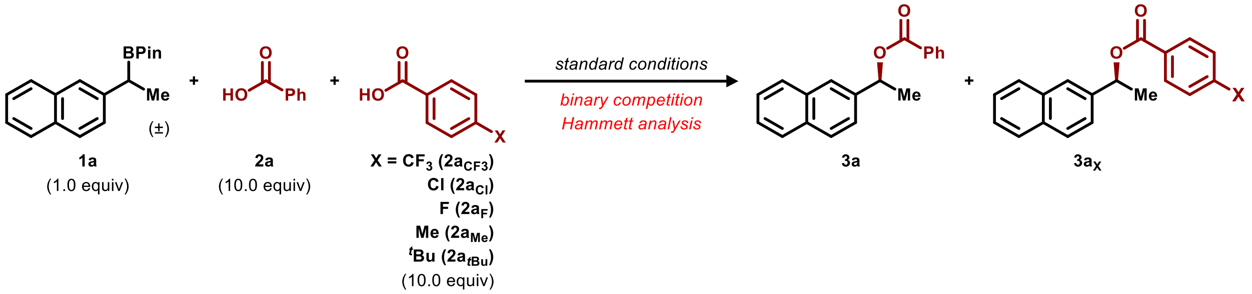


Inside a nitrogen-filled glovebox, an oven-dried reaction tube (Fisherbrand, 16 × 125 mm, catalog no. 1495935A) equipped with a magnetic stir bar was charged with CuCl (5.0 mg, 0.050 mmol, 10 mol%), NaBAr$^F{}_4$ (66.5 mg, 0.075 mmol, 15 mol%), and **L6** (73.5 mg, 0.075 mmol, 15 mol%) followed by the addition of a 1:1 mixture of PhCl and PhMe (2.5 mL). The reaction mixture was stirred at room temperature for 10 min before the addition of **4e** (14.0 mg ,0.050 mmol, 10 mol%), alkylborane **1a** (141 mg, 0.50 mmol, 1.0 equiv), and benzoic acid **2a** (610 mg, 5.00 mmol, 10.0 equiv) and substituted carboxylic acid **2a$_X$** (5.00 mmol, 10.0 equiv). The reaction mixture was stirred for 10 min before di-*tert*-butyl peroxide (183 µL, 1.00 mmol, 2.0 equiv) was added in one portion. The reaction tube was sealed with a screw cap (Kimble Chase Open Top S/T Closure, catalog no. 73804-15425) containing a PTFE septum (Thermo Scientific, catalog no. B7995-15), taken outside of the glovebox, placed in a –20 °C freezer and irradiated with 427 nm light (Kessil P160L) with stirring.

After the reaction mixture had stirred for 16 h, the screw cap was removed from the reaction tube, and the reaction mixture was concentrated *in vacuo* with the aid of a rotary evaporator. The resulting crude material was analyzed by $^1$H NMR spectroscopy using 1,1,2,2-tetrachloromethane as internal standard.

Products **3a$_{CF3}$**, **3a$_{Cl}$**, **3a$_F$**, **3a$_{Me}$**, and **3a$_{tBu}$** are synthesized independently[23] on a 0.10 mmol scale to aid the analysis.

*Result*:

| | Hammett parameter | [**3a**$_X$]/[**3a**] | log([**3a**$_X$]/[**3a**]) |
|---|---|---|---|
| **3a$_{CF3}$** | 0.54 | 8.64 | 0.94 |
| **3a$_{Cl}$** | 0.23 | 2.46 | 0.39 |
| **3a$_F$** | 0.06 | 2.07 | 0.32 |
| **3a$_{Me}$** | –0.17 | 0.85 | –0.07 |
| **3a$_{tBu}$** | –0.2 | 0.61 | –0.21 |

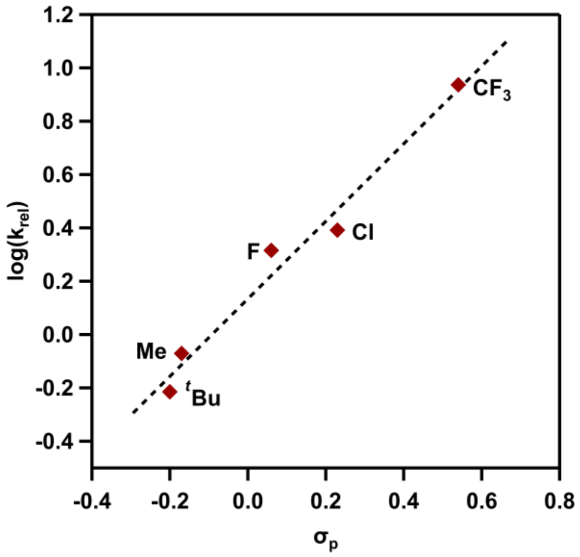


*Characterization data*:

(See "8.Associated Analytical Data" for the corresponding NMR spectra.)

**1-(naphthalen-2-yl)ethyl 4-(trifluoromethyl)benzoate (3a$_{CF3}$)**

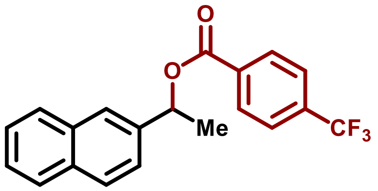


**$^1$H NMR** (400 MHz; CDCl$_3$) δ 8.21 (d, $J$ = 8.4 Hz, 2H), 7.94–7.79 (m, 4H), 7.71 (d, $J$ = 8.4 Hz, 2H), 7.58 (d, $J$ = 8.4 Hz, 1H), 7.54–7.42 (m, 2H), 6.32 (q, $J$ = 6.6 Hz, 1H), 1.79 (d, $J$ = 6.6 Hz, 3H).

**$^{13}$C NMR** (101 MHz; CDCl$_3$) δ 164.80, 138.72, 134.75, 133.87, 133.32, 133.27, 130.21, 128.72, 128.21, 127.85, 126.51, 126.38, 125.55 (q, $J$ = 3.6 Hz), 125.31, 124.09, 123.79 (q, $J$ = 272.8 Hz), 73.99, 22.36.

**$^{19}$F NMR** (376 MHz; $CDCl_3$) δ −63.11.

**HRMS** (DART) m/z: $[M+H]^+$ calcd for $C_{20}H_{16}F_3O_2^+$: 345.1097. Found: 345.1097.

**1-(naphthalen-2-yl)ethyl 4-chlorobenzoate (3a$_{Cl}$)**

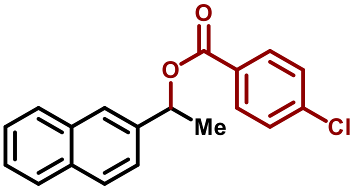


**$^1$H NMR** (400 MHz; $CDCl_3$) δ 8.04 (d, $J$ = 8.8 Hz, 2H), 7.91–7.80 (m, 4H), 7.57 (d, $J$ = 8.5 Hz, 1H), 7.52–7.45 (m, 2H), 7.44–7.39 (m, 2H), 6.29 (q, $J$ = 6.6 Hz, 1H), 1.77 (d, $J$ = 6.6 Hz, 3H).

**$^{13}$C NMR** (101 MHz; $CDCl_3$) δ 165.14, 139.54, 138.96, 133.31, 133.22, 131.20, 129.09, 128.85, 128.65, 128.19, 127.83, 126.45, 126.30, 125.22, 124.14, 73.57, 22.40.

**HRMS** (DART) m/z: $[M+H]^+$ calcd for $C_{19}H_{16}ClO_2^+$: 311.0833. Found: 311.0835.

**1-(naphthalen-2-yl)ethyl 4-fluorobenzoate (3a$_F$)**

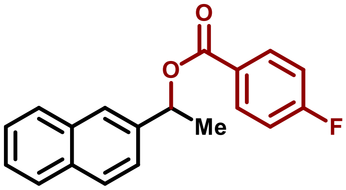


**$^1$H NMR** (400 MHz; $CDCl_3$) δ 8.15–8.07 (m, 2H), 7.91–7.79 (m, 4H), 7.57 (dd, $J$ = 8.5, 1.9 Hz, 1H), 7.53–7.44 (m, 2H), 7.12 (t, J = 8.5 Hz, 2H), 6.29 (q, $J$ = 6.6 Hz, 1H), 1.76 (d, $J$ = 6.6 Hz, 3H).

**$^{13}$C NMR (**101 MHz; CDCl3) δ 165.92 (d, $J$ = 253.8 Hz), 165.05, 139.06, 133.33, 133.22, 132.35 (d, $J$ = 9.5 Hz), 128.63, 128.20, 127.83, 126.89 (d, $J$ = 3.1 Hz), 126.43, 126.28, 125.21, 124.17, 115.65 (d, $J$ = 22.1 Hz), 73.42, 22.43.

**$^{19}$F NMR** (376 MHz; $CDCl_3$) δ −105.77.

Spectroscopic data is consistent with reported values.[24]

**1-(naphthalen-2-yl)ethyl 4-methylbenzoate (3a$_{Me}$)**

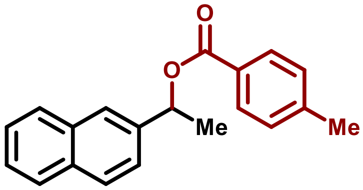


**$^{1}$H NMR** (400 MHz; $CDCl_3$) δ 7.99 (d, *J* = 8.3 Hz, 2H), 7.91–7.79 (m, 4H), 7.58 (d, *J* = 8.3 Hz, 1H), 7.51–7.43 (m, 2H), 7.25 (d, *J* = 7.9 Hz, 2H), 6.29 (d, *J* = 6.6 Hz, 1H), 2.41 (s, 3H), 1.75 (d, *J* = 6.6 Hz, 3H).

**$^{13}$C NMR** (101 MHz; $CDCl_3$) δ 166.07, 143.76, 139.41, 133.35, 133.16, 129.85, 129.21, 128.54, 128.20, 127.93, 127.81, 126.34, 126.17, 125.08, 124.25, 72.96, 22.52, 21.82.

**HRMS** (DART) m/z: $[M+H]^+$ calcd for $C_{20}H_{19}O_2^+$: 291.1380. Found: 291.1382.

**1-(naphthalen-2-yl)ethyl 4-(*tert*-butyl)benzoate (3a$_{tBu}$)**

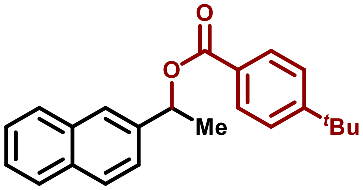


**$^{1}$H NMR** (400 MHz; $CDCl_3$) δ 8.04 (d, *J* = 8.6 Hz, 2H), 7.91–7.81 (m, 4H), 7.57 (d, *J* = 8.4 Hz, 1H), 7.51–7.42 (m, 4H), 6.30 (q, *J* = 6.6 Hz, 1H), 1.75 (d, *J* = 6.6 Hz, 3H), 1.34 (s, 9H).

**$^{13}$C NMR** (101 MHz; $CDCl_3$) δ 166.02, 156.77, 139.44, 133.36, 133.16, 129.70, 128.53, 128.21, 127.90, 127.81, 126.34, 126.16, 125.49, 125.05, 124.24, 72.90, 35.23, 31.27, 22.53.

**HRMS** (DART) m/z: $[M+H]^+$ calcd for $C_{23}H_{25}O_2^+$: 333.1849. Found: 333.1845.

*b) Substituted alkylboron reagents*

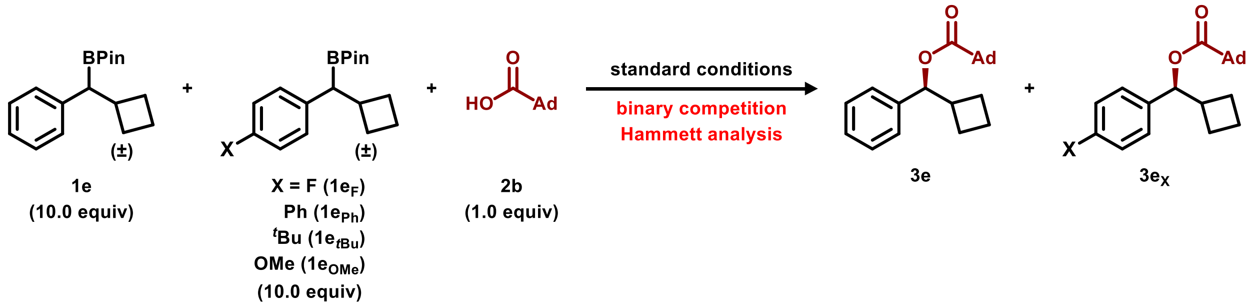


Inside a nitrogen-filled glovebox, an oven-dried reaction tube (Fisherbrand, 16 × 125 mm, catalog no. 1495935A) equipped with a magnetic stir bar was charged with CuCl (5.0 mg, 0.050 mmol, 10 mol%), NaBAr$^F{}_4$ (66.5 mg, 0.075 mmol, 15 mol%), and **L6** (73.5 mg, 0.075 mmol, 15 mol%) followed by the addition of a 1:1 mixture of PhCl and PhMe (2.5 mL). The reaction mixture was stirred at room temperature for 10 min before the addition of **4e** (14.0 mg ,0.050 mmol, 10 mol%), 1-adamantanecarboxylic acid **2b** (90.1 mg, 0.50 mmol, 1.0 equiv), alkylborane **1e** (1.36 g mg, 5.00 mmol, 10.0 equiv), and substituted alkylborane **1ex** (5.00 mmol, 10.0 equiv). The reaction mixture was stirred for 10 min before di-*tert*-butyl peroxide (183 µL, 1.00 mmol, 2.0 equiv) was added in one portion. The reaction tube was sealed with a screw cap (Kimble Chase Open Top S/T Closure, catalog no. 73804-15425) containing a PTFE septum (Thermo Scientific, catalog no. B7995-15), taken outside of the glovebox, placed in a –20 °C freezer and irradiated with 427 nm light (Kessil P160L) with stirring.

After the reaction mixture had stirred for 16 h, the screw cap was removed from the reaction tube, and the reaction mixture was concentrated *in vacuo* with the aid of a rotary evaporator. The resulting crude material was analyzed by $^1$H NMR spectroscopy using 1,1,2,2-tetrachloromethane as internal standard.

Products **3eF**, **3ePh**, **3etBu**, and **3eOMe** are synthesized independently on a 0.10 mmol scale to aid the analysis.

*Result*:

| | Hammett | [**3e**$_X$]/[**3e**] | log([**3e**$_X$]/[**3e**]) | *e.r.* | log(*e.r.*) |
|---|---|---|---|---|---|
| **3e**$_F$ | 0.06 | 0.96 | -0.02 | 90:10 | 0.95 |
| **3e**$_{Ph}$ | −0.01 | 1.16 | 0.06 | 89:11 | 0.91 |
| **3e**$_{tBu}$ | −0.2 | 1.77 | 0.25 | 87:13 | 0.83 |
| **3e**$_{OMe}$ | −0.27 | 3.45 | 0.54 | 83:17 | 0.69 |

a. Correlating relative reaction rate ($k_{rel}$) with substituent $\sigma_p$ parameters

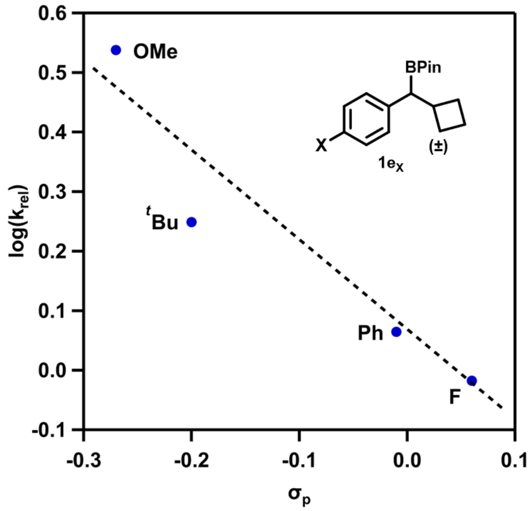


*Note*: Correlation of the relative rates with Hammett σ parameters gave a negative ρ value. This trend contrasts with the Hammett analysis using substituted benzoic acids (Figure 3E) and is consistent with faster capture of more electron-rich benzyl radicals by the electrophilic cationic Cu(II)–carboxylate intermediate and/or more rapid C–B bond cleavage by the carbazole-derived N-centered radical.

b. Correlating enantiomeric ratios (*e.r.*) with substituent $\sigma_p$ parameters

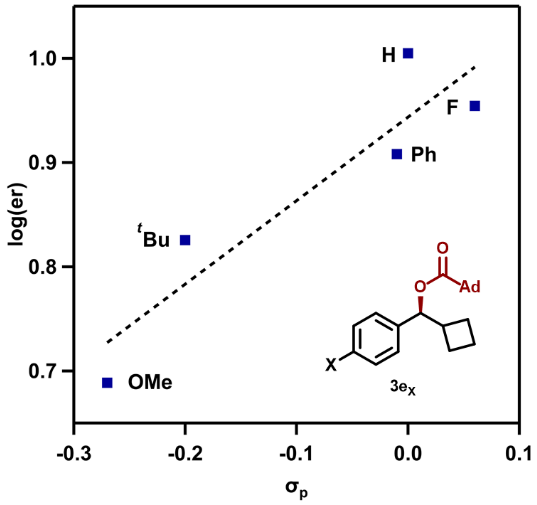

*Note*: This analysis revealed that more electron-rich substrates exhibit modest erosion in enantioselectivity, consistent with either a lower C–O bond-forming barrier that leads to a less discriminating enantiodetermining transition state and/or an increased propensity for the competing nonselective radical-polar crossover pathway.

*Characterization data*:

(See "8.Associated Analytical Data" for the corresponding NMR spectra.)

**2-(cyclobutyl(4-fluorophenyl)methyl)-4,4,5,5-tetramethyl-1,3,2-dioxaborolane (1e$_F$)**

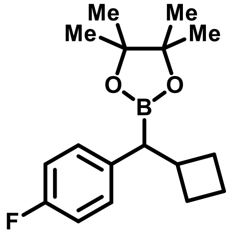


**$^1$H NMR** (400 MHz; $CDCl_3$) δ 7.17–7.07 (m, 2H), 6.97–6.87 (m, 2H), 2.81–2.61 (m, 1H), 2.30 (d, $J$ = 11.1 Hz, 1H), 2.17–2.06 (m, 1H), 1.94–1.63 (m, 4H), 1.59–1.48 (m, 1H), 1.20 (s, 6H), 1.18 (s, 6H).

**$^{13}$C NMR** (101 MHz; $CDCl_3$) δ 161.09 (d, $J$ = 242.0 Hz), 137.54 (d, $J$ = 3.1 Hz), 129.80 (d, $J$ = 7.6 Hz), 115.01 (d, $J$ = 20.8 Hz), 83.43, 38.67, 28.77, 28.61, 24.82, 24.71, 18.24.

**$^{19}$F NMR** (376 MHz; $CDCl_3$) δ −118.93.

**HRMS** (DART) m/z: $[M+H]^+$ calcd for $C_{17}H_{25}BFO_2^+$: 291.1926. Found: 291.1930.

**2-([1,1'-biphenyl]-4-yl(cyclobutyl)methyl)-4,4,5,5-tetramethyl-1,3,2-dioxaborolane (1e$_{Ph}$)**

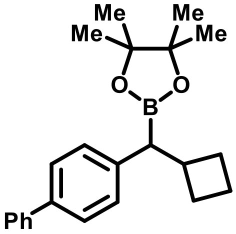


**$^1$H NMR** (400 MHz; $CDCl_3$) δ 7.58 (d, $J$ = 8.1 Hz, 2H), 7.48 (d, $J$ = 8.3 Hz, 2H), 7.44–7.36 (m, 2H), 7.30 (t, $J$ = 7.4 Hz, 1H), 7.24 (d, $J$ = 8.3 Hz, 2H), 2.86–2.69 (m, 1H), 2.39 (d, $J$ = 11.0 Hz,

1H), 2.22–2.10 (m, 1H), 2.01–1.88 (m, 1H), 1.88–1.69 (m, 3H), 1.67–1.55 (m, 1H), 1.22 (s, 6H), 1.20 (s, 6H).

**$^{13}$C NMR** (101 MHz; $CDCl_3$) δ 141.38, 141.19, 138.02, 129.00, 128.78, 127.04, 127.02, 126.94, 83.42, 38.58, 29.00, 28.74, 24.87, 24.75, 18.34.

**HRMS** (DART) m/z: $[M+H]^+$ calcd for $C_{23}H_{30}BO_2^+$: 349.2333. Found: 349.2340.

**2-((4-(tert-butyl)phenyl)(cyclobutyl)methyl)-4,4,5,5-tetramethyl-1,3,2-dioxaborolane (1e$_{t\text{Bu}}$)**

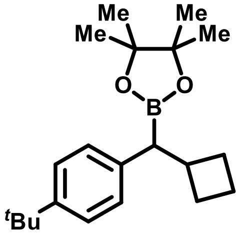


**$^1$H NMR** (400 MHz; $CDCl_3$) δ 7.24 (d, $J$ = 8.4 Hz, 2H), 7.09 (d, $J$ = 8.4 Hz, 2H), 2.79–2.62 (m, 1H), 2.31 (d, $J$ = 10.8 Hz, 1H), 2.18–2.08 (m, 1H), 1.97–1.86 (m, 1H), 1.84–1.52 (m, 4H), 1.29 (s, 9H), 1.21 (s, 6H), 1.19 (s, 6H).

**$^{13}$C NMR** (101 MHz; $CDCl_3$) δ 147.73, 138.68, 128.14, 125.17, 83.28, 38.74, 34.39, 31.58, 29.13, 28.70, 24.88, 24.76, 18.33.

**HRMS** (DART) m/z: $[M+H]^+$ calcd for $C_{21}H_{34}BO_2^+$: 329.2646. Found: 329.2660.

**2-(cyclobutyl(4-methoxyphenyl)methyl)-4,4,5,5-tetramethyl-1,3,2-dioxaborolane (1e$_{\text{OMe}}$)**

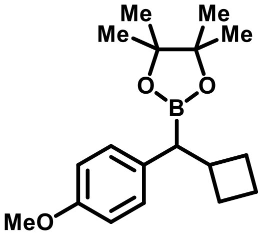


**$^1$H NMR** (400 MHz; $CDCl_3$) δ 7.09 (d, $J$ = 8.4 Hz, 2H), 6.79 (d, $J$ = 8.4 Hz, 2H), 3.77 (s, 3H), 2.75–2.63 (m, 1H), 2.27 (d, $J$ = 11.0 Hz, 1H), 2.17–2.08 (m, 1H), 1.93–1.84 (m, 1H), 1.84–1.65 (m, 3H), 1.61–1.50 (m, 1H), 1.20 (s, 6H), 1.18 (s, 6H).

**$^{13}$C NMR** (101 MHz; $CDCl_3$) δ 157.41, 133.97, 129.44, 113.75, 83.28, 55.27, 38.76, 28.84, 28.62, 24.84, 24.71, 18.26.

**HRMS** (DART) m/z: $[M+H]^+$ calcd for $C_{18}H_{28}BO_3^+$: 303.2126. Found: 303.2120.

**cyclobutyl(4-fluorophenyl)methyl adamantane-1-carboxylate (3e$_F$)**

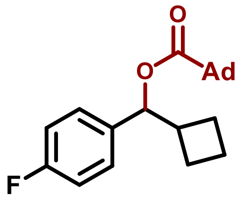


**$^1$H NMR** (400 MHz; $CDCl_3$) δ 7.26–7.20 (m, 2H), 7.03–6.94 (m, 2H), 5.64 (d, *J* = 7.8 Hz, 1H), 2.79–2.62 (m, 1H), 2.06–1.93 (m, 5H), 1.93–1.87 (m, 6H), 1.86–1.60 (m, 10H).

**$^{13}$C NMR** (101 MHz; $CDCl_3$) δ 177.08, 162.33 (d, *J* = 245.7 Hz), 135.95 (d, *J* = 3.3 Hz), 128.16 (d, *J* = 8.0 Hz), 115.30 (d, *J* = 21.4 Hz), 77.59, 41.05, 40.71, 39.01, 36.67, 28.11, 24.70, 24.46, 17.93.

**$^{19}$F NMR** (376 MHz; $CDCl_3$) δ −114.94.

**HRMS** (DART) m/z: $[M+H]^+$ calcd for $C_{22}H_{28}FO_2^+$: 343.2068. Found: 343.2060.

**[1,1'-biphenyl]-4-yl(cyclobutyl)methyl adamantane-1-carboxylate (3e$_{Ph}$)**

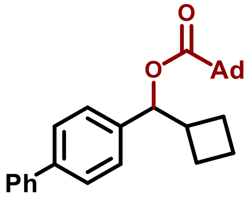


**$^1$H NMR** (400 MHz; $CDCl_3$) δ 7.59–7.50 (m, 4H), 7.46–7.39 (m, 2H), 7.37–7.30 (m, 3H), 5.71 (d, *J* = 7.6 Hz, 1H), 2.85–2.68 (m, 1H), 2.10–1.97 (m, 5H), 1.97–1.92 (m, 6H), 1.92–1.79 (m, 4H), 1.80–1.66 (m, 6H).

**$^{13}$C NMR (**101 MHz; $CDCl_3$) δ 177.16, 141.02, 140.55, 139.22, 128.87, 127.36, 127.22, 127.19, 126.87, 77.95, 41.11, 40.81, 39.07, 36.71, 28.15, 24.71, 24.57, 18.01.

**HRMS** (DART) m/z: $[M+H]^+$ calcd for $C_{28}H_{33}O_2^+$: 401.2475. Found: 401.2479.

**(4-(tert-butyl)phenyl)(cyclobutyl)methyl adamantane-1-carboxylate ($3e_{tBu}$)**

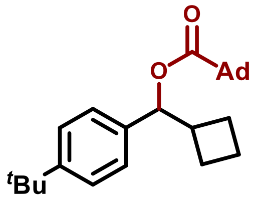


**$^{1}$H NMR** (400 MHz; $CDCl_3$) δ 7.31 (d, *J* = 8.4 Hz, 2H), 7.18 (d, *J* = 8.4 Hz, 2H), 5.67 (d, *J* = 7.5 Hz, 1H), 2.83–2.64 (m, 1H), 2.08–1.96 (m, 5H), 1.96–1.90 (m, 6H), 1.89–1.77 (m, 4H), 1.78–1.67 (m, 6H), 1.30 (s, 9H).

**$^{13}$C NMR** (101 MHz; $CDCl_3$) δ 177.14, 150.33, 137.07, 126.06, 125.27, 77.86, 40.88, 39.07, 36.71, 34.61, 31.49, 28.16, 27.82, 24.66, 24.63, 17.98.

**HRMS** (DART) m/z: $[M+H]^+$ calcd for $C_{26}H_{37}O_2^+$: 381.2788. Found: 381.2788.

**cyclobutyl(4-methoxyphenyl)methyl adamantane-1-carboxylate ($3e_{OMe}$)**

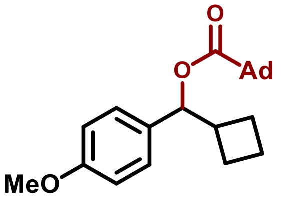


**$^{ll1}$H NMR** (400 MHz; $CDCl_3$) δ 7.20 (d, *J* = 8.8 Hz, 2H), 6.84 (d, *J* = 8.8 Hz, 2H), 5.63 (d, *J* = 8.0 Hz, 1H), 3.78 (s, 3H), 2.85–2.57 (m, 1H), 2.07–1.92 (m, 6H), 1.92–1.87 (m, 6H), 1.86–1.78 (m, 3H), 1.76–1.67 (m, 6H).

**$^{13}$C NMR** (101 MHz; $CDCl_3$) δ 177.16, 159.12, 132.30, 127.84, 113.79, 78.01, 55.34, 41.04, 40.69, 39.03, 36.70, 28.14, 24.83, 24.53, 17.96.

**HRMS** (DART) m/z: $[M+H]^+$ calcd for $C_{23}H_{31}O_3^+$: 355.2268. Found: 355.2265.

## 8) UV-Vis Characterization of Reaction Components

The UV-Vis spectra of each reaction components and upon their mixing at −20 °C were recorded using an Edinburgh FS5 spectrofluorometer.

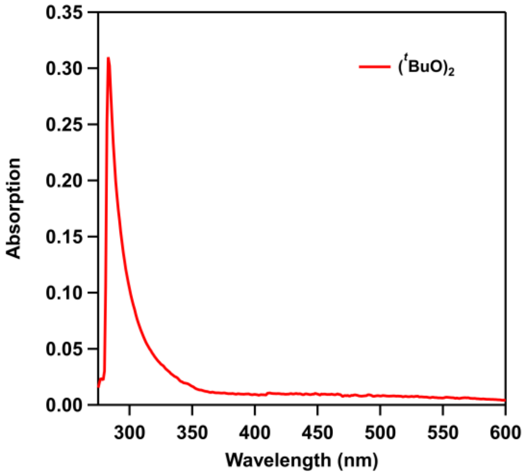


Note: The weak long-wavelength absorption of $(^t BuO)_2$, extending into the 350–450 nm region, likely enables photoinduced O–O homolysis under blue-light irradiation, consistent with recent UV–Vis data and prior photochemical studies of DTBP cleavage.[25-27]

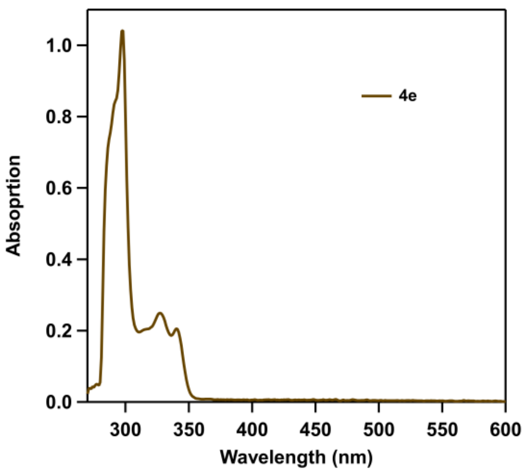


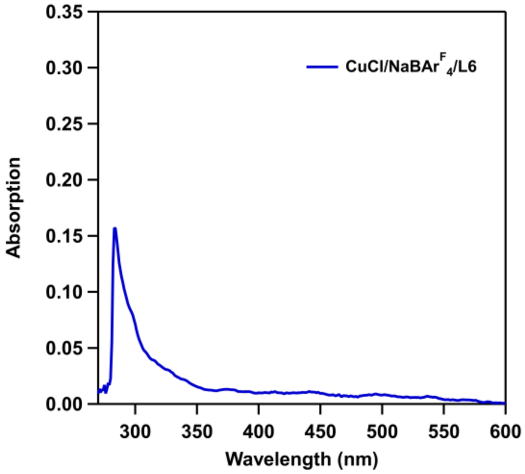


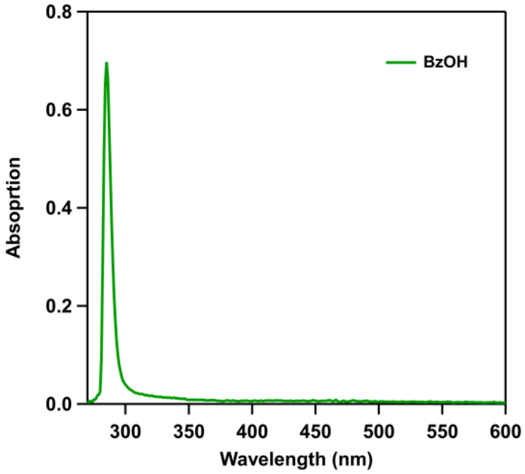


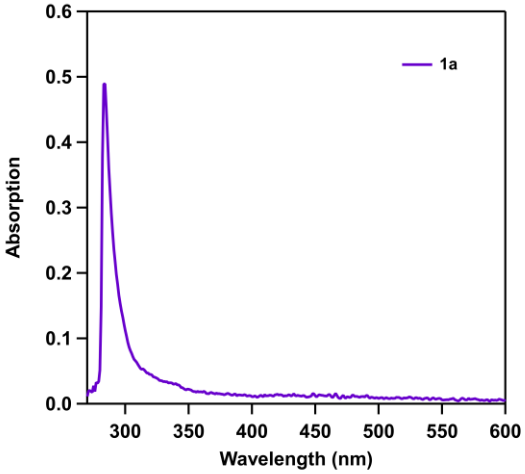

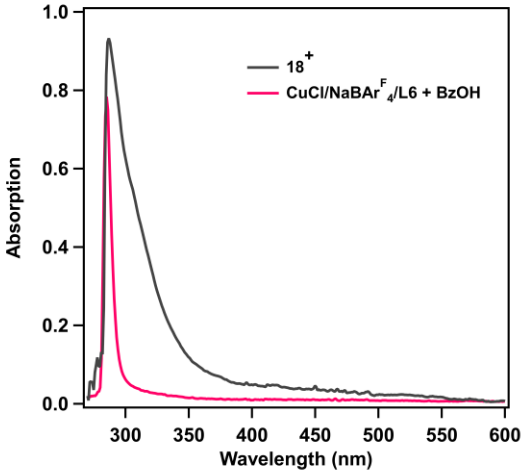


Note: The isolated Cu(II)–benzoate complex (**18**$^{+}$) exhibits features consistent with previous report by Zhou, et. al.[28]

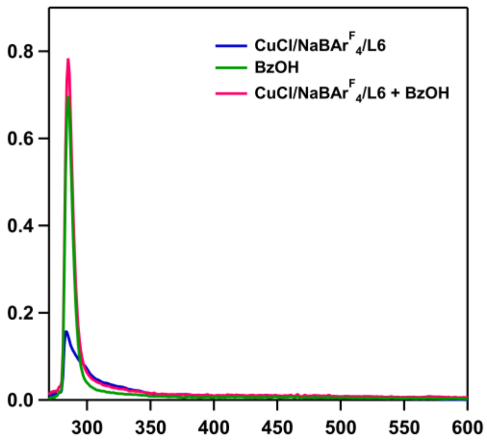


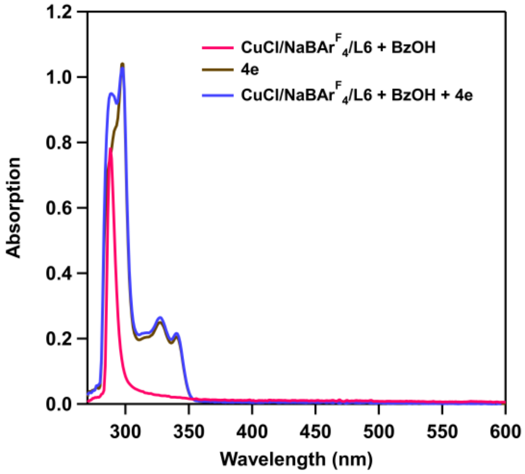


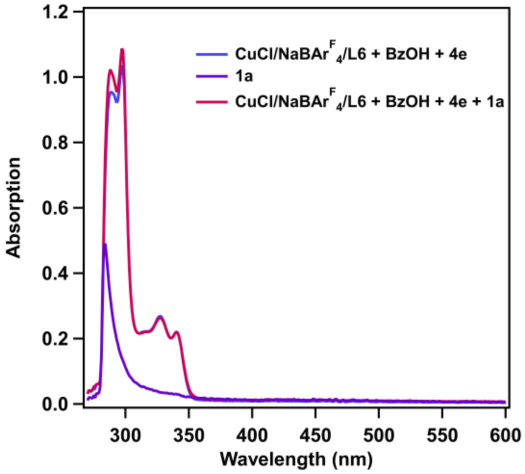


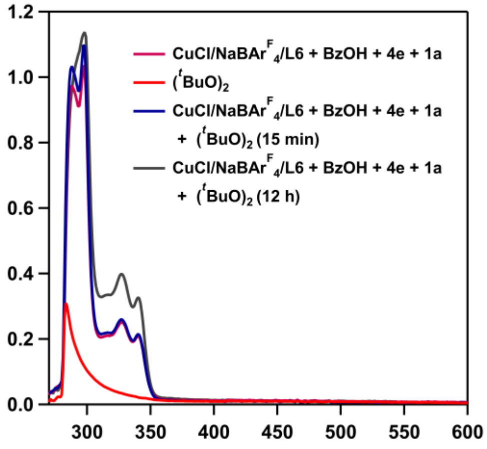

## 8. Associated Analytical Data

*NMR Data for Starting Materials*

*tert*-butyl 3-(naphthalen-2-yl(4,4,5,5-tetramethyl-1,3,2-dioxaborolan-2-yl)methyl)azetidine-1-carboxylate (**1c**)

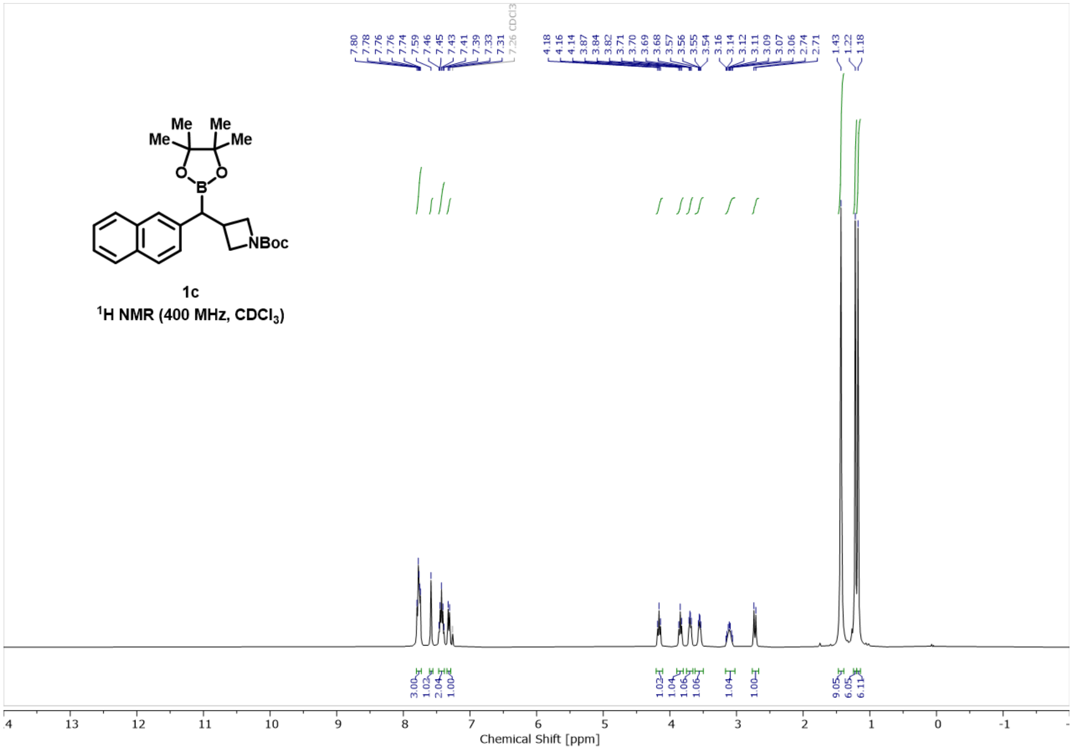


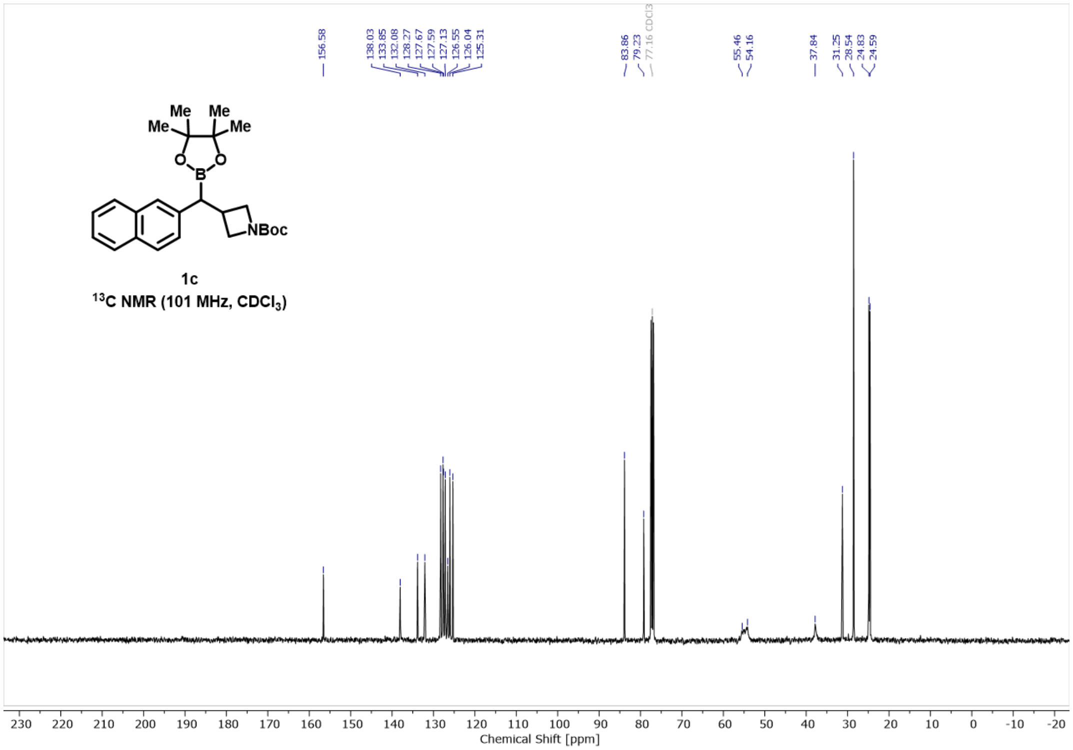

2-phenyl-4-(1-(4,4,5,5-tetramethyl-1,3,2-dioxaborolan-2-yl)butyl)thiazole (**1d**)

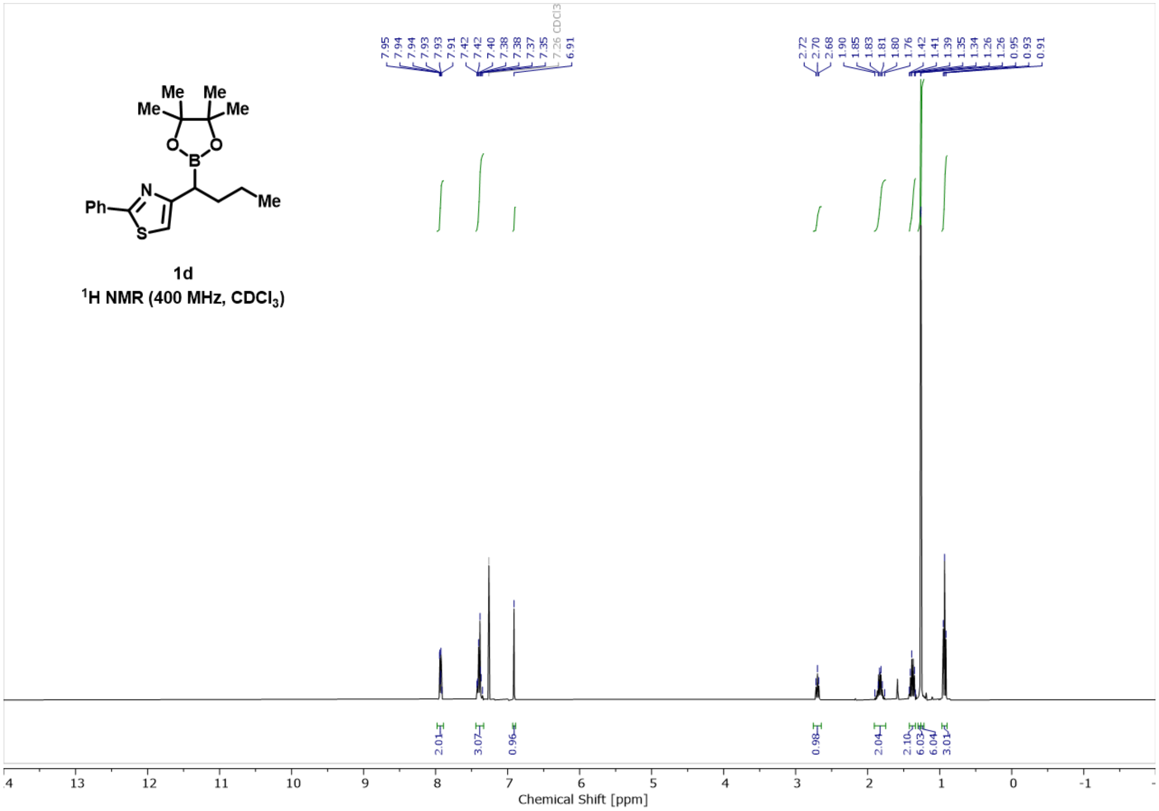


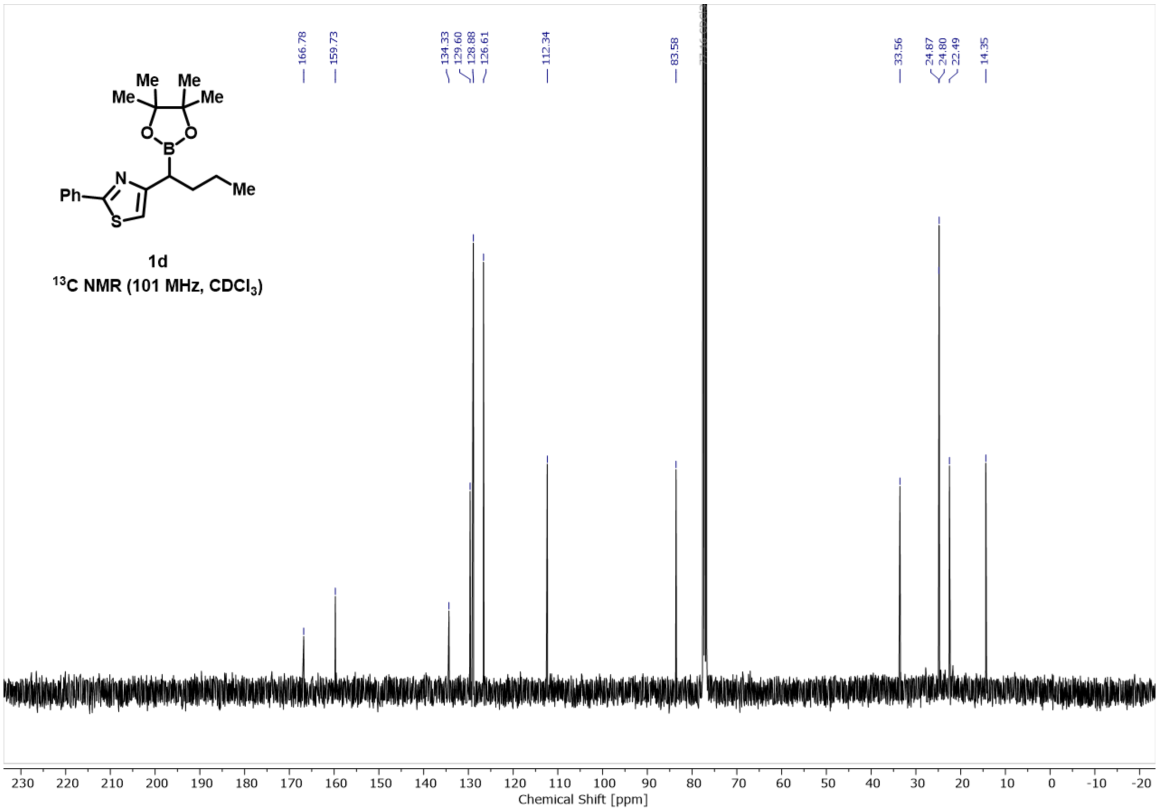

2-(1-(benzo[*b*]thiophen-5-yl)-3-phenylpropyl)-4,4,5,5-tetramethyl-1,3,2-dioxaborolane (**1f**)

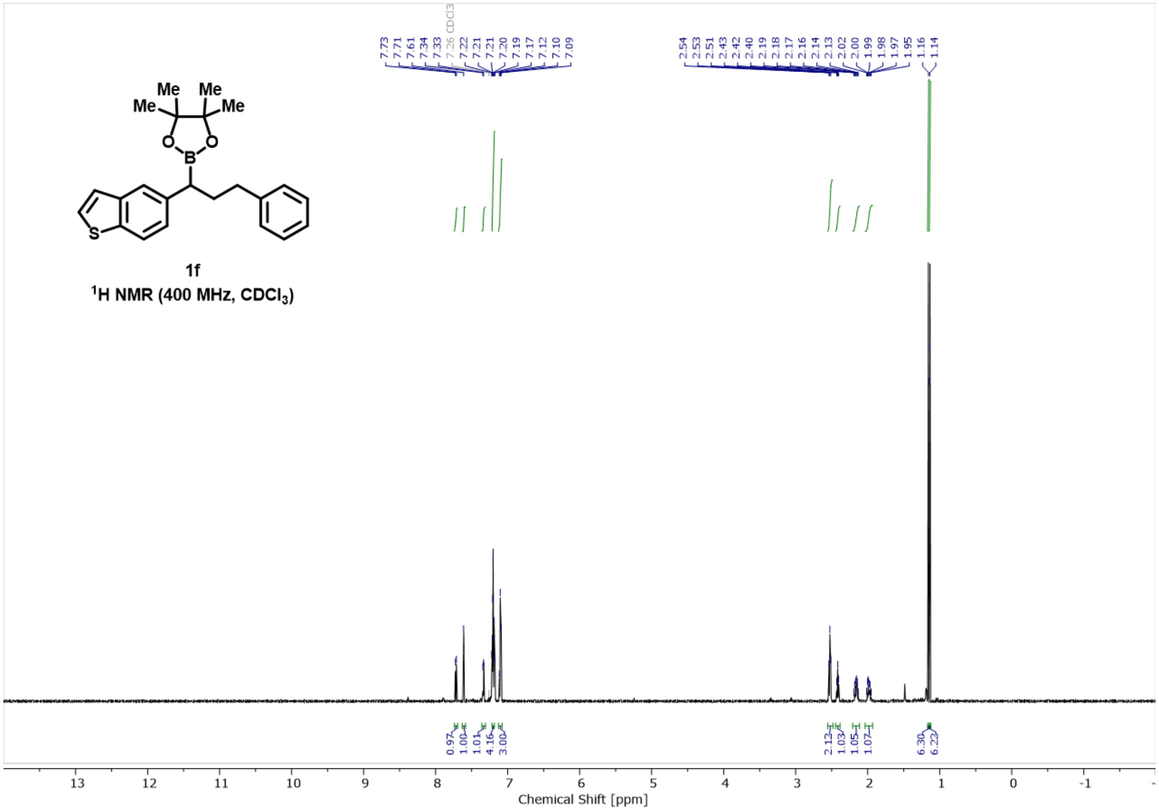


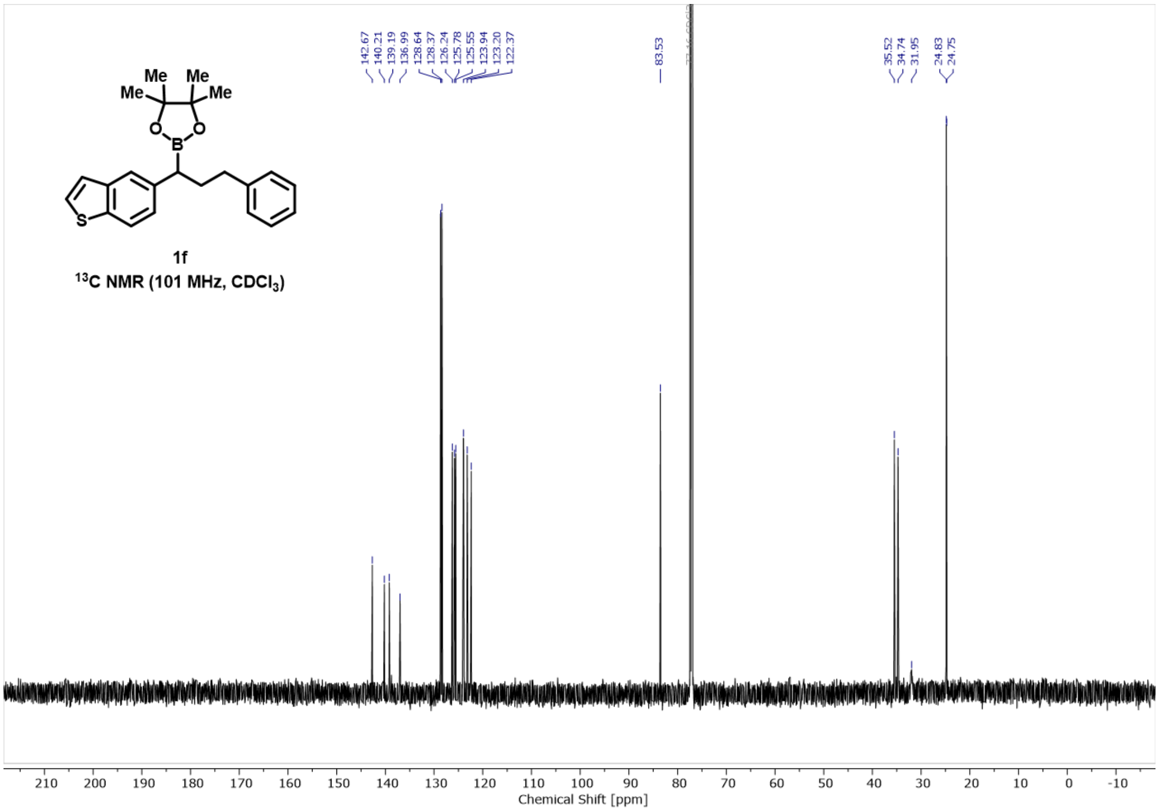

2-(1-(benzofuran-5-yl)butyl)-4,4,5,5-tetramethyl-1,3,2-dioxaborolane (**1g**)

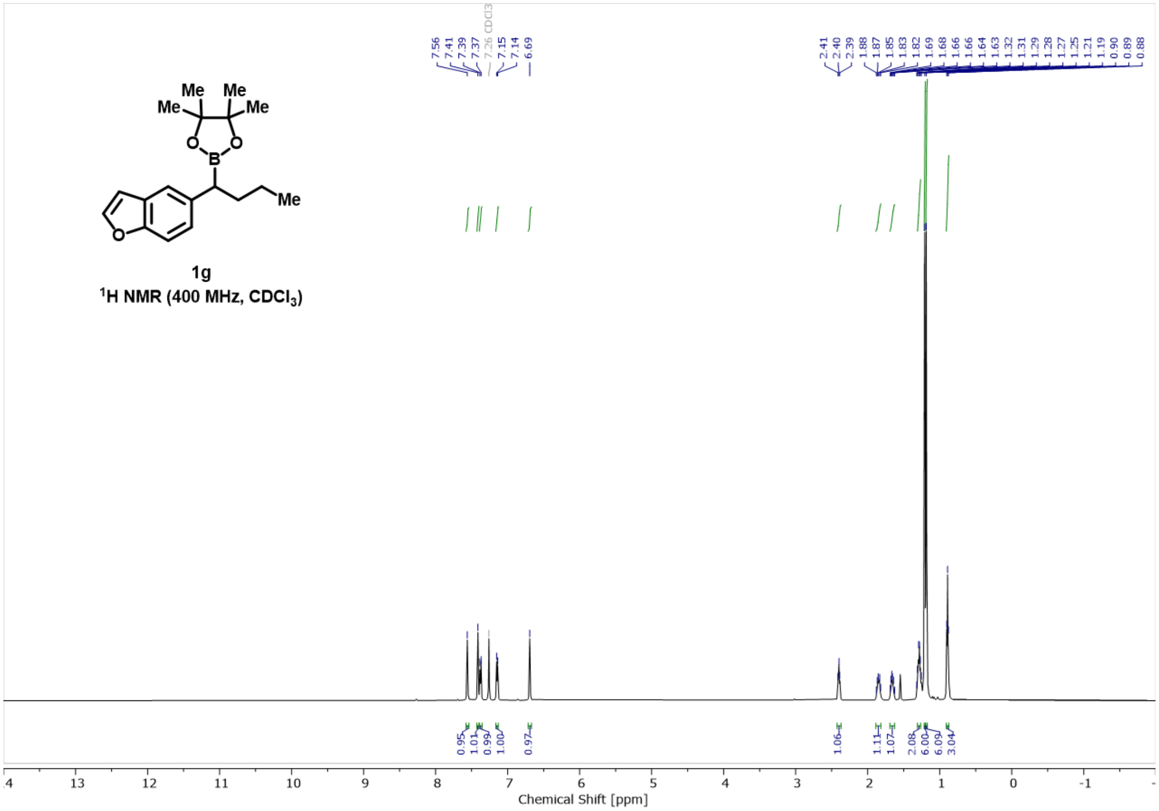


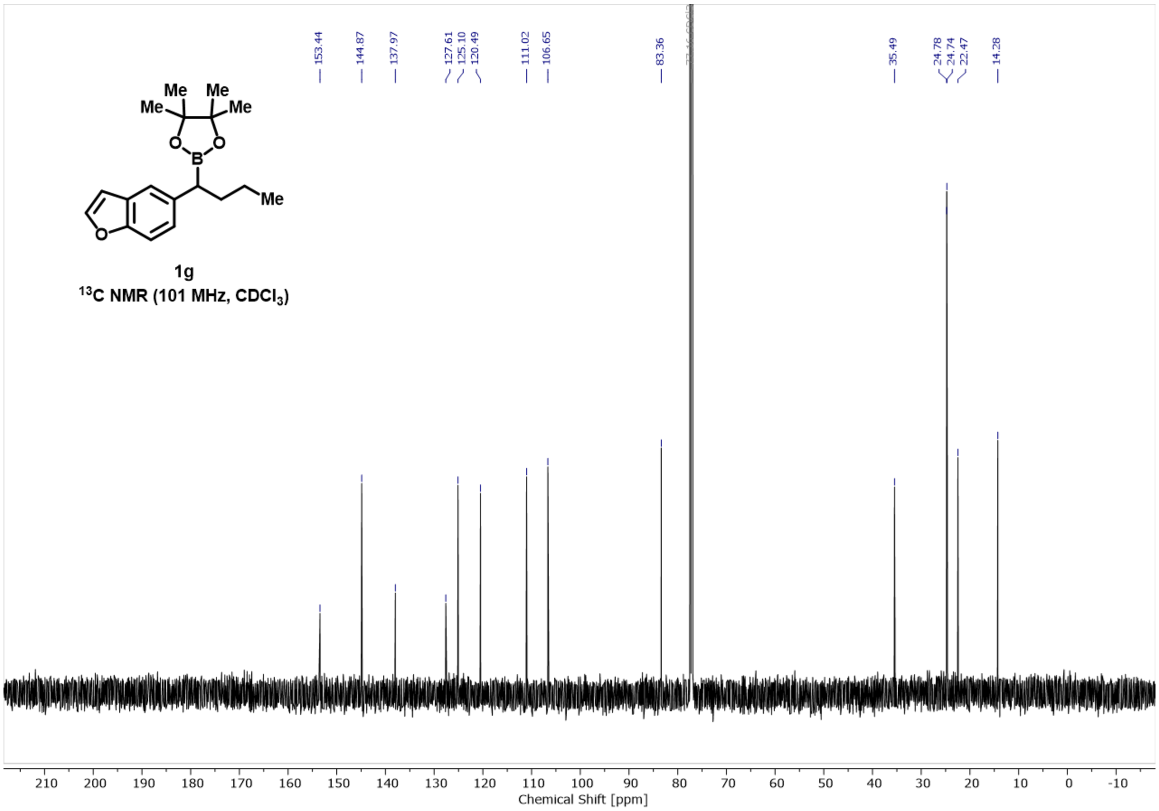

4,4,5,5-tetramethyl-2-(4-(2-(naphthalen-2-yl)-2-(4,4,5,5-tetramethyl-1,3,2-dioxaborolan-2-yl)ethyl)phenyl)-1,3,2-dioxaborolane (**1h**)

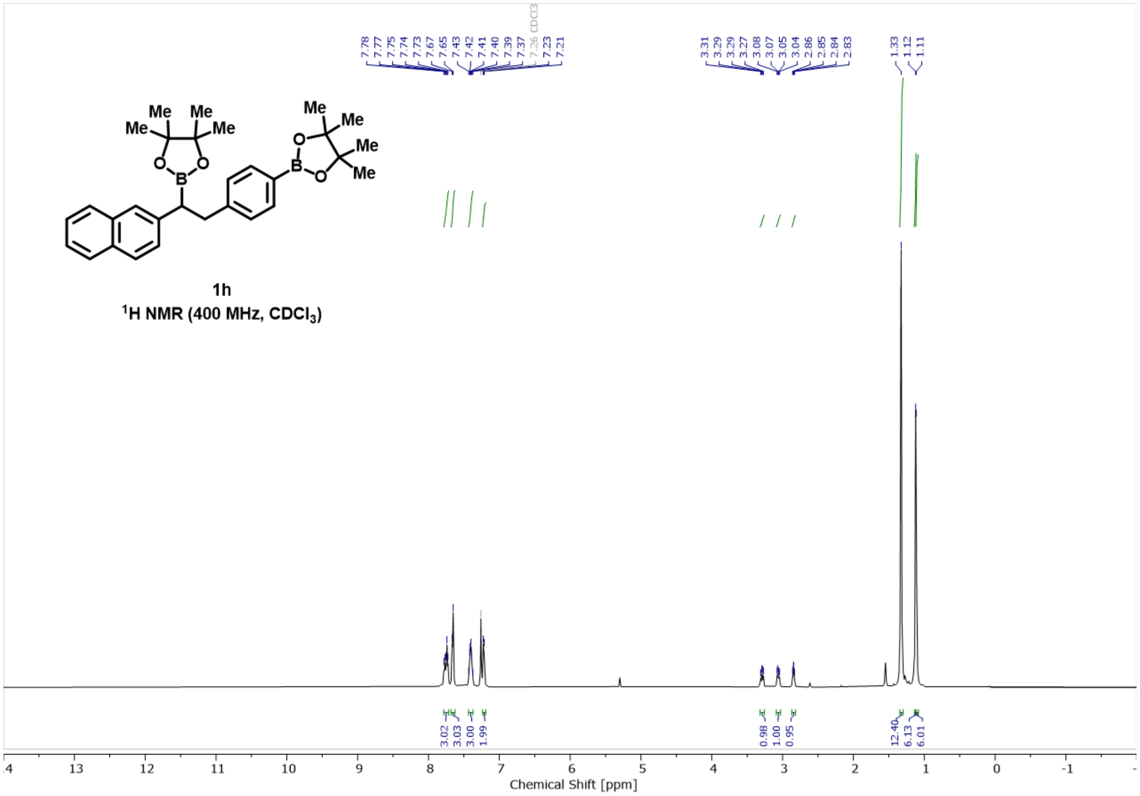


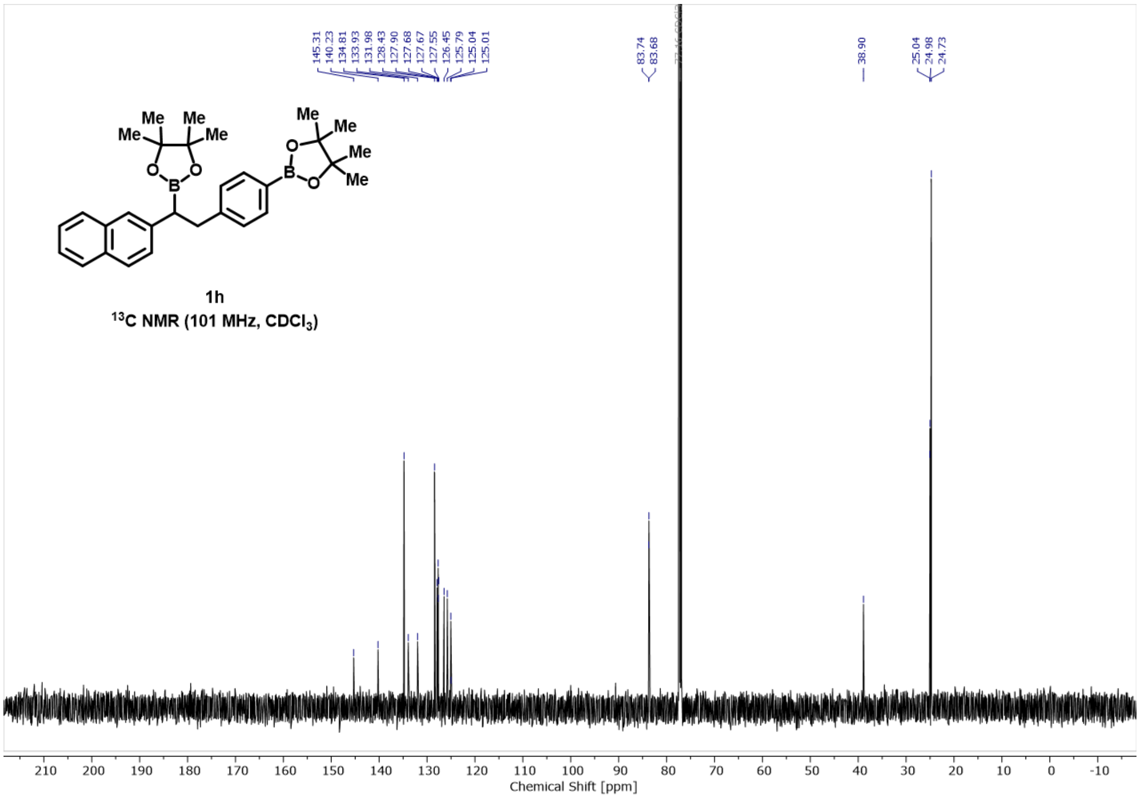

2,2'-(1-(naphthalen-2-yl)butane-1,4-diyl)bis(4,4,5,5-tetramethyl-1,3,2-dioxaborolane) (**1i**)

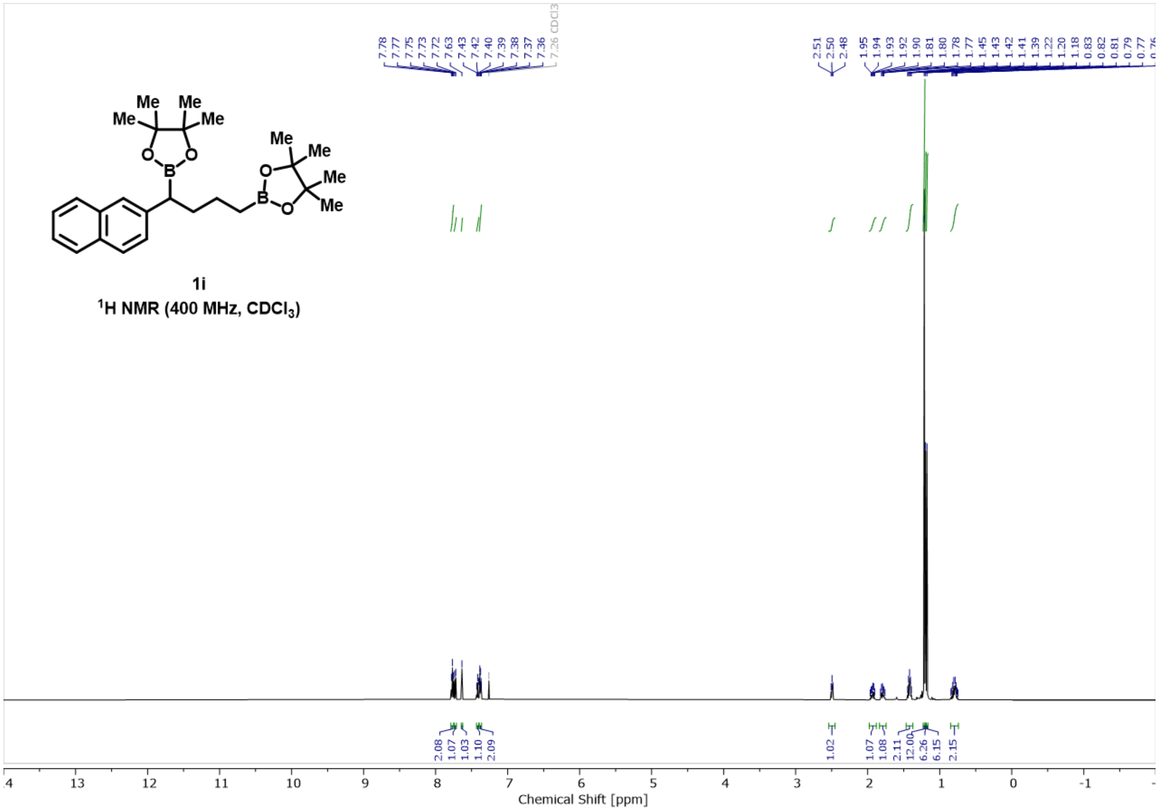


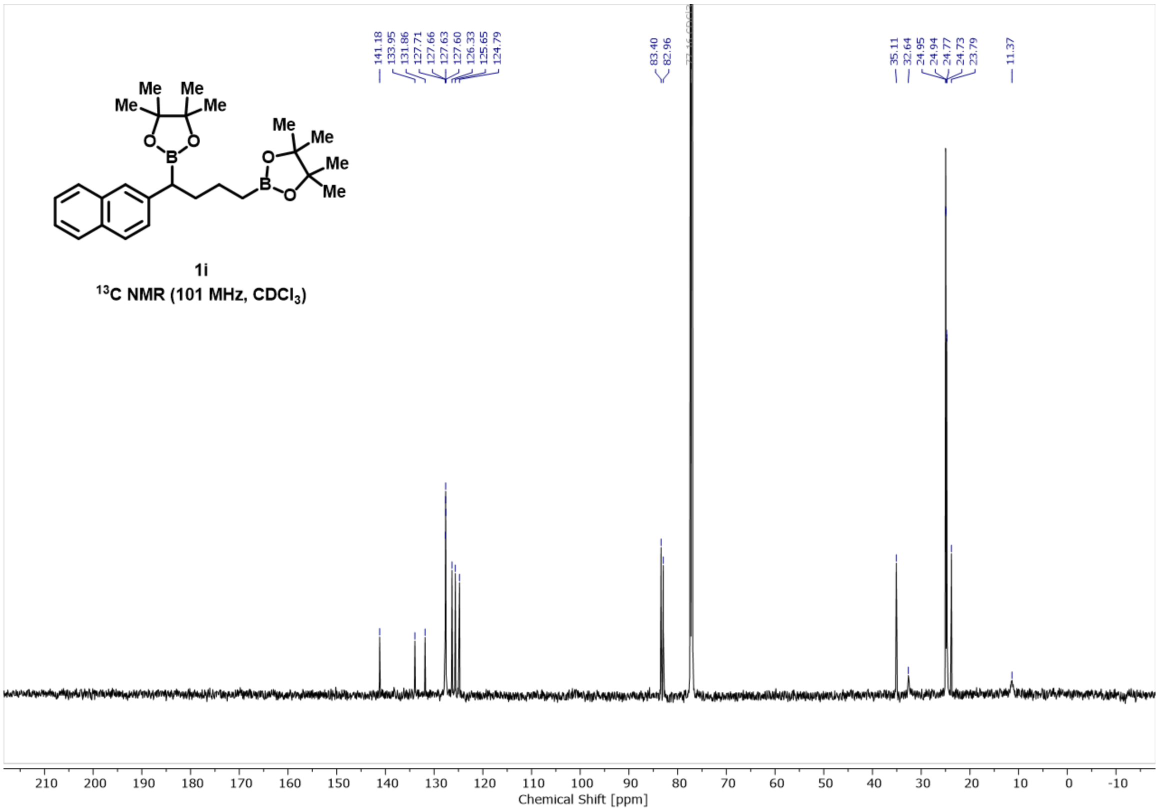

2-(3-chloro-1-(naphthalen-2-yl)propyl)-4,4,5,5-tetramethyl-1,3,2-dioxaborolane (**1j**)

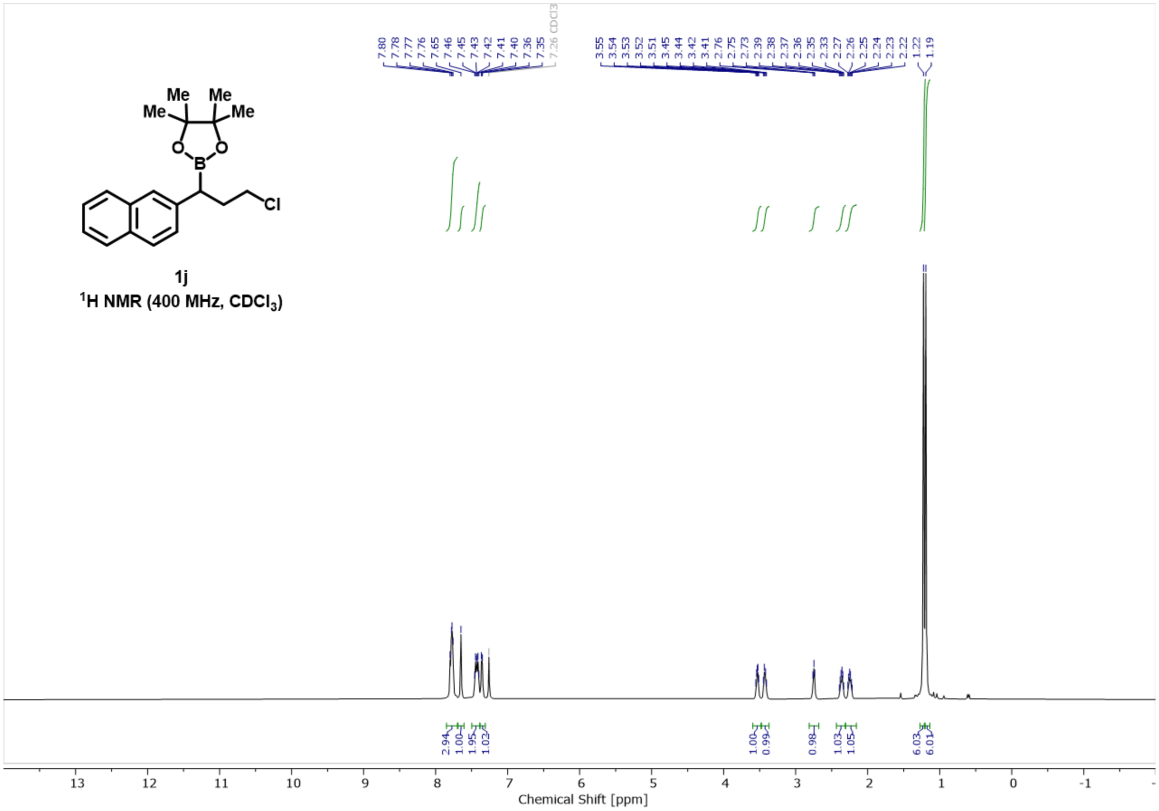


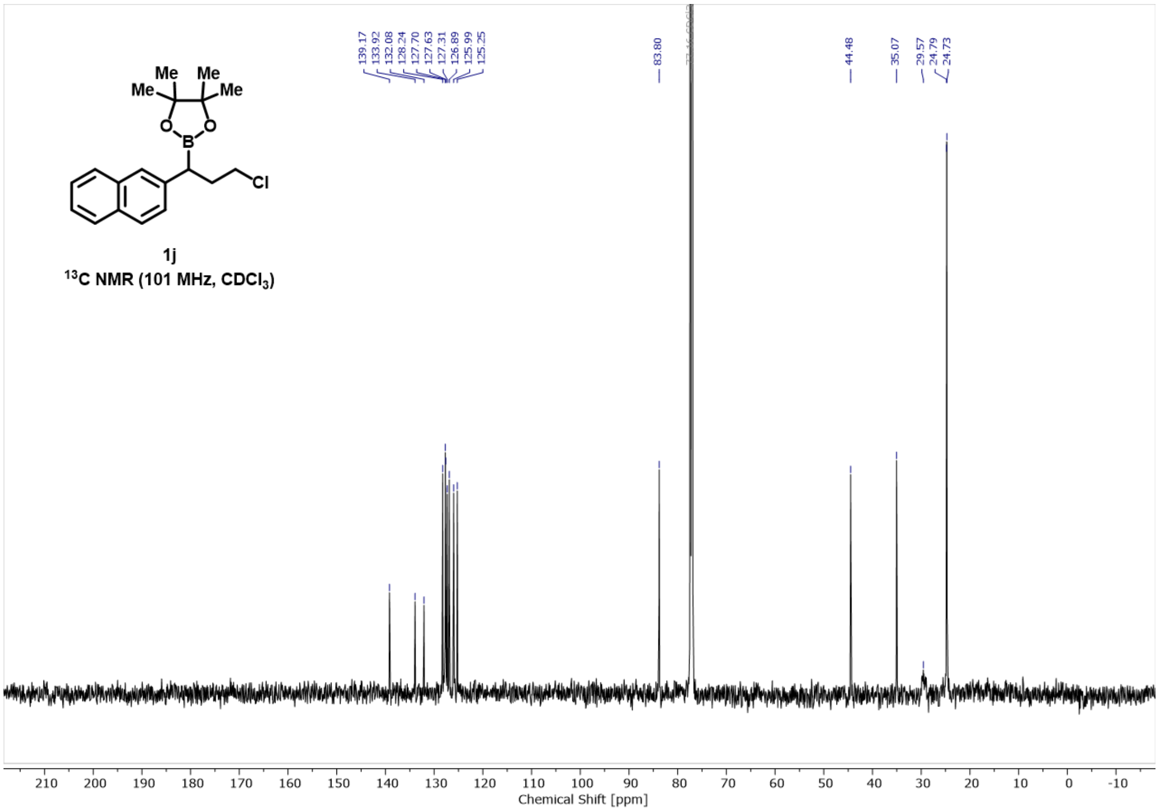

*tert*-butyl 3-(benzofuran-5-yl(4,4,5,5-tetramethyl-1,3,2-dioxaborolan-2-yl)methyl)azetidine-1-carboxylate (**1k**)

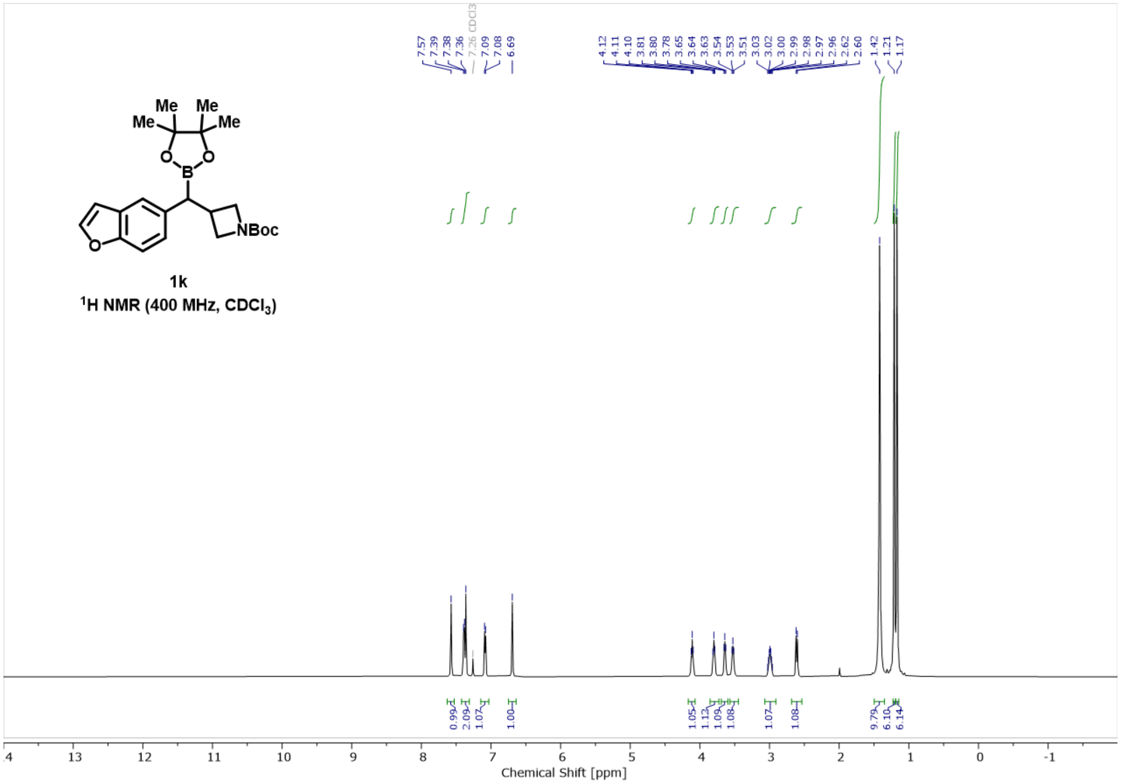


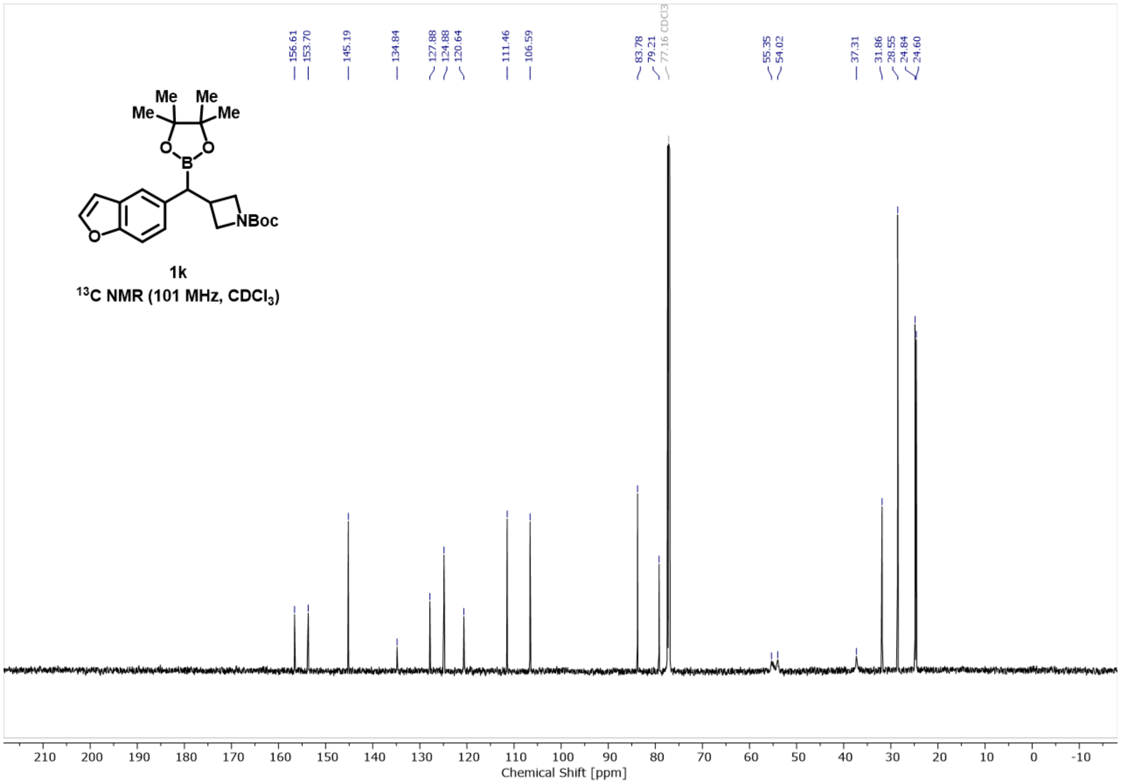

2-(3-(2,3-dihydrobenzofuran-5-yl)-1-(naphthalen-2-yl)propyl)-4,4,5,5-tetramethyl-1,3,2-dioxaborolane (**1l**)

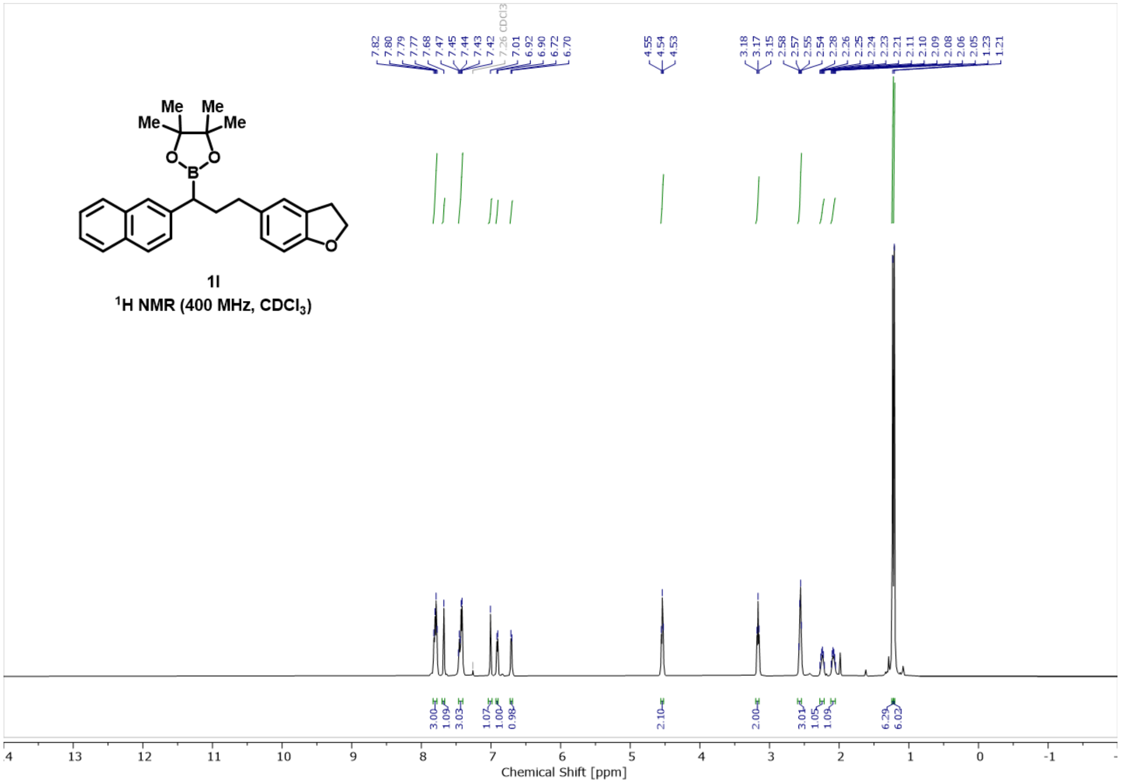

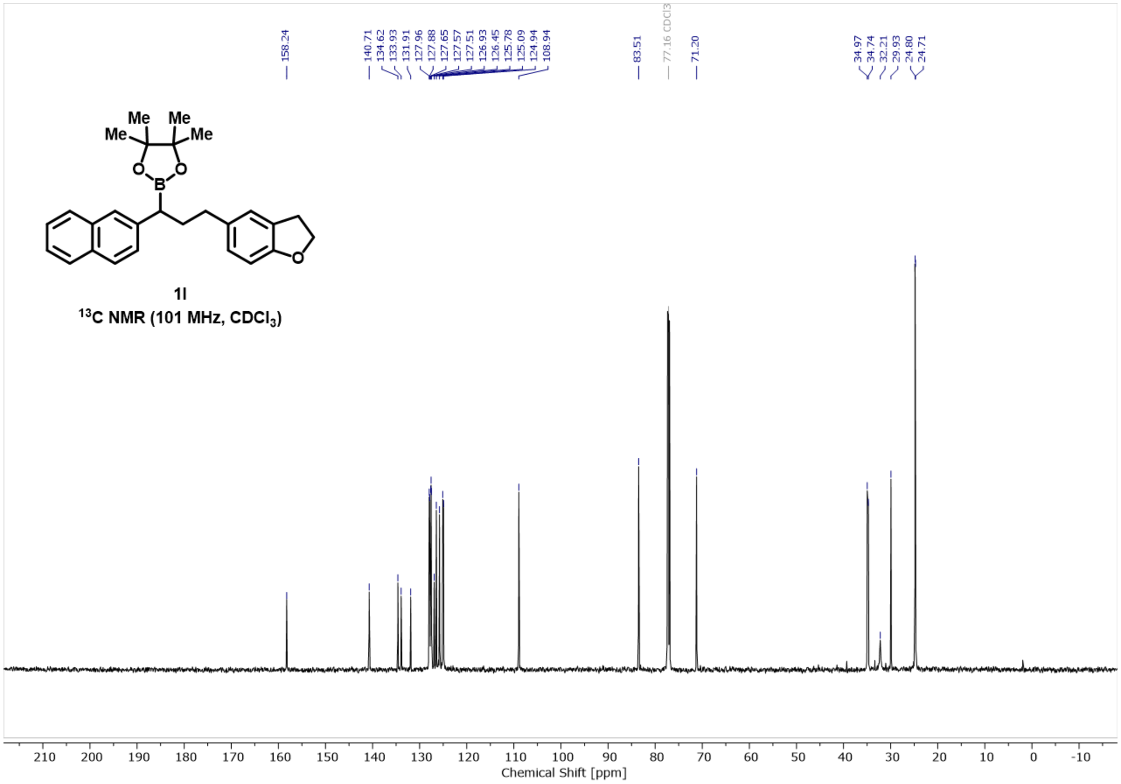

2-(1-(benzo[*b*]thiophen-5-yl)butyl)-4,4,5,5-tetramethyl-1,3,2-dioxaborolane (**1m**)

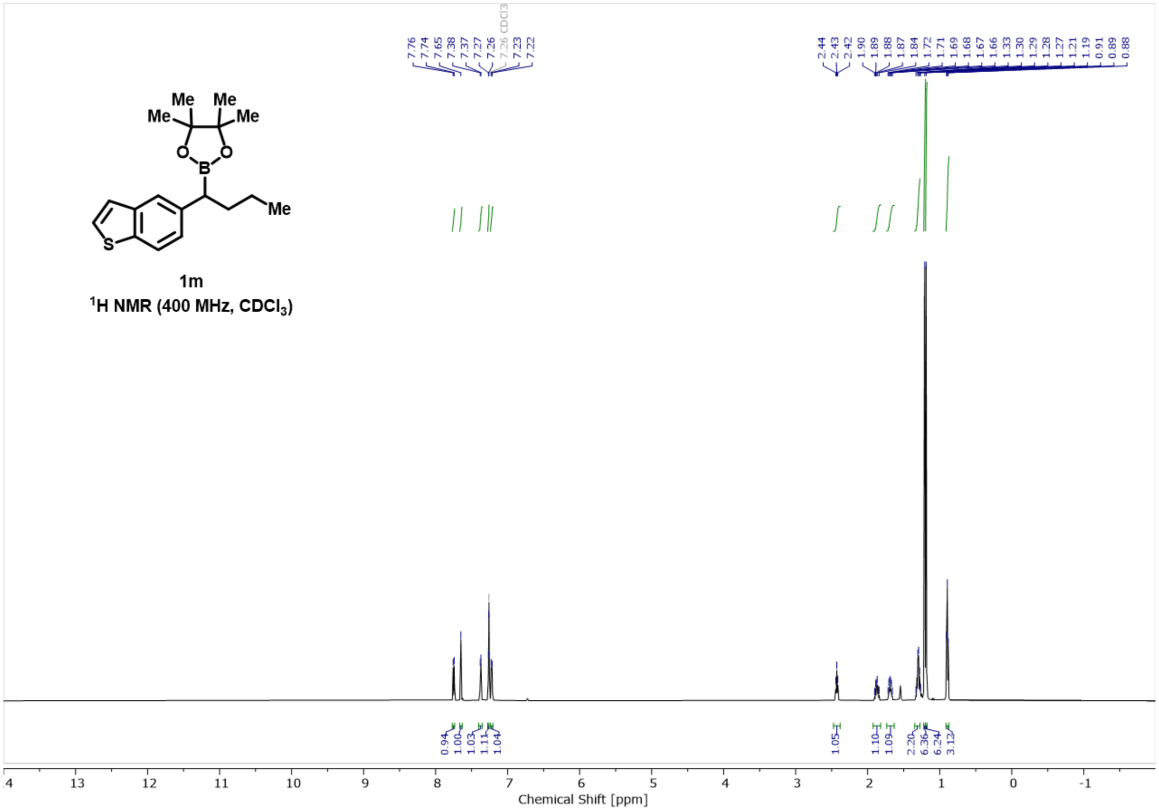


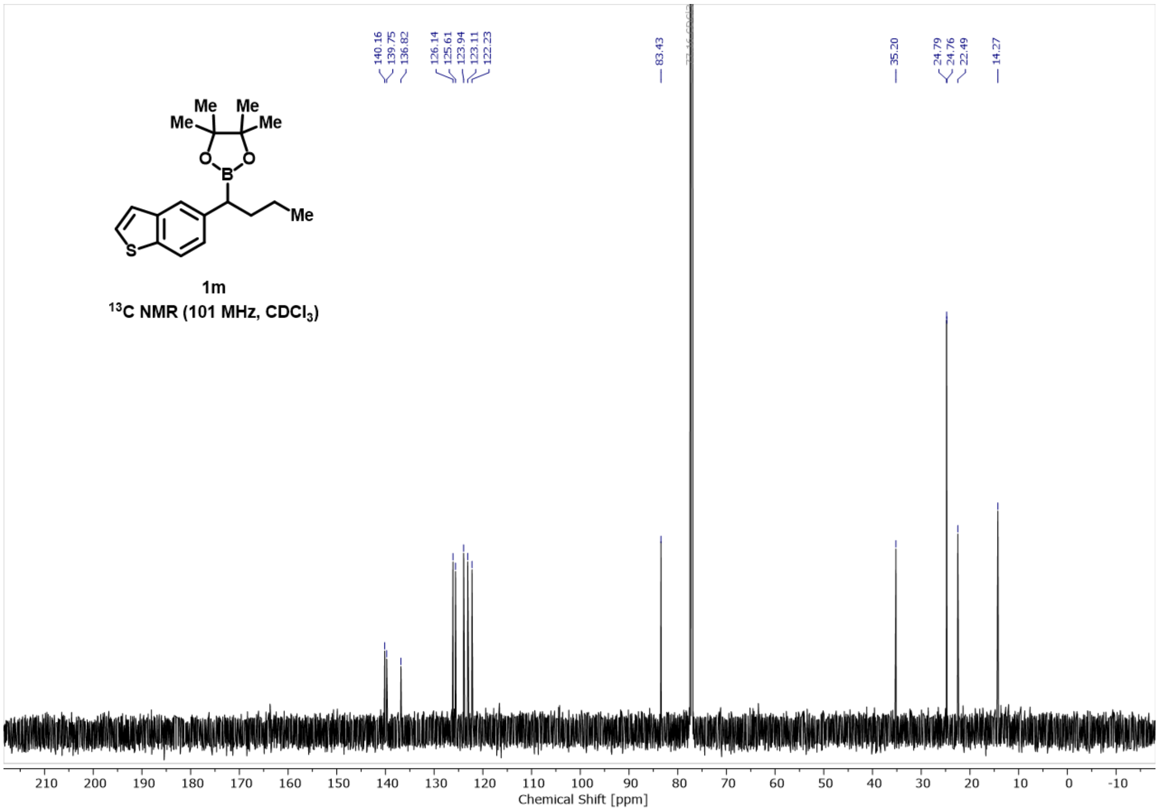

2-(1-(6-bromonaphthalen-2-yl)ethyl)-4,4,5,5-tetramethyl-1,3,2-dioxaborolane (**1n**)

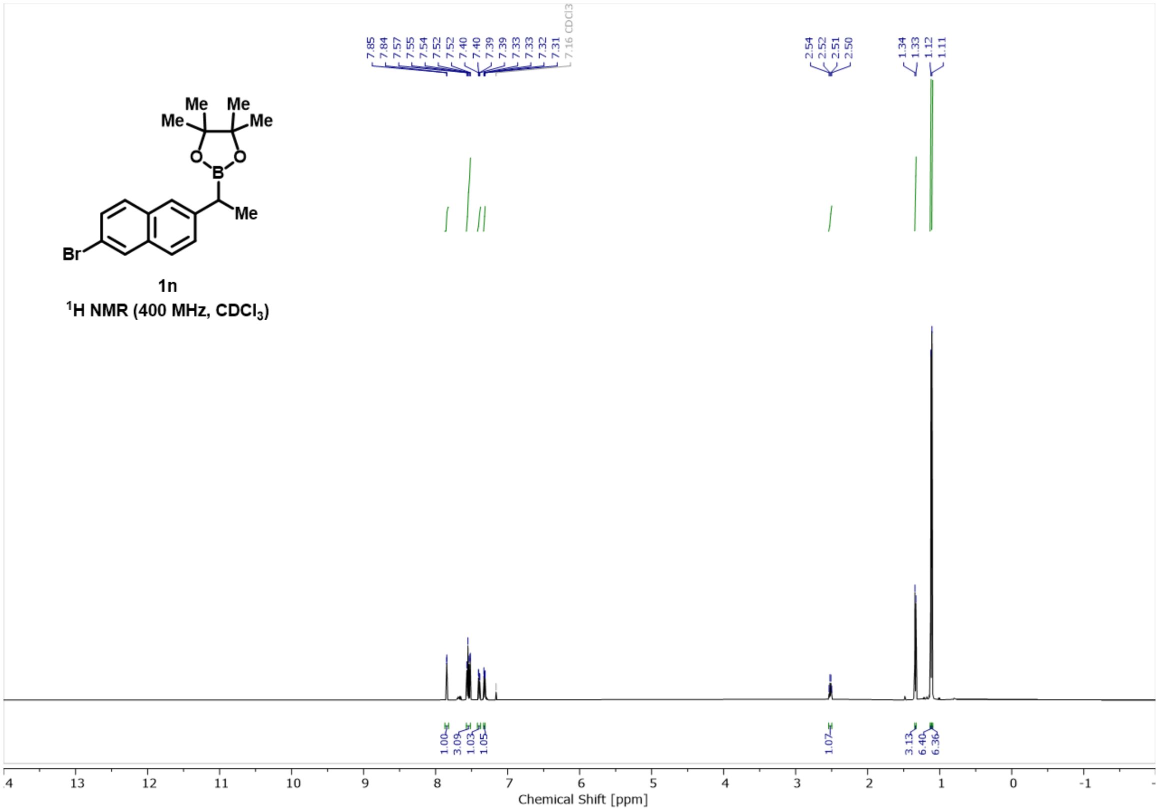


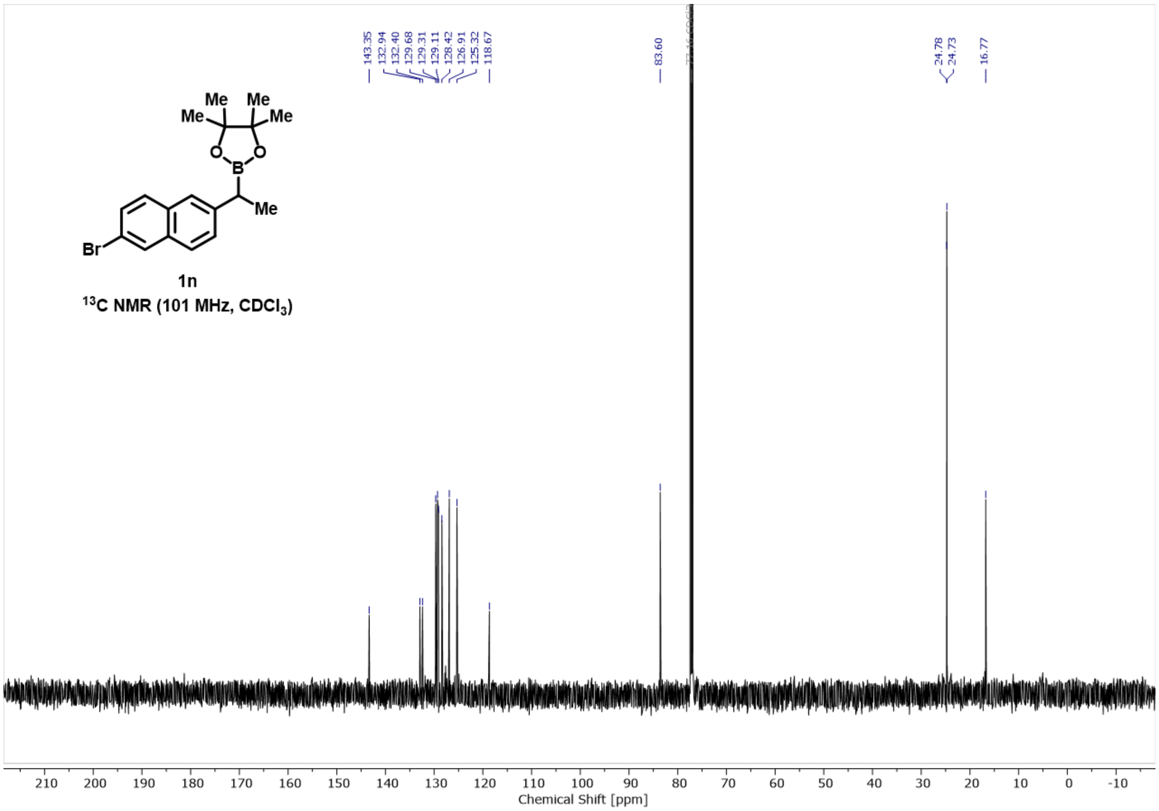

5-(cyclobutyl(4,4,5,5-tetramethyl-1,3,2-dioxaborolan-2-yl)methyl)-1-tosyl-1*H*-indole (**1o**)

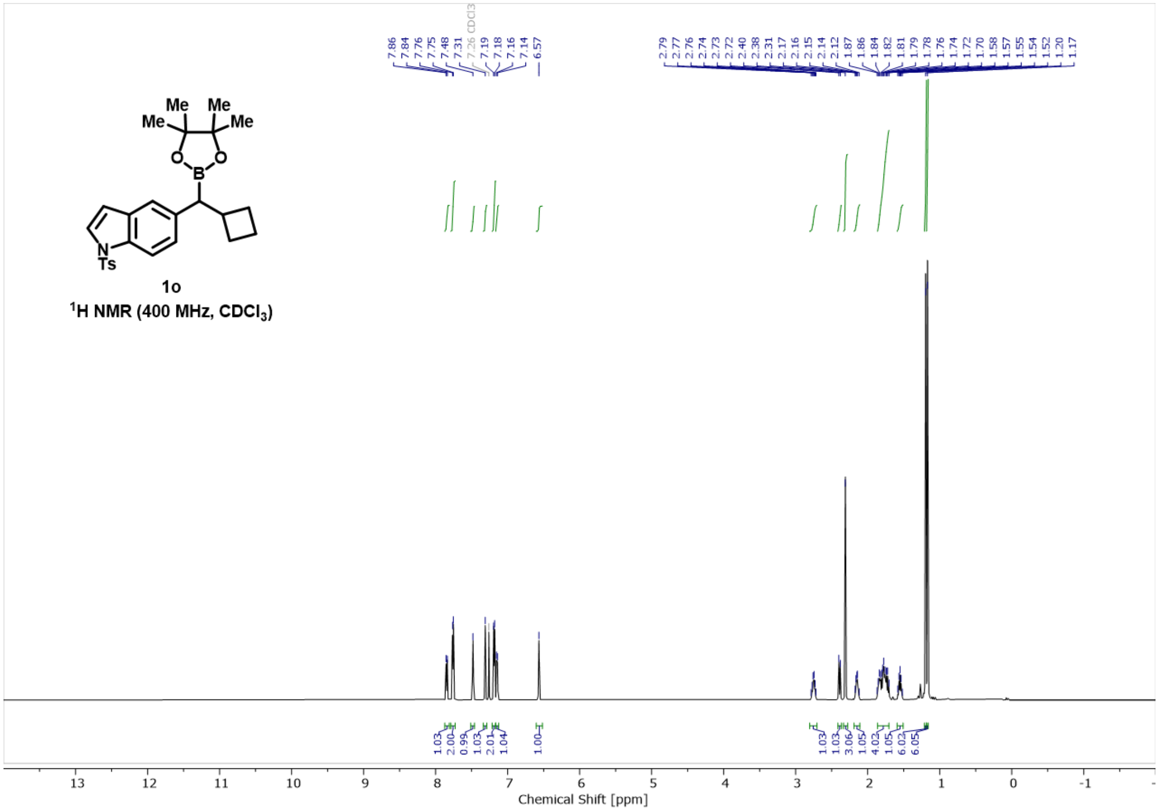


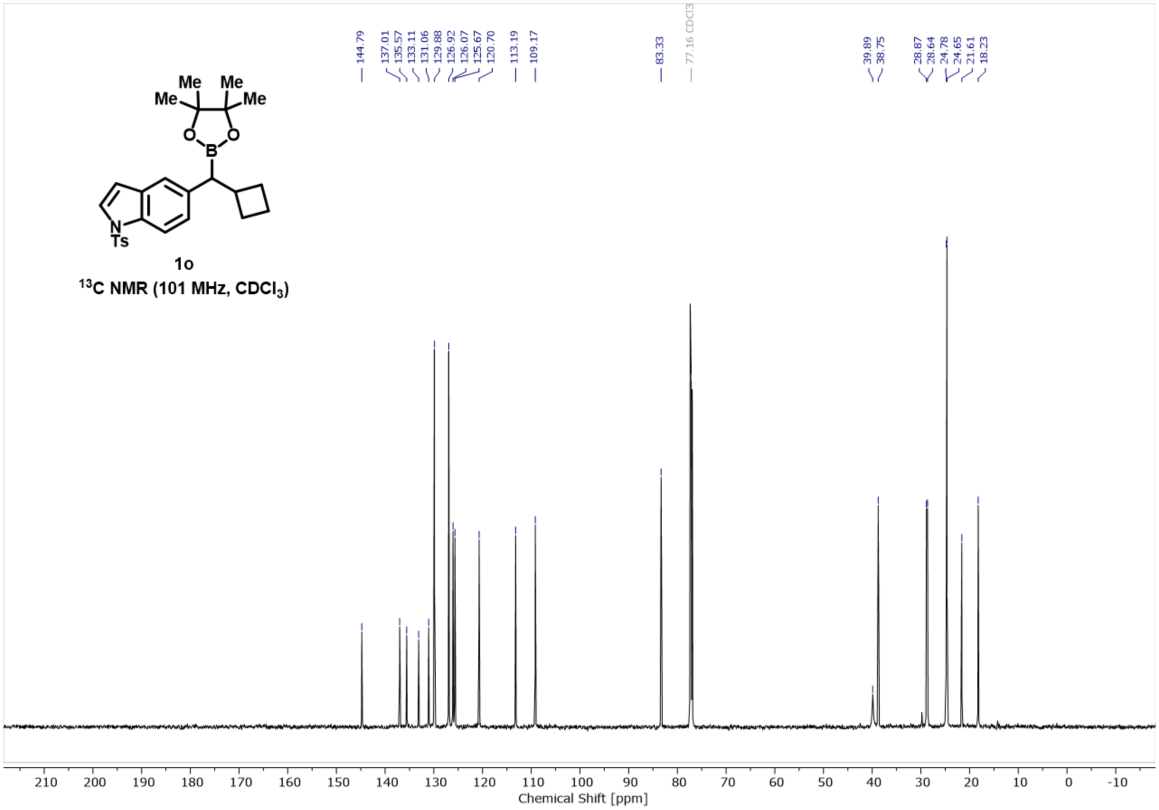

1-(3-(naphthalen-2-yl)-3-(4,4,5,5-tetramethyl-1,3,2-dioxaborolan-2-yl)propyl)-1*H*-pyrrole (**1p**)

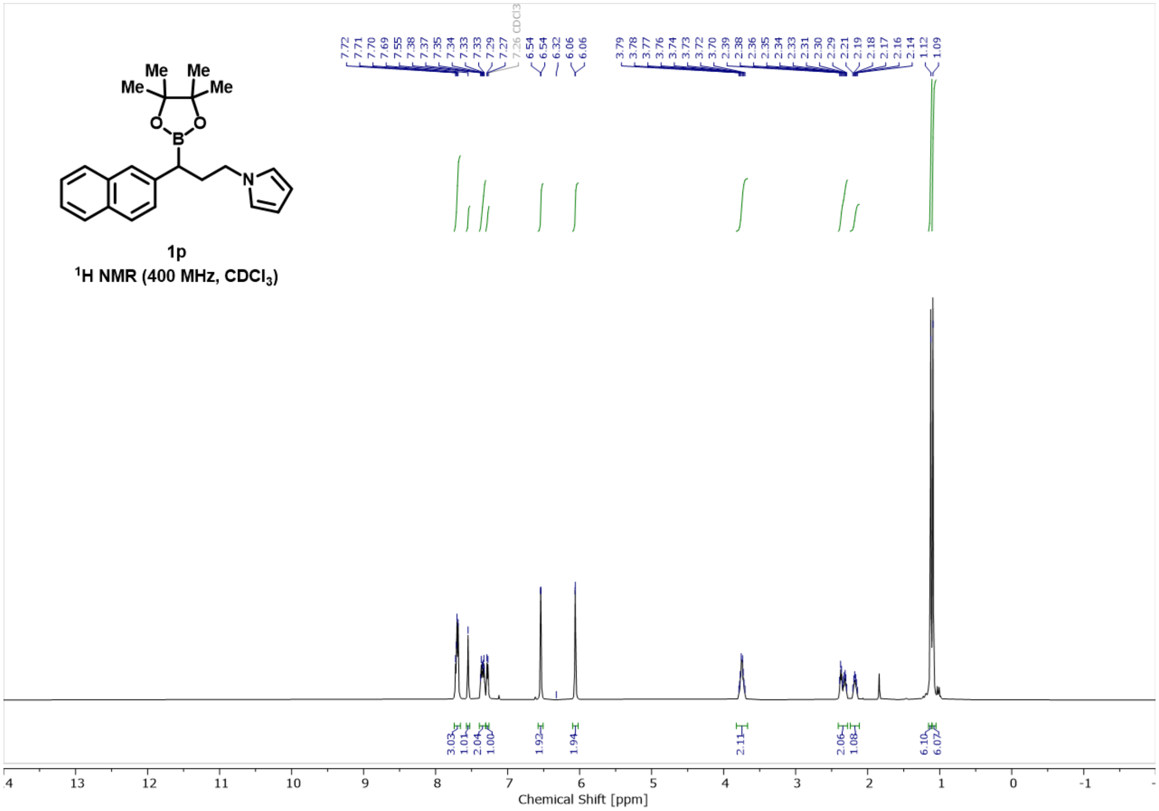


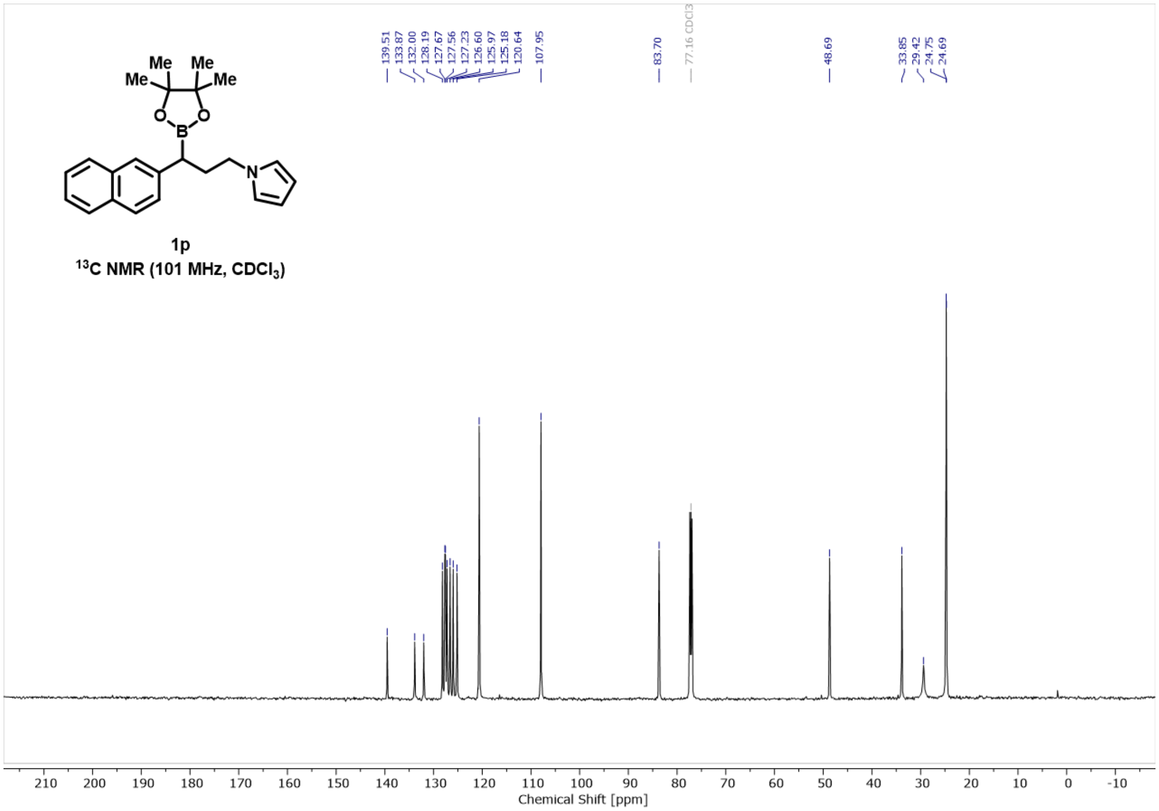

(*E*)-4,4,5,5-tetramethyl-2-(1-phenyloct-1-en-3-yl)-1,3,2-dioxaborolane (**1r**)

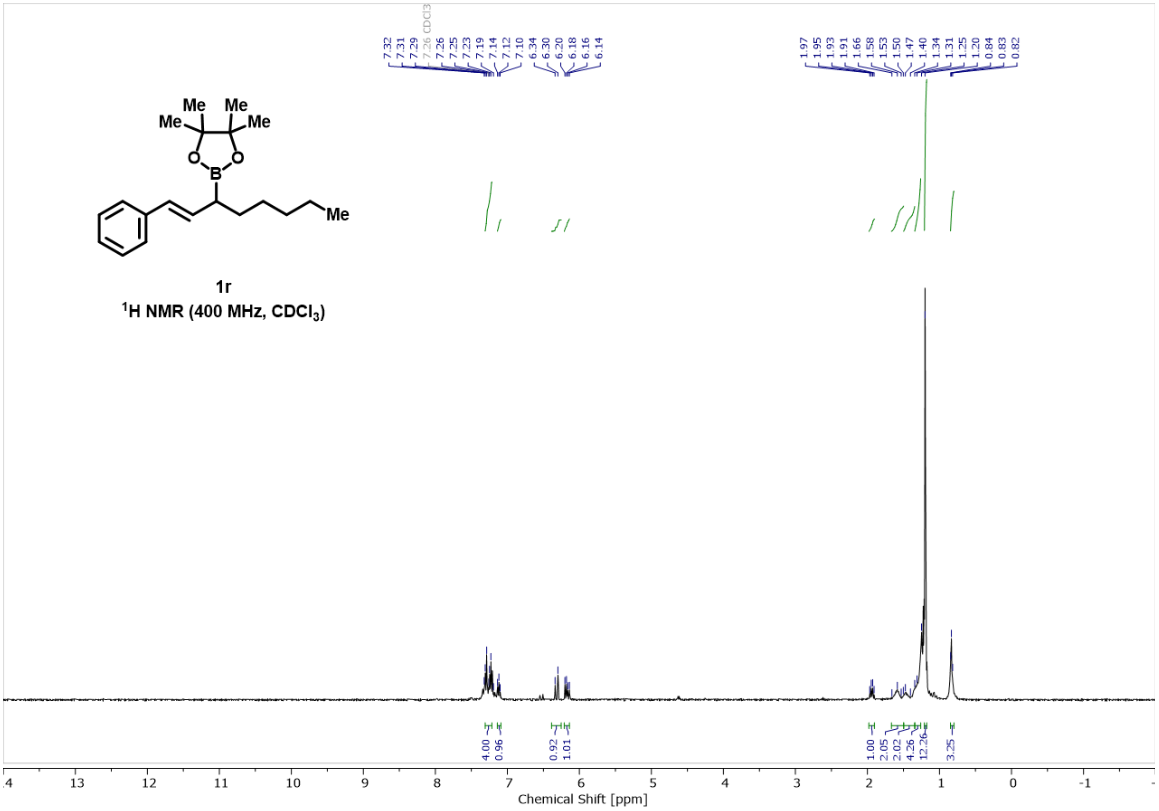


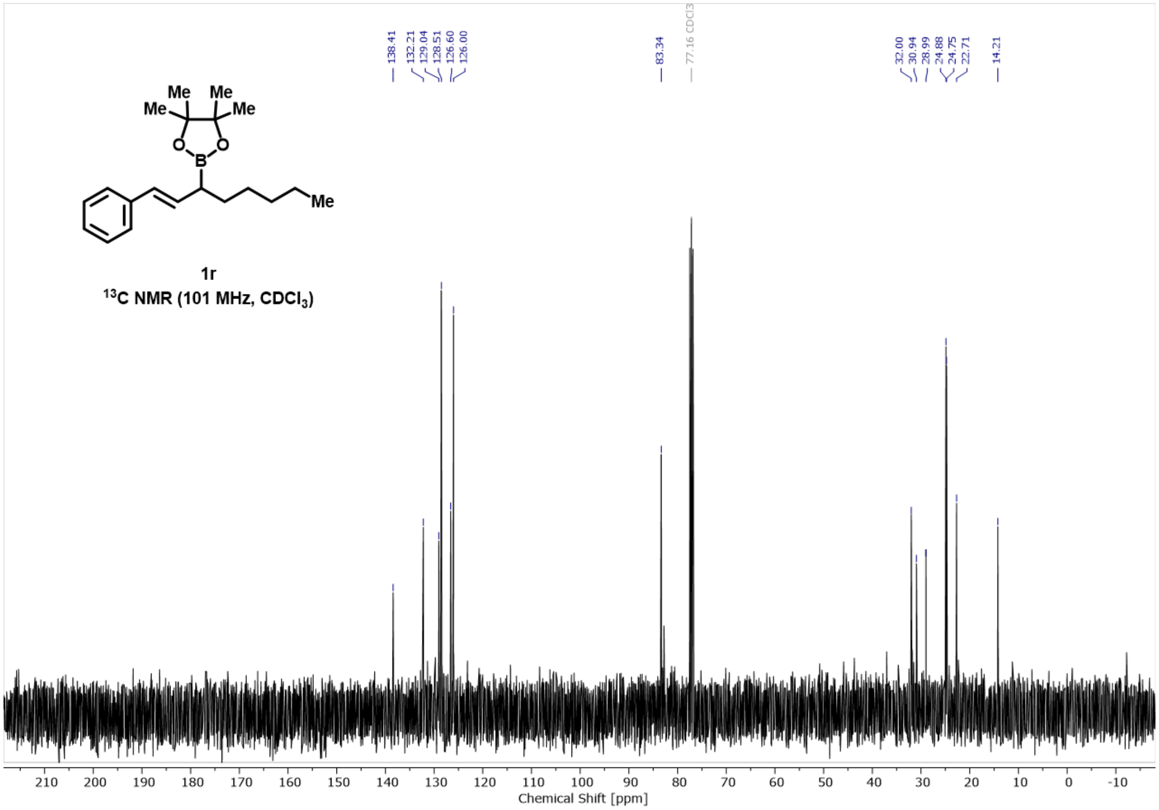

(*E*)-2-(5-(1,3-dioxan-2-yl)-1-phenylpent-1-en-3-yl)-4,4,5,5-tetramethyl-1,3,2-dioxaborolane (**1s**)

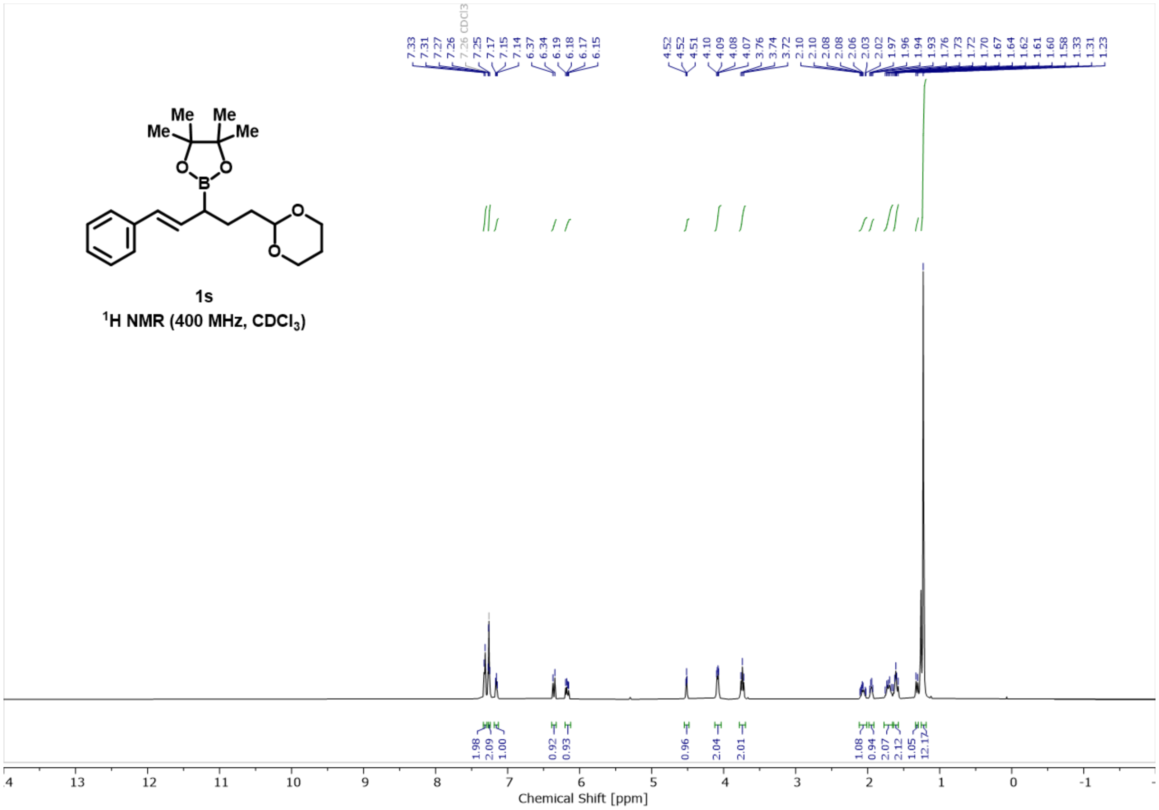


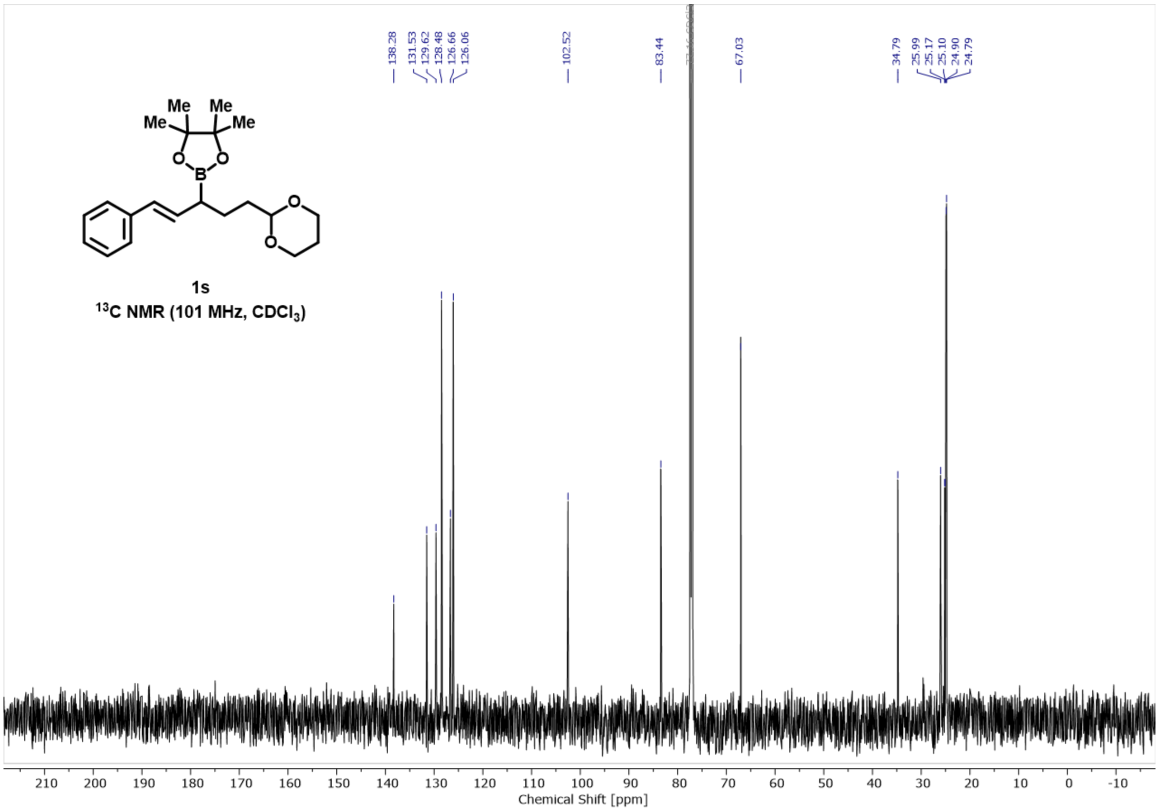

*NMR Data for Products*

(*S*)-1-(naphthalen-2-yl)ethyl benzoate (**3a**)

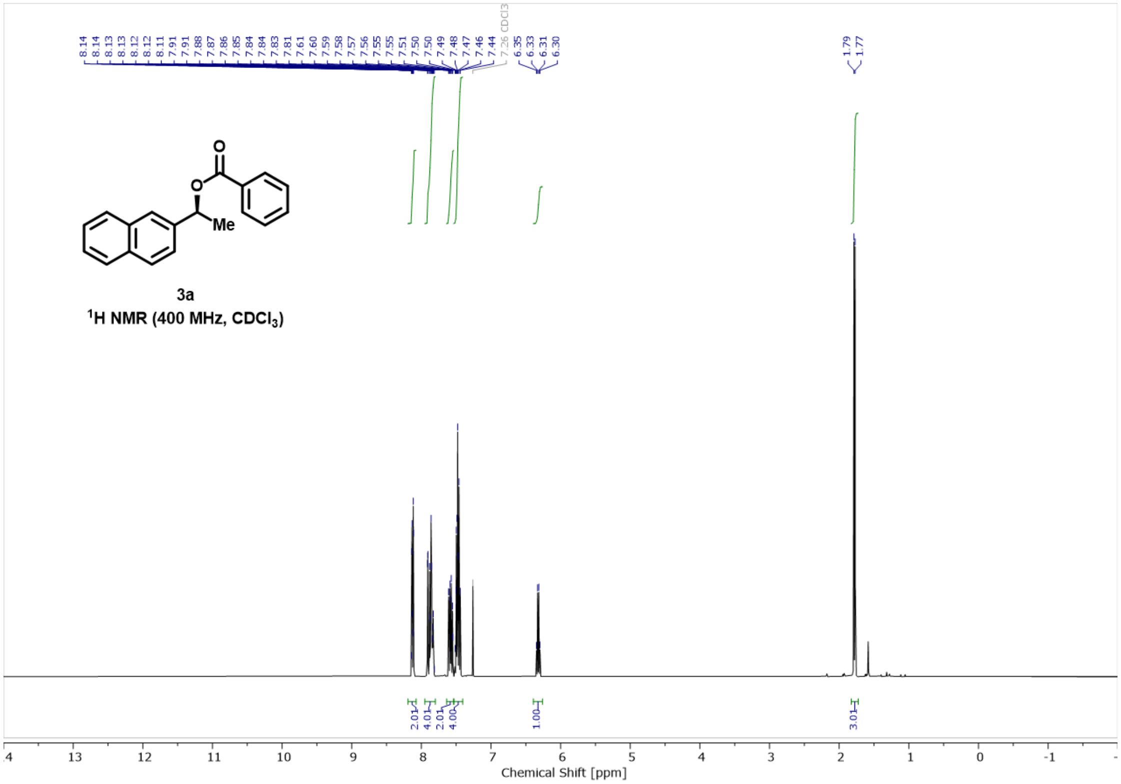


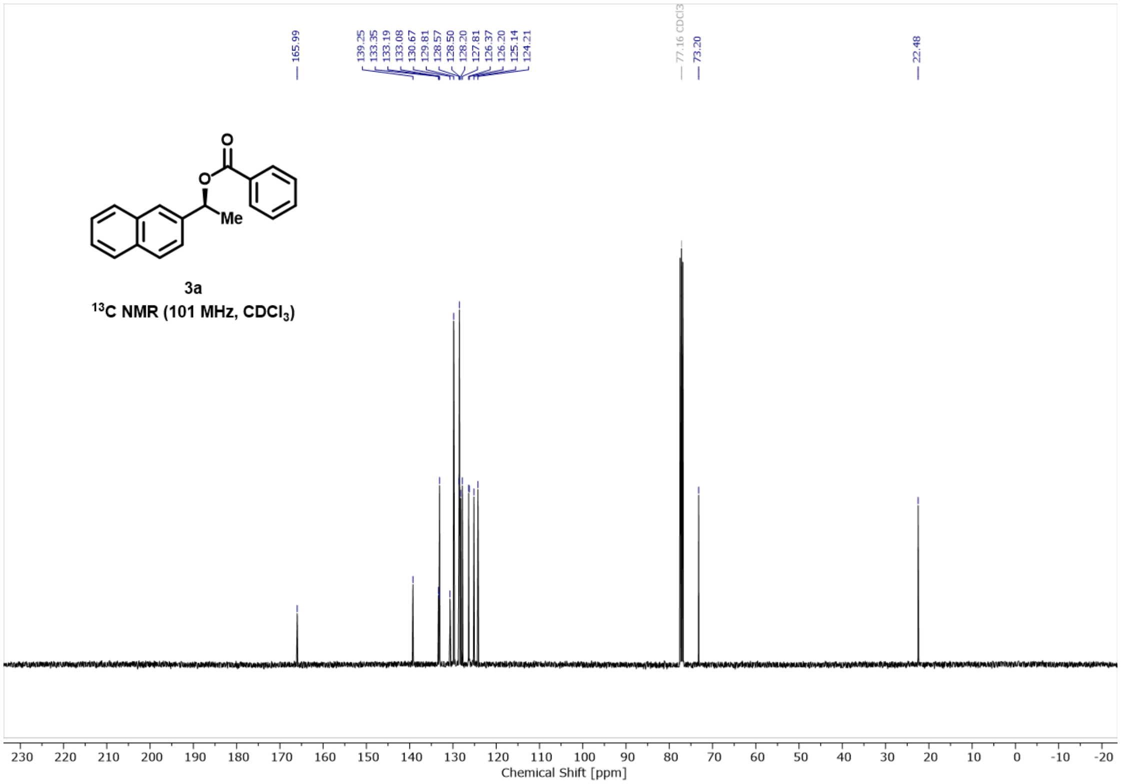

(*S*)-1-([1,1'-biphenyl]-2-yl)ethyl (3*S*,5*S*,7*S*)-adamantane-1-carboxylate (**3b**)

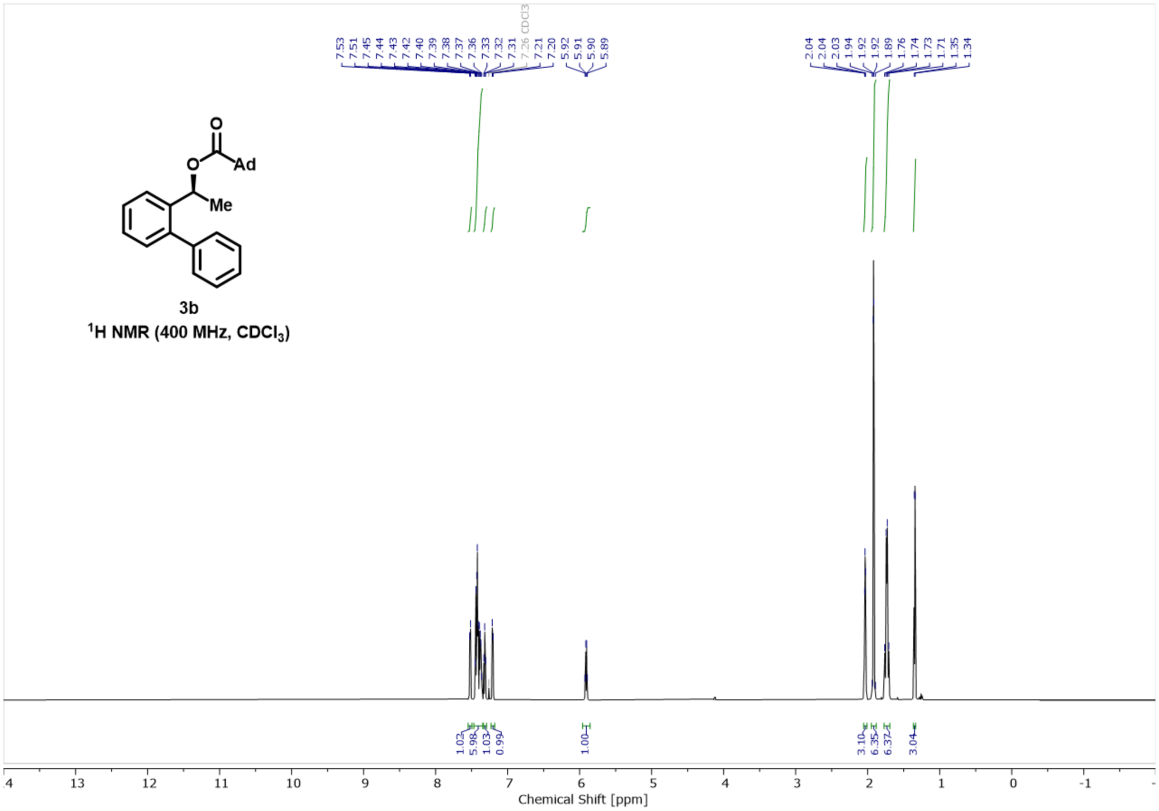

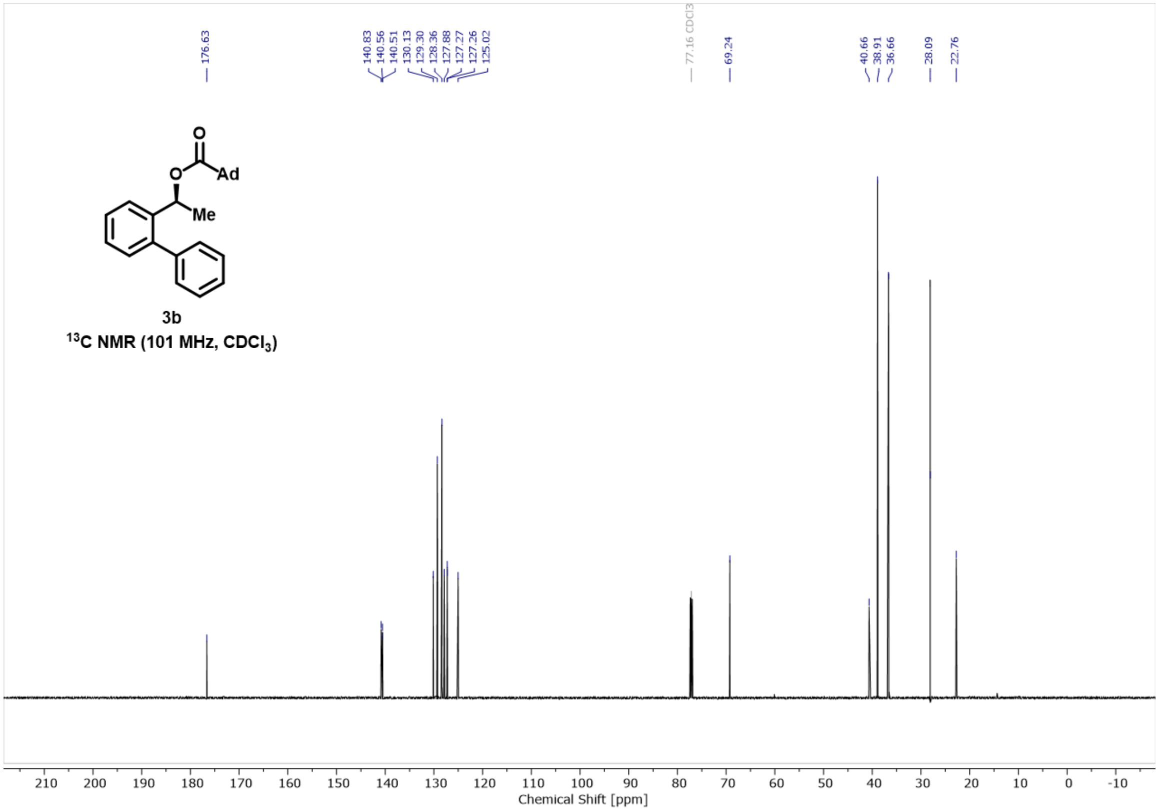

(*S*)-*tert*-butyl 3-((benzoyloxy)(naphthalen-2-yl)methyl)azetidine-1-carboxylate (**3c**)

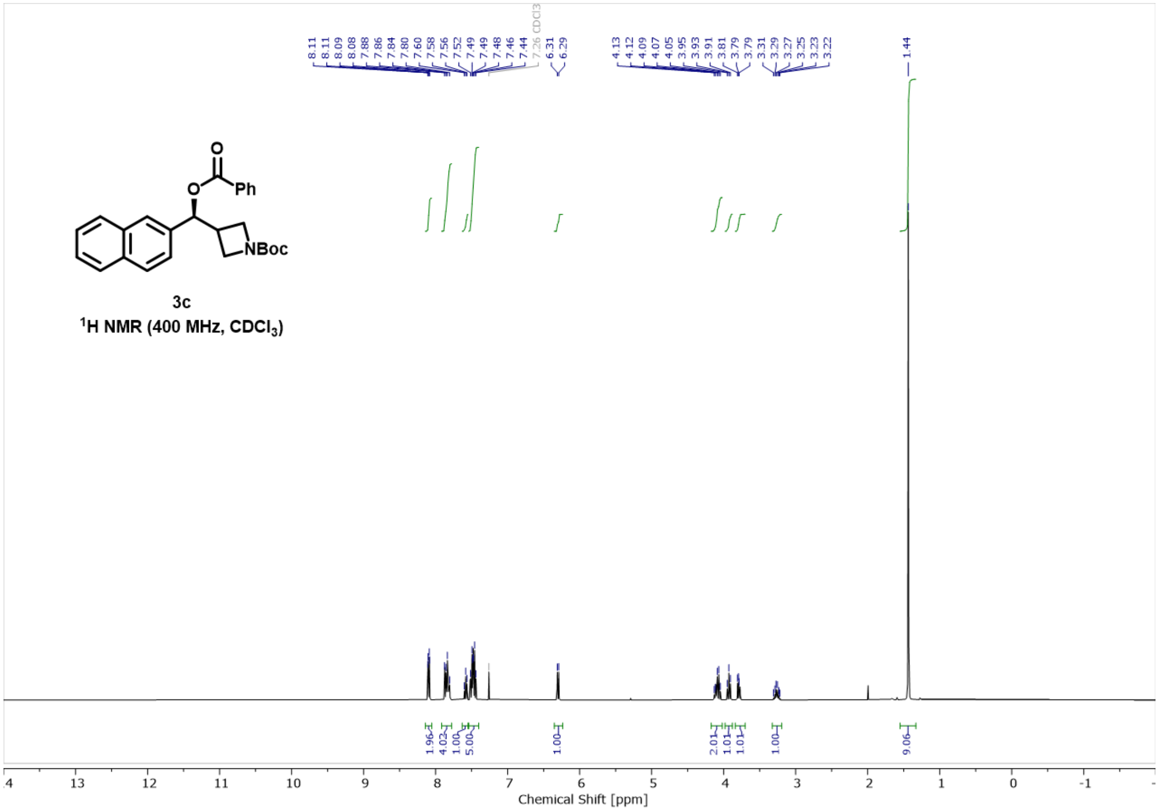


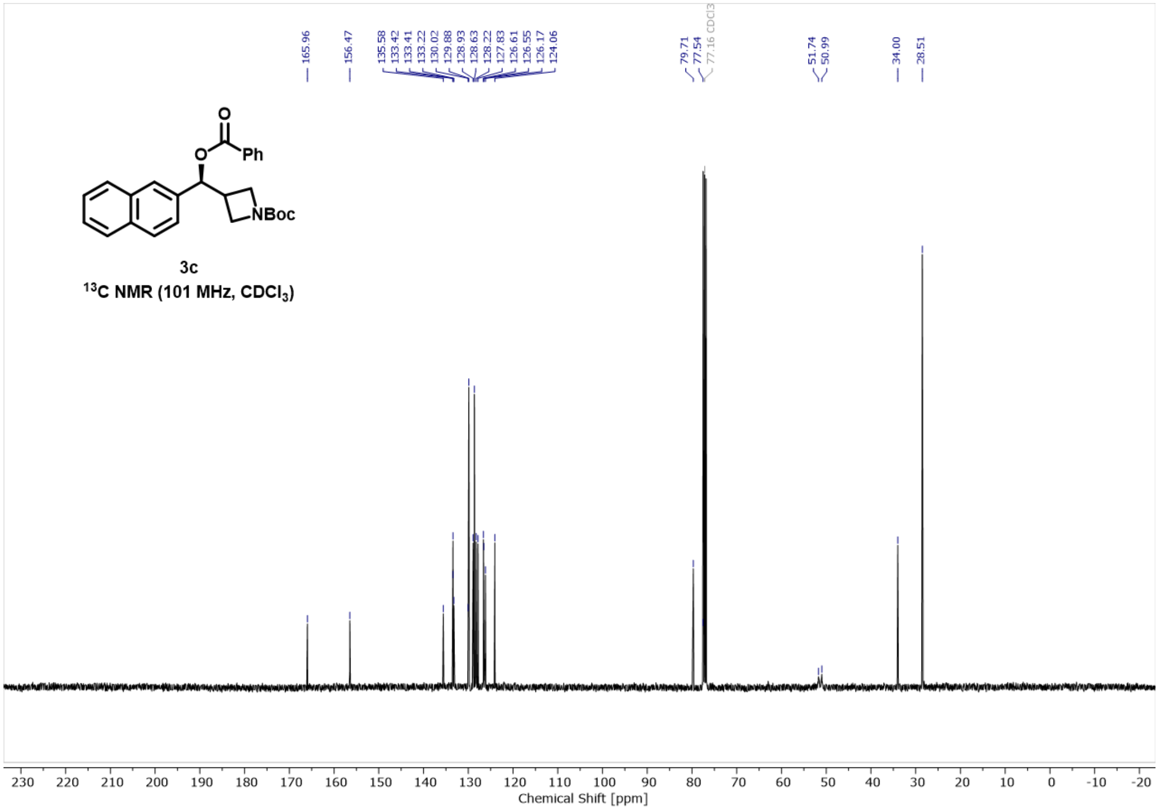

(*S*)-1-(2-phenylthiazol-4-yl)butyl benzoate (**3d**)

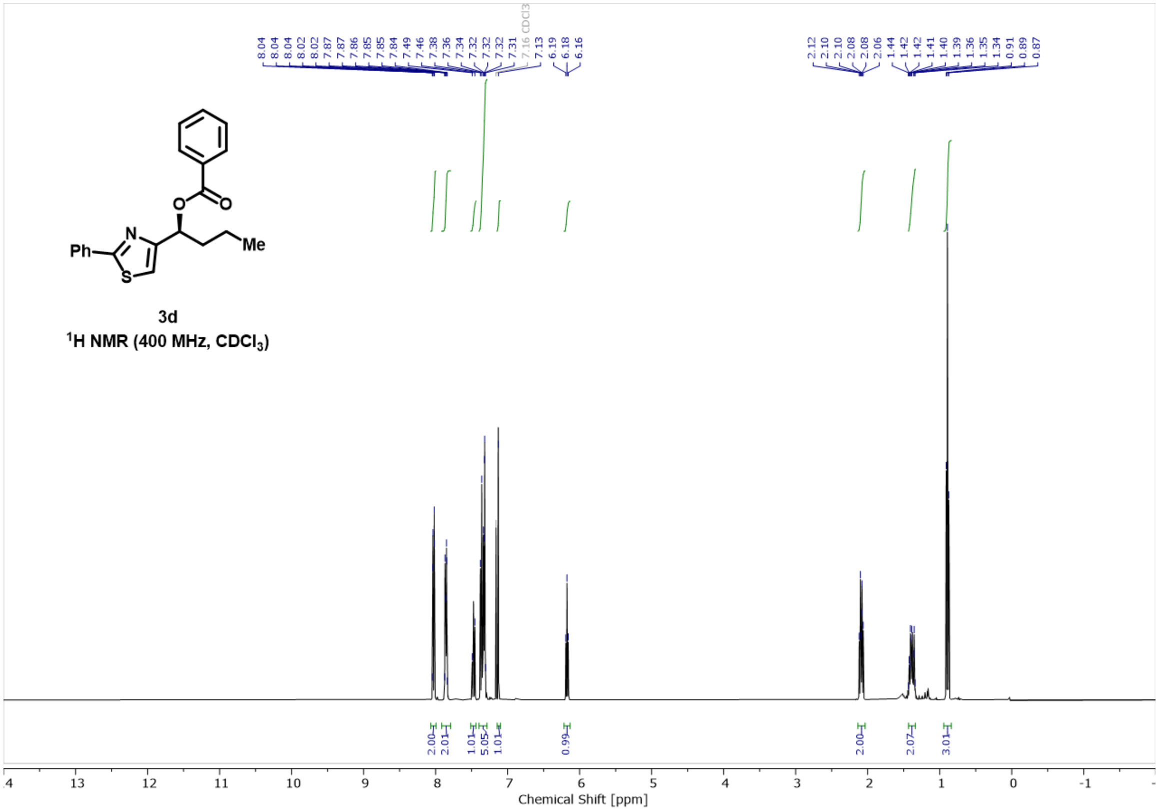


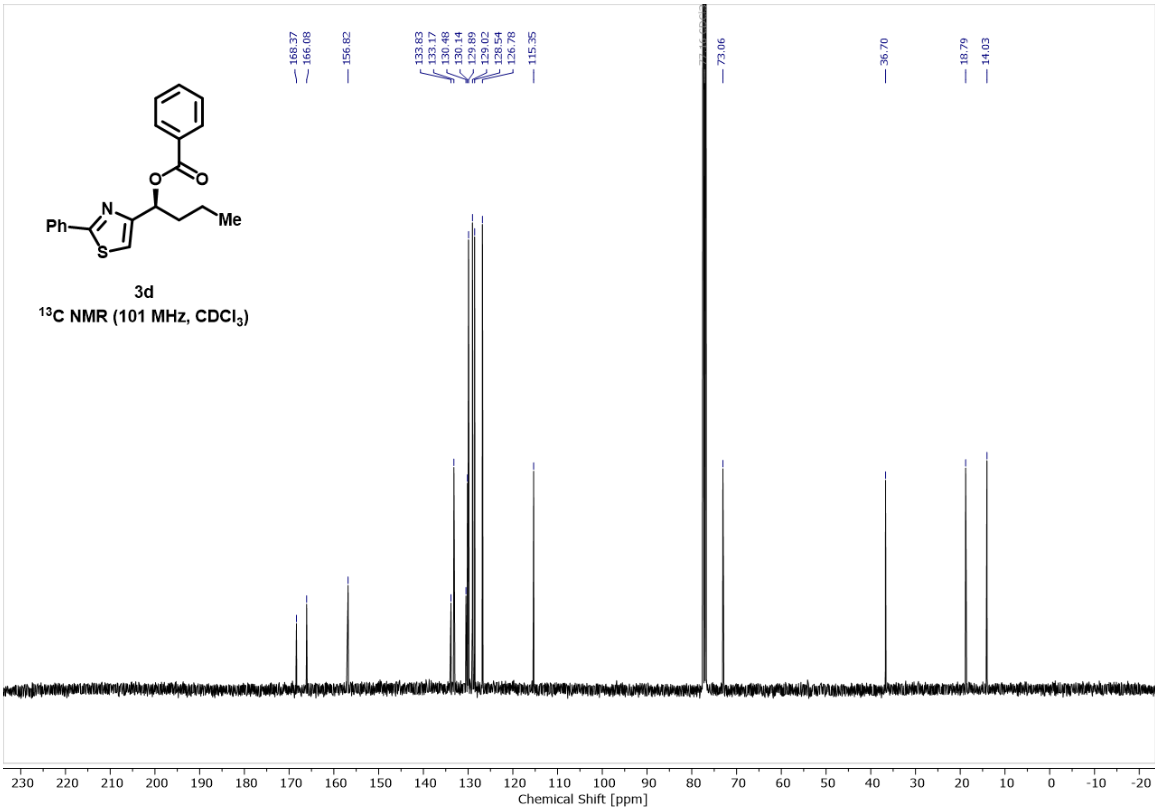

(*S*)-cyclobutyl(phenyl)methyl (3*S*,5*S*,7*S*)-adamantane-1-carboxylate (**3e**)

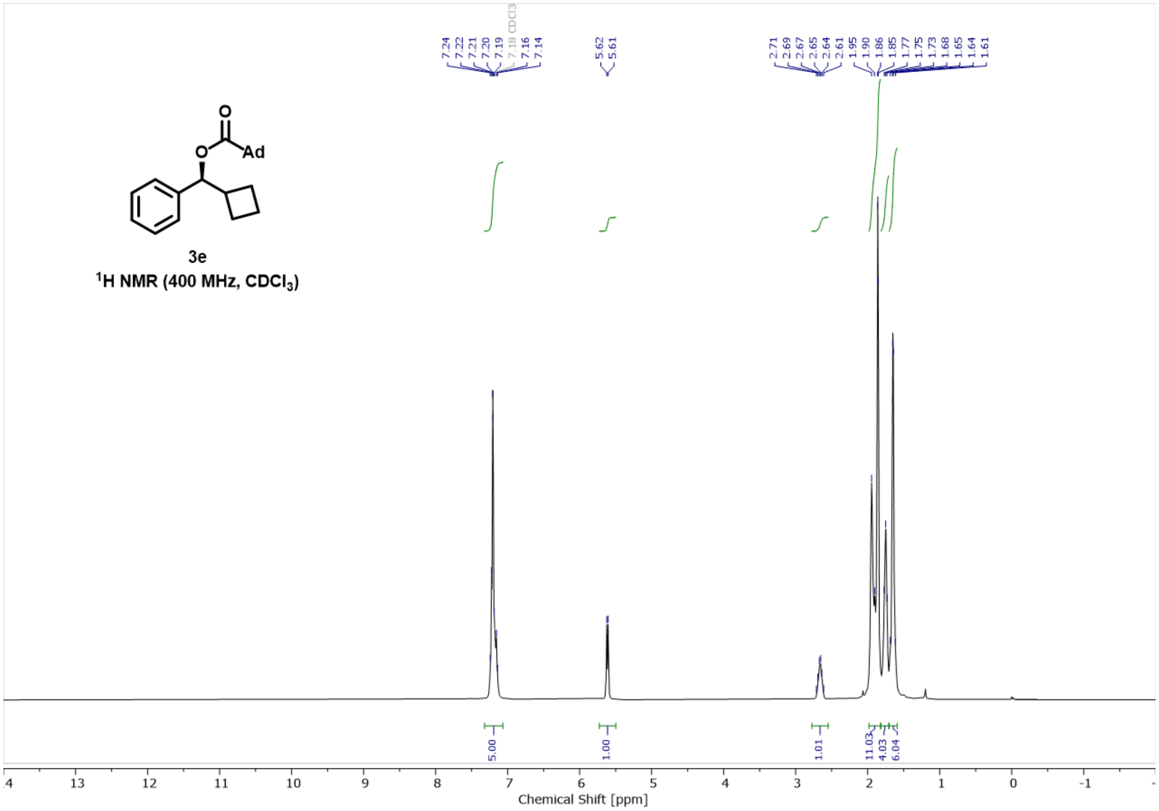


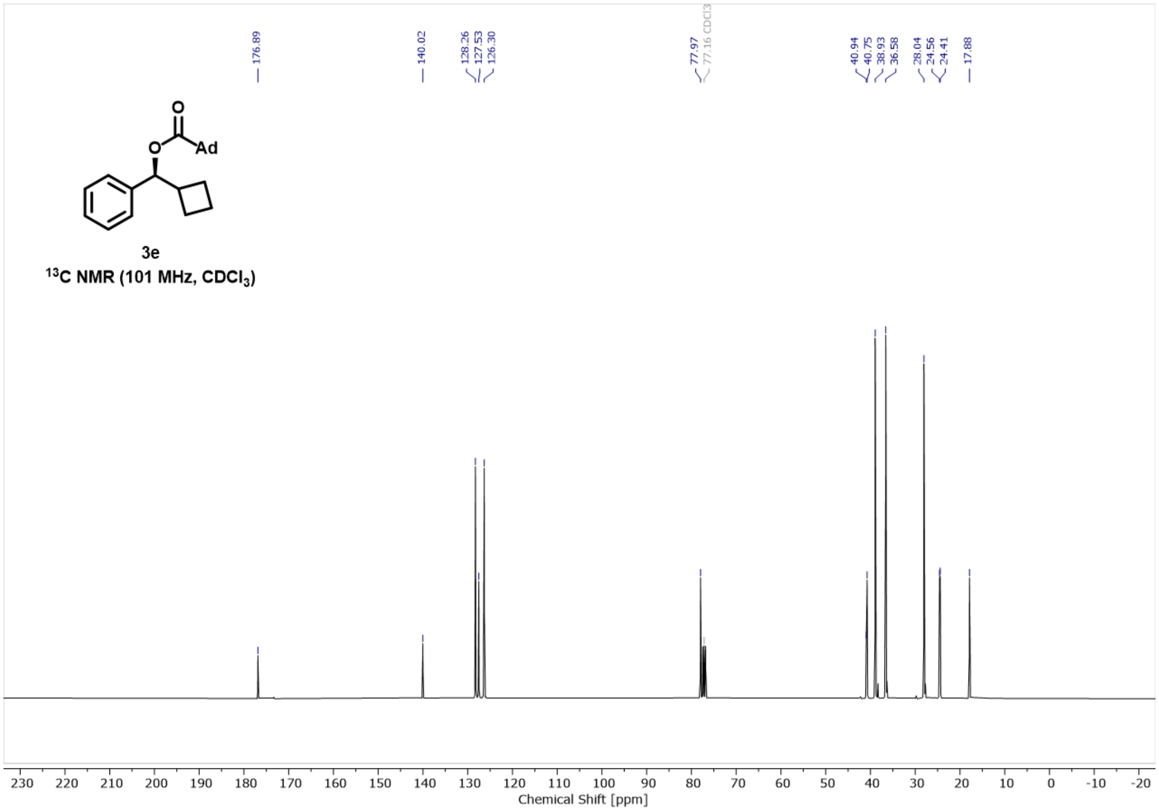

(*S*)-1-(benzo[*b*]thiophen-5-yl)-3-phenylpropyl benzoate (**3f**)

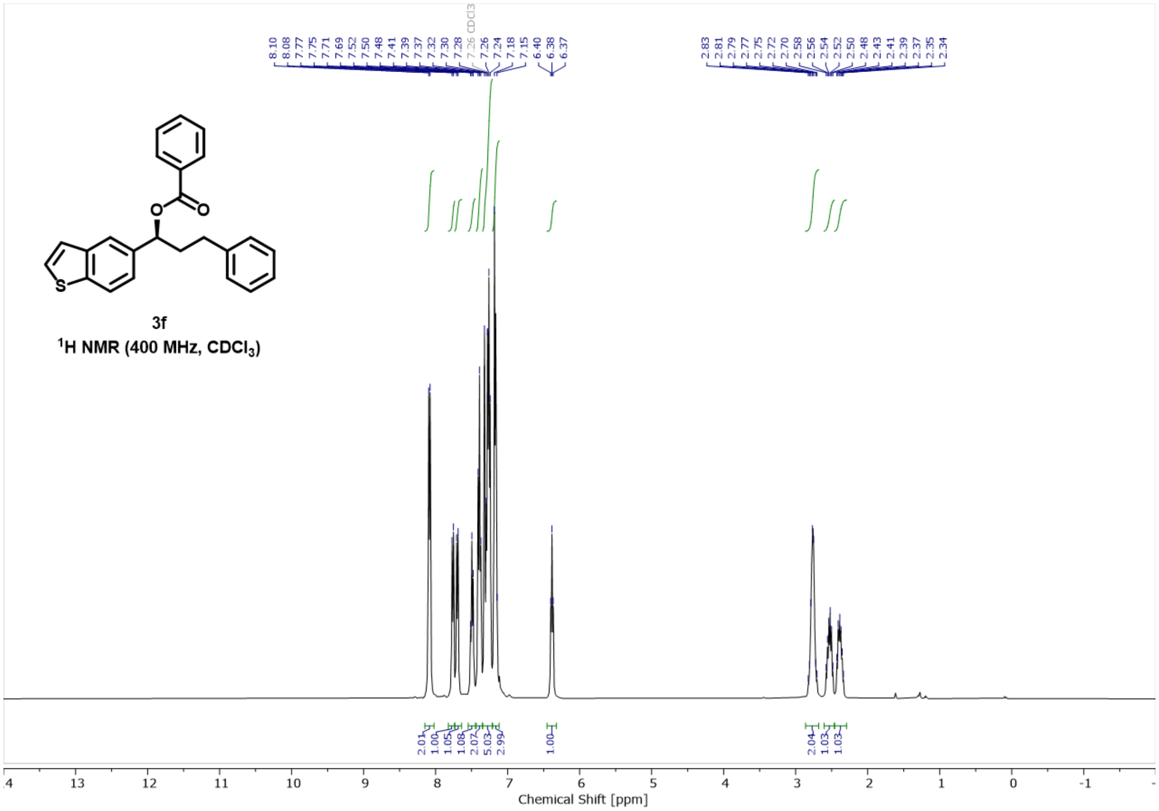


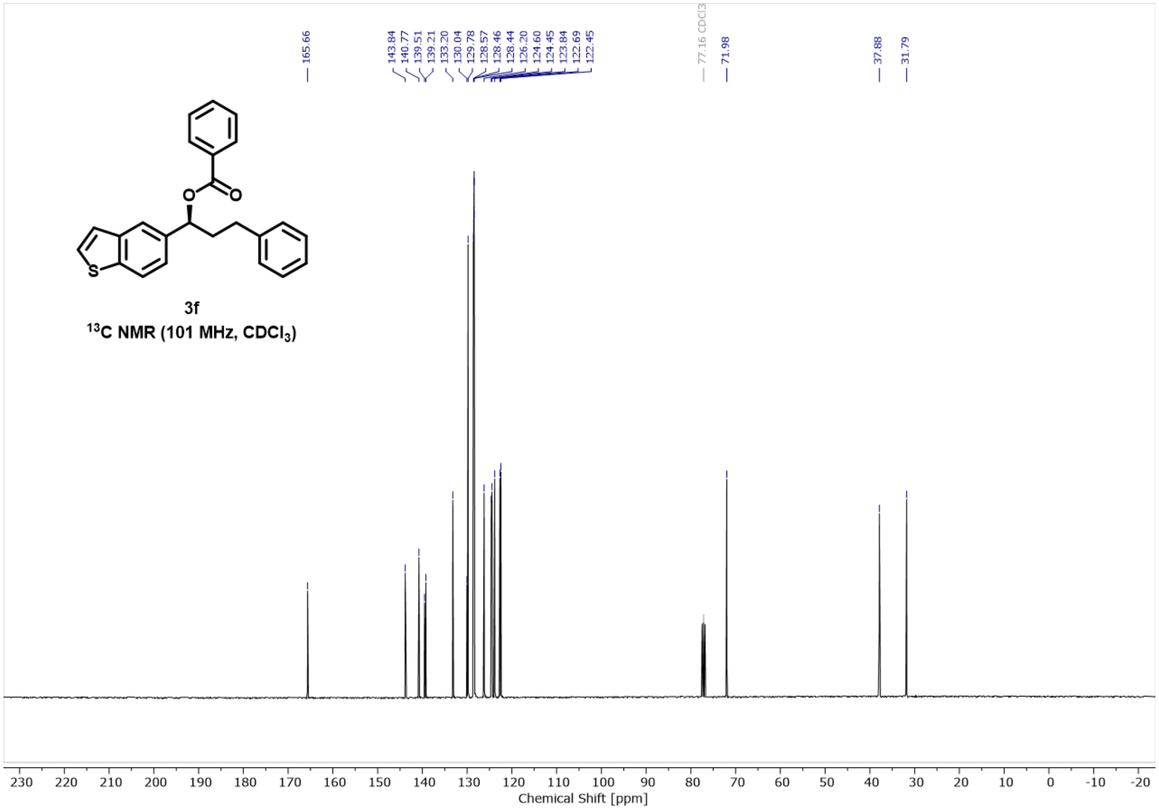

(*S*)-1-(benzofuran-5-yl)butyladamantane-1-carboxylate (**3g**)

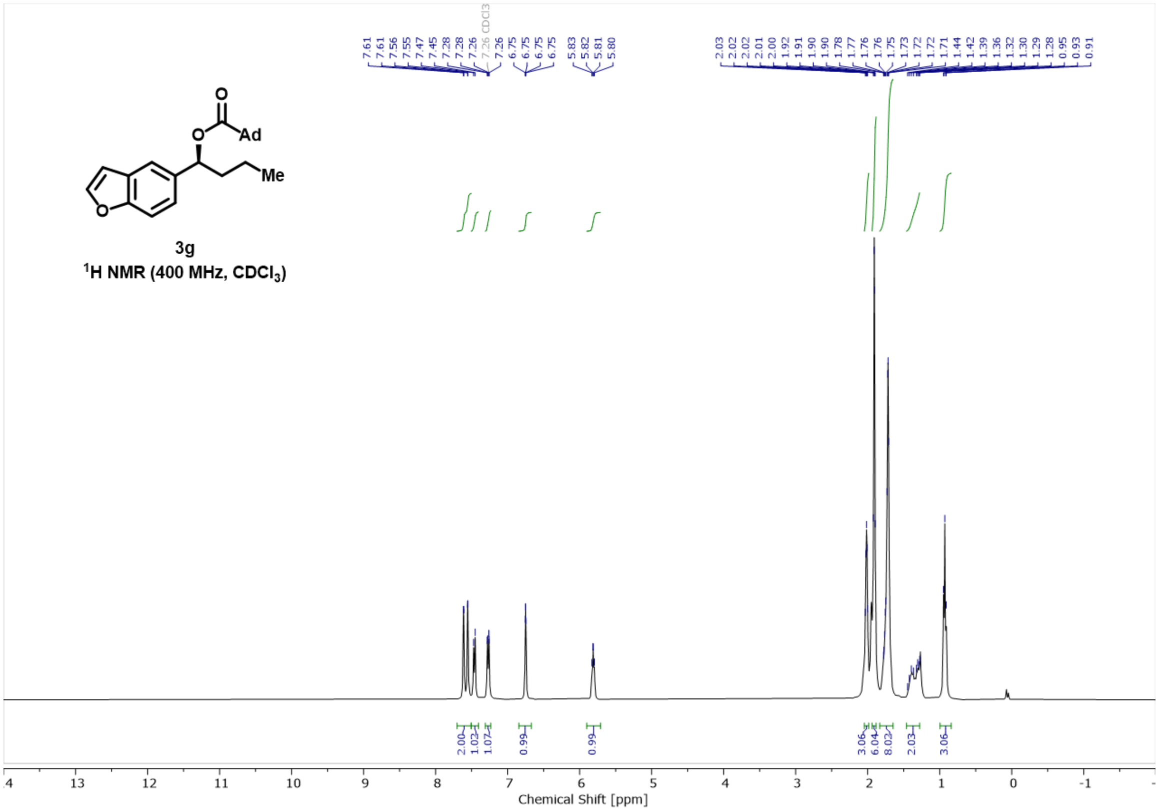


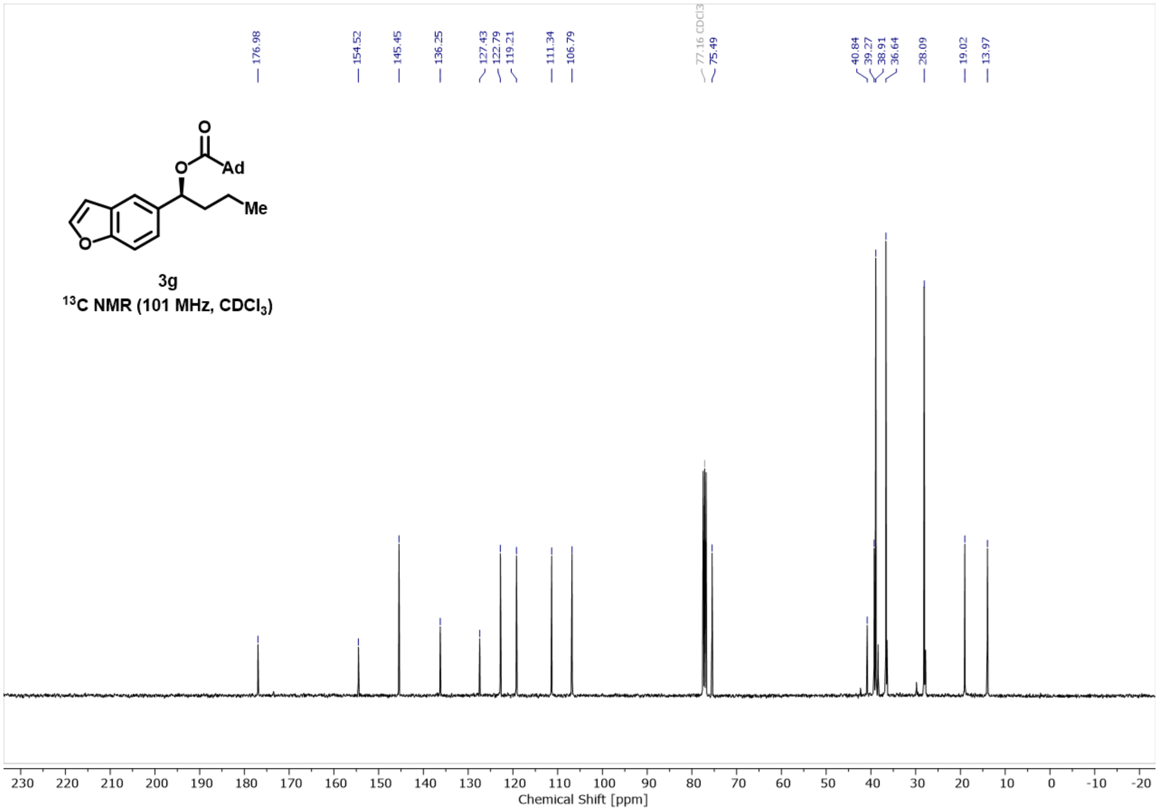

(*S*)-1-(naphthalen-2-yl)-2-(4-(4,4,5,5-tetramethyl-1,3,2-dioxaborolan-2-yl)phenyl)ethyl benzoate (**3h**)

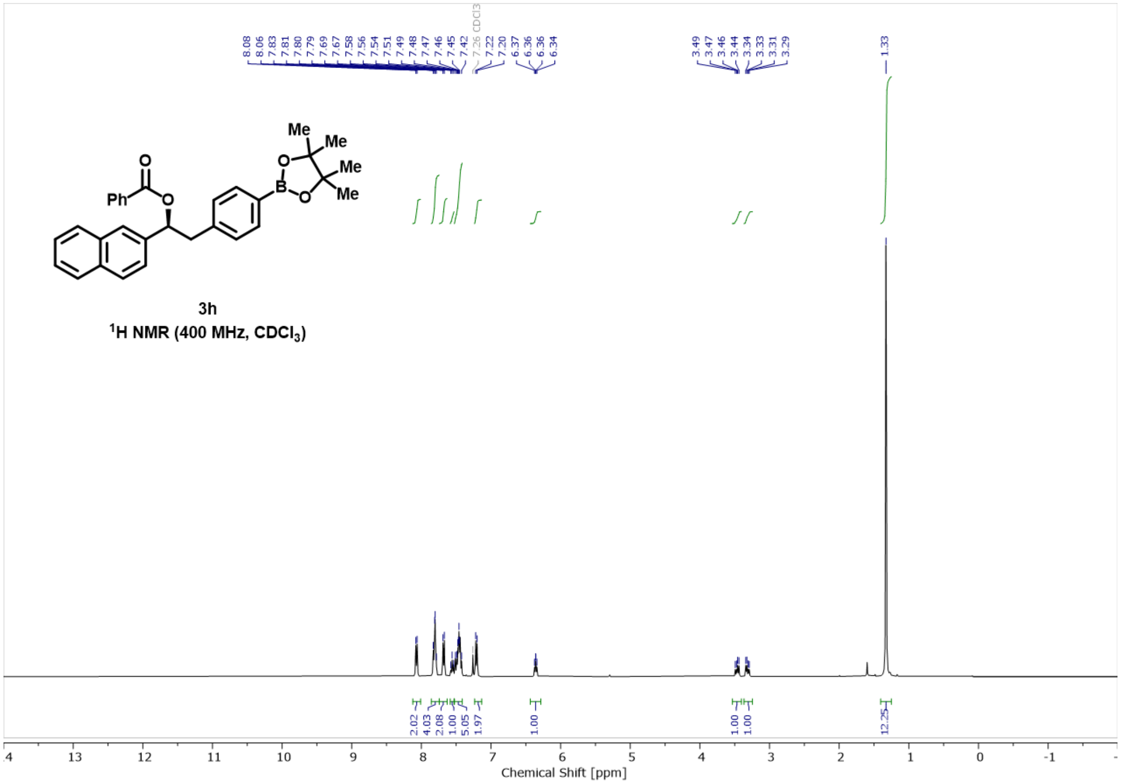


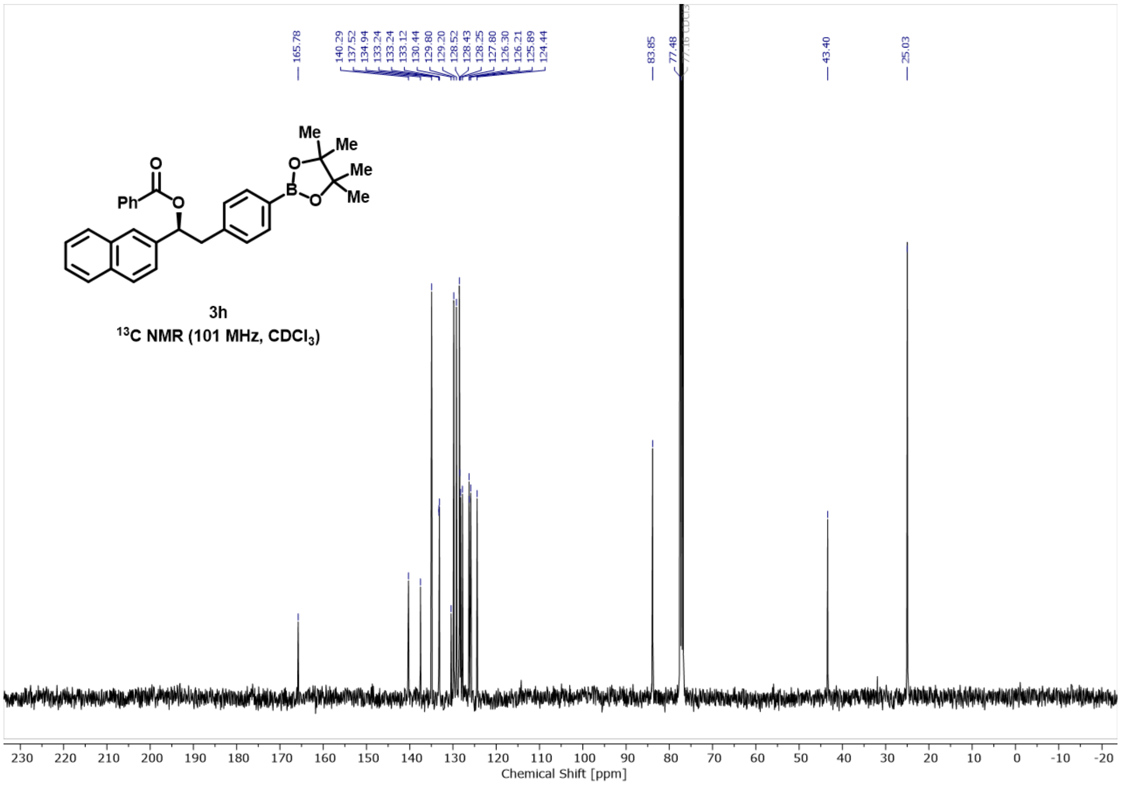

(*S*)-1-(naphthalen-2-yl)-4-(4,4,5,5-tetramethyl-1,3,2-dioxaborolan-2-yl)butyl benzoate (**3i**)

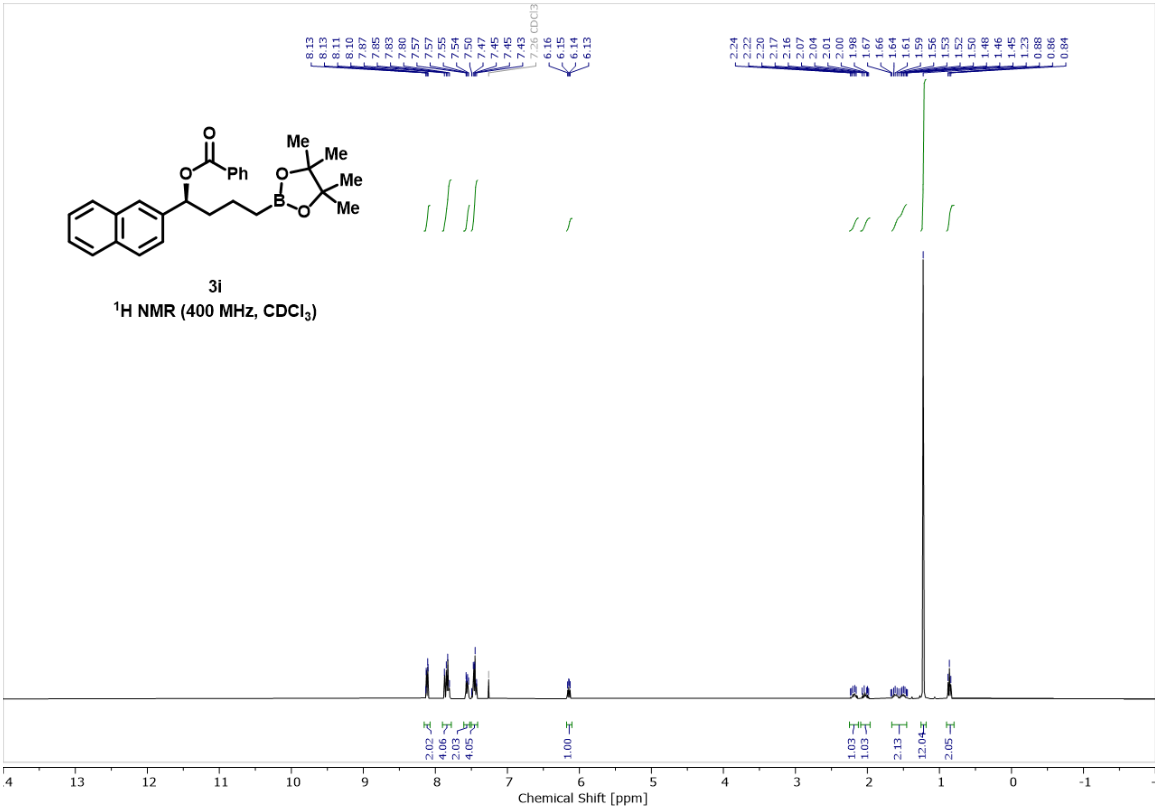


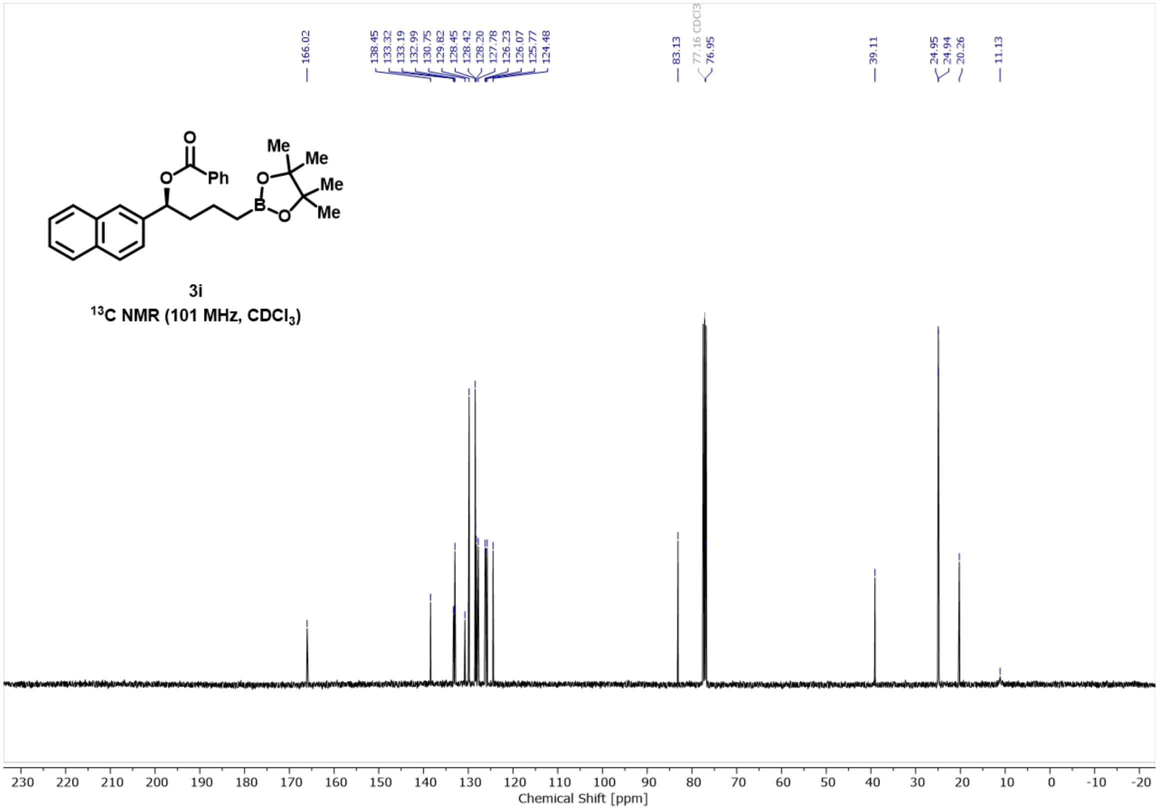

(*S*)-3-chloro-1-(naphthalen-2-yl)propyl benzoate (**3j**)

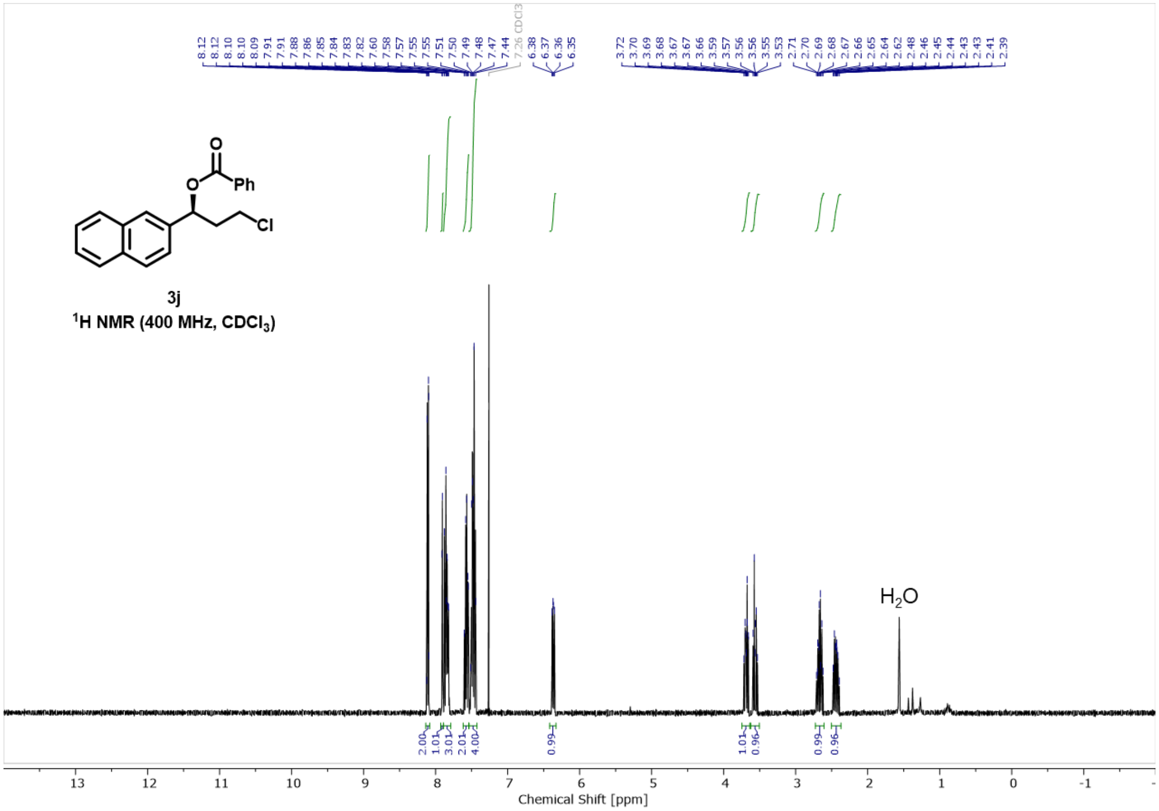


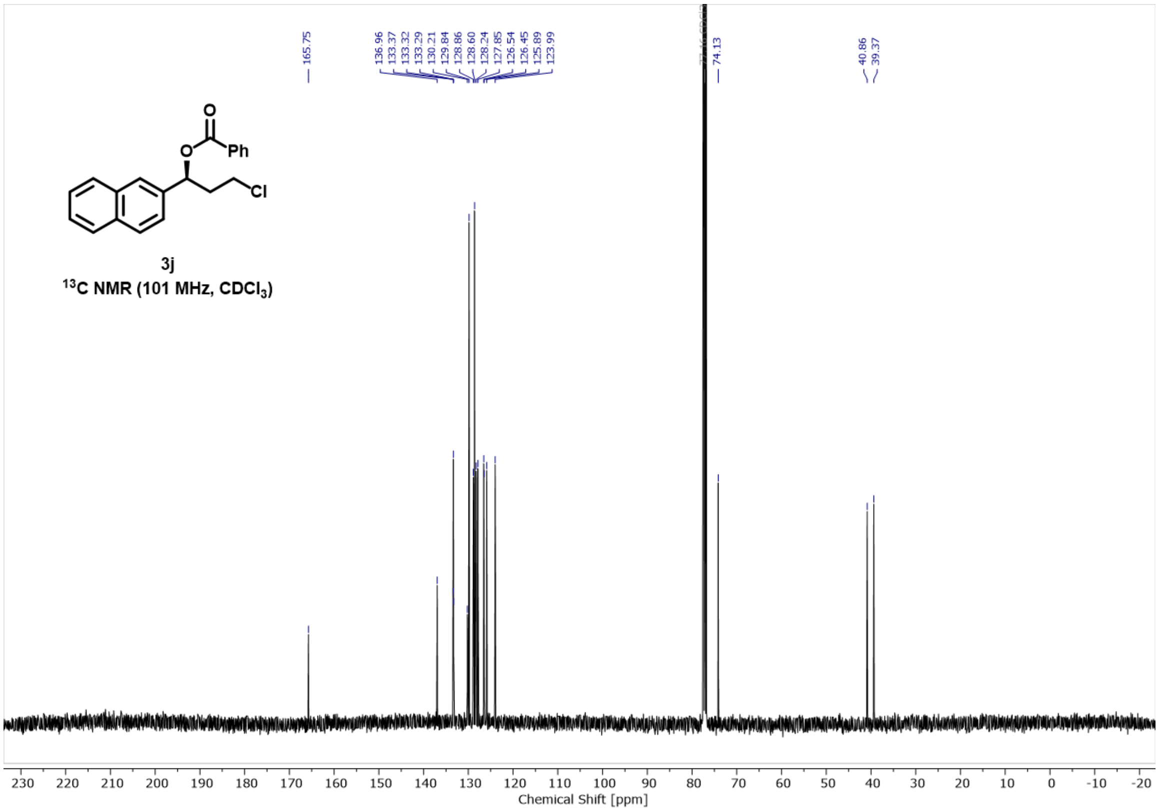

*tert*-butyl 3-((*S*)-(adamantane-1-carbonyl)oxy(benzofuran-5-yl)methyl) azetidine-1-carboxylate (**3k**)

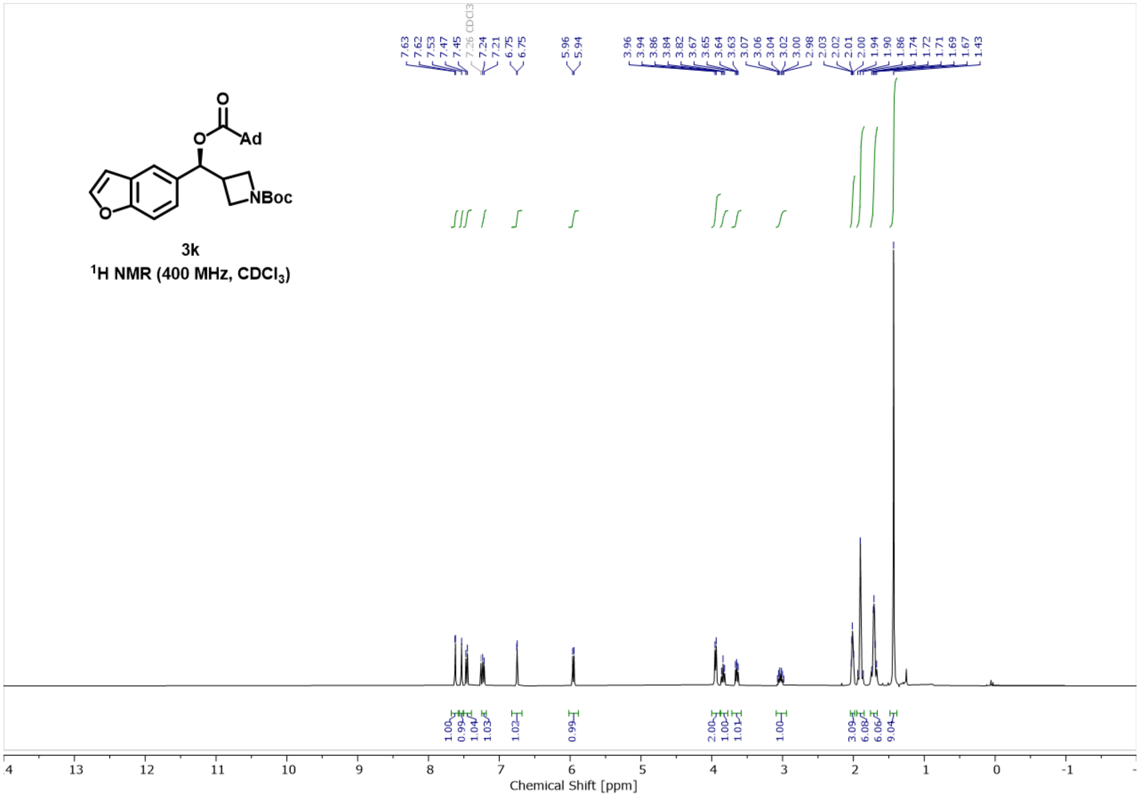


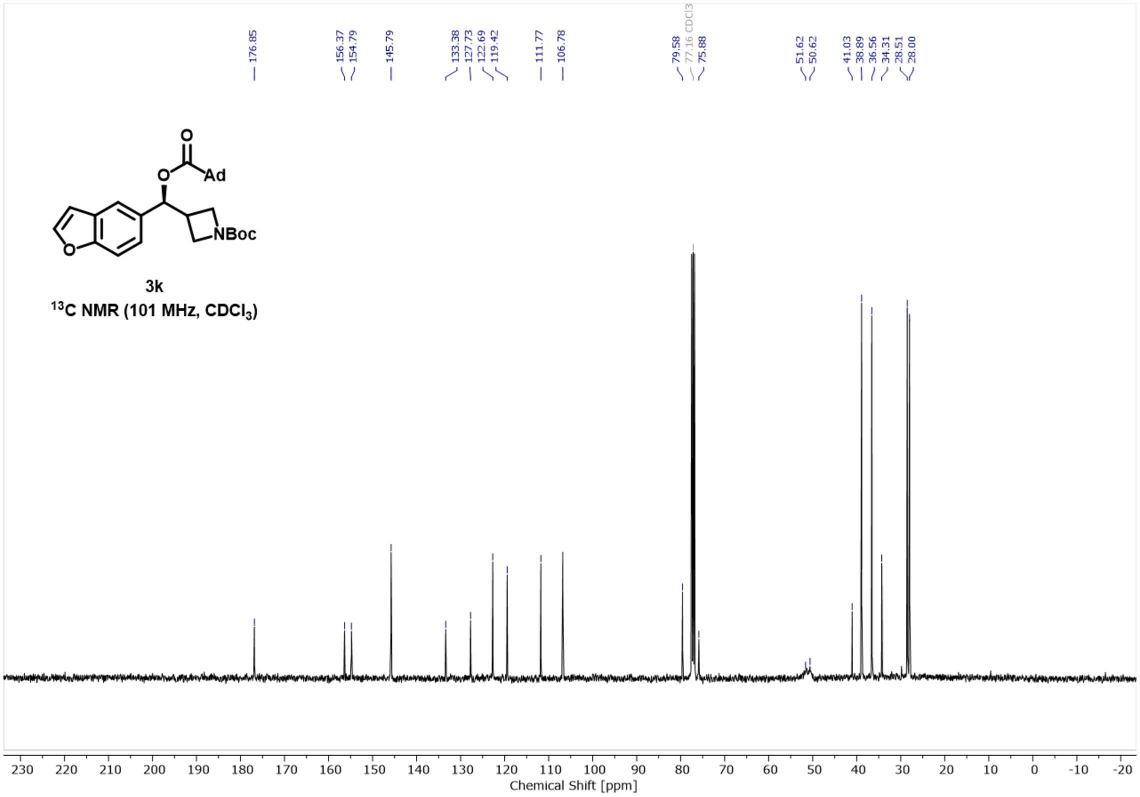

(*S*)-3-(2,3-dihydrobenzofuran-6-yl)-1-(naphthalen-2-yl)propyl benzoate (**3l**)

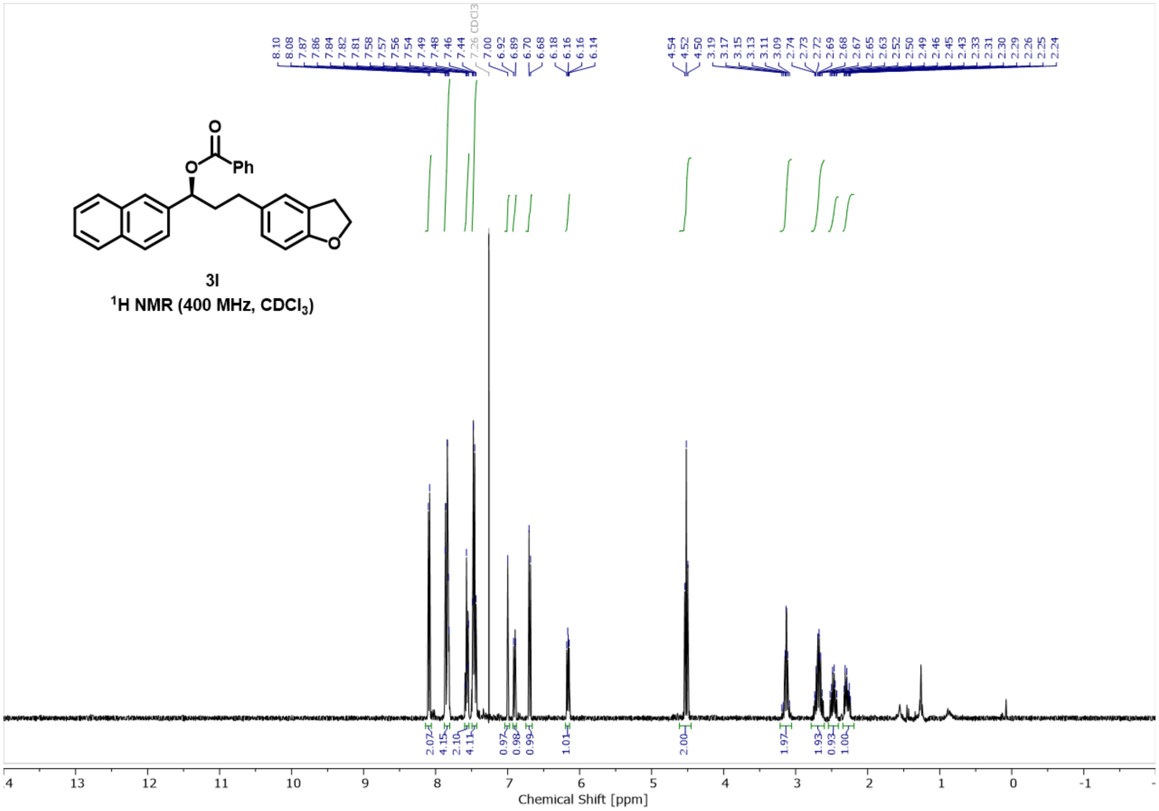


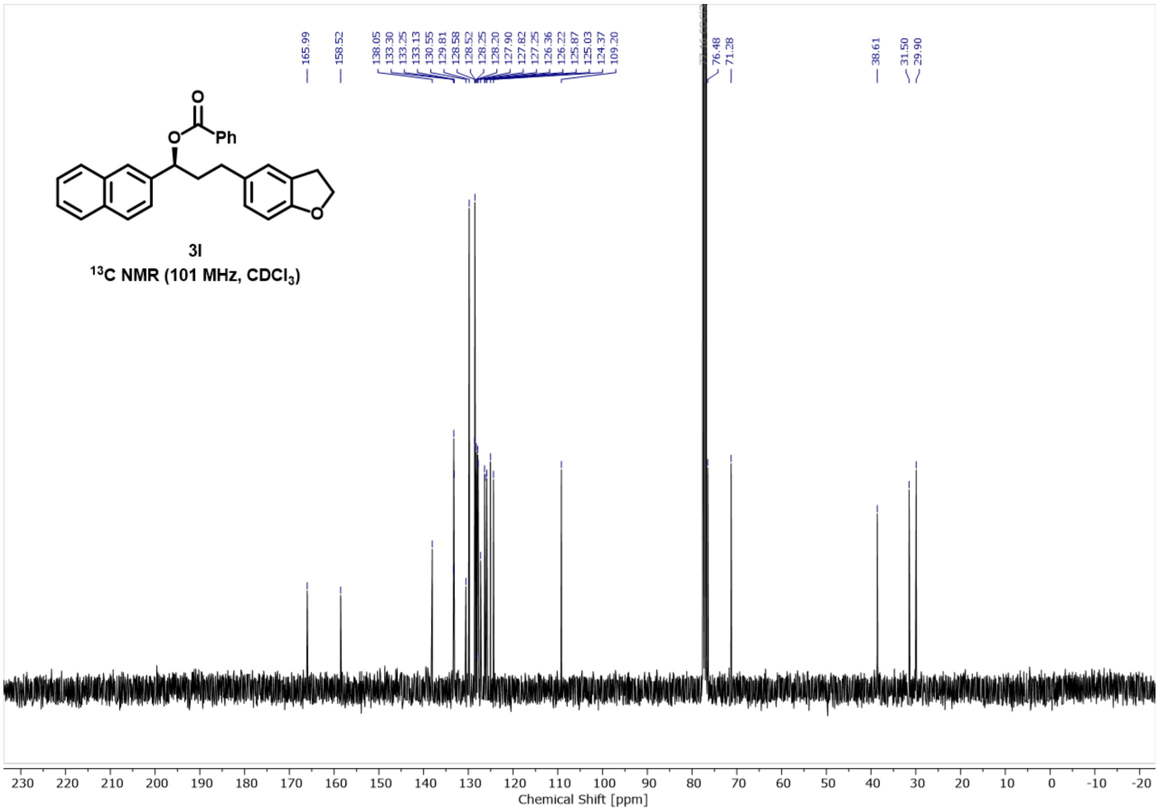

(*S*)-1-(benzo[*b*]thiophen-5-yl)butyl benzoate (**3m**)

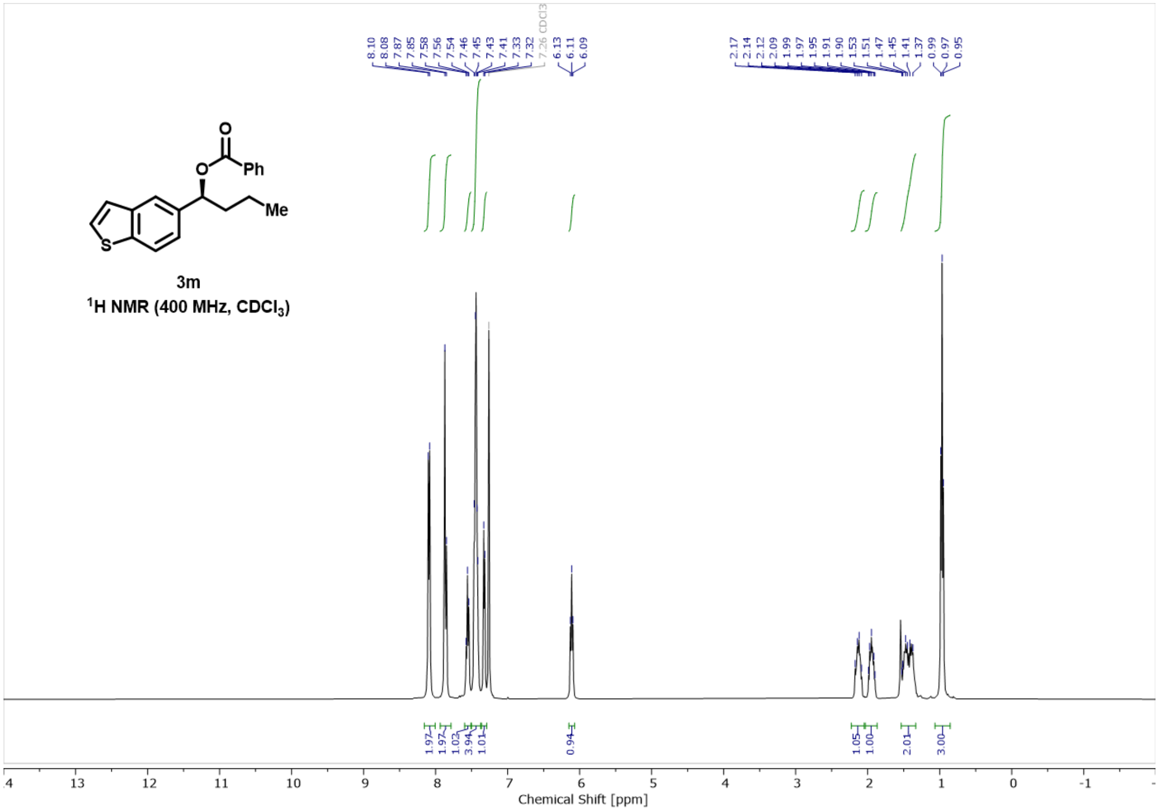


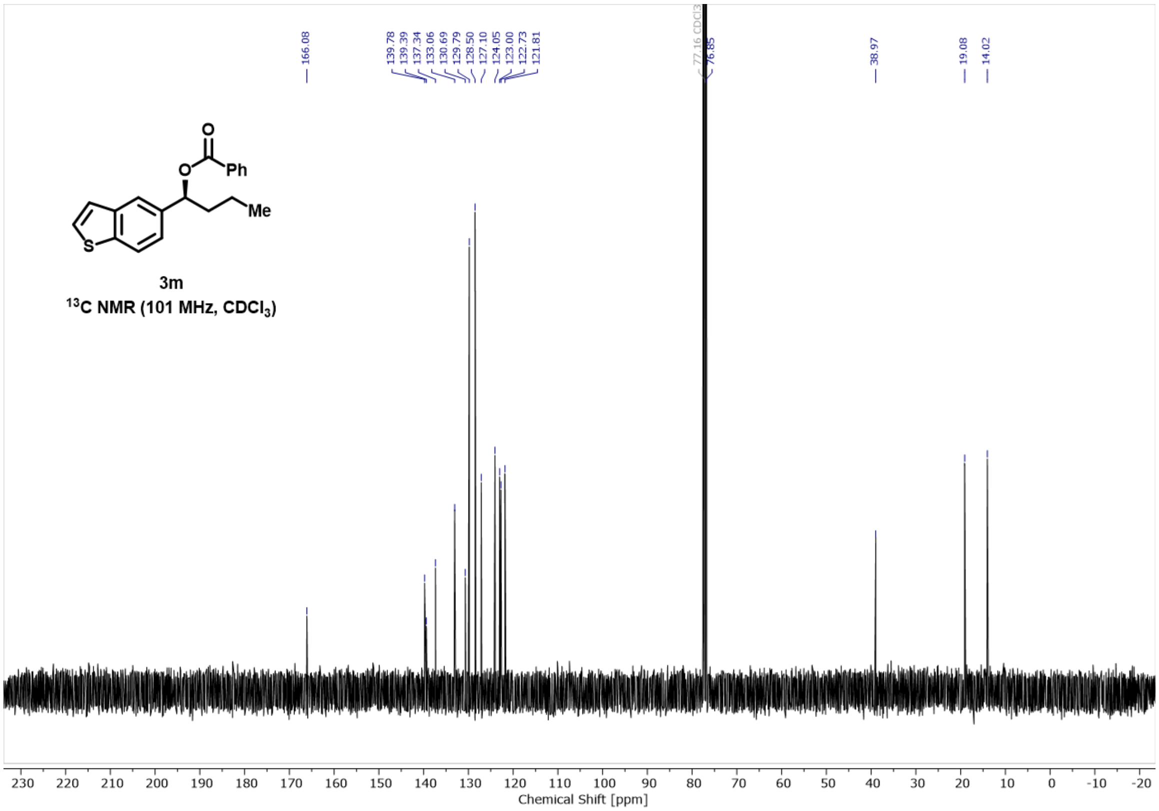

(*S*)-1-(6-bromonaphthalen-2-yl)ethyl benzoate (**3n**)

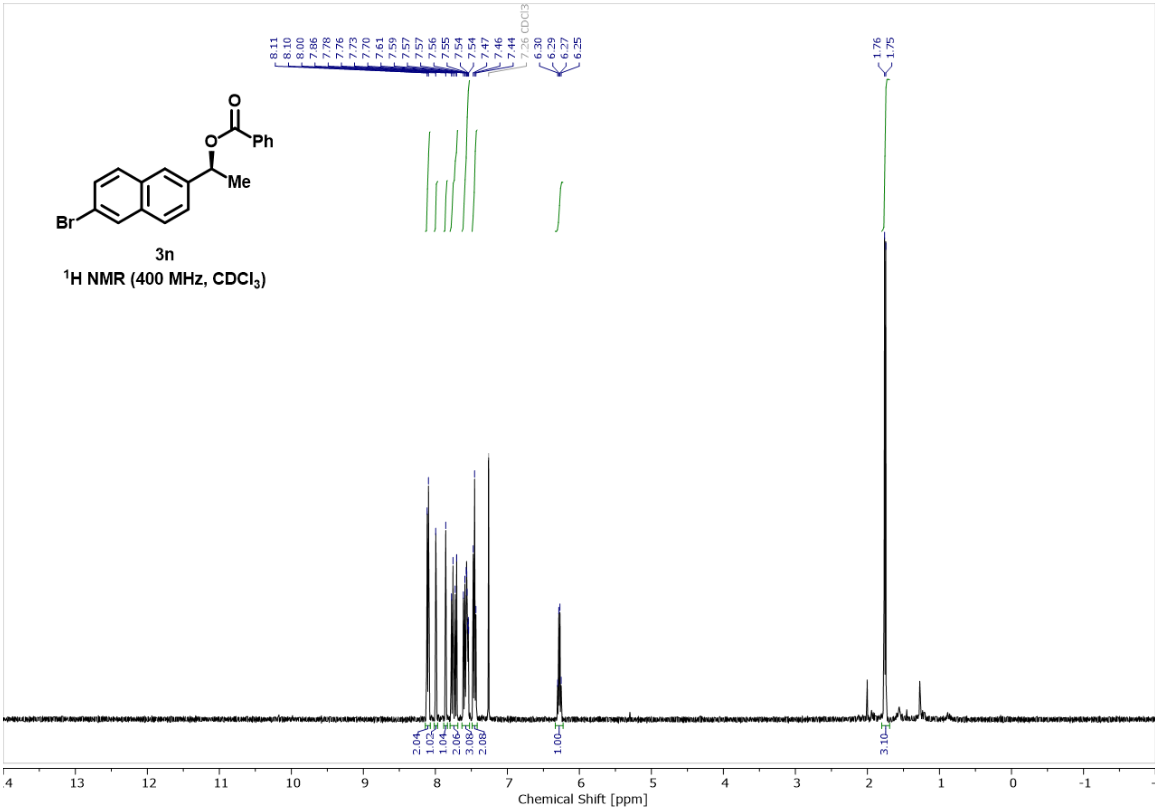


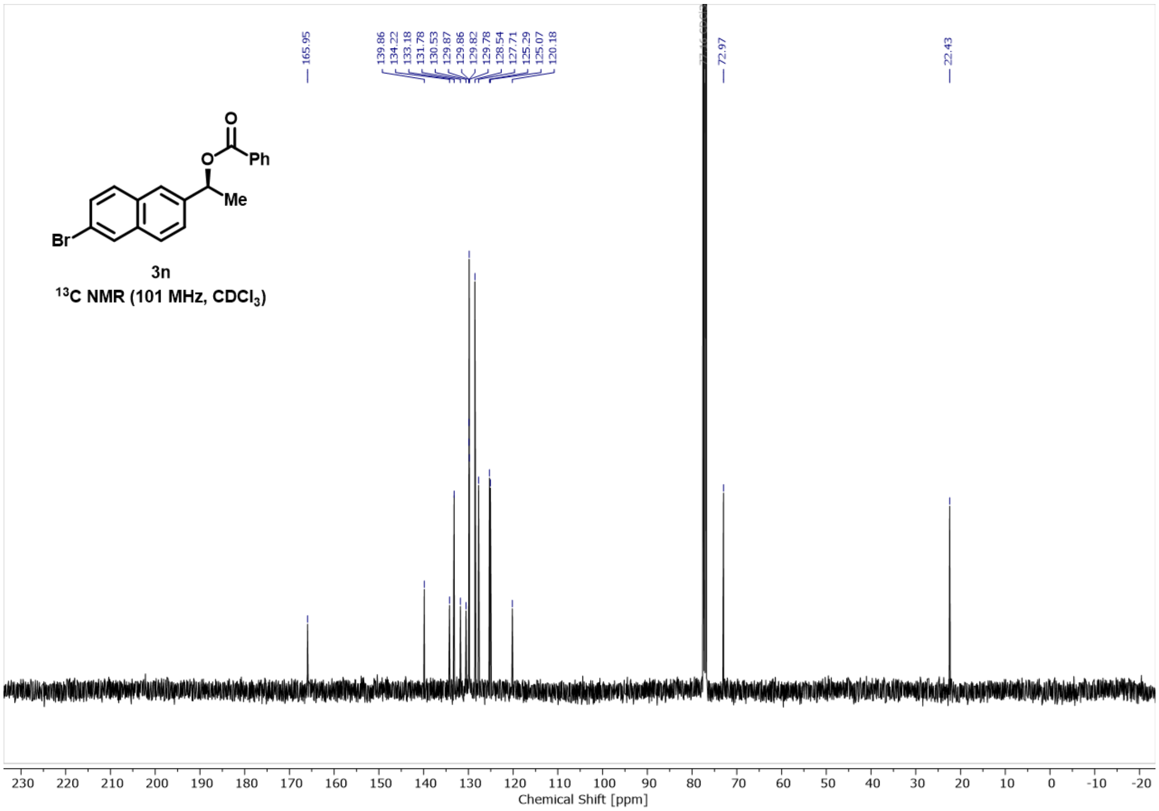

(*S*)-cyclobutyl(1-tosyl-1*H*-indol-5-yl)methyladamantane-1-carboxylate (**3o**)

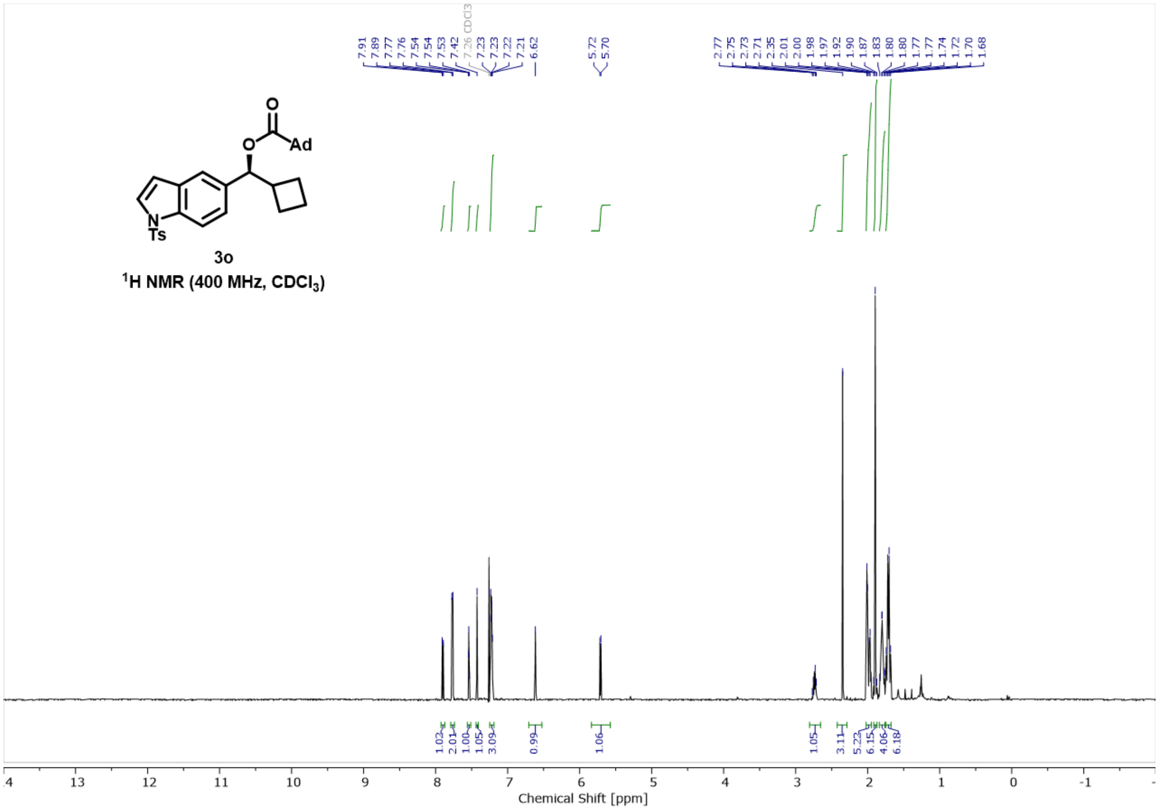


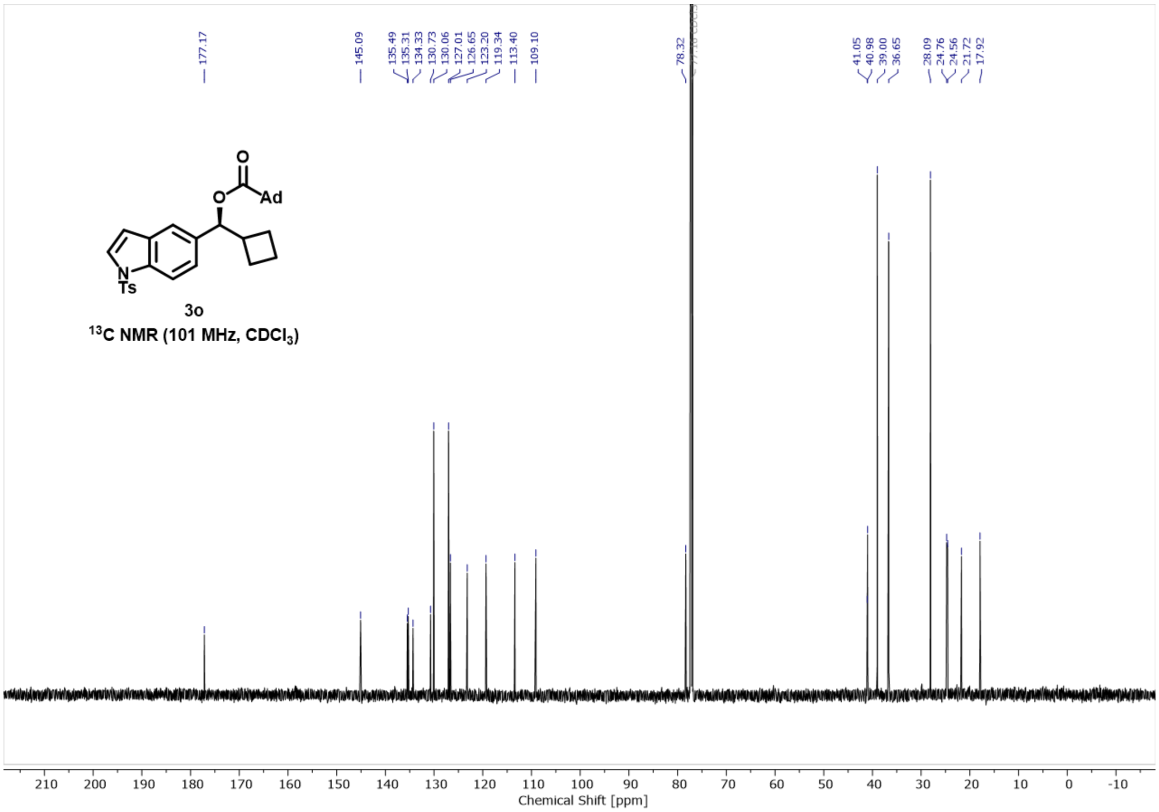

(*S*)-1-(naphthalen-2-yl)-3-(1*H*-pyrrol-1-yl)propyl benzoate (**3p**)

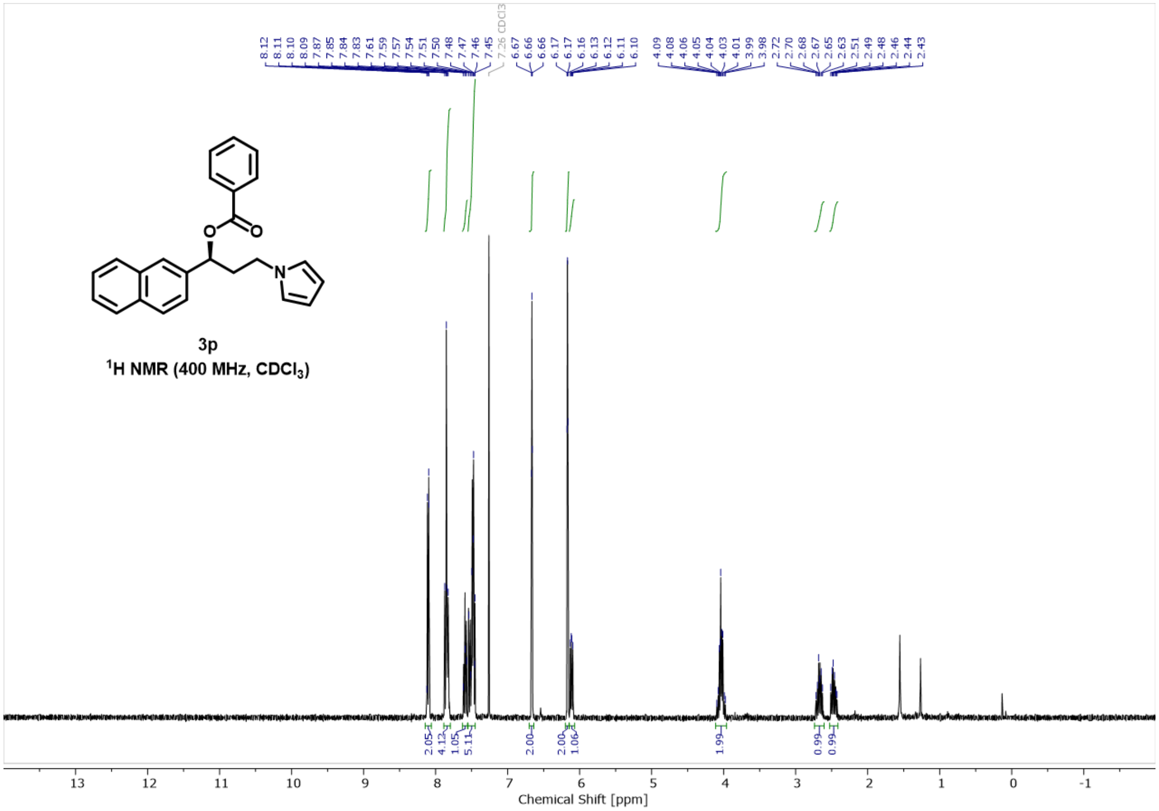


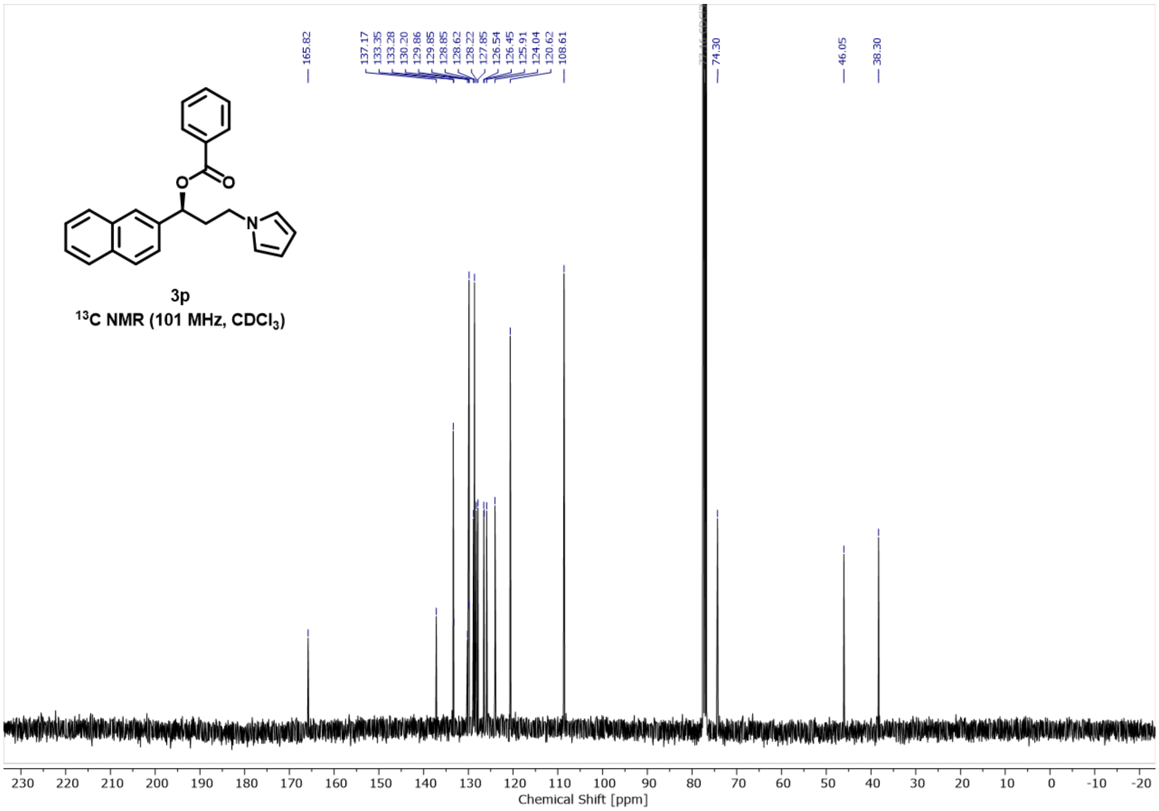

(*S*,*E*)-4-phenylbut-3-en-2-yl benzoate (**3q**)

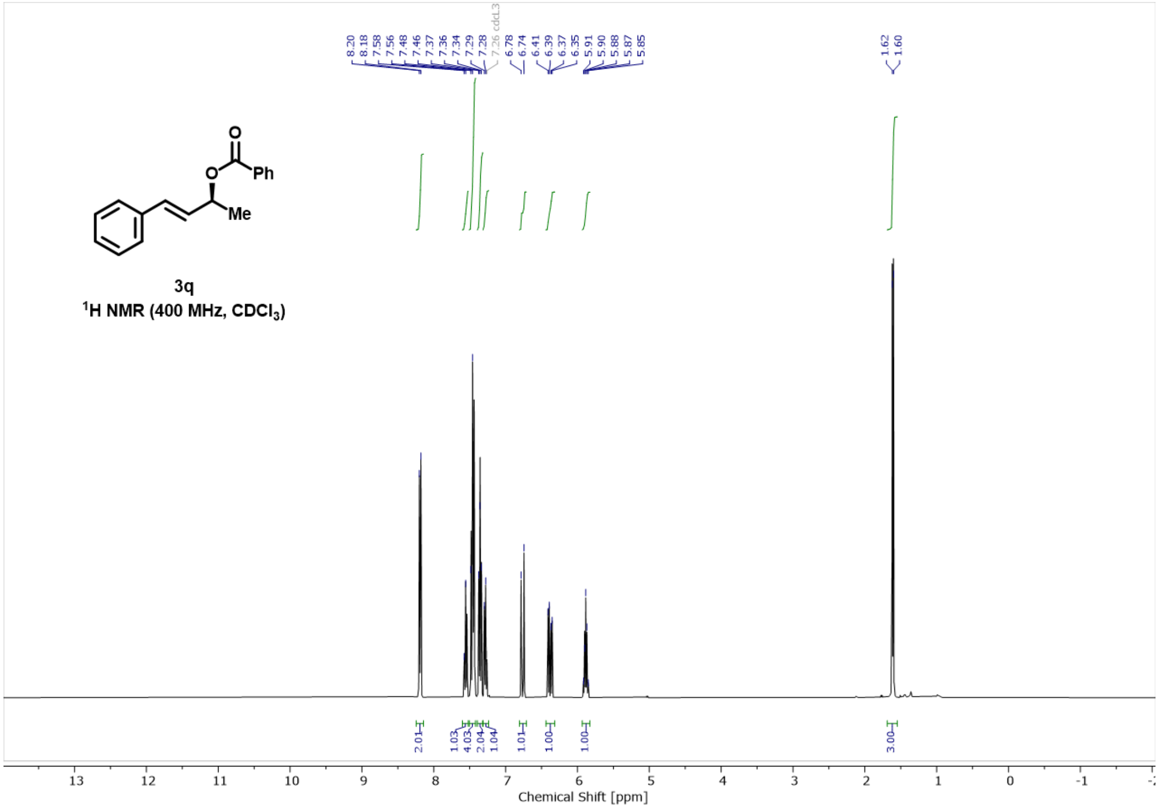


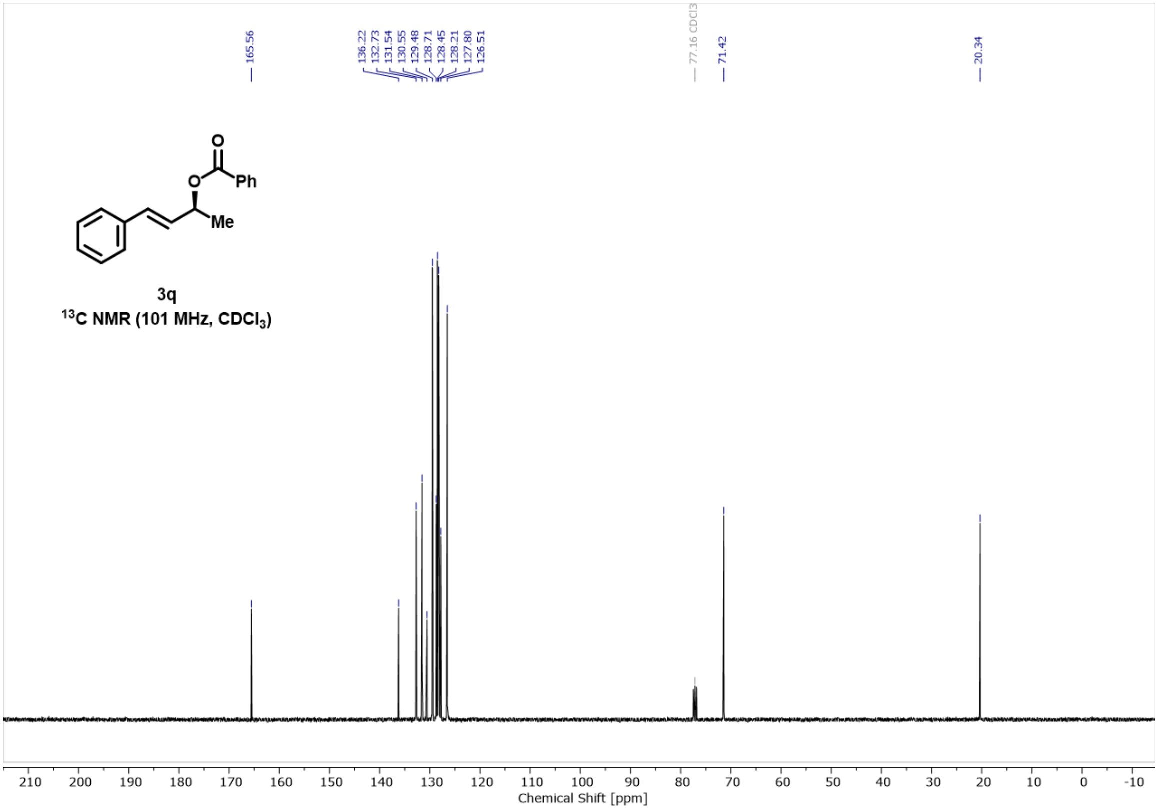

(*S*,*E*)-1-phenyloct-1-en-3-yladamantane-1-carboxylate (**3r**)

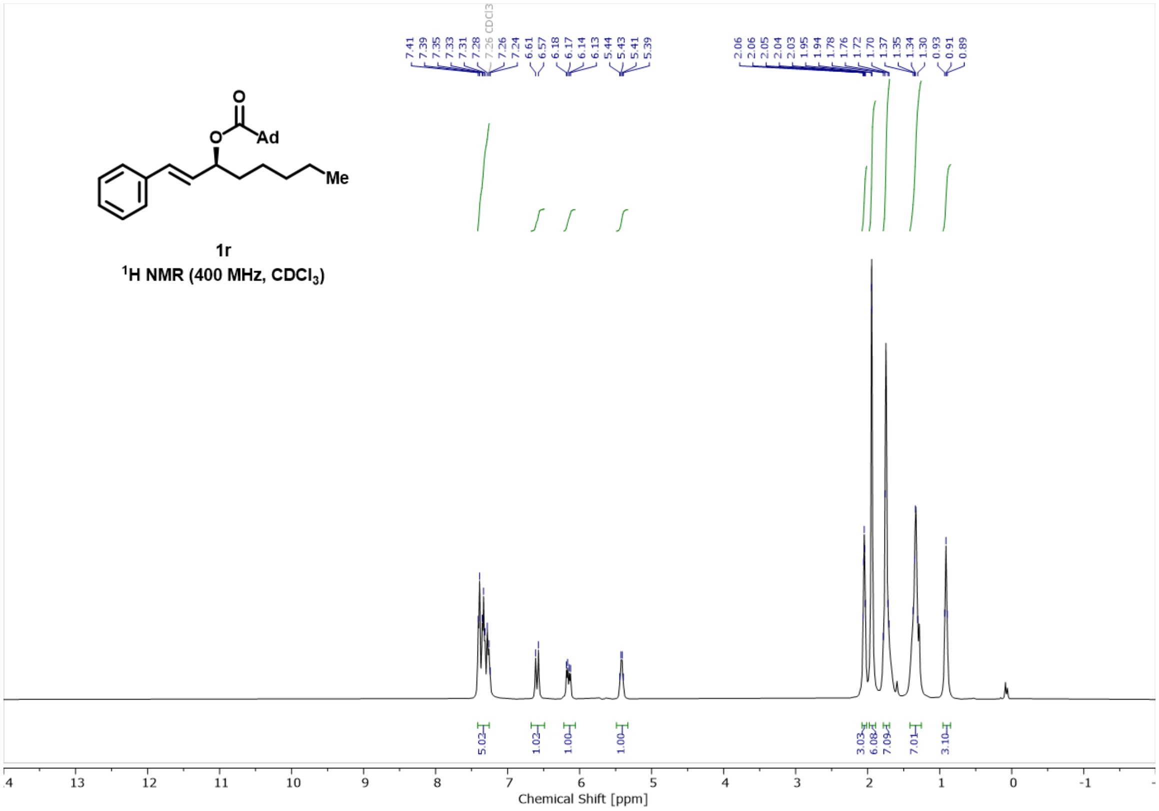


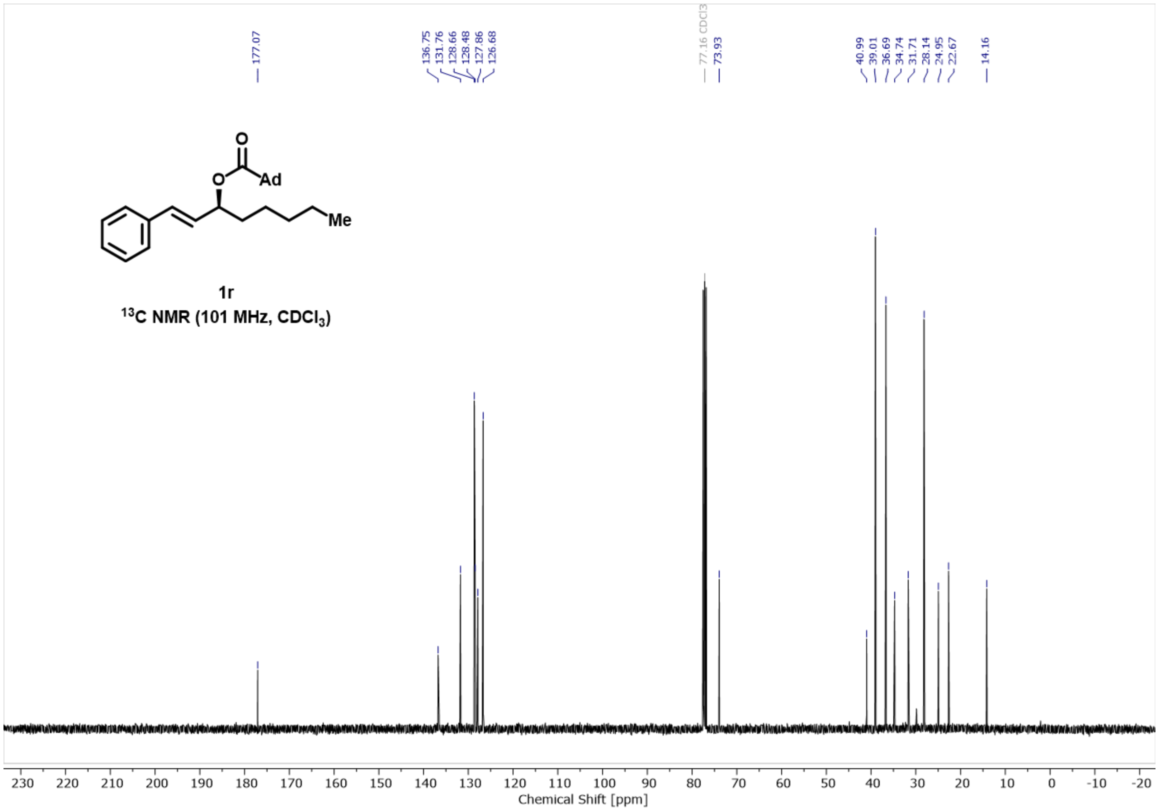

(*S*,*E*)-5-(1,3-dioxan-2-yl)-1-phenylpent-1-en-3-yladamantane-1-carboxylate (**3s**)

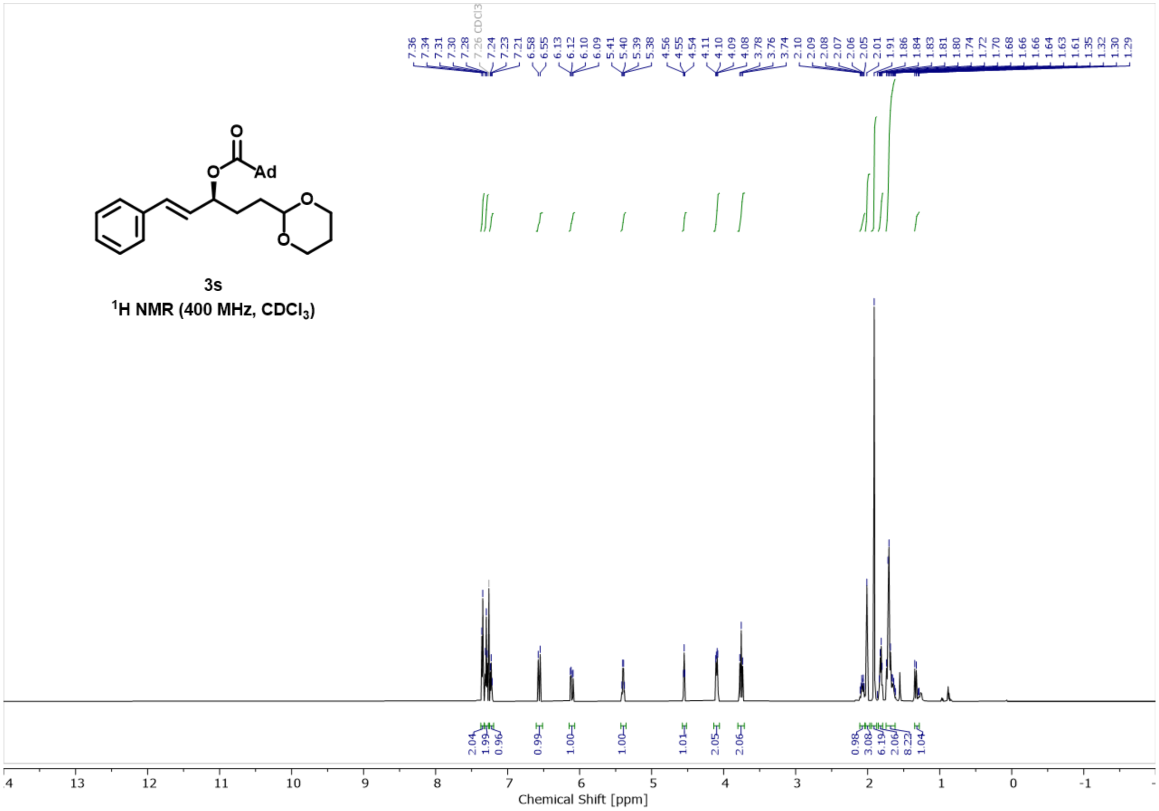


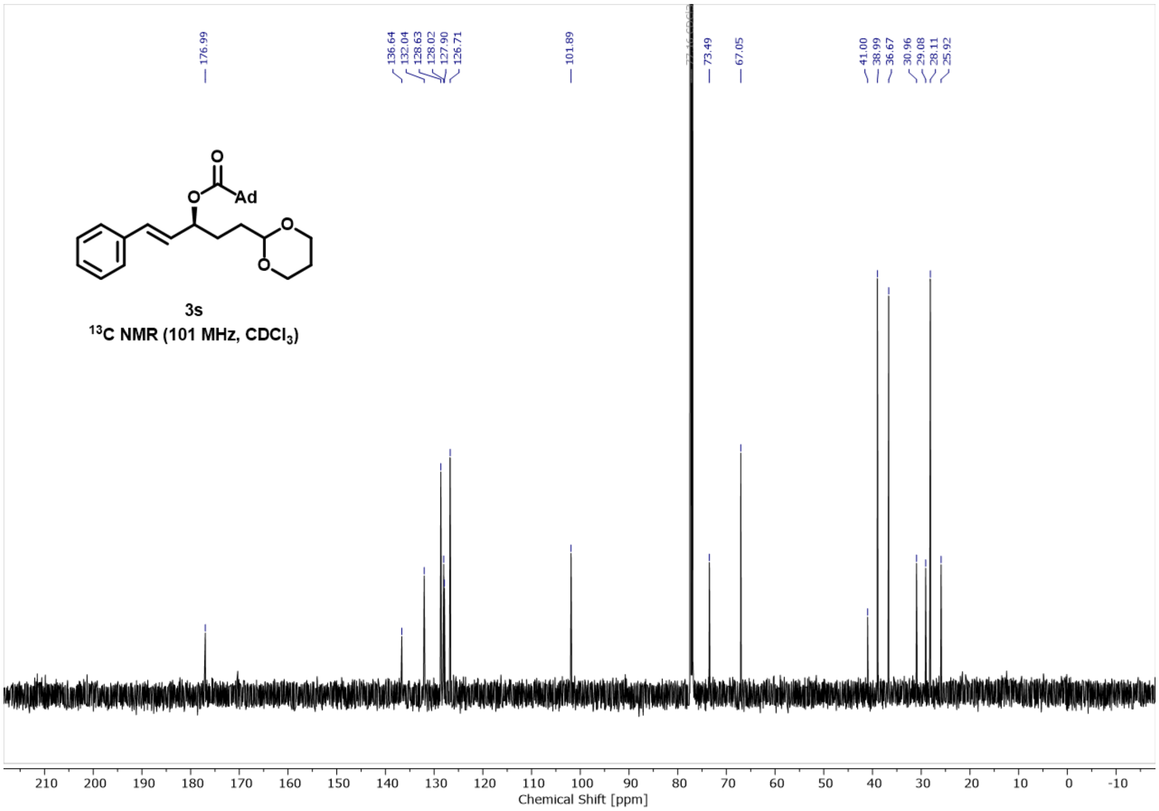

(*S*)-1-(naphthalen-2-yl)ethyl acetate (**3t**)

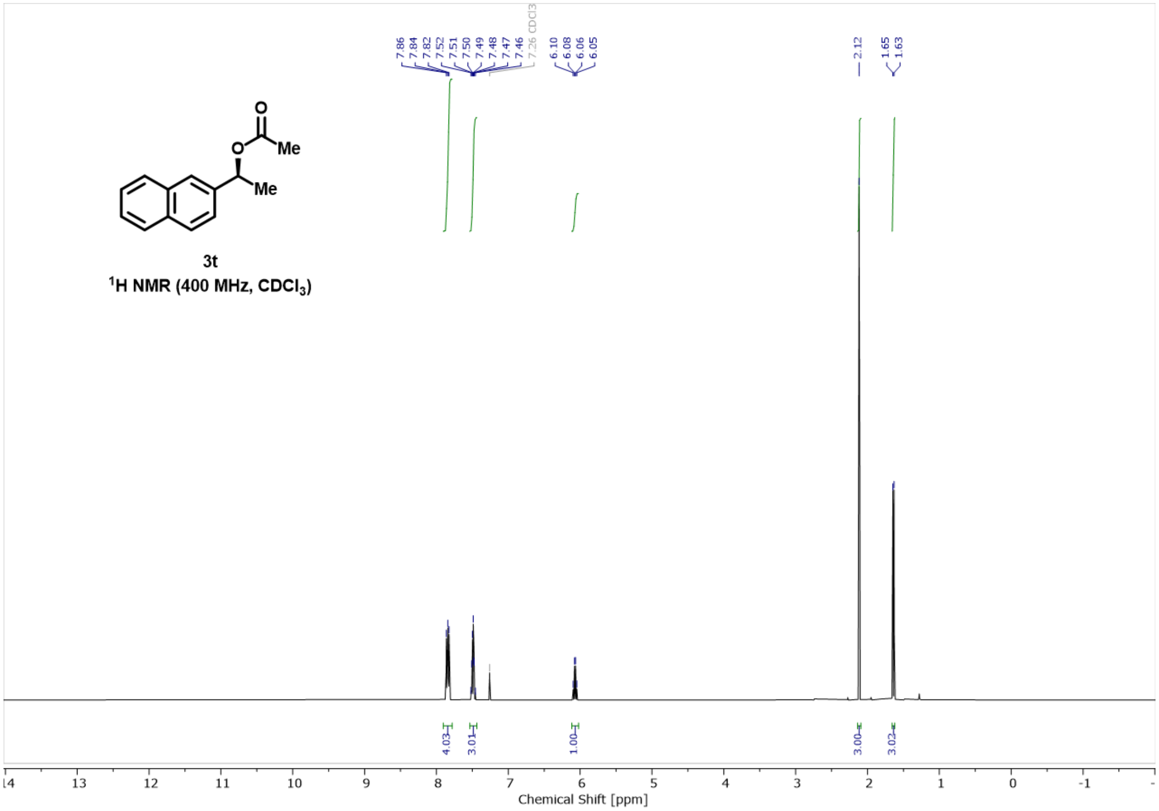


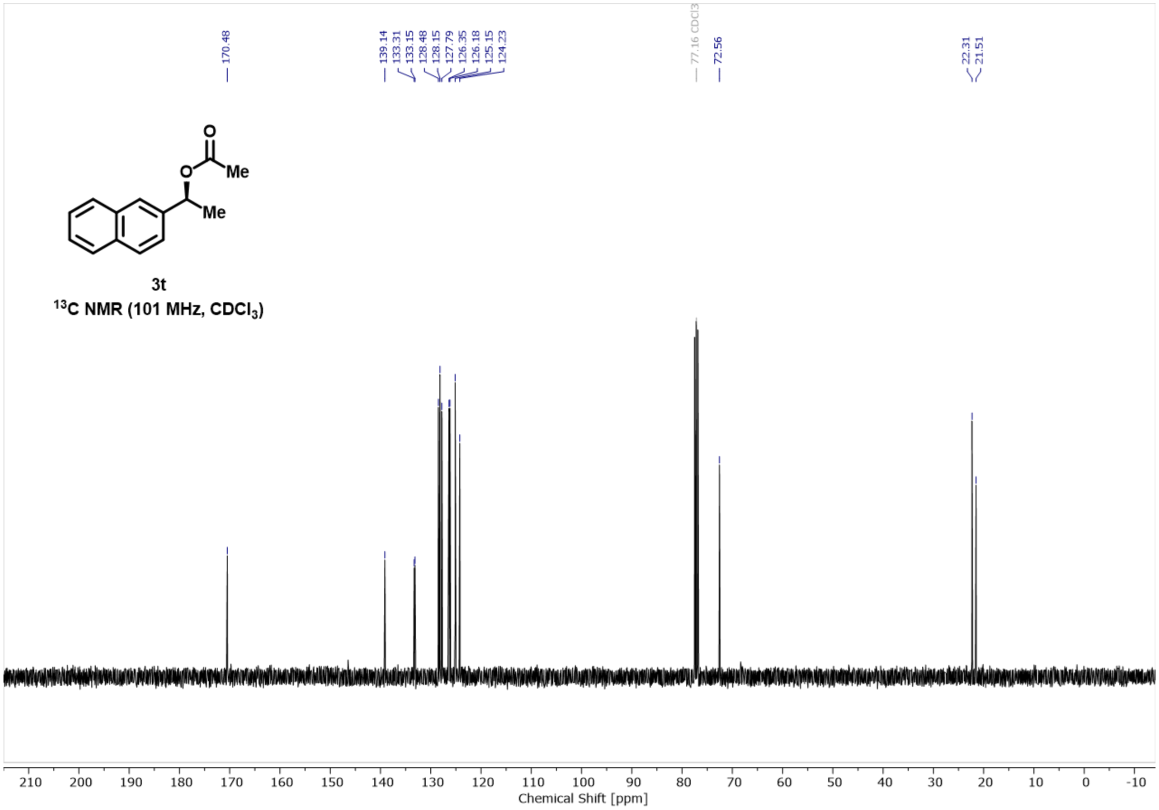

(*S*)-1-(naphthalen-2-yl)ethyl 2-methylbenzo[*d*]oxazole-5-carboxylate (**3u**)

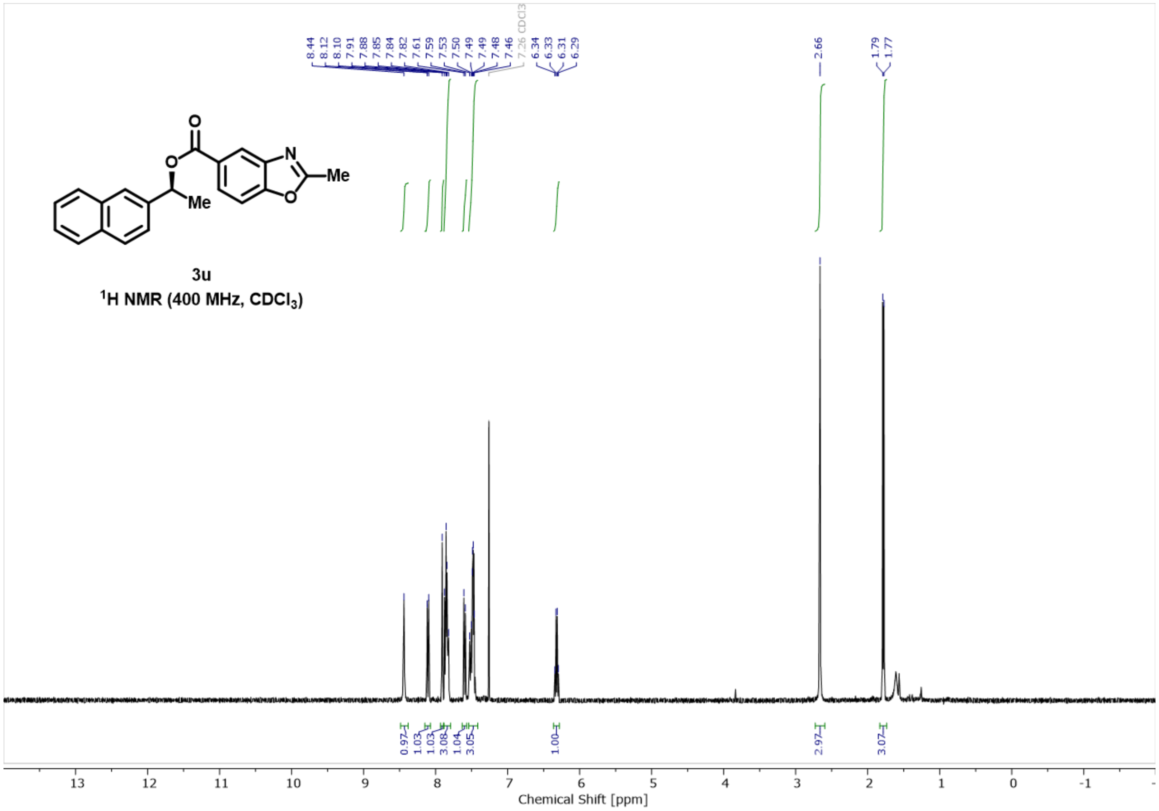

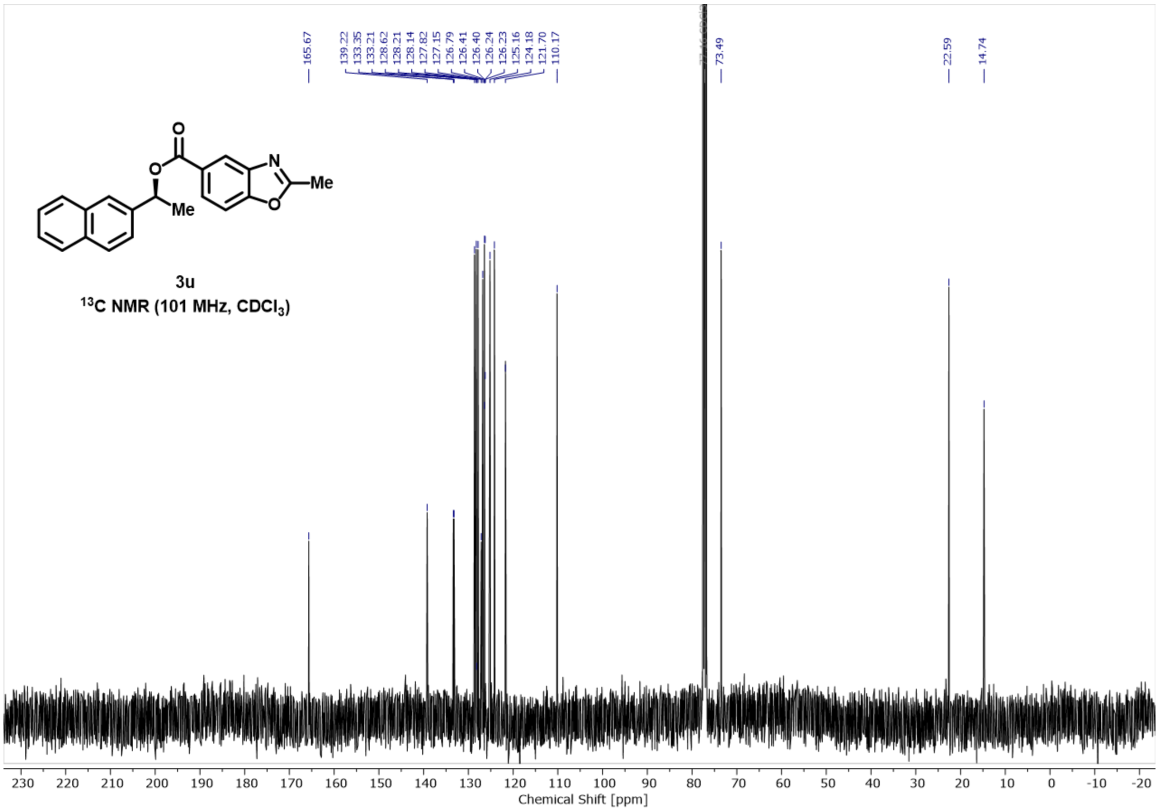

(*S*)-1-(naphthalen-2-yl)ethyladamantane-1-carboxylate (**3v**)

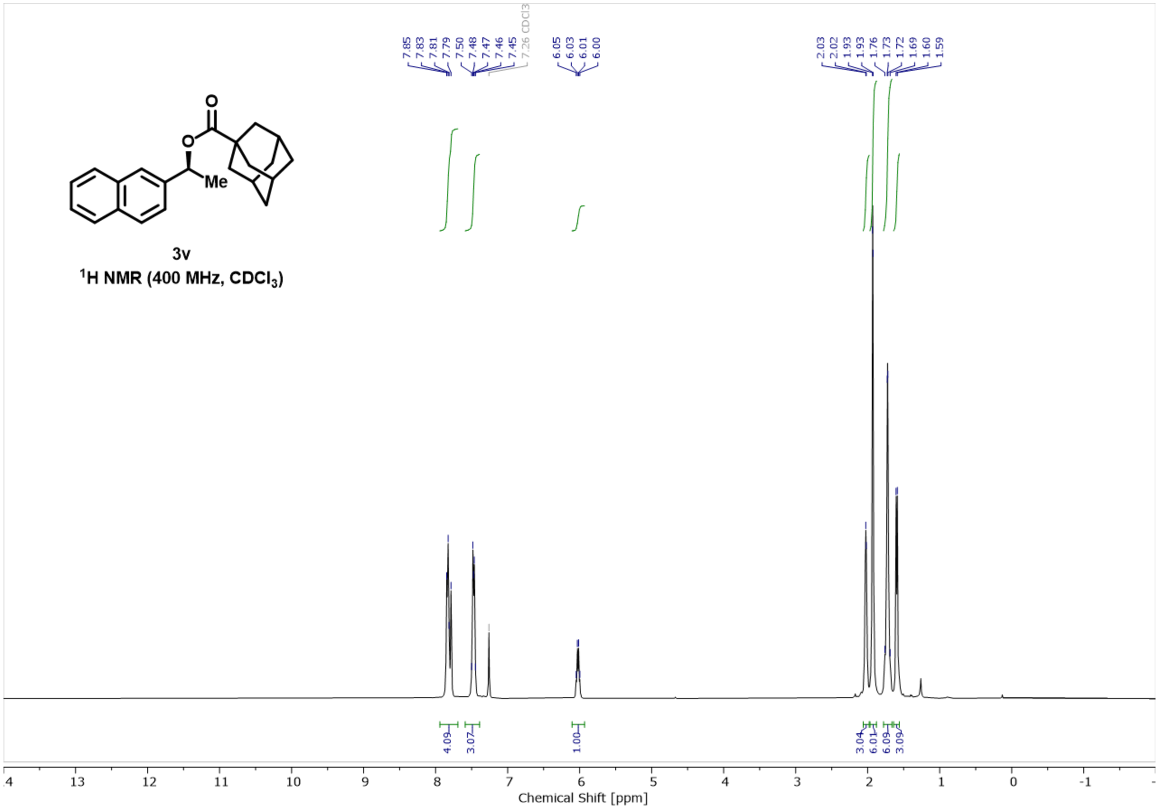


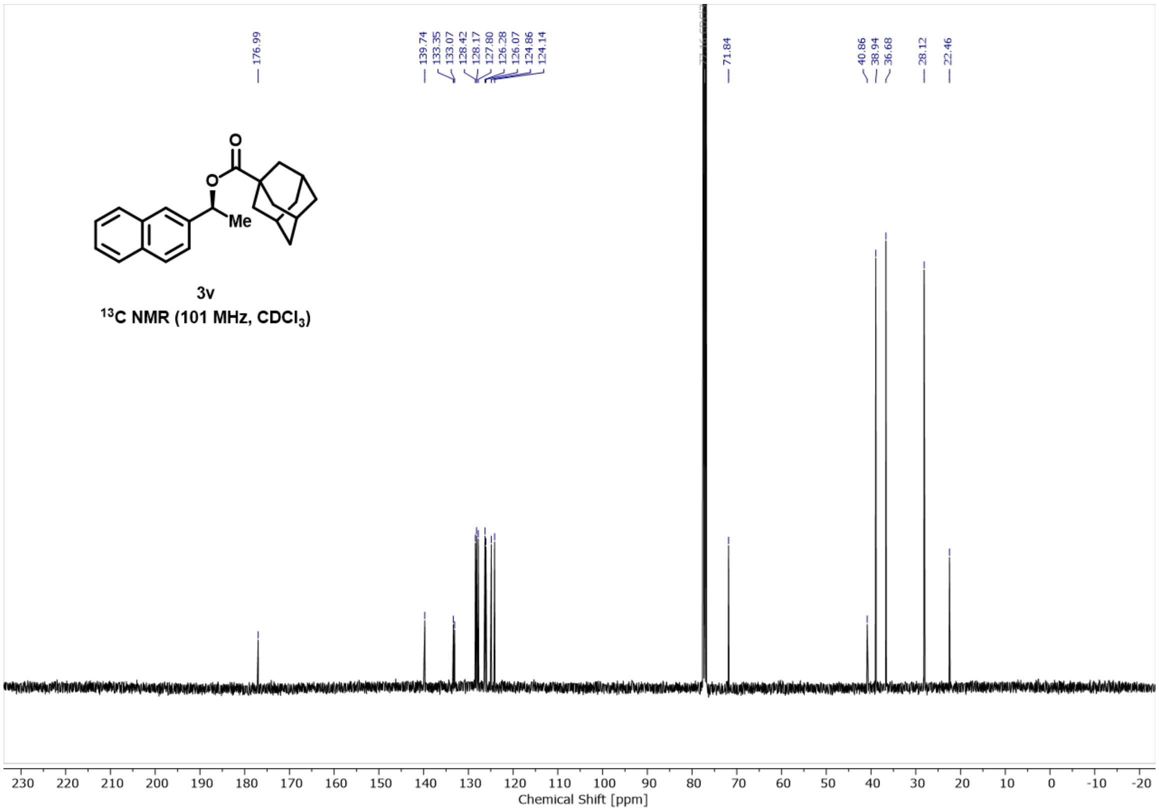

(*S*)-1-(naphthalen-2-yl)ethyl 4-bromobutanoate (**3w**)

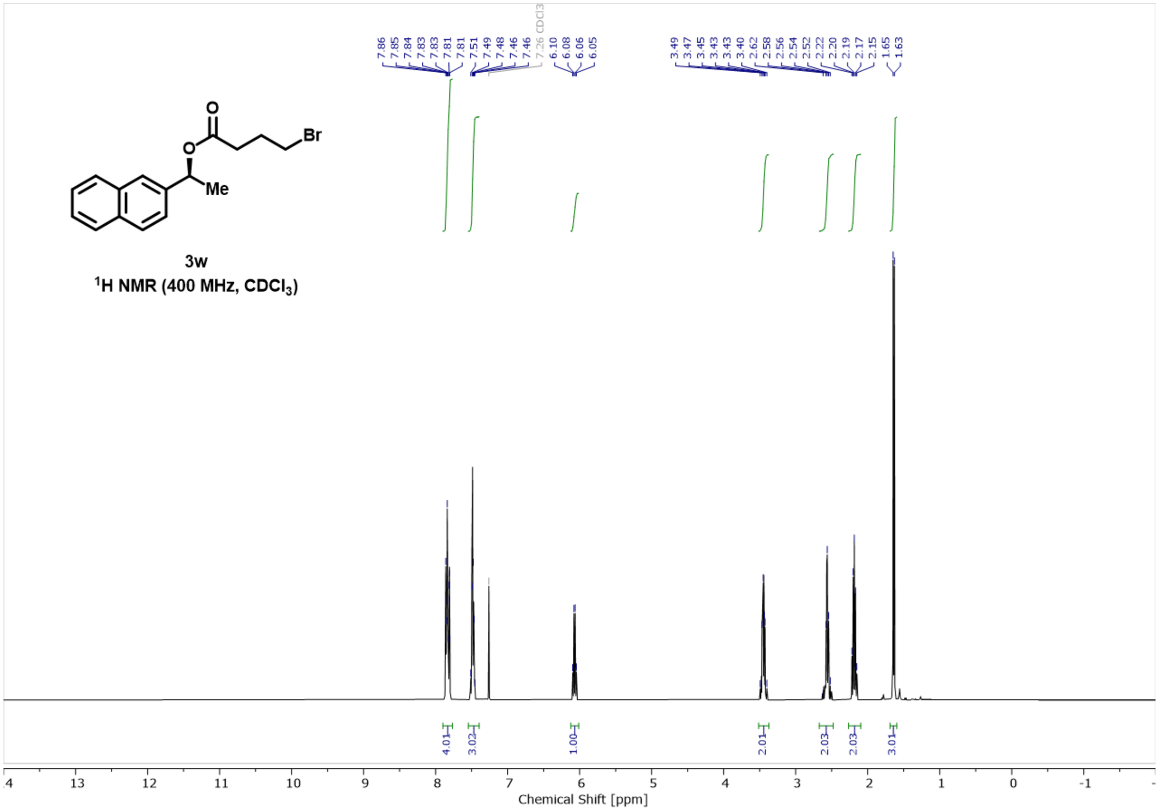


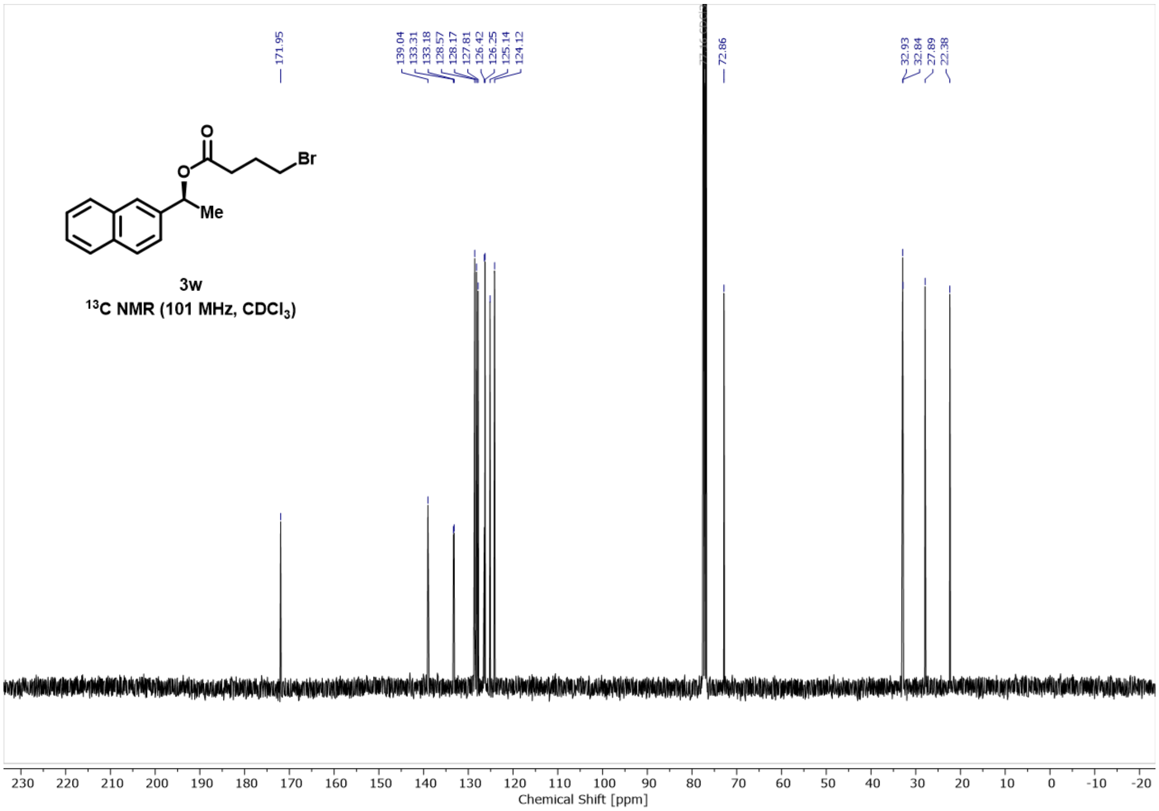

(*S*)-1-(naphthalen-2-yl)ethyl 4,4-difluorocyclohexane-1-carboxylate (**3x**)

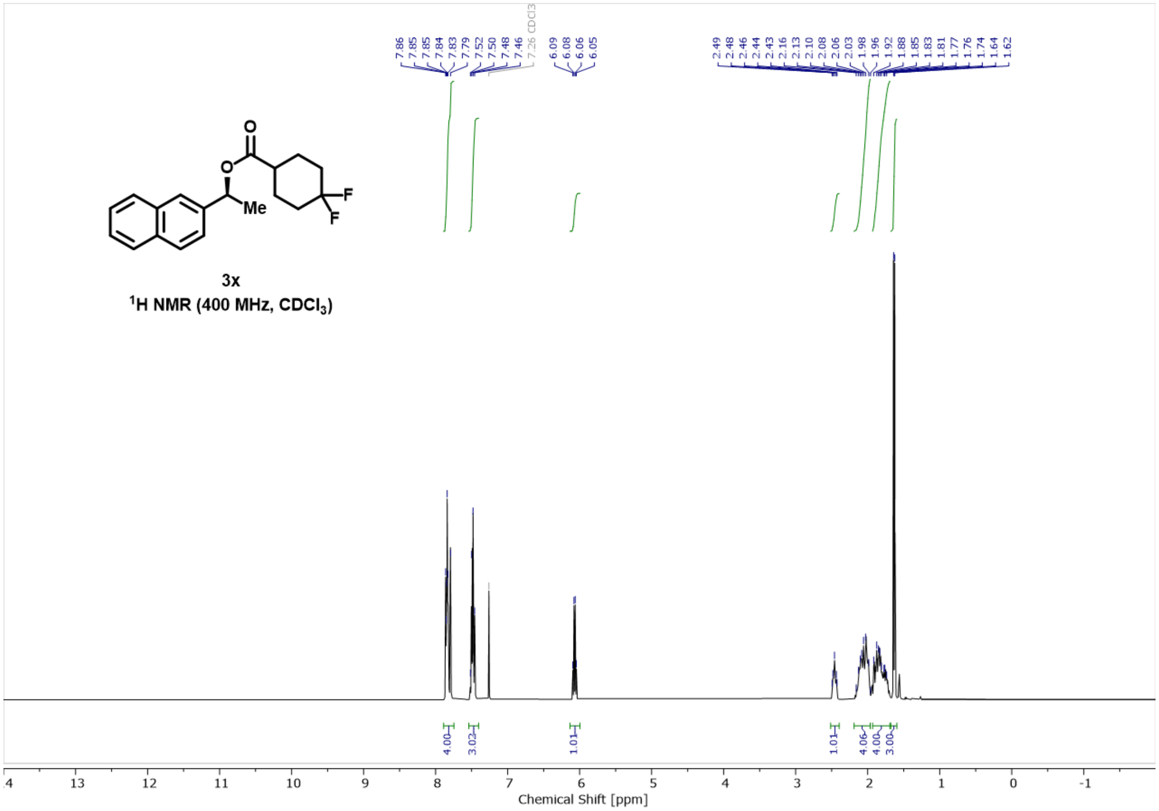


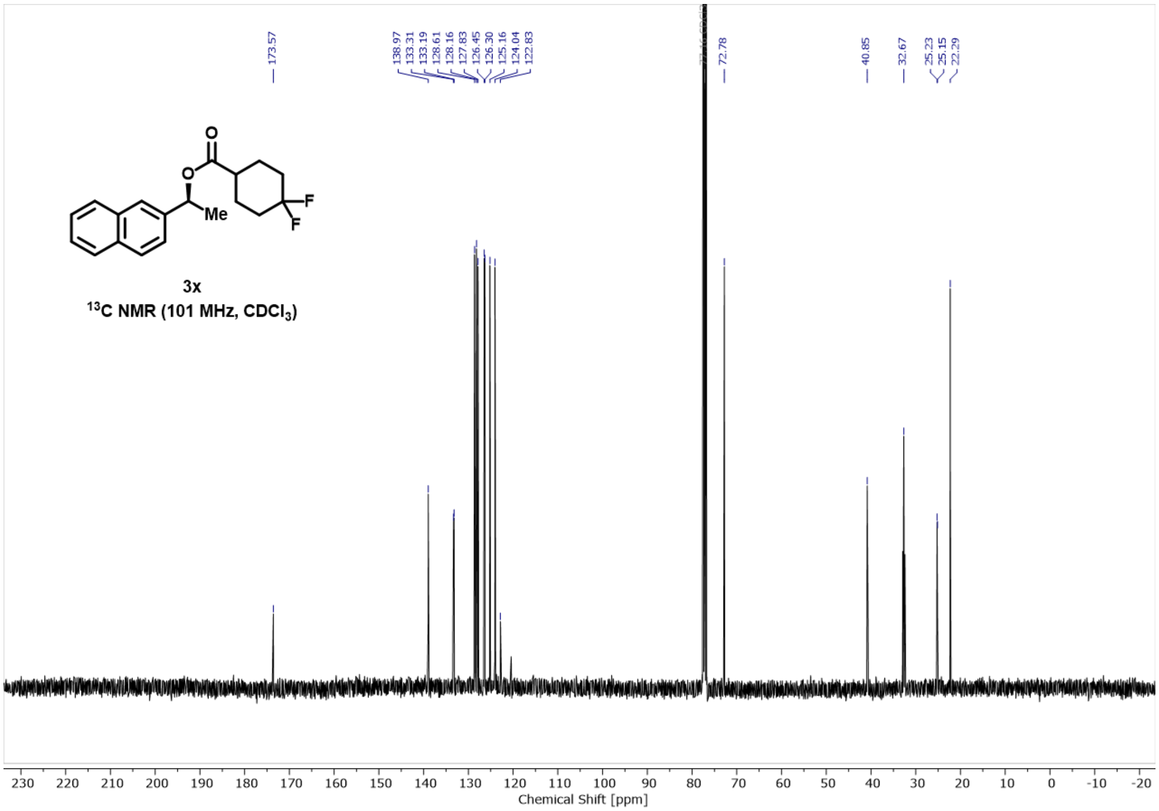

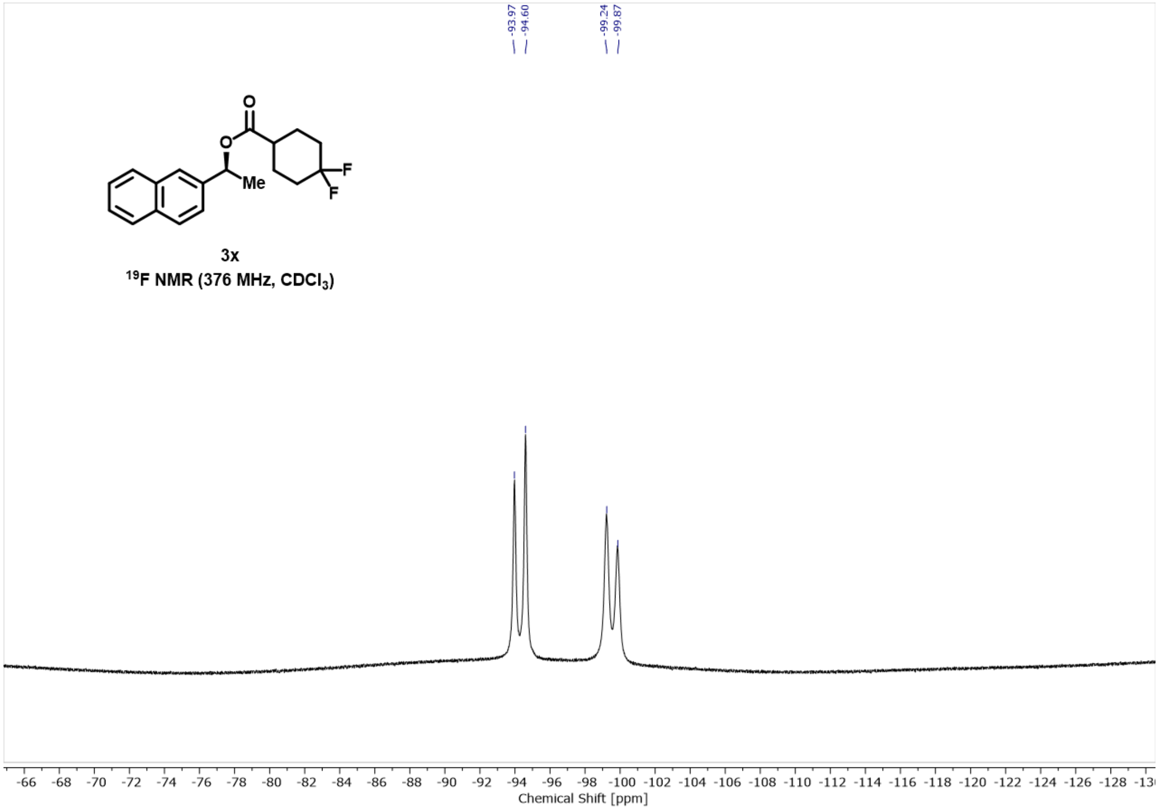
-93.97
-94.60
-99.24
-99.87
O
O
Me
F
F
3x
19F NMR (376 MHz, CDCl3)
-66 -68 -70 -72 -74 -76 -78 -80 -82 -84 -86 -88 -90 -92 -94 -96 -98 -100 -102 -104 -106 -108 -110 -112 -114 -116 -118 -120 -122 -124 -126 -128
Chemical Shift [ppm]

(*S*)-1-(naphthalen-2-yl)ethyl benzo[*b*]thiophene-3-carboxylate (**3y**)

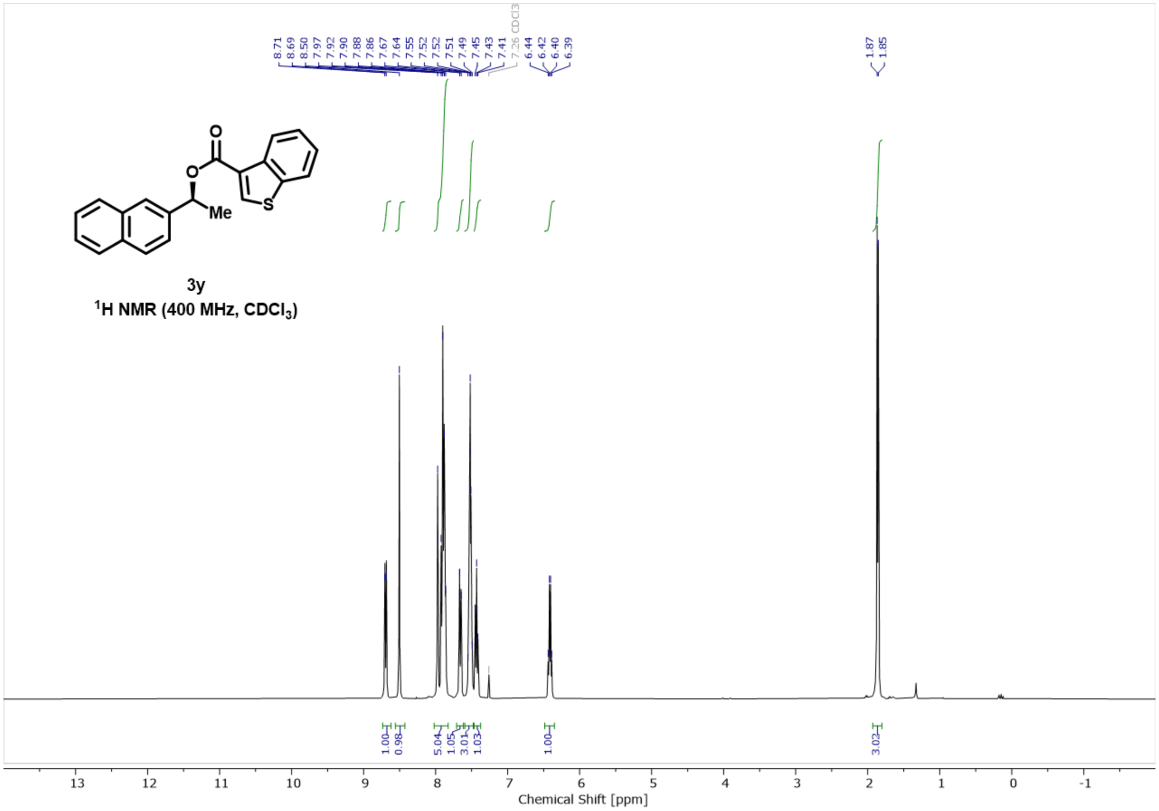


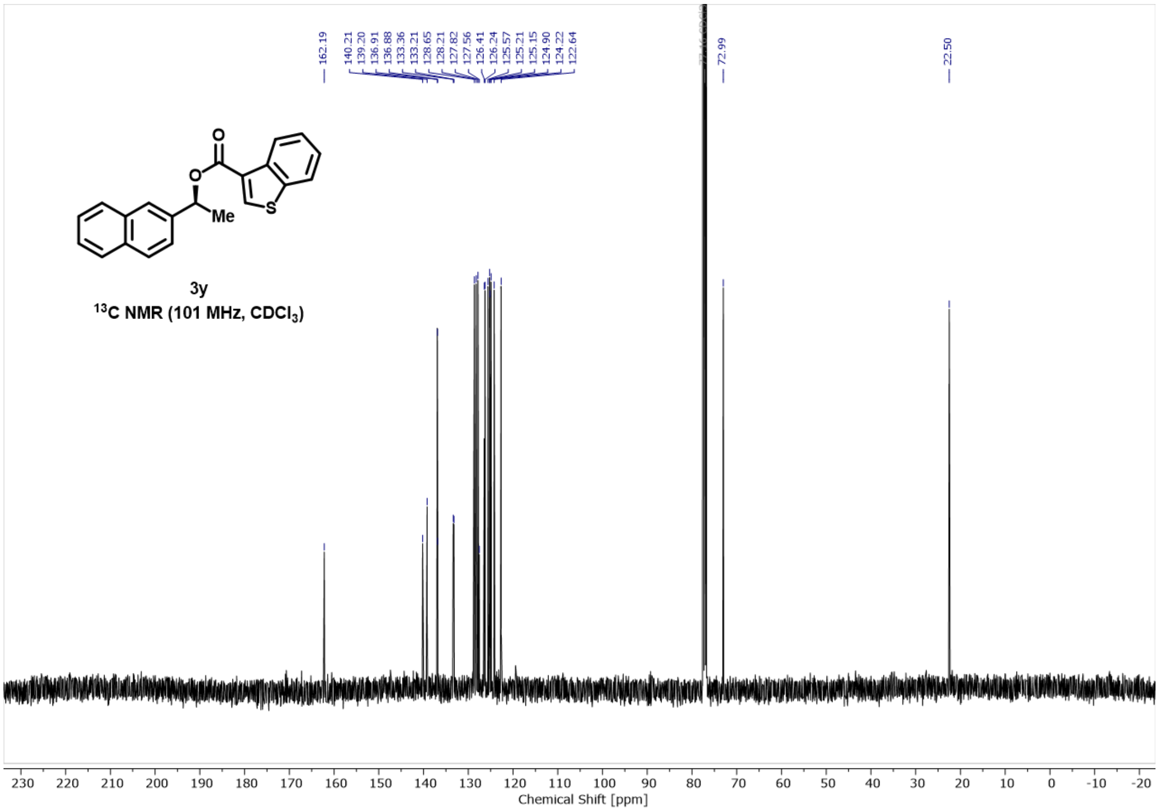

(*S*)-1-(naphthalen-2-yl)ethyl tetrahydro-2*H*-pyran-4-carboxylate (**3z**)

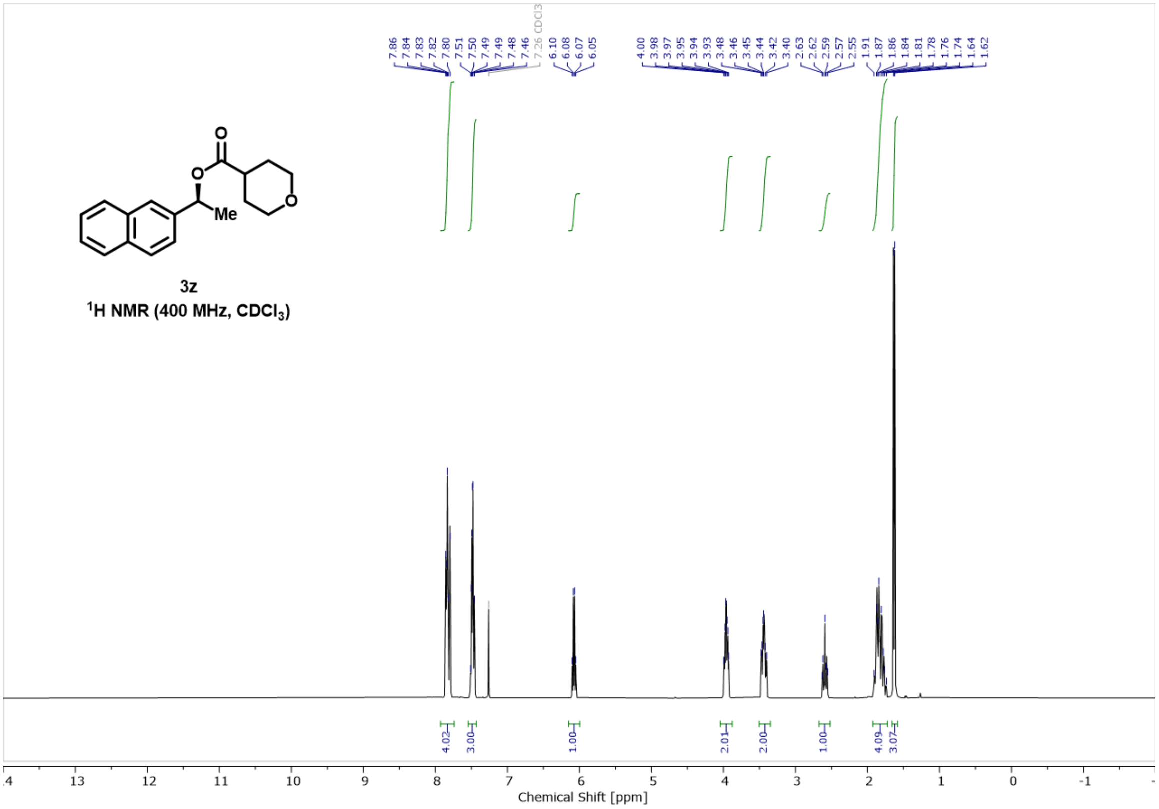


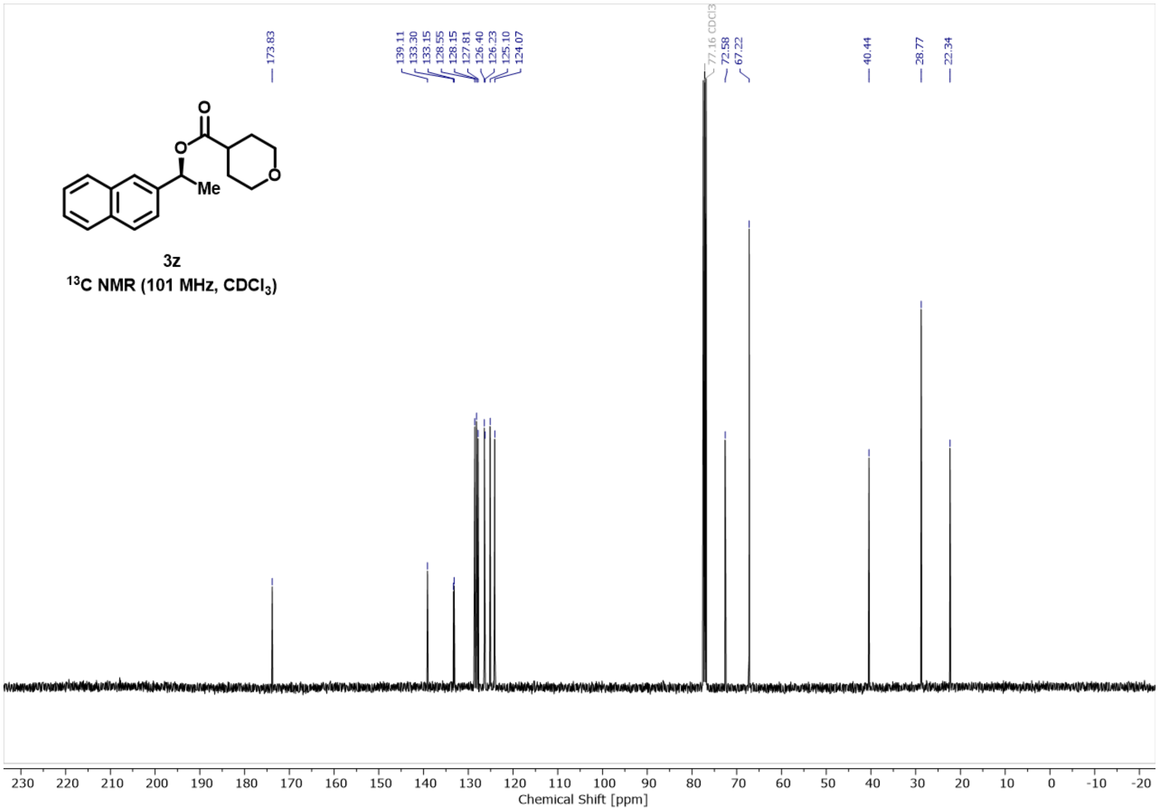

(*S*)-1-(naphthalen-2-yl)ethyl 2, 1-(naphthalen-2-yl)ethyl cyclobutanecarboxylate (**3aa**)

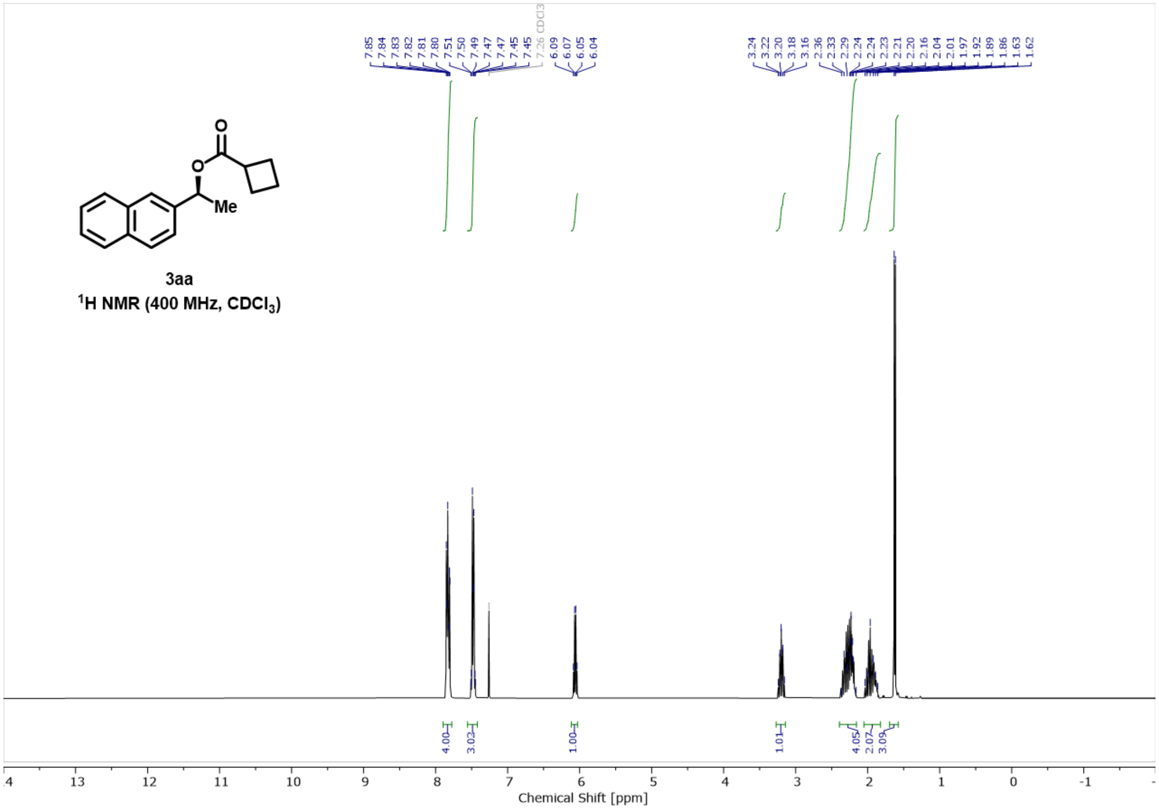


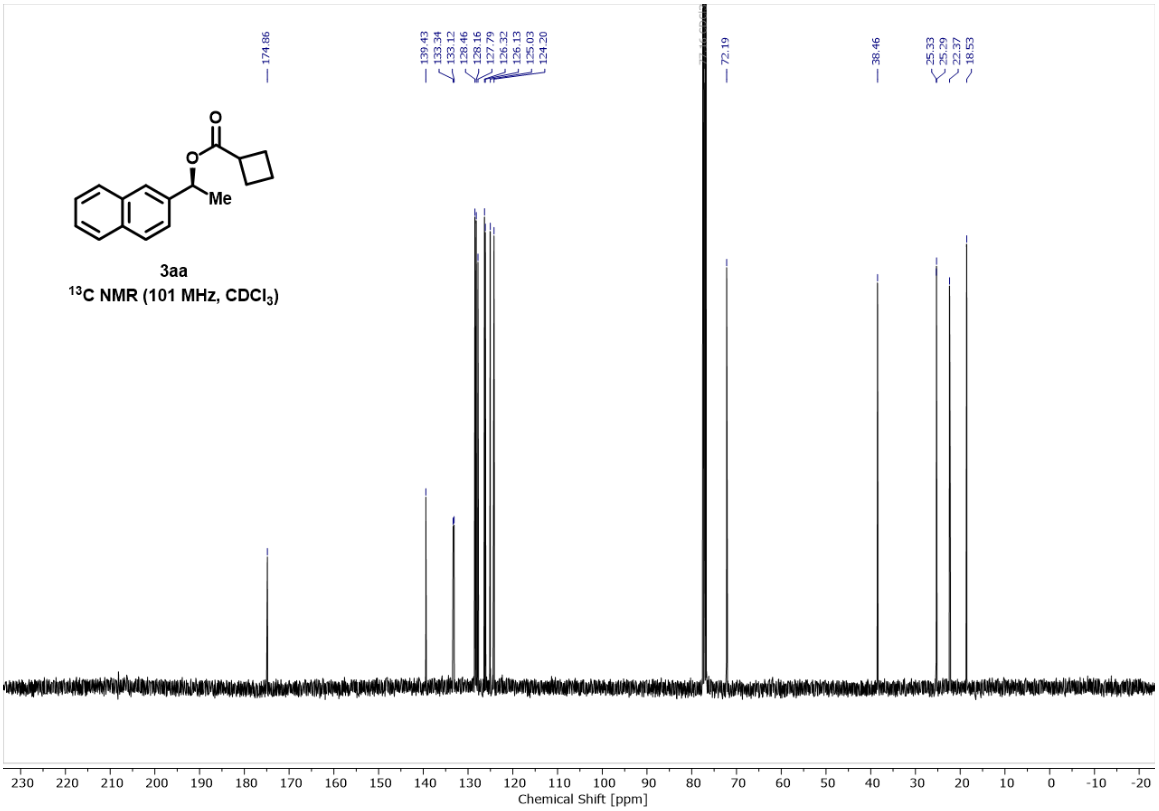

(*S*)-1-(naphthalen-2-yl)ethyl 3-methyloxetane-3-carboxylate (**3ab**)

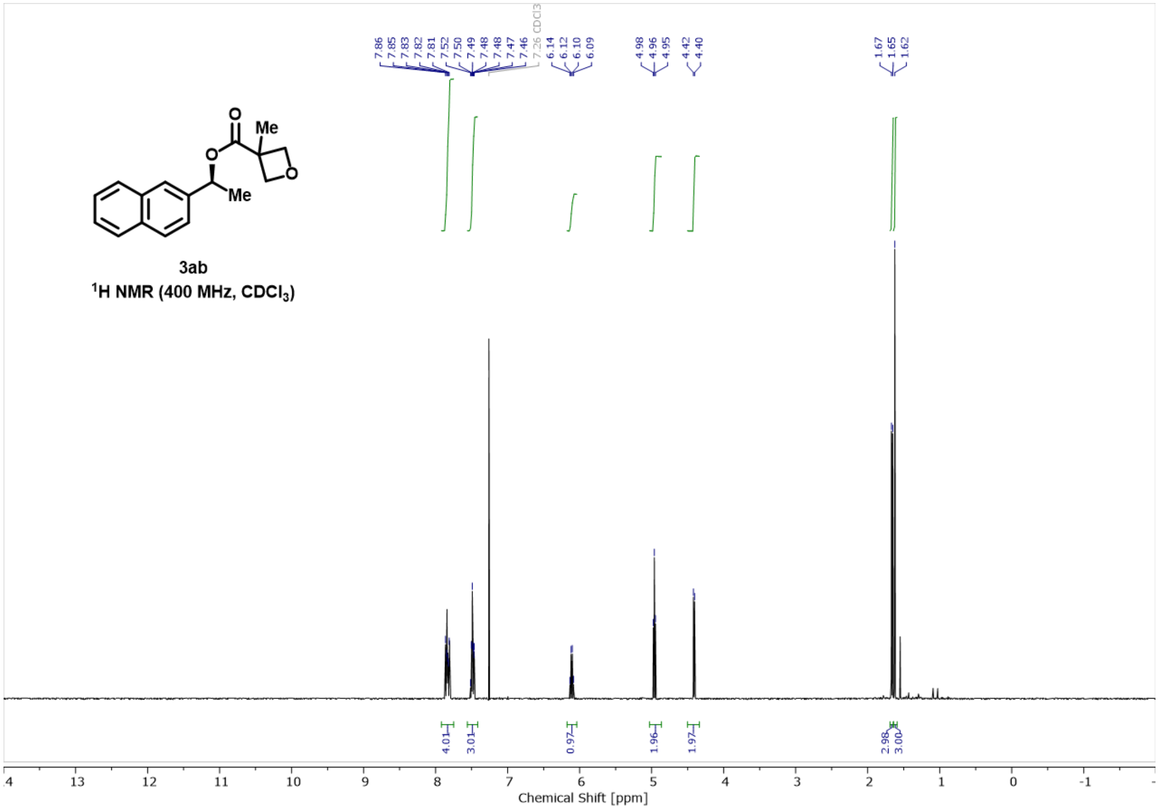


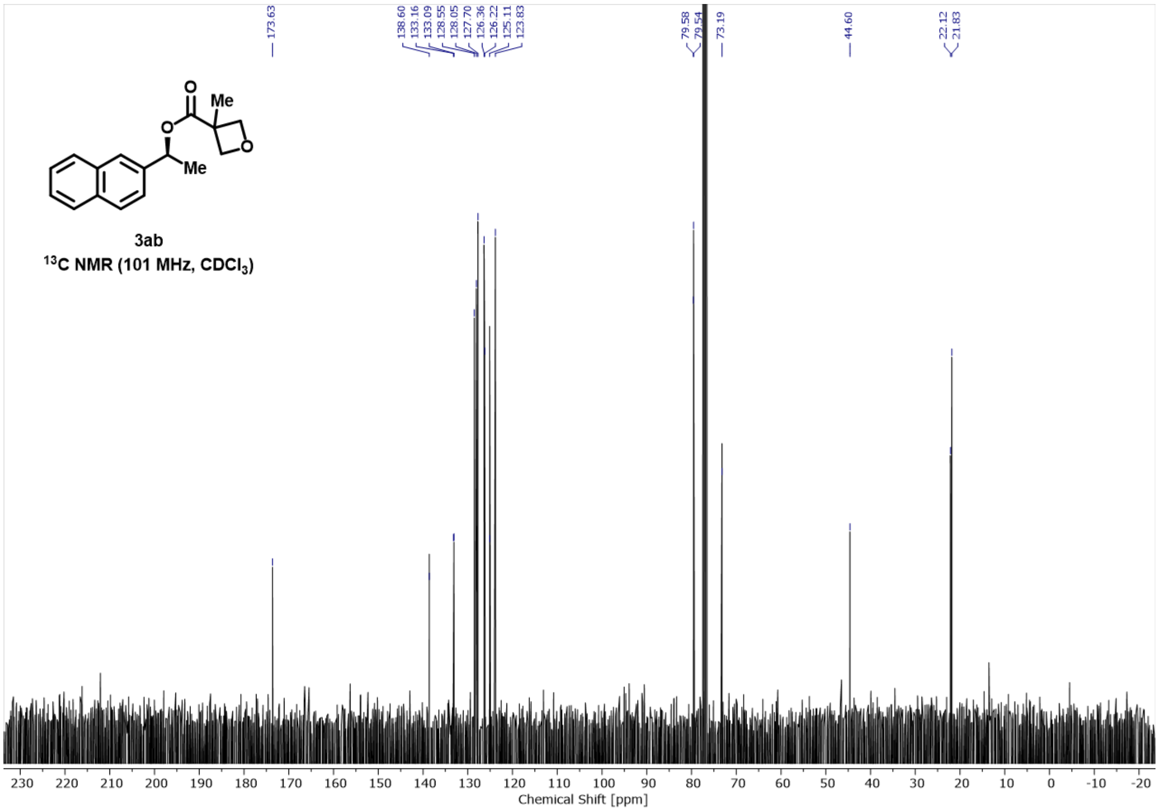

(*S*)-1-(naphthalen-2-yl)ethyl 4-(*N*,*N*-dipropylsulfamoyl)benzoate (**3ac**)

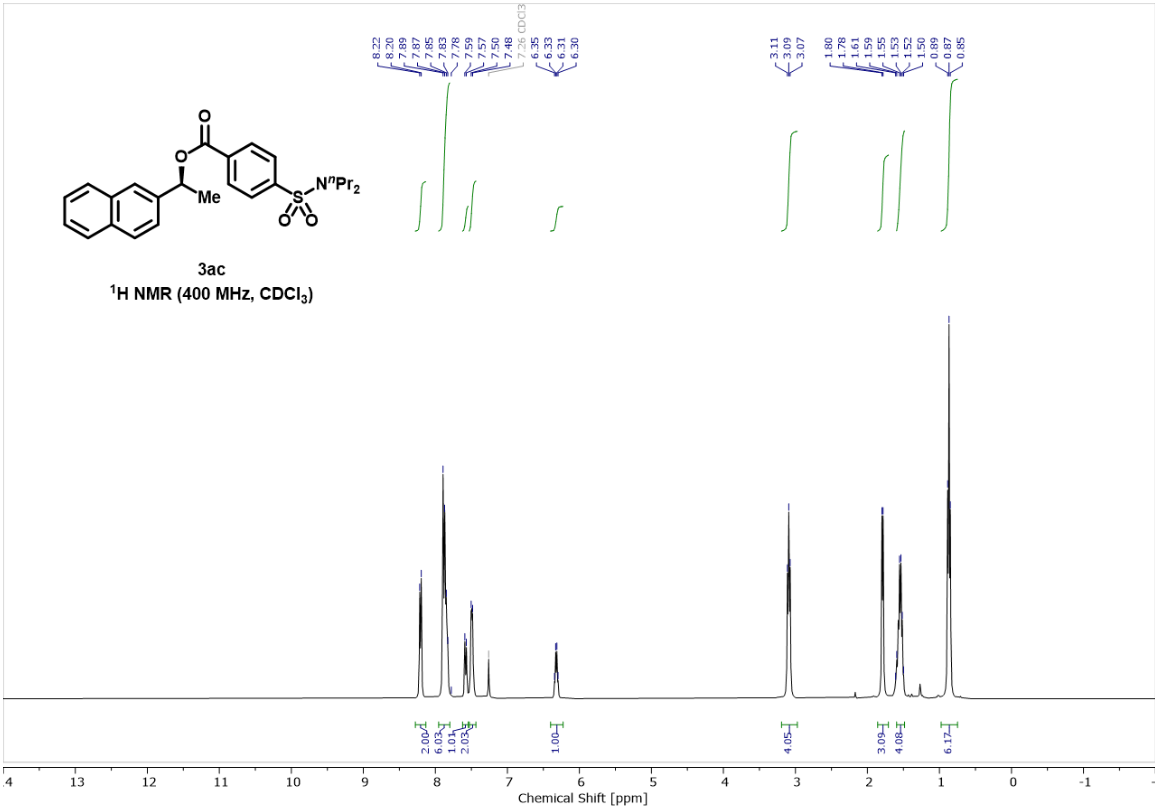


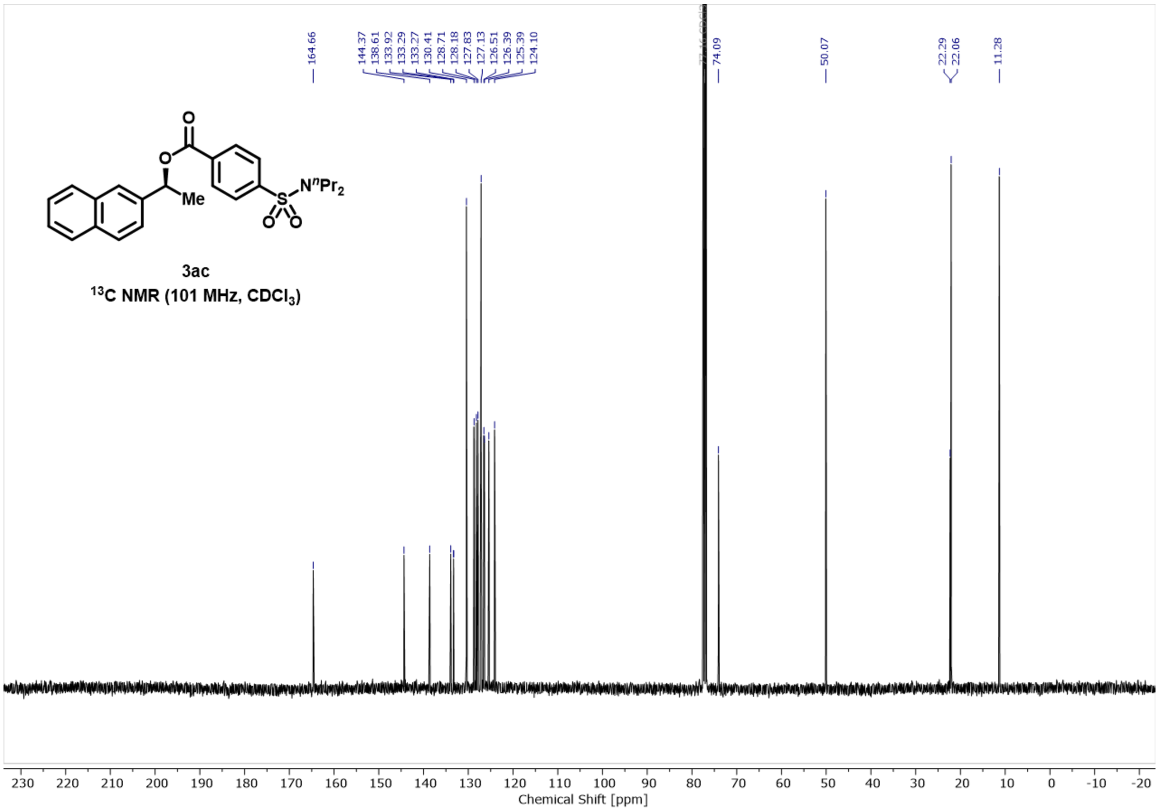

(*S*)-1-(naphthalen-2-yl)ethyl 2-(3-cyano-4-isobutoxyphenyl)-4-methylthiazole-5-carboxylate (**3ad**)

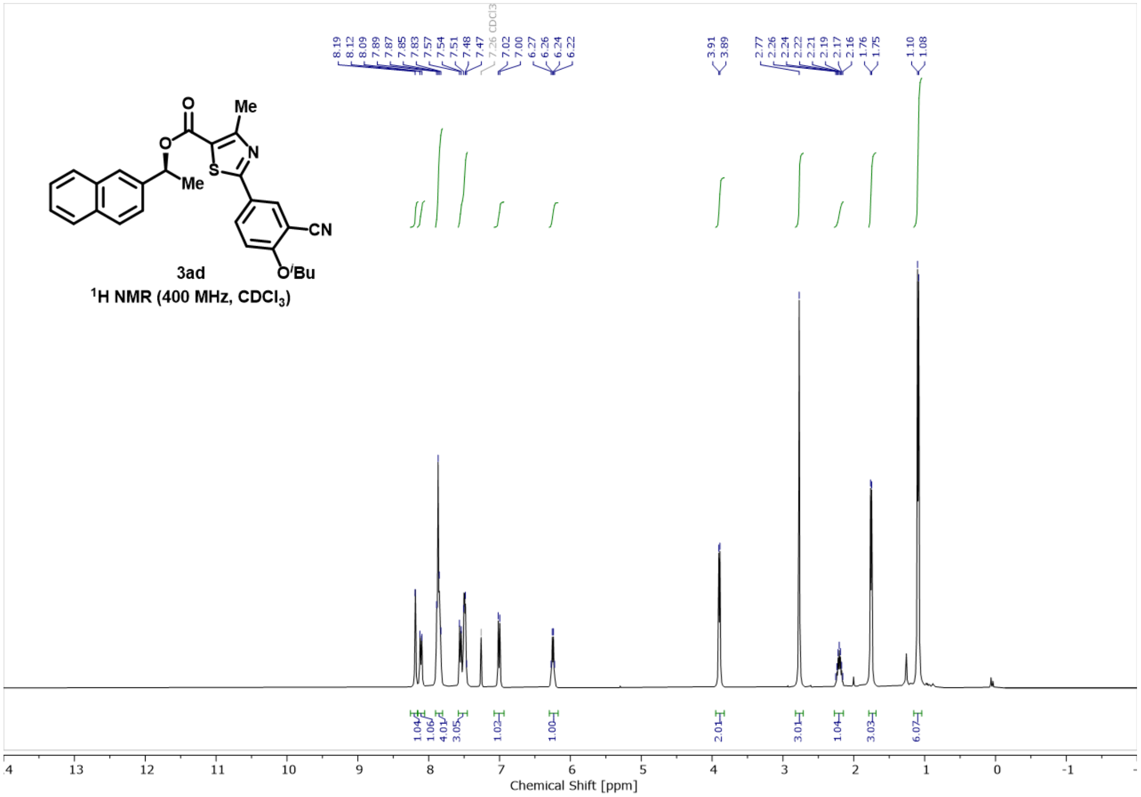


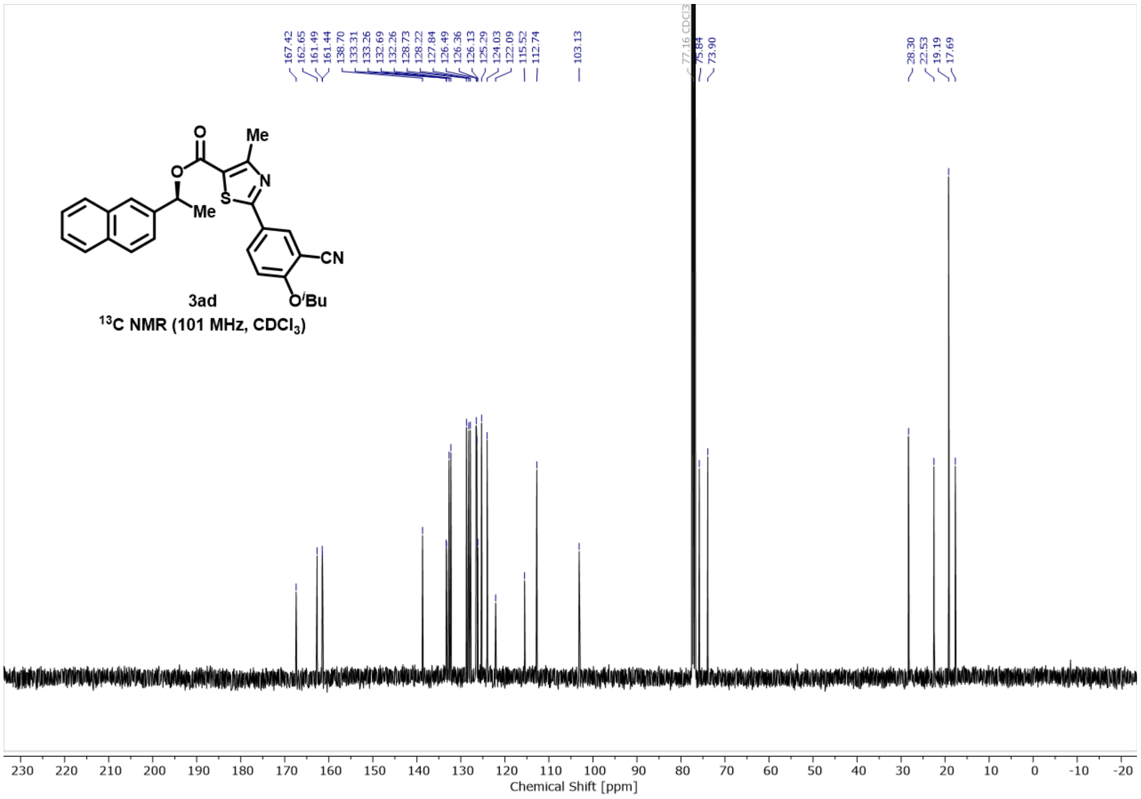

(*S*)-1-(naphthalen-2-yl)ethyl 2-(4-(2-(4-chlorobenzamido)ethyl)phenoxy)-2-methylpropanoate (**3ae**)

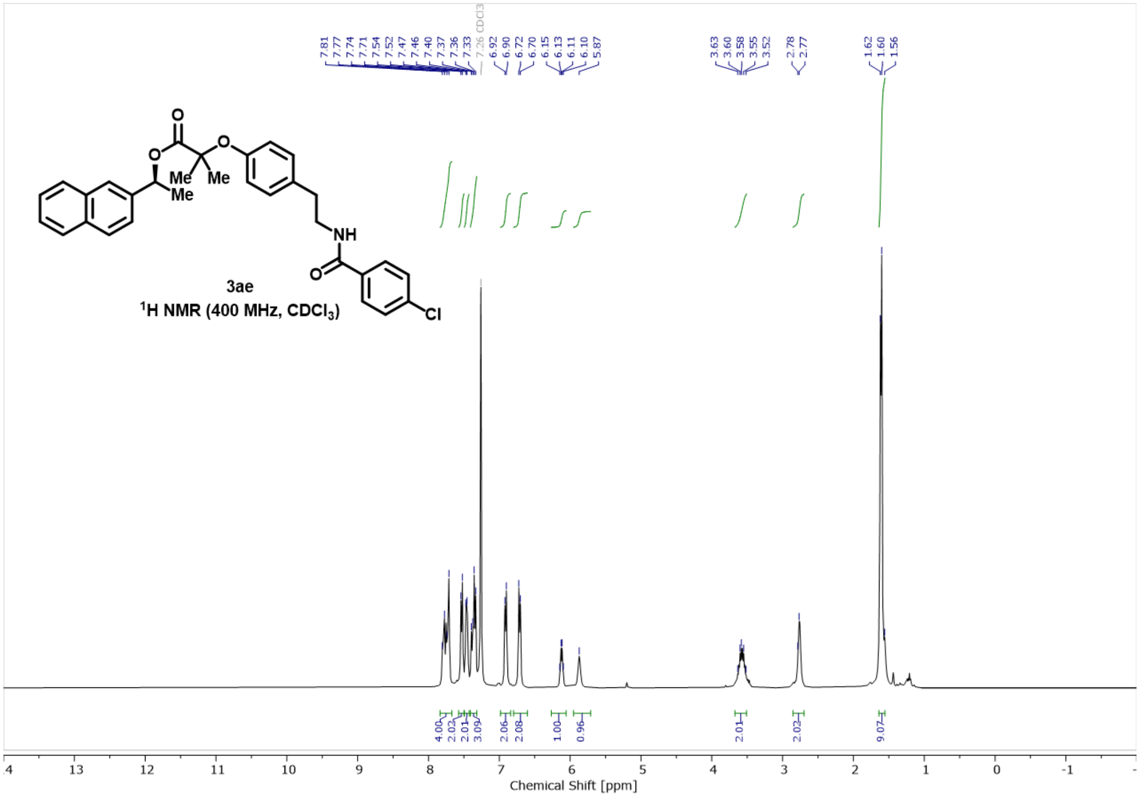


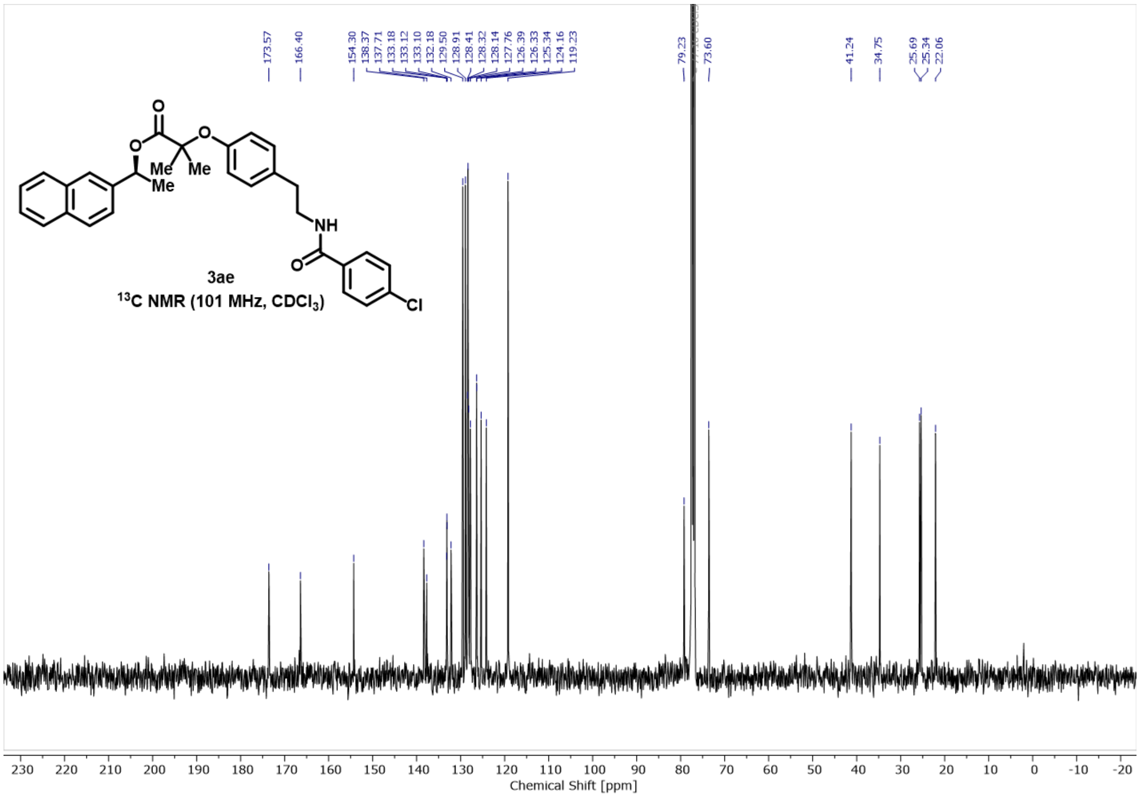

(*S*)-1-(naphthalen-2-yl)ethyl 3-(4,5-diphenyloxazol-2-yl)propanoate (**3af**)

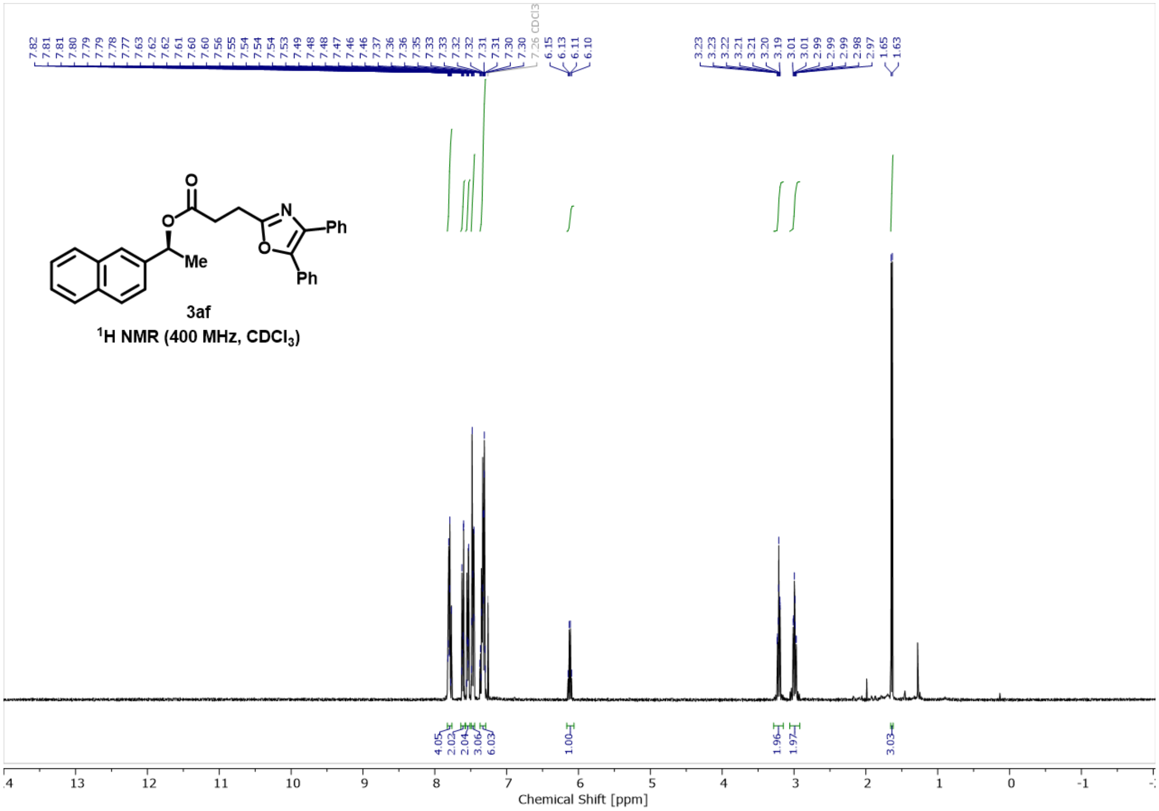


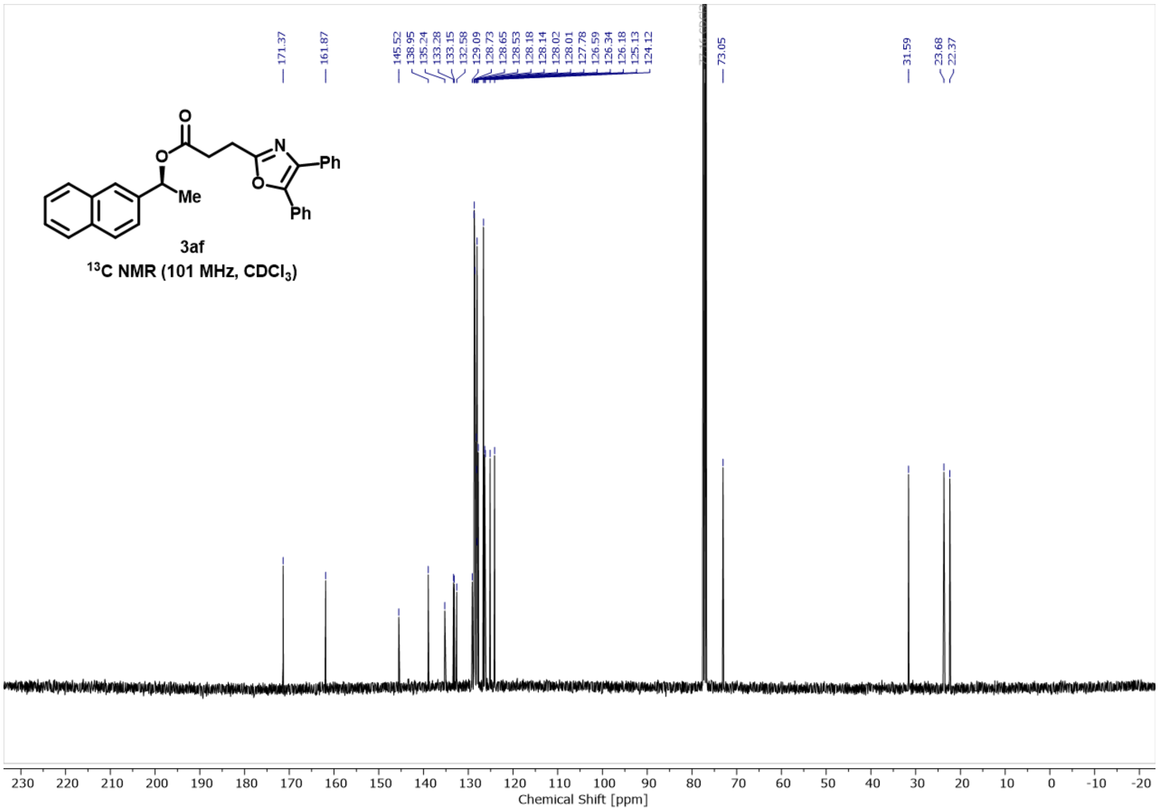

(*S*)-1-(naphthalen-2-yl)ethyl 4-(4-(bis(2-chloroethyl)amino)phenyl)butanoate (**3ag**)

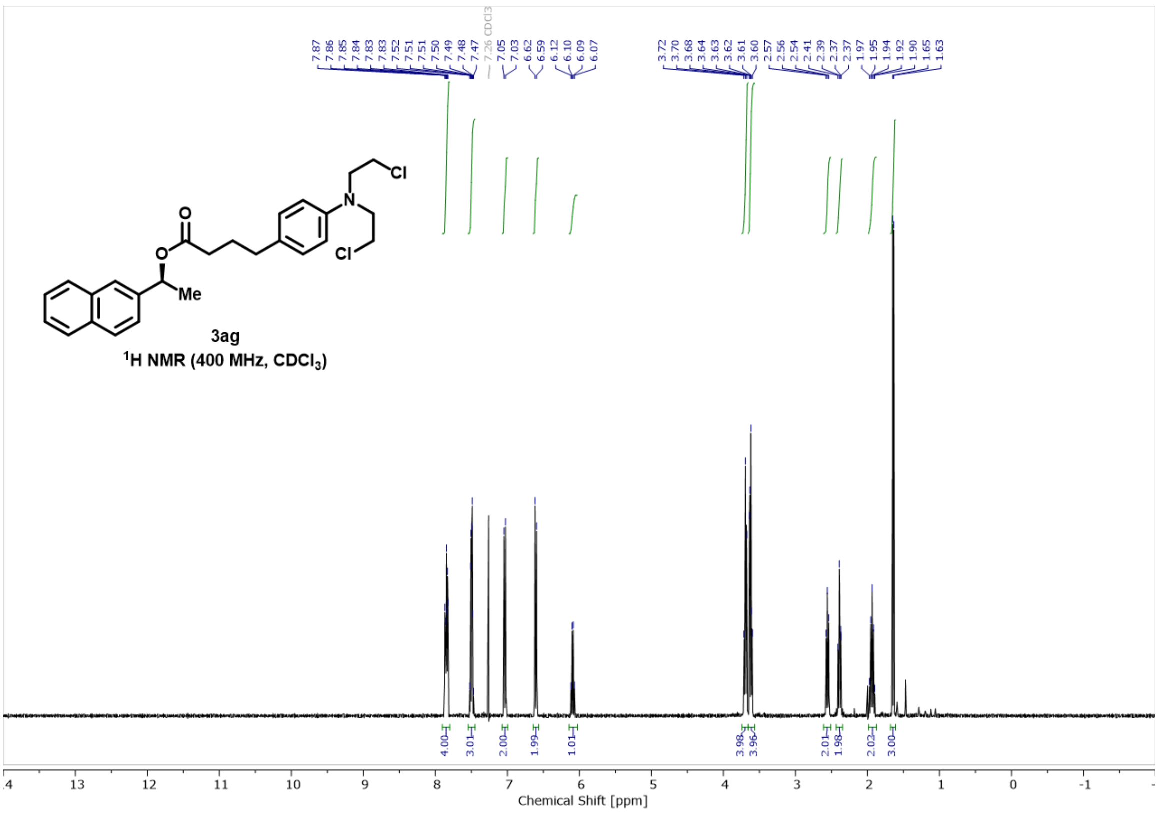


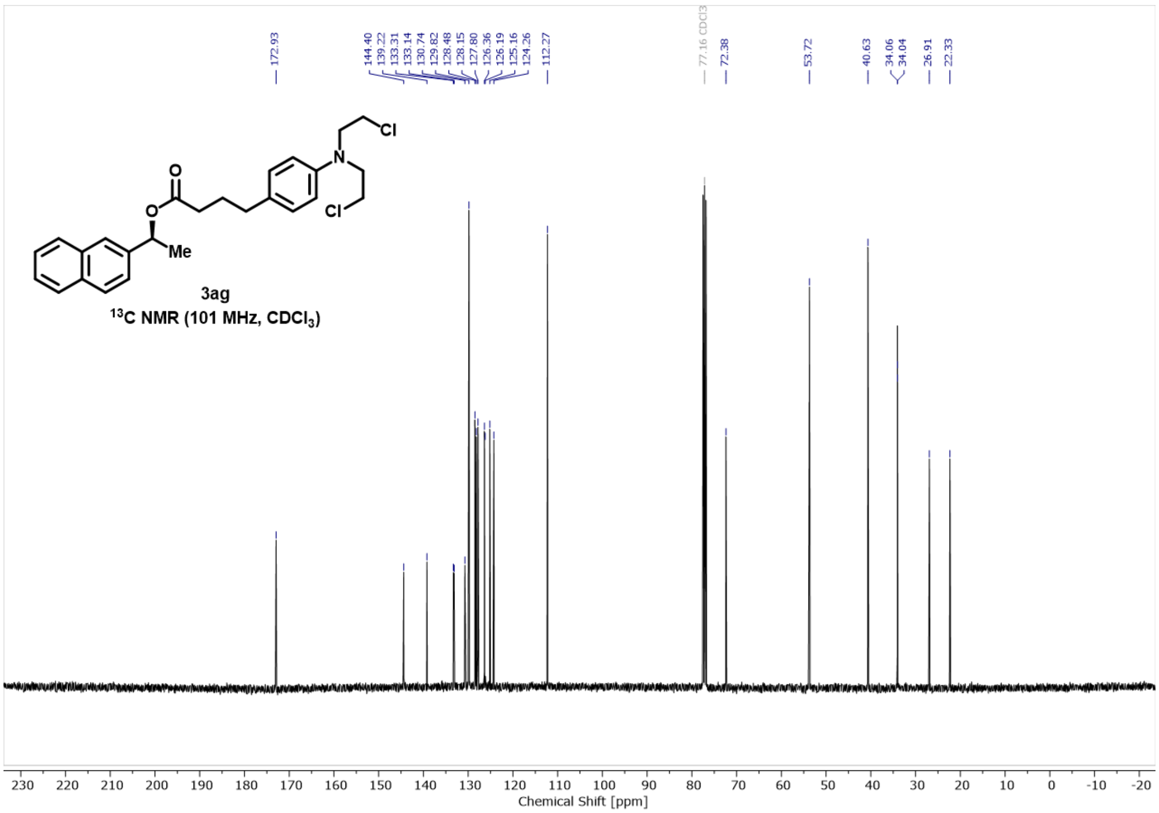

(*S*)-1-(naphthalen-2-yl)ethyl 2-(1-methyl-5-(4-methylbenzoyl)-1*H*-pyrrol-2-yl)acetate (**3ah**)

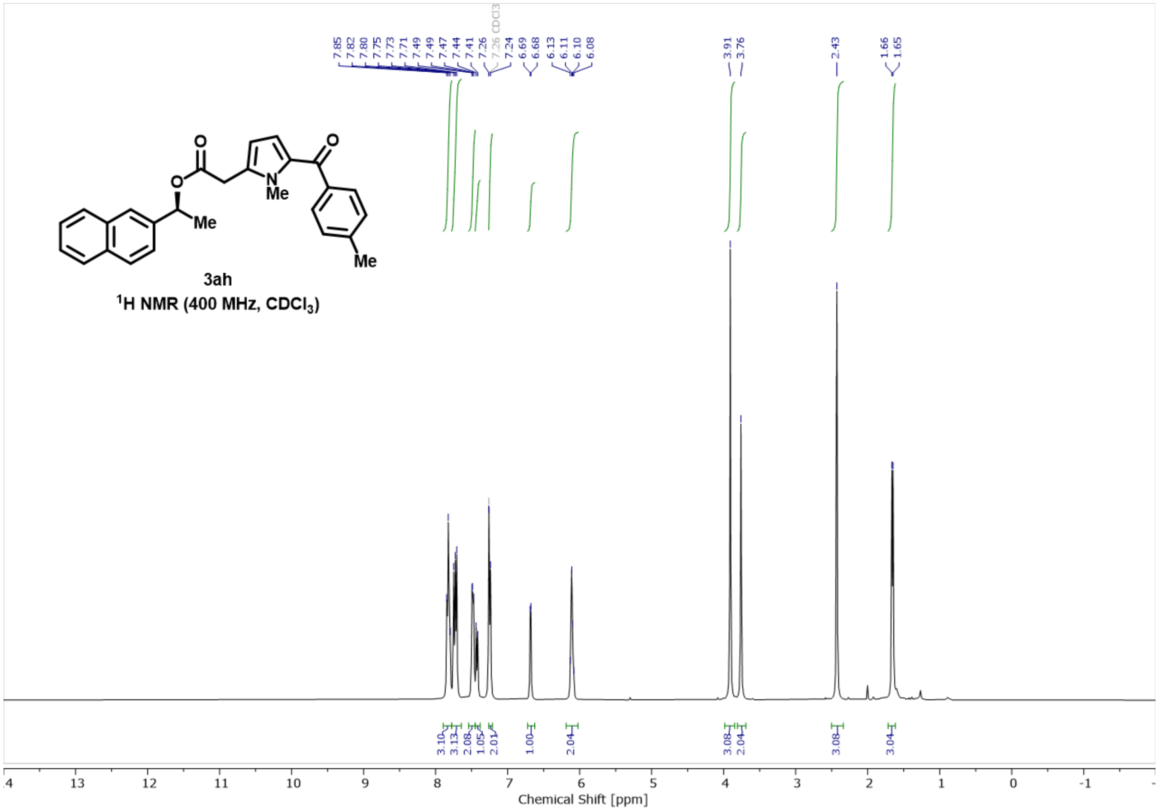


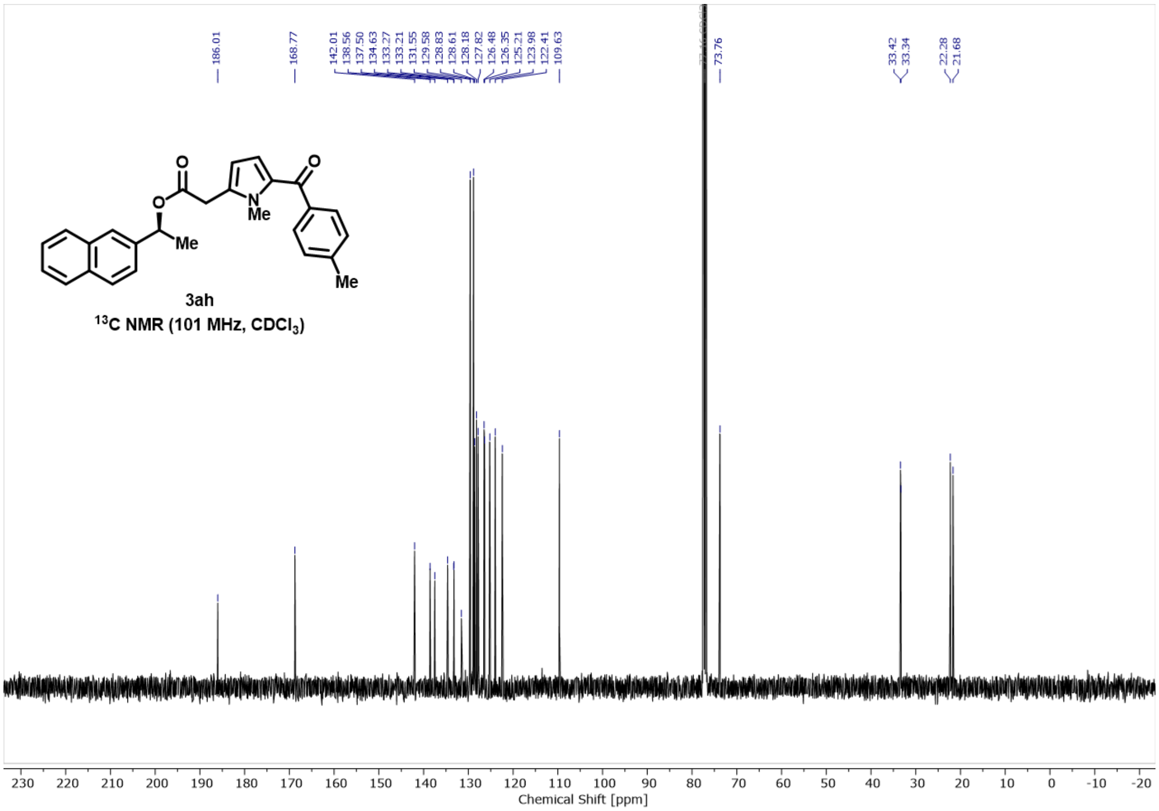

(*S*)-1-(naphthalen-2-yl)ethyl 2-(1-(4-chlorobenzoyl)-2-methyl-1*H*-indol-3-yl)acetate (**3ai**)

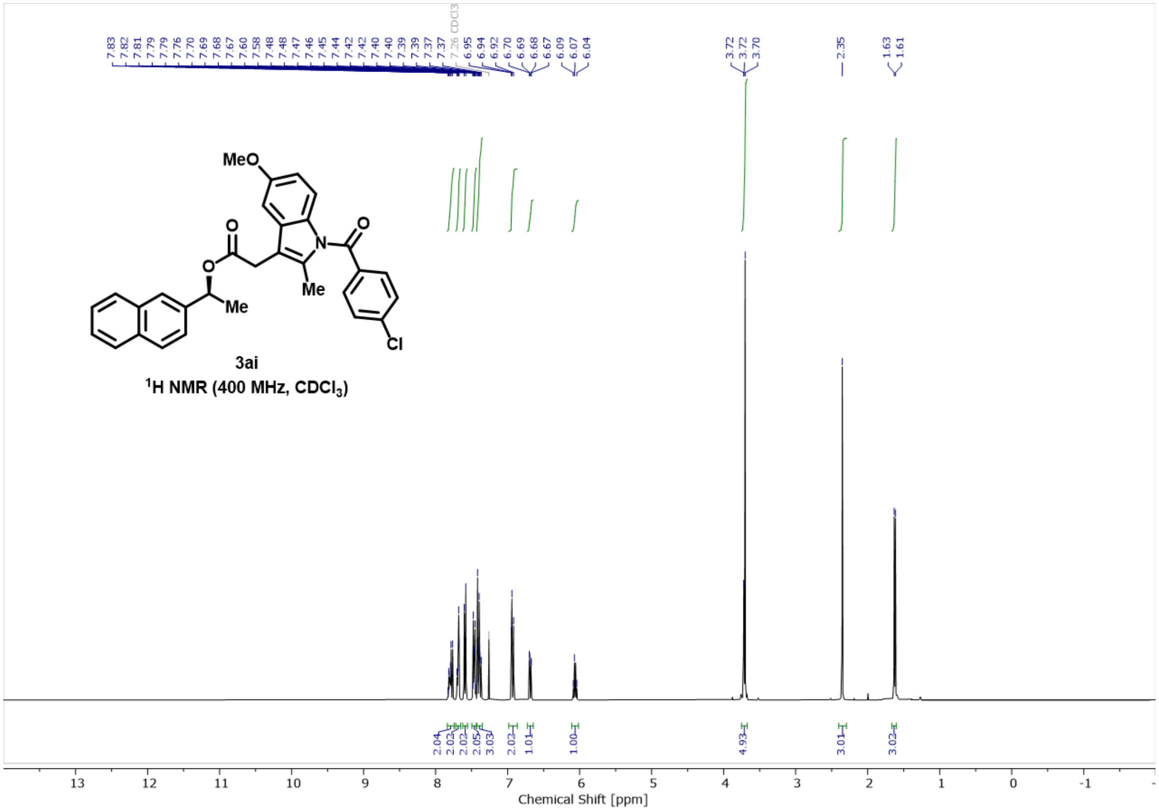


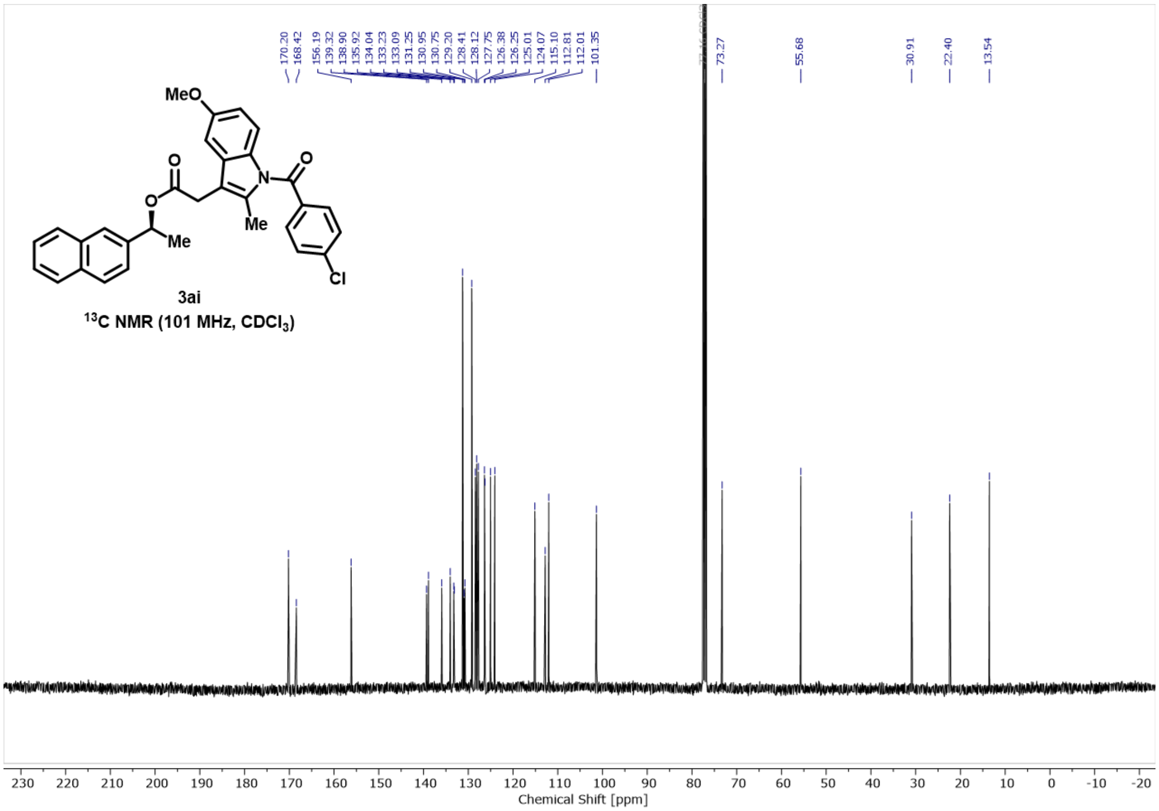

(*S*)-1-(naphthalen-2-yl)ethyl 5-(2,5-dimethylphenoxy)-2,2-dimethylpentanoate (**3aj**)

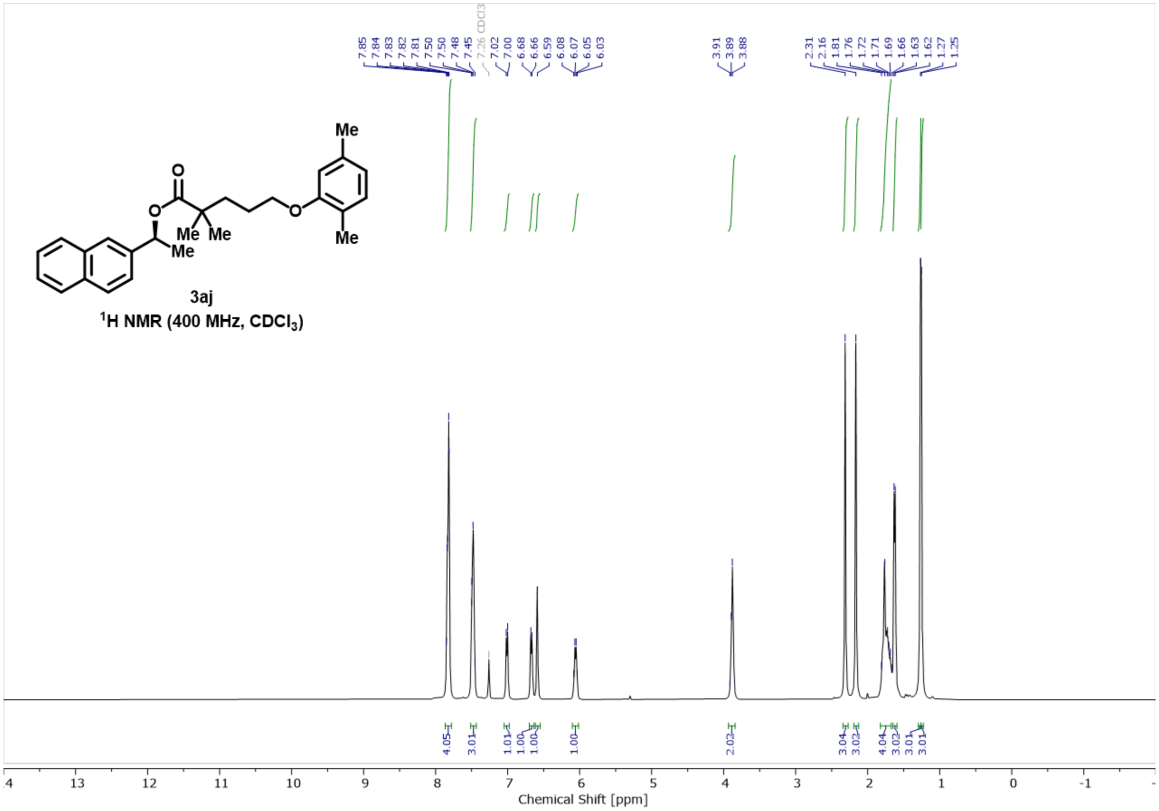


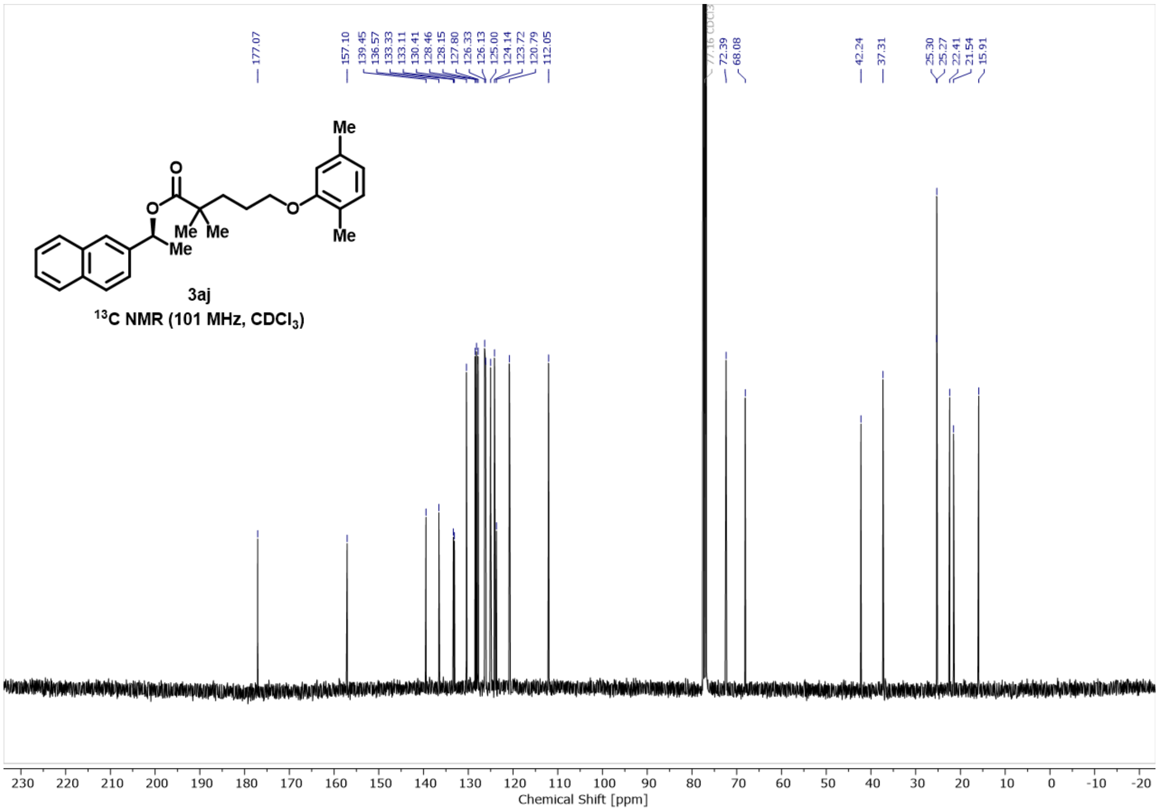

(*S*)-1-(naphthalen-2-yl)ethyl 2-(4-(2,2-dichlorocyclopropyl)phenoxy)-2-methylpropanoate (**3ak**)

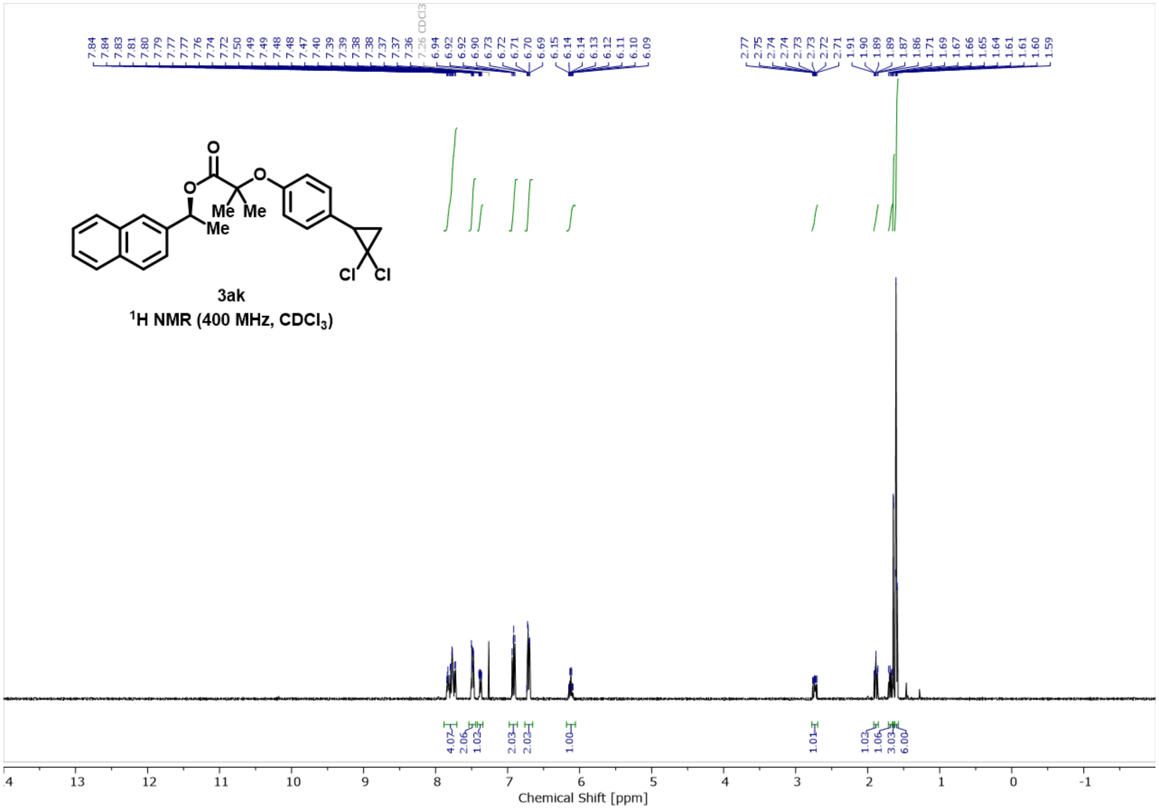


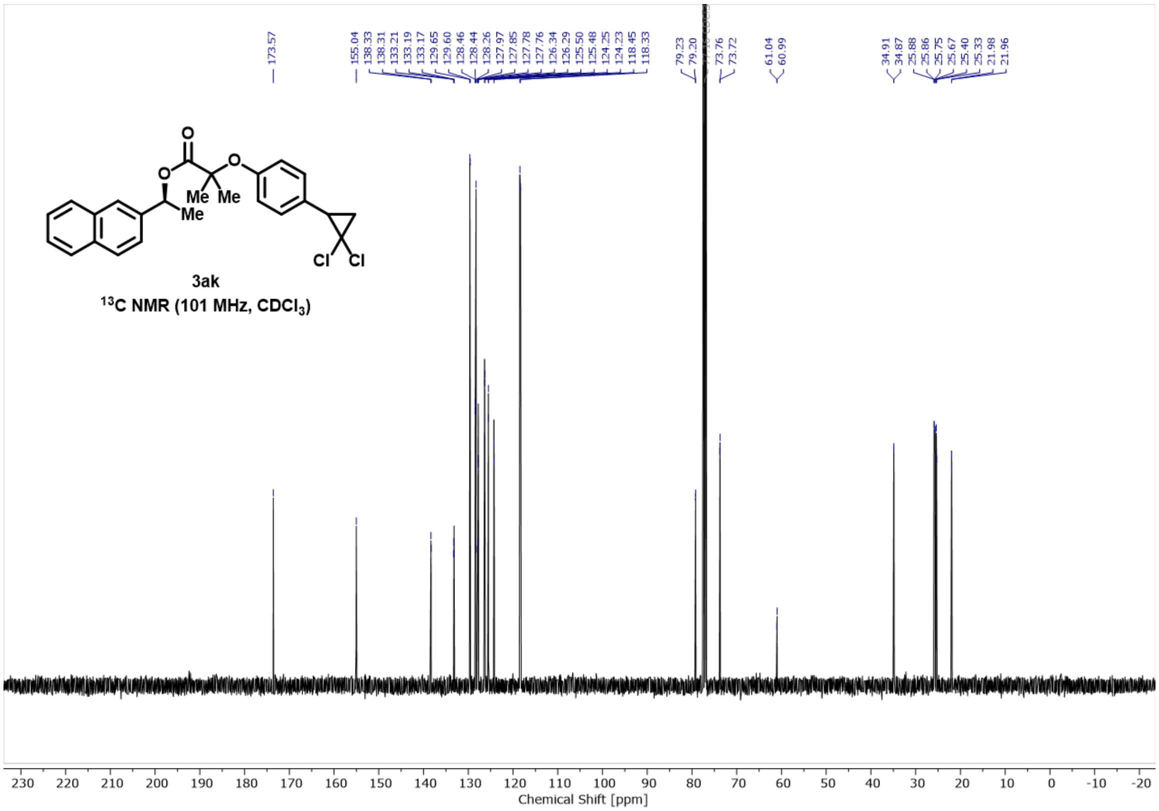

(*S*)-1-(naphthalen-2-yl)ethyl 6-(3-adamantan-1-yl-4-methoxyphenyl)-2-naphthoate (**3al**)

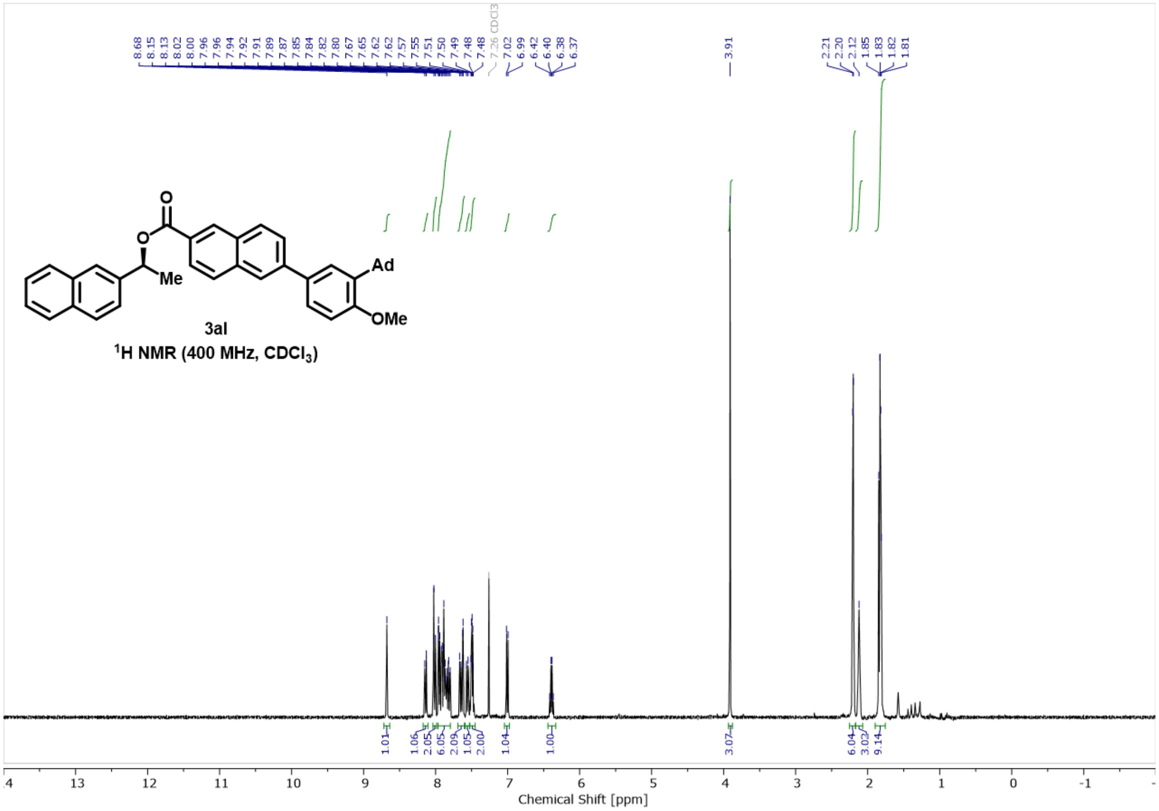


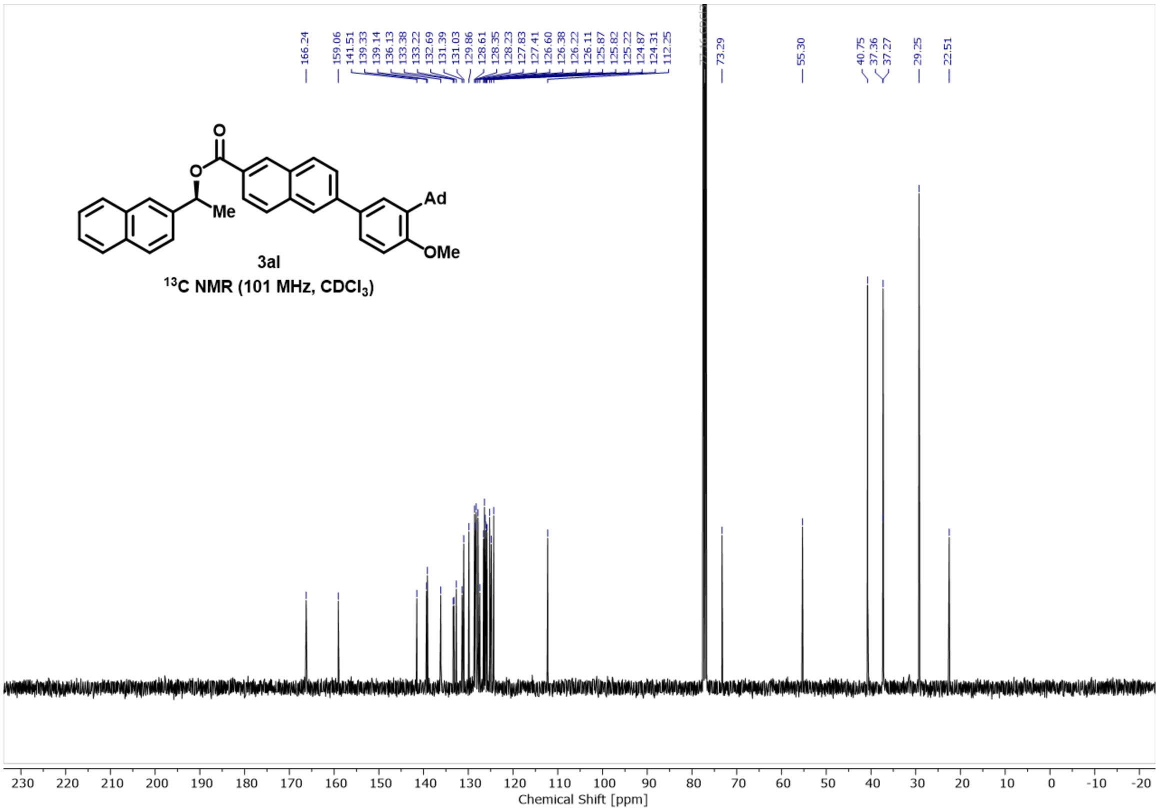

*Hammett Analysis Characterization Data*

1-(naphthalen-2-yl)ethyl 4-(trifluoromethyl)benzoate (**3a**$_{CF3}$)

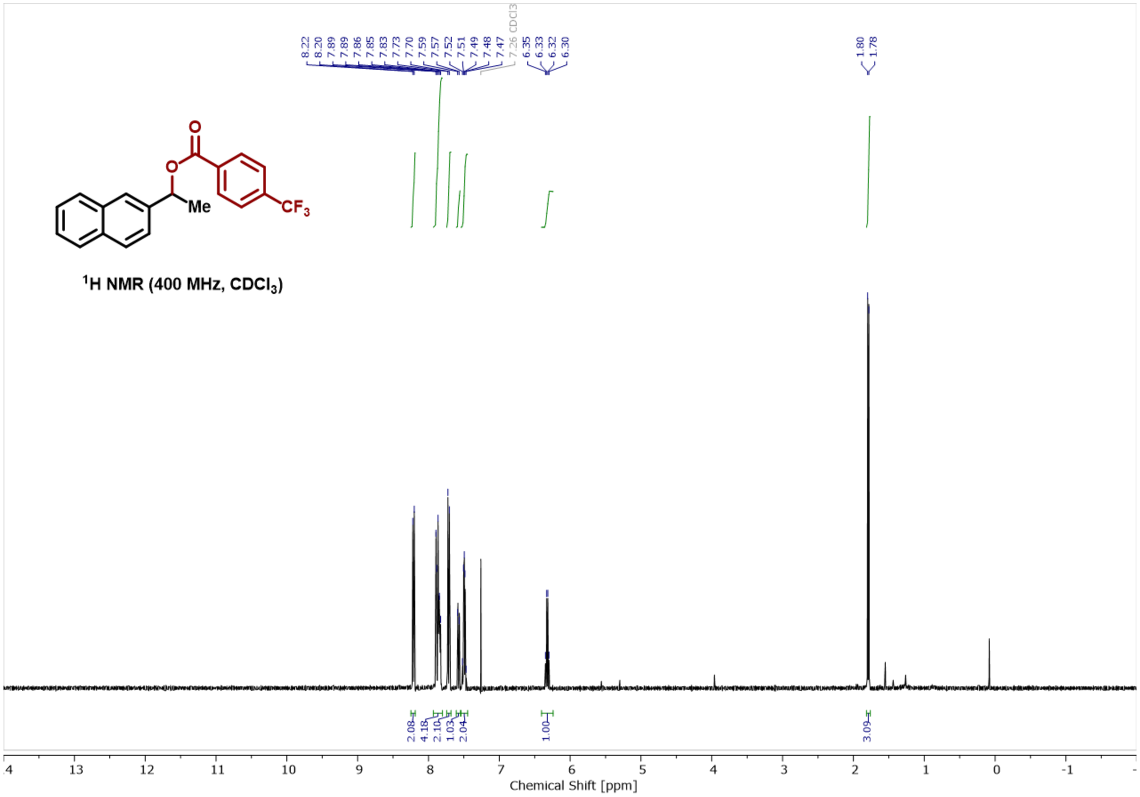


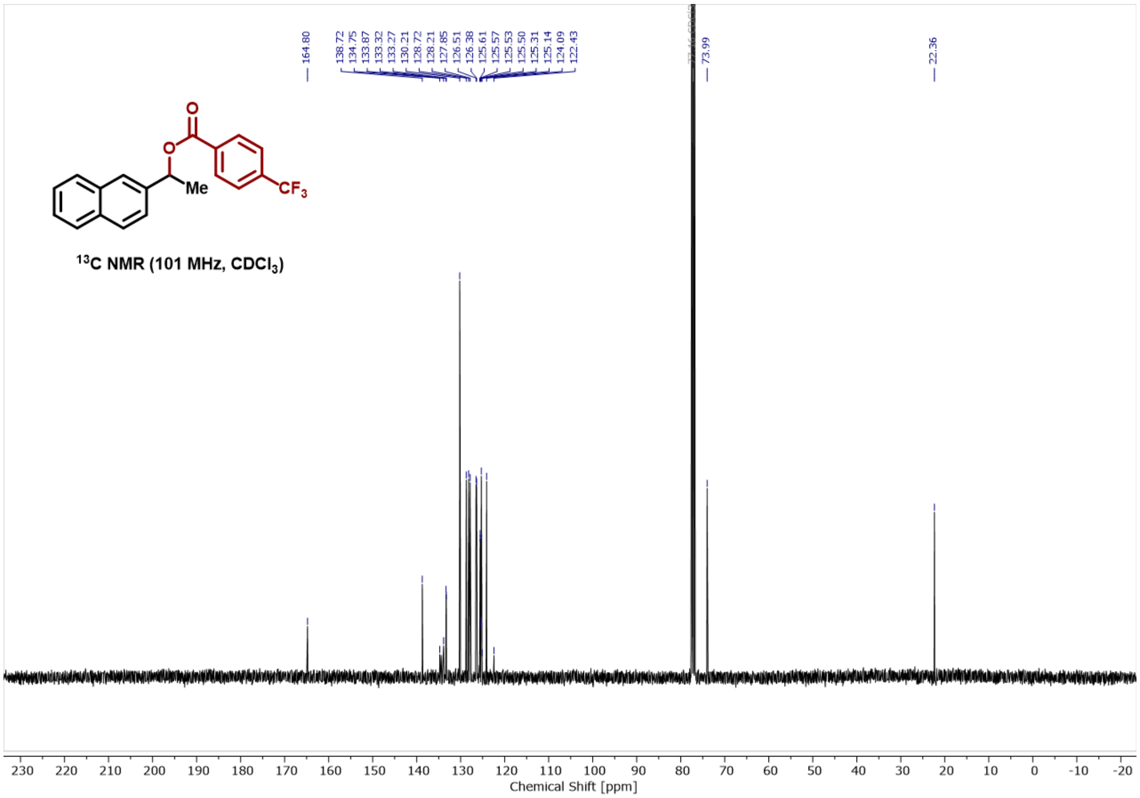

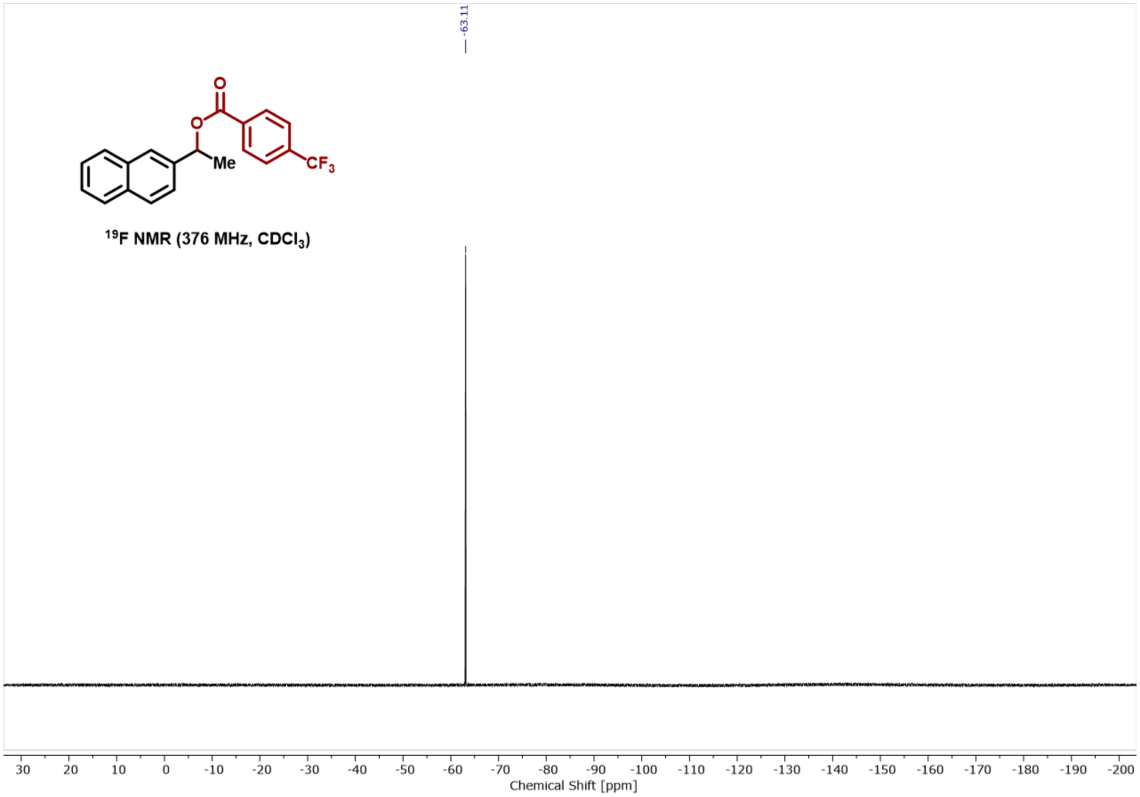

-63.11
Me
CF3
O
O
19F NMR (376 MHz, CDCl3)
30 20 10 0 -10 -20 -30 -40 -50 -60 -70 -80 -90 -100 -110 -120 -130 -140 -150 -160 -170 -180 -190 -200
Chemical Shift [ppm]

1-(naphthalen-2-yl)ethyl 4-chlorobenzoate (**3a**Cl)

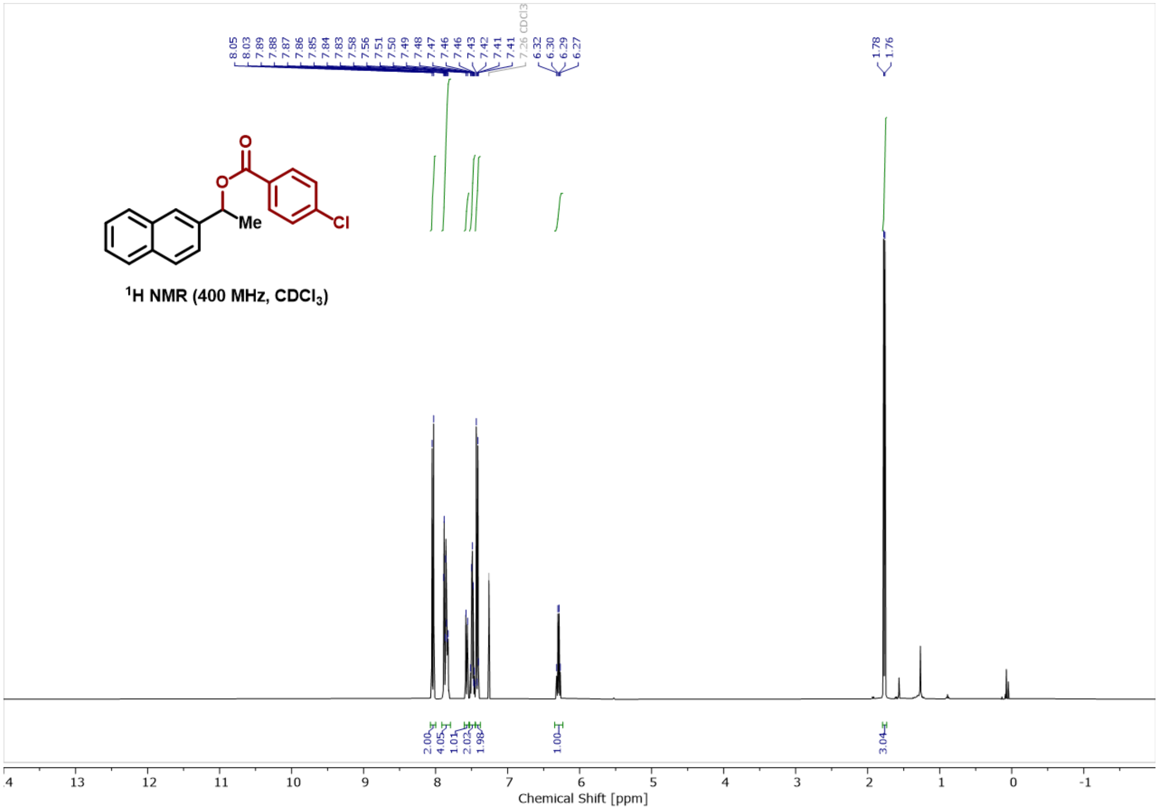


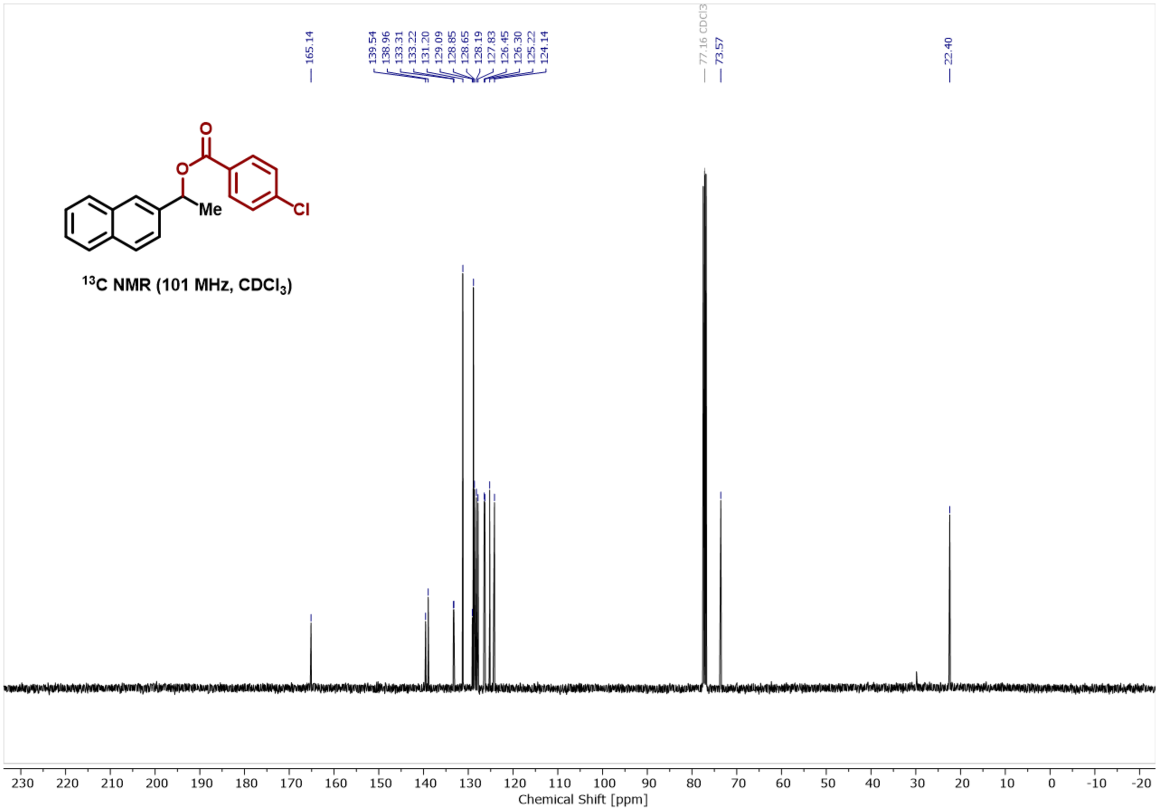

1-(naphthalen-2-yl)ethyl 4-fluorobenzoate (**3a$_F$**)

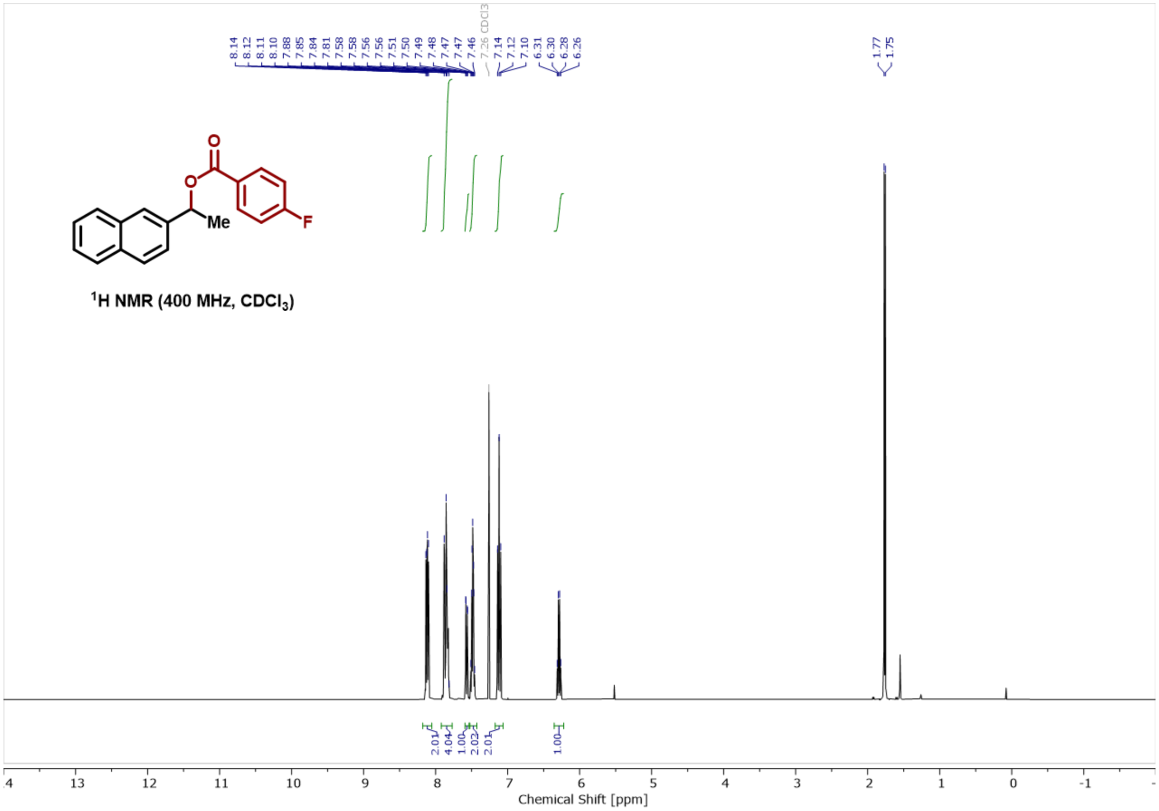


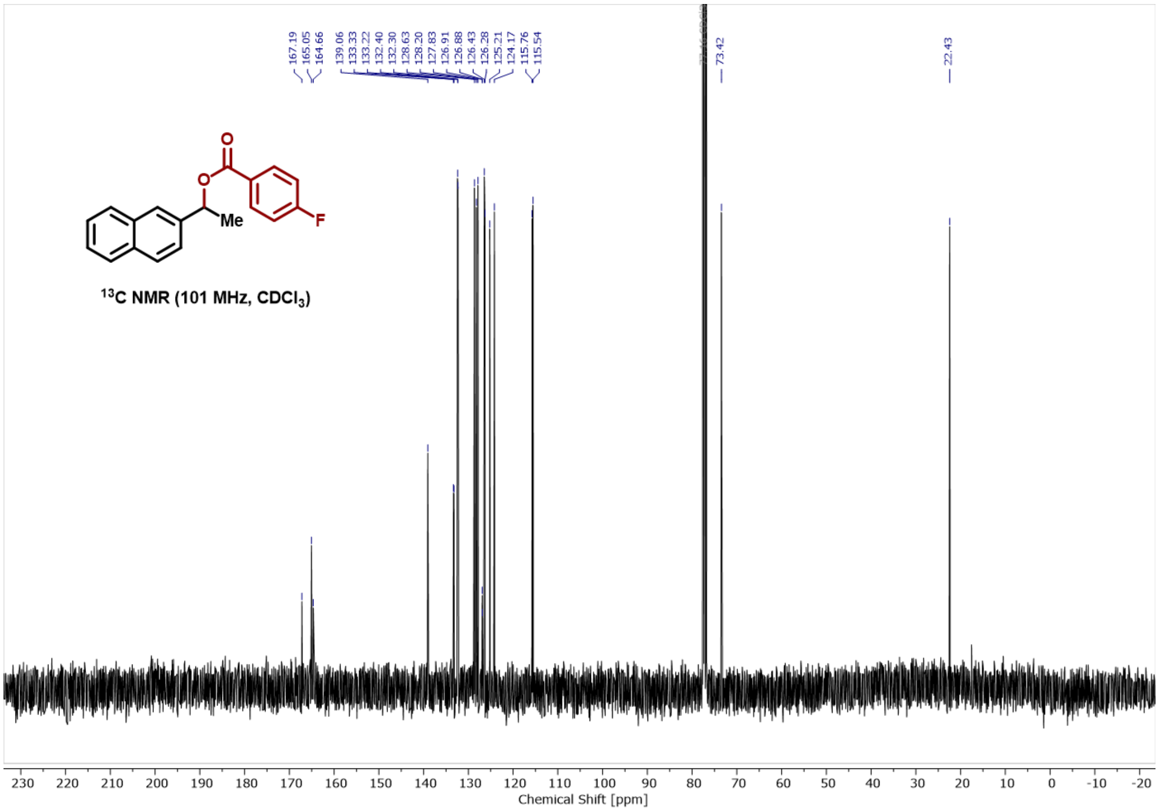

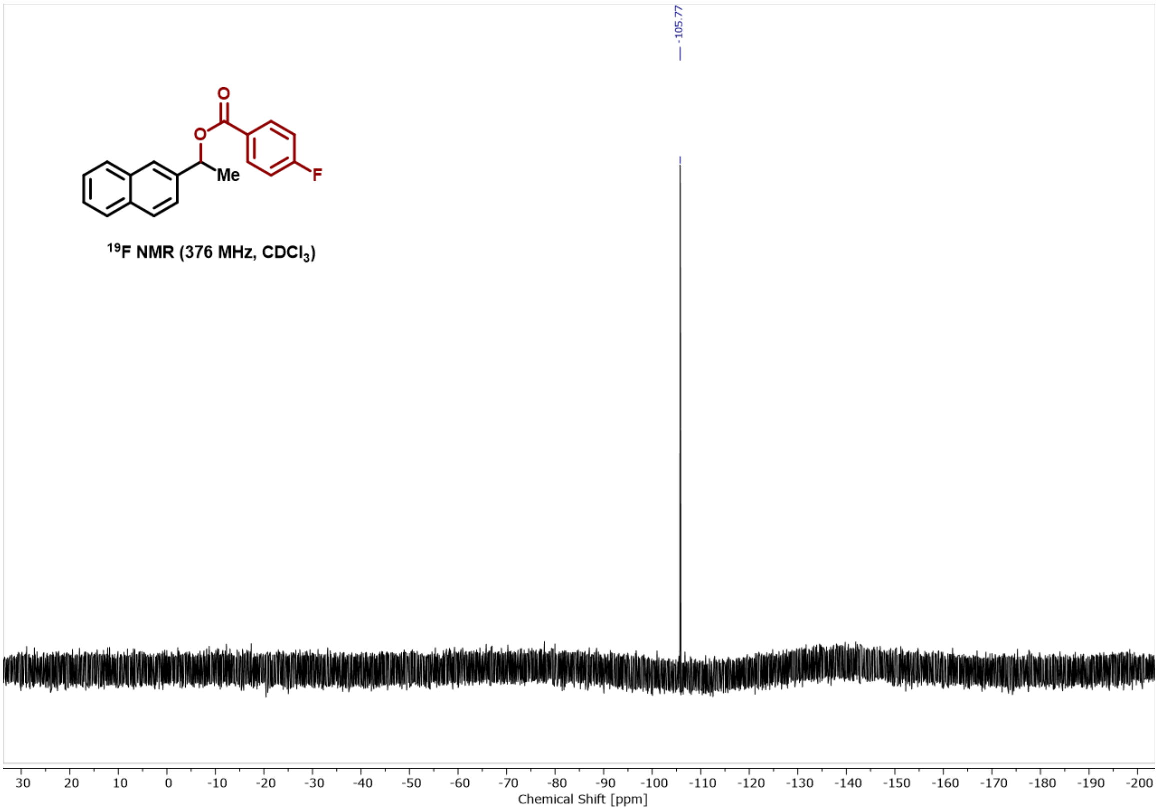
-105.77
Me
F
19F NMR (376 MHz, CDCl3)
30
20
10
0
-10
-20
-30
-40
-50
-60
-70
-80
-90
-100
-110
-120
-130
-140
-150
-160
-170
-180
-190
-200
Chemical Shift [ppm]

1-(naphthalen-2-yl)ethyl 4-methylbenzoate (**3a**$_{Me}$)

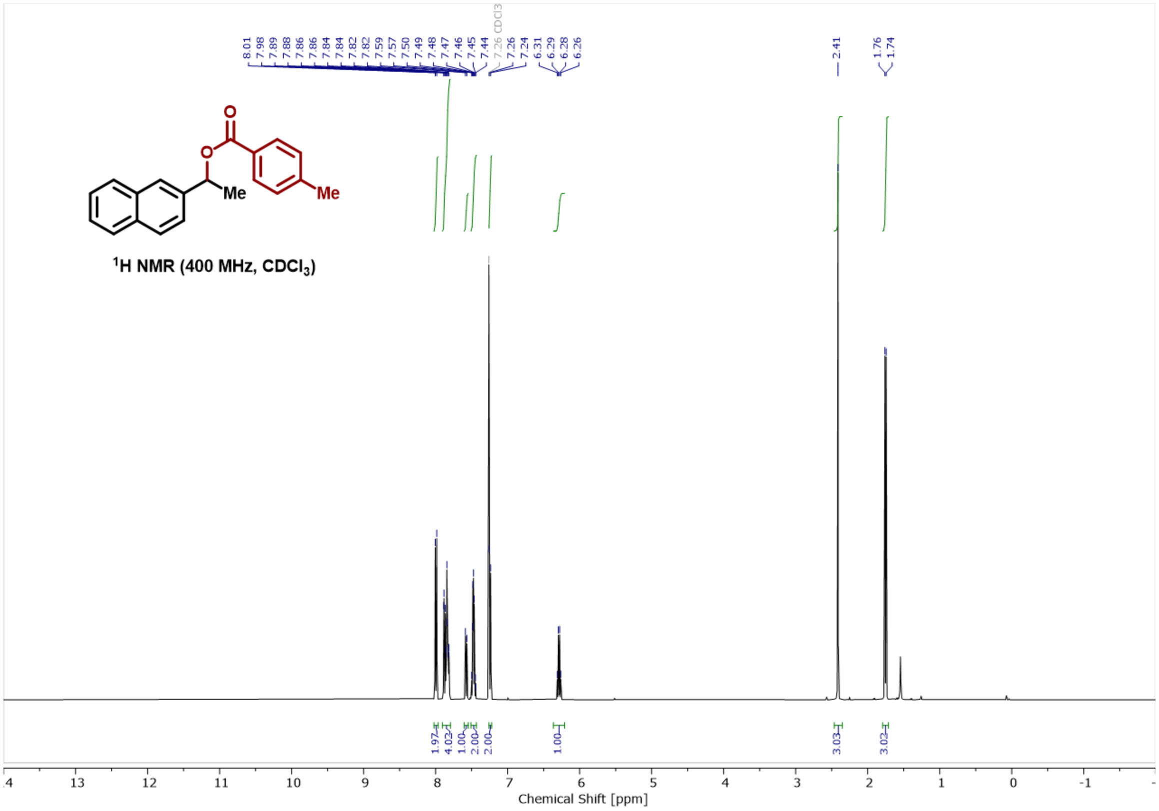


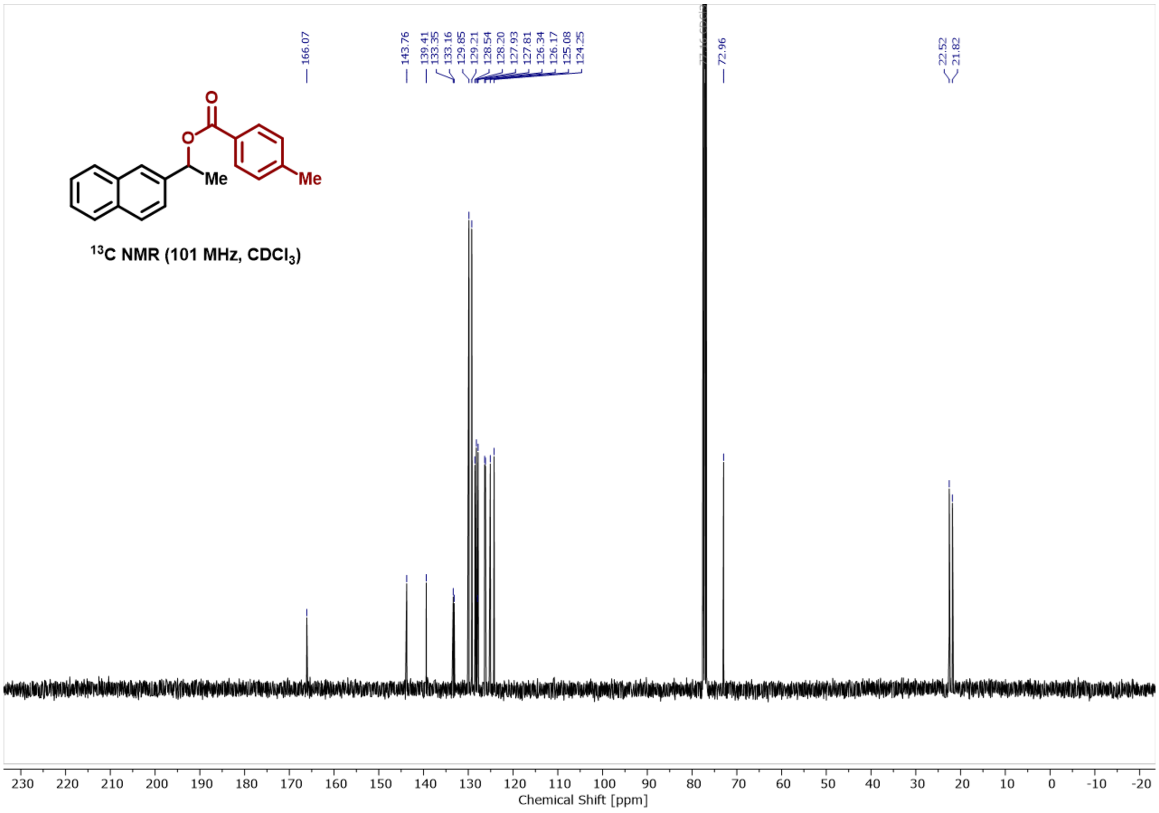

1-(naphthalen-2-yl)ethyl 4-(tert-butyl)benzoate (**3a**$_{\mathbf{tBu}}$)

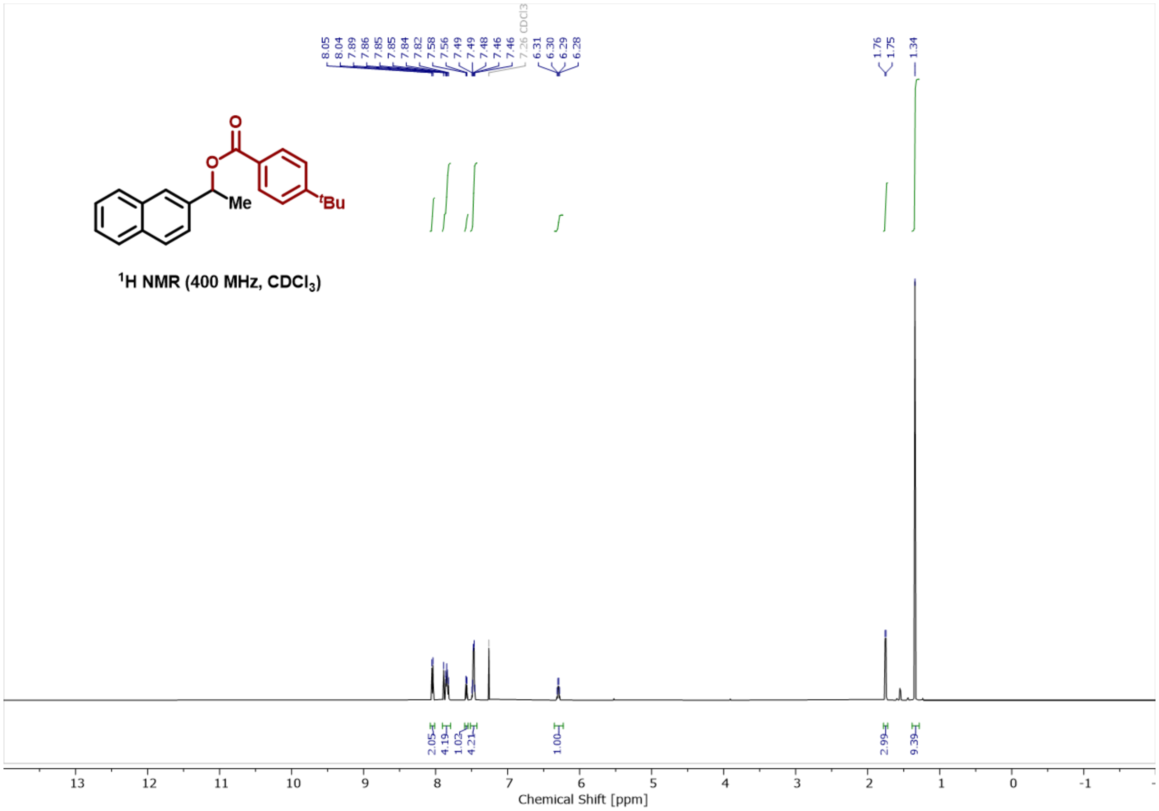


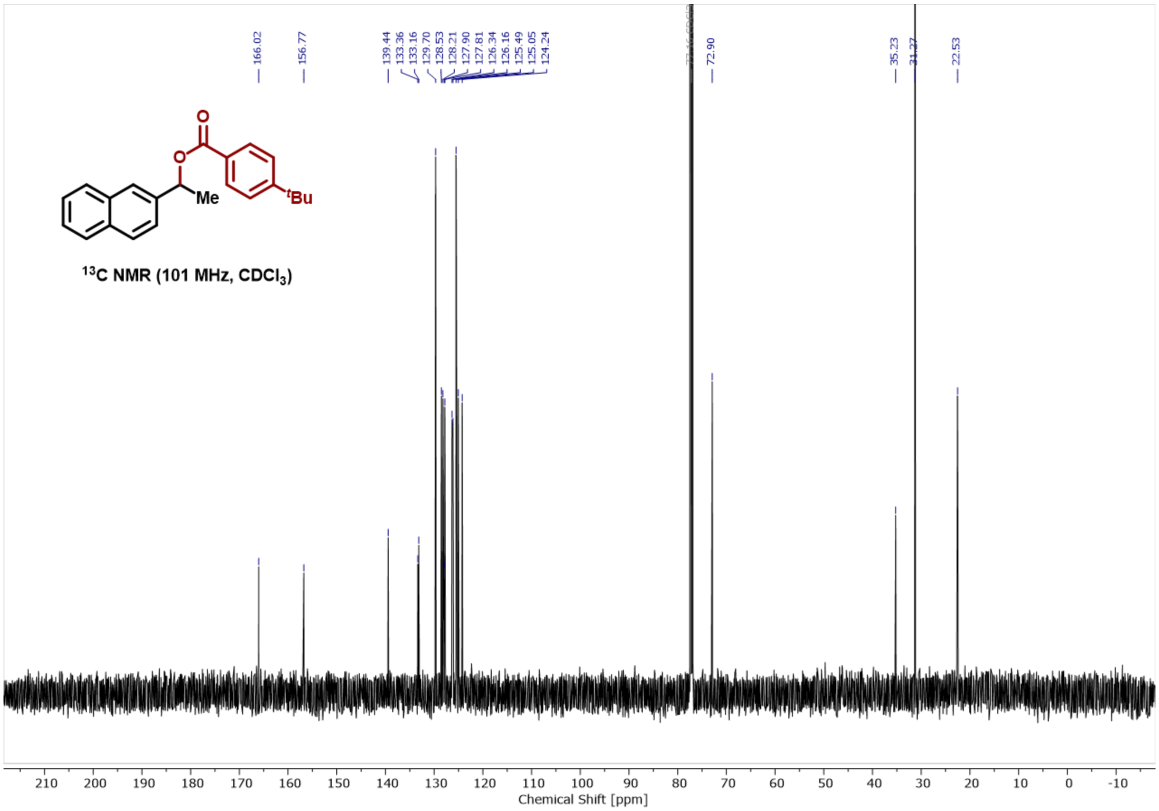

2-(cyclobutyl(4-fluorophenyl)methyl)-4,4,5,5-tetramethyl-1,3,2-dioxaborolane (**1e<sub>F</sub>**)

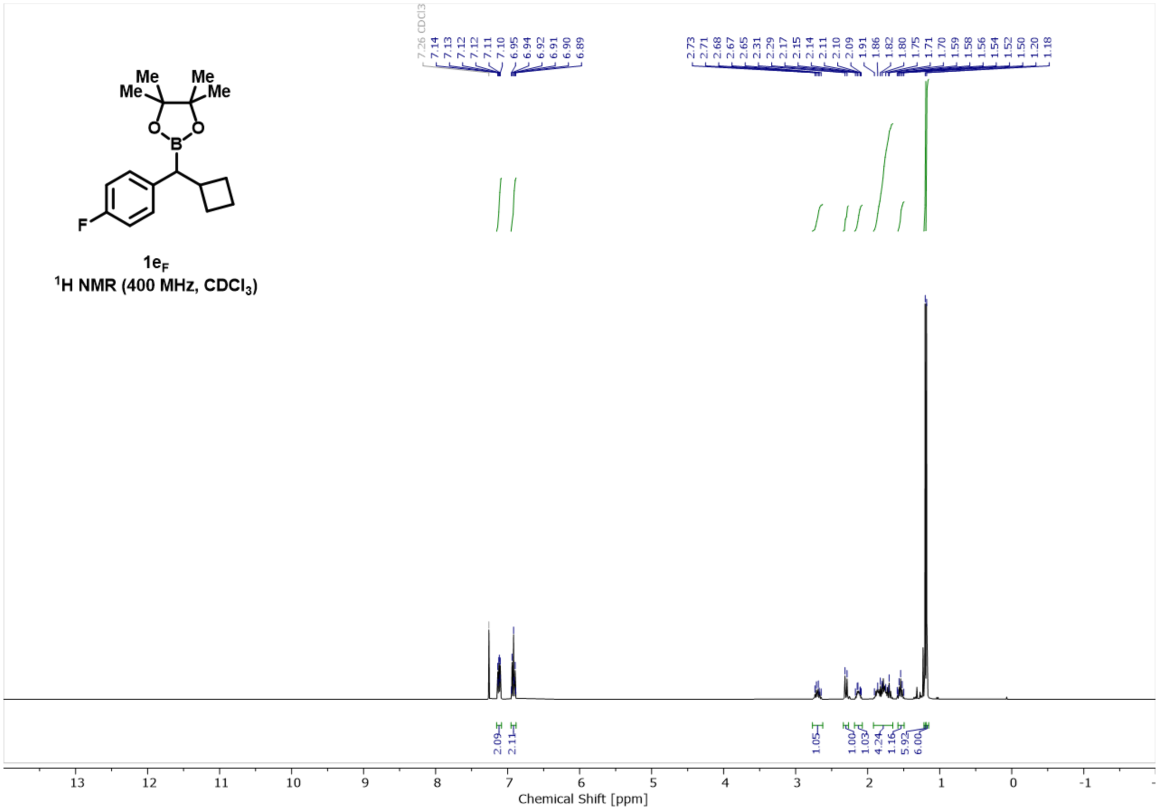


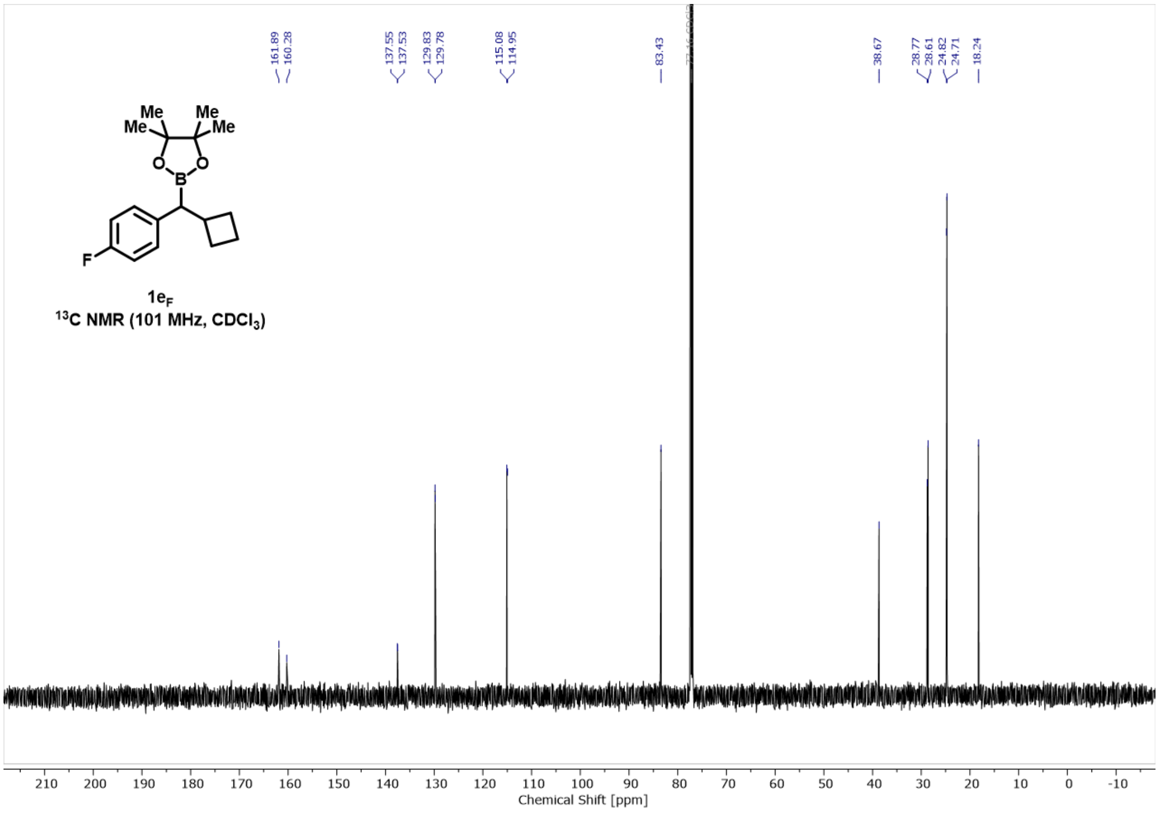

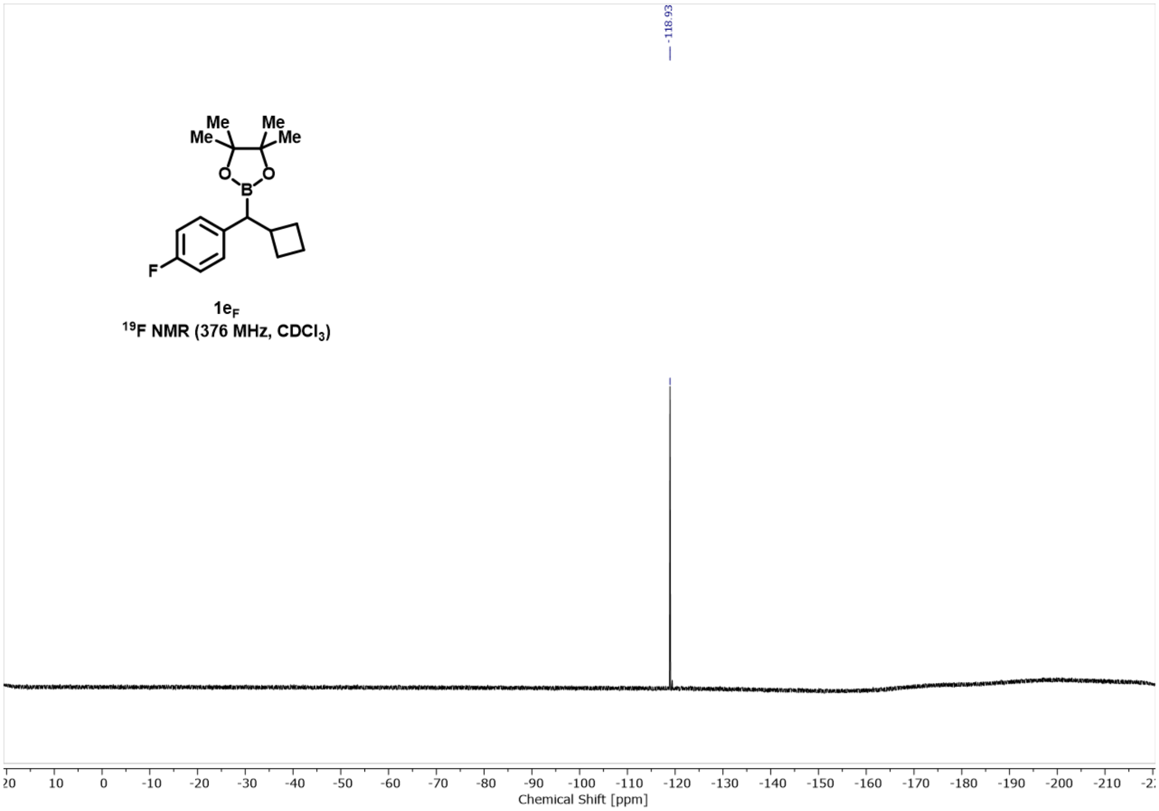

-118.93
Me
Me
Me
Me
O
B
O
F
1e$_F$
$^{19}$F NMR (376 MHz, CDCl$_3$)
20 10 0 -10 -20 -30 -40 -50 -60 -70 -80 -90 -100 -110 -120 -130 -140 -150 -160 -170 -180 -190 -200 -210
Chemical Shift [ppm]

2-([1,1'-biphenyl]-4-yl(cyclobutyl)methyl)-4,4,5,5-tetramethyl-1,3,2-dioxaborolane (**1e**$_{\mathbf{Ph}}$)

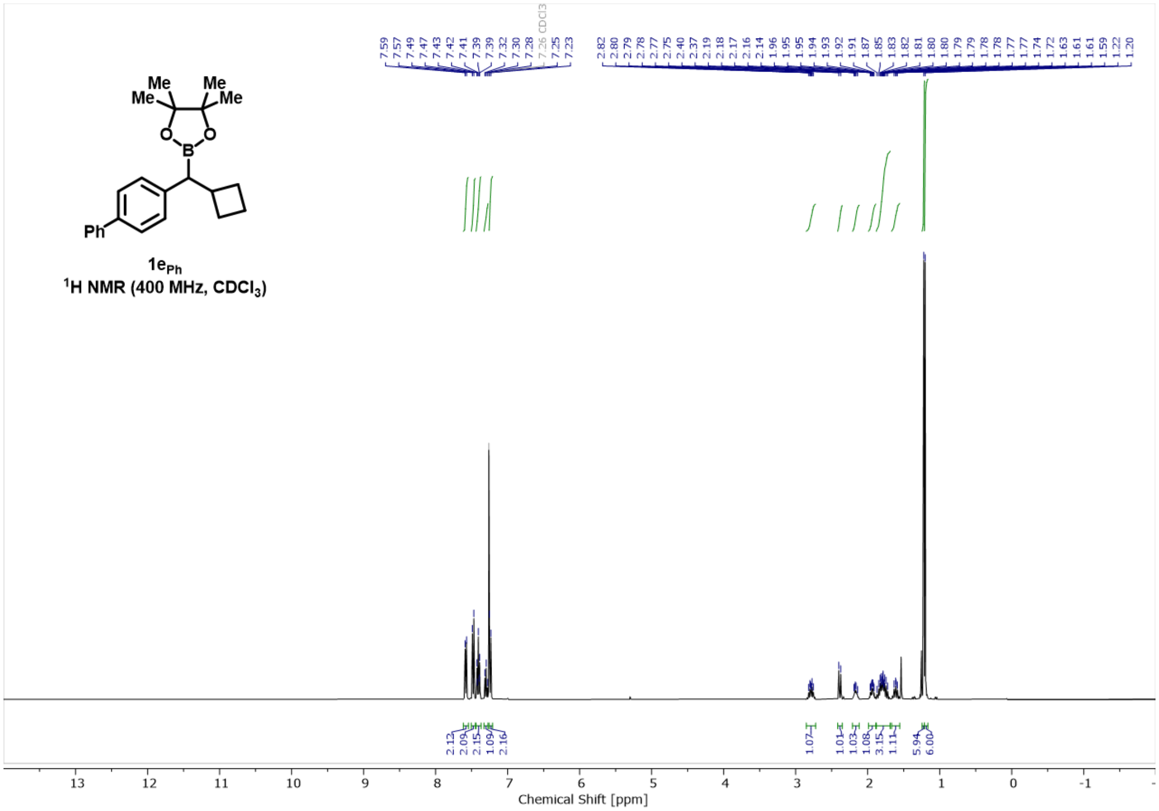


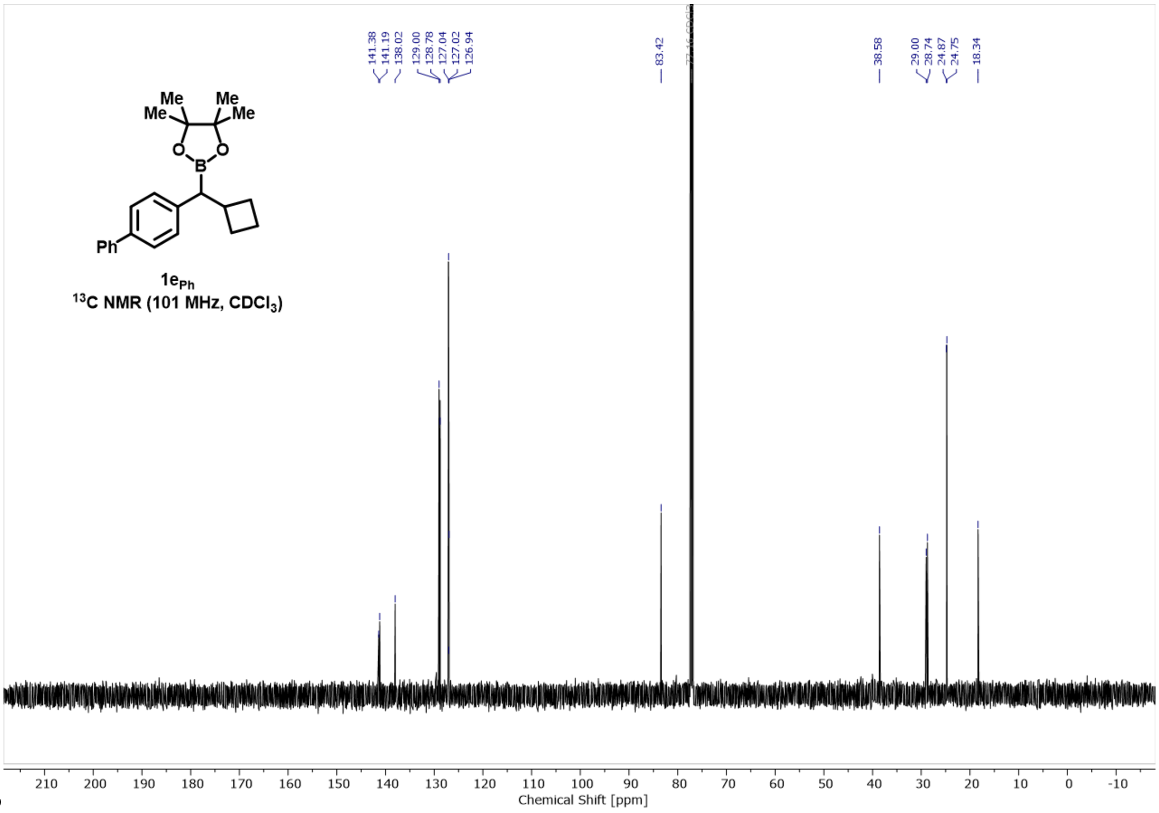


3

2-((4-(*tert*-butyl)phenyl)(cyclobutyl)methyl)-4,4,5,5-tetramethyl-1,3,2-dioxaborolane (**1e$_{tBu}$**)

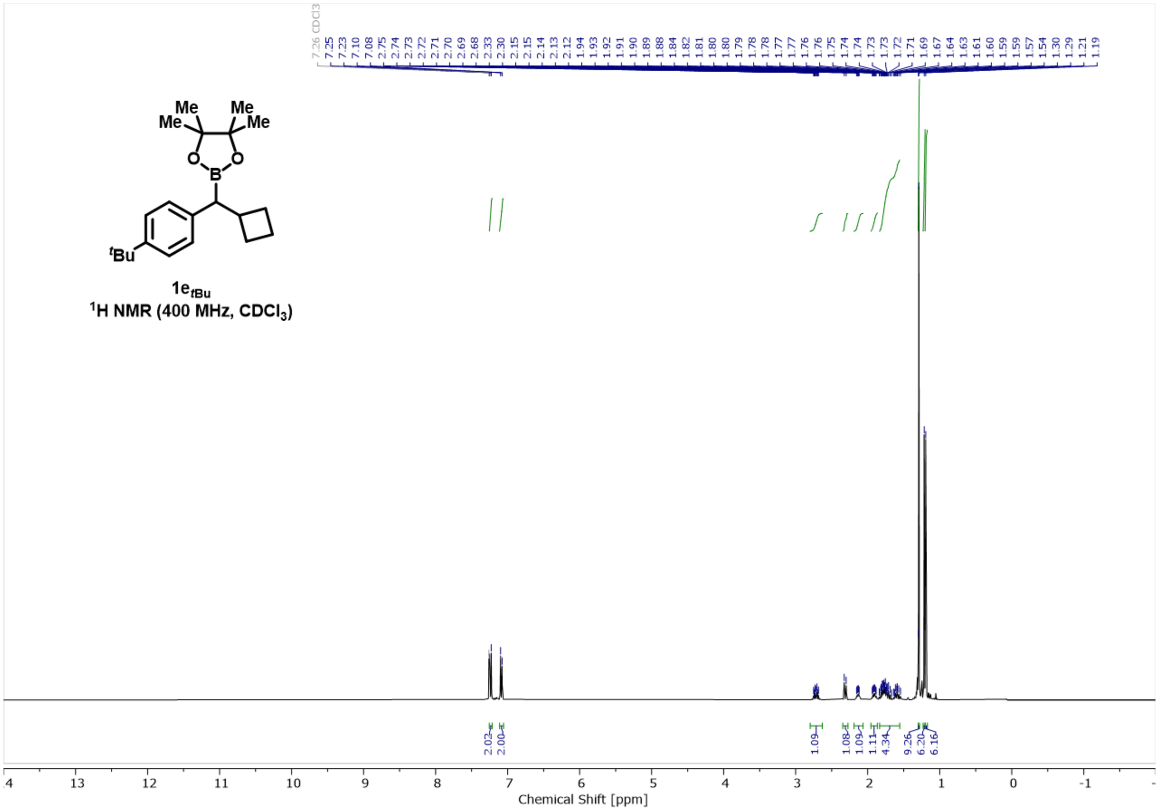


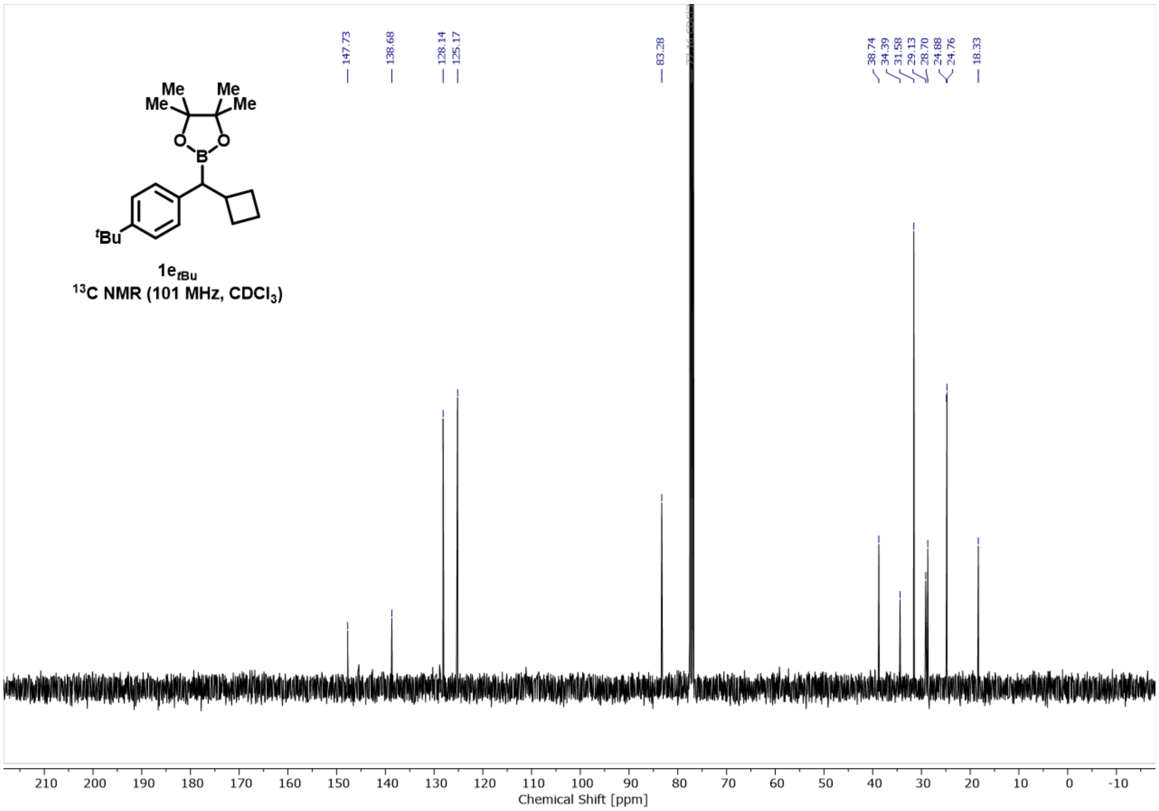

2-(cyclobutyl(4-methoxyphenyl)methyl)-4,4,5,5-tetramethyl-1,3,2-dioxaborolane (**1e$_{t\text{Bu}}$**)

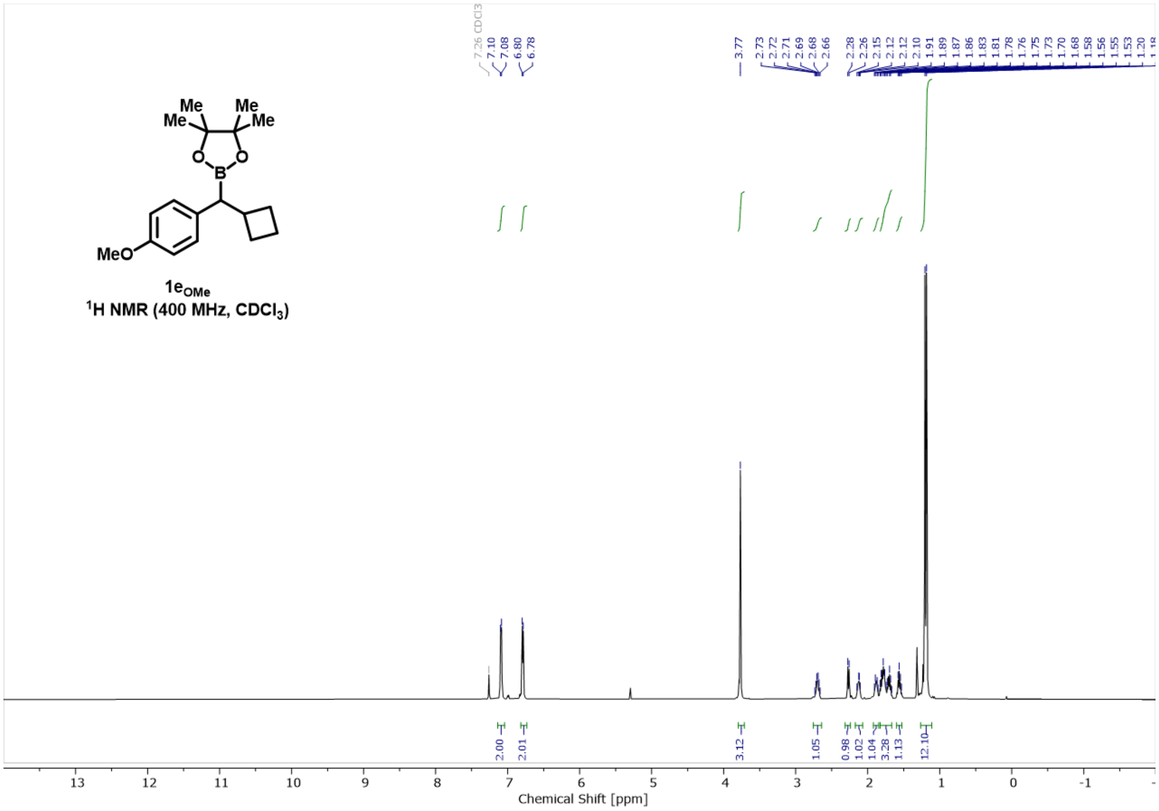


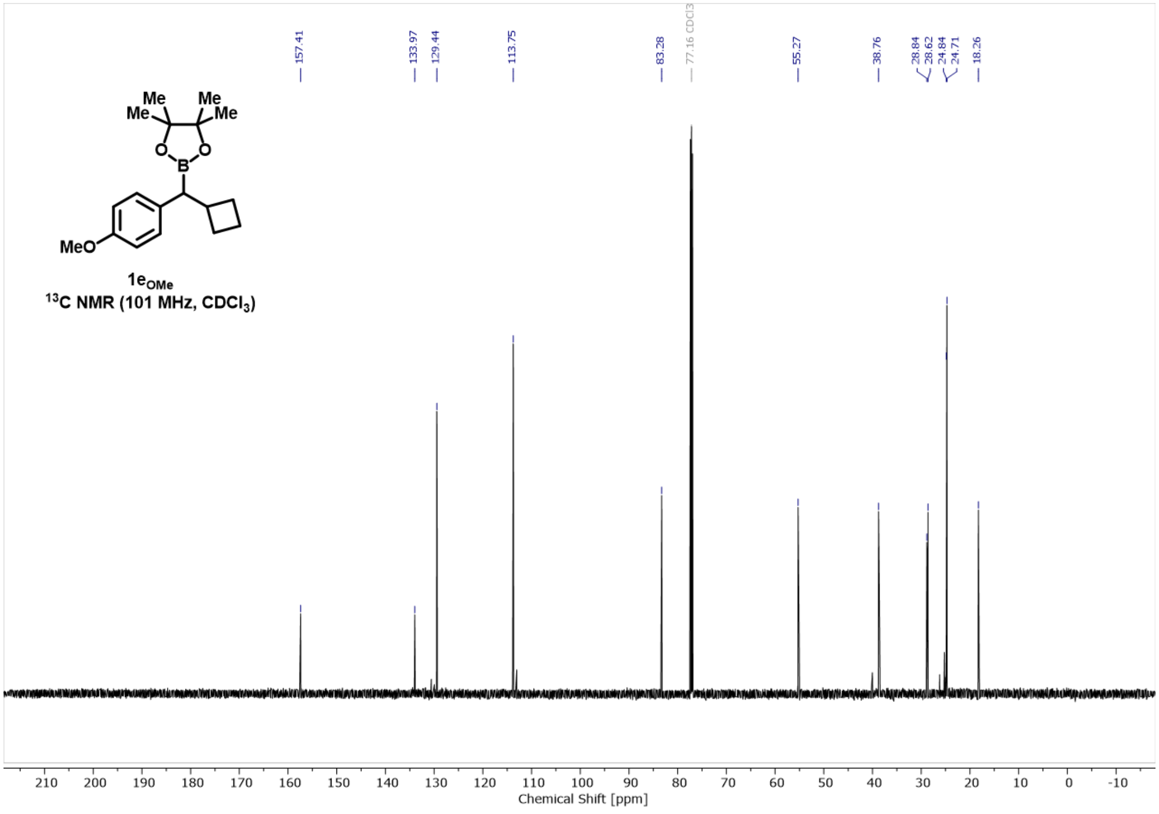

cyclobutyl(4-fluorophenyl)methyl adamantane-1-carboxylate (**3e$_F$**)

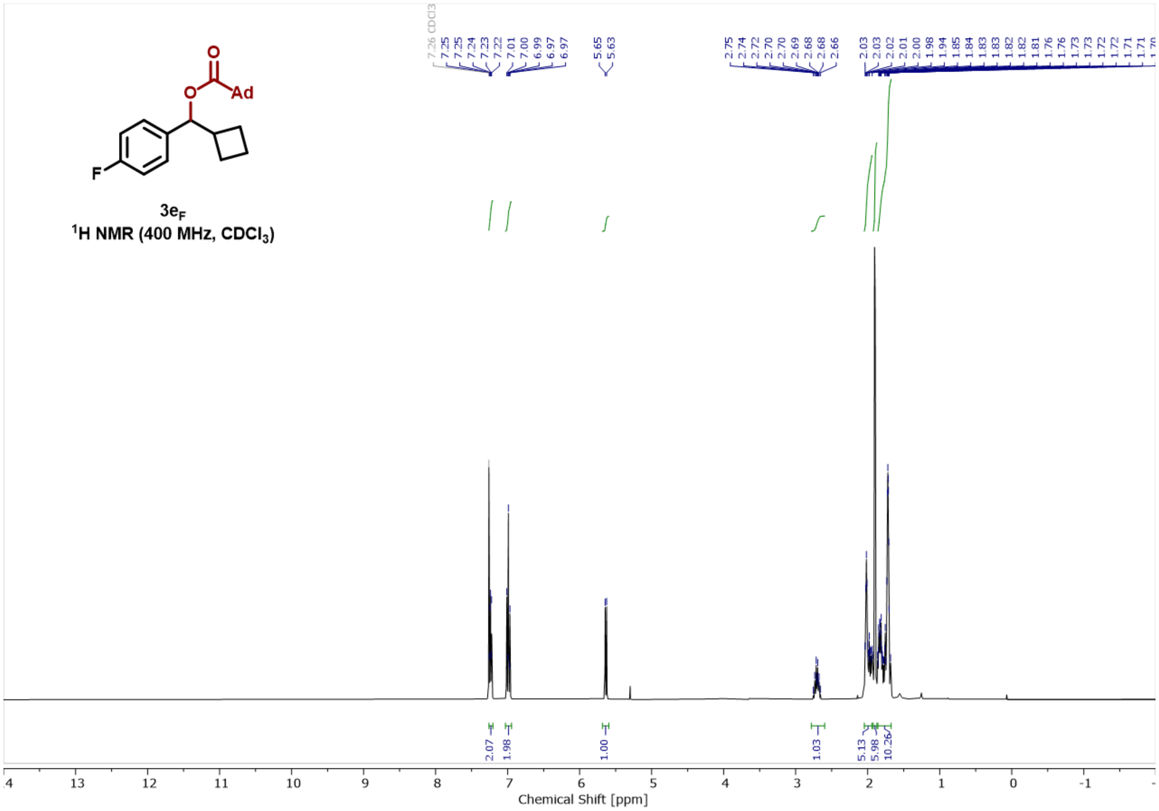

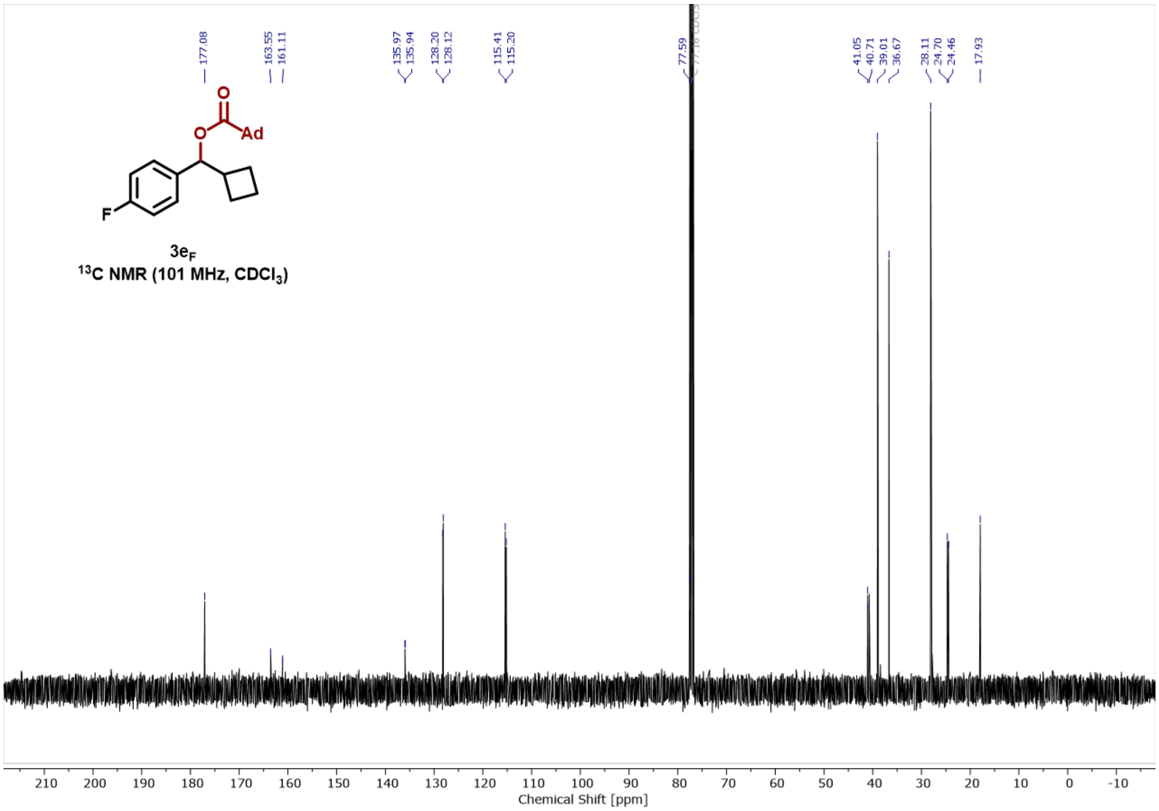

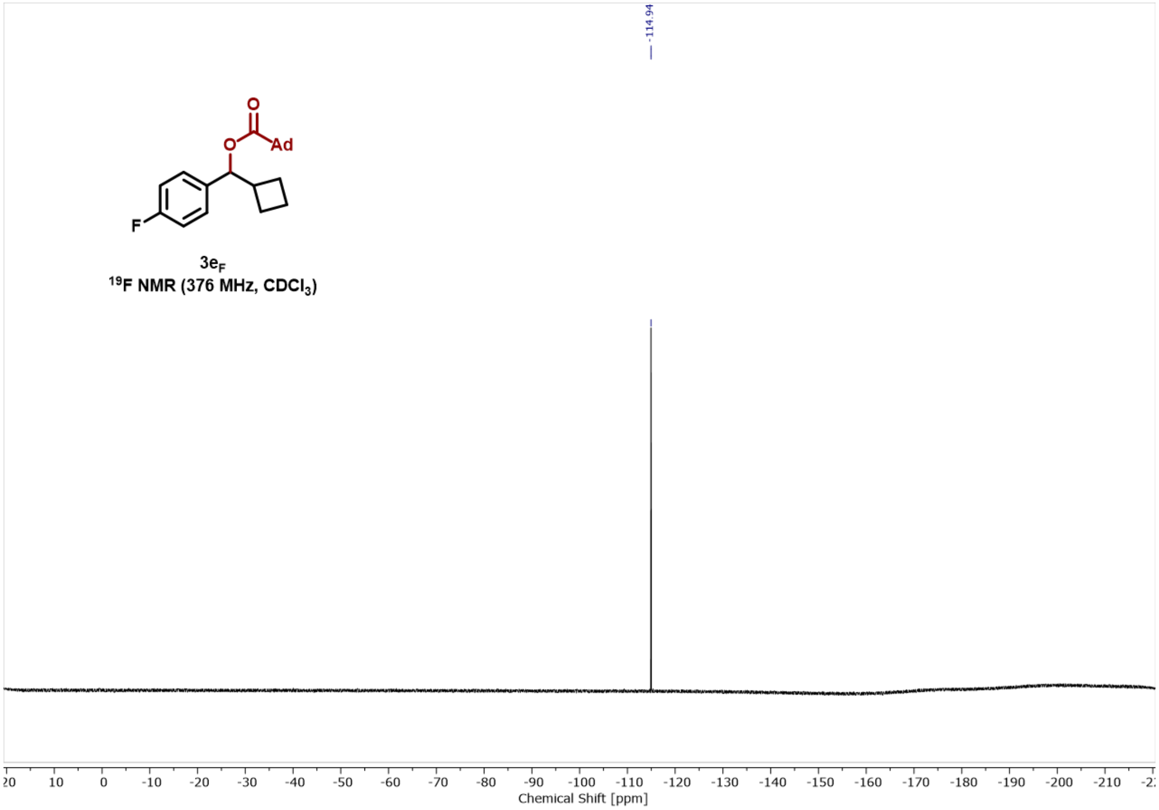

O
Ad
F
3e$_F$
$^{19}$F NMR (376 MHz, CDCl$_3$)
-114.94
20
10
0
-10
-20
-30
-40
-50
-60
-70
-80
-90
-100
-110
-120
-130
-140
-150
-160
-170
-180
-190
-200
-210
Chemical Shift [ppm]

[1,1'-biphenyl]-4-yl(cyclobutyl)methyl adamantane-1-carboxylate (**3e$_{Ph}$**)

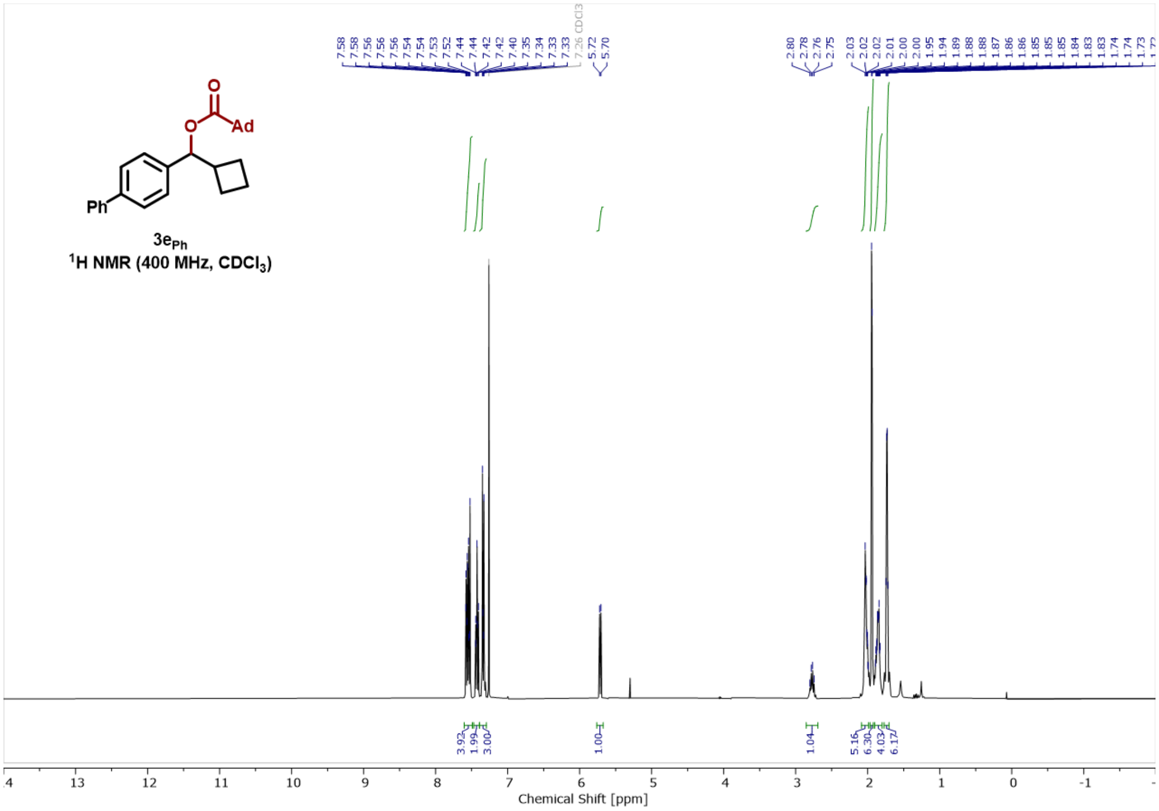


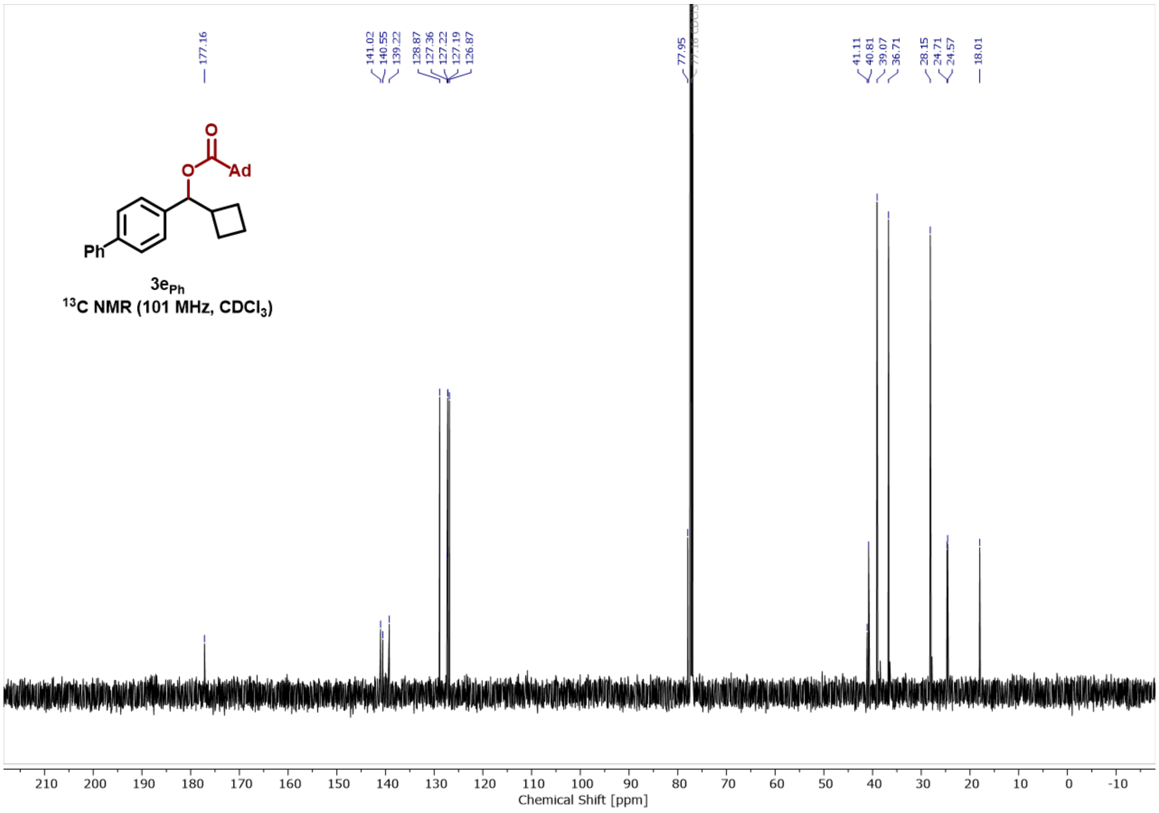

(4-(*tert*-butyl)phenyl)(cyclobutyl)methyl adamantane-1-carboxylate (**3e$_{tBu}$**)

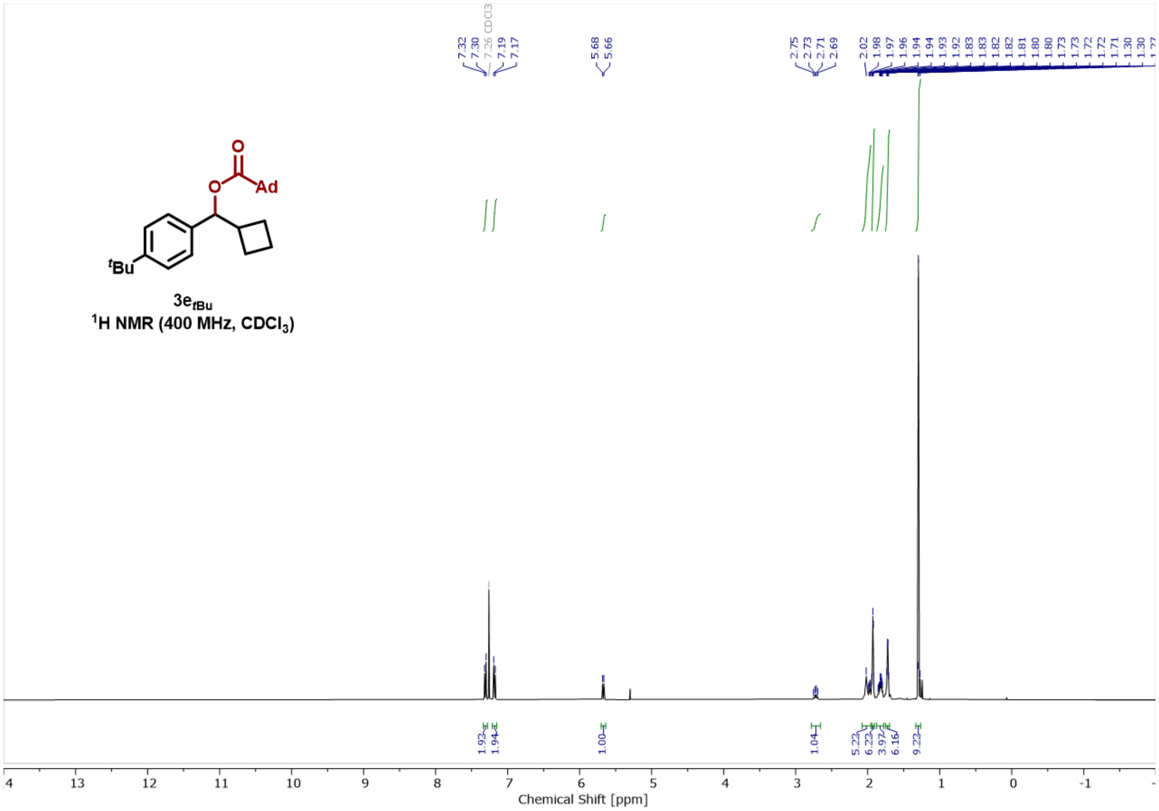


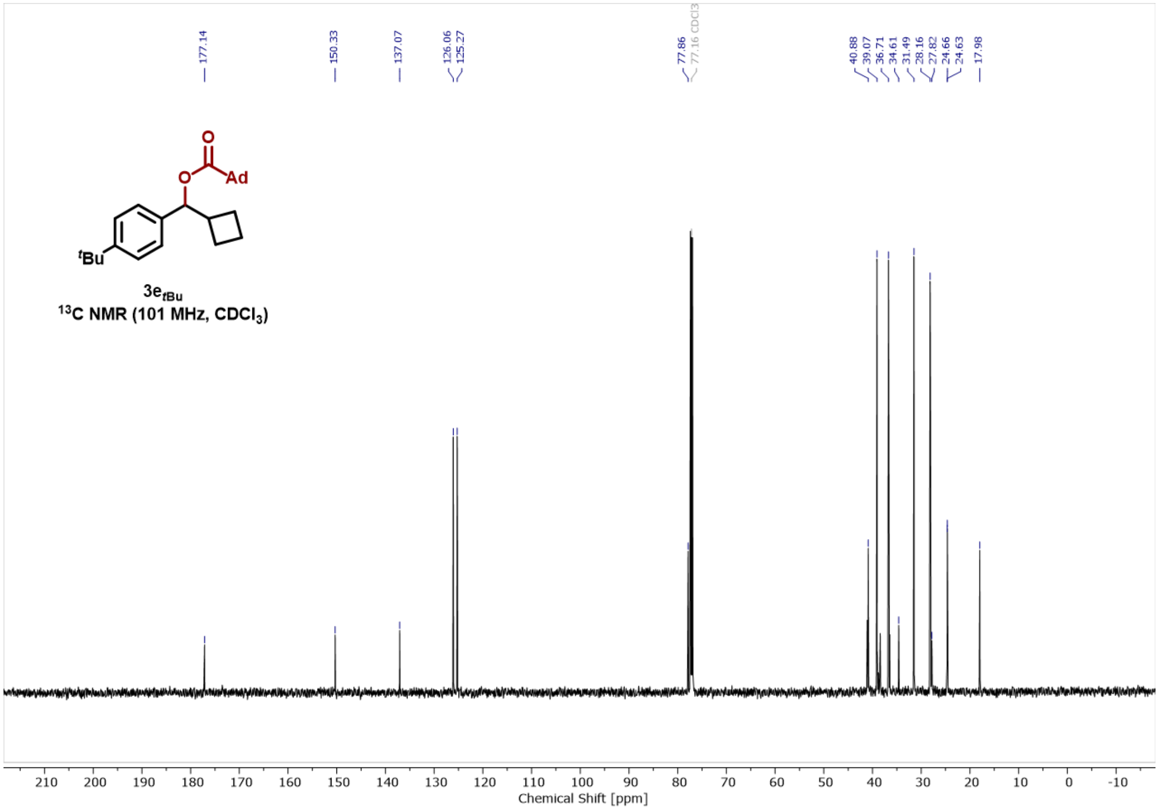

cyclobutyl(4-methoxyphenyl)methyl adamantane-1-carboxylate (**3a**$_{\mathbf{OMe}}$)

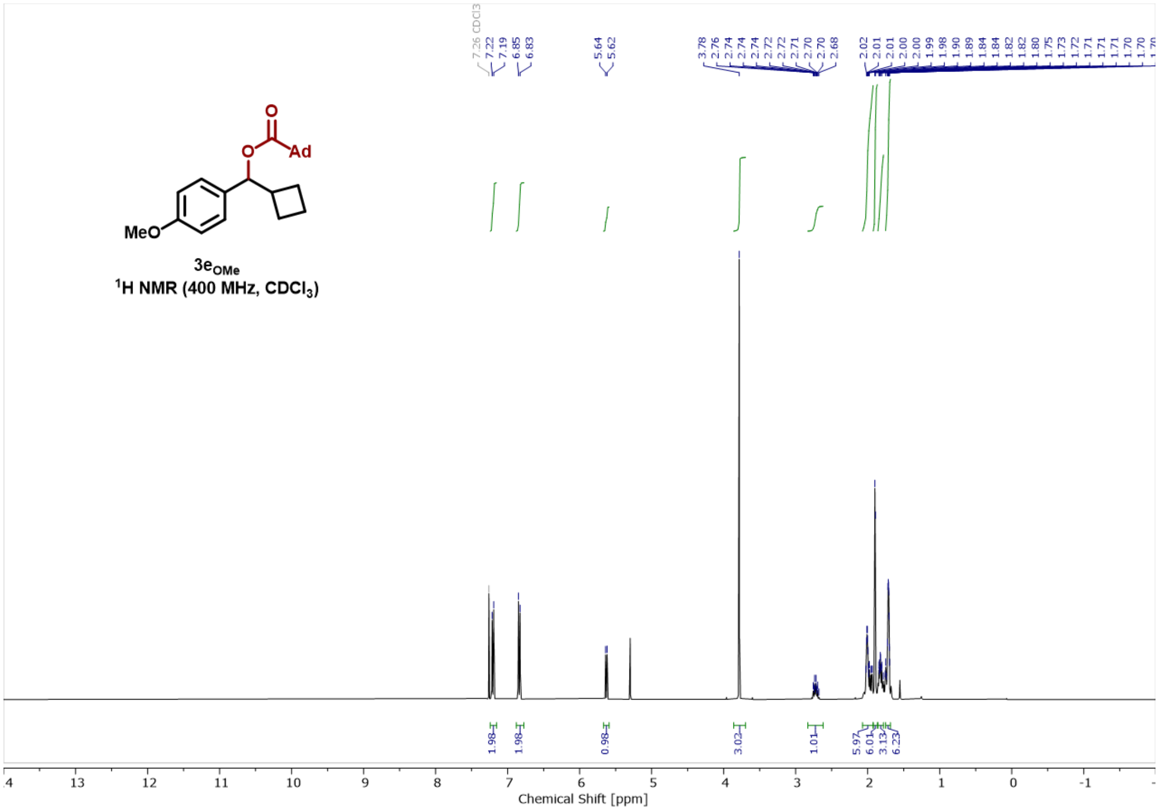


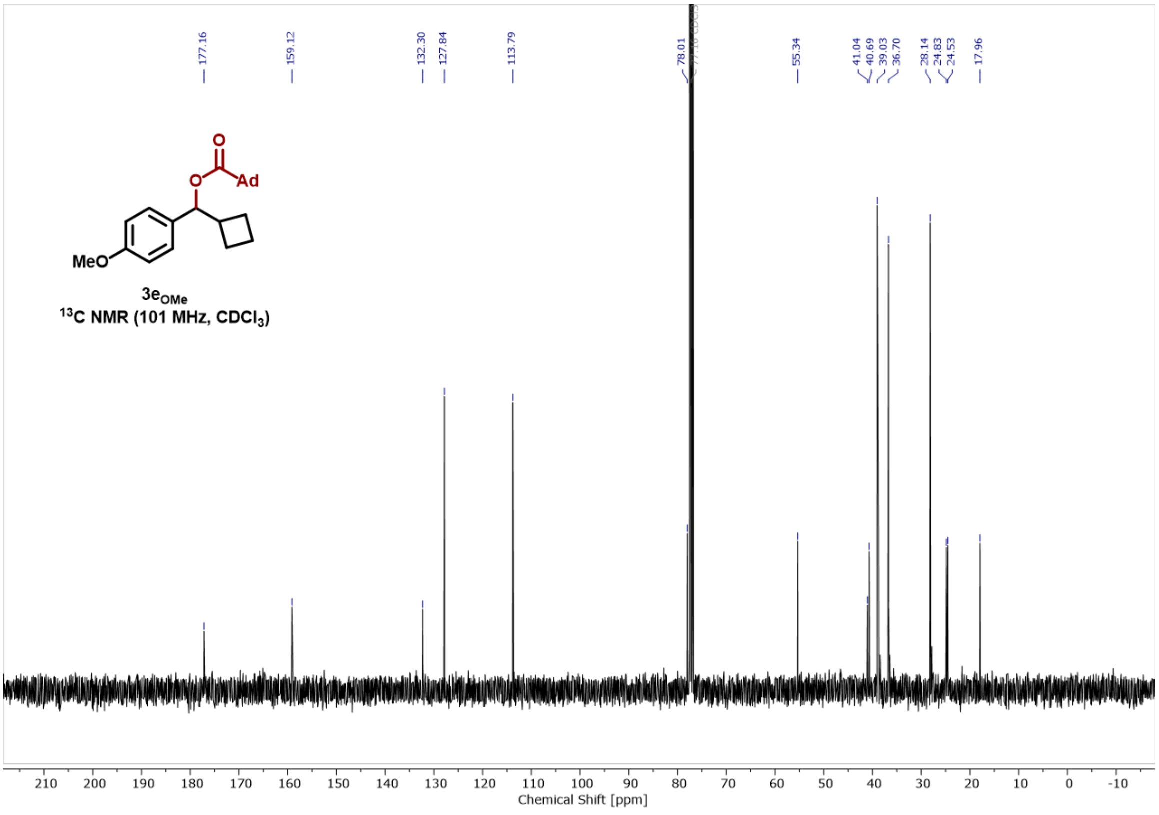

*Immunosuppressant Synthesis*

4,4,5,5-tetramethyl-2-(1-(naphthalen-2-yl)-3-phenylpropyl)-1,3,2-dioxaborolane (**6**)

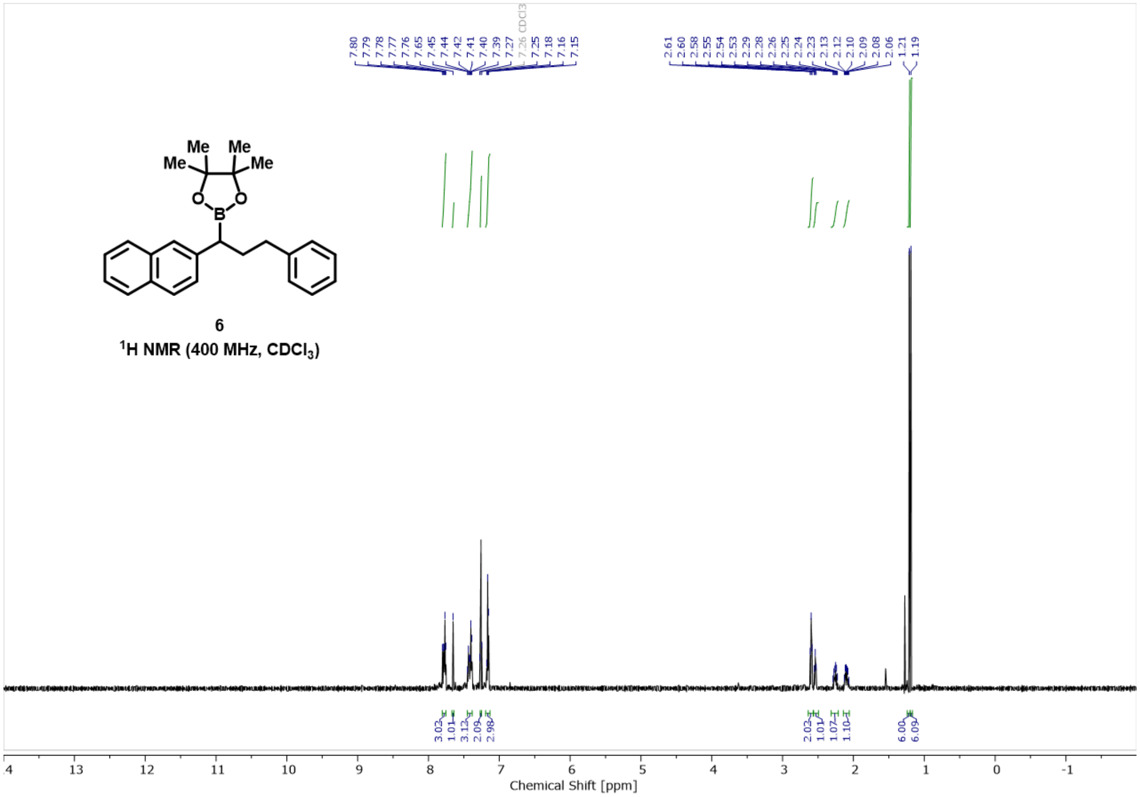

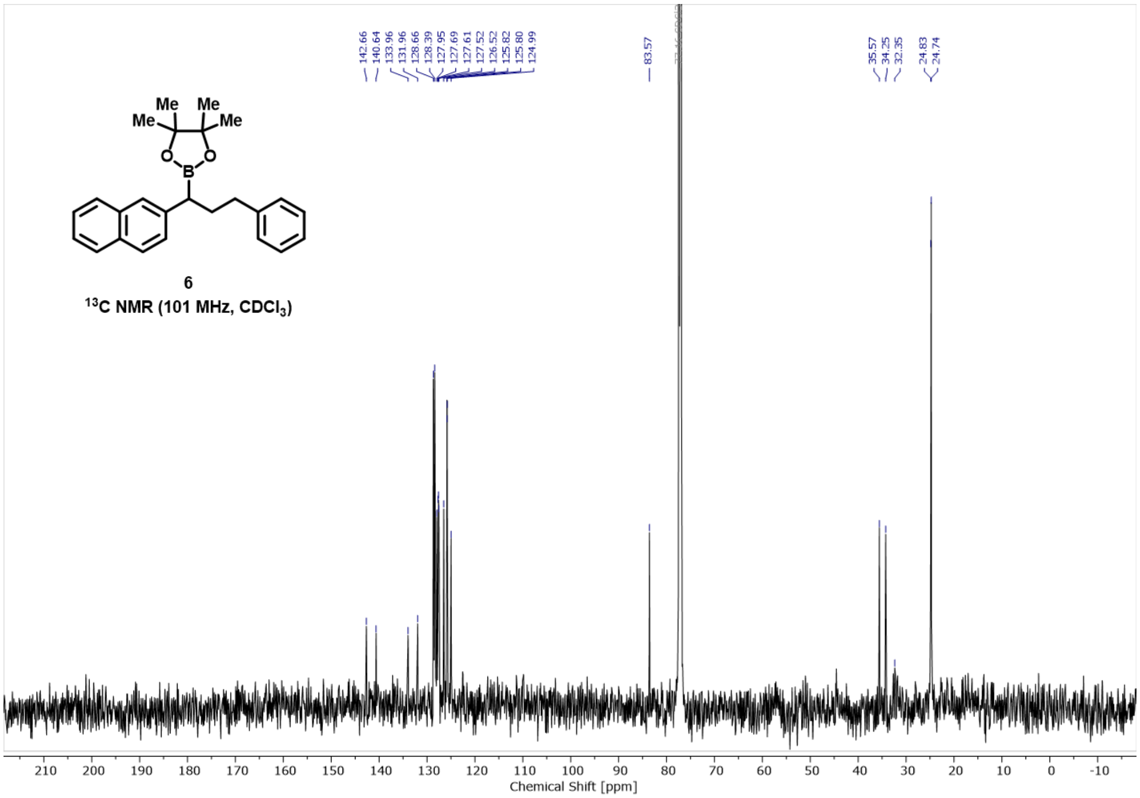

(*R*)-1-(naphthalen-2-yl)-3-phenylpropan-1-ol (**7**)

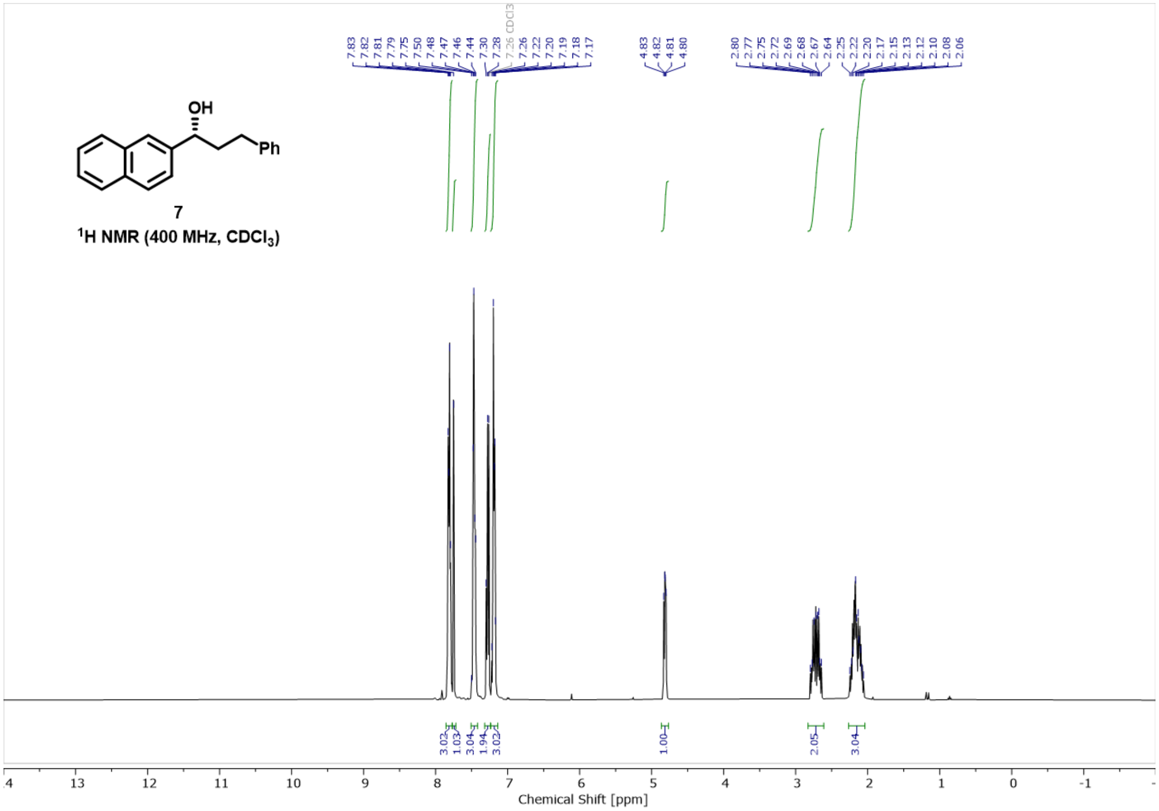


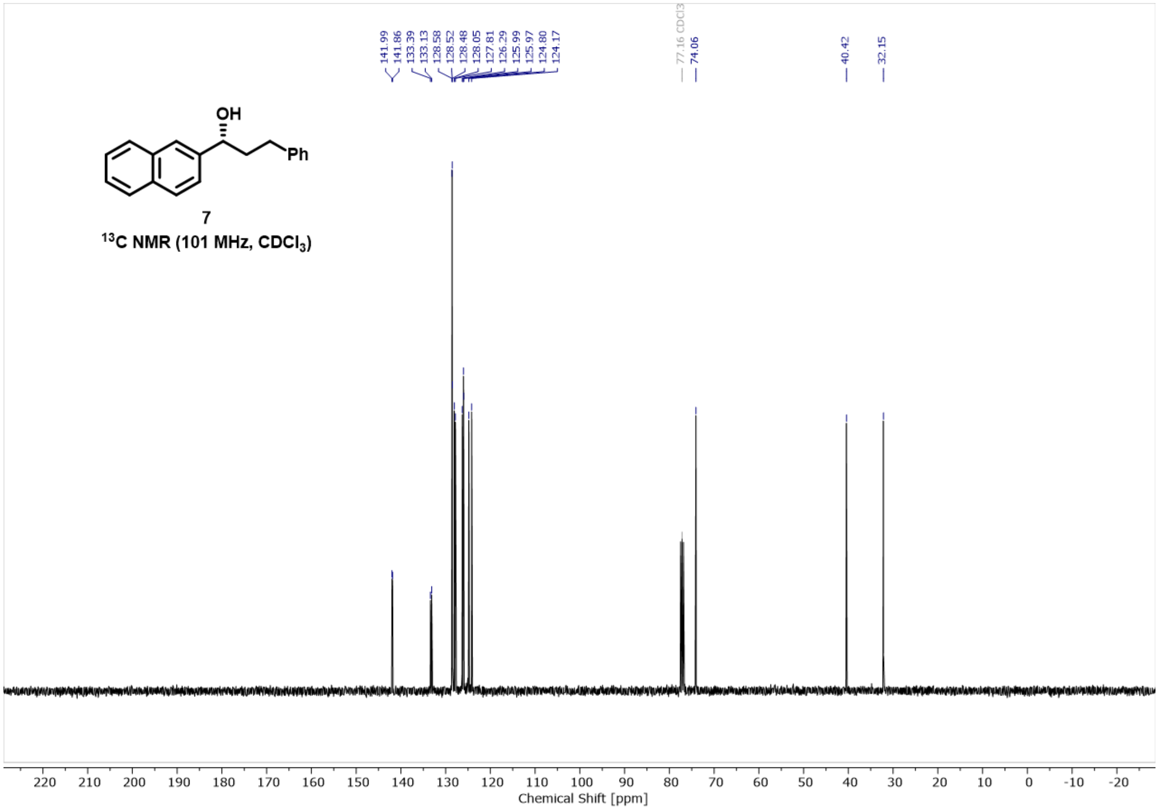

(*R*)-1-(2-naphthyl)-3-phenylpropyl (*S*)-1-(3,3-dimethyl-2-oxopentanoyl)piperidine-2-carboxylate (**8**)

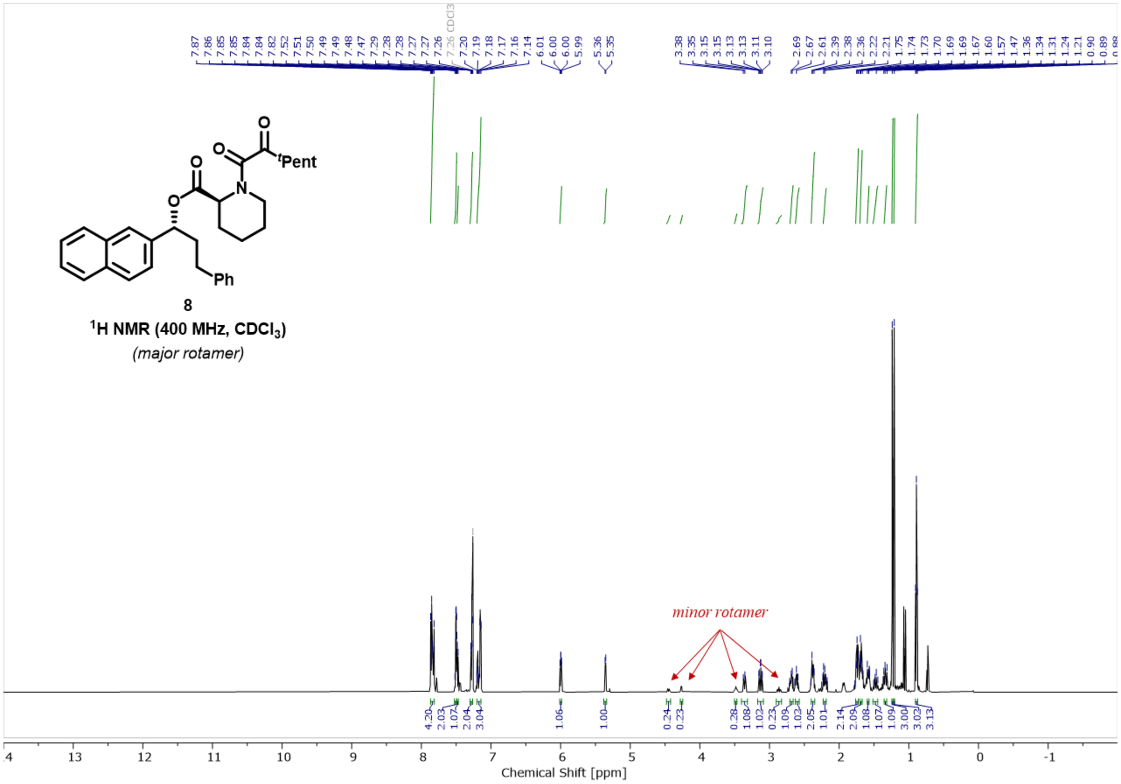


(only well-isolated peaks correspond to the minor rotamer are labeled)

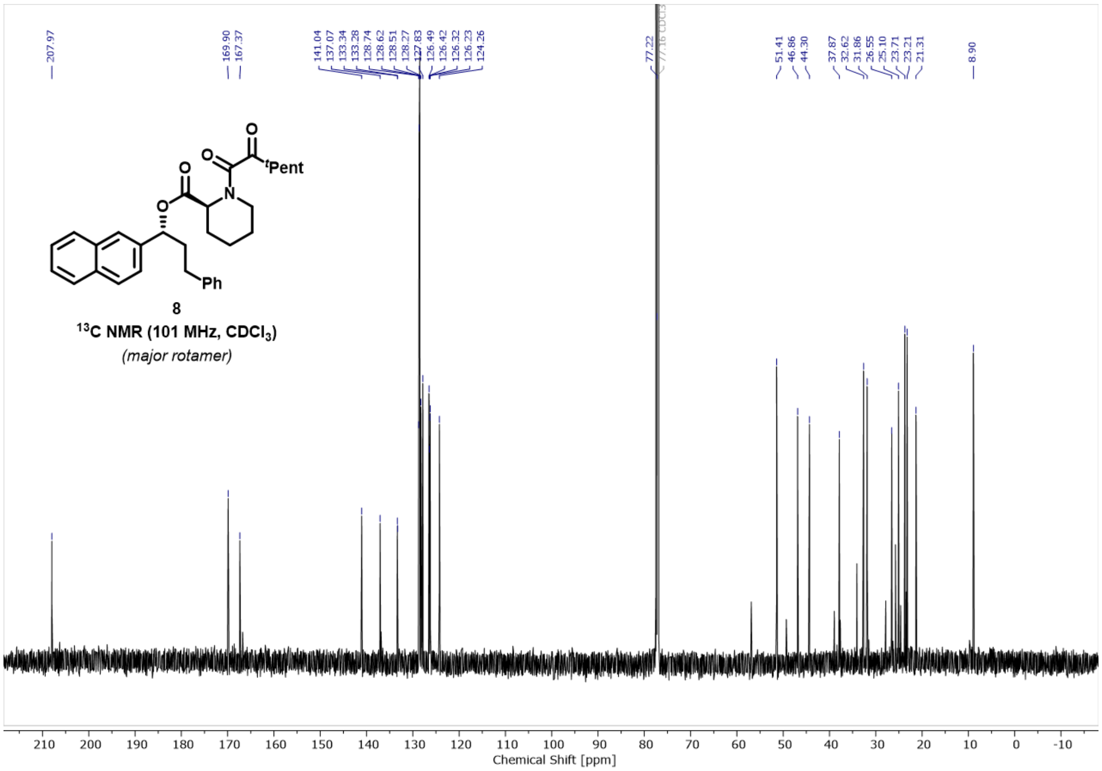

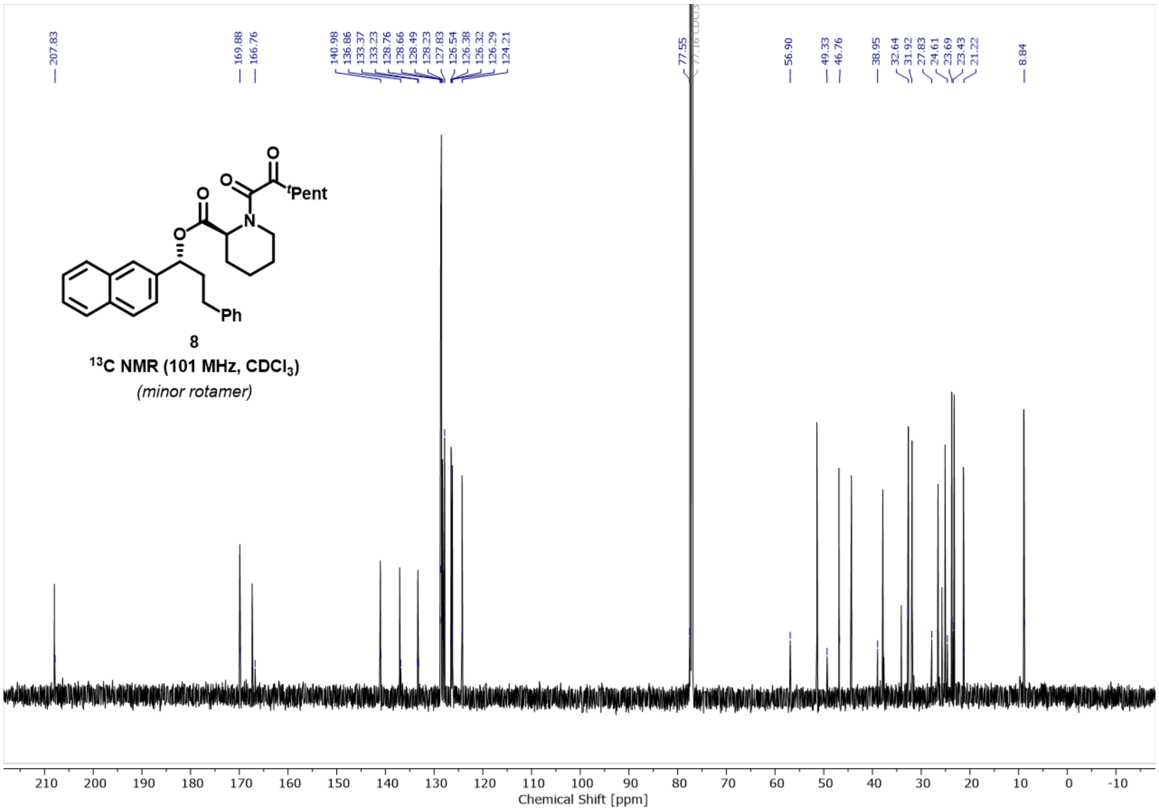

207.83
169.88
166.76
140.98
136.86
133.37
133.23
128.76
128.66
128.49
128.23
127.83
126.54
126.38
126.32
126.29
124.21
77.55
77.16 CDCl3
56.90
49.33
46.76
38.95
32.64
31.92
27.83
24.61
23.69
23.43
21.22
8.84
tPent
Ph
8
13C NMR (101 MHz, CDCl3)
(minor rotamer)
Chemical Shift [ppm]

*HPLC Traces*

(*S*)-1-(naphthalen-2-yl)ethyl benzoate (**3a**)

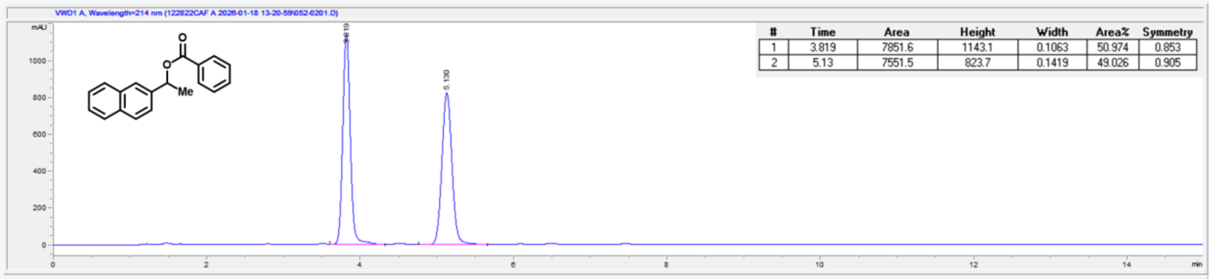


| # | Time | Area | Height | Width | Area% | Symmetry |
|---|---|---|---|---|---|---|
| 1 | 3.819 | 7851.6 | 1143.1 | 0.1063 | 50.974 | 0.853 |
| 2 | 5.13 | 7551.5 | 823.7 | 0.1419 | 49.026 | 0.905 |

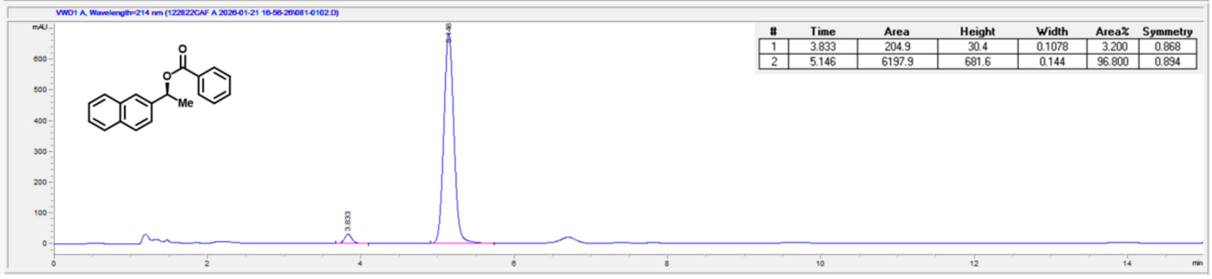


| # | Time | Area | Height | Width | Area% | Symmetry |
|---|---|---|---|---|---|---|
| 1 | 3.833 | 204.9 | 30.4 | 0.1078 | 3.200 | 0.868 |
| 2 | 5.146 | 6197.9 | 681.6 | 0.144 | 96.800 | 0.894 |

(*S*)-1-([1,1'-biphenyl]-2-yl)ethyladamantane-1-carboxylate (**3b**)

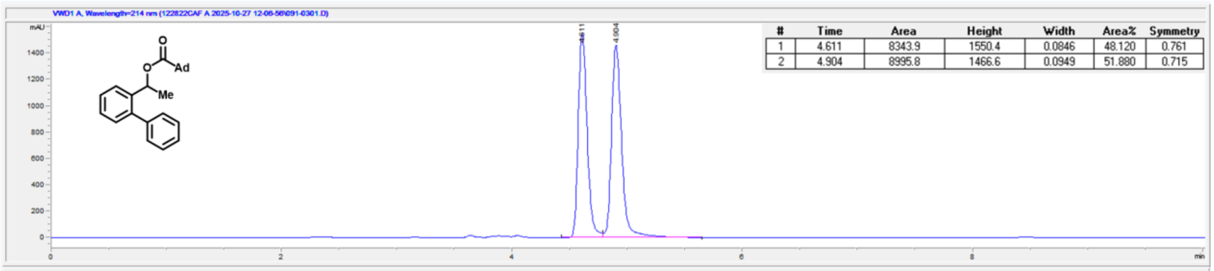


| # | Time | Area | Height | Width | Area% | Symmetry |
|---|---|---|---|---|---|---|
| 1 | 4.611 | 8343.9 | 1550.4 | 0.0846 | 48.120 | 0.761 |
| 2 | 4.904 | 8995.8 | 1466.6 | 0.0949 | 51.880 | 0.715 |

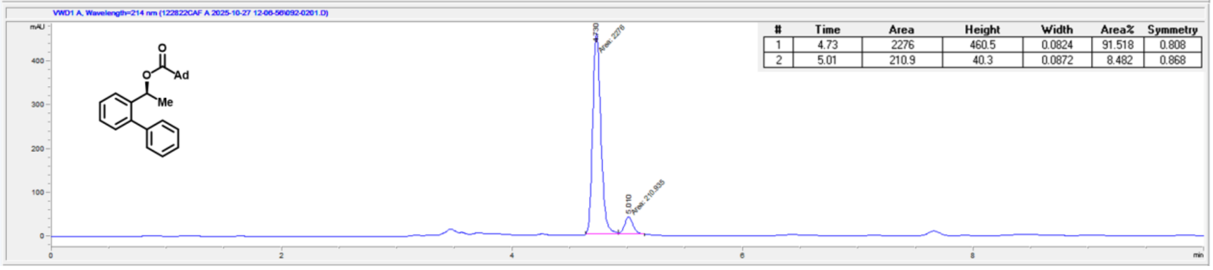


| # | Time | Area | Height | Width | Area% | Symmetry |
|---|---|---|---|---|---|---|
| 1 | 4.73 | 2276 | 460.5 | 0.0824 | 91.518 | 0.808 |
| 2 | 5.01 | 210.9 | 40.3 | 0.0872 | 8.482 | 0.868 |

(*S*)-*tert*-butyl 3-((benzoyloxy)(naphthalen-2-yl)methyl)azetidine-1-carboxylate (**3c**)

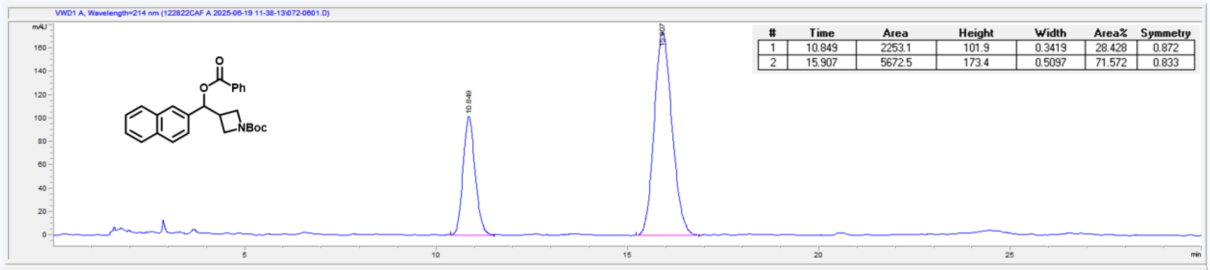

| # | Time | Area | Height | Width | Area% | Symmetry |
|---|---|---|---|---|---|---|
| 1 | 10.849 | 2253.1 | 101.9 | 0.3419 | 28.428 | 0.872 |
| 2 | 15.907 | 5672.5 | 173.4 | 0.5097 | 71.572 | 0.833 |

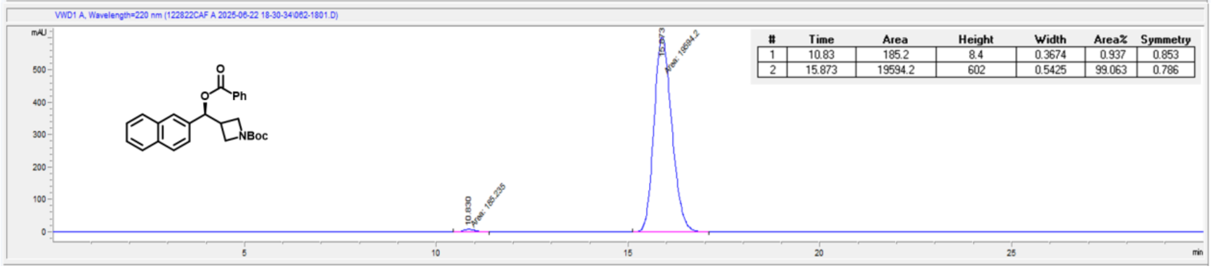

| # | Time | Area | Height | Width | Area% | Symmetry |
|---|---|---|---|---|---|---|
| 1 | 10.83 | 185.2 | 8.4 | 0.3674 | 0.937 | 0.853 |
| 2 | 15.873 | 19594.2 | 602 | 0.5425 | 99.063 | 0.786 |

---

(*S*)-1-(2-phenylthiazol-4-yl)butyl benzoate (**3d**)

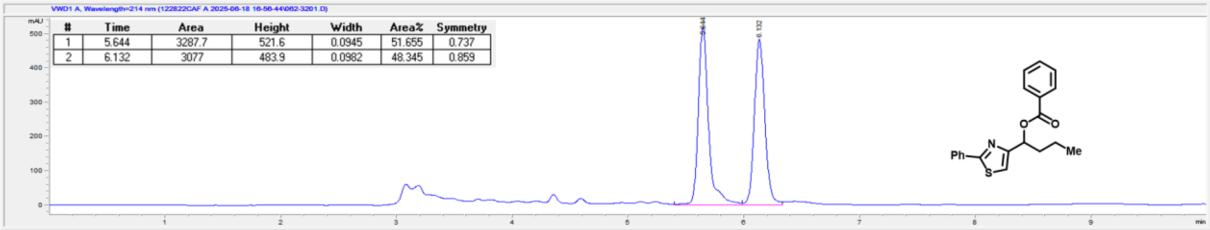

| # | Time | Area | Height | Width | Area% | Symmetry |
|---|---|---|---|---|---|---|
| 1 | 5.644 | 3287.7 | 521.6 | 0.0945 | 51.655 | 0.737 |
| 2 | 6.132 | 3077 | 483.9 | 0.0982 | 48.345 | 0.859 |

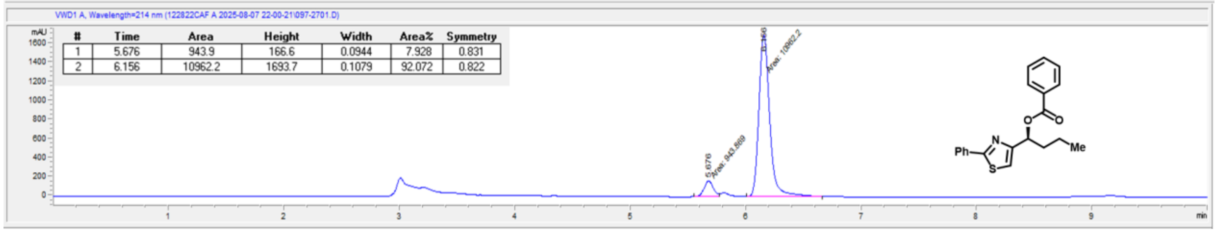

| # | Time | Area | Height | Width | Area% | Symmetry |
|---|---|---|---|---|---|---|
| 1 | 5.676 | 943.9 | 166.6 | 0.0944 | 7.928 | 0.831 |
| 2 | 6.156 | 10962.2 | 1693.7 | 0.1079 | 92.072 | 0.822 |

(*S*)-cyclobutyl(phenyl)methyladamantane-1-carboxylate (**3e**)

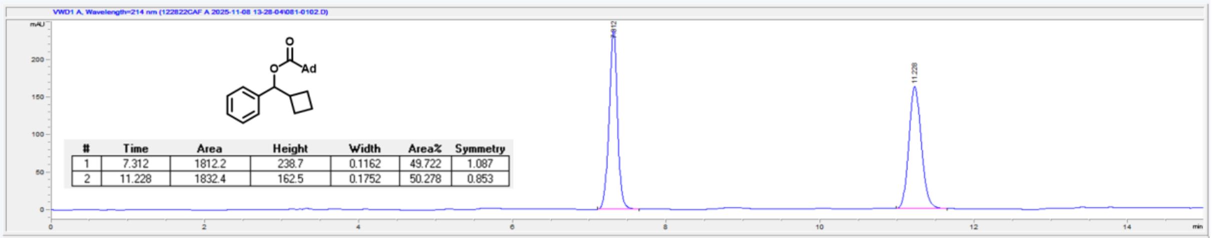


| # | Time | Area | Height | Width | Area% | Symmetry |
|---|---|---|---|---|---|---|
| 1 | 7.312 | 1812.2 | 238.7 | 0.1162 | 49.722 | 1.087 |
| 2 | 11.228 | 1832.4 | 162.5 | 0.1752 | 50.278 | 0.853 |

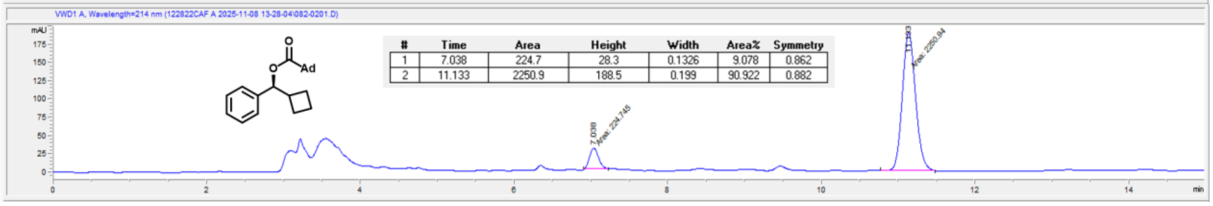


| # | Time | Area | Height | Width | Area% | Symmetry |
|---|---|---|---|---|---|---|
| 1 | 7.038 | 224.7 | 28.3 | 0.1326 | 9.078 | 0.862 |
| 2 | 11.133 | 2250.9 | 188.5 | 0.199 | 90.922 | 0.882 |

---

(*S*)-1-(benzo[b]thiophen-5-yl)-3-phenylpropylbenzoate (**3f**)

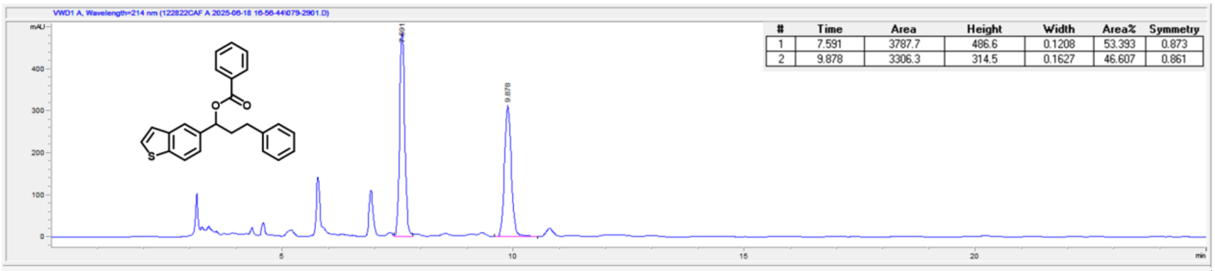


| # | Time | Area | Height | Width | Area% | Symmetry |
|---|---|---|---|---|---|---|
| 1 | 7.591 | 3787.7 | 486.6 | 0.1208 | 53.393 | 0.873 |
| 2 | 9.878 | 3306.3 | 314.5 | 0.1627 | 46.607 | 0.861 |

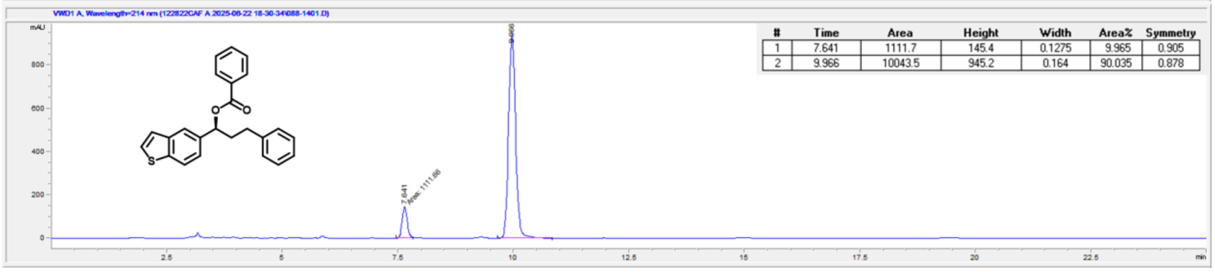


| # | Time | Area | Height | Width | Area% | Symmetry |
|---|---|---|---|---|---|---|
| 1 | 7.641 | 1111.7 | 145.4 | 0.1275 | 9.965 | 0.905 |
| 2 | 9.966 | 10043.5 | 945.2 | 0.164 | 90.035 | 0.878 |

(*S*)-1-(benzofuran-5-yl)butyladamantane-1-carboxylate (**3g**)

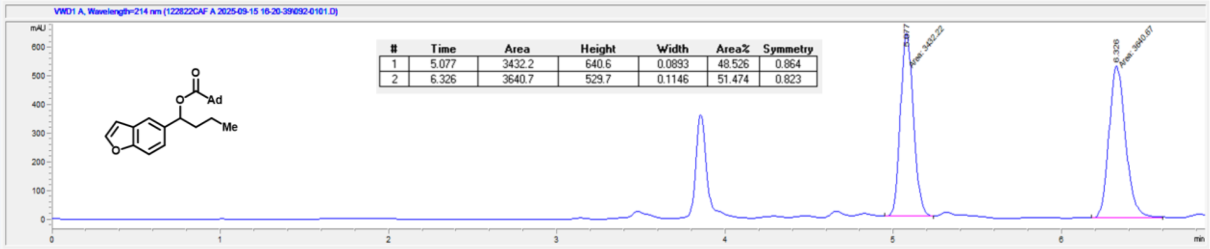

| # | Time | Area | Height | Width | Area% | Symmetry |
|---|---|---|---|---|---|---|
| 1 | 5.077 | 3432.2 | 640.6 | 0.0893 | 48.526 | 0.864 |
| 2 | 6.326 | 3640.7 | 529.7 | 0.1146 | 51.474 | 0.823 |

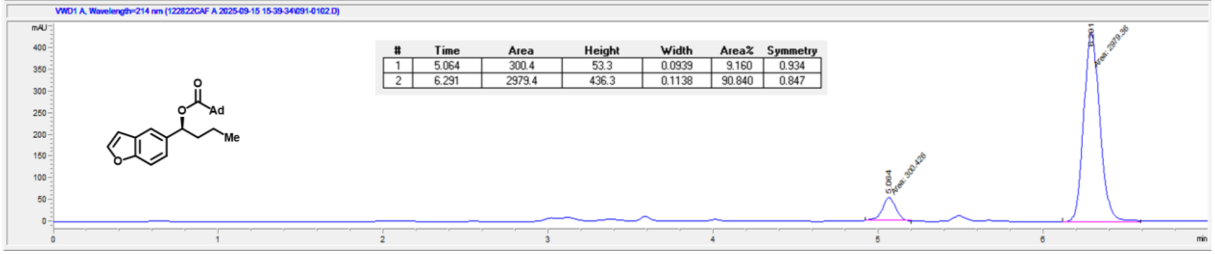

| # | Time | Area | Height | Width | Area% | Symmetry |
|---|---|---|---|---|---|---|
| 1 | 5.064 | 300.4 | 53.3 | 0.0939 | 9.160 | 0.934 |
| 2 | 6.291 | 2979.4 | 436.3 | 0.1138 | 90.840 | 0.847 |

---

(*S*)-1-(naphthalen-2-yl)-2-(4-(4,4,5,5-tetramethyl-1,3,2-dioxaborolan-2-yl)phenyl)ethyl benzoate (**3h**)

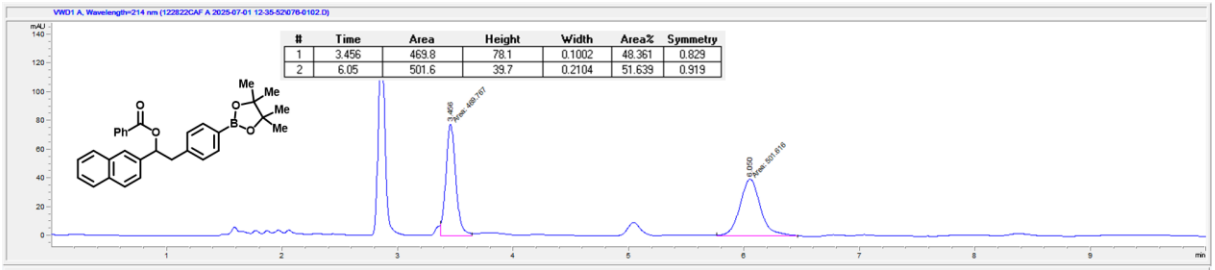

| # | Time | Area | Height | Width | Area% | Symmetry |
|---|---|---|---|---|---|---|
| 1 | 3.456 | 469.8 | 78.1 | 0.1002 | 48.361 | 0.829 |
| 2 | 6.05 | 501.6 | 39.7 | 0.2104 | 51.639 | 0.919 |

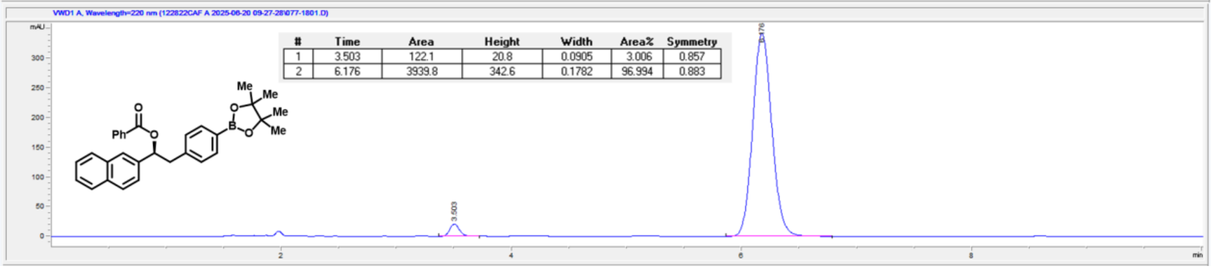

| # | Time | Area | Height | Width | Area% | Symmetry |
|---|---|---|---|---|---|---|
| 1 | 3.503 | 122.1 | 20.8 | 0.0905 | 3.006 | 0.857 |
| 2 | 6.176 | 3939.8 | 342.6 | 0.1782 | 96.994 | 0.883 |

(*S*)-1-(naphthalen-2-yl)-4-(4,4,5,5-tetramethyl-1,3,2-dioxaborolan-2-yl)butyl benzoate (**3i**)

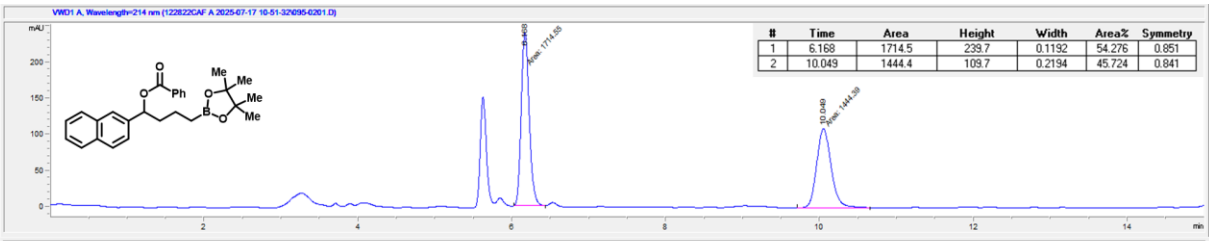


| # | Time | Area | Height | Width | Area% | Symmetry |
|---|---|---|---|---|---|---|
| 1 | 6.168 | 1714.5 | 239.7 | 0.1192 | 54.276 | 0.851 |
| 2 | 10.049 | 1444.4 | 109.7 | 0.2194 | 45.724 | 0.841 |

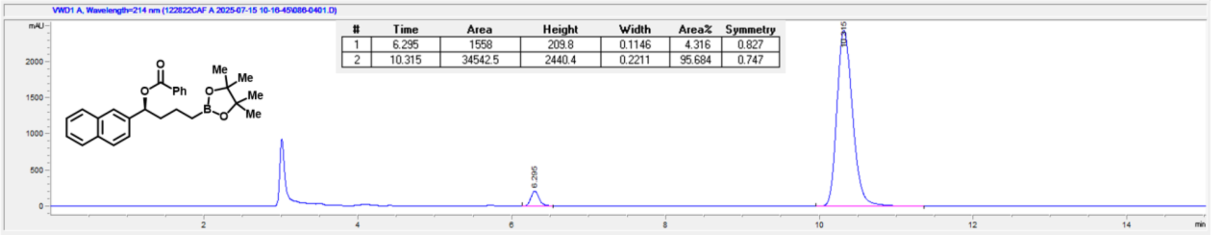


| # | Time | Area | Height | Width | Area% | Symmetry |
|---|---|---|---|---|---|---|
| 1 | 6.295 | 1558 | 209.8 | 0.1146 | 4.316 | 0.827 |
| 2 | 10.315 | 34542.5 | 2440.4 | 0.2211 | 95.684 | 0.747 |

(*S*)-3-chloro-1-(naphthalen-2-yl)propyl benzoate (**3j**)

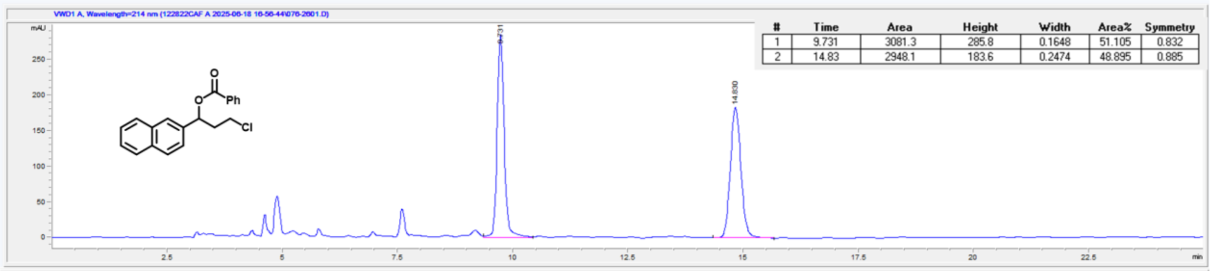


| # | Time | Area | Height | Width | Area% | Symmetry |
|---|---|---|---|---|---|---|
| 1 | 9.731 | 3081.3 | 285.8 | 0.1648 | 51.105 | 0.832 |
| 2 | 14.83 | 2948.1 | 183.6 | 0.2474 | 48.895 | 0.885 |

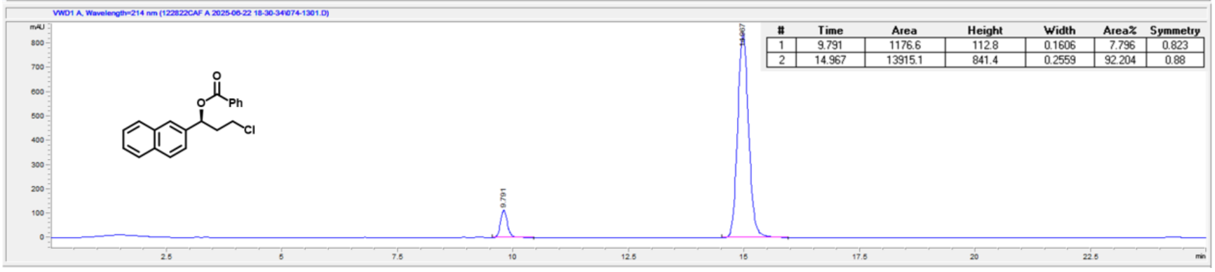


| # | Time | Area | Height | Width | Area% | Symmetry |
|---|---|---|---|---|---|---|
| 1 | 9.791 | 1176.6 | 112.8 | 0.1606 | 7.796 | 0.823 |
| 2 | 14.967 | 13915.1 | 841.4 | 0.2559 | 92.204 | 0.88 |

*tert*-butyl 3-((*S*)-( adamantane-1-carbonyl)oxy(benzofuran-5-yl)methyl)azetidine-1-carboxylate (**3k**)

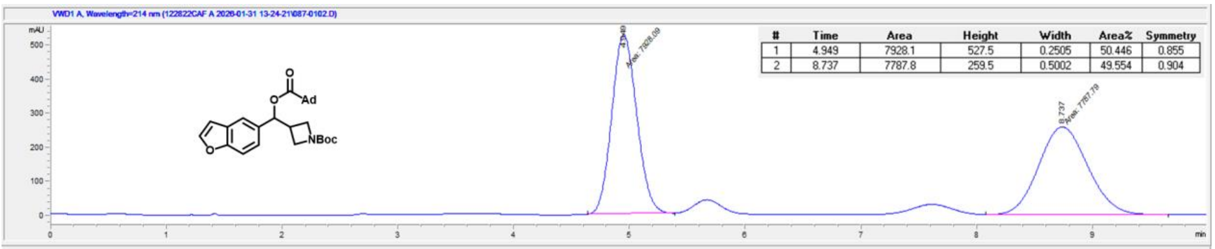


| # | Time | Area | Height | Width | Area% | Symmetry |
|---|---|---|---|---|---|---|
| 1 | 4.949 | 7928.1 | 527.5 | 0.2505 | 50.446 | 0.855 |
| 2 | 8.737 | 7787.8 | 259.5 | 0.5002 | 49.554 | 0.904 |

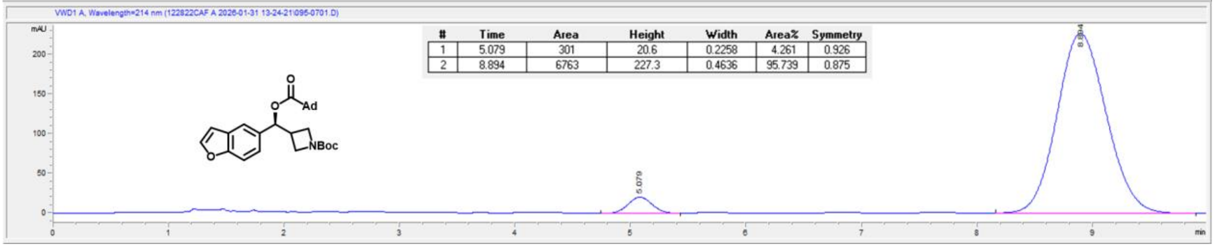


| # | Time | Area | Height | Width | Area% | Symmetry |
|---|---|---|---|---|---|---|
| 1 | 5.079 | 301 | 20.6 | 0.2258 | 4.261 | 0.926 |
| 2 | 8.894 | 6763 | 227.3 | 0.4636 | 95.739 | 0.875 |

(*S*)-3-(2,3-dihydrobenzofuran-6-yl)-1-(naphthalen-2-yl)propyl benzoate (**3l**)

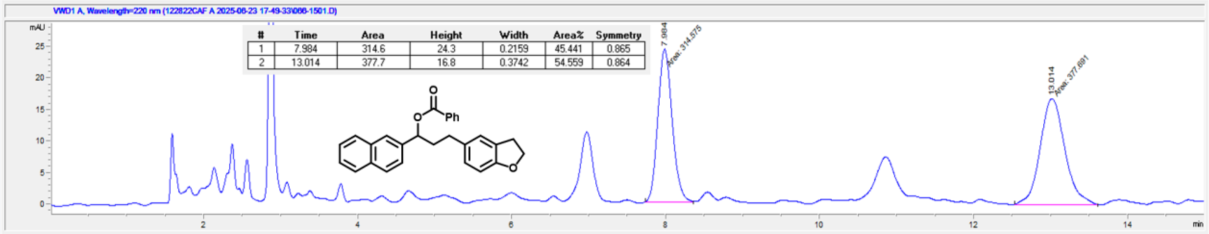


| # | Time | Area | Height | Width | Area% | Symmetry |
|---|---|---|---|---|---|---|
| 1 | 7.984 | 314.6 | 24.3 | 0.2159 | 45.441 | 0.865 |
| 2 | 13.014 | 377.7 | 16.8 | 0.3742 | 54.559 | 0.864 |

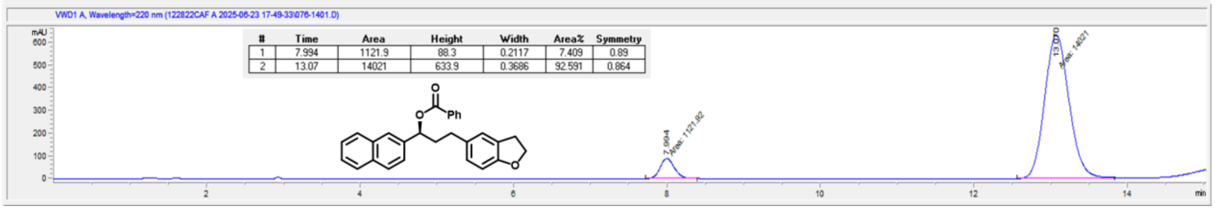


| # | Time | Area | Height | Width | Area% | Symmetry |
|---|---|---|---|---|---|---|
| 1 | 7.994 | 1121.9 | 88.3 | 0.2117 | 7.409 | 0.89 |
| 2 | 13.07 | 14021 | 633.9 | 0.3686 | 92.591 | 0.864 |

(*S*)-1-(benzo[b]thiophen-5-yl)butyl benzoate (**3m**)

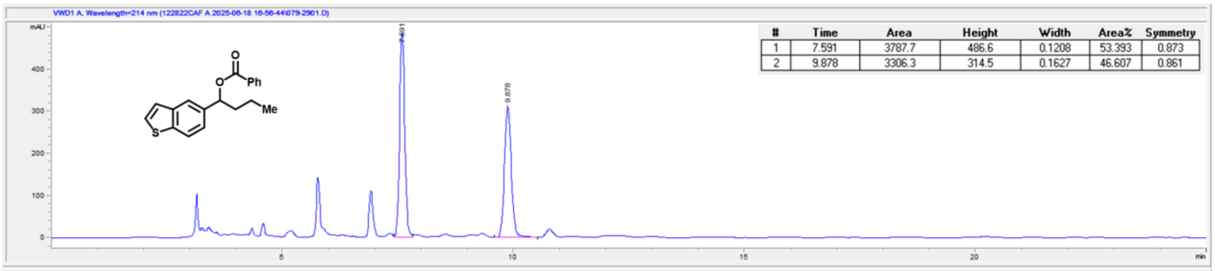

| # | Time | Area | Height | Width | Area% | Symmetry |
| --- | --- | --- | --- | --- | --- | --- |
| 1 | 7.591 | 3787.7 | 486.6 | 0.1208 | 53.393 | 0.873 |
| 2 | 9.878 | 3306.3 | 314.5 | 0.1627 | 46.607 | 0.861 |

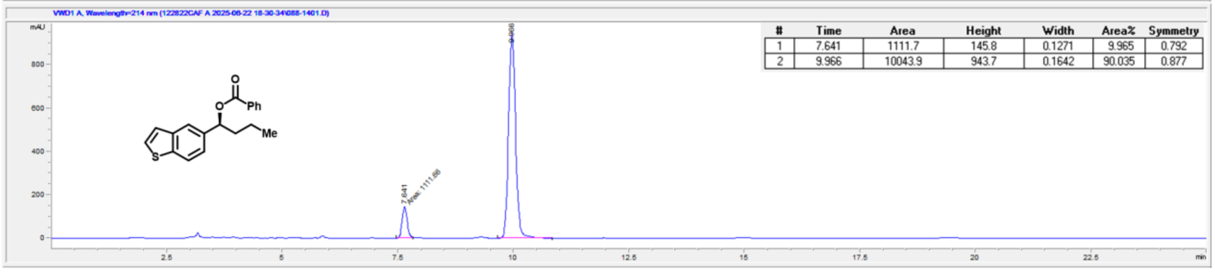

| # | Time | Area | Height | Width | Area% | Symmetry |
| --- | --- | --- | --- | --- | --- | --- |
| 1 | 7.641 | 1111.7 | 145.8 | 0.1271 | 9.965 | 0.792 |
| 2 | 9.966 | 10043.9 | 943.7 | 0.1642 | 90.035 | 0.877 |

(*S*)-1-(6-bromonaphthalen-2-yl)ethyl benzoate (**3n**)

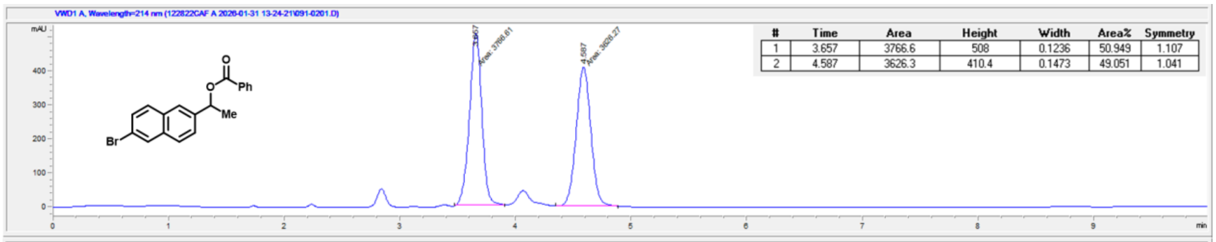

| # | Time | Area | Height | Width | Area% | Symmetry |
| --- | --- | --- | --- | --- | --- | --- |
| 1 | 3.657 | 3766.6 | 508 | 0.1236 | 50.949 | 1.107 |
| 2 | 4.587 | 3626.3 | 410.4 | 0.1473 | 49.051 | 1.041 |

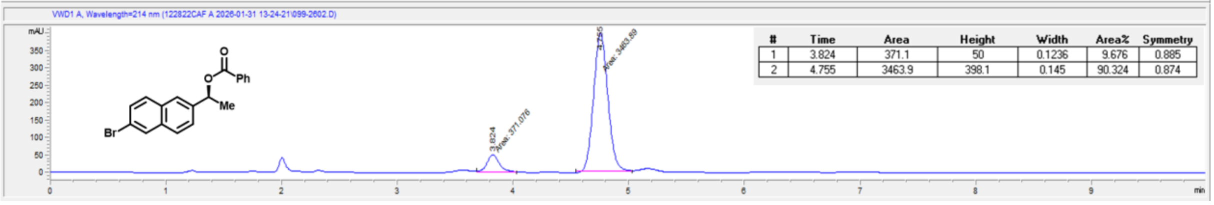

| # | Time | Area | Height | Width | Area% | Symmetry |
| --- | --- | --- | --- | --- | --- | --- |
| 1 | 3.824 | 371.1 | 50 | 0.1236 | 9.676 | 0.885 |
| 2 | 4.755 | 3463.9 | 398.1 | 0.145 | 90.324 | 0.874 |

(*S*)-cyclobutyl(1-tosyl-1*H*-indol-5-yl)methyladamantane-1-carboxylate (**3o**)

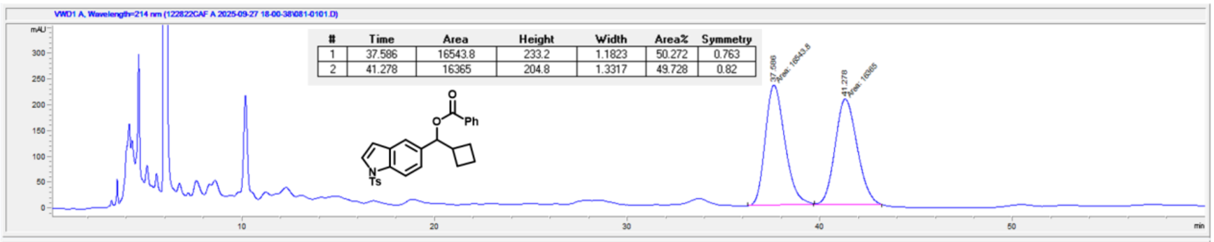

| # | Time | Area | Height | Width | Area% | Symmetry |
|---|---|---|---|---|---|---|
| 1 | 37.586 | 16543.8 | 233.2 | 1.1823 | 50.272 | 0.763 |
| 2 | 41.278 | 16365 | 204.8 | 1.3317 | 49.728 | 0.82 |

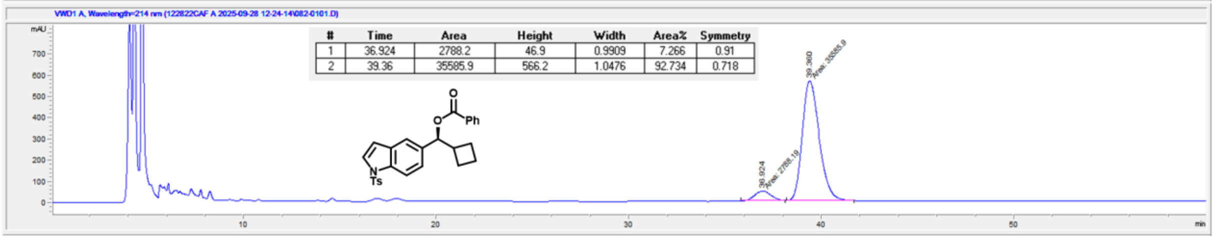

| # | Time | Area | Height | Width | Area% | Symmetry |
|---|---|---|---|---|---|---|
| 1 | 36.924 | 2788.2 | 46.9 | 0.9909 | 7.266 | 0.91 |
| 2 | 39.36 | 35585.9 | 566.2 | 1.0476 | 92.734 | 0.718 |

---

(*S*)-1-(naphthalen-2-yl)-3-(1*H*-pyrrol-1-yl)propyl benzoate (**3p**)

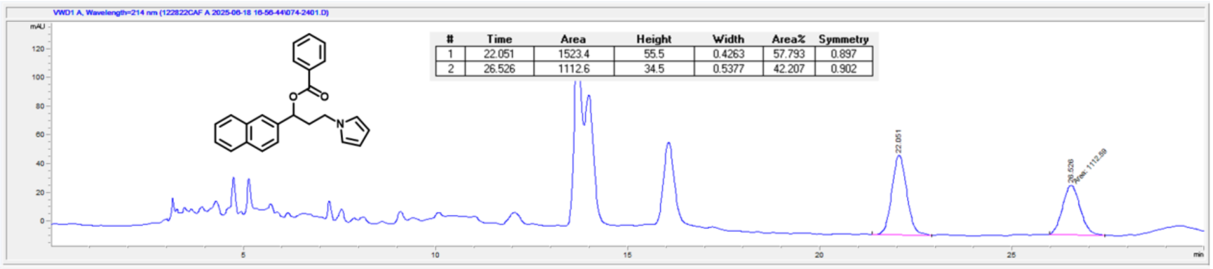

| # | Time | Area | Height | Width | Area% | Symmetry |
|---|---|---|---|---|---|---|
| 1 | 22.051 | 1523.4 | 55.5 | 0.4263 | 57.793 | 0.897 |
| 2 | 26.526 | 1112.6 | 34.5 | 0.5377 | 42.207 | 0.902 |

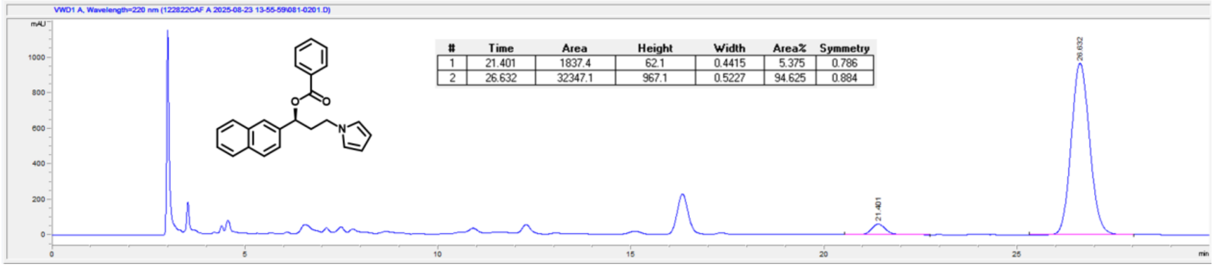

| # | Time | Area | Height | Width | Area% | Symmetry |
|---|---|---|---|---|---|---|
| 1 | 21.401 | 1837.4 | 62.1 | 0.4415 | 5.375 | 0.786 |
| 2 | 26.632 | 32347.1 | 967.1 | 0.5227 | 94.625 | 0.884 |

(*S*,*E*)-4-phenylbut-3-en-2-yl benzoate (**3q**)

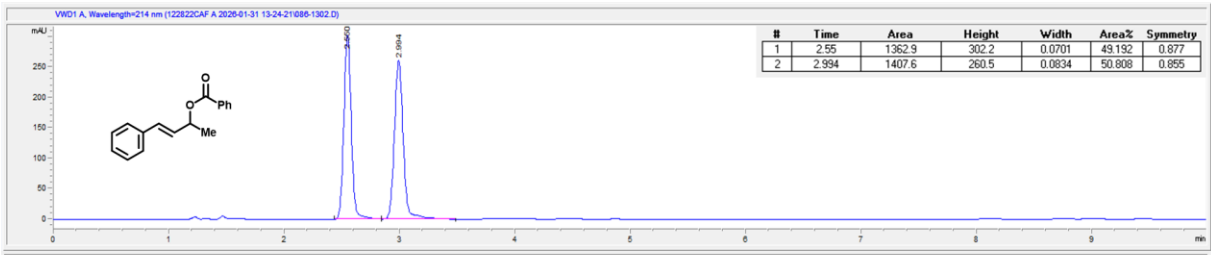


| # | Time | Area | Height | Width | Area% | Symmetry |
|---|---|---|---|---|---|---|
| 1 | 2.55 | 1362.9 | 302.2 | 0.0701 | 49.192 | 0.877 |
| 2 | 2.994 | 1407.6 | 260.5 | 0.0834 | 50.808 | 0.855 |

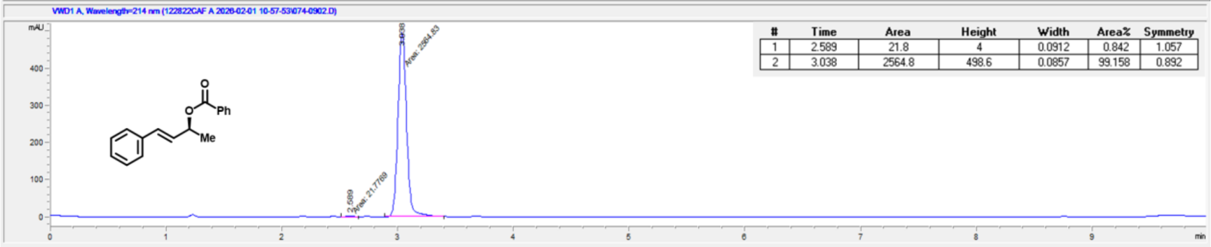


| # | Time | Area | Height | Width | Area% | Symmetry |
|---|---|---|---|---|---|---|
| 1 | 2.589 | 21.8 | 4 | 0.0912 | 0.842 | 1.057 |
| 2 | 3.038 | 2564.8 | 498.6 | 0.0857 | 99.158 | 0.892 |

---

(*S*,*E*)-1-phenyloct-1-en-3-yladamantane-1-carboxylate (**3r**)

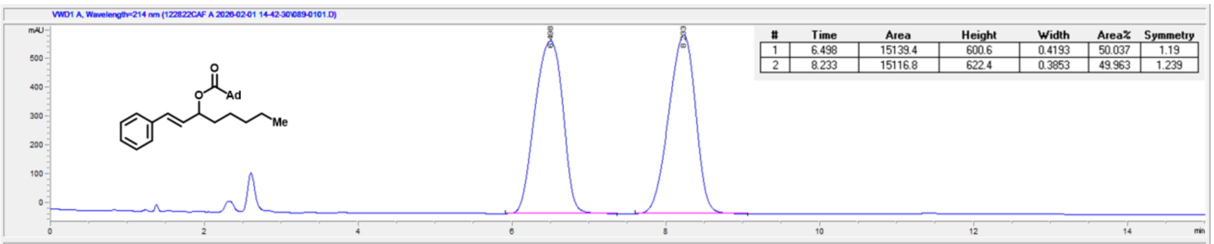


| # | Time | Area | Height | Width | Area% | Symmetry |
|---|---|---|---|---|---|---|
| 1 | 6.498 | 15139.4 | 600.6 | 0.4193 | 50.037 | 1.19 |
| 2 | 8.233 | 15116.8 | 622.4 | 0.3853 | 49.963 | 1.239 |

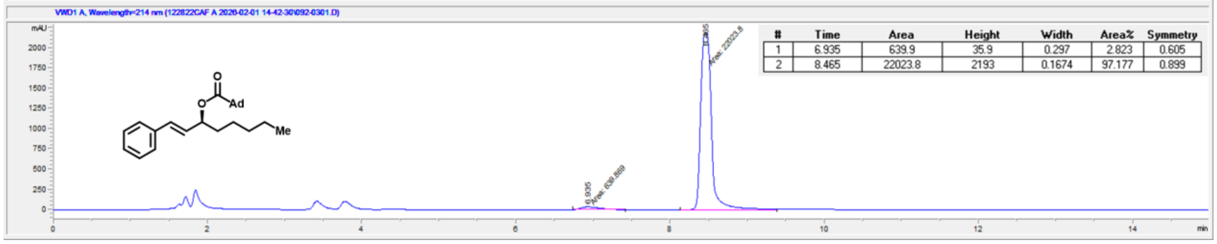


| # | Time | Area | Height | Width | Area% | Symmetry |
|---|---|---|---|---|---|---|
| 1 | 6.935 | 639.9 | 35.9 | 0.297 | 2.823 | 0.605 |
| 2 | 8.465 | 22023.8 | 2193 | 0.1674 | 97.177 | 0.899 |

(*S*,*E*)-5-(1,3-dioxan-2-yl)-1-phenylpent-1-en-3-yladamantane-1-carboxylate (**3s**)

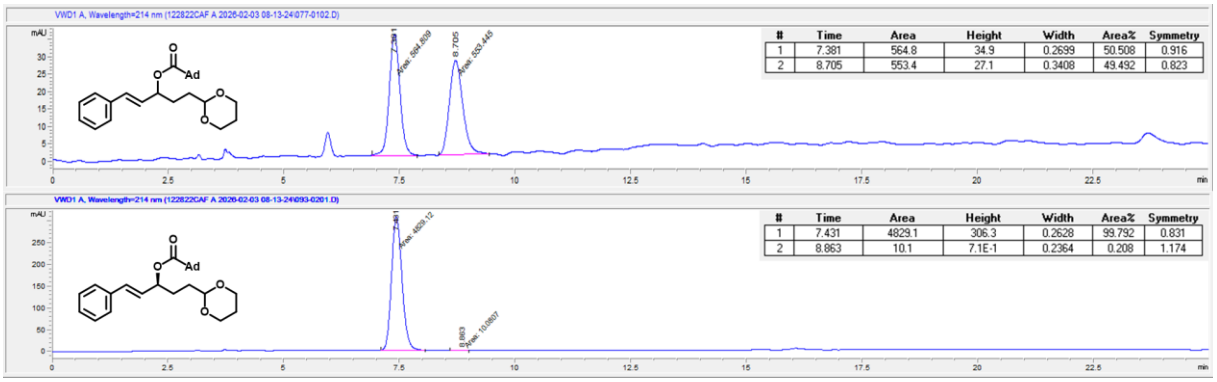

| # | Time | Area | Height | Width | Area% | Symmetry |
|---|---|---|---|---|---|---|
| 1 | 7.381 | 564.8 | 34.9 | 0.2699 | 50.508 | 0.916 |
| 2 | 8.705 | 553.4 | 27.1 | 0.3408 | 49.492 | 0.823 |

| # | Time | Area | Height | Width | Area% | Symmetry |
|---|---|---|---|---|---|---|
| 1 | 7.431 | 4829.1 | 306.3 | 0.2628 | 99.792 | 0.831 |
| 2 | 8.863 | 10.1 | 7.1E-1 | 0.2364 | 0.208 | 1.174 |

(*S*)-1-(naphthalen-2-yl)ethyl acetate (**3t**)

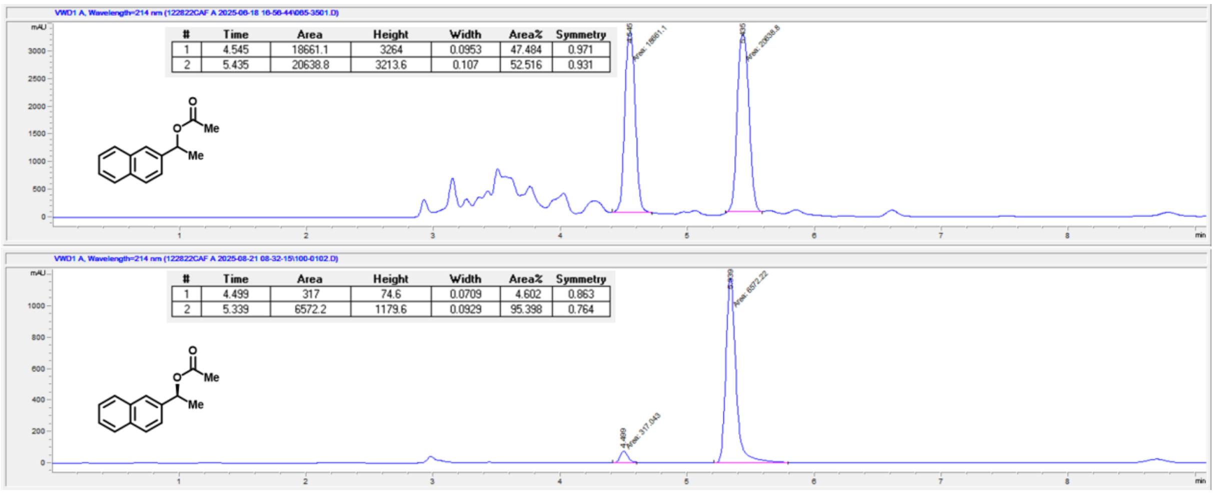

| # | Time | Area | Height | Width | Area% | Symmetry |
|---|---|---|---|---|---|---|
| 1 | 4.545 | 18661.1 | 3264 | 0.0953 | 47.484 | 0.971 |
| 2 | 5.435 | 20638.8 | 3213.6 | 0.107 | 52.516 | 0.931 |

| # | Time | Area | Height | Width | Area% | Symmetry |
|---|---|---|---|---|---|---|
| 1 | 4.499 | 317 | 74.6 | 0.0709 | 4.602 | 0.863 |
| 2 | 5.339 | 6572.2 | 1179.6 | 0.0929 | 95.398 | 0.764 |

(*S*)-1-(naphthalen-2-yl)ethyl 2-methylbenzo[d]oxazole-5-carboxylate (**3u**)

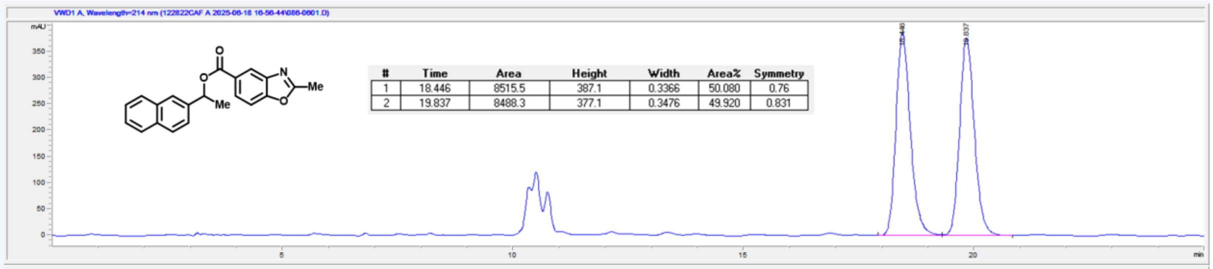

| # | Time | Area | Height | Width | Area% | Symmetry |
|---|---|---|---|---|---|---|
| 1 | 18.446 | 8515.5 | 387.1 | 0.3366 | 50.080 | 0.76 |
| 2 | 19.837 | 8488.3 | 377.1 | 0.3476 | 49.920 | 0.831 |

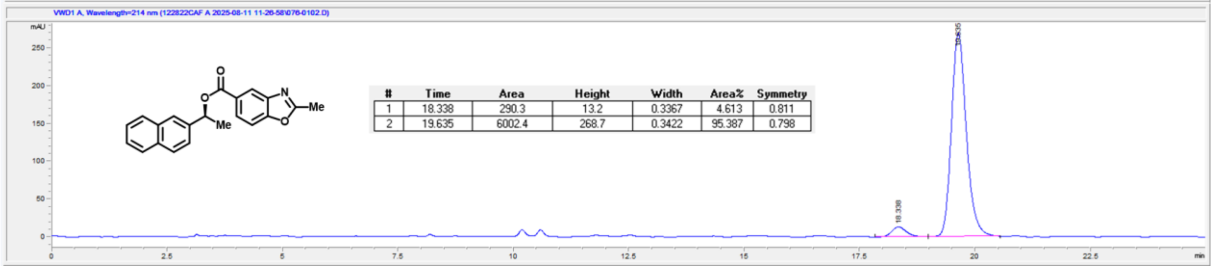

| # | Time | Area | Height | Width | Area% | Symmetry |
|---|---|---|---|---|---|---|
| 1 | 18.338 | 290.3 | 13.2 | 0.3367 | 4.613 | 0.811 |
| 2 | 19.635 | 6002.4 | 268.7 | 0.3422 | 95.387 | 0.798 |

---

(*S*)-1-(naphthalen-2-yl)ethyladamantane-1-carboxylate (**3v**)

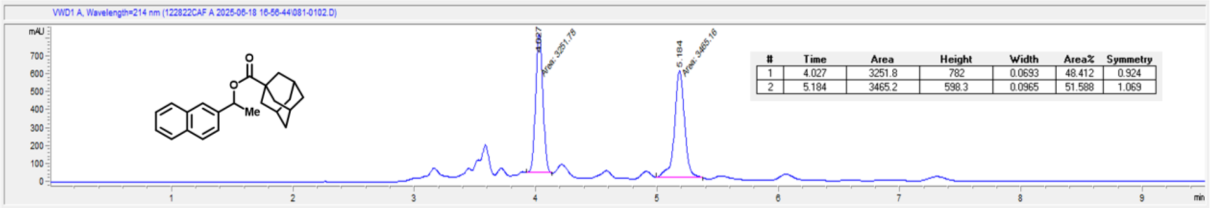

| # | Time | Area | Height | Width | Area% | Symmetry |
|---|---|---|---|---|---|---|
| 1 | 4.027 | 3251.8 | 782 | 0.0693 | 48.412 | 0.924 |
| 2 | 5.184 | 3465.2 | 598.3 | 0.0965 | 51.588 | 1.069 |

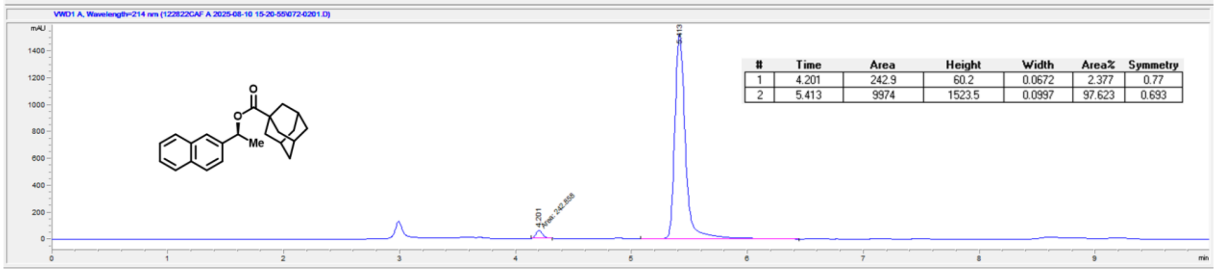

| # | Time | Area | Height | Width | Area% | Symmetry |
|---|---|---|---|---|---|---|
| 1 | 4.201 | 242.9 | 60.2 | 0.0672 | 2.377 | 0.77 |
| 2 | 5.413 | 9974 | 1523.5 | 0.0997 | 97.623 | 0.693 |

(*S*)-1-(naphthalen-2-yl)ethyl 4-bromobutanoate (**3w**)

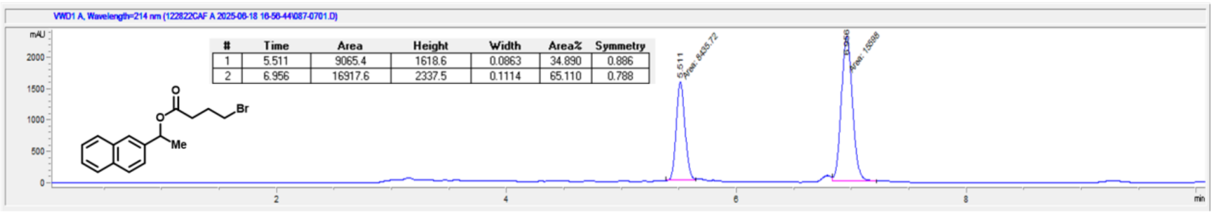

| # | Time | Area | Height | Width | Area% | Symmetry |
|---|---|---|---|---|---|---|
| 1 | 5.511 | 9065.4 | 1618.6 | 0.0863 | 34.890 | 0.886 |
| 2 | 6.956 | 16917.6 | 2337.5 | 0.1114 | 65.110 | 0.788 |

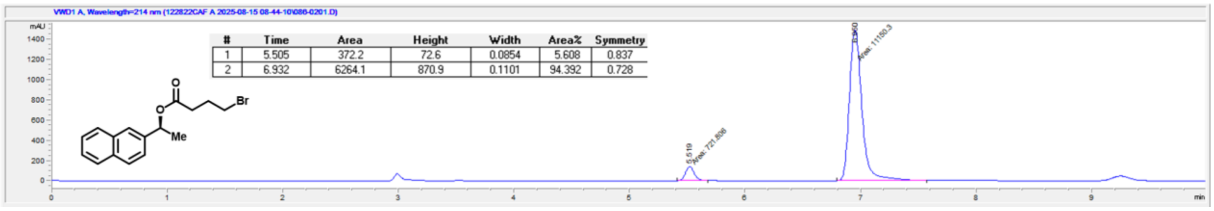

| # | Time | Area | Height | Width | Area% | Symmetry |
|---|---|---|---|---|---|---|
| 1 | 5.505 | 372.2 | 72.6 | 0.0854 | 5.608 | 0.837 |
| 2 | 6.932 | 6264.1 | 870.9 | 0.1101 | 94.392 | 0.728 |

(*S*)-1-(naphthalen-2-yl)ethyl 4,4-difluorocyclohexane-1-carboxylate (**3x**)

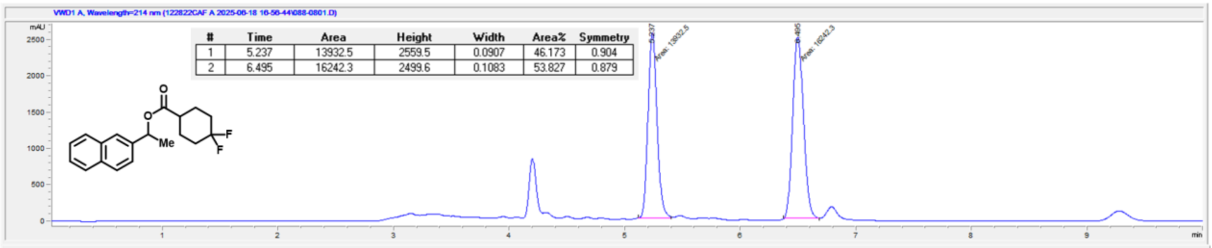

| # | Time | Area | Height | Width | Area% | Symmetry |
|---|---|---|---|---|---|---|
| 1 | 5.237 | 13932.5 | 2559.5 | 0.0907 | 46.173 | 0.904 |
| 2 | 6.495 | 16242.3 | 2499.6 | 0.1083 | 53.827 | 0.879 |

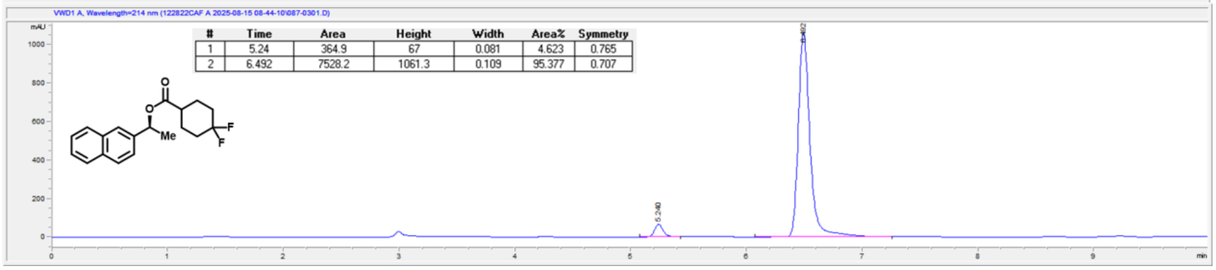

| # | Time | Area | Height | Width | Area% | Symmetry |
|---|---|---|---|---|---|---|
| 1 | 5.24 | 364.9 | 67 | 0.081 | 4.623 | 0.765 |
| 2 | 6.492 | 7528.2 | 1061.3 | 0.109 | 95.377 | 0.707 |

(*S*)-1-(naphthalen-2-yl)ethyl benzo[*b*]thiophene-3-carboxylate (**3y**)

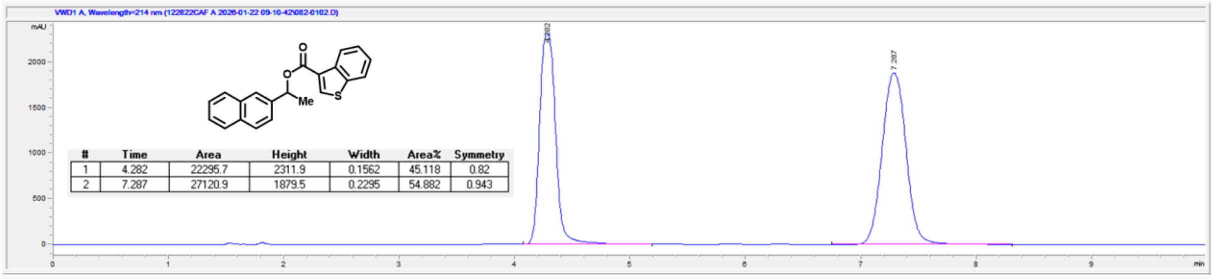

| # | Time | Area | Height | Width | Area% | Symmetry |
|---|---|---|---|---|---|---|
| 1 | 4.282 | 22295.7 | 2311.9 | 0.1562 | 45.118 | 0.82 |
| 2 | 7.287 | 27120.9 | 1879.5 | 0.2295 | 54.882 | 0.943 |

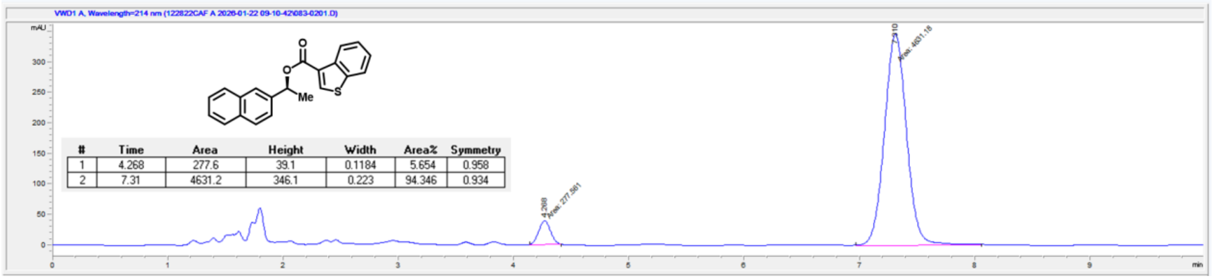

| # | Time | Area | Height | Width | Area% | Symmetry |
|---|---|---|---|---|---|---|
| 1 | 4.268 | 277.6 | 39.1 | 0.1184 | 5.654 | 0.958 |
| 2 | 7.31 | 4631.2 | 346.1 | 0.223 | 94.346 | 0.934 |

(*S*)-1-(naphthalen-2-yl)ethyl tetrahydro-2*H*-pyran-4-carboxylate (**3z**)

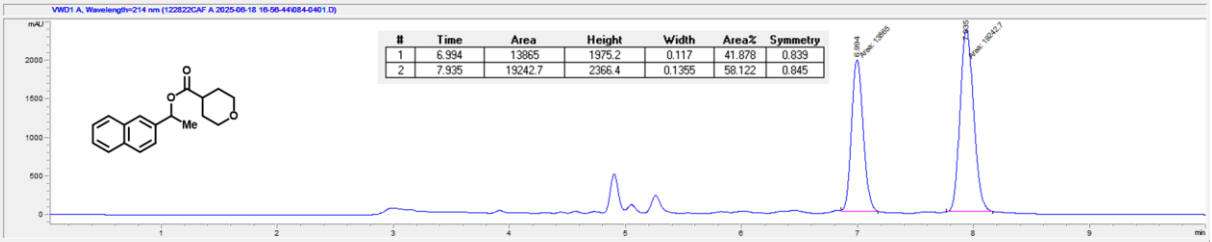

| # | Time | Area | Height | Width | Area% | Symmetry |
|---|---|---|---|---|---|---|
| 1 | 6.994 | 13865 | 1975.2 | 0.117 | 41.878 | 0.839 |
| 2 | 7.935 | 19242.7 | 2366.4 | 0.1355 | 58.122 | 0.845 |

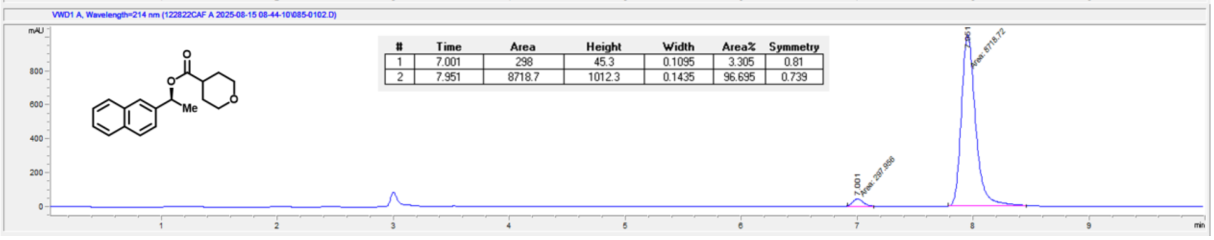

| # | Time | Area | Height | Width | Area% | Symmetry |
|---|---|---|---|---|---|---|
| 1 | 7.001 | 298 | 45.3 | 0.1095 | 3.305 | 0.81 |
| 2 | 7.951 | 8718.7 | 1012.3 | 0.1435 | 96.695 | 0.739 |

(*S*)-1-(naphthalen-2-yl)ethyl 1-(naphthalen-2-yl)ethyl cyclobutanecarboxylate (**3aa**)

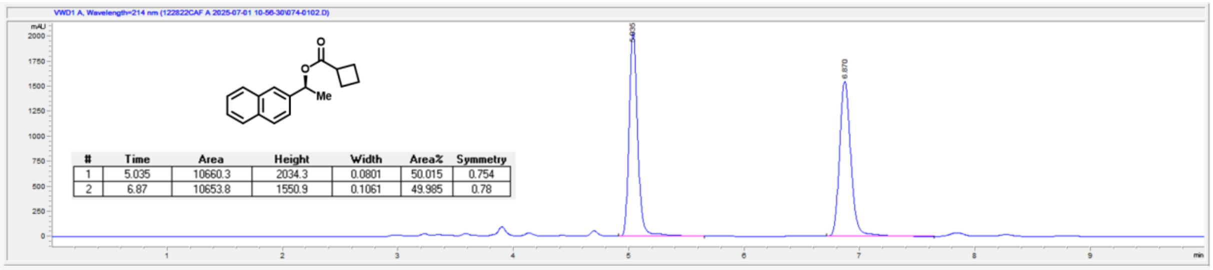

| # | Time | Area | Height | Width | Area% | Symmetry |
|---|---|---|---|---|---|---|
| 1 | 5.035 | 10660.3 | 2034.3 | 0.0801 | 50.015 | 0.754 |
| 2 | 6.87 | 10653.8 | 1550.9 | 0.1061 | 49.985 | 0.78 |

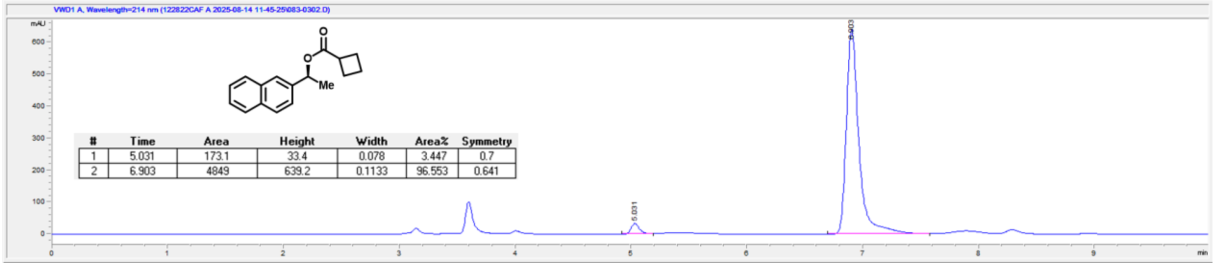

| # | Time | Area | Height | Width | Area% | Symmetry |
|---|---|---|---|---|---|---|
| 1 | 5.031 | 173.1 | 33.4 | 0.078 | 3.447 | 0.7 |
| 2 | 6.903 | 4849 | 639.2 | 0.1133 | 96.553 | 0.641 |

(*S*)-1-(naphthalen-2-yl)ethyl 3-methyloxetane-3-carboxylate (**3ab**)

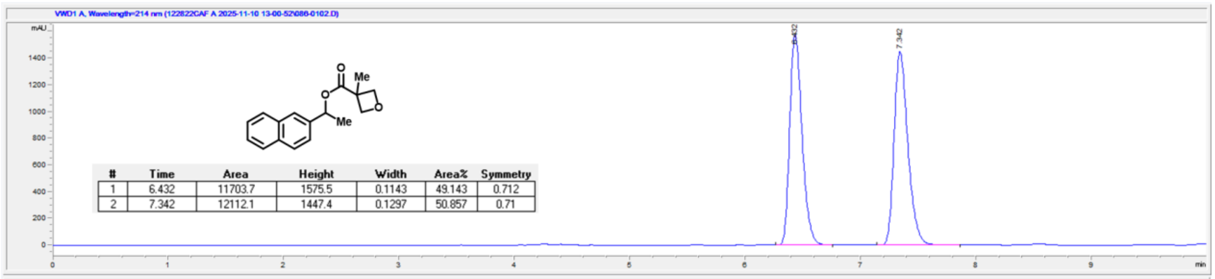

| # | Time | Area | Height | Width | Area% | Symmetry |
|---|---|---|---|---|---|---|
| 1 | 6.432 | 11703.7 | 1575.5 | 0.1143 | 49.143 | 0.712 |
| 2 | 7.342 | 12112.1 | 1447.4 | 0.1297 | 50.857 | 0.71 |

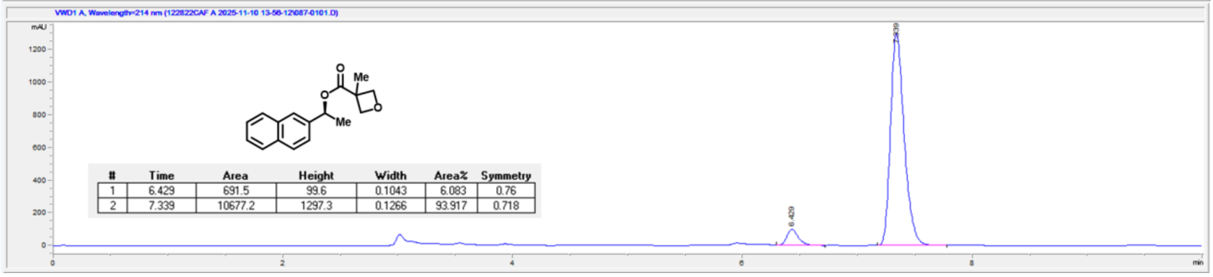

| # | Time | Area | Height | Width | Area% | Symmetry |
|---|---|---|---|---|---|---|
| 1 | 6.429 | 691.5 | 99.6 | 0.1043 | 6.083 | 0.76 |
| 2 | 7.339 | 10677.2 | 1297.3 | 0.1266 | 93.917 | 0.718 |

(*S*)-1-(naphthalen-2-yl)ethyl 4-(*N*,*N*-dipropylsulfamoyl)benzoate (**3ac**)

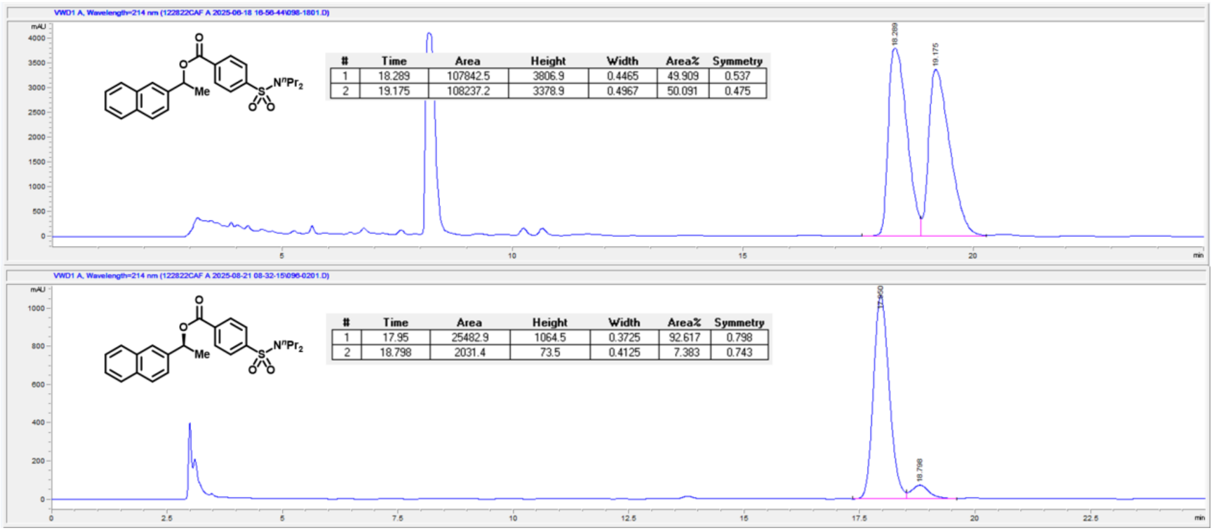


(*S*)-1-(naphthalen-2-yl)ethyl 2-(3-cyano-4-isobutoxyphenyl)-4-methylthiazole-5-carboxylate (**3ad**)

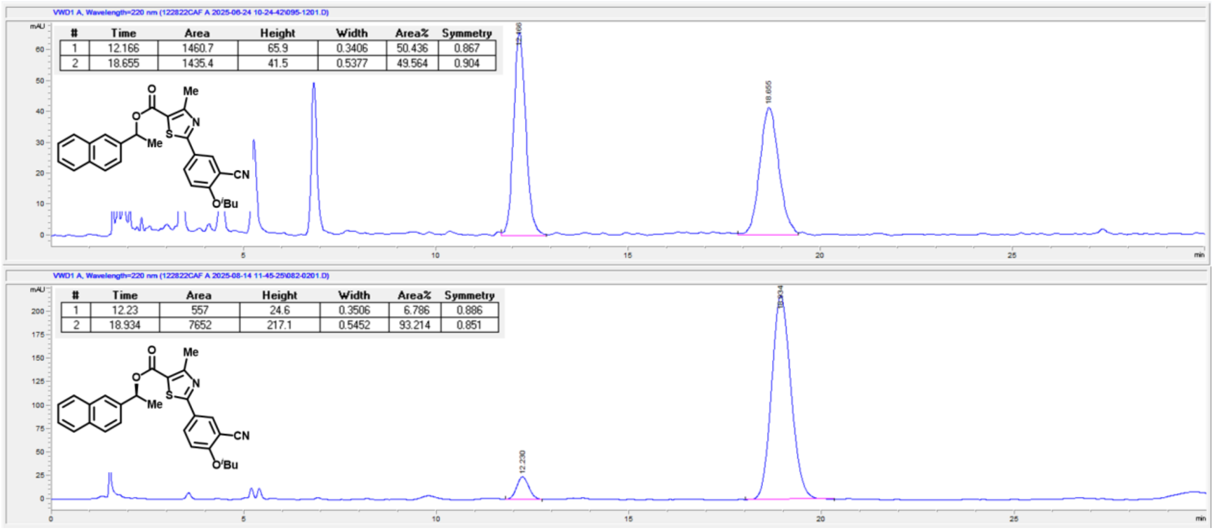

(*S*)-1-(naphthalen-2-yl)ethyl 2-(4-(2-(4-chlorobenzamido)ethyl)phenoxy)-2-methylpropanoate (**3ae**)

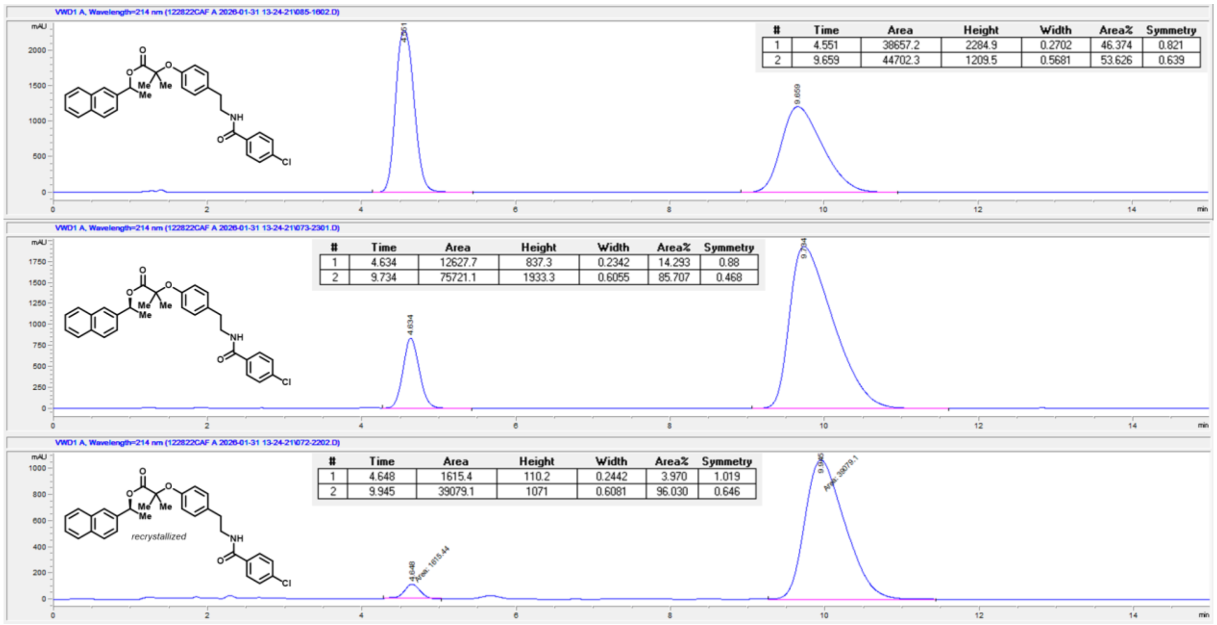


(*S*)-1-(naphthalen-2-yl)ethyl 3-(4,5-diphenyloxazol-2-yl)propanoate (**3af**)

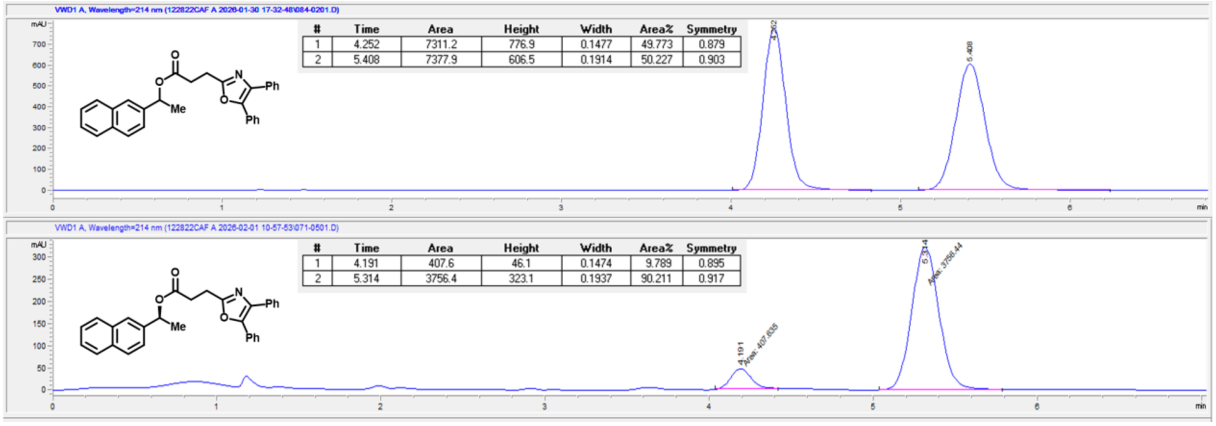

(*S*)-1-(naphthalen-2-yl)ethyl 4-(4-(bis(2-chloroethyl)amino)phenyl)butanoate (**3ag**)

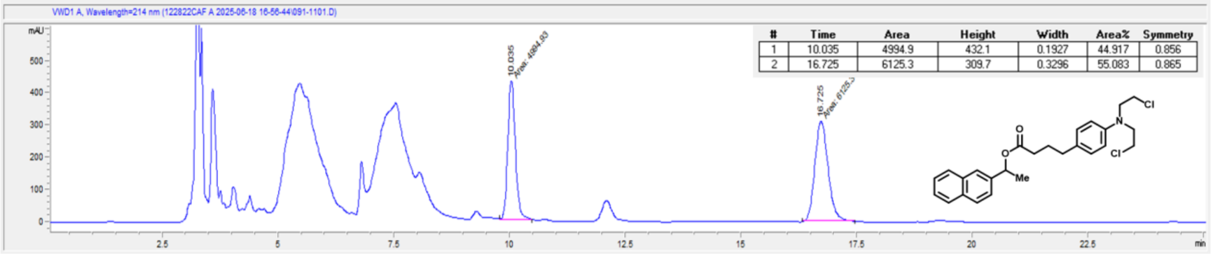

| # | Time | Area | Height | Width | Area% | Symmetry |
|---|---|---|---|---|---|---|
| 1 | 10.035 | 4994.9 | 432.1 | 0.1927 | 44.917 | 0.856 |
| 2 | 16.725 | 6125.3 | 309.7 | 0.3296 | 55.083 | 0.865 |

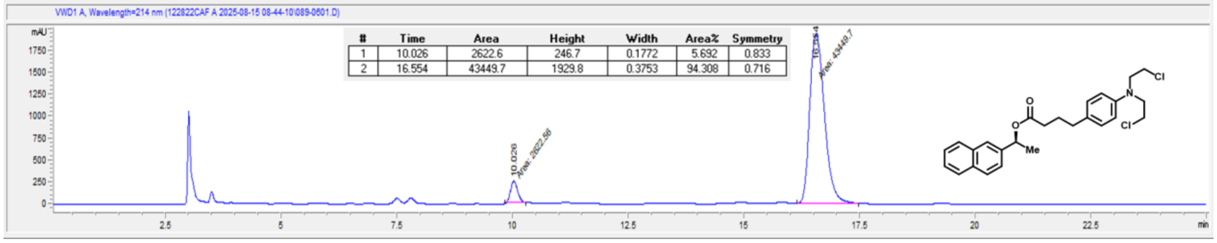

| # | Time | Area | Height | Width | Area% | Symmetry |
|---|---|---|---|---|---|---|
| 1 | 10.026 | 2622.6 | 246.7 | 0.1772 | 5.692 | 0.833 |
| 2 | 16.554 | 43449.7 | 1929.8 | 0.3753 | 94.308 | 0.716 |

(*S*)-1-(naphthalen-2-yl)ethyl 2-(1-methyl-5-(4-methylbenzoyl)-1*H*-pyrrol-2-yl)acetate (**3ah**)

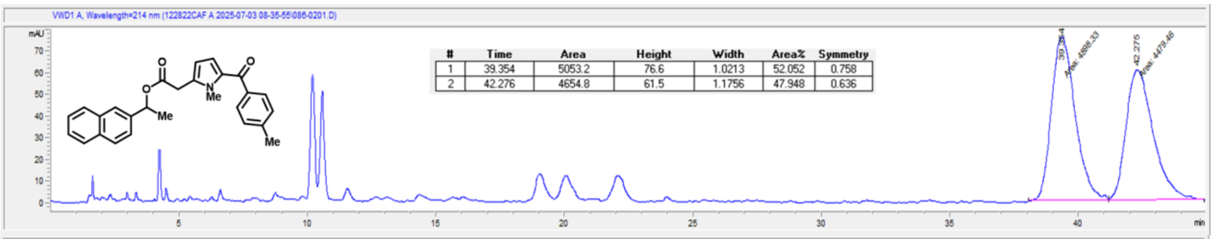

| # | Time | Area | Height | Width | Area% | Symmetry |
|---|---|---|---|---|---|---|
| 1 | 39.354 | 5053.2 | 76.6 | 1.0213 | 52.052 | 0.758 |
| 2 | 42.276 | 4654.8 | 61.5 | 1.1756 | 47.948 | 0.636 |

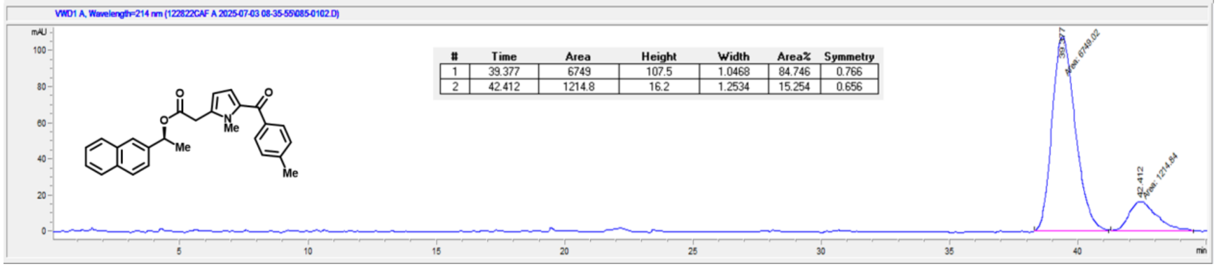

| # | Time | Area | Height | Width | Area% | Symmetry |
|---|---|---|---|---|---|---|
| 1 | 39.377 | 6749 | 107.5 | 1.0468 | 84.746 | 0.766 |
| 2 | 42.412 | 1214.8 | 16.2 | 1.2534 | 15.254 | 0.656 |

(*S*)-1-(naphthalen-2-yl)ethyl 2-(1-(4-chlorobenzoyl)-2-methyl-1*H*-indol-3-yl)acetate (**3ai**)

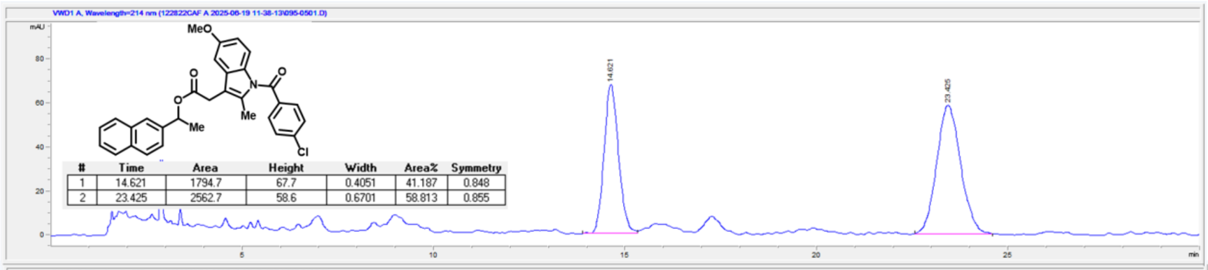

| # | Time | Area | Height | Width | Area% | Symmetry |
|---|---|---|---|---|---|---|
| 1 | 14.621 | 1794.7 | 67.7 | 0.4051 | 41.187 | 0.848 |
| 2 | 23.425 | 2562.7 | 58.6 | 0.6701 | 58.813 | 0.855 |

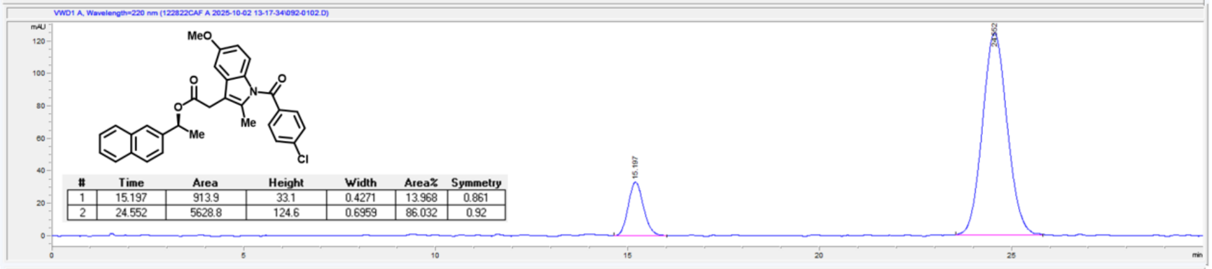

| # | Time | Area | Height | Width | Area% | Symmetry |
|---|---|---|---|---|---|---|
| 1 | 15.197 | 913.9 | 33.1 | 0.4271 | 13.968 | 0.861 |
| 2 | 24.552 | 5628.8 | 124.6 | 0.6959 | 86.032 | 0.92 |

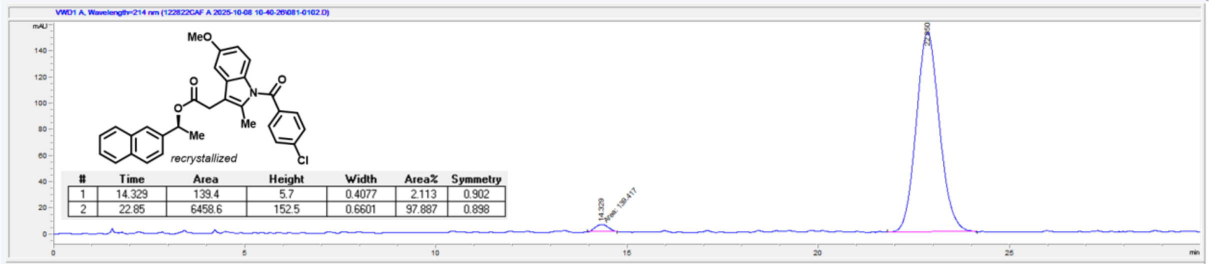

| # | Time | Area | Height | Width | Area% | Symmetry |
|---|---|---|---|---|---|---|
| 1 | 14.329 | 139.4 | 5.7 | 0.4077 | 2.113 | 0.902 |
| 2 | 22.85 | 6458.6 | 152.5 | 0.6601 | 97.887 | 0.898 |

(*S*)-1-(naphthalen-2-yl)ethyl 5-(2,5-dimethylphenoxy)-2,2-dimethylpentanoate (**3aj**)

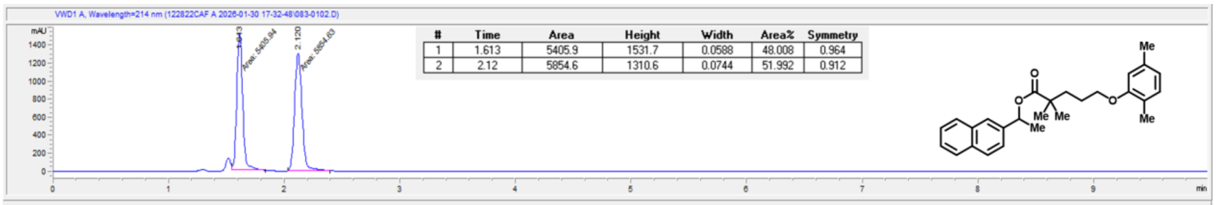

| # | Time | Area | Height | Width | Area% | Symmetry |
|---|---|---|---|---|---|---|
| 1 | 1.613 | 5405.9 | 1531.7 | 0.0588 | 48.008 | 0.964 |
| 2 | 2.12 | 5854.6 | 1310.6 | 0.0744 | 51.992 | 0.912 |

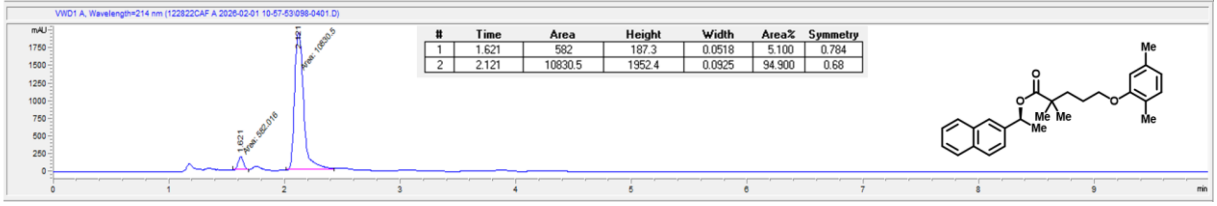

| # | Time | Area | Height | Width | Area% | Symmetry |
|---|---|---|---|---|---|---|
| 1 | 1.621 | 582 | 187.3 | 0.0518 | 5.100 | 0.784 |
| 2 | 2.121 | 10830.5 | 1952.4 | 0.0925 | 94.900 | 0.68 |

(*S*)-1-(naphthalen-2-yl)ethyl-2-(4-2,2-dichlorocyclopropylphenoxy)-2-methylpropanoate (**3ak**)

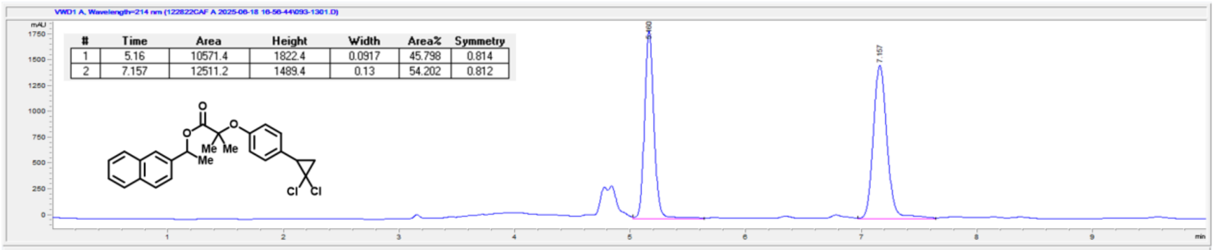


| # | Time | Area | Height | Width | Area% | Symmetry |
|---|---|---|---|---|---|---|
| 1 | 5.16 | 10571.4 | 1822.4 | 0.0917 | 45.798 | 0.814 |
| 2 | 7.157 | 12511.2 | 1489.4 | 0.13 | 54.202 | 0.812 |

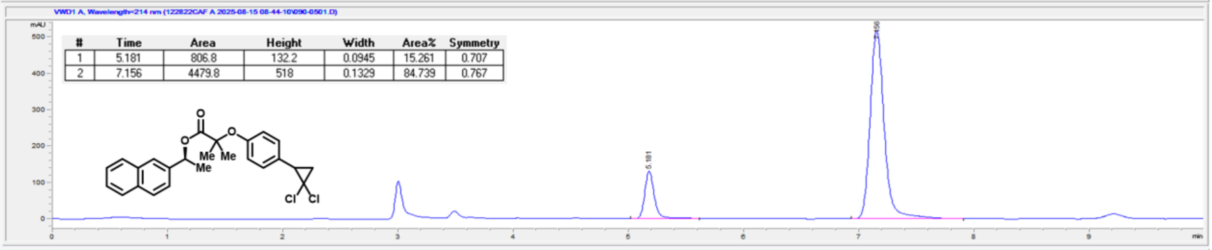


| # | Time | Area | Height | Width | Area% | Symmetry |
|---|---|---|---|---|---|---|
| 1 | 5.181 | 806.8 | 132.2 | 0.0945 | 15.261 | 0.707 |
| 2 | 7.156 | 4479.8 | 518 | 0.1329 | 84.739 | 0.767 |

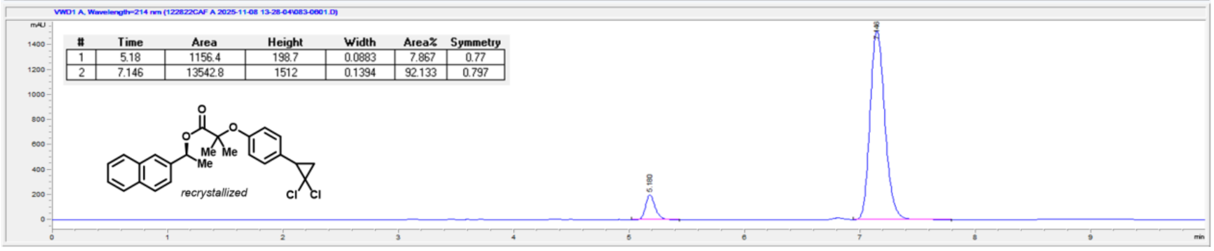


| # | Time | Area | Height | Width | Area% | Symmetry |
|---|---|---|---|---|---|---|
| 1 | 5.18 | 1156.4 | 198.7 | 0.0883 | 7.867 | 0.77 |
| 2 | 7.146 | 13542.8 | 1512 | 0.1394 | 92.133 | 0.797 |

(*S*)-1-(naphthalen-2-yl)ethyl 6-(3-adamantan-1-yl)-4-methoxyphenyl)-2-naphthoate (**3al**)

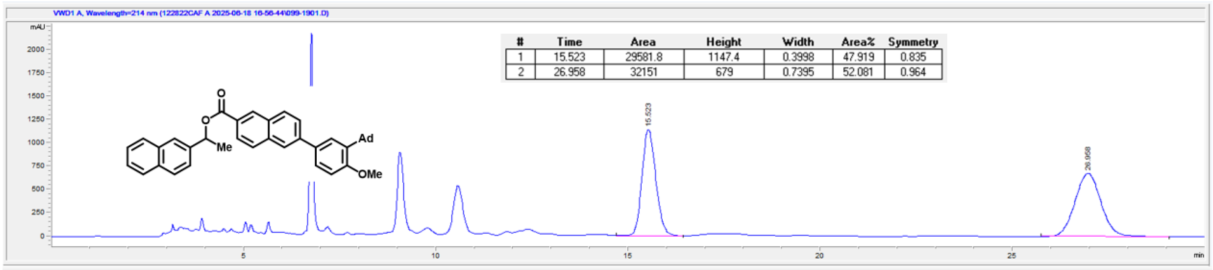


| # | Time | Area | Height | Width | Area% | Symmetry |
|---|---|---|---|---|---|---|
| 1 | 15.523 | 29581.8 | 1147.4 | 0.3998 | 47.919 | 0.835 |
| 2 | 26.958 | 32151 | 679 | 0.7395 | 52.081 | 0.964 |

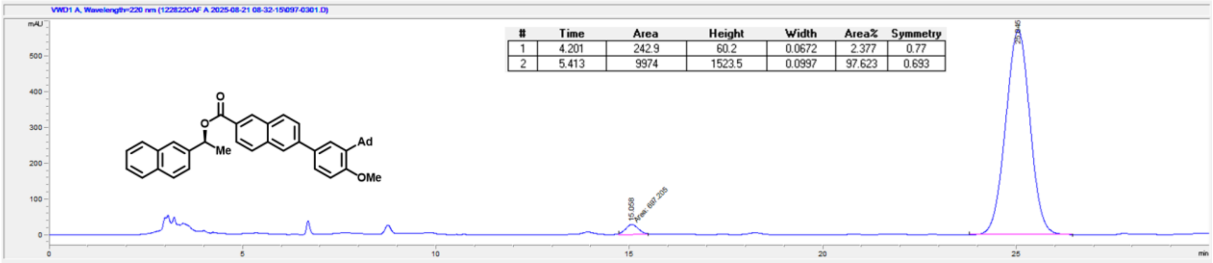


| # | Time | Area | Height | Width | Area% | Symmetry |
|---|---|---|---|---|---|---|
| 1 | 4.201 | 242.9 | 60.2 | 0.0672 | 2.377 | 0.77 |
| 2 | 5.413 | 9974 | 1523.5 | 0.0997 | 97.623 | 0.693 |

(*R*)-1-(naphthalen-2-yl)-3-phenylpropan-1-ol (**7**)

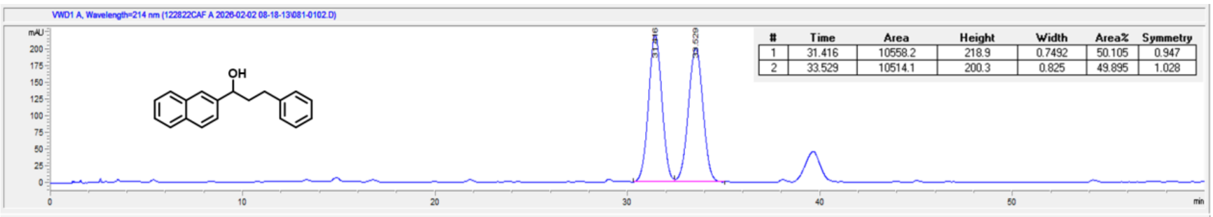


| # | Time | Area | Height | Width | Area% | Symmetry |
|---|---|---|---|---|---|---|
| 1 | 31.416 | 10558.2 | 218.9 | 0.7492 | 50.105 | 0.947 |
| 2 | 33.529 | 10514.1 | 200.3 | 0.825 | 49.895 | 1.028 |

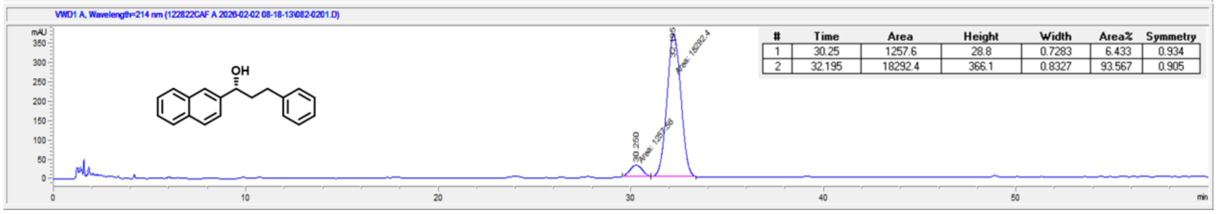


| # | Time | Area | Height | Width | Area% | Symmetry |
|---|---|---|---|---|---|---|
| 1 | 30.25 | 1257.6 | 28.8 | 0.7283 | 6.433 | 0.934 |
| 2 | 32.195 | 18292.4 | 366.1 | 0.8327 | 93.567 | 0.905 |

## 9. Computational Details

### 9.1 General Information

Prior to geometry optimizations, all starting geometries for metal complexes intermediates and transition states were obtained by performing conformational analysis using the global optimizer algorithm (GOAT) using the extended tight binding (GFN2-xTB) Hamiltonian[29, 30] with ORCA 6.0.0.[31] When relevant, a set of conformers was selected, otherwise, only the lowest energy conformer was retained. All geometry optimizations were computed with Gaussian 16 (revision C.01),[32] using the Becke-Johnson damped Grimme D3(BJ) dispersion-corrected PW6B95 functional[33-35]. All atoms were described with the double-ζ def2-SVP basis set.[36] Due to persistent SCF convergence and geometry convergence issues when attempting to include the solvent during optimization protocol (modeled by the implicit solvent model based on density (SMD)[37] with corresponding dielectric constant for either DCE, chlorobenzene or toluene ($\varepsilon = 10.125$, $\varepsilon = 5.697$, and $\varepsilon = 2.374$ respectively)), all optimizations were performed in gas phase. Analytical frequency calculations were carried out to confirm minima as displaying no imaginary frequency. Transition states (TSs) all present one negative frequency and were validated by running intrinsic reaction coordinate (IRC) calculations and subsequent optimizations, connecting them to adjacent minima. Following geometry optimizations, single point energy corrections were performed including the triple-ζ basis set Def2-TZVP[36] and implicit SMD solvation model of chlorobenzene. Final free energies were obtained after applying the quasi-harmonic rigid-rotor approximation to vibrational entropies proposed by Grimme using GoodVibes[38, 39] with a 50 $cm^{-1}$ cut-off and by converting to a 1 molar standard state. The stabilities of all unrestricted-Kohn Sham (UKS) densities/wavefunctions obtained for metal complexes were assessed using the “stable=opt” keywords. All 3D representations of key stationary points were obtained with CYLview.[40] NCI calculations were performed based on the geometric approach and visualized with VMD[41] using the NCIPLOT.[42, 43] Energy decomposition analysis was performed using the Absolutely Localized Molecular Orbital Energy Decomposition Analysis (ALMO-EDA2) method of Head-Gordon and co-workers as implemented in Q-Chem 6.0.2.[44, 45]

## 9.2 Speciation Study of Benzoic Acid Under the Reaction Conditions

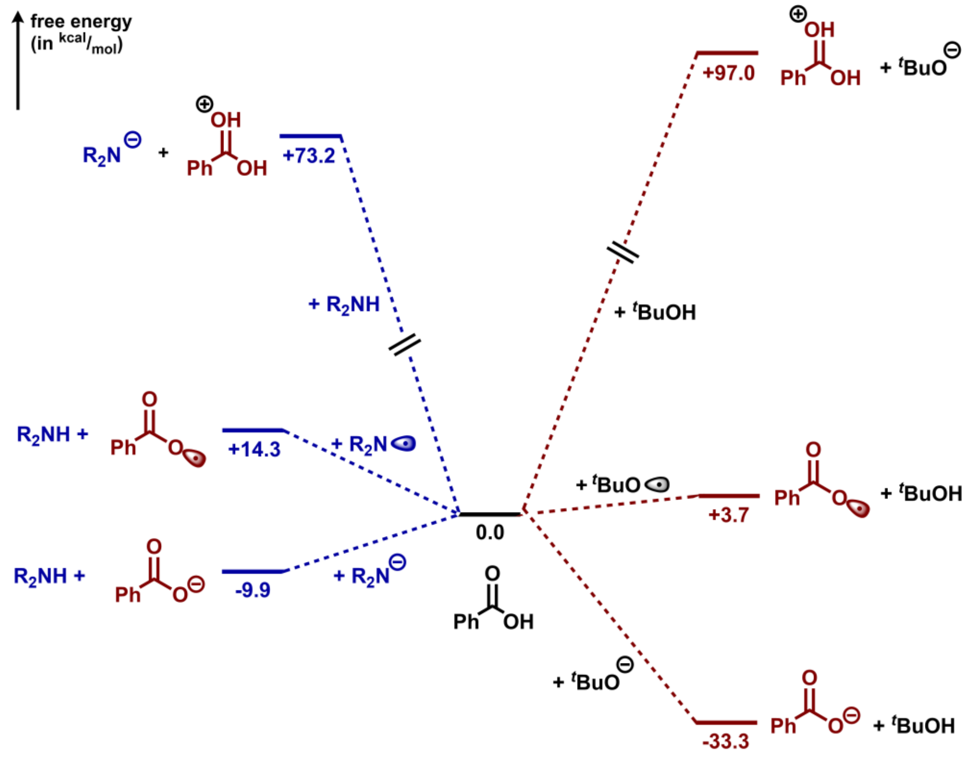


**Scheme S1**. Computed speciation study of benzoic acid under the reaction conditions (Gibbs free energies in kcal/mol).

A computed speciation study shows that on thermodynamic grounds, benzoic acid is more likely to form a radical with the *t*BuO• than with **4e** ($R_2N$•), reflective of the stronger O–H vs. N–H BDFE values. It also suggests that deprotonation of the benzoic acid by *t*BuO⁻ is thermodynamically driven, affording *t*BuOH. As presented in Figure 2A (step c), *t*BuOH then reacts with **12** to regenerate **4e**. As expected, cationic protonated benzoic acid is thermodynamically irrelevant. $R_2N^-$ is unlikely to be formed under the reaction conditions but would be able to deprotonate benzoic acid.

## 9.3 Proposed Mechanism for N-radical-mediated C–B Bond Homolysis

*9.3.1 O-Radical-Mediated C–B Homolysis*

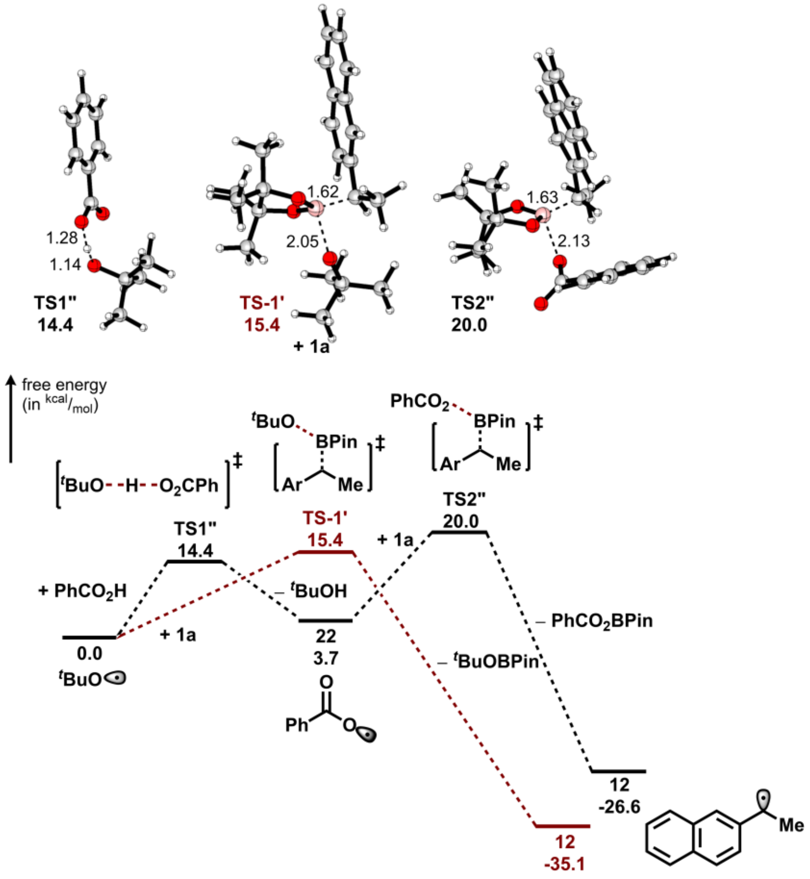


**Scheme S2**. Computed potential energy surfaces for O-radical mediated C–B bond homolysis (Gibbs free energies in kcal/mol). Geometries of key stationary points are shown with selected distances in Å.

The single-electron activation of **1a** by either an alkoxy radical or an aryl carboxyl radical (**22**) is highly exergonic (ΔG = −26.6 kcal/mol and −35.1 kcal/mol respectively). The activation barrier for direct *t*-butoxy-radical-mediated C–B bond homolysis (red pathway) proceeds through a barrier of $\Delta G^{\ddagger}$(**TS-1'**) = 15.4 kcal/mol, whereas the competitive aryl carboxyl radical generation via HAT (black pathway, $\Delta G^{\ddagger}$(**TS-1"**) = 14.4 kcal/mol), is followed by a more kinetically demanding C–B bond homolysis step ($\Delta G^{\ddagger}$(**TS-2"**) = 20.0 kcal/mol), presumably due to the greater resonance stabilization of this radical species.

*9.3.2 N-Radical-Mediated C–B Homolysis*

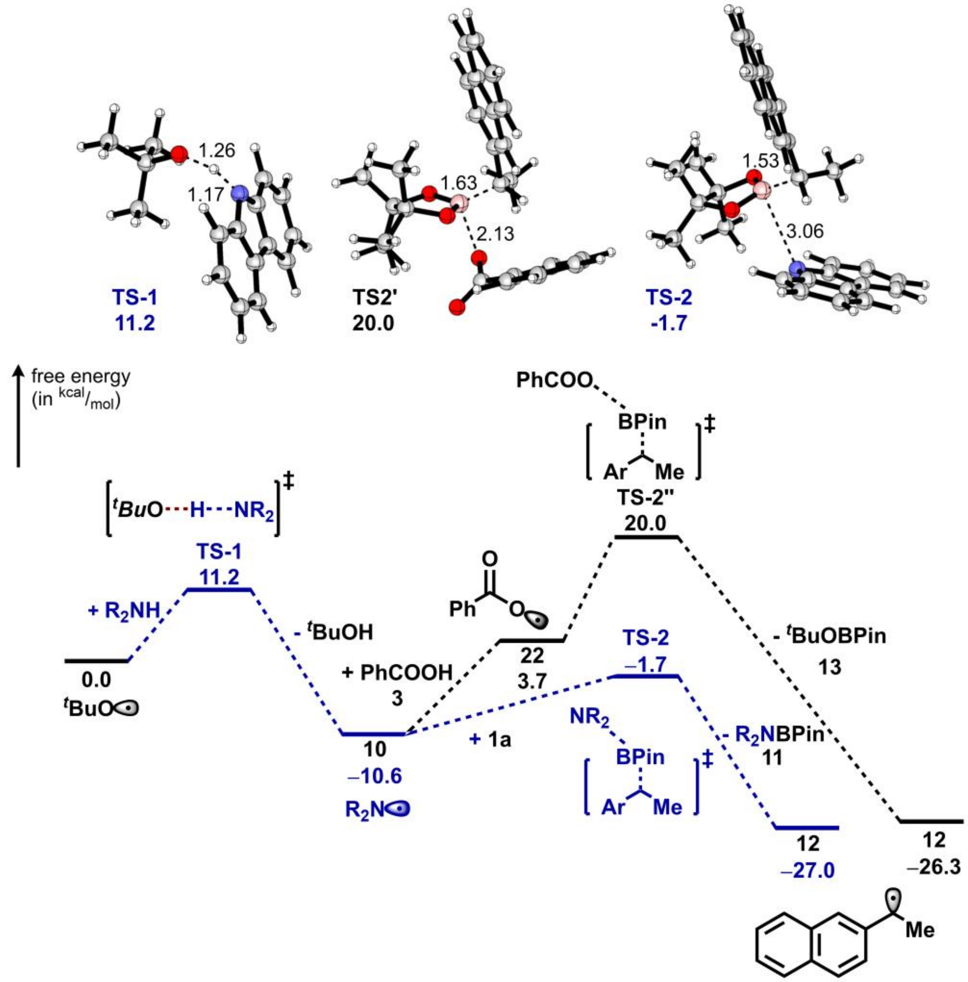


**Scheme S3**. Computed potential energy surfaces for N-radical mediated C–B bond homolysis (Gibbs free energies in kcal/mol). Geometries of key stationary points are shown with selected distances in Å.

A carbazole-derived N-centered radical (**10**) can be thermodynamically generated by HAT between the carbazole and an alkoxy radical ($\Delta G^‡$(**TS-1**) = 11.2 kcal/mol and $\Delta G$(**10**) = −10.6 kcal/mol). The N-radical can either perform direct radical-mediated C–B bond homolysis (blue pathway) with a barrier of $\Delta G^‡$(**TS-2**) = 8.9 kcal/mol, or perform HAT with the benzoic acid (black pathway), in which case C–B bond homolysis proceeds via $\Delta G^‡$(**TS-2”**) = 20.0 kcal/mol (also presented in **Scheme S2**).

## 9.4 Proposed Mechanisms for Transmetalation with Benzoic Acid

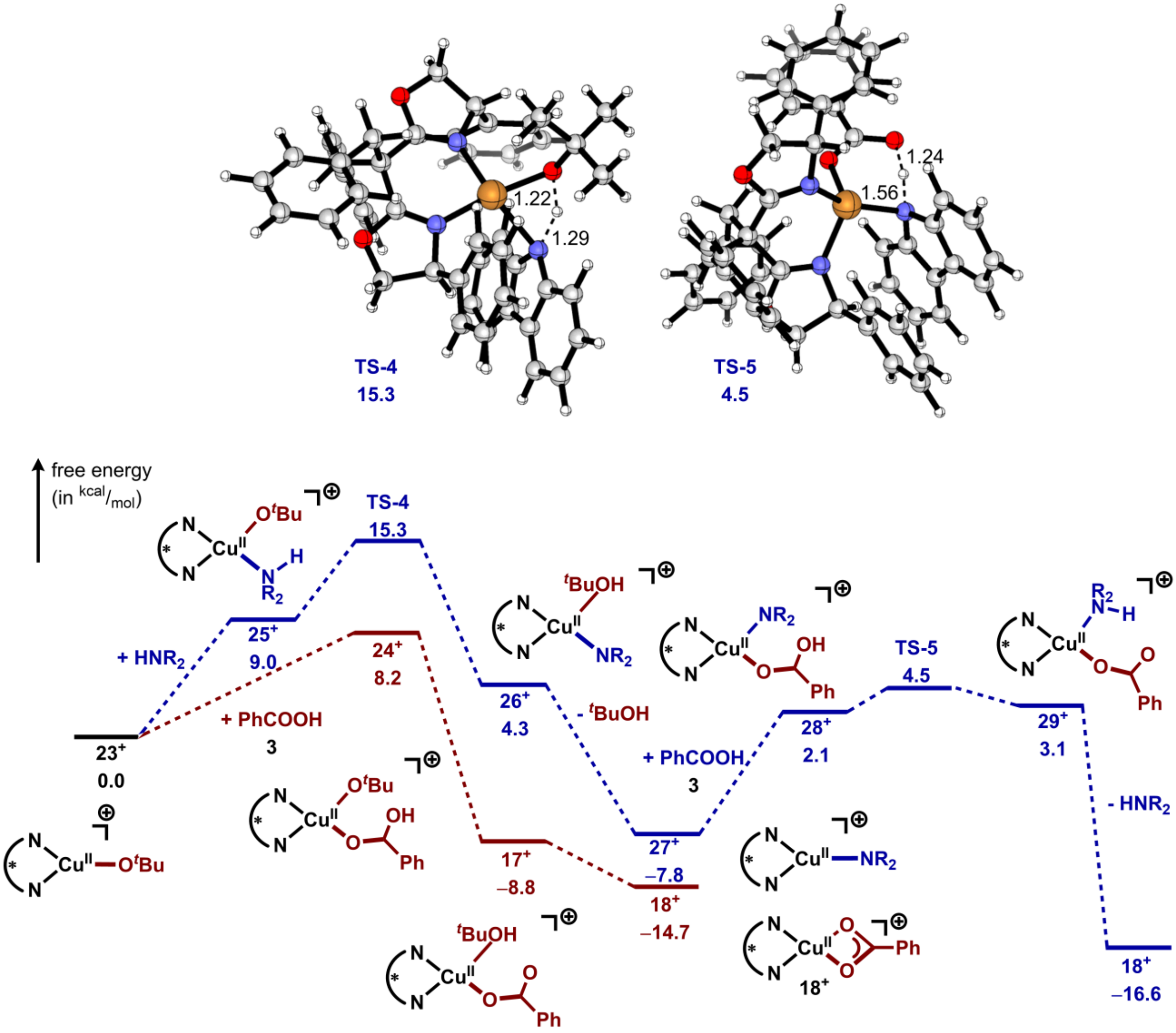


**Scheme S4**. Computed potential energy surfaces for the transmetalation of benzoic acid from **23**$^{+}$ under the reaction conditions (Gibbs free energies in kcal/mol). Geometries of key stationary points are shown with selected distances in Å.

Two pathways were investigated for the transmetalation of benzoic acid from **23**$^{+}$. Coordination of benzoic acid to **23**$^{+}$ followed by deprotonation of benzoic acid by the coordinated $^{t}$BuO was found to be barrierless and exothermic by 14.7 kcal/mol (red pathway), whereas a mechanism involving the carbazole was found to proceed through **TS-4** at 15.3 kcal/mol (blue pathway). The involvement of carbazole in transmetalation was therefore ruled out.

### 9.5 Investigations of Cu–O Bond Formation via Radical Recombination (Outer-Sphere)

We attempted to locate the outer-sphere C–O bond-forming TSs on either the triplet or singlet energy surface:[46, 47]

(1) Triplet: We identified the outer-sphere C–O bond-forming TSs with triplet spin states. Comparing these new TSs with their inner-sphere counterparts (Scheme S5) suggested that they are energetically disfavored ($\Delta\Delta G^{\ddagger}$ > 16 kcal/mol).

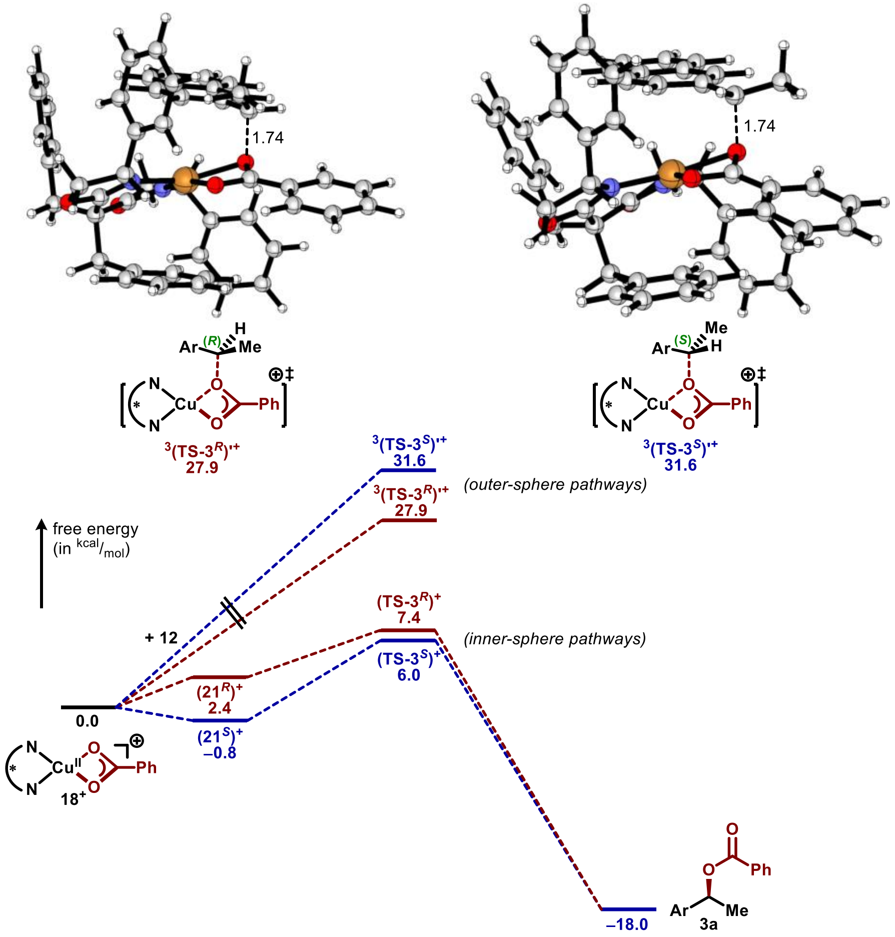


**Scheme S5**. Comparing the lowest-energy conformers of inner-sphere and outer-sphere (triplet) C–O bond formation transition states (Gibbs free energies in kcal/mol). Geometries of key stationary points are shown with selected distances in Å.

(2) Singlet: We reasoned that optimization for outer-sphere C–O bond-forming TSs on the singlet surface could lead to two scenarios: open-shell singlet (OSS) vs. closed-shell singlet (CSS). While differing only by the spin density distribution, an OSS TS indicates the direct recombination of an alkyl radical with benzoate ligand (outer-sphere radical pathway) whereas a CSS TS reflects the

recombination of a carbocation with benzoate ligand (radical-polar crossover SET pathway). All of our attempts, including testing different levels of theory, resulted in the collapse to closed-shell singlet (CSS), indicating the formation of a carbocation following SET. Stability checks were performed on each CCS TSs and found to be stable, i.e. there are no low-lying corresponding OSS TSs. Accordingly, we concluded that the outer-sphere pathway is outcompeted by the radical-polar crossover process in our current system.

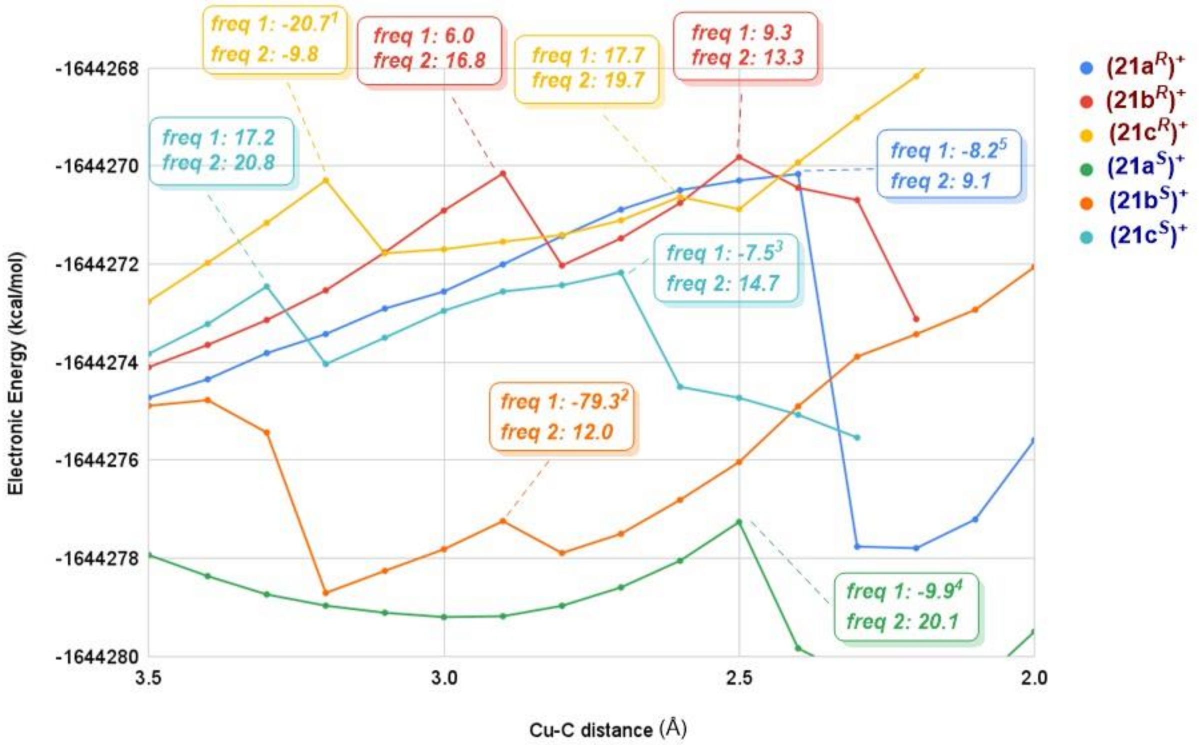


**Scheme S6**. Electronic energy plots (in kcal/mol) as a function of the Cu···C distance for each conformer of **21**$^+$. Local maxima were recomputed for the given Cu···C distance at a more refined level of theory and the first two frequencies are displayed (cm$^{-1}$).

To investigate the possible existence of an OSS TS for Cu–C bond coupling and formation of the Cu(III) intermediate **21**$^+$, scan calculations were performed along the Cu···C distance on the OSS surface for all conformers. On the basis that the sum of the atomic radii of Cu (1.32 Å) and C (0.76 Å)[48] is equal to 2.08 Å, and that the Cu–C bonds range between 2.06 Å and 2.16 Å across all conformers of **21**$^+$, all scans were performed for Cu···C distances comprised between 3.5 Å and 2.0 Å. The "*Opt=loose*" keyword was used to perform those calculations in a reasonable time frame. Each local maximum was reoptimized with default convergence criteria and constraining

the Cu···C distance at the corresponding length. In most cases, vibrational frequency analysis revealed that those geometries did not correspond to a transition state. When an imaginary frequency was obtained those were carefully analyzed and full TS optimizations were performed if necessary. Details of the procedure adopted for each imaginary frequency identified can be found in the following:

1. This imaginary frequency corresponds to the formation of a π-π stacking interaction between the aryl carboxylic radical and the coordinated benzoic acid. The geometry for Cu···C = 3.2 Å was therefore ruled out as TS for the formation of **(21c$^{R}$)$^{+}$**.
2. This imaginary frequency corresponds to a methyl rotation. Full TS optimization was not performed and the geometry for Cu···C = 2.9 Å was therefore ruled out as TS for the formation of **(21b$^{S}$)$^{+}$**.
3. Full TS optimization collapsed to the Cu(III) intermediate **(21c$^{S}$)$^{+}$** rapidly. We therefore concluded to its barrierless formation and ruled out the geometry for Cu···C = 2.7 Å as TS for the formation of **(21c$^{S}$)$^{+}$**.
4. Full TS optimization displayed an imaginary frequency of -16.6 cm$^{-1}$. Not only is this frequency extremely small, but IRC calculations confirm that this frequency corresponds to a translation of the aryl carboxyl radical. The geometry for Cu···C = 2.5 Å was therefore ruled out as TS for the formation of **(21a$^{S}$)$^{+}$**.
5. This imaginary frequency corresponds to a general motion of the phenyl groups of the bis-oxazoline ligand. Full TS optimization was not performed and the geometry for Cu···C = 2.4 Å was therefore ruled out as TS for the formation of **(21a$^{R}$)$^{+}$**.

To conclude, we were not able to locate an OSS TS for Cu–C coupling and formation of a Cu(III) intermediate **21**$^{+}$. While the absence of evidence is not evidence of absence, we believe that the above discussion detailing our concerted efforts illustrate the unlikeliness that open-shell radical coupling TSs have an effect on selectivity. Furthermore, similar conclusions were previously reported in related transformations.[46, 49-52]

## 9.6 Proposed Mechanism for Cu-mediated C–O Bond Formation

### 9.6.1 C–O Bond Formation via Inner-Sphere Pathways for all Conformers

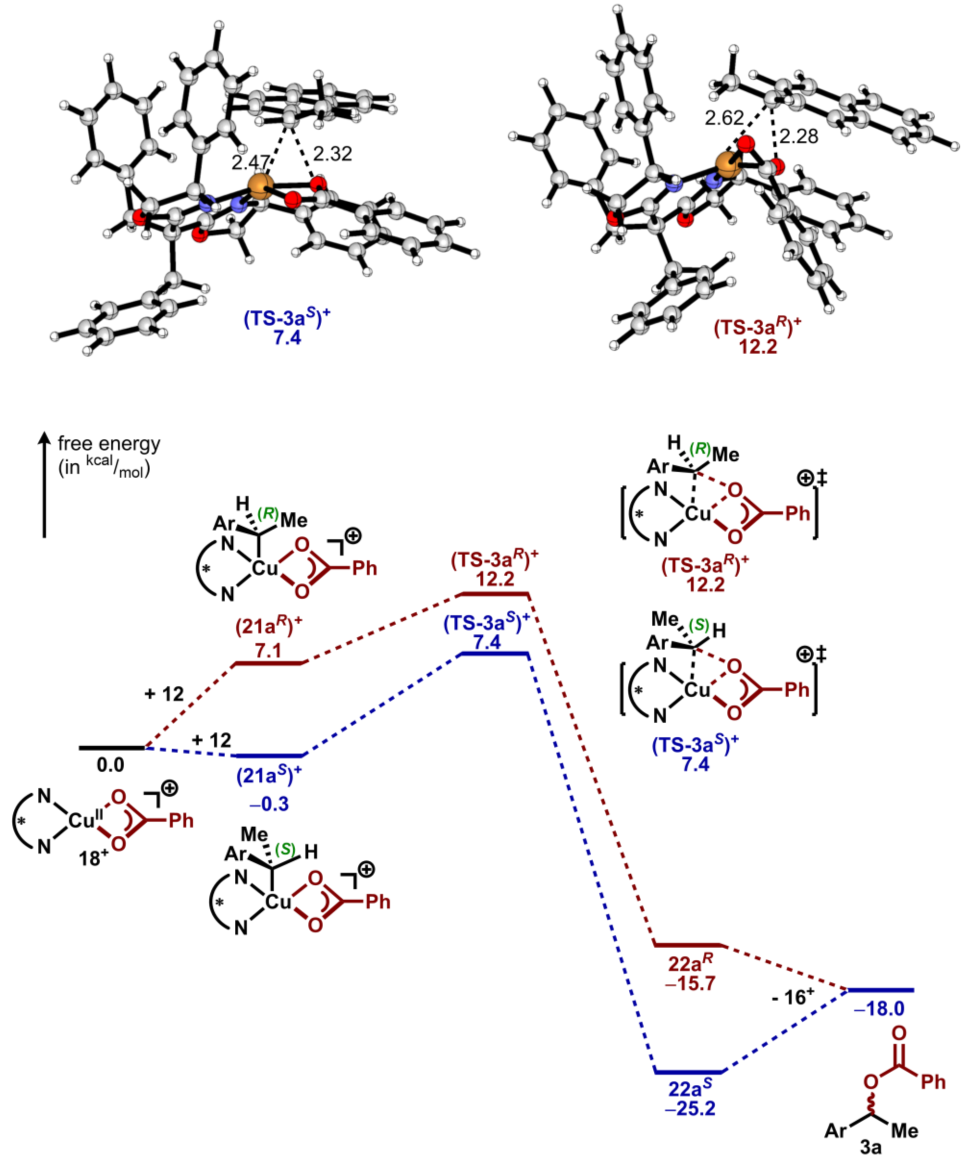


**Scheme S7**. Computed potential energy profiles (of both pro-*R* and pro-*S* pathways) for inner-sphere addition of **12** via Cu(III) intermediates $\mathbf{21}^{+}$ in conformation a (Gibbs free energies in kcal/mol). Geometries of key stationary points are shown with selected distances in Å.

The barrierless inner-sphere addition of alkyl radical **12** and $\mathbf{18}^{+}$ results in formal Cu(III) intermediates $(\mathbf{21a}^{R})^{+}$ (ΔG = 7.1 kcal/mol) and $(\mathbf{21a}^{S})^{+}$ (ΔG = -0.3 kcal/mol). Subsequent C–O bond formation proceeds via reductive elimination TSs $(\mathbf{TS\text{-}3a}^{R})^{+}$ ($\Delta G^{\ddagger,R}$ = 12.2 kcal/mol) and $(\mathbf{TS\text{-}3a}^{S})^{+}$ ($\Delta G^{\ddagger,S}$ = 7.4 kcal/mol) with retention of configuration at the benzylic positions. This process is highly exergonic and occurs irreversibly, making this step enantiodetermining. Formal

Cu(I) intermediates **22a*R*** (ΔG = -15.7 kcal/mol) and **22a*S*** (ΔG = -25.2 kcal/mol) are then formed, prior to product release via simple decoordination of **3a**.

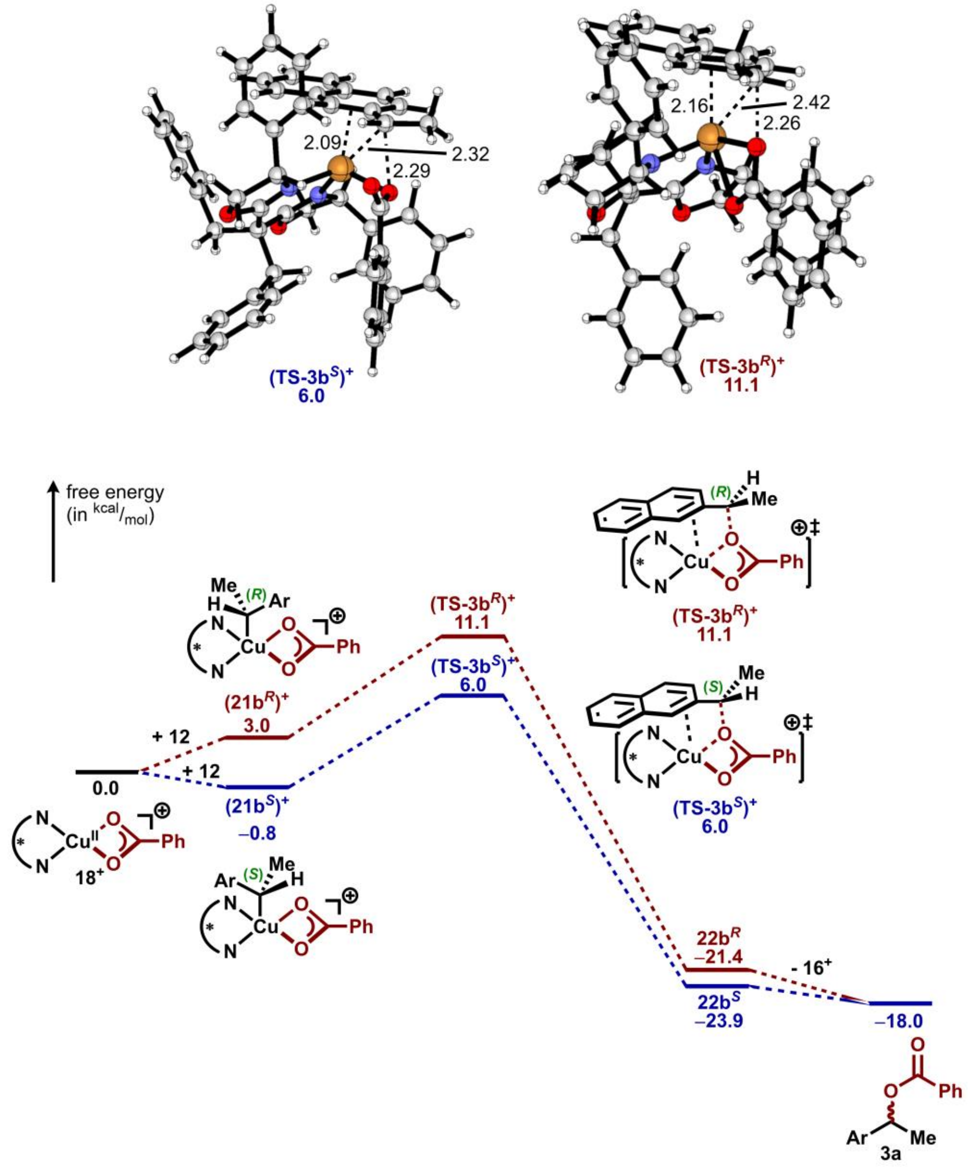


**Scheme S8**. Computed potential energy profiles (of both pro-*R* and pro-*S* pathways) for inner-sphere addition of **12** via Cu(III) intermediates **21**$^+$ in conformation b (Gibbs free energies in kcal/mol). Geometries of key stationary points are shown with selected distances in Å.

The barrierless inner-sphere addition of alkyl radical **12** and **18**$^+$ results in formal Cu(III) intermediates **(21b*R*)**$^+$ (ΔG = 3.0 kcal/mol) and **(21b*S*)**$^+$ (ΔG = -0.8 kcal/mol). Subsequent C–O bond formation proceeds via reductive elimination TSs **(TS-3b*R*)**$^+$ ($\Delta G^{\ddagger,R}$ = 11.1 kcal/mol) and **(TS-3b*S*)**$^+$ ($\Delta G^{\ddagger,S}$ = 6.0 kcal/mol) with retention of configuration at the benzylic positions. Formal

Cu(I) intermediates **22b*R*** (ΔG = -21.4 kcal/mol) and **22b*S*** (ΔG = -23.9 kcal/mol) are then formed, prior to product release via simple decoordination of **3a**.

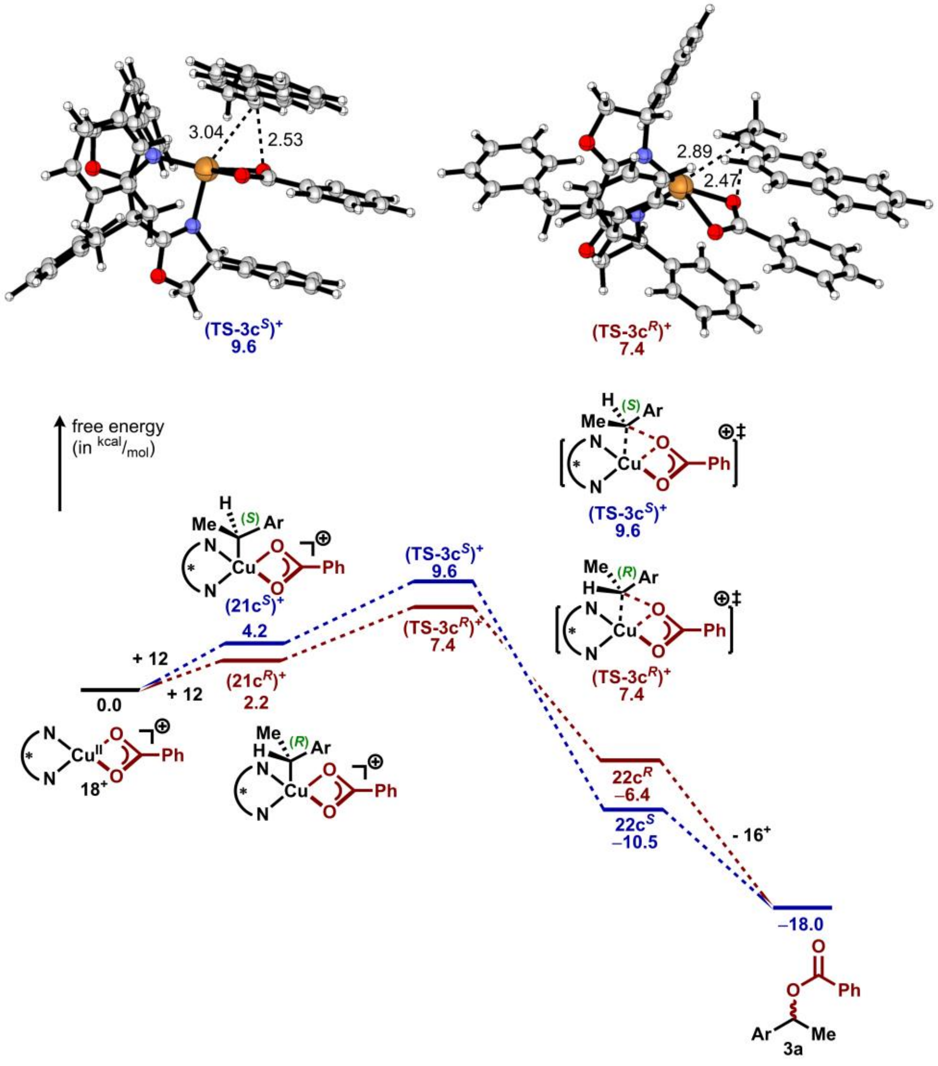


**Scheme S9**. Computed potential energy profiles (of both pro-*R* and pro-*S* pathways) for inner-sphere addition of **12** via Cu(III) intermediates **21**$^+$ in conformation c (Gibbs free energies in kcal/mol). Geometries of key stationary points are shown with selected distances in Å.

The barrierless inner-sphere addition of alkyl radical **12** and **18**$^+$ results in formal Cu(III) intermediates **(21c*R*)**$^+$ (ΔG = 2.2 kcal/mol) and **(21c*S*)**$^+$ (ΔG = 4.2 kcal/mol). Subsequent C–O bond formation proceeds via reductive elimination TSs **(TS-3c*R*)**$^+$ ($\Delta G^{\ddagger,R}$ = 7.4 kcal/mol) and **(TS-3c*S*)**$^+$ ($\Delta G^{\ddagger,S}$ = 9.6 kcal/mol) with retention of configuration at the benzylic positions. Formal Cu(I) intermediates **22c*R*** (ΔG = -6.4 kcal/mol) and **22c*S*** (ΔG = -10.5 kcal/mol) are then formed, prior to

product release via simple decoordination of **3a**. For this conformation only, an inversion of the enantioselectivity is observed, where the *pro*-R pathway is kinetically favored by 2.2 kcal/mol.

### 9.6.2 C–O Bond Formation via Radical-Polar Crossover

The activation barrier associated with SET cannot be assessed using the transition state theory (TST), since there is no well-defined structured associated with bond breaking and/or forming or geometric rearrangements. In Marcus theory, the free energy barrier ($\Delta G^{\ddagger}$) corresponding to SET is dependent on $\Delta G^{0}$ (thermodynamics for the electron transfer) and $\lambda$ (reorganisation energy or vertical energy): $\Delta G^{\ddagger} = \frac{\lambda}{4}\left(1 + \frac{\Delta G^{0}}{\lambda}\right)^{2}$.[53, 54]

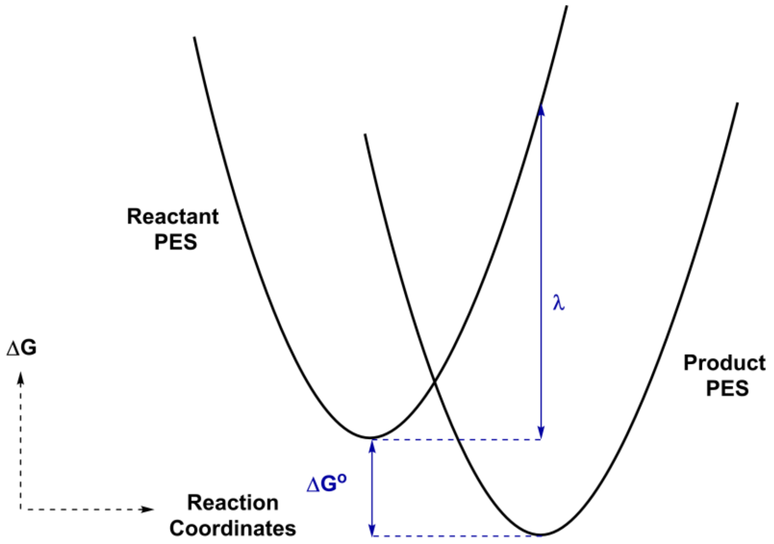


**Scheme S10**. Potential energy surface for a general single electron transfer reaction, with key parameters of Marcus theory.

$\lambda$ can be computed by applying a modified version of the Nelsen-4 points method, as detailed below.[55] First, the reorganization energies associated with the forward ($\lambda^F$) and the backward ($\lambda^B$) reactions are determined. To do so, the reorganization energies of half reactions are computed ($\lambda^{F}{}_{1}$, $\lambda^{F}{}_{2}$, $\lambda^{B}{}_{1}$ and $\lambda^{B}{}_{2}$). For example, $\lambda_1{}^F$ is the difference between the SCF energies (after single-point energy corrections) of **²18** and **18⁺**. **18⁺** corresponds to a minimum (in its optimised geometry and own electronic configuration,) and **²18** corresponds to the optimised geometry of the Cu(I) **18** and electronic configuration of the starting material **18⁺**. During this process, the solvation shell is conserved (using the *NonEquilibrium* keyword). Finally, the overall $\lambda$ is taken as the square root of the product of forward and backward reactions.

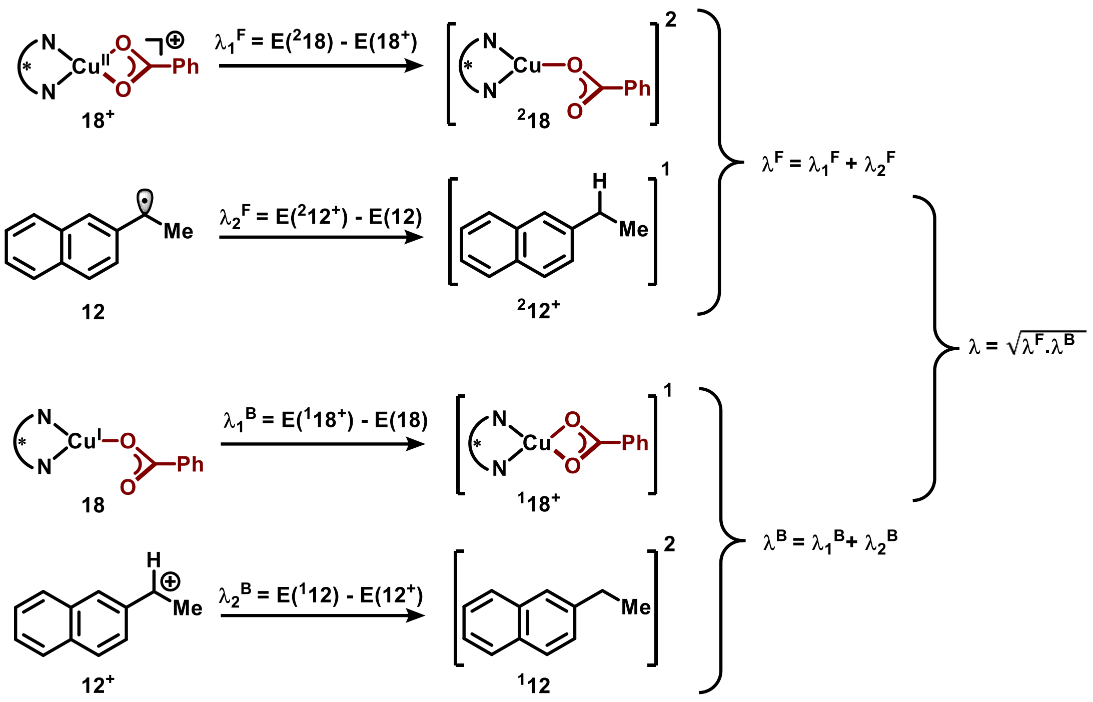


**Scheme S11**. Determination of the reorganization energy associated with SET.

| | |
|---|---|
| $\lambda_1^F$ | **24.9** |
| $\lambda_2^F$ | 12.4 |
| $\lambda^F$ | 37.3 |
| $\lambda_1^B$ | 23.0 |
| $\lambda_2^B$ | 12.9 |
| $\lambda^B$ | 35.9 |
| $\lambda$ | 36.6 |
| $\Delta G^0$ | 7.9 |
| $\Delta G^\ddagger$ | **13.6** |

**Table S6**. Computed values of the various components of the final reorganization energy, thermodynamics of the reaction and resulting activation barrier for SET (SCF and Gibbs free energies in kcal/mol).

### 9.6.3 NCI Plots for All Located Conformers of (TS-3$^R$)$^+$ and (TS-3$^S$)$^+$

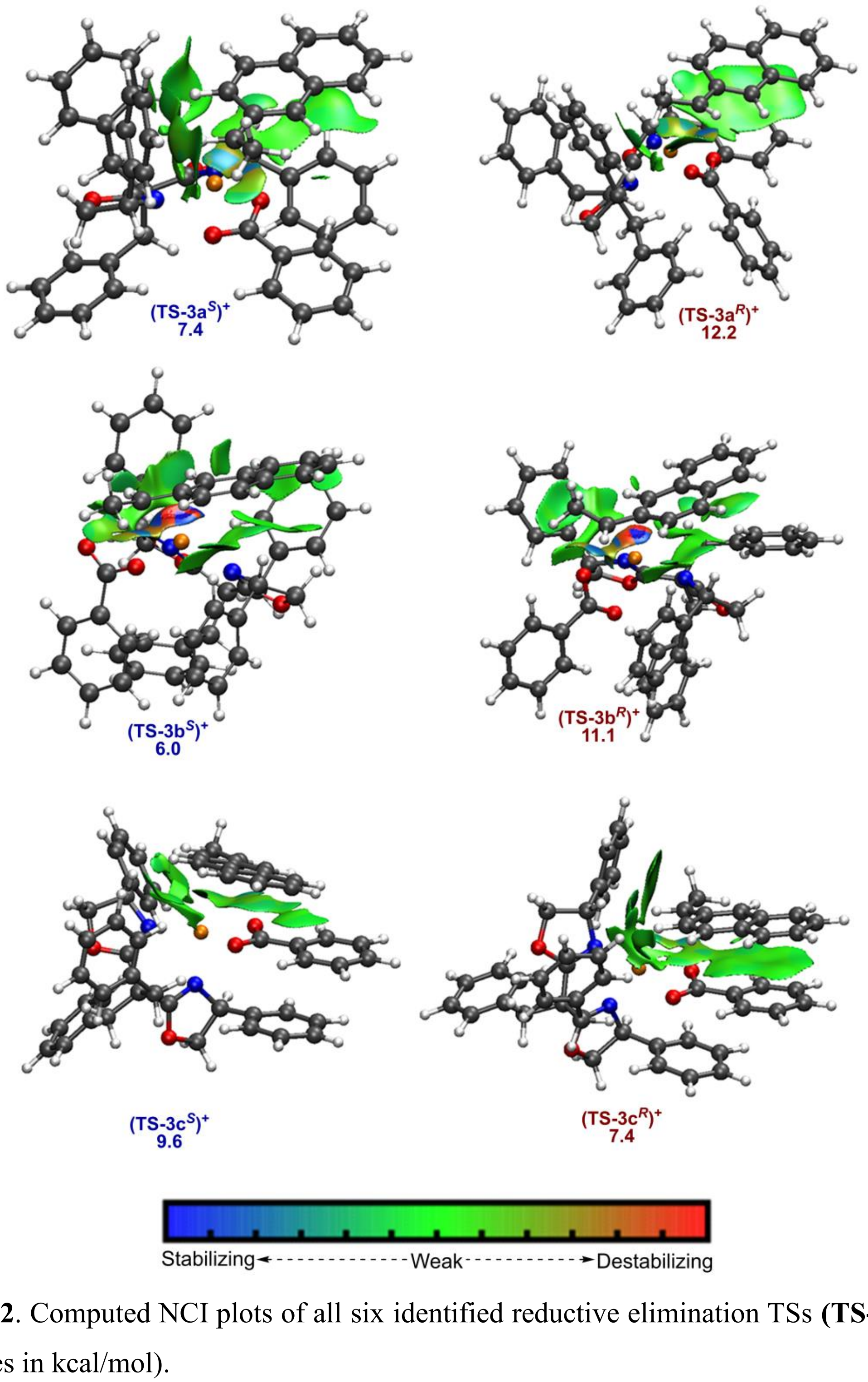


**Scheme S12**. Computed NCI plots of all six identified reductive elimination TSs **(TS-3)**$^+$ (Gibbs free energies in kcal/mol).

### 9.6.4 Temperature Dependence of Enantioselectivity

The activation barriers associated with inner-sphere addition (via reductive elimination TSs **(TS-3$^{R}$)$^{+}$** and **(TS-3$^{R}$)$^{+}$**) and radical-polar cross-over (via SET) were recomputed for T = –20, 25 and 60 °C using GoodVibes. The corresponding rate constants ***k*** were then estimated using the Eyring equation: $k = \frac{k_B T}{h} e^{-\Delta G^{\ddagger}/RT}$, where $k_B$ = 1.381.10$^{-23}$ J/K (Boltzmann constant), $h$ = 6.626.10$^{-23}$ J/s (Plank constant) and $R$ = 8.314 J/mol/K (gas constant).

**Table S7**. Computed temperature dependency of enantioselectivity.

| T | | (TS-3$^{S}$)$^{+}$ | SET | (TS-3$^{S}$)$^{+}$/SET |
|---|---|---|---|---|
| –20 °C | ΔG‡ (kcal/mol) | 3.7 | 13.5 | – |
| | k | 3.4×10$^{-2}$ | 1.2×10$^{-10}$ | 2.8×10$^{8}$ |
| 25 °C | ΔG‡ (kcal/mol) | 6.0 | 13.6 | – |
| | k | 2.5×10$^{-3}$ | 6.6×10$^{-9}$ | 3.8×10$^{5}$ |
| 60 °C | ΔG‡ (kcal/mol) | 7.8 | 13.6 | – |
| | k | 5.3×10$^{-4}$ | 2.8×10$^{-8}$ | 1.9×10$^{4}$ |

Because this analysis employs different computational algorithms for inner-sphere vs SET mechanisms, it should be viewed as providing only an approximate estimate of how temperature influences the competition between the two C(sp$^{3}$)–O bond-forming pathways. At 60 °C, the calculated partition ratio (**k[(TS-3$^{S}$)$^{+}$]/k[SET]**) of 1.9×10$^{4}$ would imply essentially no contribution from a radical-polar crossover pathway, which is likely an overestimation. Nonetheless, the model predicts a much stronger preference for the inner-sphere pathway at –20 °C (2.8×10$^{8}$), consistent with our experimental observation of improved enantioselectivity under low-temperature photochemical conditions.

### 9.6.5 Computational Modelling of Coupling with Acetic Acid

To probe the role of alkyl radical–carboxylate π–π stacking observed in **(TS-3$^{R}$)$^{+}$**, we computed the potential energy surface for the formation of **3t** (coupling between **1a** and acetic acid).

Comparing the energy profile for **3t** with that for **3a** revealed the following findings:

1) The thermodynamic driving force for the C–O coupling step are comparable (**3a**: −18.0 kcal/mol vs **3t**: −17.7 kcal/mol).

2) The lowest-energy TS leading to the favored *S*-product, **(TS-3$^{S}$)$^{+}$$_{3t}$** (ΔG$^{‡}$ = 6.5 kcal/mol), has a barrier comparable to that of **(TS-3$^{S}$)$_{3a}$$^{+}$** (ΔG$^{‡}$ = 6.0 kcal/mol). Similar π–Cu interactions (2.09 Å) were observed in both TSs.

3) The lowest-energy TS leading to the disfavored *R*-product, **(TS-3$^{R}$)$^{+}$$_{3t}$** (ΔG$^{‡}$ = 11.3 kcal/mol), corresponds to a higher barrier compared to that of **(TS-3$^{R}$)$_{3a}$$^{+}$** (ΔG$^{‡}$ = 7.4 kcal/mol). This increase in barrier is likely due to the absence of alkyl radical–carboxylate π–π stacking.

Overall, these observations suggest that the alkyl radical–carboxylate π–π stacking represents one of the main stabilizing interactions in **(TS-3$^{R}$)$_{3a}$$^{+}$**. However, this stabilization is still insufficient to make formation of the *R*-product the dominant pathway, which is consistent with the experimentally observed selectivity for the *S*-product.

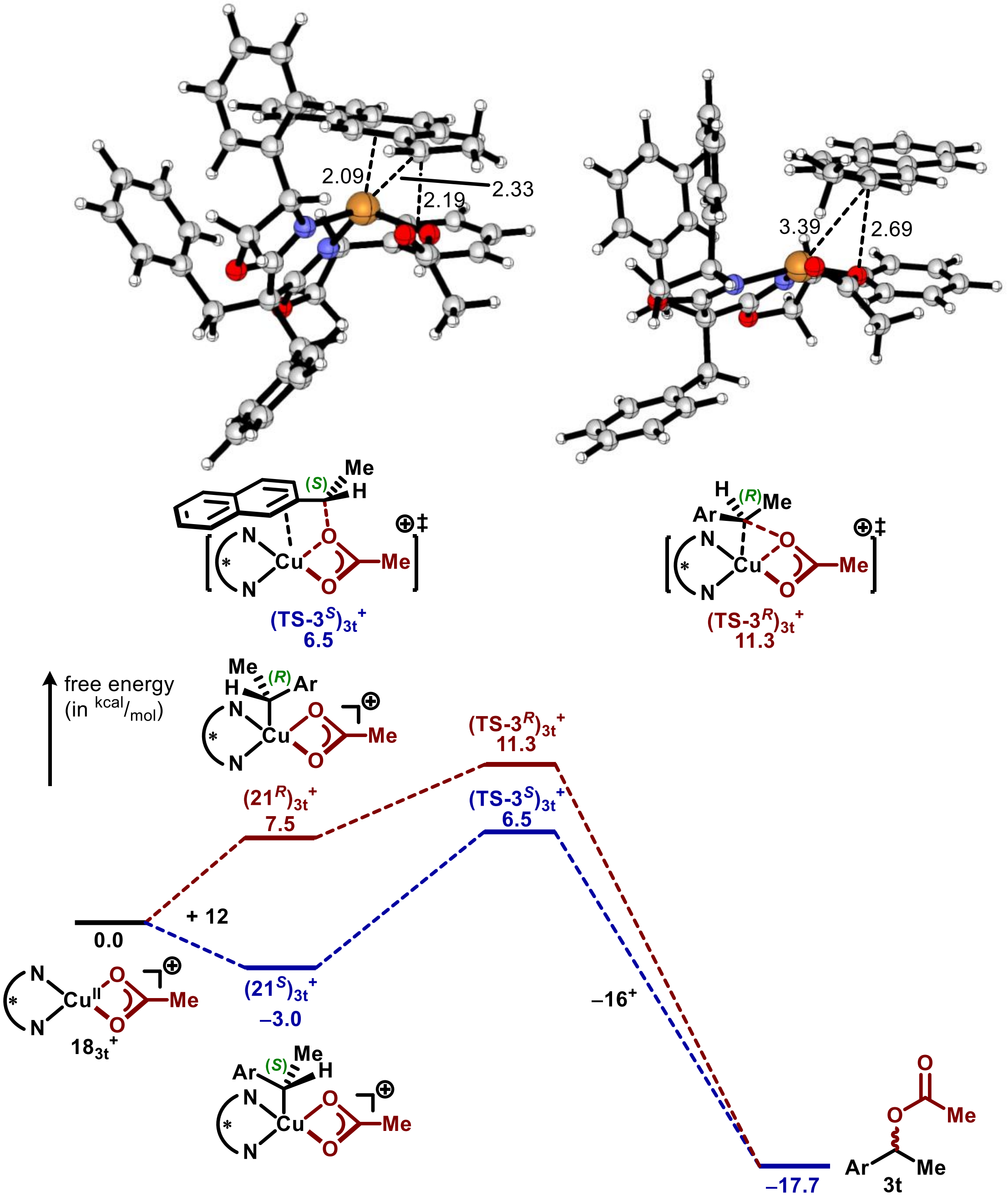


**Scheme S13**. Computed potential energy profiles (of both *pro*-R and *pro*-S pathways) for inner-sphere addition of **12** and leading to the formation of **3t** (Gibbs free energies in kcal/mol). Geometries of key stationary points are shown with selected distances in Å.

## 9.7 Benchmark Study

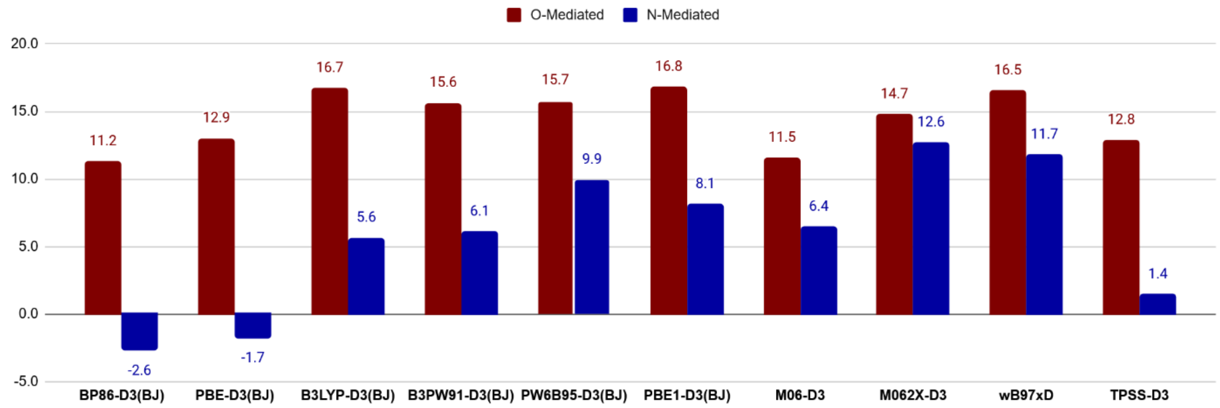


**Scheme S14**. Computed activation barriers for the lowest energy pathway of O- and N-mediated C–B cleavage mechanisms (Gibbs free energies in kcal/mol).

Functional testing was performed on **TS-1** and **TS-1’** at the XC/Def2-TZVP/SMD(PhCl) //PW6B95-D3(BJ)/def2-SVP level of theory (with XC = functional + dispersion). A set of 10 functionals was selected among which different classes of functionals are represented (pure GGAs, *meta*-GGAs, hybrids, *meta*-hybrids and heavily parametrized). Despite varying quantitative results, all 10 functionals provided the same qualitative conclusion: an N-mediated C–B cleavage is favored to an O-mediated pathway.

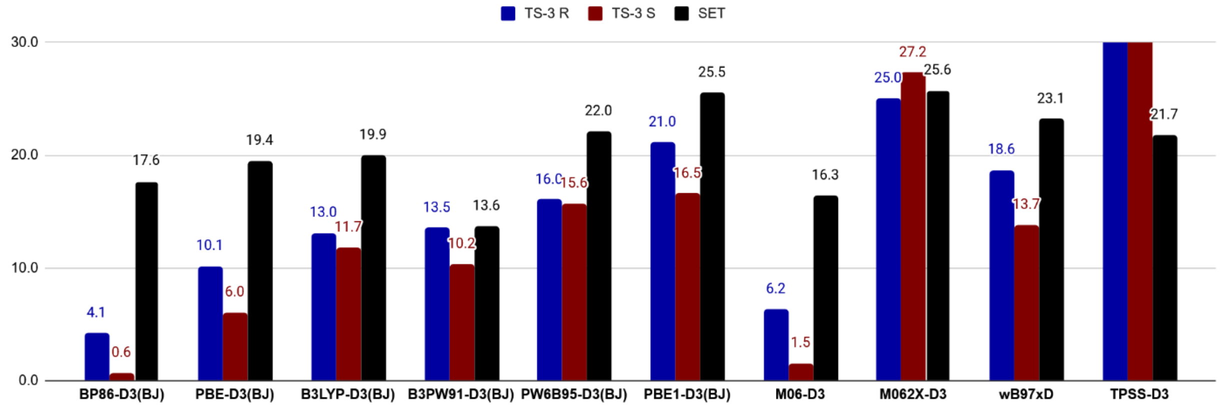


**Scheme S15**. Computed activation barriers for the lowest energy pathway for Cu-mediated C–O bond formation via **(TS-3c$^{R}$)$^{+}$**, **(TS-3b$^{S}$)$^{+}$**, or SET (Gibbs free energies in kcal/mol).

Functional testing was performed on **(TS-3c*R*)**$^+$, **(TS-3b*S*)**$^+$ and SET at the XC/Def2-TZVP/SMD(PhCl)//PW6B95-D3(BJ)/def2-SVP level of theory (with XC = functional + dispersion). A set of 10 functionals was selected among which different classes of functionals are represented (pure GGAs, *meta*-GGAs, hybrids, *meta*-hybrids and heavily parametrized). Despite varying quantitative results, the majority of functionals provided the same qualitative conclusion: an inner-sphere mechanism is preferred to SET, favoring the *pro*-S pathway.

M062X and TPSS present divergent outputs where the *pro*-R and SET pathways are favored respectively, in contradiction with the experimental observations. Additionally, note the excessively elevated free energies for **(TS-3c*R*)**$^+$ and **(TS-3b*S*)**$^+$ with TPSS (70.9 and 65.0 kcal/mol respectively).

## 9.8 N–H vs. C–H Activation

The N-radical-mediated C–B activation pathway is favored over C–H bond activation in the alkylborane substrates. This preference can be rationalized by the selective activation of the N–H bond in the radical precursor rather than the substrate C–H bonds. The following studies were conducted to further elucidate the origin of this selectivity.

Using substrate **1a** as a model system, we computed the C–H abstraction barrier from the benzylic position (**TS-6**) or the methyl group (**TS-7**) and compared the results against our N–H bond activation pathway. The results are summarized as following:

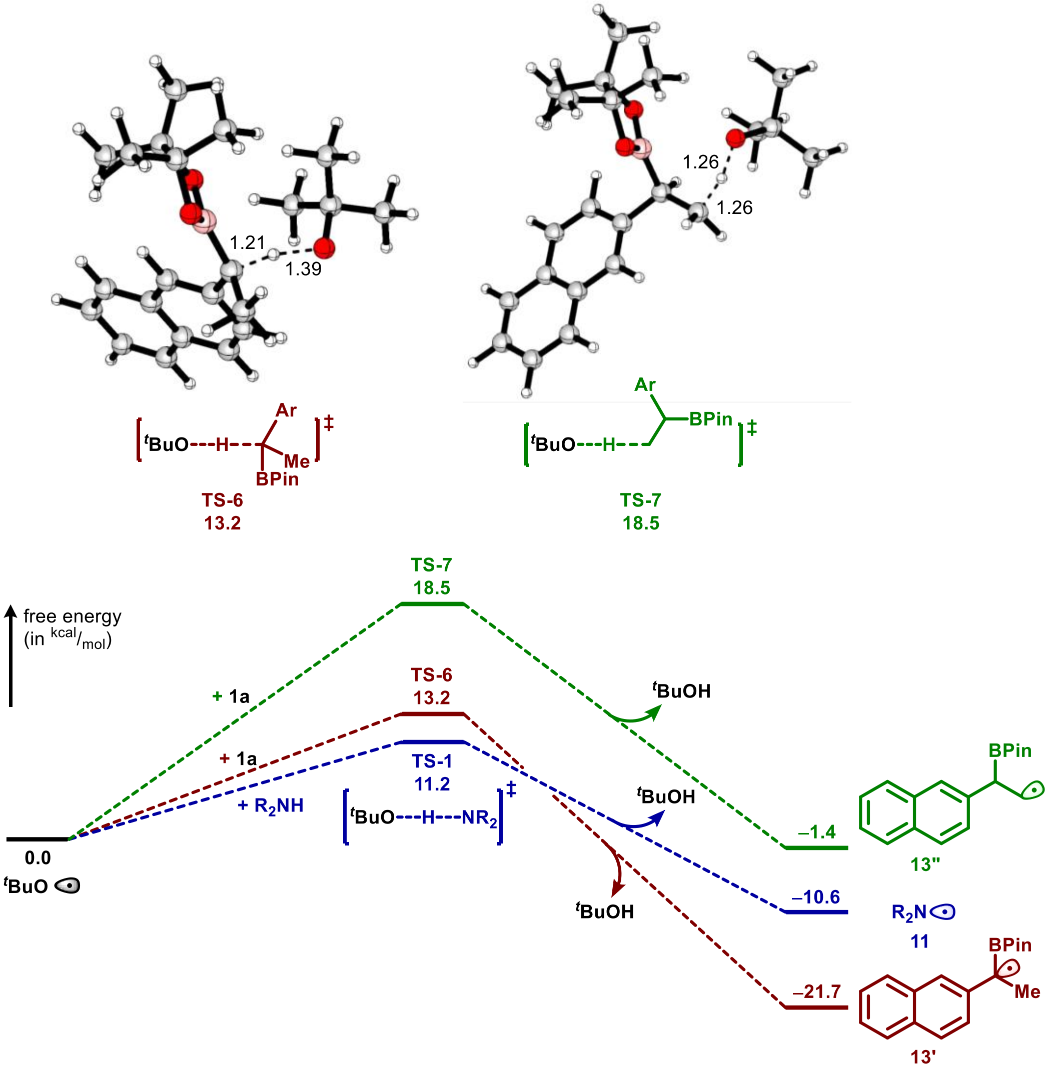


**Scheme S16.** Computed potential energy surfaces for *tert*-butoxy radical-mediated hydrogen atom transfer from **1a** (Gibbs free energies in kcal/mol) in comparison to N–H activation. Geometries of key stationary points are shown with selected distances in Å.

These calculations show that N–H activation is kinetically favored over either benzylic or methyl C–H abstraction, even though benzylic HAT is thermodynamically more favorable. We believe this kinetic–thermodynamic mismatch is mechanistically informative. In particular, the preference for N–H abstraction does not appear to arise solely from bond strengths (carbazole N–H BDE = ca. 90 kcal/mol vs benzylic C–H BDE = ca. 88 kcal/mol) or polarity matching (the benzylic C–H bond is likely more hydridic than the N–H bond). Instead, the higher barrier for benzylic HAT is consistent with Bernasconi's principle of non-perfect synchronization, wherein the resonance stabilization available to a benzylic radical develops relatively late along the HAT reaction coordinate and therefore is not fully manifested in the transition state.[56, 57] As a result, the benzylic C–H pathway benefits strongly in product stability but less so in transition-state stabilization. This interpretation is consistent with prior studies of oxygen-centered radical HAT by Bietti et. al.,[57] which found that benzylic and allylic substrates often exhibit weaker-than-expected kinetic activation relative to their bond strengths because resonance stabilization takes place late along the reaction coordinate.

The spin-density plots (Scheme S17) of the three transition states (**TS-1**, **TS-6**, and **TS-7**) support this interpretation. In **TS-1**, the carbazole π-system displays substantial spin delocalization, indicative of a more favorable HAT mechanism with partial charge-transfer character that is better accommodated by the electron-rich carbazole π-system. By contrast, in **TS-6** and **TS-7**, the arene π-system remains comparatively redox innocent, indicating that the resonance stabilization of the benzylic radical is only weakly developed at the transition state.

Comparing charge distribution in **TS-1** and **TS-6** (Scheme S18) suggests that the transferring H atom in the N–H pathway bears greater positive character, which could contribute to a lower barrier for this pathway. We therefore view the preference for N–H activation as arising from a combination of factors: delayed development of benzylic radical resonance stabilization in the C–H pathways versus a more electronically accommodating and possibly more charge-transfer-like HAT process for carbazole N–H activation.

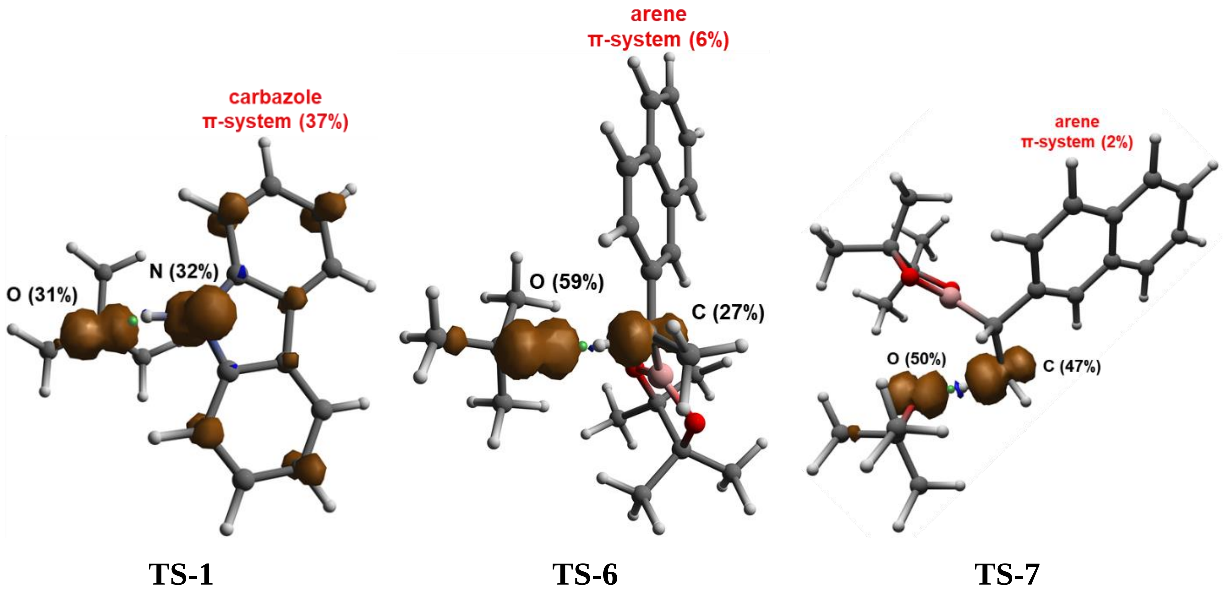


**Scheme S17**. Spin density plot of tert-butoxy radical-mediated HAT transition states.

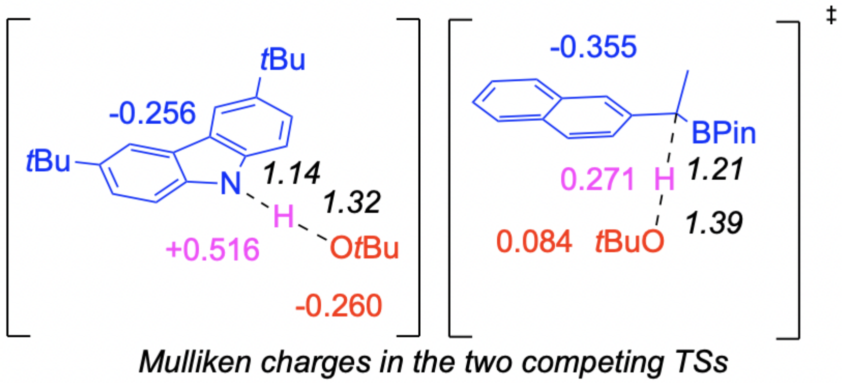


**Scheme S18**. Charge distribution analysis of **TS-1** and **TS-6**.

Following the generation of N-centered radical, we computed the barriers for C–H abstraction at the benzylic position (**TS-8**) and the methyl group (**TS-9**) in substrate **1a** and compared them to the barrier for the N-centered-radical-mediated C–B bond activation pathway. The results are summarized as the following:

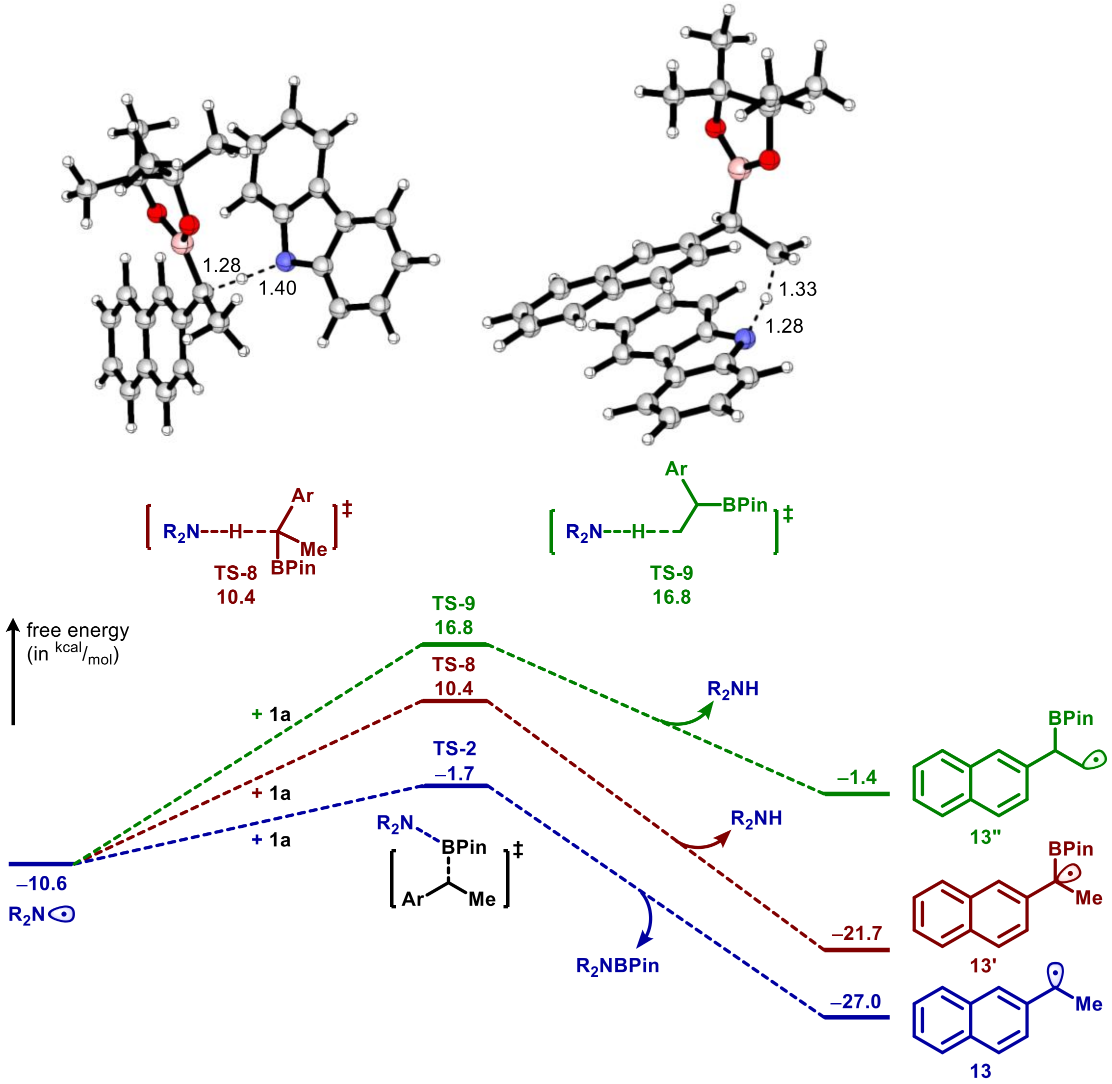


**Scheme S19**. Comparing N-radical-mediated C–B vs C–H activation pathways.

The C–B bond cleavage is strongly favored over benzylic or methyl C–H activation. We propose that the significantly lower barrier for C–B bond activation likely accounts for our experimental observed lack of reactivity from the benzylic C–H bonds. We attribute this selectivity to the weaker C–B bond (BDE = *ca*. 77 kcal/mol; Chem. Sci. **2023**, *14*, 4278) compared with a typical benzylic C–H bond (BDE = *ca*. 88 kcal/mol). The C–B cleavage pathway is also favored thermodynamically (ΔG = –27.0 kcal/mol) compared to the benzylic (ΔG = –21.7 kcal/mol) or methyl CH activation (ΔG = –1.4 kcal/mol).

## 9.9 Origin of Enantioselectivity

To gain a better understanding of the origin of the reaction enantioselectivity, we have conducted a direct structural comparison of the lowest-energy conformers viewed from the same orientation attached below. This comparison shows that the energetic preference for **(TS-3$^{S}$)$^{+}$** arises from a more favorable overall fit within the $C_2$-symmetric ligand pocket. In **(TS-3$^{S}$)$^{+}$**, the two reacting fragments occupy the two open quadrants of the chiral pocket, allowing C–O bond formation with minimal reorganization of the ligand framework while preserving a favorable π–Cu interaction. In contrast, in the lowest-energy *R*-selective conformer, both benzyl and benzoate fragments are forced into the same quadrant of the pocket. Accommodating this arrangement requires substantially greater ligand reorganization. We therefore conclude that the lower energy of **(TS-3$_{S}$)$^{+}$** reflects better geometric complementarity to the chiral Cu complex, whereas the minor pathway suffers from a distortion penalty.

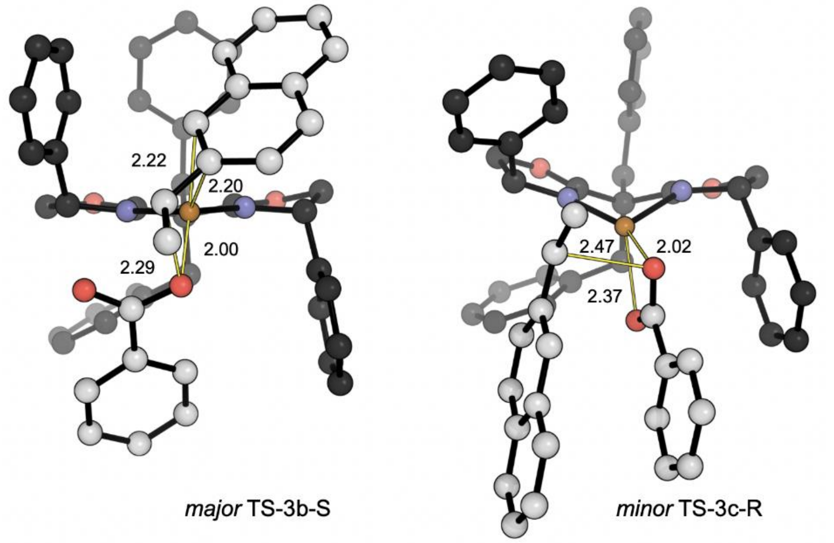


**Scheme S20.** Comparison of diastereomeric reductive elimination transition structures leading to *S* and *R* enantiomers.

## 9.10 Optimized Cartesian Coordinates

**3**

| | | | |
|---|---|---|---|
| H | 3.25286 | -0.90447 | -0.00003 |
| O | 2.30201 | -1.07948 | 0.00005 |
| O | 2.32020 | 1.14930 | -0.00028 |
| C | 1.69973 | 0.11830 | -0.00006 |
| C | 0.21945 | 0.02584 | -0.00000 |
| C | -0.50664 | 1.21752 | -0.00017 |
| C | -0.44740 | -1.20057 | 0.00022 |
| C | -1.89358 | 1.18374 | -0.00012 |
| C | -1.83606 | -1.22979 | 0.00028 |
| C | -2.55820 | -0.04004 | 0.00011 |
| H | 0.04189 | 2.15673 | -0.00034 |
| H | 0.12922 | -2.12171 | 0.00036 |
| H | -2.46090 | 2.11221 | -0.00025 |
| H | -2.35817 | -2.18447 | 0.00045 |
| H | -3.64641 | -0.06684 | 0.00015 |

**3-**

| | | | |
|---|---|---|---|
| O | -2.35129 | 1.12818 | 0.00018 |
| O | -2.35128 | -1.12818 | -0.00010 |
| C | -1.82849 | -0.00000 | -0.00007 |
| C | -0.27804 | 0.00000 | -0.00004 |
| C | 0.43233 | -1.19965 | 0.00003 |
| C | 0.43233 | 1.19966 | -0.00007 |
| C | 1.82383 | -1.20438 | 0.00010 |
| C | 1.82383 | 1.20438 | -0.00008 |
| C | 2.52549 | -0.00000 | 0.00003 |
| H | -0.16190 | -2.11259 | 0.00010 |
| H | -0.16190 | 2.11259 | -0.00018 |
| H | 2.37035 | -2.14903 | 0.00002 |
| H | 2.37035 | 2.14903 | 0.00002 |
| H | 3.61610 | -0.00000 | 0.00005 |

**22**

| | | | |
|---|---|---|---|
| O | 2.42153 | -1.03471 | 0.00066 |
| O | 2.42152 | 1.03472 | -0.00066 |
| C | 1.71611 | -0.00001 | 0.00000 |
| C | 0.24673 | -0.00001 | 0.00000 |
| C | -0.44248 | 1.21244 | 0.00024 |
| C | -0.44248 | -1.21244 | -0.00024 |
| C | -1.83020 | 1.20952 | 0.00025 |
| C | -1.83021 | -1.20951 | -0.00025 |
| C | -2.52086 | 0.00000 | -0.00000 |
| H | 0.11976 | 2.14456 | 0.00044 |
| H | 0.11975 | -2.14457 | -0.00043 |
| H | -2.37720 | 2.14980 | 0.00046 |
| H | -2.37721 | -2.14979 | -0.00046 |
| H | -3.60928 | 0.00001 | -0.00000 |

**$3^+$**

| | | | |
|---|---|---|---|
| H | 3.20118 | 1.09392 | 0.00020 |
| O | 2.23313 | 1.12979 | 0.00026 |
| O | 2.23313 | -1.12979 | -0.00026 |
| C | 1.60837 | -0.00000 | 0.00000 |
| C | 0.18307 | -0.00000 | 0.00000 |
| C | -0.51383 | -1.22548 | 0.00010 |
| C | -0.51383 | 1.22548 | -0.00010 |
| C | -1.89467 | -1.21590 | 0.00013 |
| C | -1.89467 | 1.21590 | -0.00013 |
| C | -2.58104 | 0.00000 | -0.00000 |
| H | 0.03313 | -2.16477 | 0.00017 |
| H | 0.03313 | 2.16477 | -0.00017 |
| H | -2.44479 | -2.15343 | 0.00026 |
| H | -2.44479 | 2.15343 | -0.00026 |
| H | -3.66950 | 0.00000 | -0.00000 |
| H | 3.20118 | -1.09392 | -0.00022 |

**$R_2NH$**

| | | | |
|---|---|---|---|
| C | 2.47000 | 1.21730 | -0.00010 |
| C | 3.41339 | 0.20033 | -0.00002 |
| C | 1.12506 | 0.85282 | -0.00007 |
| C | 3.03295 | -1.14842 | 0.00010 |
| C | 0.72227 | -0.50358 | 0.00007 |
| C | 1.69313 | -1.50631 | 0.00015 |
| C | -1.12506 | 0.85282 | 0.00005 |
| C | -0.72227 | -0.50358 | -0.00007 |
| H | 2.77130 | 2.26285 | -0.00016 |
| H | 4.47111 | 0.45680 | -0.00004 |
| H | 3.79942 | -1.92035 | 0.00012 |
| H | 1.40040 | -2.55474 | 0.00019 |
| C | -2.47000 | 1.21730 | 0.00010 |

| | | | |
|---|---|---|---|
| C | -1.69313 | -1.50631 | -0.00016 |
| C | -3.03295 | -1.14842 | -0.00008 |
| C | -3.41339 | 0.20033 | 0.00004 |
| H | -2.77130 | 2.26285 | 0.00016 |
| H | -1.40039 | -2.55474 | -0.00019 |
| H | -3.79942 | -1.92035 | -0.00009 |
| H | -4.47111 | 0.45680 | 0.00009 |
| N | 0.00000 | 1.64491 | -0.00004 |
| H | 0.00000 | 2.65083 | 0.00017 |

**$R2N^-$**

| | | | |
|---|---|---|---|
| C | 2.43688 | 1.24197 | 0.00010 |
| C | 3.39651 | 0.24478 | 0.00002 |
| C | 1.06999 | 0.89313 | 0.00008 |
| C | 3.04257 | -1.11929 | -0.00012 |
| C | 0.71569 | -0.50435 | -0.00008 |
| C | 1.70466 | -1.49067 | -0.00018 |
| C | -1.06998 | 0.89314 | -0.00005 |
| C | -0.71569 | -0.50435 | 0.00005 |
| H | 2.71691 | 2.29515 | 0.00016 |
| H | 4.45342 | 0.51780 | 0.00004 |
| H | 3.82320 | -1.88045 | -0.00016 |
| H | 1.42851 | -2.54713 | -0.00024 |
| C | -2.43689 | 1.24197 | -0.00005 |
| C | -1.70466 | -1.49067 | 0.00013 |
| C | -3.04257 | -1.11929 | 0.00010 |
| C | -3.39651 | 0.24477 | 0.00002 |
| H | -2.71692 | 2.29515 | -0.00010 |
| H | -1.42850 | -2.54713 | 0.00016 |
| H | -3.82320 | -1.88045 | 0.00013 |
| H | -4.45341 | 0.51780 | 0.00001 |
| N | -0.00000 | 1.72034 | -0.00002 |

**10**

| | | | |
|---|---|---|---|
| C | 2.42970 | 1.25203 | -0.00003 |
| C | 3.40340 | 0.25718 | -0.00002 |
| C | 1.08664 | 0.86492 | -0.00002 |
| C | 3.04819 | -1.09236 | -0.00001 |
| C | 0.72893 | -0.51617 | 0.00002 |
| C | 1.70355 | -1.49256 | 0.00002 |
| C | -1.08664 | 0.86492 | 0.00002 |
| C | -0.72892 | -0.51618 | -0.00004 |
| H | 2.68931 | 2.30784 | -0.00003 |
| H | 4.45601 | 0.53236 | -0.00003 |
| H | 3.82947 | -1.85004 | -0.00002 |
| H | 1.44660 | -2.55037 | 0.00000 |
| C | -2.42971 | 1.25203 | 0.00005 |
| C | -1.70355 | -1.49257 | -0.00004 |
| C | -3.04817 | -1.09237 | -0.00000 |
| C | -3.40340 | 0.25718 | 0.00003 |
| H | -2.68929 | 2.30784 | 0.00007 |
| H | -1.44659 | -2.55038 | -0.00005 |
| H | -3.82947 | -1.85003 | 0.00000 |
| H | -4.45602 | 0.53233 | 0.00005 |
| N | -0.00001 | 1.69203 | 0.00001 |

**$^t$BuOH**

| | | | |
|---|---|---|---|
| O | 0.03658 | 0.00006 | 1.43687 |
| C | -0.00668 | 0.00000 | 0.01795 |
| C | 0.67755 | 1.25464 | -0.51431 |
| H | 0.62613 | 1.31137 | -1.60832 |
| H | 0.20285 | 2.14867 | -0.09449 |
| H | 1.73885 | 1.26606 | -0.22992 |
| C | 0.67698 | -1.25500 | -0.51419 |
| H | 0.62560 | -1.31178 | -1.60820 |
| H | 1.73826 | -1.26691 | -0.22973 |
| H | 0.20183 | -2.14878 | -0.09434 |
| C | -1.48390 | 0.00032 | -0.33619 |
| H | -1.97002 | 0.88724 | 0.08523 |
| H | -1.63008 | 0.00020 | -1.42216 |
| H | -1.97043 | -0.88628 | 0.08547 |
| H | 0.96068 | 0.00000 | 1.70196 |

**$^t$BuO$^-$**

| | | | |
|---|---|---|---|
| O | 0.00016 | -0.00019 | 1.47045 |
| C | -0.00001 | -0.00002 | 0.16038 |
| C | 0.90347 | -1.13373 | -0.43514 |
| H | 0.94524 | -1.18609 | -1.53877 |
| H | 1.92289 | -0.99396 | -0.04705 |
| H | 0.53969 | -2.09624 | -0.04705 |
| C | -1.43373 | -0.21548 | -0.43487 |
| H | -1.50014 | -0.22543 | -1.53849 |
| H | -1.82245 | -1.16818 | -0.04675 |
| H | -2.08522 | 0.58089 | -0.04667 |
| C | 0.53013 | 1.34938 | -0.43486 |
| H | 1.54553 | 1.51557 | -0.04664 |
| H | 0.55474 | 1.41186 | -1.53849 |
| H | -0.10071 | 2.16227 | -0.04679 |

**$^{t}$BuO_rad**

| | | | |
|---|---|---|---|
| O | -0.00001 | 0.27480 | 1.41560 |
| C | -0.00000 | -0.02629 | 0.08415 |
| C | 1.26736 | -0.78910 | -0.30721 |
| H | 1.30432 | -0.97147 | -1.38784 |
| H | 2.15515 | -0.22041 | -0.01056 |
| H | 1.29490 | -1.75797 | 0.20477 |
| C | -1.26738 | -0.78905 | -0.30724 |
| H | -1.30428 | -0.97149 | -1.38785 |
| H | -1.29500 | -1.75788 | 0.20481 |
| H | -2.15517 | -0.22030 | -0.01068 |
| C | 0.00004 | 1.37108 | -0.58389 |
| H | 0.89160 | 1.93371 | -0.29055 |
| H | 0.00003 | 1.23382 | -1.67126 |
| H | -0.89148 | 1.93377 | -0.29055 |

**1a$^{R}$**

| | | | |
|---|---|---|---|
| H | -1.76591 | 0.97869 | -2.12819 |
| C | -1.84246 | 0.78980 | -1.05760 |
| C | -0.82195 | 1.18412 | -0.22313 |
| C | -0.94445 | 0.91516 | 1.16696 |
| C | -2.05160 | 0.28781 | 1.67425 |
| C | -3.11202 | -0.11910 | 0.82843 |
| C | -2.99977 | 0.13776 | -0.56859 |
| C | -4.05390 | -0.26849 | -1.42281 |
| C | -5.16447 | -0.89801 | -0.91992 |
| C | -5.27444 | -1.15190 | 0.46406 |
| C | -4.26971 | -0.77028 | 1.31713 |
| H | -0.13224 | 1.20451 | 1.83224 |
| H | -2.12796 | 0.08974 | 2.74266 |
| H | -4.34938 | -0.96363 | 2.38607 |
| H | -6.16006 | -1.65172 | 0.85176 |
| H | -5.96670 | -1.20454 | -1.58861 |
| H | -3.96699 | -0.07167 | -2.49044 |
| C | 0.42996 | 1.84713 | -0.75523 |
| H | 0.31077 | 1.95748 | -1.84069 |
| C | 0.70252 | 3.20979 | -0.12011 |
| H | 1.58937 | 3.67817 | -0.56401 |
| H | -0.14574 | 3.89209 | -0.25463 |
| H | 0.89551 | 3.11538 | 0.95494 |
| B | 1.57510 | 0.80647 | -0.48701 |
| O | 1.88921 | -0.22465 | -1.32268 |
| C | 2.65393 | -1.16860 | -0.55030 |
| C | 3.28282 | -0.25235 | 0.55028 |
| O | 2.29076 | 0.78292 | 0.67638 |
| C | 3.47582 | -0.91751 | 1.89651 |
| H | 3.92415 | -0.20725 | 2.59988 |
| H | 2.52252 | -1.25270 | 2.31425 |
| H | 4.14588 | -1.78147 | 1.80925 |
| C | 4.56818 | 0.41905 | 0.09136 |
| H | 4.43793 | 0.87484 | -0.89707 |
| H | 4.82864 | 1.21083 | 0.80186 |
| H | 5.40054 | -0.29157 | 0.04048 |
| C | 3.65296 | -1.85861 | -1.45378 |
| H | 4.26215 | -1.13543 | -2.00302 |
| H | 4.31675 | -2.50903 | -0.87121 |
| H | 3.12271 | -2.47892 | -2.18449 |
| C | 1.65919 | -2.16867 | 0.02127 |
| H | 2.16020 | -2.96668 | 0.58024 |
| H | 0.94248 | -1.66961 | 0.68502 |
| H | 1.09650 | -2.61859 | -0.80341 |

**1a$^{S}$**

| | | | |
|---|---|---|---|
| H | 1.76591 | 0.97869 | -2.12819 |
| C | 1.84246 | 0.78980 | -1.05760 |
| C | 0.82195 | 1.18412 | -0.22313 |
| C | 0.94445 | 0.91516 | 1.16696 |
| C | 2.05160 | 0.28781 | 1.67425 |
| C | 3.11202 | -0.11910 | 0.82843 |
| C | 2.99977 | 0.13776 | -0.56859 |
| C | 4.05390 | -0.26849 | -1.42281 |
| C | 5.16447 | -0.89801 | -0.91992 |
| C | 5.27444 | -1.15190 | 0.46406 |
| C | 4.26971 | -0.77028 | 1.31713 |
| H | 0.13224 | 1.20451 | 1.83224 |
| H | 2.12796 | 0.08974 | 2.74266 |
| H | 4.34938 | -0.96363 | 2.38607 |
| H | 6.16006 | -1.65172 | 0.85176 |
| H | 5.96670 | -1.20454 | -1.58861 |
| H | 3.96699 | -0.07167 | -2.49044 |
| C | -0.42996 | 1.84713 | -0.75523 |
| H | -0.31077 | 1.95748 | -1.84069 |
| C | -0.70252 | 3.20979 | -0.12011 |
| H | -1.58937 | 3.67817 | -0.56401 |
| H | -0.89551 | 3.11538 | 0.95494 |
| H | 0.14574 | 3.89209 | -0.25463 |
| B | -1.57510 | 0.80647 | -0.48701 |
| O | -1.88921 | -0.22465 | -1.32268 |

| | | | |
|---|---|---|---|
| C | -2.65393 | -1.16860 | -0.55030 |
| C | -3.28282 | -0.25235 | 0.55028 |
| O | -2.29076 | 0.78292 | 0.67638 |
| C | -4.56818 | 0.41905 | 0.09136 |
| H | -4.43793 | 0.87484 | -0.89707 |
| H | -5.40054 | -0.29157 | 0.04048 |
| H | -4.82864 | 1.21083 | 0.80186 |
| C | -3.47582 | -0.91751 | 1.89651 |
| H | -2.52252 | -1.25270 | 2.31425 |
| H | -3.92415 | -0.20725 | 2.59988 |
| H | -4.14588 | -1.78147 | 1.80925 |
| C | -1.65919 | -2.16867 | 0.02127 |
| H | -1.09650 | -2.61859 | -0.80341 |
| H | -0.94248 | -1.66961 | 0.68502 |
| H | -2.16020 | -2.96668 | 0.58024 |
| C | -3.65295 | -1.85861 | -1.45378 |
| H | -4.26215 | -1.13543 | -2.00302 |
| H | -3.12271 | -2.47892 | -2.18449 |
| H | -4.31675 | -2.50903 | -0.87121 |

**TS-1''**

| | | | |
|---|---|---|---|
| O | 2.23942 | -0.25323 | -0.92485 |
| C | 3.08332 | 0.07035 | 0.15741 |
| C | 2.45839 | -0.30805 | 1.49320 |
| H | 3.16619 | -0.11991 | 2.30815 |
| H | 1.56050 | 0.29349 | 1.67115 |
| H | 2.18044 | -1.36792 | 1.51487 |
| C | 4.35646 | -0.75049 | -0.09847 |
| H | 5.10040 | -0.51700 | 0.67215 |
| H | 4.13961 | -1.82377 | -0.06253 |
| H | 4.77464 | -0.50851 | -1.08082 |
| H | 1.37390 | -0.95914 | -0.68938 |
| O | 0.17742 | -1.32906 | -0.44757 |
| O | 0.34076 | 0.87295 | -0.50138 |
| C | -0.33685 | -0.17902 | -0.38554 |
| C | -1.80285 | -0.05202 | -0.15472 |
| C | -2.39919 | 1.20792 | -0.09902 |
| C | -2.57376 | -1.20312 | 0.00768 |
| C | -3.76586 | 1.31460 | 0.11968 |
| C | -3.94036 | -1.09135 | 0.22667 |
| C | -4.53565 | 0.16589 | 0.28284 |
| H | -1.77704 | 2.09006 | -0.23042 |
| H | -2.08230 | -2.17157 | -0.04240 |
| H | -4.23472 | 2.29561 | 0.16283 |
| H | -4.54524 | -1.98702 | 0.35351 |
| H | -5.60727 | 0.25113 | 0.45420 |
| C | 3.38071 | 1.56078 | 0.08121 |
| H | 4.09222 | 1.85351 | 0.86184 |
| H | 3.80344 | 1.81335 | -0.89716 |
| H | 2.44824 | 2.11948 | 0.20882 |

**TS-2''**

| | | | |
|---|---|---|---|
| H | 0.86347 | 1.78013 | 0.08901 |
| C | 1.58260 | 1.07039 | 0.49843 |
| C | 1.16068 | 0.16119 | 1.45907 |
| C | 2.09451 | -0.79222 | 1.94497 |
| C | 3.38517 | -0.80811 | 1.48997 |
| C | 3.83106 | 0.11640 | 0.50895 |
| C | 2.90277 | 1.07723 | 0.00903 |
| C | 3.33218 | 1.99636 | -0.98119 |
| C | 4.62031 | 1.96714 | -1.45426 |
| C | 5.53699 | 1.01897 | -0.95590 |
| C | 5.14910 | 0.11395 | 0.00533 |
| H | 1.76895 | -1.52341 | 2.68043 |
| H | 4.09234 | -1.54144 | 1.87487 |
| H | 5.85698 | -0.61935 | 0.38864 |
| H | 6.55663 | 1.00463 | -1.33588 |
| H | 4.93914 | 2.67633 | -2.21544 |
| H | 2.61784 | 2.72514 | -1.36092 |
| C | -0.27793 | 0.12097 | 1.84440 |
| H | -0.71705 | 1.10041 | 1.64498 |
| C | -0.60728 | -0.36795 | 3.24143 |
| H | -1.68146 | -0.26245 | 3.42439 |
| H | -0.37012 | -1.43073 | 3.36485 |
| H | -0.05981 | 0.19858 | 4.00620 |
| B | -0.79260 | -0.91430 | 0.69946 |
| O | -0.81562 | -0.59217 | -0.63721 |
| C | -0.48697 | -1.78357 | -1.37063 |
| C | -0.83067 | -2.92096 | -0.34303 |
| O | -0.65367 | -2.25361 | 0.91987 |
| C | -2.27733 | -3.38178 | -0.42329 |
| H | -2.97009 | -2.53516 | -0.40317 |
| H | -2.45721 | -3.96628 | -1.33285 |
| H | -2.49089 | -4.01592 | 0.44418 |
| C | 0.10788 | -4.11003 | -0.39002 |
| H | 1.13592 | -3.81778 | -0.15811 |
| H | -0.20847 | -4.85639 | 0.34692 |
| H | 0.08879 | -4.57980 | -1.38089 |
| C | 0.99871 | -1.70561 | -1.69415 |
| H | 1.19537 | -0.77175 | -2.23272 |

| | | | |
|---|---|---|---|
| H | 1.60338 | -1.69707 | -0.77970 |
| H | 1.32216 | -2.54496 | -2.31982 |
| C | -1.31468 | -1.81418 | -2.63867 |
| H | -2.37791 | -1.68854 | -2.41462 |
| H | -1.00277 | -0.99858 | -3.30149 |
| H | -1.17266 | -2.76074 | -3.17420 |
| O | -2.83000 | -0.45033 | 1.09218 |
| C | -3.29488 | 0.22160 | 0.10118 |
| C | -2.87499 | 1.63025 | -0.07050 |
| C | -2.72702 | 2.46378 | 1.03965 |
| C | -2.59575 | 2.10727 | -1.35173 |
| C | -2.29583 | 3.77289 | 0.86758 |
| C | -2.15181 | 3.41130 | -1.51768 |
| C | -2.00137 | 4.24297 | -0.40935 |
| H | -2.95208 | 2.07415 | 2.03083 |
| H | -2.71155 | 1.43662 | -2.19999 |
| H | -2.18651 | 4.42823 | 1.72919 |
| H | -1.91915 | 3.78422 | -2.51311 |
| H | -1.65487 | 5.26617 | -0.54301 |
| O | -4.06797 | -0.34664 | -0.66402 |

**TS-1'**

| | | | |
|---|---|---|---|
| H | -1.78188 | -1.34903 | -1.86156 |
| C | -1.97480 | -1.18434 | -0.80407 |
| C | -0.95255 | -1.29407 | 0.11330 |
| C | -1.24517 | -1.04371 | 1.48161 |
| C | -2.50429 | -0.69951 | 1.88979 |
| C | -3.56583 | -0.57722 | 0.95741 |
| C | -3.28997 | -0.83260 | -0.41640 |
| C | -4.34150 | -0.70844 | -1.35691 |
| C | -5.60472 | -0.34955 | -0.95812 |
| C | -5.87641 | -0.09926 | 0.40339 |
| C | -4.87760 | -0.21172 | 1.33883 |
| H | -0.43487 | -1.10593 | 2.20545 |
| H | -2.70741 | -0.51048 | 2.94305 |
| H | -5.08255 | -0.01934 | 2.39115 |
| H | -6.88180 | 0.18360 | 0.70955 |
| H | -6.40277 | -0.25730 | -1.69244 |
| H | -4.12806 | -0.90236 | -2.40716 |
| C | 0.47344 | -1.55375 | -0.30046 |
| H | 0.94664 | -2.15620 | 0.48457 |
| C | 0.62186 | -2.21760 | -1.65976 |
| H | 0.34004 | -1.52757 | -2.46339 |
| H | 1.66183 | -2.50077 | -1.84241 |
| H | -0.00763 | -3.11345 | -1.74061 |
| B | 1.04421 | -0.04057 | -0.27147 |
| O | 0.81374 | 0.78386 | -1.34974 |
| C | 0.53627 | 2.09259 | -0.83582 |
| C | 1.24955 | 2.05697 | 0.55686 |
| O | 1.14827 | 0.67009 | 0.90633 |
| C | 2.72531 | 2.41479 | 0.45238 |
| H | 3.20765 | 1.82534 | -0.33502 |
| H | 2.86475 | 3.47830 | 0.22832 |
| H | 3.22008 | 2.19910 | 1.40642 |
| C | 0.57673 | 2.87834 | 1.63728 |
| H | -0.43562 | 2.51443 | 1.83377 |
| H | 1.15010 | 2.80517 | 2.56843 |
| H | 0.52489 | 3.93570 | 1.34982 |
| C | -0.97511 | 2.22705 | -0.70406 |
| H | -1.43806 | 1.99313 | -1.66896 |
| H | -1.37248 | 1.52560 | 0.03834 |
| H | -1.26462 | 3.24361 | -0.41413 |
| C | 1.07759 | 3.12557 | -1.80288 |
| H | 2.12956 | 2.93886 | -2.03616 |
| H | 0.51106 | 3.08341 | -2.73972 |
| H | 0.97934 | 4.13714 | -1.38982 |
| O | 2.95057 | -0.69863 | -0.61732 |
| C | 3.83853 | -1.34848 | 0.21068 |
| C | 3.54725 | -1.14192 | 1.69356 |
| H | 4.31560 | -1.63199 | 2.30268 |
| H | 3.51618 | -0.07740 | 1.93771 |
| H | 2.57233 | -1.56537 | 1.95677 |
| C | 3.90929 | -2.83399 | -0.15101 |
| H | 4.70100 | -3.33024 | 0.42197 |
| H | 2.95859 | -3.32687 | 0.08120 |
| H | 4.11238 | -2.95709 | -1.22025 |
| C | 5.18293 | -0.67032 | -0.15968 |
| H | 5.97953 | -1.15533 | 0.41614 |
| H | 5.38593 | -0.78326 | -1.22879 |
| H | 5.15385 | 0.39390 | 0.09270 |

**PhCOOBpin**

| | | | |
|---|---|---|---|
| O | 1.88718 | 2.80504 | 0.40076 |
| O | 0.28209 | 1.76927 | -0.74165 |
| C | 1.48607 | 1.80153 | -0.10626 |
| C | 2.25198 | 0.52613 | -0.14693 |
| C | 3.35697 | 0.40194 | 0.69623 |
| C | 1.91233 | -0.52138 | -1.00442 |
| C | 4.09931 | -0.77017 | 0.70154 |
| C | 2.66172 | -1.69080 | -1.00352 |

| | | | |
|---|---|---|---|
| C | 3.75058 | -1.81821 | -0.14649 |
| H | 3.60935 | 1.23892 | 1.34319 |
| H | 1.07268 | -0.41259 | -1.68827 |
| H | 4.95417 | -0.86925 | 1.36739 |
| H | 2.39871 | -2.50407 | -1.67693 |
| H | 4.33437 | -2.73687 | -0.14386 |
| B | -0.72481 | 0.92236 | -0.37849 |
| O | -0.74647 | 0.24926 | 0.80526 |
| C | -1.82896 | -0.69587 | 0.71288 |
| C | -2.75215 | -0.02358 | -0.36126 |
| O | -1.79884 | 0.68629 | -1.17669 |
| C | -3.52090 | -0.99308 | -1.23238 |
| H | -2.84754 | -1.63810 | -1.80338 |
| H | -4.17867 | -1.62255 | -0.62102 |
| C | -3.68371 | 1.01679 | 0.24123 |
| H | -3.13225 | 1.70193 | 0.89575 |
| H | -4.48741 | 0.55144 | 0.82214 |
| C | -2.45800 | -0.85936 | 2.07931 |
| H | -2.73300 | 0.10764 | 2.50905 |
| H | -3.35457 | -1.48799 | 2.01897 |
| H | -1.74663 | -1.34232 | 2.75795 |
| C | -1.23493 | -2.01104 | 0.23236 |
| H | -1.98162 | -2.81262 | 0.22872 |
| H | -0.82912 | -1.91135 | -0.78071 |
| H | -0.41411 | -2.29549 | 0.89938 |
| H | -4.14266 | -0.43843 | -1.94336 |
| H | -4.13118 | 1.60353 | -0.56775 |

**13**

| | | | |
|---|---|---|---|
| O | 1.55553 | -0.83917 | -0.32834 |
| C | 2.69658 | -0.03841 | 0.00400 |
| B | 0.27163 | -0.46498 | -0.18898 |
| O | -0.17079 | 0.72311 | 0.34907 |
| C | -1.57777 | 0.80237 | 0.07371 |
| C | -1.96199 | -0.70881 | -0.06224 |
| O | -0.75691 | -1.27798 | -0.58677 |
| C | -2.22023 | -1.36132 | 1.28857 |
| H | -1.41269 | -1.12577 | 1.99195 |
| H | -3.16990 | -1.03315 | 1.72553 |
| C | -3.10177 | -0.99044 | -1.01856 |
| H | -4.01486 | -0.47321 | -0.69937 |
| H | -3.30795 | -2.06613 | -1.04000 |
| C | -1.73873 | 1.56054 | -1.23572 |
| H | -1.26543 | 1.01343 | -2.05961 |
| H | -2.79345 | 1.72054 | -1.48524 |
| H | -1.25076 | 2.53714 | -1.14587 |
| C | -2.26396 | 1.53594 | 1.20620 |
| H | -2.01818 | 1.09099 | 2.17443 |
| H | -1.93915 | 2.58225 | 1.22122 |
| H | -3.35286 | 1.51834 | 1.07611 |
| H | -2.25173 | -2.44791 | 1.15630 |
| H | -2.85396 | -0.67498 | -2.03586 |
| C | 2.67729 | 0.28088 | 1.49318 |
| H | 1.80137 | 0.88694 | 1.74585 |
| H | 2.64500 | -0.64660 | 2.07705 |
| H | 3.57937 | 0.83489 | 1.77775 |
| C | 3.89697 | -0.90278 | -0.34529 |
| H | 3.87152 | -1.83445 | 0.23080 |
| H | 3.88008 | -1.15992 | -1.41026 |
| H | 4.83310 | -0.37764 | -0.12455 |
| C | 2.68879 | 1.23461 | -0.83246 |
| H | 2.65781 | 0.98457 | -1.89956 |
| H | 1.81669 | 1.84944 | -0.58855 |
| H | 3.59409 | 1.82294 | -0.64318 |

**TS-1**

| | | | |
|---|---|---|---|
| O | -2.35983 | 0.10003 | -0.93495 |
| C | -2.94261 | -0.26776 | 0.26816 |
| C | -4.38304 | 0.25837 | 0.19920 |
| H | -4.92789 | 0.00424 | 1.11658 |
| H | -4.37850 | 1.34648 | 0.07441 |
| H | -4.90204 | -0.18564 | -0.65720 |
| C | -2.94453 | -1.78676 | 0.43689 |
| H | -3.46574 | -2.09412 | 1.35185 |
| H | -3.43774 | -2.25741 | -0.42167 |
| H | -1.91623 | -2.16377 | 0.49357 |
| C | 0.01510 | 2.40723 | -0.77348 |
| C | 0.74157 | 3.47677 | -0.26421 |
| C | 0.54679 | 1.12986 | -0.60602 |
| C | 1.95939 | 3.27572 | 0.39370 |
| C | 1.76223 | 0.91131 | 0.08421 |
| C | 2.47933 | 1.99379 | 0.57428 |
| C | 0.82780 | -1.07276 | -0.57721 |
| C | 1.94771 | -0.52980 | 0.10479 |
| H | -0.94534 | 2.53421 | -1.26662 |
| H | 0.35607 | 4.48806 | -0.37647 |
| H | 2.50738 | 4.13446 | 0.77595 |
| H | 3.42509 | 1.85026 | 1.09342 |
| C | 0.67542 | -2.44988 | -0.74408 |
| C | 2.92564 | -1.37554 | 0.60799 |

```
C   2.76746 -2.75216  0.44893
C   1.65561 -3.28004 -0.21553
H  -0.18748 -2.85180 -1.26949
H   3.80075 -0.97617  1.11706
H   3.52304 -3.42780  0.84439
H   1.56044 -4.35825 -0.32525
N   0.02201 -0.06403 -1.05148
H  -1.13995 -0.12501 -1.16379
C  -2.21266  0.39983  1.43885
H  -1.18978  0.01175  1.52468
H  -2.15429  1.48331  1.28367
H  -2.73050  0.20605  2.38604
```

**TS-2**

```
H  -2.03117  2.07332  0.49822
C  -2.54793  1.39091 -0.17653
C  -1.81757  0.49518 -0.92387
C  -2.51698 -0.39263 -1.78489
C  -3.88320 -0.35892 -1.88277
C  -4.64723  0.56089 -1.12369
C  -3.95957  1.45261 -0.25059
C  -4.71551  2.37323  0.51587
C  -6.08392  2.41020  0.42387
C  -6.76384  1.52662 -0.44157
C  -6.05927  0.62343 -1.19729
H  -1.94846 -1.12045 -2.36178
H  -4.40243 -1.04773 -2.54808
H  -6.57894 -0.06075 -1.86686
H  -7.84955  1.56480 -0.50712
H  -6.65134  3.12306  1.01942
H  -4.18841  3.05412  1.18283
C  -0.31402  0.40010 -0.79227
H   0.00624  1.20958 -0.12606
C   0.42157  0.50286 -2.12547
H   1.50617  0.51430 -1.97070
H   0.20125 -0.35531 -2.77138
H   0.14602  1.41678 -2.66620
B  -0.09890 -0.97302 -0.05572
O  -0.26764 -1.15034  1.28890
C  -0.49696 -2.55203  1.50632
C   0.16832 -3.20239  0.24422
O   0.06405 -2.14854 -0.72964
C   1.64840 -3.49199  0.43764
H   2.16881 -2.60175  0.80668
H   1.80740 -4.32315  1.13387
H   2.07931 -3.76855 -0.53180
C  -0.55110 -4.42615 -0.28536
H  -1.57589 -4.18969 -0.58488
H  -0.02114 -4.81343 -1.16256
H  -0.57942 -5.21764  0.47351
C  -2.00700 -2.74141  1.53932
H  -2.42634 -2.10246  2.32382
H  -2.45814 -2.44039  0.58592
H  -2.28343 -3.78089  1.74799
C   0.12671 -2.95563  2.82555
H   1.17935 -2.66253  2.87001
H  -0.40203 -2.46441  3.65012
H   0.05541 -4.04009  2.97359
C   1.62516  1.32636  2.20635
C   1.49691  2.64784  2.62394
C   2.52394  1.04940  1.17020
C   2.24180  3.66567  2.02569
C   3.28238  2.09167  0.55865
C   3.14295  3.39661  0.98401
C   3.70106  0.04761 -0.36564
C   4.07134  1.42067 -0.46858
H   1.03520  0.52196  2.63675
H   0.80321  2.89417  3.42509
H   2.11914  4.69041  2.37111
H   3.71025  4.20562  0.52738
C   4.25054 -0.90911 -1.22300
C   4.98590  1.82646 -1.41858
C   5.53580  0.85808 -2.27245
C   5.17286 -0.48575 -2.17589
H   3.94850 -1.94996 -1.13992
H   5.27776  2.87073 -1.51444
H   6.25765  1.16283 -3.02765
H   5.61517 -1.20921 -2.85740
N   2.77342 -0.17136  0.61368
```

**11**

```
C  -1.02601  2.47804  0.06647
C  -2.05525  3.41014  0.09155
C  -1.37282  1.12901  0.02942
C  -3.39870  3.01963  0.08101
C  -2.72208  0.72241  0.01896
C  -3.73961  1.67587  0.04489
C  -1.37282 -1.12901 -0.02942
C  -2.72208 -0.72241 -0.01896
H   0.01371  2.78564  0.07695
```

| | | | |
|---|---|---|---|
| H | -1.80716 | 4.46960 | 0.12097 |
| H | -4.17960 | 3.77701 | 0.10201 |
| H | -4.78347 | 1.36759 | 0.03715 |
| C | -1.02601 | -2.47804 | -0.06647 |
| C | -3.73961 | -1.67587 | -0.04489 |
| C | -3.39870 | -3.01964 | -0.08101 |
| C | -2.05525 | -3.41014 | -0.09155 |
| H | 0.01371 | -2.78564 | -0.07695 |
| H | -4.78347 | -1.36759 | -0.03715 |
| H | -4.17960 | -3.77701 | -0.10201 |
| H | -1.80715 | -4.46960 | -0.12097 |
| N | -0.54274 | 0.00000 | 0.00000 |
| B | 0.88947 | 0.00000 | 0.00000 |
| O | 1.64039 | 1.13853 | 0.07279 |
| C | 2.99851 | 0.75308 | -0.21017 |
| C | 2.99851 | -0.75308 | 0.21017 |
| O | 1.64039 | -1.13853 | -0.07279 |
| C | 3.93600 | -1.63527 | -0.58537 |
| H | 3.67436 | -1.63956 | -1.64698 |
| H | 4.97235 | -1.29306 | -0.47783 |
| C | 3.21377 | -0.94039 | 1.70417 |
| H | 2.55371 | -0.27959 | 2.27826 |
| H | 4.25019 | -0.73257 | 1.99169 |
| C | 3.93600 | 1.63527 | 0.58537 |
| H | 3.67436 | 1.63957 | 1.64698 |
| H | 4.97235 | 1.29306 | 0.47783 |
| H | 3.87751 | 2.66531 | 0.21714 |
| C | 3.21377 | 0.94039 | -1.70417 |
| H | 4.25019 | 0.73257 | -1.99169 |
| H | 2.55371 | 0.27959 | -2.27826 |
| H | 2.97830 | 1.97590 | -1.97148 |
| H | 3.87750 | -2.66531 | -0.21714 |
| H | 2.97830 | -1.97590 | 1.97148 |

**12**

| | | | |
|---|---|---|---|
| H | 0.75291 | -2.17723 | 0.00000 |
| C | 0.58368 | -1.10076 | 0.00000 |
| C | 1.69722 | -0.23883 | -0.00000 |
| C | 1.42674 | 1.17445 | 0.00000 |
| C | 0.15338 | 1.65402 | 0.00000 |
| C | -0.97429 | 0.78321 | 0.00000 |
| C | -0.73978 | -0.62545 | 0.00000 |
| C | -1.85830 | -1.49789 | 0.00000 |
| C | -3.13900 | -1.00195 | 0.00000 |
| C | -3.36481 | 0.38894 | -0.00000 |
| C | -2.29859 | 1.26095 | 0.00000 |
| H | 2.25744 | 1.87549 | 0.00000 |
| H | -0.02335 | 2.72889 | 0.00000 |
| H | -2.46800 | 2.33688 | -0.00000 |
| H | -4.38359 | 0.77120 | -0.00000 |
| H | -3.98625 | -1.68522 | 0.00000 |
| H | -1.68278 | -2.57269 | 0.00000 |
| C | 2.99726 | -0.76973 | -0.00000 |
| H | 3.07490 | -1.85846 | 0.00000 |
| C | 4.27429 | -0.01675 | -0.00000 |
| H | 4.88684 | -0.27427 | -0.87779 |
| H | 4.13813 | 1.06845 | -0.00000 |
| H | 4.88683 | -0.27426 | 0.87779 |

**$23^{+}$**

| | | | |
|---|---|---|---|
| Cu | 0.40327 | 0.79542 | 0.37810 |
| C | -2.08626 | 0.42441 | 2.06597 |
| C | -3.01621 | -0.76637 | 2.38295 |
| C | -1.32652 | -1.44419 | 1.09191 |
| N | -1.06092 | -0.20212 | 1.22277 |
| H | -3.99951 | -0.66505 | 1.91094 |
| O | -2.36038 | -1.89801 | 1.77975 |
| C | 1.63976 | -0.63695 | -2.03401 |
| C | 1.92266 | -2.12809 | -2.27362 |
| C | 0.33025 | -1.89809 | -0.73767 |
| N | 0.71456 | -0.68932 | -0.89326 |
| H | 2.87621 | -2.43760 | -1.83010 |
| O | 0.86771 | -2.79450 | -1.55198 |
| C | -0.62275 | -2.47682 | 0.26511 |
| C | 0.20358 | -3.41454 | 1.20248 |
| H | -0.44718 | -3.68455 | 2.04290 |
| C | -1.70732 | -3.31008 | -0.47641 |
| H | -2.21935 | -3.91417 | 0.27881 |
| H | -1.18124 | -3.99527 | -1.14998 |
| H | 0.40308 | -4.32964 | 0.63526 |
| H | 1.09130 | -0.20062 | -2.88140 |
| H | 1.87586 | -2.43725 | -3.31965 |
| H | -1.60668 | 0.81290 | 2.97279 |
| H | -3.13406 | -0.96808 | 3.45040 |
| C | -2.73540 | 1.56093 | 1.31970 |
| C | -2.88810 | 2.81411 | 1.90640 |
| C | -3.18110 | 1.35362 | 0.01201 |
| C | -3.48355 | 3.85194 | 1.19500 |
| H | -2.53529 | 2.98380 | 2.92293 |
| C | -3.77758 | 2.38830 | -0.69569 |

| | | | |
|---|---|---|---|
| H | -3.04963 | 0.38204 | -0.46303 |
| C | -3.92916 | 3.64011 | -0.10501 |
| C | 2.86754 | 0.18781 | -1.76719 |
| C | 3.42413 | 0.22733 | -0.48933 |
| C | 3.49295 | 0.86982 | -2.80997 |
| C | 4.59467 | 0.93755 | -0.25725 |
| H | 2.93776 | -0.29681 | 0.33199 |
| C | 4.67194 | 1.57050 | -2.57972 |
| H | 3.05821 | 0.85244 | -3.80887 |
| C | 5.22440 | 1.60426 | -1.30322 |
| C | 1.49912 | -2.81591 | 1.67548 |
| C | 1.53158 | -1.82313 | 2.65945 |
| C | 2.70737 | -3.23997 | 1.11602 |
| C | 2.74031 | -1.27329 | 3.07455 |
| H | 0.60754 | -1.49897 | 3.13548 |
| C | 3.91848 | -2.70325 | 1.54041 |
| H | 2.69564 | -4.01863 | 0.35353 |
| C | 3.93729 | -1.71742 | 2.52100 |
| H | 2.74818 | -0.50851 | 3.84831 |
| H | 4.85036 | -3.06034 | 1.10715 |
| C | -2.70640 | -2.46910 | -1.22535 |
| C | -2.35050 | -1.79109 | -2.39511 |
| C | -4.01576 | -2.34093 | -0.75435 |
| C | -3.27404 | -0.99737 | -3.06684 |
| H | -1.35081 | -1.91329 | -2.80962 |
| C | -4.94486 | -1.55678 | -1.42988 |
| H | -4.30914 | -2.87535 | 0.14792 |
| C | -4.57428 | -0.87808 | -2.58589 |
| H | -2.98242 | -0.48496 | -3.98139 |
| H | -5.96343 | -1.48075 | -1.05510 |
| H | 6.14339 | 2.15784 | -1.12286 |
| H | -4.39325 | 4.45261 | -0.65997 |
| H | 4.88336 | -1.29992 | 2.85914 |
| H | -5.30080 | -0.26838 | -3.11907 |
| O | 1.25597 | 2.31891 | 0.76306 |
| C | 1.07022 | 3.57084 | 0.15519 |
| C | 2.41267 | 3.96208 | -0.45823 |
| H | 2.34904 | 4.95307 | -0.92230 |
| H | 3.19031 | 3.97950 | 0.31254 |
| H | 2.70259 | 3.23256 | -1.22210 |
| C | -0.01221 | 3.47900 | -0.91280 |
| H | 0.27663 | 2.73421 | -1.66945 |
| H | -0.96732 | 3.17778 | -0.46236 |
| H | -0.16908 | 4.43434 | -1.42677 |
| H | 5.01364 | 0.96859 | 0.74637 |
| H | 5.15678 | 2.09740 | -3.39865 |
| C | 0.67238 | 4.54938 | 1.25818 |
| H | 1.44646 | 4.58276 | 2.03184 |
| H | 0.53834 | 5.55684 | 0.84808 |
| H | -0.27049 | 4.23241 | 1.71791 |
| H | -4.12005 | 2.21612 | -1.71398 |
| H | -3.59746 | 4.82937 | 1.65874 |

**24⁺**

| | | | |
|---|---|---|---|
| Cu | 0.15760 | -0.35378 | -0.54790 |
| C | 1.47517 | 1.12735 | 1.76423 |
| C | 1.13505 | 2.40734 | 2.54959 |
| C | -0.62835 | 1.76342 | 1.35261 |
| N | 0.25675 | 0.92841 | 0.96472 |
| H | 1.64677 | 3.28757 | 2.14380 |
| O | -0.27705 | 2.58564 | 2.32896 |
| C | -2.49852 | -0.15330 | -2.15012 |
| C | -3.90473 | 0.23080 | -1.68279 |
| C | -2.39072 | 1.11747 | -0.32974 |
| N | -1.67753 | 0.30740 | -1.01767 |
| H | -4.40711 | -0.59828 | -1.16990 |
| O | -3.65170 | 1.26197 | -0.70919 |
| C | -2.00819 | 2.00218 | 0.81904 |
| C | -3.06466 | 1.88790 | 1.94194 |
| H | -2.69323 | 2.48054 | 2.78794 |
| C | -2.04107 | 3.48372 | 0.30329 |
| H | -2.08636 | 4.11826 | 1.19393 |
| H | -2.98803 | 3.60580 | -0.23528 |
| H | -3.96484 | 2.39710 | 1.57856 |
| H | -2.21172 | 0.45859 | -3.01991 |
| H | -4.54919 | 0.63795 | -2.46372 |
| H | 1.58641 | 0.27483 | 2.44553 |
| H | 1.31001 | 2.33329 | 3.62490 |
| C | 2.70109 | 1.23177 | 0.89906 |
| C | 3.88280 | 0.58822 | 1.25628 |
| C | 2.66789 | 2.02133 | -0.25187 |
| C | 5.02699 | 0.74905 | 0.48125 |
| H | 3.90980 | -0.04811 | 2.13970 |
| C | 3.81139 | 2.18475 | -1.02236 |
| H | 1.74030 | 2.50995 | -0.54575 |
| C | 4.99592 | 1.55301 | -0.65285 |
| C | -2.32783 | -1.60023 | -2.50914 |
| C | -1.96948 | -2.54789 | -1.55191 |
| C | -2.57688 | -2.00789 | -3.82011 |
| C | -1.84418 | -3.88508 | -1.90893 |
| H | -1.76763 | -2.24738 | -0.52563 |

| | | | |
|---|---|---|---|
| C | -2.47442 | -3.34890 | -4.16988 |
| H | -2.84672 | -1.27051 | -4.57582 |
| C | -2.10123 | -4.28890 | -3.21438 |
| C | -3.44647 | 0.50317 | 2.39821 |
| C | -2.72044 | -0.63958 | 2.06148 |
| C | -4.58711 | 0.34932 | 3.19069 |
| C | -3.13064 | -1.90299 | 2.48703 |
| H | -1.82327 | -0.57623 | 1.45486 |
| C | -4.98742 | -0.90499 | 3.63312 |
| H | -5.17323 | 1.22623 | 3.46311 |
| C | -4.26316 | -2.04005 | 3.27963 |
| H | -2.55935 | -2.77784 | 2.17653 |
| H | -5.87811 | -0.99835 | 4.25088 |
| C | -0.87386 | 3.89813 | -0.54814 |
| C | -0.72354 | 3.45071 | -1.86434 |
| C | 0.08225 | 4.77657 | -0.03051 |
| C | 0.34951 | 3.87568 | -2.64072 |
| H | -1.47113 | 2.79448 | -2.30725 |
| C | 1.14921 | 5.21339 | -0.80859 |
| H | -0.02850 | 5.13882 | 0.99115 |
| C | 1.28484 | 4.76317 | -2.11734 |
| H | 0.44453 | 3.52501 | -3.66636 |
| H | 1.87236 | 5.91195 | -0.39315 |
| H | -2.00553 | -5.33689 | -3.49020 |
| H | 5.89268 | 1.67982 | -1.25566 |
| H | -4.58642 | -3.02359 | 3.61322 |
| H | 2.11306 | 5.10948 | -2.73217 |
| O | 1.07729 | -1.51176 | -1.57377 |
| C | 1.68801 | -1.28401 | -2.81294 |
| C | 1.30253 | -2.41924 | -3.75302 |
| H | 1.76812 | -2.29802 | -4.73820 |
| H | 1.62099 | -3.37839 | -3.33049 |
| H | 0.21501 | -2.45010 | -3.87825 |
| C | 1.23789 | 0.06579 | -3.36458 |
| H | 0.14819 | 0.06247 | -3.50366 |
| H | 1.50194 | 0.86732 | -2.66158 |
| H | 1.70375 | 0.28976 | -4.33130 |
| H | -1.54018 | -4.61290 | -1.15994 |
| H | -2.67222 | -3.65845 | -5.19379 |
| C | 3.19554 | -1.28164 | -2.54737 |
| H | 3.52332 | -2.27934 | -2.23483 |
| H | 3.75087 | -1.00316 | -3.45063 |
| H | 3.43097 | -0.57146 | -1.74653 |
| H | 3.77455 | 2.80450 | -1.91608 |
| H | 5.94536 | 0.24015 | 0.76635 |
| H | -0.82332 | -1.71566 | 3.27238 |
| O | 0.12356 | -1.90968 | 3.36737 |
| O | -0.04482 | -2.05729 | 1.15421 |
| C | 0.63418 | -2.13782 | 2.16255 |
| C | 2.07576 | -2.44028 | 2.16748 |
| C | 2.70341 | -2.67943 | 0.94168 |
| C | 2.80959 | -2.46453 | 3.35888 |
| C | 4.06813 | -2.93245 | 0.91237 |
| C | 4.17334 | -2.71801 | 3.31834 |
| C | 4.80128 | -2.95035 | 2.09562 |
| H | 2.11046 | -2.65205 | 0.02879 |
| H | 2.30605 | -2.28754 | 4.30574 |
| H | 4.56464 | -3.11660 | -0.03764 |
| H | 4.74934 | -2.74184 | 4.24075 |
| H | 5.87030 | -3.15324 | 2.06811 |

**$17^{+}$**

| | | | |
|---|---|---|---|
| Cu | -0.84864 | 0.47291 | -0.03575 |
| C | 0.14071 | 0.98290 | -2.86615 |
| C | 1.44633 | 0.68993 | -3.61749 |
| C | 1.71493 | 0.24273 | -1.45943 |
| N | 0.45439 | 0.45306 | -1.52639 |
| H | 1.80452 | 1.51526 | -4.23650 |
| O | 2.40332 | 0.45943 | -2.56424 |
| C | -0.01916 | -1.62693 | 2.01635 |
| C | 1.28676 | -2.38997 | 2.34168 |
| C | 1.65962 | -0.89675 | 0.73880 |
| N | 0.45007 | -0.62453 | 1.05714 |
| H | 1.20646 | -3.47225 | 2.21385 |
| O | 2.23213 | -1.89552 | 1.37461 |
| C | 2.51057 | -0.13334 | -0.24055 |
| C | 3.76204 | -0.92182 | -0.63821 |
| H | 4.42750 | -0.22959 | -1.16769 |
| C | 2.89265 | 1.22478 | 0.45133 |
| H | 1.95343 | 1.75304 | 0.65951 |
| H | 3.45178 | 1.80990 | -0.28871 |
| H | 4.27759 | -1.20300 | 0.28721 |
| H | -0.40024 | -1.11263 | 2.90660 |
| H | 1.68541 | -2.15779 | 3.33459 |
| H | -0.70518 | 0.42290 | -3.27676 |
| H | 1.39346 | -0.22747 | -4.21318 |
| C | -0.23643 | 2.43758 | -2.72836 |
| C | -1.58085 | 2.80762 | -2.70854 |
| C | 0.74576 | 3.40739 | -2.50424 |
| C | -1.93922 | 4.13148 | -2.47027 |
| H | -2.34810 | 2.05000 | -2.85373 |

| | | | |
|---|---|---|---|
| C | 0.38700 | 4.72941 | -2.26601 |
| H | 1.80033 | 3.13079 | -2.50066 |
| C | -0.95843 | 5.09278 | -2.24663 |
| C | -1.13591 | -2.47121 | 1.44590 |
| C | -1.25701 | -2.69322 | 0.07421 |
| C | -2.07736 | -3.03455 | 2.30644 |
| C | -2.30380 | -3.45911 | -0.42818 |
| H | -0.55549 | -2.23201 | -0.61938 |
| C | -3.11529 | -3.81149 | 1.80699 |
| H | -2.00757 | -2.85038 | 3.37792 |
| C | -3.23385 | -4.02253 | 0.43712 |
| C | 3.55412 | -2.14550 | -1.49481 |
| C | 4.67339 | -2.74017 | -2.08307 |
| C | 2.30672 | -2.72453 | -1.72582 |
| C | 4.54999 | -3.87595 | -2.87127 |
| H | 5.65786 | -2.30346 | -1.91914 |
| C | 2.17655 | -3.86269 | -2.51726 |
| H | 1.40596 | -2.30242 | -1.28607 |
| C | 3.29827 | -4.44340 | -3.09281 |
| H | 5.43680 | -4.32116 | -3.31762 |
| H | 1.19137 | -4.29734 | -2.67555 |
| C | 3.70506 | 1.05015 | 1.70172 |
| C | 3.08093 | 0.80314 | 2.92752 |
| C | 5.09913 | 1.10604 | 1.66102 |
| C | 3.83054 | 0.60695 | 4.08098 |
| H | 1.99351 | 0.77636 | 2.97785 |
| C | 5.85198 | 0.91129 | 2.81351 |
| H | 5.60077 | 1.31108 | 0.71590 |
| C | 5.21958 | 0.65752 | 4.02561 |
| H | 3.32901 | 0.42569 | 5.02964 |
| H | 6.93761 | 0.96302 | 2.76519 |
| H | -4.05766 | -4.61459 | 0.04474 |
| H | -1.23990 | 6.12747 | -2.06224 |
| H | 3.20065 | -5.33375 | -3.71004 |
| H | 5.80834 | 0.50897 | 4.92821 |
| C | -5.23717 | -0.82801 | -1.04056 |
| C | -4.53068 | -0.51231 | 0.12130 |
| C | -5.10323 | -0.72733 | 1.37655 |
| C | -6.38309 | -1.25516 | 1.46715 |
| C | -7.08701 | -1.57120 | 0.30759 |
| C | -6.51585 | -1.35817 | -0.94496 |
| H | -4.76595 | -0.65957 | -2.00596 |
| H | -4.52614 | -0.48531 | 2.26578 |
| H | -6.83581 | -1.42423 | 2.44178 |
| H | -8.09014 | -1.98706 | 0.38042 |
| H | -7.07182 | -1.60758 | -1.84612 |
| C | -3.15378 | -0.00694 | 0.02731 |
| O | -2.47376 | 0.21584 | 1.07998 |
| O | -2.58393 | 0.18062 | -1.08784 |
| O | -0.43343 | 2.55733 | 0.48709 |
| C | -0.91805 | 3.31034 | 1.62901 |
| C | -0.70594 | 2.41630 | 2.83441 |
| H | 0.35815 | 2.18220 | 2.95120 |
| H | -1.04294 | 2.92520 | 3.74347 |
| H | -1.27807 | 1.48866 | 2.72788 |
| C | -0.08425 | 4.57708 | 1.72329 |
| H | 0.97802 | 4.33371 | 1.84187 |
| H | -0.20343 | 5.18813 | 0.81873 |
| H | -0.39696 | 5.18483 | 2.57922 |
| H | -0.44535 | 3.13620 | -0.28553 |
| H | -2.39661 | -3.60608 | -1.50213 |
| H | -3.84665 | -4.23918 | 2.48942 |
| H | -2.99035 | 4.41164 | -2.45679 |
| H | 1.15749 | 5.47806 | -2.09460 |
| C | -2.38998 | 3.62280 | 1.41896 |
| H | -2.53430 | 4.20610 | 0.50089 |
| H | -2.96740 | 2.69581 | 1.34490 |
| H | -2.78293 | 4.21079 | 2.25582 |

**$18^{+}$**

| | | | |
|---|---|---|---|
| Cu | 0.39747 | 0.05239 | -0.01346 |
| O | -3.04805 | 1.40009 | 1.74973 |
| O | -2.81945 | -1.82415 | -1.72761 |
| O | 2.06641 | 0.00003 | -1.08197 |
| N | -0.94382 | -0.80437 | -1.17278 |
| N | -1.04518 | 0.69245 | 1.15720 |
| O | 2.04775 | 0.30466 | 1.06205 |
| C | -2.19836 | -0.93489 | -0.97268 |
| C | -0.50515 | -1.78992 | -2.16858 |
| H | -0.15338 | -1.25174 | -3.05751 |
| C | -2.30759 | 0.62528 | 0.97721 |
| C | -3.07741 | -0.21823 | 0.00694 |
| C | 0.61728 | -2.97415 | -0.25985 |
| H | -0.23803 | -2.72652 | 0.36942 |
| C | -3.38981 | 1.93197 | -1.34955 |
| C | 0.23663 | 2.71431 | 1.59038 |
| C | -3.88689 | -1.28825 | 0.79949 |
| H | -4.43587 | -0.75611 | 1.58519 |
| H | -4.62348 | -1.70906 | 0.10766 |
| C | -4.05465 | 0.71189 | -0.77316 |
| H | -4.51940 | 0.10169 | -1.55652 |

```
H  -4.84285  1.00778 -0.07350
C  -3.55422  3.17921 -0.74047
H  -4.19499  3.26092  0.13647
C  -0.74948  1.72411  2.15801
H  -0.30361  1.23908  3.03525
C  -2.12071  4.21092 -2.37953
H  -1.63450  5.09614 -2.78402
C  -1.42436 -3.17253  3.01075
H  -0.81991 -2.98624  3.89605
C  -1.81093 -2.56070 -2.45084
H  -2.09684 -2.58598 -3.50475
H  -1.79089 -3.57707 -2.04243
C   2.72145  0.17753 -0.00770
C   0.13746  3.09362  0.24983
H  -0.67419  2.71340 -0.37081
C  -2.99995 -3.64551  0.77449
H  -3.62949 -3.83596 -0.09360
C  -1.94927  2.97561 -2.99668
H  -1.33269  2.89281 -3.88930
C  -2.92945  4.31131 -1.25252
H  -3.08006  5.27635 -0.77332
C  -2.58363  1.84721 -2.48833
H  -2.47490  0.89446 -3.00498
C   2.81276 -3.60831 -1.84031
H   3.67392 -3.85096 -2.45930
C   0.63269 -2.60497 -1.60677
C   4.18696  0.22568 -0.00353
C  -2.19656 -4.65949  1.28487
H  -2.19626 -5.63960  0.81264
C  -2.15533  2.27851  2.46805
H  -2.42545  2.24787  3.52596
H  -2.30113  3.28948  2.07195
C   1.08184  3.94682 -0.30857
H   0.99831  4.22729 -1.35654
C   1.73370 -2.92966 -2.39389
H   1.76113 -2.62498 -3.43898
C  -3.03012 -2.38335  1.37439
C   2.80352 -3.95697 -0.49330
H   3.65701 -4.47273 -0.05865
C  -1.40187 -4.42334  2.40152
H  -0.77556 -5.21649  2.80447
C   1.70165 -3.64323  0.29538
H   1.68208 -3.91468  1.34888
C  -2.23651 -2.16359  2.50408
H  -2.27652 -1.20258  3.01538
C   1.28173  3.20664  2.36650
H   1.37763  2.89624  3.40582
C   2.22057  4.06682  1.80937
H   3.03907  4.44199  2.41999
C   6.27857 -0.01906 -1.16728
H   6.83589 -0.24186 -2.07455
C   4.86666  0.55243  1.17185
H   4.29219  0.77066  2.06915
C   2.12701  4.43231  0.47015
H   2.87150  5.09423  0.03319
C   4.89186 -0.05990 -1.17486
H   4.33648 -0.31328 -2.07486
C   6.95684  0.30892  0.00439
H   8.04468  0.34168  0.00743
C   6.25338  0.59524  1.17208
H   6.79124  0.85117  2.08235
```

**25⁺**

```
Cu  0.44159 -0.73635 -0.94710
C   1.39774  1.06518  1.31497
C   0.78437  2.24624  2.09943
C  -0.57142  1.64810  0.43502
N   0.36933  0.78222  0.31389
H   1.38853  3.15720  2.04953
O  -0.45515  2.50625  1.42055
C  -2.28293 -1.30112 -2.29839
C  -3.46706 -0.40021 -2.68554
C  -2.14860  0.67033 -1.24873
N  -1.45522 -0.37288 -1.50164
H  -4.44310 -0.85932 -2.51411
O  -3.34364  0.73765 -1.80791
C  -1.73347  1.89042 -0.48342
C  -2.92491  2.47587  0.28049
H  -2.57433  3.38710  0.77840
C  -1.23371  2.89855 -1.59035
H  -2.08981  3.05811 -2.25628
H  -0.45609  2.38152 -2.16724
H  -3.66687  2.78865 -0.46320
H  -1.70458 -1.62029 -3.16821
H  -3.40805 -0.02979 -3.71454
H   1.50167  0.18887  1.96625
H   0.54850  2.00742  3.13995
C   2.74198  1.35944  0.69256
C   2.93418  1.40226 -0.68711
C   3.83182  1.59256  1.53163
C   4.19010  1.67427 -1.22142
```

```
H   2.09514  1.23573 -1.36118
C   5.08211  1.88061  1.00061
H   3.70705  1.53503  2.61257
C   5.26623  1.92000 -0.37834
C  -2.64890 -2.51539 -1.48733
C  -2.07362 -3.75243 -1.77079
C  -3.54085 -2.39667 -0.41988
C  -2.38865 -4.86178 -0.99178
H  -1.35802 -3.83491 -2.58577
C  -3.85153 -3.50352  0.35943
H  -3.99414 -1.43260 -0.18910
C  -3.27360 -4.73853  0.07433
C  -3.58668  1.57521  1.29176
C  -3.04384  0.37113  1.74219
C  -4.80821  1.99282  1.83080
C  -3.70061 -0.38661  2.71081
H  -2.11042 -0.02157  1.33921
C  -5.46481  1.23857  2.79129
H  -5.24882  2.92829  1.48807
C  -4.90947  0.04249  3.23906
H  -3.25335 -1.31841  3.04813
H  -6.41454  1.58451  3.19391
C  -0.70936  4.20510 -1.07367
C   0.64897  4.35263 -0.78484
C  -1.56337  5.28805 -0.85981
C   1.14110  5.54671 -0.27306
H   1.33118  3.52248 -0.96410
C  -1.07374  6.48479 -0.34876
H  -2.62107  5.19675 -1.10368
C   0.27805  6.61510 -0.04958
H   2.20368  5.64630 -0.06008
H  -1.75022  7.32205 -0.19088
H  -3.51724 -5.60751  0.68209
H   6.24736  2.14022 -0.79327
H  -5.41968 -0.54993  3.99548
H   0.66095  7.55352  0.34577
C  -0.37590 -2.81610  1.35499
C  -0.98596 -3.02864  2.58728
C   0.96029 -2.44019  1.36231
C  -0.28878 -2.84853  3.78543
C   1.68645 -2.28127  2.55685
C   1.04939 -2.47502  3.78134
C   3.08469 -1.91477  0.77219
C   3.04922 -1.95411  2.17796
H  -0.91985 -2.96712  0.42673
H  -2.02139 -3.35897  2.60817
H  -0.79673 -3.02040  4.73174
H   1.59447 -2.35649  4.71550
C   4.26804 -1.70495  0.07664
C   4.21903 -1.74579  2.90782
C   5.40358 -1.52768  2.21854
C   5.42580 -1.51588  0.81995
H   4.29298 -1.69434 -1.00807
H   4.20747 -1.77841  3.99537
H   6.32884 -1.37647  2.76999
H   6.36741 -1.35304  0.30038
N   1.78848 -2.13086  0.26097
H   1.67379 -2.63676 -0.62659
O   0.74869 -2.04807 -2.23143
C   1.27825 -1.69596 -3.48739
C   2.80332 -1.66865 -3.41030
H   3.26235 -1.45721 -4.38339
H   3.12655 -0.88820 -2.70824
H   3.18152 -2.63506 -3.05559
C   0.81556 -2.75230 -4.48647
H   1.23530 -2.58041 -5.48447
H  -0.27868 -2.74880 -4.57019
H   1.12553 -3.74607 -4.14456
H   5.92193  2.06114  1.66817
H   4.32179  1.70157 -2.30132
H  -4.54850 -3.40047  1.18923
H  -1.93720 -5.82577 -1.21606
C   0.78868 -0.31229 -3.92578
H   1.09959  0.45565 -3.19960
H   1.21480 -0.02834 -4.89486
H  -0.30273 -0.27557 -4.01395
```

**TS-4**

```
Cu -0.21608 -0.81736  0.79316
C  -1.74533  0.84286 -1.22010
C  -1.47037  2.16423 -1.97100
C   0.10111  1.82044 -0.42342
N  -0.62252  0.77095 -0.28650
H  -2.24762  2.91767 -1.81067
O  -0.25876  2.65629 -1.37391
C   2.58668 -0.71812  2.14083
C   3.49986  0.44377  2.56965
C   1.91863  1.19257  1.19156
N   1.52996 -0.00886  1.39030
H   4.55556  0.26991  2.34891
O   3.05105  1.55413  1.77032
```

| | | | |
|---|---|---|---|
| C | 1.23058 | 2.29441 | 0.44304 |
| C | 2.27653 | 3.07165 | -0.38664 |
| H | 1.73464 | 3.79226 | -1.00822 |
| C | 0.59324 | 3.21225 | 1.55591 |
| H | 1.43497 | 3.58354 | 2.15199 |
| H | -0.01048 | 2.56321 | 2.20314 |
| H | 2.88637 | 3.64596 | 0.31749 |
| H | 2.13173 | -1.21719 | 3.00004 |
| H | 3.38155 | 0.72290 | 3.62198 |
| H | -1.68250 | -0.01302 | -1.90450 |
| H | -1.28762 | 2.03577 | -3.04127 |
| C | -3.08275 | 0.79834 | -0.52158 |
| C | -3.20167 | 0.75958 | 0.86662 |
| C | -4.24052 | 0.79722 | -1.29929 |
| C | -4.45588 | 0.72355 | 1.46851 |
| H | -2.30861 | 0.76581 | 1.48918 |
| C | -5.49272 | 0.77863 | -0.69951 |
| H | -4.16466 | 0.79529 | -2.38630 |
| C | -5.60460 | 0.73877 | 0.68687 |
| C | 3.25439 | -1.76039 | 1.28368 |
| C | 3.10830 | -3.11299 | 1.58244 |
| C | 4.00208 | -1.37759 | 0.16858 |
| C | 3.70234 | -4.07749 | 0.77514 |
| H | 2.50585 | -3.41277 | 2.43633 |
| C | 4.60421 | -2.34108 | -0.62980 |
| H | 4.11344 | -0.32362 | -0.08740 |
| C | 4.45163 | -3.69267 | -0.33069 |
| C | 3.18381 | 2.20521 | -1.22184 |
| C | 2.69628 | 1.32957 | -2.19682 |
| C | 4.56532 | 2.27661 | -1.02689 |
| C | 3.56743 | 0.56300 | -2.96344 |
| H | 1.62919 | 1.25267 | -2.39282 |
| C | 5.43917 | 1.51470 | -1.79481 |
| H | 4.96034 | 2.94645 | -0.26445 |
| C | 4.94129 | 0.65727 | -2.77016 |
| H | 3.16476 | -0.10146 | -3.72441 |
| H | 6.51208 | 1.59584 | -1.63327 |
| C | -0.24877 | 4.34387 | 1.04679 |
| C | -1.63139 | 4.18724 | 0.92868 |
| C | 0.32625 | 5.55589 | 0.66225 |
| C | -2.42108 | 5.20879 | 0.41519 |
| H | -2.09714 | 3.25382 | 1.24291 |
| C | -0.46153 | 6.58014 | 0.14935 |
| H | 1.39945 | 5.70437 | 0.77324 |
| C | -1.83539 | 6.40704 | 0.01888 |
| H | -3.49784 | 5.07182 | 0.33602 |
| H | -0.00083 | 7.52142 | -0.14283 |
| H | 4.91917 | -4.44641 | -0.96075 |
| H | -6.58578 | 0.71752 | 1.15608 |
| H | 5.62227 | 0.06834 | -3.38141 |
| H | -2.45093 | 7.21133 | -0.37835 |
| C | 0.91646 | -2.49963 | -1.32679 |
| C | 1.55234 | -2.49186 | -2.56413 |
| C | -0.47663 | -2.46462 | -1.31346 |
| C | 0.82344 | -2.45606 | -3.75588 |
| C | -1.22275 | -2.45070 | -2.51202 |
| C | -0.56768 | -2.44320 | -3.74021 |
| C | -2.62938 | -2.45077 | -0.70069 |
| C | -2.61588 | -2.46160 | -2.11497 |
| H | 1.48973 | -2.56530 | -0.40536 |
| H | 2.63748 | -2.52967 | -2.59679 |
| H | 1.35086 | -2.46112 | -4.70752 |
| H | -1.13150 | -2.44024 | -4.67111 |
| C | -3.83607 | -2.52108 | -0.00541 |
| C | -3.80792 | -2.51716 | -2.83338 |
| C | -5.00641 | -2.57913 | -2.13473 |
| C | -5.01375 | -2.58726 | -0.73603 |
| H | -3.86532 | -2.53096 | 1.07729 |
| H | -3.79887 | -2.53593 | -3.92154 |
| H | -5.94727 | -2.63561 | -2.67769 |
| H | -5.96181 | -2.64583 | -0.20587 |
| N | -1.32541 | -2.41209 | -0.19650 |
| H | -0.91488 | -2.71104 | 0.98486 |
| O | -0.24536 | -2.38173 | 1.95545 |
| C | -0.78120 | -2.37763 | 3.28168 |
| C | -2.25957 | -2.73582 | 3.23752 |
| H | -2.67051 | -2.83174 | 4.24853 |
| H | -2.82180 | -1.94744 | 2.72245 |
| H | -2.41764 | -3.68593 | 2.71358 |
| C | -0.00054 | -3.40254 | 4.09197 |
| H | -0.39503 | -3.48805 | 5.11086 |
| H | 1.05599 | -3.11728 | 4.16140 |
| H | -0.05901 | -4.38563 | 3.61243 |
| H | -6.38693 | 0.77880 | -1.31908 |
| H | -4.53283 | 0.69184 | 2.55368 |
| H | 5.19041 | -2.03312 | -1.49282 |
| H | 3.57832 | -5.13206 | 1.01100 |
| C | -0.63084 | -0.98486 | 3.88614 |
| H | -1.16227 | -0.24160 | 3.27352 |
| H | -1.06075 | -0.94765 | 4.89335 |
| H | 0.41863 | -0.68228 | 3.96121 |

**26⁺**

| | | | |
|---|---|---|---|
| Cu | 0.67456 | -0.36550 | 0.49026 |
| C | -0.38868 | -0.99053 | 3.22786 |
| C | -1.62823 | -0.56746 | 4.03361 |
| C | -1.93610 | -0.14066 | 1.85807 |
| N | -0.79570 | -0.71455 | 1.83479 |
| H | -1.42168 | 0.20818 | 4.77711 |
| O | -2.51772 | -0.01808 | 3.04670 |
| C | -0.48699 | 0.80494 | -2.19851 |
| C | -1.91226 | 0.94342 | -2.76206 |
| C | -1.98680 | 0.50196 | -0.57652 |
| N | -0.72853 | 0.46111 | -0.78538 |
| H | -2.16692 | 0.19629 | -3.51899 |
| O | -2.76314 | 0.73622 | -1.62448 |
| C | -2.70019 | 0.51678 | 0.74594 |
| C | -4.10735 | -0.10885 | 0.62720 |
| H | -4.59997 | 0.02935 | 1.59625 |
| C | -2.80332 | 2.02911 | 1.18615 |
| H | -1.81162 | 2.32944 | 1.54894 |
| H | -3.47994 | 2.04883 | 2.04675 |
| H | -4.66015 | 0.47447 | -0.11538 |
| H | 0.02258 | 1.77877 | -2.19357 |
| H | -2.12226 | 1.94187 | -3.15403 |
| H | -0.19493 | -2.06164 | 3.33830 |
| H | -2.13470 | -1.40795 | 4.51685 |
| C | 0.89094 | -0.23543 | 3.48127 |
| C | 0.88693 | 1.16070 | 3.49515 |
| C | 2.10530 | -0.91485 | 3.54890 |
| C | 2.08024 | 1.86577 | 3.56909 |
| H | -0.05808 | 1.70379 | 3.43065 |
| C | 3.30170 | -0.20964 | 3.61991 |
| H | 2.11486 | -2.00390 | 3.53862 |
| C | 3.29034 | 1.17998 | 3.62325 |
| C | 0.43144 | -0.18762 | -2.86446 |
| C | -0.02894 | -1.37588 | -3.42954 |
| C | 1.80512 | 0.05755 | -2.83276 |
| C | 0.87062 | -2.30076 | -3.95124 |
| H | -1.09359 | -1.60306 | -3.44775 |
| C | 2.70973 | -0.87933 | -3.32020 |
| H | 2.17213 | 0.99185 | -2.41315 |
| C | 2.24135 | -2.06179 | -3.88676 |
| C | -4.10548 | -1.56532 | 0.24993 |
| C | -4.30284 | -1.96387 | -1.07421 |
| C | -3.92841 | -2.55140 | 1.22517 |
| C | -4.33458 | -3.31266 | -1.41289 |
| H | -4.45821 | -1.20512 | -1.83877 |
| C | -3.95757 | -3.89979 | 0.88858 |
| H | -3.80715 | -2.25712 | 2.26737 |
| C | -4.16613 | -4.28373 | -0.43217 |
| H | -4.51027 | -3.60650 | -2.44591 |
| H | -3.83804 | -4.65430 | 1.66370 |
| C | -3.25214 | 2.98991 | 0.12594 |
| C | -4.60603 | 3.24592 | -0.09302 |
| C | -2.30828 | 3.63530 | -0.67756 |
| C | -5.00863 | 4.11035 | -1.10529 |
| H | -5.35390 | 2.77257 | 0.54183 |
| C | -2.70767 | 4.50129 | -1.68832 |
| H | -1.24666 | 3.45619 | -0.50932 |
| C | -4.06166 | 4.73607 | -1.90861 |
| H | -6.06799 | 4.30280 | -1.26149 |
| H | -1.95907 | 5.00358 | -2.29845 |
| H | 2.94235 | -2.79339 | -4.28325 |
| H | 4.22629 | 1.73246 | 3.66775 |
| H | -4.20564 | -5.33895 | -0.69480 |
| H | -4.37785 | 5.41729 | -2.69571 |
| C | 4.04255 | -1.23961 | 0.05884 |
| C | 5.30394 | -1.51718 | -0.45448 |
| C | 3.56327 | 0.06770 | -0.04370 |
| C | 6.08332 | -0.51480 | -1.04098 |
| C | 4.36148 | 1.09236 | -0.61537 |
| C | 5.62185 | 0.79965 | -1.11751 |
| C | 2.35203 | 1.90927 | 0.08885 |
| C | 3.56745 | 2.30478 | -0.52107 |
| H | 3.43509 | -2.00999 | 0.52400 |
| H | 5.69820 | -2.52930 | -0.38978 |
| H | 7.06931 | -0.76129 | -1.42887 |
| H | 6.24309 | 1.57680 | -1.55853 |
| C | 1.34128 | 2.83677 | 0.34088 |
| C | 3.76362 | 3.62994 | -0.88839 |
| C | 2.74555 | 4.55131 | -0.64464 |
| C | 1.54853 | 4.15837 | -0.03597 |
| H | 0.42431 | 2.51580 | 0.83012 |
| H | 4.69288 | 3.94893 | -1.35635 |
| H | 2.88617 | 5.59312 | -0.92392 |
| H | 0.77591 | 4.90097 | 0.15514 |
| N | 2.33973 | 0.55587 | 0.37051 |
| H | 1.51068 | -2.17781 | -1.28621 |
| O | 1.13981 | -2.29492 | -0.40189 |
| C | 0.61744 | -3.63559 | -0.26932 |
| C | -0.74194 | -3.71446 | -0.94157 |
| H | -0.65504 | -3.64193 | -2.02934 |

```
H  -1.23553 -4.66301 -0.70593
H  -1.38245 -2.89827 -0.58354
C   1.61978 -4.59125 -0.89430
H   2.58601 -4.53537 -0.37948
H   1.77645 -4.34747 -1.95292
H   1.25775 -5.62365 -0.83964
H   2.06950  2.95347  3.56868
H   4.24632 -0.74738  3.66330
H   3.77816 -0.68025 -3.25404
H   0.49967 -3.22055 -4.39962
C   0.47560 -3.88941  1.21884
H   0.22455 -4.93942  1.40119
H   1.41187 -3.66098  1.74141
H  -0.33188 -3.27534  1.62909
```

**27⁺**

```
C  -1.56901 -2.15040 -1.66862
C  -2.21126 -2.77157 -2.73536
C  -2.35629 -1.47123 -0.74052
C  -3.60145 -2.71692 -2.86526
C  -3.76526 -1.40827 -0.86756
C  -4.39006 -2.03562 -1.93310
C  -3.03995 -0.21043  0.94163
C  -4.21747 -0.58145  0.24214
H  -0.48636 -2.18861 -1.55364
H  -1.62645 -3.31276 -3.47653
H  -4.07857 -3.21432 -3.70684
H  -5.47091 -1.99751 -2.05290
C  -3.09067  0.60280  2.07392
C  -5.44899 -0.11848  0.67154
C  -5.49567  0.71195  1.79583
C  -4.33365  1.06594  2.48738
H  -2.17809  0.86198  2.60808
H  -6.36519 -0.38880  0.15047
H  -6.45683  1.08301  2.14518
H  -4.40744  1.70532  3.36421
N  -1.92074 -0.74904  0.35363
Cu -0.15032 -0.15210  0.49672
C  -0.08328  1.24958 -2.21850
C   0.92160  2.19766 -2.89780
C   1.91275  1.10136 -1.23409
N   0.70477  0.72331 -1.10082
H   0.72420  3.24822 -2.65314
O   2.18683  1.84126 -2.30453
C   1.76669 -0.97442  2.61648
C   3.26353 -1.31976  2.56818
C   2.74587  0.06045  0.89362
N   1.60021 -0.13306  1.42132
H   3.44742 -2.35369  2.25258
O   3.78529 -0.45195  1.54250
C   3.09639  0.80412 -0.36175
C   4.10117 -0.06632 -1.17305
H   4.25880  0.43860 -2.13315
C   3.76389  2.16045  0.02212
H   4.28937  2.51970 -0.86875
H   4.52026  1.94623  0.78669
H   5.05216 -0.05421 -0.63146
H   1.52329 -0.37238  3.50157
H   3.79930 -1.11513  3.49740
H  -0.34776  0.40693 -2.87357
H   1.00056  2.07406 -3.97982
C  -1.34945  1.88734 -1.71274
C  -2.58179  1.58991 -2.28705
C  -1.28932  2.76002 -0.62306
C  -3.74413  2.16750 -1.78484
H  -2.63756  0.88525 -3.11662
C  -2.45027  3.33579 -0.12398
H  -0.32872  2.98278 -0.15764
C  -3.68077  3.04073 -0.70510
C   0.78030 -2.10784  2.50284
C  -0.28524 -2.21347  3.39167
C   0.83537 -2.95294  1.39001
C  -1.29044 -3.15045  3.17236
H  -0.34081 -1.54644  4.25120
C  -0.16328 -3.89371  1.17724
H   1.64907 -2.85702  0.67009
C  -1.23243 -3.98777  2.06464
C   3.64109 -1.48567 -1.37315
C   4.23852 -2.52898 -0.66052
C   2.60790 -1.79646 -2.26275
C   3.81398 -3.84365 -0.82377
H   5.05737 -2.30347  0.02093
C   2.17433 -3.10851 -2.42076
H   2.15989 -1.01032 -2.86883
C   2.77334 -4.13634 -1.69899
H   4.30283 -4.64287 -0.27044
H   1.37966 -3.33277 -3.13006
C   2.78464  3.20264  0.49039
C   2.20566  3.14464  1.76114
C   2.41935  4.25038 -0.35944
C   1.27955  4.10044  2.16545
```

| | | | |
|---|---|---|---|
| H | 2.50145 | 2.36068 | 2.45621 |
| C | 1.50156 | 5.21363 | 0.04560 |
| H | 2.87458 | 4.31522 | -1.34668 |
| C | 0.92439 | 5.13758 | 1.30875 |
| H | 0.84737 | 4.04539 | 3.16263 |
| H | 1.24171 | 6.02998 | -0.62513 |
| H | -2.02284 | -4.71428 | 1.88923 |
| H | -4.59184 | 3.48374 | -0.30855 |
| H | 2.44256 | -5.16417 | -1.83268 |
| H | 0.20974 | 5.89241 | 1.63032 |
| H | -0.11590 | -4.54285 | 0.30500 |
| H | -2.12516 | -3.22106 | 3.86629 |
| H | -4.70498 | 1.92310 | -2.23279 |
| H | -2.39402 | 4.01233 | 0.72658 |

**28⁺**

| | | | |
|---|---|---|---|
| Cu | -0.11613 | 0.50606 | -0.49993 |
| C | 0.26429 | -1.06858 | -3.12362 |
| C | 0.28476 | -2.56619 | -3.46437 |
| C | -0.10820 | -2.29133 | -1.29275 |
| N | -0.15629 | -1.08454 | -1.71056 |
| H | 1.18141 | -2.89251 | -3.99581 |
| O | 0.25303 | -3.20901 | -2.17981 |
| C | -1.64032 | -0.01017 | 2.11963 |
| C | -2.06494 | -1.28245 | 2.88801 |
| C | -0.98163 | -1.83780 | 1.01825 |
| N | -0.89303 | -0.56554 | 0.99350 |
| H | -3.14574 | -1.36112 | 3.03605 |
| O | -1.64937 | -2.36194 | 2.03306 |
| C | -0.31971 | -2.82465 | 0.09568 |
| C | -1.16232 | -4.12262 | 0.00570 |
| H | -0.53413 | -4.88328 | -0.46778 |
| C | 1.12907 | -3.08797 | 0.65581 |
| H | 1.75599 | -2.25655 | 0.31302 |
| H | 1.48835 | -4.00706 | 0.17929 |
| H | -1.37885 | -4.45308 | 1.02632 |
| H | -0.95850 | 0.59449 | 2.72895 |
| H | -1.54475 | -1.40297 | 3.84280 |
| H | -0.50015 | -0.54551 | -3.70837 |
| H | -0.61082 | -2.88390 | -4.00991 |
| C | 1.57738 | -0.34018 | -3.26423 |
| C | 1.60494 | 0.96710 | -3.74785 |
| C | 2.77310 | -0.95179 | -2.88108 |
| C | 2.81185 | 1.65025 | -3.86411 |
| H | 0.67761 | 1.44969 | -4.05540 |
| C | 3.97708 | -0.26992 | -2.99426 |
| H | 2.76329 | -1.96675 | -2.48244 |
| C | 3.99772 | 1.03194 | -3.48877 |
| C | -2.76233 | 0.87272 | 1.63478 |
| C | -3.47172 | 0.54693 | 0.47728 |
| C | -3.10205 | 2.02371 | 2.34269 |
| C | -4.50633 | 1.36280 | 0.03521 |
| H | -3.20385 | -0.33953 | -0.09635 |
| C | -4.14436 | 2.83301 | 1.90592 |
| H | -2.52467 | 2.30538 | 3.22377 |
| C | -4.84713 | 2.50508 | 0.75134 |
| C | -2.42872 | -3.94921 | -0.79756 |
| C | -2.49326 | -4.42789 | -2.10961 |
| C | -3.54839 | -3.29005 | -0.28066 |
| C | -3.62753 | -4.23324 | -2.89006 |
| H | -1.64330 | -4.97243 | -2.51768 |
| C | -4.68129 | -3.08718 | -1.06171 |
| H | -3.54642 | -2.95024 | 0.75130 |
| C | -4.72273 | -3.55097 | -2.37165 |
| H | -3.65965 | -4.62658 | -3.90435 |
| H | -5.54183 | -2.57407 | -0.63649 |
| C | 1.20850 | -3.16797 | 2.15118 |
| C | 0.94832 | -4.35337 | 2.83934 |
| C | 1.49594 | -2.00998 | 2.88009 |
| C | 0.95971 | -4.37844 | 4.22925 |
| H | 0.73891 | -5.26635 | 2.28325 |
| C | 1.50932 | -2.03488 | 4.27029 |
| H | 1.71152 | -1.08690 | 2.34152 |
| C | 1.23413 | -3.21898 | 4.94776 |
| H | 0.75830 | -5.30951 | 4.75493 |
| H | 1.74614 | -1.13007 | 4.82718 |
| H | -5.65253 | 3.14767 | 0.40203 |
| H | 4.94279 | 1.56372 | -3.57687 |
| H | -5.61184 | -3.39828 | -2.97980 |
| H | 1.24746 | -3.24252 | 6.03544 |
| C | 0.71229 | 2.70235 | 2.03300 |
| C | 0.70199 | 3.51368 | 3.16364 |
| C | -0.10881 | 3.05837 | 0.96383 |
| C | -0.10201 | 4.65509 | 3.22470 |
| C | -0.93757 | 4.20380 | 1.02794 |
| C | -0.92856 | 5.00829 | 2.15806 |
| C | -1.27872 | 3.06197 | -0.91991 |
| C | -1.70851 | 4.20274 | -0.20152 |
| H | 1.32315 | 1.80326 | 1.98981 |
| H | 1.33382 | 3.25805 | 4.01159 |
| H | -0.08375 | 5.27439 | 4.11877 |

| | | | |
|---|---|---|---|
| H | -1.56156 | 5.89127 | 2.22012 |
| C | -1.84035 | 2.72952 | -2.15248 |
| C | -2.70165 | 5.01806 | -0.72397 |
| C | -3.27334 | 4.67478 | -1.94913 |
| C | -2.85218 | 3.54115 | -2.65081 |
| H | -1.49628 | 1.85342 | -2.70085 |
| H | -3.03625 | 5.90492 | -0.18956 |
| H | -4.05593 | 5.30198 | -2.37046 |
| H | -3.31460 | 3.30035 | -3.60548 |
| N | -0.28132 | 2.38257 | -0.23881 |
| C | 4.72203 | -0.41036 | 0.68933 |
| C | 5.31646 | 1.79461 | -0.11928 |
| C | 6.65306 | 1.52238 | 0.13687 |
| C | 7.02381 | 0.28876 | 0.66601 |
| C | 6.05898 | -0.67653 | 0.94480 |
| H | 3.95344 | -1.14978 | 0.90203 |
| H | 5.01117 | 2.75162 | -0.53434 |
| H | 7.41044 | 2.27379 | -0.07493 |
| H | 8.07281 | 0.07888 | 0.86550 |
| H | 6.35281 | -1.63643 | 1.36351 |
| C | 4.34880 | 0.82485 | 0.15408 |
| C | 2.92559 | 1.06947 | -0.13406 |
| O | 2.65404 | 2.25763 | -0.62285 |
| O | 2.05226 | 0.23326 | 0.07239 |
| H | 1.68440 | 2.33587 | -0.75476 |
| H | 2.82381 | 2.66722 | -4.24997 |
| H | 4.90379 | -0.74973 | -2.68652 |
| H | -5.04408 | 1.10788 | -0.87561 |
| H | -4.39607 | 3.73432 | 2.46120 |

**TS-5**

| | | | |
|---|---|---|---|
| Cu | -0.39607 | -0.35474 | -0.48825 |
| C | -0.58523 | 2.04652 | -2.47295 |
| C | -0.18783 | 3.52497 | -2.33154 |
| C | 0.29569 | 2.44486 | -0.45430 |
| N | -0.04758 | 1.47785 | -1.21876 |
| H | -0.98908 | 4.22547 | -2.57561 |
| O | 0.12952 | 3.66269 | -0.93492 |
| C | 2.09854 | -1.07824 | 1.37569 |
| C | 2.60293 | -0.29477 | 2.59613 |
| C | 1.41938 | 1.03486 | 1.27479 |
| N | 1.16147 | -0.11928 | 0.77980 |
| H | 3.67718 | -0.38258 | 2.77174 |
| O | 2.30701 | 1.06893 | 2.25041 |
| C | 0.76463 | 2.35600 | 0.96869 |
| C | 1.74823 | 3.51712 | 1.25859 |
| H | 1.16140 | 4.44072 | 1.25234 |
| C | -0.54492 | 2.44141 | 1.85335 |
| H | -1.36056 | 1.99310 | 1.27156 |
| H | -0.76960 | 3.50609 | 1.98293 |
| H | 2.13558 | 3.37913 | 2.27299 |
| H | 1.56311 | -1.97367 | 1.70595 |
| H | 2.04151 | -0.52296 | 3.50943 |
| H | -0.06485 | 1.57823 | -3.31512 |
| H | 0.71702 | 3.77791 | -2.89511 |
| C | -2.06415 | 1.78493 | -2.59039 |
| C | -2.54104 | 0.80636 | -3.45818 |
| C | -2.96991 | 2.49657 | -1.79987 |
| C | -3.90442 | 0.53490 | -3.53559 |
| H | -1.84518 | 0.26079 | -4.09475 |
| C | -4.32952 | 2.23714 | -1.88594 |
| H | -2.60875 | 3.25623 | -1.10550 |
| C | -4.79887 | 1.25228 | -2.75207 |
| C | 3.18288 | -1.46087 | 0.39250 |
| C | 3.37048 | -0.77376 | -0.80574 |
| C | 4.04060 | -2.51575 | 0.71218 |
| C | 4.41098 | -1.12313 | -1.66160 |
| H | 2.70169 | 0.03851 | -1.08125 |
| C | 5.07796 | -2.86419 | -0.14272 |
| H | 3.89111 | -3.07054 | 1.63835 |
| C | 5.26835 | -2.16482 | -1.33199 |
| C | 2.86977 | 3.64446 | 0.25196 |
| C | 2.87359 | 4.72482 | -0.63607 |
| C | 3.90537 | 2.71030 | 0.15359 |
| C | 3.86266 | 4.85962 | -1.60267 |
| H | 2.08335 | 5.47046 | -0.56552 |
| C | 4.89033 | 2.83555 | -0.82120 |
| H | 3.96455 | 1.88148 | 0.85199 |
| C | 4.87134 | 3.90705 | -1.70559 |
| H | 3.84792 | 5.71526 | -2.27500 |
| H | 5.68206 | 2.09082 | -0.87850 |
| C | -0.45366 | 1.72796 | 3.17088 |
| C | 0.15781 | 2.30631 | 4.28441 |
| C | -0.94125 | 0.42182 | 3.26901 |
| C | 0.29045 | 1.58800 | 5.46708 |
| H | 0.53147 | 3.32788 | 4.22816 |
| C | -0.81515 | -0.29552 | 4.45365 |
| H | -1.43157 | -0.02641 | 2.40547 |
| C | -0.19289 | 0.28514 | 5.55400 |
| H | 0.76599 | 2.05053 | 6.32942 |
| H | -1.20964 | -1.30765 | 4.51833 |

```
H   6.08252 -2.43706 -2.00034
H  -5.86470  1.04189 -2.80767
H   5.64614  4.00814 -2.46271
H  -0.09559 -0.27069  6.48429
C  -0.94340 -3.12619  1.57553
C  -0.41601 -3.94913  2.56554
C  -0.30886 -3.12146  0.33886
C   0.70800 -4.74641  2.32136
C   0.81143 -3.93185  0.07247
C   1.32501 -4.75134  1.07449
C   0.32261 -2.65819 -1.76474
C   1.21813 -3.63716 -1.28865
H  -1.81731 -2.50599  1.76409
H  -0.89029 -3.98081  3.54424
H   1.09438 -5.38085  3.11593
H   2.19392 -5.37912  0.88787
C   0.43696 -2.11758 -3.04221
C   2.24749 -4.08683 -2.10715
C   2.37008 -3.54433 -3.38282
C   1.47863 -2.57149 -3.84401
H  -0.27617 -1.37904 -3.40314
H   2.95079 -4.83858 -1.75573
H   3.17219 -3.88323 -4.03451
H   1.59372 -2.17333 -4.84971
N  -0.61652 -2.32046 -0.77668
C  -4.83771  0.49601  0.97903
C  -5.67360 -1.39232 -0.28265
C  -6.96444 -1.09422  0.13266
C  -7.19126 -0.00611  0.97136
C  -6.12778  0.78579  1.39904
H  -3.99472  1.10711  1.29249
H  -5.47043 -2.23784 -0.93513
H  -7.79746 -1.71270 -0.19442
H  -8.20351  0.22428  1.29796
H  -6.30959  1.63005  2.06068
C  -4.60923 -0.59222  0.13480
C  -3.23922 -0.89519 -0.33353
O  -3.04962 -1.93545 -1.02346
O  -2.30290 -0.10878 -0.00695
H  -1.83962 -2.18232 -1.02073
H  -4.26561 -0.23457 -4.21409
H  -5.02962  2.79229 -1.26588
H   4.54601 -0.57627 -2.59253
H   5.74282 -3.68465  0.11998
```

**29$^+$**

```
Cu -0.39628  0.37430  0.47603
C  -0.65634 -1.99277  2.49882
C  -0.30841 -3.48563  2.36639
C   0.19015 -2.43956  0.47429
N  -0.11222 -1.45486  1.23351
H  -1.13317 -4.15476  2.61998
O  -0.00564 -3.64604  0.96831
C   2.11968  0.99504 -1.39825
C   2.54404  0.19102 -2.63592
C   1.34258 -1.08471 -1.27788
N   1.16009  0.07955 -0.76929
H   3.61477  0.22666 -2.84754
O   2.19337 -1.15704 -2.28182
C   0.63380 -2.37375 -0.95723
C   1.55629 -3.57835 -1.26855
H   0.92922 -4.47460 -1.23653
C  -0.70019 -2.39733 -1.81179
H  -1.48355 -1.92227 -1.20640
H  -0.96949 -3.45125 -1.94418
H   1.91743 -3.46411 -2.29556
H   1.61508  1.91309 -1.71275
H   1.96353  0.44811 -3.52955
H  -0.11233 -1.53452  3.33141
H   0.59098 -3.76622  2.92549
C  -2.12549 -1.69198  2.63361
C  -2.57265 -0.74513  3.54981
C  -3.05244 -2.34885  1.82024
C  -3.92948 -0.45044  3.65300
H  -1.85844 -0.23897  4.19865
C  -4.40484 -2.06471  1.92956
H  -2.71185 -3.08234  1.08786
C  -4.84506 -1.11114  2.84530
C   3.25232  1.33063 -0.45392
C   3.45528  0.63003  0.73398
C   4.13869  2.35275 -0.80068
C   4.53742  0.93557  1.55424
H   2.76846 -0.16031  1.02838
C   5.21832  2.65657  0.01844
H   3.97993  2.91580 -1.72033
C   5.42204  1.94552  1.19846
C   2.70206 -3.74864 -0.29578
C   2.68521 -4.82035  0.60251
C   3.78207 -2.86235 -0.23957
C   3.69790 -4.99211  1.53836
H   1.85984 -5.52934  0.56464
```

| | | | |
|---|---|---|---|
| C | 4.79152 | -3.02462 | 0.70419 |
| H | 3.85739 | -2.04347 | -0.94799 |
| C | 4.75203 | -4.08637 | 1.59948 |
| H | 3.66558 | -5.84031 | 2.21941 |
| H | 5.61893 | -2.31787 | 0.72787 |
| C | -0.61251 | -1.67719 | -3.12589 |
| C | -0.04858 | -2.26979 | -4.25704 |
| C | -1.05280 | -0.35288 | -3.20365 |
| C | 0.08447 | -1.54811 | -5.43749 |
| H | 0.28762 | -3.30505 | -4.21600 |
| C | -0.92617 | 0.36794 | -4.38643 |
| H | -1.51002 | 0.10595 | -2.32743 |
| C | -0.35115 | -0.22719 | -5.50452 |
| H | 0.52219 | -2.02171 | -6.31375 |
| H | -1.28520 | 1.39407 | -4.43574 |
| H | 6.26896 | 2.18294 | 1.83877 |
| H | -5.90539 | -0.88019 | 2.91948 |
| H | 5.54563 | -4.21695 | 2.33220 |
| H | -0.25452 | 0.33140 | -6.43324 |
| C | -0.87235 | 3.21302 | -1.49858 |
| C | -0.33777 | 4.00229 | -2.51242 |
| C | -0.18392 | 3.17589 | -0.29506 |
| C | 0.84372 | 4.72651 | -2.31846 |
| C | 0.99188 | 3.90997 | -0.07327 |
| C | 1.51345 | 4.69407 | -1.10018 |
| C | 0.50968 | 2.69024 | 1.80815 |
| C | 1.43274 | 3.60567 | 1.27719 |
| H | -1.79157 | 2.64889 | -1.64178 |
| H | -0.85130 | 4.06337 | -3.46957 |
| H | 1.23487 | 5.33465 | -3.13105 |
| H | 2.42683 | 5.26634 | -0.95247 |
| C | 0.62913 | 2.17303 | 3.09037 |
| C | 2.51280 | 4.01799 | 2.05138 |
| C | 2.64441 | 3.50383 | 3.33616 |
| C | 1.71612 | 2.59386 | 3.84976 |
| H | -0.10812 | 1.47849 | 3.48795 |
| H | 3.24139 | 4.72382 | 1.65945 |
| H | 3.48300 | 3.81504 | 3.95483 |
| H | 1.83960 | 2.21616 | 4.86226 |
| N | -0.47870 | 2.38161 | 0.84101 |
| C | -4.74583 | -0.34568 | -1.02241 |
| C | -5.65256 | 1.45648 | 0.30873 |
| C | -6.92192 | 1.17620 | -0.17954 |
| C | -7.10332 | 0.13843 | -1.09000 |
| C | -6.01467 | -0.61938 | -1.51488 |
| H | -3.88632 | -0.93344 | -1.33659 |
| H | -5.48041 | 2.26140 | 1.01930 |
| H | -7.77384 | 1.76888 | 0.14721 |
| H | -8.09825 | -0.07910 | -1.47373 |
| H | -6.15927 | -1.42527 | -2.23153 |
| C | -4.56251 | 0.69227 | -0.10734 |
| C | -3.21705 | 0.99637 | 0.45100 |
| O | -3.05290 | 1.96607 | 1.19871 |
| O | -2.26723 | 0.20567 | 0.08572 |
| H | -1.50240 | 2.35301 | 1.14205 |
| H | -4.26839 | 0.29685 | 4.36670 |
| H | -5.12147 | -2.57362 | 1.28908 |
| H | 4.68470 | 0.37847 | 2.47720 |
| H | 5.90574 | 3.45107 | -0.26515 |

**$(21a^S)^+$**

| | | | |
|---|---|---|---|
| C | 4.01799 | 5.29932 | 0.62522 |
| C | 2.90875 | 4.73099 | 1.24571 |
| C | 2.07335 | 3.87471 | 0.53776 |
| C | 2.34153 | 3.57287 | -0.80124 |
| C | 3.44282 | 4.16382 | -1.42122 |
| C | 4.27855 | 5.01994 | -0.71170 |
| C | 1.44724 | 2.61002 | -1.52389 |
| C | 1.89499 | 1.09927 | -1.45765 |
| C | 3.11899 | 0.85310 | -2.37603 |
| C | 3.73830 | -0.51466 | -2.24244 |
| C | 3.08453 | -1.66986 | -2.68239 |
| C | 3.67028 | -2.92286 | -2.52984 |
| C | 4.92635 | -3.04277 | -1.94468 |
| C | 5.59157 | -1.90115 | -1.51028 |
| C | 4.99955 | -0.65217 | -1.65631 |
| C | 0.69903 | 0.34018 | -1.95275 |
| N | -0.25647 | -0.16155 | -1.27310 |
| C | -1.26746 | -0.68896 | -2.19875 |
| C | -0.63240 | -0.40875 | -3.58014 |
| O | 0.58307 | 0.29052 | -3.27476 |
| C | -2.62263 | -0.05539 | -2.03122 |
| C | -2.75725 | 1.33047 | -1.93994 |
| C | -4.01723 | 1.90701 | -1.85443 |
| C | -5.15471 | 1.10404 | -1.86343 |
| C | -5.02637 | -0.27604 | -1.96046 |
| C | -3.76326 | -0.85264 | -2.04654 |
| Cu | -0.33281 | -0.14468 | 0.76472 |
| C | -0.50664 | -1.28446 | 2.48337 |
| C | -1.09537 | -0.55344 | 3.63085 |
| N | 1.63702 | 0.15423 | 0.87815 |

| | | | |
|---|---|---|---|
| C | 2.52694 | 0.01656 | 2.04105 |
| C | 3.70278 | 0.93225 | 1.65962 |
| O | 3.51819 | 1.13590 | 0.25645 |
| C | 2.29284 | 0.74964 | -0.05125 |
| C | 2.91857 | -1.42311 | 2.27612 |
| C | 3.21781 | -2.26678 | 1.20498 |
| C | 3.60241 | -3.58248 | 1.43900 |
| C | 3.69360 | -4.06669 | 2.74025 |
| C | 3.40246 | -3.22866 | 3.81135 |
| C | 3.01694 | -1.91308 | 3.57889 |
| O | -2.11962 | 0.58613 | 1.22524 |
| C | -1.91663 | 1.84384 | 1.07367 |
| O | -0.79510 | 2.28646 | 0.78635 |
| C | -1.20876 | -2.18986 | 1.62396 |
| C | -2.60477 | -2.18424 | 1.51889 |
| C | -3.26775 | -3.03459 | 0.62552 |
| C | -2.50074 | -3.91127 | -0.20620 |
| C | -1.08324 | -3.91100 | -0.08840 |
| C | -0.45261 | -3.08046 | 0.79004 |
| C | -4.68276 | -3.02848 | 0.51456 |
| C | -5.31246 | -3.84861 | -0.38405 |
| C | -4.55373 | -4.70758 | -1.20934 |
| C | -3.18068 | -4.74040 | -1.12245 |
| C | -3.08790 | 2.74831 | 1.22326 |
| C | -2.92254 | 4.11548 | 1.00134 |
| C | -4.01696 | 4.96785 | 1.07044 |
| C | -5.27821 | 4.45637 | 1.36521 |
| C | -5.44477 | 3.09281 | 1.59477 |
| C | -4.35202 | 2.23972 | 1.52276 |
| H | -1.25032 | 0.23814 | -4.20896 |
| H | 4.68269 | 0.47583 | 1.81712 |
| H | 2.79117 | 1.03595 | -3.40444 |
| H | 0.44478 | 2.66262 | -1.08643 |
| H | 1.38079 | 2.85203 | -2.59115 |
| H | 3.86501 | 1.61115 | -2.12330 |
| H | 2.01657 | 0.40163 | 2.93135 |
| H | 3.65678 | 1.91451 | 2.14272 |
| H | -1.36393 | -1.76688 | -2.02025 |
| H | -0.37144 | -1.31607 | -4.13455 |
| H | -1.86997 | 1.96276 | -1.90364 |
| H | -3.66479 | -1.93434 | -2.13054 |
| H | 3.14381 | -1.90482 | 0.18082 |
| H | 2.78558 | -1.25997 | 4.42033 |
| H | 2.12227 | -1.59010 | -3.18162 |
| H | 5.52025 | 0.23952 | -1.31171 |
| H | 3.15046 | -3.80864 | -2.89027 |
| H | 6.57825 | -1.98261 | -1.05905 |
| H | 3.99281 | -5.09706 | 2.92010 |
| H | -6.14013 | 1.55902 | -1.78926 |
| H | 5.39088 | -4.02116 | -1.83878 |
| H | -1.92774 | 4.48695 | 0.76673 |
| H | -4.46464 | 1.16884 | 1.67168 |
| H | -6.43192 | 2.69710 | 1.82529 |
| H | -6.13586 | 5.12437 | 1.41814 |
| H | -3.88944 | 6.03407 | 0.89478 |
| H | -3.18644 | -1.49573 | 2.12368 |
| H | 0.63242 | -3.08528 | 0.88170 |
| H | -0.50543 | -4.59060 | -0.71195 |
| H | -2.60545 | -5.41125 | -1.75767 |
| H | -5.06483 | -5.35457 | -1.91943 |
| H | -6.39687 | -3.84248 | -0.46700 |
| H | -5.25374 | -2.35336 | 1.14872 |
| H | 0.55519 | -1.51330 | 2.58196 |
| H | -2.12164 | -0.22802 | 3.45523 |
| H | -1.07042 | -1.22149 | 4.50695 |
| H | -0.49108 | 0.32470 | 3.88509 |
| H | 1.19067 | 3.44037 | 1.00681 |
| H | 3.64256 | 3.96507 | -2.47363 |
| H | 2.68432 | 4.97043 | 2.28383 |
| H | 5.13153 | 5.47799 | -1.20831 |
| H | 4.66850 | 5.97408 | 1.17796 |
| H | 3.47350 | -3.60019 | 4.83139 |
| H | 3.83418 | -4.22870 | 0.59514 |
| H | -5.91041 | -0.91093 | -1.96892 |
| H | -4.11283 | 2.98644 | -1.75447 |

**$(TS\text{-}3a^{S})^{+}$**

| | | | |
|---|---|---|---|
| C | 6.23412 | 3.04698 | -1.97465 |
| C | 4.91578 | 3.43052 | -1.74612 |
| C | 3.87868 | 2.54406 | -2.00957 |
| C | 4.14217 | 1.26273 | -2.50097 |
| C | 5.46591 | 0.89583 | -2.74458 |
| C | 6.50580 | 1.78053 | -2.48007 |
| C | 3.00876 | 0.30961 | -2.73318 |
| C | 2.61951 | -0.60010 | -1.50593 |
| C | 3.62092 | -1.77349 | -1.35965 |
| C | 3.44856 | -2.55927 | -0.08672 |
| C | 2.31110 | -3.33702 | 0.15022 |
| C | 2.16289 | -4.03126 | 1.34746 |
| C | 3.15052 | -3.96589 | 2.32507 |
| C | 4.29130 | -3.20374 | 2.09639 |

| | | | |
|---|---|---|---|
| C | 4.43326 | -2.50542 | 0.90292 |
| C | 1.26031 | -1.12851 | -1.86690 |
| N | 0.11528 | -0.88182 | -1.36206 |
| C | -0.88727 | -1.62173 | -2.13528 |
| C | -0.08267 | -2.09611 | -3.35530 |
| O | 1.28031 | -1.92297 | -2.94050 |
| C | -2.10869 | -0.81020 | -2.46816 |
| C | -2.03903 | 0.57441 | -2.61597 |
| C | -3.17167 | 1.30374 | -2.95619 |
| C | -4.38370 | 0.65339 | -3.16312 |
| C | -4.45937 | -0.72893 | -3.02408 |
| C | -3.32748 | -1.45591 | -2.67543 |
| Cu | -0.28745 | 0.46376 | 0.15377 |
| C | -1.61453 | 0.10469 | 2.20589 |
| C | -2.44056 | 1.03886 | 2.98145 |
| N | 1.70753 | 0.81586 | 0.35295 |
| C | 2.27273 | 1.52424 | 1.51029 |
| C | 3.78981 | 1.36010 | 1.29646 |
| O | 3.88409 | 0.39844 | 0.23694 |
| C | 2.66206 | 0.22612 | -0.25326 |
| C | 1.74242 | 0.90649 | 2.77696 |
| C | 1.87731 | -0.46993 | 2.98070 |
| C | 1.33604 | -1.06927 | 4.11156 |
| C | 0.66216 | -0.29467 | 5.05434 |
| C | 0.54207 | 1.07882 | 4.86430 |
| C | 1.07540 | 1.67812 | 3.72599 |
| O | -1.91431 | 1.52305 | 0.39115 |
| C | -1.58041 | 2.73511 | 0.73073 |
| O | -0.50483 | 3.01613 | 1.24702 |
| C | -2.08780 | -1.07556 | 1.56274 |
| C | -3.40274 | -1.18677 | 1.11585 |
| C | -3.86874 | -2.36956 | 0.52392 |
| C | -2.97338 | -3.47695 | 0.38880 |
| C | -1.63590 | -3.34710 | 0.85406 |
| C | -1.19170 | -2.17952 | 1.40064 |
| C | -5.19751 | -2.48580 | 0.04173 |
| C | -5.62085 | -3.64420 | -0.55538 |
| C | -4.73438 | -4.73475 | -0.69268 |
| C | -3.44185 | -4.65575 | -0.22864 |
| C | -2.62381 | 3.76815 | 0.47480 |
| C | -2.33521 | 5.10227 | 0.76642 |
| C | -3.28940 | 6.08474 | 0.54231 |
| C | -4.53542 | 5.73854 | 0.02506 |
| C | -4.82728 | 4.40913 | -0.26775 |
| C | -3.87397 | 3.42352 | -0.04511 |
| H | -0.25481 | -1.46475 | -4.23535 |
| H | 4.31720 | 0.96209 | 2.16777 |
| H | 3.50540 | -2.41501 | -2.24026 |
| H | 2.11447 | 0.88167 | -3.00912 |
| H | 3.22629 | -0.37241 | -3.56202 |
| H | 4.62705 | -1.34522 | -1.38642 |
| H | 1.95658 | 2.56969 | 1.46490 |
| H | 4.27562 | 2.27902 | 0.95215 |
| H | -1.20145 | -2.48169 | -1.52902 |
| H | -0.23786 | -3.14444 | -3.61964 |
| H | -1.09735 | 1.09168 | -2.44011 |
| H | -3.39764 | -2.53619 | -2.54340 |
| H | 2.39359 | -1.08233 | 2.24067 |
| H | 0.94629 | 2.74421 | 3.55032 |
| H | 1.54456 | -3.42706 | -0.61908 |
| H | 5.32018 | -1.89843 | 0.73045 |
| H | 1.28106 | -4.64755 | 1.51334 |
| H | 5.07453 | -3.15287 | 2.84984 |
| H | 0.23988 | -0.76173 | 5.94186 |
| H | -5.27000 | 1.22459 | -3.43206 |
| H | 3.03770 | -4.51957 | 3.25502 |
| H | -1.35417 | 5.34184 | 1.16986 |
| H | -4.07887 | 2.38075 | -0.27443 |
| H | -5.80221 | 4.14300 | -0.67160 |
| H | -5.28315 | 6.50964 | -0.15050 |
| H | -3.06496 | 7.12466 | 0.77054 |
| H | -4.07278 | -0.33396 | 1.19409 |
| H | -0.16344 | -2.07819 | 1.74387 |
| H | -0.96542 | -4.19801 | 0.75202 |
| H | -2.76709 | -5.50342 | -0.33243 |
| H | -5.08279 | -5.64964 | -1.16766 |
| H | -6.64000 | -3.73001 | -0.92524 |
| H | -5.86874 | -1.63574 | 0.14604 |
| H | -0.55313 | 0.10822 | 2.46563 |
| H | -3.44231 | 1.19348 | 2.57392 |
| H | -2.53178 | 0.58844 | 3.98484 |
| H | -1.92220 | 1.99242 | 3.11410 |
| H | 2.84653 | 2.84869 | -1.83367 |
| H | 5.68622 | -0.08772 | -3.15780 |
| H | 4.69450 | 4.42886 | -1.37327 |
| H | 7.53306 | 1.48205 | -2.67874 |
| H | 7.04775 | 3.74096 | -1.77444 |
| H | 0.02266 | 1.68709 | 5.60244 |
| H | 1.44343 | -2.14269 | 4.25584 |
| H | -5.40487 | -1.24450 | -3.18079 |
| H | -3.10882 | 2.38584 | -3.04925 |

**22a$^{S}$**

```
C   6.59890  1.16400 -0.26019
C   5.45174  1.60892  0.39175
C   4.20957  1.46107 -0.21024
C   4.09271  0.86786 -1.46975
C   5.24805  0.43353 -2.11832
C   6.49421  0.57736 -1.51606
C   2.74044  0.71122 -2.09719
C   1.93875 -0.57750 -1.69079
C   2.56739 -1.85608 -2.30855
C   1.93531 -3.12430 -1.79604
C   0.62117 -3.47118 -2.12610
C   0.02915 -4.61369 -1.59753
C   0.74938 -5.44273 -0.74325
C   2.06587 -5.12361 -0.42695
C   2.64801 -3.97178 -0.94426
C   0.57600 -0.37594 -2.28333
N  -0.56047 -0.24810 -1.71765
C  -1.56304  0.03423 -2.75594
C  -0.76667 -0.17754 -4.05748
O   0.59123 -0.29454 -3.61224
C  -2.12512  1.42075 -2.58609
C  -1.27521  2.52787 -2.54082
C  -1.79440  3.80537 -2.38051
C  -3.17112  3.98831 -2.26952
C  -4.02191  2.89161 -2.33145
C  -3.49861  1.61234 -2.49251
Cu -0.92324 -0.38858  0.24326
C  -2.12607  1.38670  2.90548
C  -2.88409  2.53294  3.52866
N   1.11816 -0.32834  0.68430
C   1.67640 -0.66761  2.00766
C   3.09799 -1.15814  1.67398
O   3.09763 -1.24543  0.24779
C   1.97036 -0.70448 -0.19371
C   0.81017 -1.69114  2.68865
C   0.40294 -2.82836  1.98560
C  -0.43180 -3.76080  2.58848
C  -0.86426 -3.56558  3.89879
C  -0.44918 -2.44192  4.60592
C   0.38724 -1.50516  4.00293
O  -1.06108  1.91530  2.07573
C   0.09050  2.25246  2.67023
O   0.34023  1.99616  3.82192
C  -2.89139  0.49069  1.97360
C  -4.00404  0.88387  1.28247
C  -4.60267  0.04687  0.29968
C  -3.95351 -1.16446 -0.06500
C  -2.80258 -1.57462  0.67643
C  -2.33492 -0.80517  1.73306
C  -5.81216  0.39519 -0.34070
C  -6.34711 -0.41201 -1.31724
C  -5.68353 -1.59297 -1.70390
C  -4.50987 -1.96256 -1.08747
C   1.00012  2.98861  1.75814
C   2.14710  3.56691  2.30660
C   2.97565  4.35165  1.51466
C   2.66583  4.55299  0.17238
C   1.53237  3.96064 -0.38014
C   0.69693  3.17916  0.40779
H  -0.83249  0.66521 -4.75059
H   3.32522 -2.14814  2.07941
H   2.46911 -1.77510 -3.39657
H   2.11674  1.57085 -1.81983
H   2.81654  0.69378 -3.18942
H   3.63383 -1.84848 -2.06577
H   1.71617  0.23406  2.62522
H   3.87836 -0.44709  1.96426
H  -2.38021 -0.68973 -2.66525
H  -1.03212 -1.10283 -4.58080
H  -0.19654  2.38778 -2.62692
H  -4.16156  0.75139 -2.54386
H   0.72570 -2.97991  0.95504
H   0.69114 -0.61035  4.54425
H   0.05829 -2.86151 -2.83071
H   3.67374 -3.71699 -0.68415
H  -0.99226 -4.87003 -1.87337
H   2.64272 -5.77382  0.22745
H  -1.52278 -4.29253  4.36941
H  -3.57766  4.98946 -2.14303
H   0.29191 -6.34405 -0.34015
H   2.35385  3.41086  3.36325
H  -0.19959  2.72649 -0.00714
H   1.29584  4.12178 -1.43060
H   3.31199  5.17151 -0.44696
H   3.86058  4.81546  1.94498
H  -4.45649  1.85424  1.47556
H  -1.64413 -1.22464  2.45867
H  -2.41367 -2.58233  0.52770
H  -4.01024 -2.89045 -1.36289
H  -6.11296 -2.22259 -2.48025
```

| | | | |
|---|---|---|---|
| H | -7.28463 | -0.13953 | -1.79709 |
| H | -6.31515 | 1.31532 | -0.04825 |
| H | -1.65457 | 0.78121 | 3.68858 |
| H | -3.25275 | 3.22580 | 2.76464 |
| H | -3.73393 | 2.15733 | 4.10851 |
| H | -2.22408 | 3.08407 | 4.20604 |
| H | 3.31126 | 1.80102 | 0.30468 |
| H | 5.17451 | -0.01224 | -3.10972 |
| H | 5.52586 | 2.08289 | 1.36919 |
| H | 7.38792 | 0.23720 | -2.03502 |
| H | 7.57436 | 1.28314 | 0.20669 |
| H | -0.78311 | -2.28854 | 5.62999 |
| H | -0.74427 | -4.64316 | 2.03289 |
| H | -5.09894 | 3.02805 | -2.25539 |
| H | -1.12754 | 4.66452 | -2.34110 |

**(21a$^{R}$)$^{+}$**

| | | | |
|---|---|---|---|
| C | 3.38460 | 4.22817 | -2.03160 |
| C | 2.14718 | 3.89998 | -1.48506 |
| C | 1.55102 | 2.68450 | -1.80489 |
| C | 2.17856 | 1.78674 | -2.67474 |
| C | 3.41400 | 2.13179 | -3.22344 |
| C | 4.01510 | 3.34369 | -2.90193 |
| C | 1.54582 | 0.45294 | -2.95007 |
| C | 1.96549 | -0.69676 | -1.93774 |
| C | 3.11043 | -1.55285 | -2.53309 |
| C | 3.69421 | -2.54998 | -1.56441 |
| C | 2.94455 | -3.62079 | -1.06926 |
| C | 3.48453 | -4.50078 | -0.13670 |
| C | 4.79187 | -4.33470 | 0.30689 |
| C | 5.55635 | -3.28523 | -0.19283 |
| C | 5.00877 | -2.40179 | -1.11532 |
| C | 0.72014 | -1.52260 | -1.76935 |
| N | -0.16911 | -1.50889 | -0.84553 |
| C | -1.31428 | -2.28769 | -1.32710 |
| C | -0.70762 | -3.05514 | -2.51217 |
| O | 0.46386 | -2.29295 | -2.82223 |
| C | -2.48410 | -1.41456 | -1.73070 |
| C | -2.38810 | -0.02788 | -1.83111 |
| C | -3.47565 | 0.72123 | -2.27138 |
| C | -4.66529 | 0.09469 | -2.61953 |
| C | -4.77031 | -1.28950 | -2.51624 |
| C | -3.68717 | -2.03634 | -2.07177 |
| Cu | -0.18914 | -0.12593 | 0.68728 |
| C | -1.50663 | -0.77021 | 2.16523 |
| C | -0.43631 | -1.70111 | 2.57840 |
| N | 1.77309 | 0.28587 | 0.34868 |
| C | 2.70345 | 0.86351 | 1.33718 |
| C | 3.98269 | 1.04227 | 0.50450 |
| O | 3.73711 | 0.25719 | -0.67319 |
| C | 2.45146 | -0.05576 | -0.67394 |
| C | 2.80522 | -0.08799 | 2.50273 |
| C | 3.25483 | -1.39502 | 2.28989 |
| C | 3.27394 | -2.30952 | 3.33494 |
| C | 2.84575 | -1.92485 | 4.60399 |
| C | 2.40343 | -0.62394 | 4.82126 |
| C | 2.37745 | 0.29187 | 3.77280 |
| O | -1.12815 | 1.60311 | 0.56178 |
| C | -0.54404 | 2.36860 | 1.41596 |
| O | 0.08518 | 1.92672 | 2.38253 |
| C | -2.79161 | -1.15348 | 1.68769 |
| C | -3.68956 | -0.13753 | 1.34036 |
| C | -4.98605 | -0.42622 | 0.89631 |
| C | -5.40408 | -1.79052 | 0.81718 |
| C | -4.48442 | -2.81344 | 1.17919 |
| C | -3.21770 | -2.51453 | 1.58511 |
| C | -5.88981 | 0.60274 | 0.52804 |
| C | -7.15361 | 0.29307 | 0.09650 |
| C | -7.56282 | -1.05453 | 0.01811 |
| C | -6.70933 | -2.07523 | 0.37291 |
| C | -0.60842 | 3.83373 | 1.14044 |
| C | 0.09994 | 4.71358 | 1.95942 |
| C | 0.12190 | 6.07104 | 1.66627 |
| C | -0.57105 | 6.55478 | 0.55923 |
| C | -1.29175 | 5.68173 | -0.25195 |
| C | -1.30990 | 4.32365 | 0.03702 |
| H | -1.34820 | -3.08809 | -3.39596 |
| H | 4.88451 | 0.65875 | 0.98872 |
| H | 2.71667 | -2.05066 | -3.42514 |
| H | 0.45677 | 0.56338 | -2.90509 |
| H | 1.79013 | 0.09352 | -3.95517 |
| H | 3.89317 | -0.86043 | -2.85738 |
| H | 2.30673 | 1.82017 | 1.68247 |
| H | 4.13642 | 2.07720 | 0.17900 |
| H | -1.64263 | -2.98165 | -0.54499 |
| H | -0.38652 | -4.06982 | -2.24491 |
| H | -1.47307 | 0.48534 | -1.55010 |
| H | -3.77996 | -3.11929 | -1.98582 |
| H | 3.58182 | -1.70809 | 1.29739 |
| H | 1.99621 | 1.29842 | 3.92746 |
| H | 1.93436 | -3.79364 | -1.43330 |

| | | | |
|---|---|---|---|
| H | 5.60420 | -1.57146 | -1.49041 |
| H | 2.88530 | -5.33084 | 0.23250 |
| H | 6.58423 | -3.15254 | 0.13839 |
| H | 2.86328 | -2.63934 | 5.42468 |
| H | -5.51341 | 0.68131 | -2.96591 |
| H | 5.21799 | -5.02789 | 1.02932 |
| H | 0.63290 | 4.30918 | 2.81672 |
| H | -1.85276 | 3.62118 | -0.59067 |
| H | -1.83465 | 6.06348 | -1.11416 |
| H | -0.55194 | 7.61835 | 0.32922 |
| H | 0.67871 | 6.75666 | 2.30175 |
| H | -3.34926 | 0.89566 | 1.38136 |
| H | -2.54519 | -3.31631 | 1.87857 |
| H | -4.81489 | -3.84954 | 1.13446 |
| H | -7.03580 | -3.11167 | 0.31311 |
| H | -8.56926 | -1.28770 | -0.32385 |
| H | -7.84630 | 1.08284 | -0.18500 |
| H | -5.55885 | 1.63746 | 0.59138 |
| H | -1.42032 | 0.24083 | 2.56923 |
| H | 0.56526 | -1.35856 | 2.23543 |
| H | -0.31317 | -1.65785 | 3.67211 |
| H | -0.57523 | -2.73272 | 2.24980 |
| H | 0.58648 | 2.42585 | -1.36816 |
| H | 3.90955 | 1.44856 | -3.91232 |
| H | 1.63965 | 4.59287 | -0.81553 |
| H | 4.97650 | 3.60313 | -3.34048 |
| H | 3.85302 | 5.17979 | -1.78853 |
| H | 2.07209 | -0.31948 | 5.81218 |
| H | 3.62644 | -3.32349 | 3.15673 |
| H | -5.70150 | -1.78816 | -2.77693 |
| H | -3.38457 | 1.80302 | -2.34397 |

**(TS-3a$^{R}$)$^{+}$**

| | | | |
|---|---|---|---|
| C | 3.25773 | 4.25418 | -1.88512 |
| C | 2.00770 | 3.81731 | -1.45608 |
| C | 1.52801 | 2.58150 | -1.87384 |
| C | 2.28457 | 1.76657 | -2.72065 |
| C | 3.53515 | 2.21695 | -3.14490 |
| C | 4.01928 | 3.45298 | -2.73075 |
| C | 1.76523 | 0.41493 | -3.11744 |
| C | 2.02433 | -0.74761 | -2.06110 |
| C | 3.11311 | -1.72020 | -2.58581 |
| C | 3.52854 | -2.75336 | -1.57088 |
| C | 2.65621 | -3.76869 | -1.16783 |
| C | 3.02978 | -4.68554 | -0.19093 |
| C | 4.29138 | -4.61267 | 0.39026 |
| C | 5.17695 | -3.62042 | -0.01640 |
| C | 4.79509 | -2.69834 | -0.98430 |
| C | 0.70301 | -1.45789 | -1.96184 |
| N | -0.21197 | -1.39180 | -1.06938 |
| C | -1.39633 | -2.08390 | -1.57615 |
| C | -0.86994 | -2.76397 | -2.85722 |
| O | 0.41584 | -2.16563 | -3.05166 |
| C | -2.56332 | -1.15153 | -1.81915 |
| C | -2.44903 | 0.23631 | -1.76117 |
| C | -3.54680 | 1.04509 | -2.04304 |
| C | -4.76413 | 0.47874 | -2.39756 |
| C | -4.88441 | -0.90621 | -2.46493 |
| C | -3.79319 | -1.71212 | -2.17130 |
| Cu | -0.14549 | -0.08256 | 0.43797 |
| C | -1.61002 | -0.29551 | 2.60514 |
| C | -0.40260 | -1.02983 | 3.01140 |
| N | 1.86082 | 0.21112 | 0.25164 |
| C | 2.80315 | 0.79998 | 1.21204 |
| C | 4.08245 | 0.94769 | 0.36751 |
| O | 3.81613 | 0.14850 | -0.79389 |
| C | 2.51891 | -0.13580 | -0.78196 |
| C | 2.92645 | -0.09141 | 2.42115 |
| C | 3.09290 | -1.46934 | 2.26209 |
| C | 3.17306 | -2.29813 | 3.37389 |
| C | 3.08866 | -1.75754 | 4.65486 |
| C | 2.92854 | -0.38546 | 4.81782 |
| C | 2.84483 | 0.44520 | 3.70456 |
| O | -1.18142 | 1.41021 | 1.14747 |
| C | -0.47555 | 2.32212 | 1.75930 |
| O | 0.23811 | 2.09124 | 2.72395 |
| C | -2.73678 | -0.85835 | 1.94490 |
| C | -3.81725 | -0.02591 | 1.64872 |
| C | -4.99958 | -0.53353 | 1.09289 |
| C | -5.10562 | -1.94035 | 0.86308 |
| C | -3.98915 | -2.77251 | 1.15150 |
| C | -2.83220 | -2.25404 | 1.65363 |
| C | -6.10238 | 0.30626 | 0.79338 |
| C | -7.26488 | -0.22565 | 0.29887 |
| C | -7.37064 | -1.61593 | 0.08308 |
| C | -6.31539 | -2.45544 | 0.35627 |
| C | -0.59687 | 3.69732 | 1.18696 |
| C | 0.11275 | 4.74045 | 1.78338 |
| C | 0.04520 | 6.02098 | 1.24968 |
| C | -0.73004 | 6.26327 | 0.11790 |
| C | -1.44432 | 5.22584 | -0.47640 |

| | | | |
|---|---|---|---|
| C | -1.38142 | 3.94583 | 0.05938 |
| H | -1.48173 | -2.56777 | -3.74152 |
| H | 4.98144 | 0.56036 | 0.85359 |
| H | 2.72264 | -2.19032 | -3.49418 |
| H | 0.68390 | 0.48953 | -3.28097 |
| H | 2.20533 | 0.09050 | -4.06561 |
| H | 3.97740 | -1.11253 | -2.86958 |
| H | 2.42567 | 1.77669 | 1.52760 |
| H | 4.24738 | 1.97660 | 0.02678 |
| H | -1.70928 | -2.83772 | -0.84402 |
| H | -0.72558 | -3.84423 | -2.74250 |
| H | -1.49920 | 0.69788 | -1.50076 |
| H | -3.90095 | -2.79649 | -2.21175 |
| H | 3.14563 | -1.90449 | 1.26441 |
| H | 2.67335 | 1.51277 | 3.82721 |
| H | 1.68239 | -3.86747 | -1.64278 |
| H | 5.48554 | -1.91475 | -1.29020 |
| H | 2.33838 | -5.47221 | 0.10488 |
| H | 6.17045 | -3.56195 | 0.42326 |
| H | 3.15034 | -2.40659 | 5.52618 |
| H | -5.61869 | 1.11274 | -2.62379 |
| H | 4.58835 | -5.33535 | 1.14763 |
| H | 0.71128 | 4.52159 | 2.66460 |
| H | -1.93178 | 3.12007 | -0.38490 |
| H | -2.05016 | 5.41947 | -1.35930 |
| H | -0.77993 | 7.26614 | -0.30169 |
| H | 0.59686 | 6.83493 | 1.71557 |
| H | -3.72404 | 1.04106 | 1.84182 |
| H | -1.99737 | -2.91125 | 1.88346 |
| H | -4.07869 | -3.84281 | 0.97391 |
| H | -6.40598 | -3.52682 | 0.18764 |
| H | -8.30155 | -2.02703 | -0.30252 |
| H | -8.11187 | 0.41928 | 0.07641 |
| H | -6.00954 | 1.37615 | 0.96932 |
| H | -1.76399 | 0.64003 | 3.13403 |
| H | 0.44986 | -0.34232 | 3.03956 |
| H | -0.55079 | -1.34486 | 4.05966 |
| H | -0.17033 | -1.91320 | 2.41027 |
| H | 0.55157 | 2.24219 | -1.52720 |
| H | 4.13526 | 1.59378 | -3.80687 |
| H | 1.39861 | 4.44171 | -0.80650 |
| H | 4.99298 | 3.79486 | -3.07573 |
| H | 3.63392 | 5.22454 | -1.56757 |
| H | 2.85863 | 0.04095 | 5.81648 |
| H | 3.30279 | -3.37010 | 3.23724 |
| H | -5.83469 | -1.36013 | -2.73760 |
| H | -3.44238 | 2.12722 | -1.99917 |

**22a$^{R}$**

| | | | |
|---|---|---|---|
| C | 5.34879 | 2.41988 | 3.26144 |
| C | 4.12181 | 1.79803 | 3.47335 |
| C | 3.06799 | 2.02504 | 2.59677 |
| C | 3.22268 | 2.87206 | 1.49605 |
| C | 4.45220 | 3.50131 | 1.30096 |
| C | 5.50880 | 3.27556 | 2.17685 |
| C | 2.08411 | 3.04893 | 0.53616 |
| C | 2.04065 | 1.99643 | -0.63779 |
| C | 3.01443 | 2.40304 | -1.77374 |
| C | 3.12691 | 1.36934 | -2.86391 |
| C | 2.05679 | 1.09719 | -3.72132 |
| C | 2.15143 | 0.10131 | -4.68777 |
| C | 3.32696 | -0.62952 | -4.82484 |
| C | 4.40765 | -0.35313 | -3.99371 |
| C | 4.30449 | 0.63467 | -3.02134 |
| C | 0.62256 | 2.06089 | -1.13598 |
| N | -0.33907 | 1.22546 | -1.02289 |
| C | -1.56669 | 1.88949 | -1.46147 |
| C | -1.03265 | 3.16376 | -2.14239 |
| O | 0.32925 | 3.23020 | -1.69914 |
| C | -2.49755 | 2.16128 | -0.30175 |
| C | -2.09930 | 1.99646 | 1.02358 |
| C | -2.97448 | 2.28682 | 2.06674 |
| C | -4.24616 | 2.77353 | 1.79361 |
| C | -4.64293 | 2.96226 | 0.47323 |
| C | -3.77919 | 2.64545 | -0.56564 |
| Cu | -0.24021 | -0.44985 | 0.06905 |
| C | -2.18096 | -2.60688 | -0.44971 |
| C | -1.22459 | -2.88426 | -1.57976 |
| N | 1.73913 | -0.26332 | 0.41359 |
| C | 2.62476 | -1.38140 | 0.76050 |
| C | 4.02231 | -0.74745 | 0.63309 |
| O | 3.77291 | 0.46937 | -0.08168 |
| C | 2.46068 | 0.66151 | -0.09122 |
| C | 2.37923 | -2.53907 | -0.17717 |
| C | 2.37651 | -2.32960 | -1.56024 |
| C | 2.22869 | -3.40088 | -2.43097 |
| C | 2.06500 | -4.69136 | -1.93193 |
| C | 2.05044 | -4.90466 | -0.55852 |
| C | 2.21395 | -3.83205 | 0.31343 |
| O | -1.41375 | -2.05218 | 0.66335 |
| C | -0.88939 | -2.93154 | 1.57192 |

| | | | |
|---|---|---|---|
| O | -0.95360 | -4.11598 | 1.42599 |
| C | -3.30542 | -1.64156 | -0.72085 |
| C | -4.11812 | -1.24976 | 0.32101 |
| C | -5.26469 | -0.45524 | 0.10868 |
| C | -5.57613 | -0.03545 | -1.21771 |
| C | -4.70993 | -0.41619 | -2.26791 |
| C | -3.60688 | -1.19921 | -2.02983 |
| C | -6.13593 | -0.09565 | 1.16640 |
| C | -7.26387 | 0.64357 | 0.92128 |
| C | -7.56718 | 1.06811 | -0.39186 |
| C | -6.74095 | 0.73966 | -1.43649 |
| C | -0.24163 | -2.23081 | 2.70220 |
| C | 0.60036 | -2.97166 | 3.53399 |
| C | 1.26989 | -2.34530 | 4.57630 |
| C | 1.08258 | -0.98342 | 4.80389 |
| C | 0.20708 | -0.25248 | 4.00461 |
| C | -0.45856 | -0.87402 | 2.95640 |
| H | -1.54372 | 4.07864 | -1.83309 |
| H | 4.72751 | -1.34853 | 0.05346 |
| H | 2.66298 | 3.36037 | -2.17314 |
| H | 1.13826 | 2.96868 | 1.08639 |
| H | 2.10136 | 4.03885 | 0.06806 |
| H | 3.99572 | 2.56812 | -1.31946 |
| H | 2.42918 | -1.69309 | 1.79132 |
| H | 4.45825 | -0.47788 | 1.60098 |
| H | -2.08934 | 1.25209 | -2.18224 |
| H | -1.02754 | 3.09457 | -3.23641 |
| H | -1.08940 | 1.65334 | 1.24380 |
| H | -4.11193 | 2.76708 | -1.59615 |
| H | 2.49795 | -1.32566 | -1.96682 |
| H | 2.21675 | -4.00412 | 1.38812 |
| H | 1.14549 | 1.68844 | -3.65682 |
| H | 5.14685 | 0.83952 | -2.36343 |
| H | 1.30894 | -0.09215 | -5.34886 |
| H | 5.33608 | -0.90990 | -4.10250 |
| H | 1.94603 | -5.52995 | -2.61490 |
| H | -4.92963 | 3.00714 | 2.60691 |
| H | 3.40586 | -1.40152 | -5.58771 |
| H | 0.71716 | -4.03629 | 3.34293 |
| H | -1.17396 | -0.31849 | 2.35221 |
| H | 0.03357 | 0.80214 | 4.20981 |
| H | 1.60359 | -0.49397 | 5.62466 |
| H | 1.93250 | -2.91952 | 5.21997 |
| H | -3.89862 | -1.58262 | 1.33506 |
| H | -2.97539 | -1.49850 | -2.86280 |
| H | -4.94290 | -0.09694 | -3.28268 |
| H | -6.97881 | 1.05800 | -2.45024 |
| H | -8.46718 | 1.65249 | -0.57245 |
| H | -7.93324 | 0.90534 | 1.73812 |
| H | -5.89757 | -0.42337 | 2.17680 |
| H | -2.58788 | -3.55444 | -0.07961 |
| H | -0.43489 | -3.55575 | -1.23025 |
| H | -1.73946 | -3.37074 | -2.41464 |
| H | -0.76717 | -1.95445 | -1.94880 |
| H | 2.11006 | 1.53366 | 2.76264 |
| H | 4.58286 | 4.18226 | 0.46090 |
| H | 3.98195 | 1.13992 | 4.32942 |
| H | 6.46092 | 3.77647 | 2.01480 |
| H | 6.17514 | 2.24906 | 3.94818 |
| H | 1.91366 | -5.90776 | -0.16086 |
| H | 2.24099 | -3.22317 | -3.50429 |
| H | -5.63914 | 3.33691 | 0.25079 |
| H | -2.65380 | 2.14698 | 3.09762 |

**(21b$^{S}$)$^{+}$**

| | | | |
|---|---|---|---|
| C | 0.61541 | 6.40137 | 0.60859 |
| C | 0.99873 | 6.25844 | -0.72054 |
| C | 0.70452 | 5.08643 | -1.40893 |
| C | 0.03126 | 4.04141 | -0.77510 |
| C | -0.37173 | 4.20059 | 0.55478 |
| C | -0.07882 | 5.37376 | 1.24122 |
| C | -0.25580 | 2.74356 | -1.46938 |
| C | 0.89440 | 1.66536 | -1.36319 |
| C | 2.05507 | 2.02961 | -2.31790 |
| C | 3.30304 | 1.19939 | -2.16516 |
| C | 3.33900 | -0.16184 | -2.48292 |
| C | 4.51210 | -0.89602 | -2.33386 |
| C | 5.67034 | -0.28496 | -1.86543 |
| C | 5.64772 | 1.06867 | -1.54563 |
| C | 4.47452 | 1.79888 | -1.69431 |
| C | 0.22805 | 0.38743 | -1.77770 |
| N | -0.39703 | -0.44549 | -1.04100 |
| C | -1.14283 | -1.36680 | -1.91625 |
| C | -0.47426 | -1.09769 | -3.27320 |
| O | 0.15828 | 0.18068 | -3.08954 |
| C | -2.62383 | -1.08072 | -1.90753 |
| C | -3.53135 | -2.07983 | -1.56603 |
| C | -4.89972 | -1.84485 | -1.64326 |
| C | -5.37169 | -0.60342 | -2.05257 |
| C | -4.47055 | 0.40481 | -2.37891 |
| C | -3.10380 | 0.16768 | -2.30695 |

| | | | |
|---|---|---|---|
| Cu | -0.45126 | -0.44029 | 0.96225 |
| C | -0.18020 | -1.39503 | 2.77161 |
| C | -1.21399 | -1.21670 | 3.81554 |
| N | 1.11658 | 0.86990 | 1.02768 |
| C | 1.98033 | 1.24507 | 2.16272 |
| C | 2.55847 | 2.59050 | 1.69841 |
| O | 2.31530 | 2.57982 | 0.28881 |
| C | 1.41730 | 1.63830 | 0.04574 |
| C | 3.02096 | 0.18029 | 2.42027 |
| C | 3.83075 | -0.27881 | 1.37751 |
| C | 4.77493 | -1.27125 | 1.61034 |
| C | 4.92326 | -1.81469 | 2.88407 |
| C | 4.13153 | -1.35221 | 3.92841 |
| C | 3.18737 | -0.35626 | 3.69602 |
| O | -2.37717 | -0.70344 | 1.30688 |
| C | -2.85561 | 0.46127 | 1.04770 |
| O | -2.12619 | 1.42214 | 0.77614 |
| C | 1.05523 | -2.58839 | 1.06816 |
| C | -0.09601 | -2.50382 | 1.86680 |
| C | -1.17489 | -3.41542 | 1.62846 |
| C | -1.07041 | -4.35503 | 0.64806 |
| C | 0.07861 | -4.43229 | -0.19315 |
| C | 1.15781 | -3.52455 | 0.02515 |
| C | 2.29262 | -3.56377 | -0.82056 |
| C | 2.35021 | -4.46233 | -1.85684 |
| C | 1.28441 | -5.36046 | -2.07370 |
| C | 0.17483 | -5.35076 | -1.25724 |
| C | -4.33719 | 0.59582 | 1.07283 |
| C | -4.91292 | 1.81209 | 0.70454 |
| C | -6.29395 | 1.94651 | 0.67591 |
| C | -7.10458 | 0.86904 | 1.02660 |
| C | -6.53308 | -0.34068 | 1.41037 |
| C | -5.15107 | -0.47814 | 1.43113 |
| H | -1.17714 | -1.01950 | -4.10500 |
| H | 3.63380 | 2.68797 | 1.86396 |
| H | 1.66407 | 1.95787 | -3.33828 |
| H | -1.15316 | 2.29821 | -1.02721 |
| H | -0.42415 | 2.89404 | -2.54275 |
| H | 2.30350 | 3.07760 | -2.13104 |
| H | 1.36352 | 1.37590 | 3.05880 |
| H | 2.02712 | 3.45223 | 2.11742 |
| H | -0.97232 | -2.39537 | -1.58749 |
| H | 0.31103 | -1.82669 | -3.51037 |
| H | -3.16091 | -3.05094 | -1.24064 |
| H | -2.40715 | 0.96299 | -2.57046 |
| H | 3.72008 | 0.12959 | 0.37350 |
| H | 2.56983 | 0.00611 | 4.51810 |
| H | 2.45566 | -0.65285 | -2.88394 |
| H | 4.45801 | 2.85710 | -1.43937 |
| H | 4.52602 | -1.94902 | -2.61026 |
| H | 6.54875 | 1.55975 | -1.18408 |
| H | 5.66038 | -2.59459 | 3.06253 |
| H | -6.44191 | -0.41440 | -2.10095 |
| H | 6.59001 | -0.85755 | -1.76273 |
| H | -4.25563 | 2.63535 | 0.43481 |
| H | -4.68446 | -1.42047 | 1.70648 |
| H | -7.16912 | -1.17832 | 1.68981 |
| H | -8.18764 | 0.97574 | 1.00681 |
| H | -6.74381 | 2.89336 | 0.38389 |
| H | 1.88621 | -1.91104 | 1.25745 |
| H | -2.07754 | -3.33750 | 2.22629 |
| H | -1.88635 | -5.05456 | 0.47546 |
| H | -0.64074 | -6.05034 | -1.42992 |
| H | 1.34403 | -6.07304 | -2.89382 |
| H | 3.22016 | -4.49314 | -2.50975 |
| H | 3.10679 | -2.86668 | -0.63304 |
| H | 0.78171 | -0.92507 | 2.98151 |
| H | -1.30861 | -0.16159 | 4.09363 |
| H | -2.19492 | -1.58545 | 3.51334 |
| H | -0.88245 | -1.75768 | 4.71688 |
| H | 0.98986 | 4.99058 | -2.45597 |
| H | -0.93542 | 3.39852 | 1.03100 |
| H | 1.52083 | 7.06747 | -1.22740 |
| H | -0.40910 | 5.49458 | 2.27161 |
| H | 0.84001 | 7.32120 | 1.14478 |
| H | 4.24815 | -1.76566 | 4.92788 |
| H | 5.39949 | -1.61842 | 0.78995 |
| H | -5.60080 | -2.63220 | -1.37451 |
| H | -4.83490 | 1.38271 | -2.68542 |

**(TS-3b$^{S}$)$^{+}$**

| | | | |
|---|---|---|---|
| C | -4.87841 | 4.08021 | 0.13882 |
| C | -4.26003 | 4.44041 | -1.05394 |
| C | -3.33645 | 3.58683 | -1.64686 |
| C | -3.01338 | 2.36686 | -1.05109 |
| C | -3.64682 | 2.00973 | 0.14090 |
| C | -4.57333 | 2.85929 | 0.73282 |
| C | -2.01012 | 1.43809 | -1.66447 |
| C | -0.50430 | 1.69023 | -1.26044 |
| C | 0.00368 | 3.01772 | -1.86523 |
| C | 1.47038 | 3.29882 | -1.66116 |

| | | | | | | | |
|---|---|---|---|---|---|---|---|
| C | 2.40030 | 2.96888 | -2.65154 | C | -5.75345 | -0.92178 | 0.21988 |
| C | 3.74952 | 3.26373 | -2.49306 | C | -4.72651 | -1.42198 | -0.57637 |
| C | 4.20025 | 3.88253 | -1.33184 | C | -3.50161 | -1.74819 | -0.00736 |
| C | 3.28620 | 4.22119 | -0.33953 | H | 0.67895 | -0.88229 | -4.57597 |
| C | 1.93407 | 3.94031 | -0.50833 | H | -0.31131 | 3.53193 | 2.67581 |
| C | 0.19813 | 0.51375 | -1.87306 | H | -0.21399 | 2.98226 | -2.93752 |
| N | 0.45505 | -0.62392 | -1.35560 | H | -2.24556 | 0.40639 | -1.37658 |
| C | 0.74290 | -1.55565 | -2.46479 | H | -2.04257 | 1.49644 | -2.75904 |
| C | 1.06372 | -0.58536 | -3.59816 | H | -0.59868 | 3.82129 | -1.43219 |
| O | 0.39794 | 0.62025 | -3.18905 | H | -1.19552 | 0.74830 | 2.96084 |
| C | -0.47370 | -2.42817 | -2.66629 | H | -1.96746 | 2.91774 | 2.38419 |
| C | -0.54359 | -3.67408 | -2.04614 | H | 1.60571 | -2.17624 | -2.20818 |
| C | -1.68793 | -4.45594 | -2.15614 | H | 2.13731 | -0.36836 | -3.66370 |
| C | -2.77435 | -3.99986 | -2.89423 | H | 0.30654 | -4.02967 | -1.46648 |
| C | -2.71450 | -2.75598 | -3.51596 | H | -1.54625 | -0.99442 | -3.87872 |
| C | -1.57368 | -1.97150 | -3.39724 | H | 1.92426 | 2.56718 | 1.86967 |
| Cu | 0.33870 | -1.18056 | 0.59736 | H | 0.10706 | -0.20190 | 4.60013 |
| C | 0.86287 | -2.63801 | 2.31941 | H | 2.05283 | 2.50706 | -3.57308 |
| C | 0.32589 | -3.90349 | 2.84838 | H | 1.21928 | 4.25627 | 0.24732 |
| N | -0.29182 | 0.77484 | 1.07240 | H | 4.45056 | 3.02104 | -3.28972 |
| C | -0.43471 | 1.32979 | 2.43363 | H | 3.62234 | 4.73585 | 0.55904 |
| C | -0.90526 | 2.76840 | 2.16541 | H | 4.30844 | 0.68921 | 4.90503 |
| O | -0.73020 | 2.92837 | 0.75158 | H | -3.66832 | -4.61298 | -2.98498 |
| C | -0.45450 | 1.73215 | 0.24243 | H | 5.25448 | 4.12521 | -1.21141 |
| C | 0.87366 | 1.20067 | 3.16927 | H | -4.14390 | -0.92412 | 3.22167 |
| C | 2.00127 | 1.89620 | 2.72532 | H | -2.68834 | -2.14321 | -0.61184 |
| C | 3.23081 | 1.71499 | 3.34585 | H | -4.88069 | -1.56105 | -1.64460 |
| C | 3.34556 | 0.83441 | 4.41986 | H | -6.71230 | -0.66685 | -0.22706 |
| C | 2.22396 | 0.15167 | 4.87707 | H | -6.36562 | -0.36569 | 2.20892 |
| C | 0.98977 | 0.33693 | 4.25688 | H | 2.30617 | -0.46897 | 1.78361 |
| O | -1.11677 | -2.42291 | 1.18698 | H | 1.85971 | -4.65249 | 0.74208 |
| C | -1.95982 | -1.82046 | 1.97151 | H | 3.61316 | -4.41066 | -0.95739 |
| O | -1.69867 | -1.45280 | 3.11128 | H | 5.17105 | -2.99622 | -2.23794 |
| C | 2.49356 | -1.29507 | 1.09756 | H | 6.16667 | -0.78032 | -2.69018 |
| C | 1.89605 | -2.55260 | 1.33758 | H | 5.44198 | 1.21039 | -1.39264 |
| C | 2.31442 | -3.68326 | 0.55837 | H | 3.72226 | 0.99438 | 0.36538 |
| C | 3.28279 | -3.54591 | -0.38466 | H | 0.70158 | -1.75527 | 2.93997 |
| C | 3.87925 | -2.27827 | -0.66520 | H | -0.56422 | -3.71030 | 3.44964 |
| C | 3.47519 | -1.13677 | 0.08583 | H | 0.09572 | -4.63026 | 2.06537 |
| C | 4.04866 | 0.12187 | -0.19748 | H | 1.10444 | -4.33458 | 3.50027 |
| C | 5.00099 | 0.24042 | -1.18264 | H | -2.86937 | 3.86726 | -2.59026 |
| C | 5.40717 | -0.88747 | -1.91843 | H | -3.41801 | 1.05041 | 0.60221 |
| C | 4.85475 | -2.12474 | -1.66793 | H | -4.50557 | 5.38703 | -1.53086 |
| C | -3.29846 | -1.55825 | 1.36107 | H | -5.06917 | 2.55548 | 1.65318 |
| C | -4.32985 | -1.05990 | 2.15852 | H | -5.60762 | 4.74495 | 0.59731 |
| C | -5.55748 | -0.74771 | 1.58821 | H | 4.10362 | 2.25950 | 2.98997 |

| | | | |
|---|---|---|---|
| H | 2.30754 | -0.52623 | 5.72424 |
| H | -1.73137 | -5.42557 | -1.66513 |
| H | -3.56038 | -2.39522 | -4.09779 |

**22b$^{S}$**

| | | | |
|---|---|---|---|
| C | -5.34231 | 3.45259 | 0.20884 |
| C | -5.00026 | 3.74015 | -1.10784 |
| C | -4.08670 | 2.93784 | -1.78389 |
| C | -3.50078 | 1.84235 | -1.14994 |
| C | -3.85875 | 1.55557 | 0.16965 |
| C | -4.77163 | 2.35410 | 0.84589 |
| C | -2.49527 | 0.97544 | -1.84540 |
| C | -0.98537 | 1.38586 | -1.64756 |
| C | -0.63939 | 2.65702 | -2.45719 |
| C | 0.72262 | 3.21776 | -2.13911 |
| C | 1.89306 | 2.49699 | -2.39362 |
| C | 3.13914 | 3.02218 | -2.06594 |
| C | 3.23771 | 4.28155 | -1.48419 |
| C | 2.08097 | 5.01177 | -1.23034 |
| C | 0.83734 | 4.47992 | -1.55132 |
| C | -0.24920 | 0.19144 | -2.18879 |
| N | 0.25494 | -0.80802 | -1.57225 |
| C | 0.50627 | -1.86300 | -2.57802 |
| C | 0.46264 | -1.06479 | -3.88220 |
| O | -0.27745 | 0.11166 | -3.52087 |
| C | -0.56337 | -2.92249 | -2.45232 |
| C | -0.28880 | -4.14689 | -1.84791 |
| C | -1.29168 | -5.09690 | -1.68082 |
| C | -2.58004 | -4.83639 | -2.13159 |
| C | -2.85997 | -3.62321 | -2.75453 |
| C | -1.86114 | -2.66993 | -2.90624 |
| Cu | 0.80706 | -0.72896 | 0.36352 |
| C | 1.07705 | -2.07575 | 2.80286 |
| C | 1.28712 | -3.17020 | 3.82285 |
| N | -0.33751 | 0.91727 | 0.74600 |
| C | -0.31847 | 1.71019 | 1.99006 |
| C | -1.05295 | 2.99944 | 1.58068 |
| O | -1.13107 | 2.90497 | 0.15489 |
| C | -0.76643 | 1.67764 | -0.18882 |
| C | 1.10154 | 1.91818 | 2.44509 |
| C | 2.02274 | 2.51329 | 1.57979 |
| C | 3.33369 | 2.71819 | 1.98952 |
| C | 3.74205 | 2.31242 | 3.25883 |
| C | 2.82860 | 1.71657 | 4.12197 |
| C | 1.50868 | 1.52629 | 3.71901 |
| O | -0.26164 | -2.15141 | 2.25658 |
| C | -1.23255 | -1.51077 | 2.92885 |
| O | -1.02854 | -0.93235 | 3.96757 |
| C | 2.79554 | -1.04703 | 1.27424 |
| C | 1.98276 | -2.12980 | 1.59961 |
| C | 2.05155 | -3.31495 | 0.81056 |
| C | 2.92659 | -3.40707 | -0.23584 |
| C | 3.74546 | -2.31017 | -0.61078 |
| C | 3.67489 | -1.10832 | 0.15067 |
| C | 4.49571 | -0.01756 | -0.21154 |
| C | 5.34271 | -0.11014 | -1.28926 |
| C | 5.40398 | -1.29367 | -2.05178 |
| C | 4.62252 | -2.37289 | -1.71760 |
| C | -2.54644 | -1.57352 | 2.25013 |
| C | -3.64784 | -1.06525 | 2.94489 |
| C | -4.91149 | -1.10624 | 2.37291 |
| C | -5.07644 | -1.64403 | 1.09953 |
| C | -3.97975 | -2.14255 | 0.40155 |
| C | -2.71239 | -2.11579 | 0.97170 |
| H | -0.06020 | -1.56671 | -4.69923 |
| H | -0.51094 | 3.91423 | 1.83490 |
| H | -0.72409 | 2.40534 | -3.51963 |
| H | -2.59181 | -0.05313 | -1.47506 |
| H | -2.66891 | 0.96059 | -2.92765 |
| H | -1.40139 | 3.40763 | -2.22990 |
| H | -0.86924 | 1.17508 | 2.76813 |
| H | -2.07692 | 3.04958 | 1.96563 |
| H | 1.49182 | -2.30302 | -2.40688 |
| H | 1.45824 | -0.74810 | -4.21603 |
| H | 0.72065 | -4.35931 | -1.50763 |
| H | -2.09681 | -1.72290 | -3.39112 |
| H | 1.71714 | 2.81985 | 0.57898 |
| H | 0.78862 | 1.06152 | 4.39164 |
| H | 1.84352 | 1.52479 | -2.88011 |
| H | -0.06722 | 5.04768 | -1.33994 |
| H | 4.03653 | 2.44799 | -2.28361 |
| H | 2.14661 | 6.00045 | -0.78084 |
| H | 4.77059 | 2.46888 | 3.57729 |
| H | -3.36325 | -5.58137 | -2.00905 |
| H | 4.21308 | 4.69833 | -1.24056 |
| H | -3.48917 | -0.65499 | 3.93967 |
| H | -1.85982 | -2.52101 | 0.43045 |
| H | -4.11159 | -2.56390 | -0.59104 |
| H | -6.06559 | -1.67439 | 0.64732 |
| H | -5.76998 | -0.72168 | 2.91936 |
| H | 2.84698 | -0.18025 | 1.93265 |

| | | | |
|---|---|---|---|
| H | 1.40917 | -4.15462 | 1.06706 |
| H | 2.99831 | -4.32943 | -0.81078 |
| H | 4.67662 | -3.29433 | -2.29501 |
| H | 6.08165 | -1.35353 | -2.90072 |
| H | 5.97788 | 0.73249 | -1.55514 |
| H | 4.44056 | 0.89481 | 0.37655 |
| H | 1.18030 | -1.09827 | 3.28522 |
| H | 0.59328 | -3.03069 | 4.65800 |
| H | 1.12211 | -4.16106 | 3.38592 |
| H | 2.31000 | -3.12654 | 4.21203 |
| H | -3.83507 | 3.16016 | -2.82031 |
| H | -3.41227 | 0.69687 | 0.66920 |
| H | -5.45304 | 4.58937 | -1.61537 |
| H | -5.04686 | 2.10977 | 1.87052 |
| H | -6.06175 | 4.07713 | 0.73426 |
| H | 4.03713 | 3.19983 | 1.31280 |
| H | 3.14130 | 1.40472 | 5.11636 |
| H | -1.06234 | -6.04879 | -1.20646 |
| H | -3.86210 | -3.41967 | -3.12711 |

**$(21b^{R})^{+}$**

| | | | |
|---|---|---|---|
| C | -0.77748 | 5.18913 | 2.36264 |
| C | 0.53423 | 5.44900 | 2.75267 |
| C | 1.52310 | 4.49426 | 2.55964 |
| C | 1.22617 | 3.26070 | 1.97322 |
| C | -0.08983 | 3.01173 | 1.58669 |
| C | -1.08512 | 3.96697 | 1.78117 |
| C | 2.34145 | 2.26377 | 1.78980 |
| C | 1.98368 | 0.96919 | 1.04452 |
| C | 3.24649 | 0.05082 | 0.93137 |
| C | 4.40294 | 0.65150 | 0.18623 |
| C | 5.46269 | 1.24714 | 0.87018 |
| C | 6.52825 | 1.81460 | 0.17876 |
| C | 6.54854 | 1.79392 | -1.21070 |
| C | 5.49950 | 1.19884 | -1.90584 |
| C | 4.43930 | 0.63143 | -1.21129 |
| C | 1.52750 | 1.27923 | -0.35731 |
| N | 0.84511 | 0.53701 | -1.14326 |
| C | 0.73311 | 1.24315 | -2.43388 |
| C | 1.62586 | 2.48226 | -2.22746 |
| O | 1.99524 | 2.41724 | -0.84691 |
| C | -0.71010 | 1.53075 | -2.75214 |
| C | -1.42146 | 2.45007 | -1.98081 |
| C | -2.76749 | 2.67977 | -2.22934 |
| C | -3.42004 | 1.98093 | -3.24050 |
| C | -2.71651 | 1.06860 | -4.01809 |
| C | -1.36357 | 0.85085 | -3.77753 |
| Cu | -0.64350 | -0.89942 | -0.51037 |
| C | -1.19399 | -2.41220 | -1.85728 |
| C | -2.18255 | -3.25678 | -1.15269 |
| N | -0.00803 | -0.52819 | 1.39869 |
| C | -0.86257 | -0.83182 | 2.56517 |
| C | 0.09642 | -0.58039 | 3.72531 |
| O | 1.07374 | 0.29896 | 3.14578 |
| C | 0.95486 | 0.19567 | 1.82637 |
| C | -1.53492 | -2.17283 | 2.49096 |
| C | -2.90939 | -2.23106 | 2.25629 |
| C | -3.56241 | -3.45641 | 2.17024 |
| C | -2.84631 | -4.63975 | 2.31182 |
| C | -1.47384 | -4.59404 | 2.54657 |
| C | -0.82549 | -3.36703 | 2.63925 |
| O | -2.52405 | -0.51379 | -0.74733 |
| C | -3.03309 | 0.40730 | -0.00035 |
| O | -2.40539 | 1.10106 | 0.78995 |
| C | 0.21627 | -2.58181 | -1.77265 |
| C | 0.79077 | -3.05850 | -0.58149 |
| C | 2.18728 | -3.13491 | -0.42581 |
| C | 3.02665 | -2.71543 | -1.50093 |
| C | 2.42733 | -2.25173 | -2.71094 |
| C | 1.07853 | -2.16688 | -2.84257 |
| C | 2.77293 | -3.59564 | 0.77752 |
| C | 4.13832 | -3.61161 | 0.92246 |
| C | 4.96465 | -3.17924 | -0.13302 |
| C | 4.42161 | -2.75178 | -1.32513 |
| C | -4.51646 | 0.56591 | -0.14464 |
| C | -5.23893 | -0.15399 | -1.09766 |
| C | -6.61174 | 0.02255 | -1.21118 |
| C | -7.27011 | 0.91794 | -0.37219 |
| C | -6.55346 | 1.63799 | 0.57943 |
| C | -5.17964 | 1.46422 | 0.69169 |
| H | 1.10542 | 3.42815 | -2.40109 |
| H | 0.61403 | -1.48498 | 4.07005 |
| H | 3.54451 | -0.20407 | 1.95500 |
| H | 3.16264 | 2.73527 | 1.23917 |
| H | 2.74197 | 1.97589 | 2.76939 |
| H | 2.92227 | -0.86930 | 0.43879 |
| H | -1.64144 | -0.05981 | 2.54917 |
| H | -0.36093 | -0.07406 | 4.57724 |
| H | 1.14488 | 0.59513 | -3.21658 |
| H | 2.54661 | 2.45675 | -2.81981 |
| H | -0.92868 | 2.97503 | -1.16242 |

| | | | |
|---|---|---|---|
| H | -0.80600 | 0.15520 | -4.40550 |
| H | -3.47327 | -1.30554 | 2.15355 |
| H | 0.24405 | -3.33813 | 2.84448 |
| H | 5.45386 | 1.26491 | 1.95937 |
| H | 3.62219 | 0.15906 | -1.75673 |
| H | 7.34682 | 2.27349 | 0.72923 |
| H | 5.51331 | 1.17376 | -2.99413 |
| H | -3.35639 | -5.59887 | 2.25096 |
| H | -4.47956 | 2.14919 | -3.42051 |
| H | 7.38165 | 2.23677 | -1.75224 |
| H | -4.70660 | -0.83709 | -1.75465 |
| H | -4.59604 | 2.01625 | 1.42488 |
| H | -7.06880 | 2.33752 | 1.23453 |
| H | -8.34598 | 1.05574 | -0.46141 |
| H | -7.17326 | -0.53661 | -1.95711 |
| H | 0.15418 | -3.40181 | 0.23100 |
| H | 0.62706 | -1.81020 | -3.76522 |
| H | 3.07950 | -1.96131 | -3.53275 |
| H | 5.06849 | -2.42031 | -2.13436 |
| H | 6.04495 | -3.18747 | -0.00571 |
| H | 4.58691 | -3.95918 | 1.85014 |
| H | 2.12466 | -3.93179 | 1.58335 |
| H | -1.56589 | -1.87742 | -2.73363 |
| H | -2.43893 | -4.08824 | -1.83027 |
| H | -1.81701 | -3.69118 | -0.21770 |
| H | -3.09741 | -2.68919 | -0.96208 |
| H | 2.54627 | 4.70898 | 2.86667 |
| H | -0.39638 | 2.07560 | 1.12437 |
| H | 0.78827 | 6.40333 | 3.20989 |
| H | -2.10095 | 3.72763 | 1.47339 |
| H | -1.55220 | 5.93767 | 2.51568 |
| H | -4.63594 | -3.48360 | 1.99558 |
| H | -0.91195 | -5.51737 | 2.67403 |
| H | -3.31715 | 3.38856 | -1.61373 |
| H | -3.21968 | 0.53325 | -4.82084 |

**(TS-3b$^{R}$)$^{+}$**

| | | | |
|---|---|---|---|
| C | -1.43692 | 5.43748 | 1.54146 |
| C | -0.25483 | 5.76879 | 2.19827 |
| C | 0.76933 | 4.83816 | 2.29780 |
| C | 0.63982 | 3.55875 | 1.74957 |
| C | -0.54888 | 3.23493 | 1.09561 |
| C | -1.57607 | 4.17058 | 0.99213 |
| C | 1.79841 | 2.60538 | 1.89742 |
| C | 1.62339 | 1.21249 | 1.28074 |
| C | 2.88256 | 0.32838 | 1.57019 |
| C | 4.15595 | 0.82256 | 0.95261 |
| C | 5.03780 | 1.64244 | 1.65759 |
| C | 6.20222 | 2.11073 | 1.05850 |
| C | 6.50196 | 1.76175 | -0.25394 |
| C | 5.63436 | 0.93656 | -0.96286 |
| C | 4.47226 | 0.47109 | -0.36080 |
| C | 1.47078 | 1.32428 | -0.21937 |
| N | 0.95664 | 0.48866 | -1.03779 |
| C | 1.00968 | 1.11128 | -2.38111 |
| C | 2.00552 | 2.25360 | -2.16349 |
| O | 2.02111 | 2.41661 | -0.74081 |
| C | -0.38353 | 1.51833 | -2.79182 |
| C | -0.94646 | 2.70972 | -2.33601 |
| C | -2.27119 | 3.01374 | -2.62065 |
| C | -3.05114 | 2.12906 | -3.35784 |
| C | -2.49320 | 0.94618 | -3.82940 |
| C | -1.16395 | 0.64891 | -3.55095 |
| Cu | -0.42572 | -1.02302 | -0.48415 |
| C | -1.28164 | -3.05557 | -1.48830 |
| C | -1.83653 | -3.98321 | -0.49141 |
| N | -0.18804 | -0.50067 | 1.49421 |
| C | -1.26259 | -0.77859 | 2.45382 |
| C | -0.79734 | -0.00203 | 3.69435 |
| O | 0.18944 | 0.89905 | 3.17271 |
| C | 0.48052 | 0.48944 | 1.94275 |
| C | -1.49557 | -2.24597 | 2.66525 |
| C | -2.78061 | -2.77645 | 2.58008 |
| C | -2.99717 | -4.13323 | 2.80301 |
| C | -1.92827 | -4.97220 | 3.09755 |
| C | -0.63832 | -4.45053 | 3.17737 |
| C | -0.42640 | -3.09301 | 2.96823 |
| O | -2.71563 | -1.46874 | -0.74425 |
| C | -2.93598 | -0.23043 | -0.53769 |
| O | -2.02951 | 0.59240 | -0.35102 |
| C | 0.10572 | -2.79869 | -1.65427 |
| C | 0.99736 | -2.89894 | -0.56650 |
| C | 2.39435 | -2.80361 | -0.76657 |
| C | 2.89319 | -2.56669 | -2.08148 |
| C | 1.96961 | -2.39829 | -3.15697 |
| C | 0.62981 | -2.49379 | -2.95806 |
| C | 3.30747 | -3.00938 | 0.29354 |
| C | 4.66154 | -2.98508 | 0.06323 |
| C | 5.15171 | -2.75942 | -1.23702 |
| C | 4.28610 | -2.55174 | -2.28828 |
| C | -4.36670 | 0.19630 | -0.47458 |

| | | | |
|---|---|---|---|
| C | -5.39580 | -0.70545 | -0.74692 |
| C | -6.71969 | -0.28859 | -0.68773 |
| C | -7.02010 | 1.02814 | -0.35122 |
| C | -5.99553 | 1.93008 | -0.07503 |
| C | -4.67229 | 1.51578 | -0.13838 |
| H | 1.71148 | 3.20076 | -2.62069 |
| H | -0.31322 | -0.64714 | 4.43820 |
| H | 2.98038 | 0.26600 | 2.66085 |
| H | 2.69107 | 3.05203 | 1.44701 |
| H | 2.01529 | 2.46650 | 2.96330 |
| H | 2.65250 | -0.67392 | 1.19467 |
| H | -2.17635 | -0.32804 | 2.04439 |
| H | -1.58326 | 0.58501 | 4.17403 |
| H | 1.39714 | 0.37720 | -3.09420 |
| H | 3.02323 | 1.99291 | -2.47710 |
| H | -0.35996 | 3.40039 | -1.73218 |
| H | -0.72118 | -0.26775 | -3.93498 |
| H | -3.61364 | -2.12332 | 2.32277 |
| H | 0.58003 | -2.67925 | 3.04136 |
| H | 4.81194 | 1.91411 | 2.68805 |
| H | 3.79553 | -0.18243 | -0.90966 |
| H | 6.88101 | 2.74819 | 1.62112 |
| H | 5.86927 | 0.64696 | -1.98593 |
| H | -2.09811 | -6.03284 | 3.27058 |
| H | -4.09250 | 2.36319 | -3.56810 |
| H | 7.41443 | 2.12651 | -0.72083 |
| H | -5.14037 | -1.72865 | -1.00985 |
| H | -3.85245 | 2.19840 | 0.06672 |
| H | -6.23249 | 2.95879 | 0.18913 |
| H | -8.05759 | 1.35341 | -0.30377 |
| H | -7.52181 | -0.99118 | -0.90450 |
| H | 0.62894 | -3.18550 | 0.41679 |
| H | -0.06464 | -2.41067 | -3.79043 |
| H | 2.36297 | -2.22096 | -4.15625 |
| H | 4.66997 | -2.38346 | -3.29260 |
| H | 6.22574 | -2.74739 | -1.40921 |
| H | 5.35905 | -3.14595 | 0.88160 |
| H | 2.91923 | -3.20122 | 1.29223 |
| H | -1.90673 | -2.85682 | -2.35668 |
| H | -1.82552 | -4.97926 | -0.96625 |
| H | -1.25713 | -4.05133 | 0.43217 |
| H | -2.87290 | -3.73003 | -0.26342 |
| H | 1.69218 | 5.10714 | 2.81083 |
| H | -0.71569 | 2.25892 | 0.63912 |
| H | -0.13035 | 6.75837 | 2.63340 |
| H | -2.49035 | 3.89643 | 0.46989 |
| H | -2.24188 | 6.16483 | 1.45982 |
| H | -4.00563 | -4.53714 | 2.74121 |
| H | 0.19945 | -5.10341 | 3.41469 |
| H | -2.69918 | 3.94636 | -2.25823 |
| H | -3.09375 | 0.25815 | -4.42101 |

**22b$^{R}$**

| | | | |
|---|---|---|---|
| C | -0.29710 | 6.07186 | 0.63547 |
| C | 0.79550 | 6.18406 | 1.49128 |
| C | 1.51412 | 5.05532 | 1.85975 |
| C | 1.15861 | 3.78733 | 1.39147 |
| C | 0.05176 | 3.68643 | 0.54806 |
| C | -0.66462 | 4.81866 | 0.16562 |
| C | 1.99733 | 2.60372 | 1.80390 |
| C | 1.54121 | 1.24429 | 1.26547 |
| C | 2.43660 | 0.07969 | 1.82452 |
| C | 3.83489 | 0.06239 | 1.28407 |
| C | 4.87209 | 0.76226 | 1.90173 |
| C | 6.15147 | 0.75459 | 1.35666 |
| C | 6.41048 | 0.04246 | 0.19010 |
| C | 5.38535 | -0.66873 | -0.42614 |
| C | 4.10726 | -0.65710 | 0.11861 |
| C | 1.67901 | 1.19985 | -0.23478 |
| N | 1.15014 | 0.36321 | -1.04155 |
| C | 1.63543 | 0.67524 | -2.39866 |
| C | 2.73803 | 1.71451 | -2.12150 |
| O | 2.54746 | 2.06159 | -0.74220 |
| C | 0.49311 | 1.16415 | -3.24993 |
| C | -0.15811 | 2.35114 | -2.91059 |
| C | -1.26662 | 2.77579 | -3.62878 |
| C | -1.73320 | 2.01890 | -4.69999 |
| C | -1.07009 | 0.85330 | -5.06307 |
| C | 0.04150 | 0.43027 | -4.34130 |
| Cu | -0.40322 | -0.82805 | -0.51763 |
| C | -2.67952 | -2.65283 | -1.57522 |
| C | -3.30547 | -3.96394 | -1.16166 |
| N | -0.73732 | 0.18021 | 1.25092 |
| C | -1.78055 | -0.03760 | 2.27361 |
| C | -1.41814 | 1.00770 | 3.33726 |
| O | -0.07106 | 1.37172 | 3.00592 |
| C | 0.16135 | 0.92166 | 1.77750 |
| C | -1.74757 | -1.46983 | 2.73835 |
| C | -2.83192 | -2.31288 | 2.50680 |
| C | -2.78321 | -3.64777 | 2.89922 |
| C | -1.65152 | -4.14681 | 3.53465 |

| | | | |
|---|---|---|---|
| C | -0.57052 | -3.30450 | 3.78477 |
| C | -0.61672 | -1.97510 | 3.38367 |
| O | -3.29006 | -1.63969 | -0.75588 |
| C | -3.05150 | -0.37327 | -1.10537 |
| O | -2.23285 | -0.07567 | -1.94949 |
| C | -1.16241 | -2.68493 | -1.49423 |
| C | -0.47415 | -3.00569 | -0.32434 |
| C | 0.92682 | -3.29477 | -0.36261 |
| C | 1.61069 | -3.28348 | -1.61379 |
| C | 0.89099 | -2.92079 | -2.78107 |
| C | -0.43931 | -2.61246 | -2.72063 |
| C | 1.64065 | -3.66489 | 0.80069 |
| C | 2.96482 | -4.02391 | 0.72731 |
| C | 3.63511 | -4.03177 | -0.51257 |
| C | 2.97313 | -3.66094 | -1.65726 |
| C | -3.83140 | 0.60372 | -0.32232 |
| C | -4.95404 | 0.22850 | 0.41931 |
| C | -5.58781 | 1.16407 | 1.22628 |
| C | -5.09402 | 2.46442 | 1.30224 |
| C | -3.98383 | 2.84275 | 0.54951 |
| C | -3.35869 | 1.91669 | -0.27421 |
| H | 2.64276 | 2.62381 | -2.72045 |
| H | -1.43408 | 0.62858 | 4.36203 |
| H | 2.43225 | 0.17065 | 2.91764 |
| H | 3.02900 | 2.76297 | 1.47230 |
| H | 2.01778 | 2.53834 | 2.89763 |
| H | 1.93273 | -0.85893 | 1.55795 |
| H | -2.76290 | 0.17401 | 1.84953 |
| H | -2.03607 | 1.90947 | 3.26064 |
| H | 2.05237 | -0.23823 | -2.83713 |
| H | 3.74857 | 1.30504 | -2.22116 |
| H | 0.20636 | 2.95013 | -2.07608 |
| H | 0.56183 | -0.48297 | -4.62546 |
| H | -3.71443 | -1.92223 | 2.00473 |
| H | 0.23624 | -1.32266 | 3.57550 |
| H | 4.67905 | 1.31166 | 2.82235 |
| H | 3.30391 | -1.21817 | -0.35528 |
| H | 6.95234 | 1.30186 | 1.84940 |
| H | 5.58376 | -1.24645 | -1.32753 |
| H | -1.61412 | -5.18851 | 3.84594 |
| H | -2.60528 | 2.34760 | -5.26128 |
| H | 7.41355 | 0.03289 | -0.23120 |
| H | -5.31855 | -0.79402 | 0.35924 |
| H | -2.49317 | 2.17605 | -0.88065 |
| H | -3.60948 | 3.86195 | 0.61038 |
| H | -5.58481 | 3.19228 | 1.94537 |
| H | -6.46651 | 0.88049 | 1.80129 |
| H | -1.01089 | -3.17807 | 0.60831 |
| H | -0.98071 | -2.32590 | -3.61947 |
| H | 1.41527 | -2.89638 | -3.73539 |
| H | 3.48480 | -3.66575 | -2.61832 |
| H | 4.68097 | -4.32751 | -0.55731 |
| H | 3.49980 | -4.31464 | 1.62870 |
| H | 1.11356 | -3.67768 | 1.75129 |
| H | -2.93727 | -2.41194 | -2.61356 |
| H | -2.90815 | -4.77857 | -1.77529 |
| H | -3.08233 | -4.18106 | -0.11116 |
| H | -4.39201 | -3.92396 | -1.29008 |
| H | 2.37423 | 5.15757 | 2.52039 |
| H | -0.26731 | 2.72139 | 0.15964 |
| H | 1.09388 | 7.16018 | 1.86817 |
| H | -1.50824 | 4.71653 | -0.51595 |
| H | -0.85357 | 6.95739 | 0.33634 |
| H | -3.63535 | -4.29868 | 2.71349 |
| H | 0.31043 | -3.68729 | 4.29650 |
| H | -1.76781 | 3.70304 | -3.35636 |
| H | -1.41825 | 0.27126 | -5.91405 |

**(21c$^{S}$)$^{+}$**

| | | | |
|---|---|---|---|
| C | 4.27051 | -5.24109 | -0.76667 |
| C | 4.46447 | -4.85100 | -2.08831 |
| C | 3.78049 | -3.75753 | -2.60272 |
| C | 2.88645 | -3.02966 | -1.81450 |
| C | 2.69902 | -3.43519 | -0.49291 |
| C | 3.38483 | -4.52745 | 0.02993 |
| C | 2.18616 | -1.84687 | -2.43996 |
| C | 1.17431 | -1.09614 | -1.56904 |
| C | 0.48814 | 0.06790 | -2.36327 |
| C | 1.44450 | 1.14001 | -2.79606 |
| C | 2.01726 | 1.13216 | -4.06859 |
| C | 2.92205 | 2.11938 | -4.44370 |
| C | 3.26434 | 3.12991 | -3.55135 |
| C | 2.69428 | 3.15217 | -2.28239 |
| C | 1.79285 | 2.16230 | -1.91128 |
| C | 1.85368 | -0.50047 | -0.36584 |
| N | 1.30515 | -0.07587 | 0.71937 |
| C | 2.38341 | 0.20084 | 1.66961 |
| C | 3.60001 | 0.29370 | 0.74048 |
| O | 3.16052 | -0.39506 | -0.43876 |
| C | 2.47594 | -0.86651 | 2.73955 |
| C | 3.45323 | -0.73948 | 3.72905 |

| | | | |
|---|---|---|---|
| C | 3.56809 | -1.68898 | 4.73599 |
| C | 2.69957 | -2.77616 | 4.77250 |
| C | 1.71870 | -2.90472 | 3.79730 |
| C | 1.60642 | -1.95570 | 2.78498 |
| Cu | -0.64084 | -0.08449 | 1.01330 |
| C | -0.75428 | 1.61365 | 2.32948 |
| C | -0.52309 | 0.94937 | 3.64007 |
| N | -0.74562 | -1.79771 | -0.16785 |
| C | -1.75384 | -2.87095 | -0.19605 |
| C | -1.05553 | -3.91105 | -1.09239 |
| O | -0.10410 | -3.12181 | -1.82862 |
| C | 0.07098 | -2.01738 | -1.12455 |
| C | -3.07622 | -2.42830 | -0.77163 |
| C | -3.13255 | -1.62156 | -1.90894 |
| C | -4.35423 | -1.32675 | -2.49901 |
| C | -5.53332 | -1.83074 | -1.95794 |
| C | -5.48438 | -2.62751 | -0.82128 |
| C | -4.26030 | -2.92288 | -0.23075 |
| C | -3.04449 | 0.41460 | 0.56949 |
| O | -2.31650 | 1.01010 | -0.24676 |
| O | -2.53015 | -0.28525 | 1.51693 |
| C | -4.52208 | 0.51582 | 0.50817 |
| C | -5.32002 | -0.12369 | 1.45772 |
| C | -6.70235 | -0.02135 | 1.38186 |
| C | -7.28890 | 0.72242 | 0.36140 |
| C | -6.49431 | 1.37156 | -0.58021 |
| C | -5.11228 | 1.26988 | -0.50659 |
| C | 0.05320 | 2.70824 | 1.85278 |
| C | -0.35357 | 3.39248 | 0.70772 |
| C | 0.36252 | 4.50105 | 0.22151 |
| C | 1.52517 | 4.93956 | 0.92633 |
| C | 1.90822 | 4.25376 | 2.11035 |
| C | 1.20369 | 3.17371 | 2.55985 |
| C | -0.03188 | 5.18396 | -0.95674 |
| C | 0.69281 | 6.25459 | -1.41481 |
| C | 1.83751 | 6.68935 | -0.71366 |
| C | 2.24571 | 6.04725 | 0.43262 |
| H | 4.49304 | -0.20825 | 1.11896 |
| H | -1.72274 | -4.40425 | -1.80134 |
| H | 0.00106 | -0.38987 | -3.23264 |
| H | 2.93536 | -1.12170 | -2.77668 |
| H | 1.64846 | -2.18441 | -3.33443 |
| H | -0.30505 | 0.48330 | -1.72542 |
| H | -1.91084 | -3.25398 | 0.81676 |
| H | -0.48508 | -4.65617 | -0.52392 |
| H | 2.21323 | 1.16966 | 2.14513 |
| H | 3.83296 | 1.32505 | 0.44968 |
| H | 4.13448 | 0.11161 | 3.71405 |
| H | 0.83193 | -2.06733 | 2.02787 |
| H | -2.22029 | -1.20244 | -2.32928 |
| H | -4.22539 | -3.54346 | 0.66391 |
| H | 1.74605 | 0.34901 | -4.77564 |
| H | 1.33458 | 2.18927 | -0.92485 |
| H | 3.35681 | 2.10272 | -5.44092 |
| H | 2.93613 | 3.95212 | -1.58434 |
| H | -6.49047 | -1.59021 | -2.41527 |
| H | 2.78749 | -3.51883 | 5.56236 |
| H | 3.96720 | 3.90518 | -3.84895 |
| H | -4.83987 | -0.70356 | 2.24195 |
| H | -4.46619 | 1.75807 | -1.23217 |
| H | -6.95696 | 1.95678 | -1.37229 |
| H | -8.37287 | 0.80198 | 0.30326 |
| H | -7.32704 | -0.52090 | 2.11951 |
| H | -1.23090 | 3.03420 | 0.16855 |
| H | 1.49057 | 2.69481 | 3.49346 |
| H | 2.76820 | 4.61835 | 2.66928 |
| H | 3.12640 | 6.38971 | 0.97269 |
| H | 2.39997 | 7.54358 | -1.08493 |
| H | 0.38741 | 6.77379 | -2.32028 |
| H | -0.91462 | 4.83932 | -1.49161 |
| H | -1.79727 | 1.69972 | 2.04302 |
| H | 0.51390 | 0.63756 | 3.80324 |
| H | -0.77522 | 1.66518 | 4.44077 |
| H | -1.18670 | 0.08709 | 3.75885 |
| H | 3.94295 | -3.46028 | -3.63816 |
| H | 2.01622 | -2.90329 | 0.16800 |
| H | 5.15531 | -5.40178 | -2.72339 |
| H | 3.22508 | -4.81325 | 1.06813 |
| H | 4.80831 | -6.09555 | -0.36171 |
| H | 4.33678 | -1.57856 | 5.49787 |
| H | 1.03261 | -3.74894 | 3.82163 |
| H | -6.40315 | -3.01208 | -0.38397 |
| H | -4.38773 | -0.69073 | -3.38102 |

**(TS-3c$^S$)$^+$**

| | | | |
|---|---|---|---|
| C | -7.04136 | 1.82267 | -0.36105 |
| C | -7.01715 | 1.59292 | -1.73337 |
| C | -5.85746 | 1.13640 | -2.34505 |
| C | -4.69609 | 0.89720 | -1.60657 |
| C | -4.73499 | 1.13267 | -0.23165 |
| C | -5.89487 | 1.59045 | 0.38688 |

| | | | |
|---|---|---|---|
| C | -3.47474 | 0.39642 | -2.34029 |
| C | -2.18512 | 0.26943 | -1.52330 |
| C | -0.96552 | -0.15191 | -2.41996 |
| C | -1.01259 | -1.58700 | -2.85539 |
| C | -1.74681 | -1.99622 | -3.97006 |
| C | -1.83383 | -3.34188 | -4.30846 |
| C | -1.18482 | -4.29846 | -3.53549 |
| C | -0.43000 | -3.89878 | -2.43690 |
| C | -0.33705 | -2.55245 | -2.10233 |
| C | -2.33700 | -0.79344 | -0.46544 |
| N | -1.55415 | -1.03059 | 0.52137 |
| C | -2.14350 | -2.11162 | 1.30446 |
| C | -3.25824 | -2.63242 | 0.37401 |
| O | -3.36142 | -1.61328 | -0.62450 |
| C | -2.61839 | -1.62362 | 2.65532 |
| C | -2.97132 | -2.55688 | 3.63163 |
| C | -3.41547 | -2.14058 | 4.88050 |
| C | -3.50561 | -0.78229 | 5.17134 |
| C | -3.15166 | 0.15260 | 4.20589 |
| C | -2.71124 | -0.26358 | 2.95234 |
| Cu | -0.02786 | 0.15867 | 0.93658 |
| C | 1.92878 | -1.14214 | 2.86184 |
| C | 0.69486 | -1.40263 | 3.60898 |
| N | -1.04931 | 1.80947 | 0.06801 |
| C | -0.82100 | 3.25823 | 0.15590 |
| C | -1.92291 | 3.80775 | -0.77244 |
| O | -2.27497 | 2.67162 | -1.57673 |
| C | -1.81721 | 1.60571 | -0.92740 |
| C | 0.56733 | 3.64598 | -0.29309 |
| C | 1.05424 | 3.22004 | -1.53056 |
| C | 2.28007 | 3.67424 | -1.99804 |
| C | 3.03775 | 4.55653 | -1.23301 |
| C | 2.56692 | 4.97195 | 0.00586 |
| C | 1.33888 | 4.51470 | 0.47437 |
| C | 2.32067 | 0.89305 | 0.54107 |
| O | 1.89644 | 0.16687 | -0.37669 |
| O | 1.66975 | 1.05701 | 1.62973 |
| C | 3.64721 | 1.55624 | 0.41780 |
| C | 4.14656 | 2.34794 | 1.45223 |
| C | 5.38933 | 2.95644 | 1.32399 |
| C | 6.13550 | 2.77358 | 0.16279 |
| C | 5.64160 | 1.97817 | -0.87004 |
| C | 4.39982 | 1.37110 | -0.74308 |
| C | 2.48574 | -1.90811 | 1.83465 |
| C | 3.76698 | -1.53955 | 1.38978 |
| C | 4.39336 | -2.19091 | 0.32476 |
| C | 3.70479 | -3.26312 | -0.32242 |
| C | 2.41447 | -3.64668 | 0.15296 |
| C | 1.81609 | -2.99636 | 1.18443 |
| C | 5.67340 | -1.79281 | -0.13653 |
| C | 6.24992 | -2.43413 | -1.20272 |
| C | 5.56896 | -3.49116 | -1.84009 |
| C | 4.32336 | -3.89989 | -1.41081 |
| H | -4.22971 | -2.73637 | 0.86391 |
| H | -1.58765 | 4.61134 | -1.43153 |
| H | -0.95478 | 0.52743 | -3.28126 |
| H | -3.69616 | -0.58510 | -2.77293 |
| H | -3.26396 | 1.07514 | -3.17481 |
| H | -0.05271 | 0.02473 | -1.83586 |
| H | -0.96887 | 3.59070 | 1.18817 |
| H | -2.82361 | 4.11726 | -0.22851 |
| H | -1.39676 | -2.90116 | 1.45793 |
| H | -2.98791 | -3.56563 | -0.13191 |
| H | -2.89591 | -3.62291 | 3.41586 |
| H | -2.41814 | 0.47512 | 2.20764 |
| H | 0.47070 | 2.52985 | -2.13853 |
| H | 0.97071 | 4.84445 | 1.44503 |
| H | -2.24976 | -1.25218 | -4.58601 |
| H | 0.27416 | -2.22622 | -1.26101 |
| H | -2.40842 | -3.64451 | -5.18139 |
| H | 0.09748 | -4.64586 | -1.84619 |
| H | 4.00095 | 4.90735 | -1.59769 |
| H | -3.85075 | -0.45524 | 6.14980 |
| H | -1.25464 | -5.35198 | -3.79832 |
| H | 3.53565 | 2.49024 | 2.34059 |
| H | 3.98319 | 0.74792 | -1.53137 |
| H | 6.22754 | 1.83887 | -1.77669 |
| H | 7.10733 | 3.25325 | 0.06141 |
| H | 5.77745 | 3.57999 | 2.12693 |
| H | 4.27854 | -0.71157 | 1.87782 |
| H | 0.82579 | -3.29385 | 1.51704 |
| H | 1.91185 | -4.47570 | -0.33983 |
| H | 3.80854 | -4.71525 | -1.91459 |
| H | 6.03561 | -3.99195 | -2.68608 |
| H | 7.23003 | -2.13012 | -1.56228 |
| H | 6.17665 | -0.96537 | 0.35949 |
| H | 2.55710 | -0.34555 | 3.25489 |
| H | 0.04788 | -2.16240 | 3.17038 |
| H | 0.98559 | -1.73186 | 4.62148 |
| H | 0.12839 | -0.47459 | 3.75613 |
| H | -5.85002 | 0.96198 | -3.42048 |
| H | -3.85919 | 0.96106 | 0.39213 |

| | | | |
|---|---|---|---|
| H | -7.90811 | 1.77162 | -2.33198 |
| H | -5.89772 | 1.76061 | 1.46197 |
| H | -7.94914 | 2.17996 | 0.12032 |
| H | -3.68904 | -2.87885 | 5.63145 |
| H | -3.21767 | 1.21613 | 4.42690 |
| H | 3.16005 | 5.65078 | 0.61508 |
| H | 2.64939 | 3.33440 | -2.96335 |

**22c**$^{S}$

| | | | |
|---|---|---|---|
| C | -7.44173 | -0.76529 | 1.51872 |
| C | -7.79231 | -0.68454 | 0.17393 |
| C | -6.81980 | -0.45207 | -0.78821 |
| C | -5.47626 | -0.29618 | -0.43619 |
| C | -5.13840 | -0.37879 | 0.91581 |
| C | -6.11200 | -0.61048 | 1.88482 |
| C | -4.47493 | -0.05001 | -1.53797 |
| C | -3.03158 | 0.20171 | -1.09573 |
| C | -2.10154 | 0.54684 | -2.32161 |
| C | -1.66552 | -0.64959 | -3.11683 |
| C | -2.47855 | -1.21499 | -4.10040 |
| C | -2.07940 | -2.36159 | -4.77820 |
| C | -0.85744 | -2.95694 | -4.48458 |
| C | -0.02848 | -2.38922 | -3.52160 |
| C | -0.42862 | -1.24333 | -2.84430 |
| C | -2.44956 | -1.03463 | -0.46050 |
| N | -1.35608 | -1.12070 | 0.19709 |
| C | -1.07329 | -2.54420 | 0.41650 |
| C | -2.37495 | -3.21682 | -0.05390 |
| O | -3.07260 | -2.16780 | -0.74002 |
| C | -0.67225 | -2.82355 | 1.83800 |
| C | 0.47642 | -3.55882 | 2.12200 |
| C | 0.86403 | -3.77959 | 3.43985 |
| C | 0.10519 | -3.26286 | 4.48380 |
| C | -1.04755 | -2.53130 | 4.20782 |
| C | -1.43479 | -2.31445 | 2.89171 |
| Cu | -0.41836 | 0.55343 | 0.76993 |
| C | 2.50147 | 0.46620 | 2.06578 |
| C | 1.61881 | -0.12431 | 3.13552 |
| N | -2.11654 | 1.67491 | 0.70864 |
| C | -2.27668 | 3.09194 | 1.07861 |
| C | -3.61082 | 3.45316 | 0.40882 |
| O | -3.81402 | 2.38944 | -0.53576 |
| C | -2.96378 | 1.42124 | -0.21240 |
| C | -1.06738 | 3.84696 | 0.58546 |
| C | -0.91046 | 4.12122 | -0.77483 |
| C | 0.23996 | 4.74355 | -1.24062 |
| C | 1.24642 | 5.10304 | -0.34933 |
| C | 1.09535 | 4.83723 | 1.00749 |
| C | -0.05477 | 4.20678 | 1.47144 |
| C | 1.91771 | 1.59196 | -0.06263 |
| O | 1.03906 | 1.51200 | -0.88501 |
| O | 1.58204 | 1.23788 | 1.21134 |
| C | 3.30678 | 1.98281 | -0.34341 |
| C | 4.06214 | 2.73824 | 0.55581 |
| C | 5.39547 | 3.00254 | 0.27801 |
| C | 5.97757 | 2.49694 | -0.88253 |
| C | 5.21878 | 1.76034 | -1.78749 |
| C | 3.87743 | 1.51703 | -1.52964 |
| C | 3.23210 | -0.53274 | 1.21458 |
| C | 4.60343 | -0.58686 | 1.20516 |
| C | 5.29723 | -1.43192 | 0.30827 |
| C | 4.55018 | -2.23832 | -0.60044 |
| C | 3.13494 | -2.17622 | -0.55931 |
| C | 2.49330 | -1.35013 | 0.32465 |
| C | 6.71119 | -1.47021 | 0.25894 |
| C | 7.35685 | -2.27440 | -0.64521 |
| C | 6.61774 | -3.07738 | -1.54061 |
| C | 5.24603 | -3.05992 | -1.51863 |
| H | -3.00240 | -3.54858 | 0.78167 |
| H | -3.59650 | 4.40127 | -0.13412 |
| H | -2.64926 | 1.26455 | -2.94377 |
| H | -4.47187 | -0.90810 | -2.21900 |
| H | -4.79548 | 0.82121 | -2.12048 |
| H | -1.21349 | 1.05336 | -1.92314 |
| H | -2.34077 | 3.17824 | 2.16811 |
| H | -4.45287 | 3.44019 | 1.10917 |
| H | -0.24187 | -2.82097 | -0.24816 |
| H | -2.22160 | -4.04216 | -0.75246 |
| H | 1.08252 | -3.94848 | 1.30549 |
| H | -2.33040 | -1.73207 | 2.67691 |
| H | -1.68767 | 3.83062 | -1.48243 |
| H | -0.17075 | 3.99745 | 2.53424 |
| H | -3.42985 | -0.74689 | -4.34897 |
| H | 0.22325 | -0.78814 | -2.09871 |
| H | -2.72322 | -2.78825 | -5.54446 |
| H | 0.94324 | -2.83245 | -3.30993 |
| H | 2.14705 | 5.59362 | -0.71264 |
| H | 0.40869 | -3.43240 | 5.51482 |
| H | -0.54403 | -3.85174 | -5.01832 |
| H | 3.59869 | 3.12519 | 1.46084 |
| H | 3.26834 | 0.93509 | -2.21779 |

| | | | |
|---|---|---|---|
| H | 5.67619 | 1.37207 | -2.69453 |
| H | 7.02926 | 2.68713 | -1.08667 |
| H | 5.98631 | 3.60088 | 0.96810 |
| H | 5.18029 | 0.06565 | 1.86031 |
| H | 1.40603 | -1.28450 | 0.32988 |
| H | 2.56148 | -2.79212 | -1.25160 |
| H | 4.67521 | -3.67790 | -2.20974 |
| H | 7.14289 | -3.71246 | -2.25090 |
| H | 8.44412 | -2.29802 | -0.67514 |
| H | 7.27777 | -0.84865 | 0.95022 |
| H | 3.21644 | 1.16967 | 2.50398 |
| H | 0.85919 | -0.77518 | 2.68365 |
| H | 2.21486 | -0.73255 | 3.82284 |
| H | 1.11909 | 0.66671 | 3.70562 |
| H | -7.10575 | -0.38966 | -1.83762 |
| H | -4.10715 | -0.25937 | 1.24497 |
| H | -8.83135 | -0.80287 | -0.12673 |
| H | -5.82371 | -0.67018 | 2.93272 |
| H | -8.20256 | -0.94712 | 2.27464 |
| H | 1.76442 | -4.35305 | 3.64972 |
| H | -1.64561 | -2.12866 | 5.02272 |
| H | 1.87167 | 5.13339 | 1.71068 |
| H | 0.35423 | 4.94650 | -2.30306 |

**$(21c^{R})^{+}$**

| | | | |
|---|---|---|---|
| C | 6.99542 | 1.75100 | -1.03365 |
| C | 7.17668 | 1.99227 | 0.32484 |
| C | 6.09713 | 1.93306 | 1.19581 |
| C | 4.81220 | 1.63743 | 0.73515 |
| C | 4.64440 | 1.40091 | -0.63034 |
| C | 5.72453 | 1.45429 | -1.50750 |
| C | 3.68501 | 1.59316 | 1.73744 |
| C | 2.34217 | 1.04917 | 1.23950 |
| C | 1.28393 | 0.98788 | 2.40938 |
| C | 1.37366 | -0.25593 | 3.24537 |
| C | 2.32330 | -0.41902 | 4.25516 |
| C | 2.42549 | -1.62508 | 4.94023 |
| C | 1.57323 | -2.68031 | 4.63090 |
| C | 0.60337 | -2.51669 | 3.64574 |
| C | 0.50315 | -1.31181 | 2.95889 |
| C | 2.52013 | -0.35347 | 0.72115 |
| N | 1.75660 | -1.02667 | -0.06481 |
| C | 2.32036 | -2.38023 | -0.19736 |
| C | 3.58617 | -2.31042 | 0.67884 |
| O | 3.56094 | -0.98431 | 1.21604 |
| C | 2.55959 | -2.74367 | -1.64052 |
| C | 2.18511 | -3.99529 | -2.12591 |
| C | 2.37709 | -4.31872 | -3.46494 |
| C | 2.94783 | -3.39080 | -4.32937 |
| C | 3.33528 | -2.14330 | -3.84873 |
| C | 3.14279 | -1.82142 | -2.51046 |
| Cu | 0.07640 | -0.28112 | -0.79980 |
| C | -0.72012 | -2.17737 | -1.46673 |
| C | -0.70319 | -1.92596 | -2.93321 |
| N | 0.85443 | 1.64311 | -0.65140 |
| C | 0.45636 | 2.88005 | -1.34710 |
| C | 1.62603 | 3.81599 | -0.98766 |
| O | 2.18725 | 3.20687 | 0.18870 |
| C | 1.76174 | 1.95329 | 0.18954 |
| C | -0.87140 | 3.42336 | -0.87703 |
| C | -1.15843 | 3.53338 | 0.48424 |
| C | -2.33944 | 4.12969 | 0.90746 |
| C | -3.24365 | 4.63009 | -0.02404 |
| C | -2.96138 | 4.52806 | -1.38083 |
| C | -1.78349 | 3.92198 | -1.80445 |
| C | -2.19289 | 0.59635 | -0.26676 |
| O | -1.59597 | 0.33426 | 0.78714 |
| O | -1.64472 | 0.38914 | -1.41490 |
| C | -3.58116 | 1.12075 | -0.25575 |
| C | -4.23476 | 1.44613 | -1.44310 |
| C | -5.54529 | 1.90654 | -1.40841 |
| C | -6.20392 | 2.03446 | -0.18899 |
| C | -5.55437 | 1.70097 | 0.99779 |
| C | -4.24407 | 1.24579 | 0.96527 |
| C | -1.90507 | -2.39782 | -0.70007 |
| C | -3.17149 | -2.06047 | -1.17491 |
| C | -4.30302 | -2.13906 | -0.34901 |
| C | -4.14797 | -2.57142 | 1.00440 |
| C | -2.86121 | -2.97543 | 1.45525 |
| C | -1.78043 | -2.89984 | 0.63133 |
| C | -5.58275 | -1.73245 | -0.80681 |
| C | -6.65779 | -1.73733 | 0.04188 |
| C | -6.50089 | -2.15878 | 1.37958 |
| C | -5.27580 | -2.57147 | 1.84946 |
| H | 4.51055 | -2.42449 | 0.10423 |
| H | 1.31798 | 4.83328 | -0.73937 |
| H | 1.42999 | 1.89395 | 3.00940 |
| H | 3.99645 | 0.98139 | 2.58992 |
| H | 3.49829 | 2.60413 | 2.11869 |
| H | 0.28778 | 1.02754 | 1.95369 |
| H | 0.40915 | 2.69610 | -2.42458 |

| | | | |
|---|---|---|---|
| H | 2.41321 | 3.83440 | -1.75146 |
| H | 1.61174 | -3.09581 | 0.24061 |
| H | 3.57687 | -3.01366 | 1.51602 |
| H | 1.72758 | -4.72096 | -1.45333 |
| H | 3.44496 | -0.84419 | -2.13811 |
| H | -0.46328 | 3.14102 | 1.22452 |
| H | -1.56952 | 3.83560 | -2.86909 |
| H | 2.98297 | 0.40556 | 4.52086 |
| H | -0.26800 | -1.15980 | 2.20441 |
| H | 3.16850 | -1.73834 | 5.72696 |
| H | -0.09159 | -3.32669 | 3.42686 |
| H | -4.17143 | 5.09083 | 0.30832 |
| H | 3.09483 | -3.64088 | -5.37783 |
| H | 1.65089 | -3.62067 | 5.17282 |
| H | -3.69718 | 1.34373 | -2.38287 |
| H | -3.71029 | 0.97575 | 1.87337 |
| H | -6.07498 | 1.79581 | 1.94863 |
| H | -7.23097 | 2.39417 | -0.16268 |
| H | -6.05605 | 2.16932 | -2.33282 |
| H | -3.28764 | -1.67587 | -2.18527 |
| H | -0.79440 | -3.19292 | 0.98747 |
| H | -2.75347 | -3.33229 | 2.47801 |
| H | -5.16309 | -2.89799 | 2.88143 |
| H | -7.36185 | -2.15735 | 2.04500 |
| H | -7.63379 | -1.41071 | -0.30933 |
| H | -5.68772 | -1.39310 | -1.83523 |
| H | 0.14286 | -2.71672 | -1.08855 |
| H | -0.95737 | -2.86581 | -3.45008 |
| H | -1.42508 | -1.15973 | -3.22970 |
| H | 0.29844 | -1.64309 | -3.27834 |
| H | 6.25282 | 2.11814 | 2.25800 |
| H | 3.66221 | 1.16767 | -1.04052 |
| H | 8.16748 | 2.22514 | 0.70950 |
| H | 5.56767 | 1.26412 | -2.56787 |
| H | 7.84059 | 1.79450 | -1.71700 |
| H | 2.07814 | -5.29746 | -3.83397 |
| H | 3.78935 | -1.41767 | -4.52034 |
| H | -3.66742 | 4.91030 | -2.11502 |
| H | -2.55795 | 4.19854 | 1.97090 |

**(TS-3c$^{R}$)$^{+}$**

| | | | |
|---|---|---|---|
| C | 7.52338 | 0.39487 | -1.27840 |
| C | 7.81767 | 0.58561 | 0.06862 |
| C | 6.79738 | 0.61204 | 1.00899 |
| C | 5.46134 | 0.44714 | 0.63304 |
| C | 5.18054 | 0.25738 | -0.72127 |
| C | 6.20138 | 0.23213 | -1.66843 |
| C | 4.40834 | 0.47993 | 1.71432 |
| C | 2.95067 | 0.46389 | 1.24468 |
| C | 1.94772 | 0.64052 | 2.44811 |
| C | 1.64432 | -0.63616 | 3.17817 |
| C | 2.53085 | -1.21295 | 4.08903 |
| C | 2.24545 | -2.44412 | 4.67053 |
| C | 1.06828 | -3.11289 | 4.35211 |
| C | 0.16561 | -2.53299 | 3.46529 |
| C | 0.44967 | -1.30116 | 2.88677 |
| C | 2.62183 | -0.85115 | 0.59089 |
| N | 1.58477 | -1.12557 | -0.10470 |
| C | 1.61498 | -2.54736 | -0.43412 |
| C | 2.96974 | -3.00504 | 0.15128 |
| O | 3.44734 | -1.85080 | 0.85271 |
| C | 1.44690 | -2.78534 | -1.91411 |
| C | 0.90106 | -3.98412 | -2.37448 |
| C | 0.76191 | -4.21850 | -3.73826 |
| C | 1.16040 | -3.25108 | -4.65638 |
| C | 1.69604 | -2.04957 | -4.20416 |
| C | 1.83940 | -1.81608 | -2.84015 |
| Cu | 0.31206 | 0.28531 | -0.69039 |
| C | -1.75231 | -1.46848 | -1.70261 |
| C | -1.80725 | -1.18577 | -3.13873 |
| N | 1.85441 | 1.70826 | -0.62311 |
| C | 1.79127 | 3.11528 | -1.05336 |
| C | 2.99557 | 3.73621 | -0.32060 |
| O | 3.34116 | 2.74956 | 0.66537 |
| C | 2.69009 | 1.63876 | 0.33669 |
| C | 0.45154 | 3.69991 | -0.68278 |
| C | 0.09733 | 3.82940 | 0.66115 |
| C | -1.14774 | 4.32980 | 1.01710 |
| C | -2.05068 | 4.71130 | 0.02961 |
| C | -1.70303 | 4.59021 | -1.31099 |
| C | -0.45685 | 4.08553 | -1.66472 |
| C | -1.98633 | 1.13015 | -0.12728 |
| O | -1.33590 | 0.88760 | 0.90078 |
| O | -1.49823 | 0.95346 | -1.29990 |
| C | -3.41590 | 1.53385 | -0.04457 |
| C | -4.12625 | 1.86996 | -1.20009 |
| C | -5.47884 | 2.17274 | -1.12043 |
| C | -6.12881 | 2.12627 | 0.11131 |
| C | -5.42248 | 1.79297 | 1.26504 |
| C | -4.06430 | 1.51023 | 1.18931 |
| C | -2.82195 | -1.62282 | -0.81051 |

| | | | |
|---|---|---|---|
| C | -4.15441 | -1.39055 | -1.18584 |
| C | -5.19625 | -1.49641 | -0.25958 |
| C | -4.88751 | -1.82217 | 1.10052 |
| C | -3.53249 | -2.04536 | 1.47220 |
| C | -2.53346 | -1.95128 | 0.55427 |
| C | -6.54542 | -1.25582 | -0.63153 |
| C | -7.54630 | -1.33707 | 0.29999 |
| C | -7.23785 | -1.65598 | 1.63922 |
| C | -5.93993 | -1.89233 | 2.03250 |
| H | 3.70202 | -3.25736 | -0.62310 |
| H | 2.76435 | 4.67256 | 0.19317 |
| H | 2.37557 | 1.40174 | 3.11107 |
| H | 4.55533 | -0.37443 | 2.38431 |
| H | 4.54667 | 1.38663 | 2.31361 |
| H | 1.00969 | 1.03320 | 2.03396 |
| H | 1.91834 | 3.16635 | -2.13968 |
| H | 3.86645 | 3.87368 | -0.97077 |
| H | 0.78872 | -3.04073 | 0.10049 |
| H | 2.88573 | -3.82820 | 0.86529 |
| H | 0.58027 | -4.74174 | -1.65982 |
| H | 2.24730 | -0.86903 | -2.48821 |
| H | 0.79637 | 3.52290 | 1.44033 |
| H | -0.19022 | 3.97979 | -2.71537 |
| H | 3.44886 | -0.69303 | 4.35802 |
| H | -0.25465 | -0.82060 | 2.20846 |
| H | 2.94442 | -2.88048 | 5.38109 |
| H | -0.76990 | -3.04153 | 3.23483 |
| H | -3.03057 | 5.09501 | 0.30605 |
| H | 1.05348 | -3.43460 | -5.72344 |
| H | 0.84711 | -4.07535 | 4.80904 |
| H | -3.59291 | 1.89282 | -2.14786 |
| H | -3.48297 | 1.24914 | 2.07082 |
| H | -5.93445 | 1.75875 | 2.22483 |
| H | -7.19132 | 2.35427 | 0.17325 |
| H | -6.03177 | 2.44718 | -2.01691 |
| H | -4.39388 | -1.11884 | -2.21073 |
| H | -1.49357 | -2.09163 | 0.84123 |
| H | -3.30863 | -2.27544 | 2.51169 |
| H | -5.71411 | -2.13578 | 3.06860 |
| H | -8.04091 | -1.71531 | 2.37125 |
| H | -8.57910 | -1.15256 | 0.01421 |
| H | -6.76581 | -1.00022 | -1.66606 |
| H | -0.77283 | -1.75140 | -1.31886 |
| H | -1.62373 | -2.13413 | -3.67243 |
| H | -2.75016 | -0.75048 | -3.47476 |
| H | -0.97940 | -0.52274 | -3.40893 |
| H | 7.03916 | 0.76513 | 2.06022 |
| H | 4.15719 | 0.12946 | -1.07067 |
| H | 8.84949 | 0.71662 | 0.38832 |
| H | 5.95598 | 0.08220 | -2.71818 |
| H | 8.32133 | 0.37468 | -2.01750 |
| H | 0.34102 | -5.16001 | -4.08522 |
| H | 2.00703 | -1.28873 | -4.91732 |
| H | -2.40830 | 4.88495 | -2.08535 |
| H | -1.41778 | 4.41491 | 2.06731 |

**22c$^{R}$**

| | | | |
|---|---|---|---|
| C | 7.51494 | -0.22199 | -1.92507 |
| C | 7.92403 | -0.41022 | -0.60817 |
| C | 6.98334 | -0.49935 | 0.40821 |
| C | 5.61505 | -0.40858 | 0.13894 |
| C | 5.21840 | -0.22107 | -1.18630 |
| C | 6.15948 | -0.12629 | -2.20874 |
| C | 4.65582 | -0.52484 | 1.29873 |
| C | 3.19442 | -0.16672 | 1.01582 |
| C | 2.34185 | -0.09065 | 2.33641 |
| C | 1.97044 | -1.42831 | 2.90694 |
| C | 2.86502 | -2.16907 | 3.68168 |
| C | 2.51736 | -3.42837 | 4.15734 |
| C | 1.26633 | -3.96288 | 3.86903 |
| C | 0.35847 | -3.22470 | 3.11606 |
| C | 0.70708 | -1.96603 | 2.64061 |
| C | 2.54434 | -1.20378 | 0.13832 |
| N | 1.36587 | -1.13230 | -0.34735 |
| C | 1.06452 | -2.40461 | -1.00329 |
| C | 2.43339 | -3.11021 | -0.99103 |
| O | 3.20810 | -2.33242 | -0.06306 |
| C | 0.44058 | -2.17090 | -2.35241 |
| C | -0.68002 | -2.89170 | -2.75965 |
| C | -1.28034 | -2.62737 | -3.98735 |
| C | -0.76436 | -1.63721 | -4.81705 |
| C | 0.35690 | -0.91330 | -4.41813 |
| C | 0.95600 | -1.17862 | -3.19244 |
| Cu | 0.48211 | 0.65834 | -0.54025 |
| C | -2.30775 | 0.31913 | -1.97337 |
| C | -2.93805 | 1.11008 | -3.08525 |
| N | 2.23948 | 1.66864 | -0.39522 |
| C | 2.39371 | 3.13369 | -0.44648 |
| C | 3.73382 | 3.34593 | 0.27457 |
| O | 3.95810 | 2.09666 | 0.94952 |
| C | 3.10496 | 1.22168 | 0.43130 |

| | | | |
|---|---|---|---|
| C | 1.18224 | 3.75278 | 0.20550 |
| C | 1.03447 | 3.72783 | 1.59374 |
| C | -0.12070 | 4.21802 | 2.18846 |
| C | -1.14306 | 4.73788 | 1.40067 |
| C | -1.00374 | 4.76559 | 0.01722 |
| C | 0.15303 | 4.27161 | -0.57749 |
| C | -1.92173 | 1.40281 | 0.25792 |
| O | -1.04857 | 1.23829 | 1.07905 |
| O | -1.56433 | 1.21868 | -1.04374 |
| C | -3.30495 | 1.77601 | 0.57911 |
| C | -4.04477 | 2.63157 | -0.23888 |
| C | -5.36758 | 2.90854 | 0.07616 |
| C | -5.95375 | 2.32028 | 1.19447 |
| C | -5.20761 | 1.48819 | 2.02453 |
| C | -3.87696 | 1.22951 | 1.73011 |
| C | -3.19069 | -0.60074 | -1.18126 |
| C | -4.55893 | -0.60244 | -1.28760 |
| C | -5.36095 | -1.38146 | -0.41986 |
| C | -4.73009 | -2.18270 | 0.57572 |
| C | -3.31414 | -2.19314 | 0.64277 |
| C | -2.56681 | -1.42873 | -0.21141 |
| C | -6.77414 | -1.35349 | -0.48593 |
| C | -7.52925 | -2.08371 | 0.39647 |
| C | -6.90452 | -2.87817 | 1.38145 |
| C | -5.53570 | -2.92879 | 1.46744 |
| H | 2.93614 | -3.06888 | -1.96486 |
| H | 3.71975 | 4.14499 | 1.01976 |
| H | 2.91776 | 0.50609 | 3.05376 |
| H | 4.68000 | -1.55052 | 1.68417 |
| H | 5.00370 | 0.13008 | 2.10561 |
| H | 1.42452 | 0.46491 | 2.09905 |
| H | 2.44834 | 3.45548 | -1.49157 |
| H | 4.56675 | 3.50167 | -0.41924 |
| H | 0.34883 | -2.95012 | -0.37077 |
| H | 2.40168 | -4.14166 | -0.63470 |
| H | -1.10170 | -3.65022 | -2.10165 |
| H | 1.82223 | -0.59867 | -2.87372 |
| H | 1.82392 | 3.30764 | 2.21813 |
| H | 0.26085 | 4.29380 | -1.66135 |
| H | 3.83949 | -1.75076 | 3.92896 |
| H | -0.00553 | -1.37676 | 2.06318 |
| H | 3.22434 | -3.99165 | 4.76280 |
| H | -0.63155 | -3.62810 | 2.91025 |
| H | -2.04870 | 5.12181 | 1.86555 |
| H | -1.23404 | -1.42994 | -5.77645 |
| H | 0.99364 | -4.94697 | 4.24442 |
| H | -3.57171 | 3.09024 | -1.10338 |
| H | -3.27757 | 0.57517 | 2.35878 |
| H | -5.66746 | 1.03655 | 2.90053 |
| H | -6.99753 | 2.52142 | 1.42671 |
| H | -5.94590 | 3.58396 | -0.55056 |
| H | -5.05982 | 0.03075 | -2.01675 |
| H | -1.47961 | -1.41433 | -0.13465 |
| H | -2.82926 | -2.81428 | 1.39435 |
| H | -5.05252 | -3.54257 | 2.22580 |
| H | -7.51518 | -3.45397 | 2.07372 |
| H | -8.61528 | -2.05433 | 0.33941 |
| H | -7.25252 | -0.73792 | -1.24587 |
| H | -1.48193 | -0.26511 | -2.39012 |
| H | -3.26014 | 0.41625 | -3.86989 |
| H | -3.80745 | 1.68727 | -2.75968 |
| H | -2.20094 | 1.79320 | -3.51916 |
| H | 7.31495 | -0.64148 | 1.43623 |
| H | 4.16501 | -0.14084 | -1.45008 |
| H | 8.98339 | -0.48491 | -0.37120 |
| H | 5.82568 | 0.02088 | -3.23420 |
| H | 8.24998 | -0.14976 | -2.72374 |
| H | -2.15795 | -3.19345 | -4.29210 |
| H | 0.76482 | -0.13887 | -5.06450 |
| H | -1.79619 | 5.18227 | -0.60169 |
| H | -0.22681 | 4.18982 | 3.27061 |

**3a**$^{S}$

| | | | |
|---|---|---|---|
| H | -0.34611 | -0.42267 | -0.93980 |
| C | -1.20510 | 0.05296 | -0.46995 |
| C | -1.12230 | 1.35923 | -0.05584 |
| C | -2.24506 | 1.96337 | 0.55929 |
| C | -3.40830 | 1.26364 | 0.74011 |
| C | -3.51806 | -0.08518 | 0.32246 |
| C | -2.38970 | -0.70127 | -0.29127 |
| C | -2.48954 | -2.05146 | -0.70528 |
| C | -3.65121 | -2.75825 | -0.52245 |
| C | -4.76889 | -2.14710 | 0.08506 |
| C | -4.70211 | -0.84047 | 0.49824 |
| H | -2.17381 | 2.99596 | 0.89984 |
| H | -4.26444 | 1.73608 | 1.21929 |
| H | -5.56137 | -0.36519 | 0.96921 |
| H | -5.68476 | -2.71787 | 0.22566 |
| H | -3.71441 | -3.79578 | -0.84492 |
| H | -1.62466 | -2.52056 | -1.17197 |
| C | 0.11806 | 2.18853 | -0.28379 |

| | | | |
|---|---|---|---|
| H | 0.29653 | 2.79305 | 0.61440 |
| C | 0.00729 | 3.07481 | -1.50731 |
| H | -0.85154 | 3.74692 | -1.40841 |
| H | 0.91463 | 3.67686 | -1.62745 |
| H | -0.13678 | 2.46478 | -2.40595 |
| O | 1.25844 | 1.35369 | -0.48566 |
| O | 1.48436 | 1.10623 | 1.73038 |
| C | 1.84013 | 0.84472 | 0.61192 |
| C | 2.96026 | -0.07249 | 0.28140 |
| C | 3.34730 | -0.33146 | -1.03467 |
| C | 3.63014 | -0.68709 | 1.34017 |
| C | 4.39947 | -1.20279 | -1.28571 |
| C | 4.68033 | -1.55734 | 1.08509 |
| C | 5.06511 | -1.81547 | -0.22801 |
| H | 2.81890 | 0.15506 | -1.85006 |
| H | 3.30517 | -0.46552 | 2.35421 |
| H | 4.70281 | -1.40560 | -2.31089 |
| H | 5.20169 | -2.03767 | 1.91061 |
| H | 5.88873 | -2.49848 | -0.42822 |

**3a$^{R}$**

| | | | |
|---|---|---|---|
| H | 0.34611 | -0.42267 | -0.93979 |
| C | 1.20510 | 0.05296 | -0.46995 |
| C | 1.12230 | 1.35922 | -0.05584 |
| C | 2.24506 | 1.96337 | 0.55929 |
| C | 3.40830 | 1.26365 | 0.74011 |
| C | 3.51806 | -0.08518 | 0.32246 |
| C | 2.38970 | -0.70127 | -0.29127 |
| C | 2.48954 | -2.05147 | -0.70528 |
| C | 3.65121 | -2.75825 | -0.52245 |
| C | 4.76890 | -2.14710 | 0.08506 |
| C | 4.70211 | -0.84047 | 0.49824 |
| H | 2.17381 | 2.99596 | 0.89984 |
| H | 4.26445 | 1.73608 | 1.21928 |
| H | 5.56137 | -0.36519 | 0.96921 |
| H | 5.68476 | -2.71787 | 0.22566 |
| H | 3.71441 | -3.79579 | -0.84492 |
| H | 1.62466 | -2.52056 | -1.17196 |
| C | -0.11806 | 2.18853 | -0.28379 |
| H | -0.29653 | 2.79305 | 0.61440 |
| C | -0.00729 | 3.07481 | -1.50731 |
| H | -0.91463 | 3.67686 | -1.62744 |
| H | 0.85154 | 3.74692 | -1.40841 |
| H | 0.13678 | 2.46478 | -2.40595 |
| O | -1.25844 | 1.35368 | -0.48566 |
| O | -1.48436 | 1.10623 | 1.73038 |
| C | -1.84013 | 0.84472 | 0.61192 |
| C | -2.96027 | -0.07249 | 0.28140 |
| C | -3.34730 | -0.33146 | -1.03467 |
| C | -3.63014 | -0.68709 | 1.34017 |
| C | -4.39947 | -1.20279 | -1.28571 |
| C | -4.68033 | -1.55734 | 1.08509 |
| C | -5.06511 | -1.81547 | -0.22801 |
| H | -2.81890 | 0.15506 | -1.85006 |
| H | -3.30517 | -0.46552 | 2.35421 |
| H | -4.70281 | -1.40560 | -2.31089 |
| H | -5.20169 | -2.03766 | 1.91061 |
| H | -5.88873 | -2.49848 | -0.42822 |

**16$^{+}$**

| | | | |
|---|---|---|---|
| C | 2.96593 | -3.63869 | 1.01002 |
| H | 3.33710 | -4.56932 | 0.58726 |
| C | 2.27358 | -3.64433 | 2.21223 |
| H | 2.09997 | -4.58338 | 2.73325 |
| O | 1.45148 | 2.15523 | 1.55242 |
| N | 0.31692 | 0.24758 | 1.33316 |
| C | 1.21253 | 0.00396 | 2.47171 |
| H | 0.61853 | -0.18870 | 3.37235 |
| C | 0.57925 | 1.40645 | 0.87550 |
| C | 1.97038 | -1.24970 | 2.09421 |
| C | 2.01118 | 1.32051 | 2.58268 |
| H | 1.88475 | 1.82626 | 3.54328 |
| H | 3.08081 | 1.19959 | 2.37327 |
| C | 2.73617 | -1.23253 | 0.90596 |
| H | 3.02966 | -0.27989 | 0.45307 |
| C | 3.20592 | -2.42879 | 0.35353 |
| H | 3.80541 | -2.39979 | -0.55410 |
| C | 1.76124 | -2.45808 | 2.74282 |
| H | 1.16768 | -2.48381 | 3.65497 |
| C | 1.14278 | 2.73662 | -1.15906 |
| H | 0.71814 | 3.04756 | -2.12138 |
| H | 1.40457 | 3.64747 | -0.61075 |
| C | 2.37856 | 1.89858 | -1.35668 |
| C | 3.52462 | 2.15381 | -0.59596 |
| H | 3.51101 | 2.97204 | 0.12145 |
| C | 2.42903 | 0.86534 | -2.29901 |
| H | 1.57665 | 0.68311 | -2.94930 |
| C | 4.67691 | 1.39131 | -0.75450 |
| H | 5.56184 | 1.61941 | -0.16387 |
| C | 3.57521 | 0.08883 | -2.44858 |

| | | | |
|---|---|---|---|
| H | 3.59642 | -0.70174 | -3.19641 |
| C | 4.70207 | 0.34731 | -1.67474 |
| H | 5.60473 | -0.24621 | -1.80497 |
| Cu | 0.99021 | -1.10474 | -0.28989 |
| C | -1.04801 | 3.09890 | 0.08399 |
| H | -0.51880 | 3.79596 | 0.74490 |
| H | -1.35115 | 3.65509 | -0.80877 |
| C | -2.25064 | 2.51618 | 0.77579 |
| C | -3.45937 | 2.34847 | 0.09526 |
| H | -3.54216 | 2.69130 | -0.93382 |
| C | -2.17830 | 2.10899 | 2.11068 |
| H | -1.26242 | 2.27990 | 2.67565 |
| C | -4.55227 | 1.75712 | 0.71774 |
| H | -5.48608 | 1.63788 | 0.17238 |
| C | -3.26694 | 1.51168 | 2.73501 |
| H | -3.19305 | 1.20504 | 3.77647 |
| C | -4.45505 | 1.32496 | 2.03567 |
| H | -5.31013 | 0.86234 | 2.52430 |
| C | 0.00301 | 2.03572 | -0.35938 |
| O | -1.58576 | 1.51309 | -2.05999 |
| N | -0.39473 | -0.22932 | -1.39283 |
| C | -1.40573 | -0.81253 | -2.27701 |
| H | -0.92046 | -1.41637 | -3.05176 |
| C | -0.65321 | 1.01871 | -1.26021 |
| C | -2.35530 | -1.68354 | -1.48666 |
| C | -2.06138 | 0.44162 | -2.89052 |
| H | -1.73840 | 0.64285 | -3.91773 |
| H | -3.15317 | 0.42937 | -2.83959 |
| C | -2.68729 | -1.37141 | -0.16600 |
| H | -2.21186 | -0.53601 | 0.34645 |
| C | -3.62129 | -2.14204 | 0.51748 |
| H | -3.87521 | -1.88491 | 1.54386 |
| C | -4.22591 | -3.22892 | -0.10432 |
| H | -4.95563 | -3.83047 | 0.43361 |
| C | -3.89055 | -3.54917 | -1.41600 |
| H | -4.35436 | -4.40255 | -1.90615 |
| C | -2.95790 | -2.78065 | -2.10233 |
| H | -2.69682 | -3.03676 | -3.12888 |

**$^{3}$(TS-3$^{S}$)’$^{+}$**

| | | | |
|---|---|---|---|
| C | -1.55066 | 0.34528 | 3.06585 |
| O | -2.11457 | -0.24223 | 1.52603 |
| C | -2.90115 | 0.62256 | 0.86150 |
| O | -2.21340 | 1.50950 | 0.20068 |
| Cu | -0.68852 | 0.30619 | 0.09770 |
| N | 0.37210 | 1.46783 | -1.08491 |
| C | 0.05422 | 2.89465 | -1.25470 |
| C | 0.88551 | 3.25833 | -2.49994 |
| O | 1.53920 | 2.02675 | -2.87344 |
| C | 1.16646 | 1.09712 | -2.01046 |
| C | 0.36766 | 3.66686 | -0.00103 |
| C | -0.66506 | 4.18267 | 0.77955 |
| C | -0.38022 | 4.81931 | 1.98374 |
| C | 0.93629 | 4.94330 | 2.41445 |
| C | 1.97269 | 4.43896 | 1.63325 |
| C | 1.68775 | 3.80628 | 0.42989 |
| C | 1.57233 | -0.30225 | -2.34339 |
| C | 0.57787 | -0.77563 | -3.49089 |
| C | -0.82959 | -0.27283 | -3.34504 |
| C | -1.19734 | 0.93754 | -3.94013 |
| C | -2.46138 | 1.47834 | -3.73152 |
| C | -3.38118 | 0.81432 | -2.92633 |
| C | -3.04164 | -0.40930 | -2.36083 |
| C | -1.77735 | -0.95053 | -2.57260 |
| C | -0.11664 | 0.06805 | 3.03044 |
| C | 0.42695 | -1.11959 | 3.48793 |
| C | 1.78808 | -1.43507 | 3.27105 |
| C | 2.61114 | -0.50932 | 2.56204 |
| C | 2.04962 | 0.72479 | 2.14499 |
| C | 0.73326 | 1.01051 | 2.38081 |
| C | 2.35335 | -2.66474 | 3.69149 |
| C | 3.66741 | -2.96190 | 3.42125 |
| C | 4.47822 | -2.04365 | 2.72098 |
| C | 3.95890 | -0.84110 | 2.30419 |
| C | -2.43310 | -0.33990 | 4.05279 |
| N | 0.50186 | -1.23281 | -0.27014 |
| C | 0.47697 | -2.59309 | 0.33928 |
| C | 1.85781 | -3.09045 | -0.04902 |
| O | 2.13179 | -2.34817 | -1.25875 |
| C | 1.38450 | -1.25860 | -1.20201 |
| C | -0.68579 | -3.35778 | -0.24672 |
| C | -0.54640 | -4.14586 | -1.39192 |
| C | -1.66264 | -4.72499 | -1.98697 |
| C | -2.92864 | -4.51509 | -1.45069 |
| C | -3.07301 | -3.74149 | -0.30296 |
| C | -1.95674 | -3.17312 | 0.29954 |
| C | 3.01479 | -0.36616 | -2.88747 |
| C | 4.11559 | -0.06707 | -1.90253 |
| C | 4.94548 | -1.09176 | -1.44192 |
| C | 6.02566 | -0.81791 | -0.61065 |
| C | 6.28459 | 0.48804 | -0.20855 |
| C | 5.45487 | 1.51660 | -0.64375 |
| C | 4.38518 | 1.24158 | -1.48929 |

| | | | |
|---|---|---|---|
| C | -4.32090 | 0.53969 | 0.89557 |
| C | -4.98660 | -0.52792 | 1.53871 |
| C | -6.36694 | -0.61399 | 1.50057 |
| C | -7.12007 | 0.34948 | 0.82707 |
| C | -6.47204 | 1.40493 | 0.18417 |
| C | -5.09190 | 1.50473 | 0.21114 |
| H | 1.91539 | -4.15580 | -0.27525 |
| H | 1.65989 | 4.00559 | -2.30270 |
| H | 3.15622 | -1.37025 | -3.29901 |
| H | 0.61513 | -1.87160 | -3.51032 |
| H | 1.01152 | -0.41311 | -4.42937 |
| H | 3.06372 | 0.33714 | -3.72597 |
| H | -1.02091 | 2.95577 | -1.45495 |
| H | 0.27450 | 3.57960 | -3.34780 |
| H | 0.36595 | -2.49995 | 1.41965 |
| H | 2.62214 | -2.81391 | 0.68546 |
| H | 0.43192 | -4.30283 | -1.84325 |
| H | -2.07439 | -2.55612 | 1.18680 |
| H | -1.69470 | 4.05535 | 0.45363 |
| H | 2.50255 | 3.39408 | -0.16631 |
| H | 4.75319 | -2.11396 | -1.76253 |
| H | 3.78578 | 2.06266 | -1.87672 |
| H | 6.67418 | -1.62865 | -0.28417 |
| H | 5.66038 | 2.54400 | -0.34886 |
| H | -0.48415 | 1.45565 | -4.58094 |
| H | -1.53339 | -1.91799 | -2.14615 |
| H | -2.73394 | 2.41726 | -4.21009 |
| H | -3.76361 | -0.94792 | -1.74984 |
| H | 1.15755 | 5.43715 | 3.35820 |
| H | -3.80132 | -4.96054 | -1.92327 |
| H | 7.13556 | 0.70601 | 0.43354 |
| H | -4.36893 | 1.23551 | -2.75527 |
| H | -4.40140 | -1.28777 | 2.05159 |
| H | -4.57898 | 2.32355 | -0.28763 |
| H | -7.05617 | 2.15901 | -0.34008 |
| H | -8.20492 | 0.27698 | 0.80296 |
| H | -6.86801 | -1.44189 | 1.99863 |
| H | -0.19553 | -1.84508 | 4.00840 |
| H | 0.31888 | 1.97293 | 2.08110 |
| H | 2.68632 | 1.44599 | 1.63687 |
| H | 4.57664 | -0.12776 | 1.76522 |
| H | 5.51845 | -2.28835 | 2.51580 |
| H | 4.08943 | -3.90791 | 3.75426 |
| H | 1.72979 | -3.37175 | 4.23646 |
| H | -1.76953 | 1.40727 | 2.92795 |
| H | -3.47266 | -0.02402 | 3.93000 |
| H | -2.38652 | -1.42968 | 3.94840 |
| H | -2.11786 | -0.08317 | 5.07313 |
| H | 3.00397 | 4.53707 | 1.96586 |
| H | -1.19134 | 5.21901 | 2.58882 |
| H | -4.05978 | -3.57867 | 0.12634 |
| H | -1.54269 | -5.33734 | -2.87809 |

**$^{3}$(TS-3$^{R}$)’$^{+}$**

| | | | |
|---|---|---|---|
| C | -1.28085 | 1.20294 | -2.64983 |
| O | -1.85672 | 1.10604 | -1.00568 |
| C | -2.88012 | 0.26379 | -0.77444 |
| O | -2.47856 | -0.90923 | -0.37359 |
| Cu | -0.72924 | -0.24217 | 0.09444 |
| N | 0.03729 | -2.00676 | 0.47740 |
| C | -0.62067 | -3.23908 | 0.02781 |
| C | 0.19902 | -4.31722 | 0.76352 |
| O | 1.05667 | -3.57108 | 1.65299 |
| C | 0.90234 | -2.28903 | 1.37053 |
| C | -0.63380 | -3.35657 | -1.47166 |
| C | -1.82321 | -3.61790 | -2.14729 |
| C | -1.83150 | -3.72797 | -3.53367 |
| C | -0.65324 | -3.56296 | -4.25413 |
| C | 0.53760 | -3.29328 | -3.58523 |
| C | 0.54751 | -3.19691 | -2.19873 |
| C | 1.64809 | -1.34015 | 2.25446 |
| C | 0.87363 | -1.35531 | 3.63205 |
| C | -0.61781 | -1.31000 | 3.45229 |
| C | -1.35408 | -2.49642 | 3.39859 |
| C | -2.70606 | -2.47985 | 3.07073 |
| C | -3.34401 | -1.27625 | 2.79019 |
| C | -2.63296 | -0.08468 | 2.88108 |
| C | -1.28268 | -0.10333 | 3.21779 |
| C | 0.13943 | 1.51419 | -2.50413 |
| C | 0.57708 | 2.82674 | -2.56930 |
| C | 1.93732 | 3.16528 | -2.39017 |
| C | 2.87970 | 2.12623 | -2.11871 |
| C | 2.41346 | 0.78759 | -2.04119 |
| C | 1.09234 | 0.49035 | -2.23903 |
| C | 2.39515 | 4.50477 | -2.45043 |
| C | 3.71989 | 4.80198 | -2.25126 |
| C | 4.65056 | 3.77485 | -1.98722 |
| C | 4.23867 | 2.46546 | -1.92522 |
| C | -1.69661 | -0.06819 | -3.30580 |
| N | 0.80948 | 0.64868 | 0.95926 |
| C | 1.12696 | 2.10072 | 0.95416 |
| C | 2.54711 | 2.08045 | 1.50133 |
| O | 2.58968 | 0.84694 | 2.24965 |

| | | | |
|---|---|---|---|
| C | 1.64122 | 0.07246 | 1.74432 |
| C | 0.10923 | 2.84259 | 1.78858 |
| C | 0.30605 | 3.08580 | 3.15000 |
| C | -0.69539 | 3.67946 | 3.91077 |
| C | -1.90563 | 4.03062 | 3.32137 |
| C | -2.10410 | 3.80138 | 1.96374 |
| C | -1.09906 | 3.21742 | 1.20114 |
| C | 3.10775 | -1.81573 | 2.44390 |
| C | 3.93993 | -1.72724 | 1.18921 |
| C | 4.88681 | -0.71281 | 1.02944 |
| C | 5.66436 | -0.63583 | -0.12158 |
| C | 5.49884 | -1.56488 | -1.14334 |
| C | 4.56119 | -2.58202 | -0.99684 |
| C | 3.79638 | -2.66545 | 0.16168 |
| C | -4.23147 | 0.64481 | -0.99421 |
| C | -4.56313 | 1.94650 | -1.43109 |
| C | -5.88720 | 2.29867 | -1.62752 |
| C | -6.90830 | 1.37737 | -1.39219 |
| C | -6.59016 | 0.09025 | -0.95442 |
| C | -5.27231 | -0.27965 | -0.75624 |
| H | 2.79377 | 2.90517 | 2.17068 |
| H | 0.83867 | -4.89625 | 0.08853 |
| H | 3.55469 | -1.20669 | 3.23508 |
| H | 1.24500 | -0.50797 | 4.22016 |
| H | 1.17244 | -2.27612 | 4.14516 |
| H | 3.06534 | -2.84897 | 2.80433 |
| H | -1.65325 | -3.19673 | 0.39456 |
| H | -0.40570 | -4.99655 | 1.36926 |
| H | 1.09815 | 2.45603 | -0.07785 |
| H | 3.29587 | 2.00523 | 0.70461 |
| H | 1.23548 | 2.79632 | 3.63793 |
| H | -1.25895 | 3.03422 | 0.14209 |
| H | -2.74959 | -3.71493 | -1.58454 |
| H | 1.48009 | -2.97671 | -1.67800 |
| H | 5.03079 | 0.01028 | 1.82991 |
| H | 3.10759 | -3.49835 | 0.28580 |
| H | 6.41507 | 0.14700 | -0.21229 |
| H | 4.44203 | -3.33071 | -1.77784 |
| H | -0.85837 | -3.44338 | 3.61227 |
| H | -0.74415 | 0.83582 | 3.30162 |
| H | -3.26539 | -3.41297 | 3.03704 |
| H | -3.12540 | 0.86662 | 2.68849 |
| H | -0.66201 | -3.64223 | -5.33916 |
| H | -2.68960 | 4.49039 | 3.91932 |
| H | 6.11035 | -1.50837 | -2.04142 |
| H | -4.39593 | -1.26311 | 2.51419 |
| H | -3.76921 | 2.67148 | -1.59832 |
| H | -5.01414 | -1.28101 | -0.41956 |
| H | -7.38530 | -0.62968 | -0.76985 |
| H | -7.94669 | 1.66132 | -1.54693 |
| H | -6.13215 | 3.30420 | -1.96428 |
| H | -0.13807 | 3.62337 | -2.77208 |
| H | 0.76884 | -0.54829 | -2.19411 |
| H | 3.12618 | -0.00714 | -1.82447 |
| H | 4.95229 | 1.66732 | -1.73087 |
| H | 5.69923 | 4.02476 | -1.84052 |
| H | 4.05907 | 5.83444 | -2.30154 |
| H | 1.67750 | 5.29641 | -2.65854 |
| H | -1.87002 | 2.06635 | -2.96396 |
| H | -1.47402 | -0.00989 | -4.37883 |
| H | -1.18296 | -0.94311 | -2.89568 |
| H | -2.77534 | -0.22667 | -3.19870 |
| H | -2.76454 | -3.93315 | -4.05406 |
| H | 1.46031 | -3.15729 | -4.14552 |
| H | -3.04487 | 4.07972 | 1.49257 |
| H | -0.53068 | 3.86423 | 4.97013 |

**$(21^{R})_{3t}^{+}$**

| | | | |
|---|---|---|---|
| C | 5.60492 | -3.87016 | -0.68287 |
| C | 4.51889 | -3.60238 | -1.51183 |
| C | 3.29827 | -3.22307 | -0.96604 |
| C | 3.15139 | -3.10058 | 0.41899 |
| C | 4.23795 | -3.39477 | 1.24341 |
| C | 5.45941 | -3.77357 | 0.69636 |
| C | 1.83430 | -2.65411 | 0.98093 |
| C | 1.68576 | -1.10451 | 1.22761 |
| C | 2.59042 | -0.65578 | 2.40702 |
| C | 2.62875 | 0.83321 | 2.63270 |
| C | 1.54487 | 1.52299 | 3.18519 |
| C | 1.59863 | 2.89963 | 3.37889 |
| C | 2.74320 | 3.61077 | 3.03455 |
| C | 3.83127 | 2.93651 | 2.49156 |
| C | 3.77059 | 1.56268 | 2.29039 |
| C | 0.24331 | -0.92467 | 1.58506 |
| N | -0.73156 | -0.58574 | 0.83381 |
| C | -1.97155 | -0.71596 | 1.60458 |
| C | -1.44888 | -0.98944 | 3.03095 |
| O | -0.05778 | -1.26562 | 2.83426 |
| C | -2.87826 | -1.80711 | 1.09691 |
| C | -2.37414 | -2.95160 | 0.48313 |
| C | -3.23279 | -3.97853 | 0.10750 |
| C | -4.59924 | -3.87233 | 0.34567 |
| C | -5.10605 | -2.73466 | 0.96595 |

| | | | |
|---|---|---|---|
| C | -4.24716 | -1.70840 | 1.34007 |
| Cu | -0.55965 | -0.06254 | -1.13508 |
| C | -1.29071 | 1.27322 | -2.60013 |
| C | -0.37049 | 2.26894 | -3.18685 |
| N | 1.42929 | 0.29391 | -0.87958 |
| C | 2.38702 | 0.96575 | -1.76663 |
| C | 3.72115 | 0.31126 | -1.37556 |
| O | 3.42806 | -0.27406 | -0.10862 |
| C | 2.11112 | -0.32783 | 0.01401 |
| C | 2.37207 | 2.46516 | -1.58872 |
| C | 1.89552 | 3.06265 | -0.42367 |
| C | 1.87357 | 4.45073 | -0.30900 |
| C | 2.33605 | 5.25015 | -1.34738 |
| C | 2.83580 | 4.65643 | -2.50373 |
| C | 2.85096 | 3.27306 | -2.62210 |
| O | -1.64891 | -1.35502 | -2.23235 |
| C | -0.89227 | -2.38863 | -2.30251 |
| O | 0.21409 | -2.42583 | -1.75475 |
| C | -2.17591 | 1.56564 | -1.51532 |
| C | -3.39131 | 0.86231 | -1.44818 |
| C | -4.38340 | 1.21613 | -0.52473 |
| C | -4.10511 | 2.24218 | 0.43289 |
| C | -2.83723 | 2.89210 | 0.40381 |
| C | -1.90637 | 2.58652 | -0.54353 |
| C | -5.65657 | 0.58976 | -0.53091 |
| C | -6.61344 | 0.95918 | 0.37848 |
| C | -6.32792 | 1.95185 | 1.33964 |
| C | -5.10121 | 2.57786 | 1.37054 |
| H | -1.91204 | -1.85741 | 3.50596 |
| H | 4.54049 | 1.02284 | -1.25057 |
| H | 2.23518 | -1.17723 | 3.30155 |
| H | 1.03828 | -2.94365 | 0.28777 |
| H | 1.64355 | -3.12275 | 1.95365 |
| H | 3.59930 | -1.01422 | 2.18868 |
| H | 2.14628 | 0.72277 | -2.80711 |
| H | 4.01950 | -0.49716 | -2.05302 |
| H | -2.51185 | 0.23696 | 1.56906 |
| H | -1.52979 | -0.11898 | 3.69286 |
| H | -1.30822 | -3.03526 | 0.28226 |
| H | -4.64368 | -0.81537 | 1.82365 |
| H | 1.53300 | 2.44959 | 0.39970 |
| H | 3.22455 | 2.81510 | -3.53821 |
| H | 0.66254 | 0.97457 | 3.50571 |
| H | 4.62521 | 1.03685 | 1.86856 |
| H | 0.74995 | 3.41542 | 3.82461 |
| H | 4.73427 | 3.48240 | 2.22636 |
| H | 2.31510 | 6.33399 | -1.25564 |
| H | -5.26878 | -4.67795 | 0.05175 |
| H | 2.79180 | 4.68525 | 3.19982 |
| H | -3.56488 | 0.05269 | -2.15393 |
| H | -0.95382 | 3.11030 | -0.56616 |
| H | -2.63052 | 3.66279 | 1.14390 |
| H | -4.89688 | 3.35057 | 2.10903 |
| H | -7.09266 | 2.23106 | 2.06172 |
| H | -7.59254 | 0.48592 | 0.36787 |
| H | -5.85308 | -0.18912 | -1.26455 |
| H | -1.63698 | 0.49160 | -3.27471 |
| H | 0.46263 | 1.80012 | -3.71807 |
| H | -0.95374 | 2.80034 | -3.95811 |
| H | 0.00665 | 3.01429 | -2.48564 |
| H | 2.43559 | -3.02853 | -1.60312 |
| H | 4.12595 | -3.34075 | 2.32579 |
| H | 4.62076 | -3.70481 | -2.59093 |
| H | 6.29766 | -4.00358 | 1.35096 |
| H | 6.55893 | -4.17154 | -1.11077 |
| H | 3.20667 | 5.27445 | -3.31873 |
| H | 1.49948 | 4.90412 | 0.60661 |
| H | -6.17347 | -2.64643 | 1.15914 |
| H | -2.83043 | -4.86719 | -0.37467 |
| C | -1.41898 | -3.54893 | -3.10290 |
| H | -2.50008 | -3.65005 | -2.97135 |
| H | -1.23057 | -3.35845 | -4.16694 |
| H | -0.90439 | -4.47077 | -2.82073 |

**$(21^{S})_{3t}^{+}$**

| | | | |
|---|---|---|---|
| C | -6.43935 | -2.29509 | -0.15327 |
| C | -6.17309 | -2.08229 | -1.50073 |
| C | -4.86161 | -1.91840 | -1.93441 |
| C | -3.80098 | -1.95920 | -1.02856 |
| C | -4.07969 | -2.17914 | 0.32346 |
| C | -5.38847 | -2.34503 | 0.75808 |
| C | -2.38642 | -1.77730 | -1.49472 |
| C | -1.82359 | -0.31020 | -1.41695 |
| C | -2.59323 | 0.64670 | -2.35916 |
| C | -2.23278 | 2.09965 | -2.17416 |
| C | -0.95808 | 2.59166 | -2.47009 |
| C | -0.64513 | 3.93193 | -2.26309 |
| C | -1.60171 | 4.80816 | -1.76208 |
| C | -2.87711 | 4.33359 | -1.47262 |
| C | -3.18434 | 2.99282 | -1.67415 |
| C | -0.39807 | -0.45191 | -1.87295 |
| N | 0.67719 | -0.57805 | -1.19096 |
| C | 1.80149 | -0.66111 | -2.13738 |

| | | | |
|---|---|---|---|
| C | 1.08018 | -0.97370 | -3.45147 |
| O | -0.26518 | -0.54497 | -3.19502 |
| C | 2.86959 | -1.64901 | -1.76343 |
| C | 4.16637 | -1.45878 | -2.24347 |
| C | 5.17803 | -2.35286 | -1.91730 |
| C | 4.90334 | -3.44469 | -1.09765 |
| C | 3.60873 | -3.64903 | -0.63415 |
| C | 2.58918 | -2.76397 | -0.97502 |
| Cu | 0.73904 | -0.90201 | 0.81637 |
| C | 1.51524 | -0.97823 | 2.75773 |
| C | 1.78225 | -2.27803 | 3.41400 |
| N | -1.12780 | 0.15242 | 0.94306 |
| C | -1.76355 | 0.74587 | 2.12881 |
| C | -3.22514 | 0.91813 | 1.67730 |
| O | -3.17500 | 0.68383 | 0.26509 |
| C | -1.97187 | 0.18970 | -0.00896 |
| C | -1.06131 | 2.02947 | 2.48960 |
| C | -0.96099 | 3.05060 | 1.54039 |
| C | -0.26634 | 4.21640 | 1.83504 |
| C | 0.34125 | 4.37260 | 3.07956 |
| C | 0.23587 | 3.36564 | 4.03183 |
| C | -0.46721 | 2.19950 | 3.73810 |
| O | 0.11415 | -2.74241 | 0.92223 |
| C | -0.82156 | -3.01342 | 1.77101 |
| O | -1.22579 | -2.23292 | 2.62588 |
| C | 2.03742 | 0.87939 | 1.30712 |
| C | 2.47385 | -0.29450 | 1.96713 |
| C | 3.77877 | -0.82132 | 1.66788 |
| C | 4.58413 | -0.17846 | 0.78716 |
| C | 4.14367 | 0.98350 | 0.07593 |
| C | 2.85094 | 1.51349 | 0.33648 |
| C | 2.39399 | 2.64350 | -0.37552 |
| C | 3.19335 | 3.22109 | -1.33471 |
| C | 4.47404 | 2.69914 | -1.59466 |
| C | 4.94470 | 1.60598 | -0.89703 |
| H | 1.06140 | -2.04961 | -3.66383 |
| H | -3.62138 | 1.92319 | 1.84559 |
| H | -2.41272 | 0.32244 | -3.38936 |
| H | -1.70739 | -2.40027 | -0.89915 |
| H | -2.28900 | -2.08526 | -2.54235 |
| H | -3.65796 | 0.51100 | -2.14981 |
| H | -1.68794 | 0.02343 | 2.94699 |
| H | -3.89729 | 0.17285 | 2.11593 |
| H | 2.26053 | 0.33920 | -2.18702 |
| H | 1.46145 | -0.43082 | -4.31864 |
| H | 4.38898 | -0.59065 | -2.86341 |
| H | 1.58007 | -2.94791 | -0.61272 |
| H | -1.41998 | 2.92674 | 0.55881 |
| H | -0.55621 | 1.41292 | 4.48744 |
| H | -0.20586 | 1.93422 | -2.90059 |
| H | -4.17935 | 2.62220 | -1.43451 |
| H | 0.34828 | 4.29750 | -2.51690 |
| H | -3.63767 | 5.01048 | -1.08895 |
| H | 0.88966 | 5.28387 | 3.30855 |
| H | 5.69618 | -4.14181 | -0.83423 |
| H | -1.36004 | 5.85859 | -1.61246 |
| H | 1.12180 | 1.37285 | 1.62737 |
| H | 4.11621 | -1.72497 | 2.16686 |
| H | 5.57929 | -0.56453 | 0.57764 |
| H | 5.93672 | 1.20763 | -1.10054 |
| H | 5.10125 | 3.16874 | -2.34969 |
| H | 2.84337 | 4.08905 | -1.88908 |
| H | 1.40187 | 3.03364 | -0.16118 |
| H | 0.67145 | -0.39336 | 3.13152 |
| H | 0.86615 | -2.68530 | 3.84351 |
| H | 2.21287 | -3.00448 | 2.71670 |
| H | 2.50781 | -2.11812 | 4.22722 |
| H | -4.65781 | -1.76749 | -2.99409 |
| H | -3.26627 | -2.20652 | 1.04878 |
| H | -6.98852 | -2.05232 | -2.22043 |
| H | -5.58837 | -2.52265 | 1.81311 |
| H | -7.46413 | -2.43069 | 0.18605 |
| H | -0.19924 | 5.00545 | 1.08813 |
| H | 0.69865 | 3.48815 | 5.00886 |
| H | 6.18500 | -2.19645 | -2.29942 |
| H | 3.38114 | -4.51166 | -0.01043 |
| C | -1.41264 | -4.39458 | 1.61679 |
| H | -2.02973 | -4.42233 | 0.70953 |
| H | -0.62071 | -5.13960 | 1.49297 |
| H | -2.03866 | -4.63722 | 2.47811 |

**(TS-3$^{R}$)$_{3t}^{+}$**

| | | | |
|---|---|---|---|
| C | 6.81678 | -1.60582 | -1.83012 |
| C | 5.73109 | -1.10575 | -2.54274 |
| C | 4.43841 | -1.47036 | -2.18601 |
| C | 4.21059 | -2.33535 | -1.11284 |
| C | 5.30749 | -2.84505 | -0.41738 |
| C | 6.60192 | -2.48040 | -0.77068 |
| C | 2.80614 | -2.68308 | -0.72002 |
| C | 2.15164 | -1.74616 | 0.35891 |
| C | 2.71979 | -2.04618 | 1.77396 |
| C | 2.19140 | -1.11257 | 2.83173 |
| C | 0.87105 | -1.20348 | 3.28471 |

| | | | |
|---|---|---|---|
| C | 0.37857 | -0.31005 | 4.22996 |
| C | 1.20119 | 0.68528 | 4.74875 |
| C | 2.52164 | 0.77330 | 4.32217 |
| C | 3.00877 | -0.11631 | 3.37115 |
| C | 0.69071 | -2.10496 | 0.34888 |
| N | -0.34572 | -1.43092 | 0.02381 |
| C | -1.51343 | -2.26912 | 0.32645 |
| C | -0.89078 | -3.66059 | 0.46502 |
| O | 0.48196 | -3.36654 | 0.74447 |
| C | -2.65036 | -2.20946 | -0.65369 |
| C | -2.54462 | -1.58501 | -1.89185 |
| C | -3.62866 | -1.56954 | -2.76637 |
| C | -4.81387 | -2.20837 | -2.42682 |
| C | -4.91705 | -2.85743 | -1.19854 |
| C | -3.84856 | -2.84479 | -0.31347 |
| Cu | -0.23383 | 0.33654 | -0.99675 |
| C | -2.45983 | 2.87910 | -1.24123 |
| C | -1.35053 | 3.73010 | -1.02720 |
| N | 1.75675 | 0.53285 | -0.61415 |
| C | 2.50283 | 1.79403 | -0.65390 |
| C | 3.90070 | 1.36706 | -0.16635 |
| O | 3.67050 | 0.07896 | 0.41686 |
| C | 2.46287 | -0.31604 | 0.02383 |
| C | 1.85278 | 2.83674 | 0.22144 |
| C | 1.23866 | 2.48723 | 1.42491 |
| C | 0.71364 | 3.47128 | 2.25859 |
| C | 0.79974 | 4.81250 | 1.89835 |
| C | 1.41012 | 5.16622 | 0.69734 |
| C | 1.92804 | 4.18312 | -0.13737 |
| O | -1.04679 | 1.08340 | -2.66398 |
| C | -0.27887 | 1.84631 | -3.33197 |
| O | 0.46635 | 2.69323 | -2.79326 |
| C | -2.93972 | 1.87437 | -0.36713 |
| C | -4.18704 | 1.30962 | -0.63210 |
| C | -4.80924 | 0.44976 | 0.28738 |
| C | -4.10687 | 0.09564 | 1.48065 |
| C | -2.80377 | 0.62442 | 1.70019 |
| C | -2.23750 | 1.49536 | 0.81879 |
| C | -6.11978 | -0.04442 | 0.07762 |
| C | -6.71453 | -0.85472 | 1.01157 |
| C | -6.01445 | -1.21638 | 2.17933 |
| C | -4.73648 | -0.75633 | 2.40878 |
| H | -0.94077 | -4.22743 | -0.47331 |
| H | 4.32331 | 2.02248 | 0.59921 |
| H | 2.47665 | -3.08714 | 2.01142 |
| H | 2.16567 | -2.64791 | -1.60994 |
| H | 2.74895 | -3.70052 | -0.31874 |
| H | 3.80787 | -1.95748 | 1.71167 |
| H | 2.52358 | 2.16473 | -1.68237 |
| H | 4.61615 | 1.23531 | -0.98588 |
| H | -1.89410 | -1.93682 | 1.30594 |
| H | -1.29469 | -4.26281 | 1.28160 |
| H | -1.62174 | -1.09931 | -2.19366 |
| H | -3.94899 | -3.33060 | 0.65712 |
| H | 1.16239 | 1.43991 | 1.71637 |
| H | 2.38416 | 4.45800 | -1.08726 |
| H | 0.23044 | -2.00615 | 2.92323 |
| H | 4.04105 | -0.04101 | 3.03532 |
| H | -0.64501 | -0.40961 | 4.58781 |
| H | 3.17765 | 1.53825 | 4.73230 |
| H | 0.39422 | 5.58176 | 2.55231 |
| H | -5.65459 | -2.21012 | -3.11780 |
| H | 0.81962 | 1.37674 | 5.49744 |
| H | -4.71241 | 1.57539 | -1.54818 |
| H | -1.25828 | 1.92060 | 1.02184 |
| H | -2.26561 | 0.33959 | 2.60217 |
| H | -4.20831 | -1.03026 | 3.32036 |
| H | -6.49513 | -1.86158 | 2.91210 |
| H | -7.72533 | -1.22334 | 0.85328 |
| H | -6.64596 | 0.23263 | -0.83367 |
| H | -3.03602 | 3.01510 | -2.15863 |
| H | -0.47956 | 3.23551 | -1.66788 |
| H | -1.43325 | 4.70263 | -1.52102 |
| H | -0.97152 | 3.79352 | -0.00536 |
| H | 3.58926 | -1.08147 | -2.74882 |
| H | 5.14843 | -3.54449 | 0.40240 |
| H | 5.89351 | -0.43900 | -3.38759 |
| H | 7.44760 | -2.88887 | -0.22131 |
| H | 7.83013 | -1.32631 | -2.11053 |
| H | 1.47656 | 6.21291 | 0.40728 |
| H | 0.24411 | 3.18304 | 3.19722 |
| H | -5.84042 | -3.36246 | -0.92279 |
| H | -3.52831 | -1.06200 | -3.72357 |
| C | -0.31674 | 1.72519 | -4.82982 |
| H | -0.00050 | 0.71599 | -5.11834 |
| H | -1.34731 | 1.84695 | -5.18240 |
| H | 0.33096 | 2.46683 | -5.30131 |

**(TS-3$^{S}$)$_{3t}^{+}$**

| | | | |
|---|---|---|---|
| C | 6.23819 | 2.64190 | -0.10936 |
| C | 6.10889 | 2.24819 | -1.43618 |
| C | 4.85108 | 1.97471 | -1.96294 |
| C | 3.70830 | 2.08568 | -1.17022 |

| | | | |
|---|---|---|---|
| C | 3.84804 | 2.49211 | 0.15989 |
| C | 5.10365 | 2.76647 | 0.68738 |
| C | 2.35142 | 1.76827 | -1.72474 |
| C | 1.86912 | 0.28433 | -1.52886 |
| C | 2.69758 | -0.70787 | -2.37994 |
| C | 2.41046 | -2.15592 | -2.06647 |
| C | 1.15546 | -2.73209 | -2.28661 |
| C | 0.91290 | -4.06133 | -1.95303 |
| C | 1.92114 | -4.84260 | -1.39937 |
| C | 3.17763 | -4.28468 | -1.18487 |
| C | 3.41444 | -2.95522 | -1.51311 |
| C | 0.44407 | 0.30345 | -2.00822 |
| N | -0.63875 | 0.41565 | -1.34183 |
| C | -1.75838 | 0.39317 | -2.28838 |
| C | -1.04798 | 0.59618 | -3.63515 |
| O | 0.32290 | 0.29344 | -3.33608 |
| C | -2.82785 | 1.40739 | -1.99303 |
| C | -4.10876 | 1.21700 | -2.51439 |
| C | -5.11634 | 2.13936 | -2.26391 |
| C | -4.85544 | 3.25942 | -1.47795 |
| C | -3.57955 | 3.45810 | -0.96416 |
| C | -2.56556 | 2.54231 | -1.22869 |
| Cu | -0.74781 | 0.71494 | 0.66933 |
| C | -1.65596 | 1.11026 | 2.78267 |
| C | -2.11564 | 2.19291 | 3.67319 |
| N | 1.21499 | 0.04947 | 0.88460 |
| C | 1.87448 | -0.45080 | 2.10352 |
| C | 3.33376 | -0.61690 | 1.64899 |
| O | 3.25699 | -0.51445 | 0.22182 |
| C | 2.04149 | -0.07332 | -0.07863 |
| C | 1.20350 | -1.72410 | 2.54846 |
| C | 1.13257 | -2.81268 | 1.67472 |
| C | 0.46858 | -3.97214 | 2.05328 |
| C | -0.14009 | -4.05387 | 3.30436 |
| C | -0.06298 | -2.97884 | 4.18235 |
| C | 0.61243 | -1.81996 | 3.80653 |
| O | -0.76997 | 2.61559 | 1.46599 |
| C | 0.33524 | 3.05381 | 2.00189 |
| O | 0.99267 | 2.43477 | 2.82100 |
| C | -2.07216 | -0.87235 | 1.41026 |
| C | -2.52816 | 0.36066 | 1.92435 |
| C | -3.81499 | 0.84810 | 1.52350 |
| C | -4.57802 | 0.13876 | 0.65033 |
| C | -4.10997 | -1.07786 | 0.07151 |
| C | -2.84636 | -1.59505 | 0.46901 |
| C | -2.36240 | -2.78941 | -0.11119 |
| C | -3.09938 | -3.43745 | -1.07257 |
| C | -4.35003 | -2.92509 | -1.47205 |
| C | -4.84953 | -1.77367 | -0.90597 |
| H | -1.10097 | 1.63664 | -3.97713 |
| H | 3.76613 | -1.58904 | 1.90043 |
| H | 2.50514 | -0.48551 | -3.43475 |
| H | 1.59900 | 2.40486 | -1.24122 |
| H | 2.31303 | 1.96808 | -2.80182 |
| H | 3.75282 | -0.50118 | -2.18173 |
| H | 1.78068 | 0.31457 | 2.87752 |
| H | 3.98555 | 0.18804 | 2.00515 |
| H | -2.21159 | -0.61023 | -2.23997 |
| H | -1.38672 | -0.07092 | -4.43073 |
| H | -4.32244 | 0.32823 | -3.10784 |
| H | -1.57615 | 2.71080 | -0.81052 |
| H | 1.59009 | -2.75149 | 0.68648 |
| H | 0.68239 | -0.97989 | 4.49756 |
| H | 0.35997 | -2.15388 | -2.75131 |
| H | 4.39471 | -2.51856 | -1.33088 |
| H | -0.06709 | -4.49346 | -2.14665 |
| H | 3.97772 | -4.88761 | -0.76046 |
| H | -0.66602 | -4.96006 | 3.59729 |
| H | -5.64595 | 3.97897 | -1.27479 |
| H | 1.73373 | -5.88495 | -1.14915 |
| H | -1.17887 | -1.34227 | 1.82111 |
| H | -4.17078 | 1.79824 | 1.91037 |
| H | -5.55057 | 0.51856 | 0.34514 |
| H | -5.81712 | -1.38149 | -1.21312 |
| H | -4.92828 | -3.45117 | -2.22890 |
| H | -2.72637 | -4.35478 | -1.52286 |
| H | -1.39591 | -3.17124 | 0.20818 |
| H | -0.72976 | 0.61520 | 3.08451 |
| H | -1.26244 | 2.72003 | 4.10570 |
| H | -2.78319 | 2.89927 | 3.17419 |
| H | -2.67322 | 1.71398 | 4.49476 |
| H | 4.75499 | 1.68167 | -3.00784 |
| H | 2.96767 | 2.58290 | 0.79664 |
| H | 6.99031 | 2.16120 | -2.06806 |
| H | 5.19545 | 3.08786 | 1.72319 |
| H | 7.22127 | 2.86204 | 0.30143 |
| H | 0.42617 | -4.81474 | 1.36542 |
| H | -0.52513 | -3.04313 | 5.16524 |
| H | -6.11052 | 1.98286 | -2.67797 |
| H | -3.36352 | 4.33201 | -0.35287 |
| C | 0.72831 | 4.43276 | 1.52927 |
| H | 0.92109 | 4.41893 | 0.44997 |

| | | | |
|---|---|---|---|
| H | -0.09773 | 5.13215 | 1.69725 |
| H | 1.62159 | 4.77178 | 2.05830 |

**TS6**

| | | | |
|---|---|---|---|
| C | -0.48733 | 0.72192 | 2.86131 |
| H | -1.52920 | 0.98575 | 3.06997 |
| H | 0.15459 | 1.52206 | 3.24767 |
| C | -0.27481 | 0.49157 | 1.37394 |
| H | -0.53111 | 1.57426 | 0.90451 |
| C | 1.14857 | 0.26091 | 0.98220 |
| C | 2.11947 | 1.24975 | 1.30449 |
| C | 1.56690 | -0.87347 | 0.32011 |
| C | 3.43829 | 1.08344 | 0.98273 |
| H | 1.78469 | 2.16789 | 1.78293 |
| H | 0.84547 | -1.63815 | 0.03981 |
| H | 4.16509 | 1.85671 | 1.22813 |
| O | -0.58715 | 2.92713 | 0.57769 |
| C | -0.74068 | 3.13581 | -0.79151 |
| C | 0.29181 | 2.35222 | -1.60000 |
| H | 1.30454 | 2.56215 | -1.23872 |
| H | 0.10887 | 1.27485 | -1.51224 |
| H | 0.23291 | 2.62105 | -2.66174 |
| C | -2.15761 | 2.78820 | -1.24381 |
| H | -2.89160 | 3.30725 | -0.61691 |
| H | -2.32531 | 3.06872 | -2.29090 |
| H | -2.31735 | 1.70756 | -1.15628 |
| C | -0.49057 | 4.64496 | -0.93896 |
| H | -1.20528 | 5.21018 | -0.33118 |
| H | 0.52350 | 4.89292 | -0.60880 |
| H | -0.60735 | 4.93690 | -1.98951 |
| B | -1.36954 | -0.40002 | 0.70810 |
| O | -2.41540 | -0.94106 | 1.39700 |
| C | -3.35997 | -1.44494 | 0.43655 |
| C | -2.46316 | -1.64983 | -0.83179 |
| O | -1.42555 | -0.67470 | -0.63059 |
| C | -1.78482 | -3.01171 | -0.86060 |
| H | -1.29636 | -3.22469 | 0.09779 |
| H | -2.49631 | -3.81680 | -1.07417 |
| H | -1.01774 | -3.00860 | -1.64280 |
| C | -3.15373 | -1.36788 | -2.14937 |
| H | -3.49984 | -0.33176 | -2.20439 |
| H | -2.45506 | -1.53573 | -2.97618 |
| H | -4.01396 | -2.03416 | -2.28662 |
| C | -4.41613 | -0.36594 | 0.24999 |
| H | -4.86322 | -0.13735 | 1.22321 |
| H | -3.97218 | 0.55545 | -0.14272 |
| H | -5.21012 | -0.69036 | -0.43149 |
| C | -3.98807 | -2.71182 | 0.97880 |
| H | -3.22906 | -3.44015 | 1.27688 |
| H | -4.59418 | -2.47468 | 1.85975 |
| H | -4.64196 | -3.17104 | 0.22754 |
| H | -0.25620 | -0.19175 | 3.42595 |
| C | 3.88551 | -0.08526 | 0.31687 |
| C | 5.24072 | -0.29350 | -0.02943 |
| C | 2.92464 | -1.08100 | -0.02116 |
| C | 5.63218 | -1.43655 | -0.68137 |
| C | 3.35932 | -2.24854 | -0.69417 |
| C | 4.68158 | -2.42366 | -1.01722 |
| H | 5.97106 | 0.47148 | 0.23056 |
| H | 6.67833 | -1.58523 | -0.94209 |
| H | 2.62220 | -3.00784 | -0.95212 |
| H | 5.00193 | -3.32594 | -1.53474 |

**TS7**

| | | | |
|---|---|---|---|
| C | 0.49180 | -2.03623 | 0.65000 |
| H | 1.74432 | -1.89088 | 0.73806 |
| H | 0.31190 | -3.08599 | 0.39228 |
| C | 0.07269 | -1.01029 | -0.36586 |
| H | 0.39979 | -1.32595 | -1.36508 |
| C | -1.41265 | -0.72729 | -0.45200 |
| C | -1.86970 | 0.10852 | -1.50754 |
| C | -2.33297 | -1.20745 | 0.44902 |
| C | -3.19246 | 0.43530 | -1.63437 |
| H | -1.14051 | 0.49463 | -2.21957 |
| H | -2.01617 | -1.85064 | 1.26779 |
| H | -3.52356 | 1.07561 | -2.45095 |
| O | 2.91688 | -1.43883 | 0.66546 |
| C | 3.70407 | -2.22087 | -0.19425 |
| C | 3.86828 | -3.62855 | 0.37026 |
| H | 4.28033 | -3.58318 | 1.38452 |
| H | 2.89418 | -4.13148 | 0.41965 |
| H | 4.53323 | -4.23719 | -0.25435 |
| C | 3.12230 | -2.25867 | -1.60429 |
| H | 2.91011 | -1.24081 | -1.95157 |
| H | 3.81899 | -2.73498 | -2.30443 |
| H | 2.18811 | -2.83365 | -1.62160 |
| C | 5.05036 | -1.48983 | -0.20709 |
| H | 4.93247 | -0.48513 | -0.62833 |
| H | 5.44115 | -1.39857 | 0.81203 |
| H | 5.77401 | -2.04416 | -0.81642 |
| B | 0.78366 | 0.36988 | -0.07702 |
| O | 1.37716 | 1.12504 | -1.04490 |
| C | 1.59911 | 2.43281 | -0.48400 |
| C | 1.58991 | 2.14543 | 1.05875 |

O 0.76303 0.97075 1.14233
C 2.95929 1.76924 1.60165
H 3.39984 0.95740 1.01490
H 3.63813 2.62937 1.60922
H 2.84301 1.40671 2.62861
C 0.96609 3.24128 1.89825
H -0.07977 3.40841 1.62652
H 1.00065 2.95750 2.95563
H 1.51743 4.18195 1.77871
C 0.42970 3.29850 -0.92933
H 0.38218 3.29493 -2.02391
H -0.51767 2.90047 -0.54666
H 0.53771 4.33420 -0.58915
C 2.90627 2.97967 -1.01670
H 3.72862 2.28082 -0.83965
H 2.82539 3.14625 -2.09651
H 3.14948 3.93661 -0.53926
H 0.20466 -1.80627 1.68106
C -4.15412 -0.04948 -0.71383
C -5.52934 0.26744 -0.81232
C -3.70982 -0.88790 0.34828
C -6.43048 -0.22198 0.09981
C -4.66409 -1.37758 1.27246
C -5.99219 -1.05280 1.15257
H -5.86145 0.90906 -1.62741
H -7.48602 0.02891 0.01435
H -4.32416 -2.01841 2.08488
H -6.71361 -1.43634 1.87168

**TS8**

C -0.03985 -1.07330 -2.58657
H 0.11098 -2.15883 -2.58909
H 0.45509 -0.67693 -3.48540
C 0.49421 -0.40921 -1.33376
H -0.10033 -1.02951 -0.38482
C 1.90992 -0.65756 -0.99112
C 2.53509 0.01374 0.05260
C 2.67321 -1.64042 -1.68667
C 3.86703 -0.25618 0.43022
C 3.96816 -1.91990 -1.34621
H 1.99569 0.78878 0.58910
H 2.22254 -2.17853 -2.51541
H 4.52745 -2.67225 -1.90077
C -1.99190 -3.37030 -0.88870
C -3.20920 -3.94306 -1.23618
C -1.98943 -2.34763 0.06207
C -4.40183 -3.52502 -0.63773
C -3.19916 -1.92603 0.67397
C -4.40432 -2.52109 0.32968
C -1.40764 -0.77046 1.49012
C -2.81520 -0.89255 1.61728
H -1.06071 -3.71480 -1.33039
H -3.23393 -4.73929 -1.97751
H -5.33862 -3.99926 -0.92337
H -5.33423 -2.21604 0.80647
C -0.69597 0.14938 2.25942
C -3.50630 -0.10863 2.53065
C -2.79183 0.81488 3.29330
C -1.40589 0.94212 3.15510
H 0.38192 0.23513 2.15719
H -4.58356 -0.20875 2.65109
H -3.31770 1.44094 4.01134
H -0.87183 1.66636 3.76715
N -0.89738 -1.67430 0.57404
B -0.12672 1.00777 -1.11480
O 0.31415 1.97530 -0.25423
C -0.40636 3.18520 -0.55202
C -1.70027 2.63788 -1.24357
O -1.24021 1.39672 -1.80409
C -2.80130 2.30071 -0.25192
H -2.41518 1.69870 0.57623
H -3.26208 3.20506 0.16076
H -3.57340 1.71642 -0.76382
C -2.23776 3.50931 -2.35983
H -1.50883 3.62374 -3.16679
H -3.14026 3.05234 -2.78007
H -2.50331 4.50344 -1.98032
C 0.47133 3.99463 -1.49487
H 1.44359 4.16258 -1.01952
H 0.64048 3.45283 -2.43261
H 0.02432 4.96697 -1.72928
C -0.65037 3.94142 0.73654
H -1.13207 3.30361 1.48252
H 0.30349 4.29069 1.14736
H -1.28512 4.81725 0.55540
H -1.11189 -0.88265 -2.69265
C 4.60860 -1.24599 -0.27786
C 5.94185 -1.51347 0.10571
C 4.49588 0.43138 1.49910
C 6.52239 -0.83236 1.14765
C 5.79167 0.14978 1.85050
H 3.92815 1.18859 2.03798
H 7.54971 -1.04757 1.43532

H 6.26199 0.68360 2.67411
H 6.50293 -2.27121 -0.43941

**TS9**

C 0.96007 -1.94687 1.40986
H -0.34073 -2.21004 1.51492
H 1.13622 -1.36221 2.31918
C 1.21153 -1.21805 0.12052
C 0.36721 0.03180 -0.03127
C -0.43981 0.22418 -1.12818
C 0.39069 1.02301 0.98427
C -1.27765 1.35977 -1.23703
C -0.40410 2.13446 0.90537
H -0.49093 -0.53912 -1.90583
H 1.04716 0.88704 1.84187
H -0.39210 2.87562 1.70293
C -1.53571 -3.36708 -0.82747
C -2.07151 -3.36499 -2.11057
C -2.00480 -2.41878 0.07927
C -3.06901 -2.45424 -2.47693
C -2.99378 -1.47709 -0.29337
C -3.54305 -1.51114 -1.56922
C -2.25322 -1.07805 1.83121
C -3.14890 -0.59679 0.84606
H -0.76607 -4.07973 -0.53806
H -1.71620 -4.09065 -2.83973
H -3.47901 -2.48766 -3.48442
H -4.31035 -0.79639 -1.86091
C -2.12058 -0.42976 3.05732
C -3.91960 0.53376 1.09073
C -3.77622 1.18600 2.31043
C -2.88612 0.70834 3.27909
H -1.42800 -0.80006 3.81006
H -4.60377 0.91452 0.33377
H -4.36081 2.08079 2.51453
H -2.79362 1.23759 4.22576
N -1.61256 -2.23042 1.39517
B 2.69699 -0.69838 0.01165
O 3.38721 -0.60016 -1.15757
C 4.47996 0.30878 -0.91576
C 4.69792 0.15212 0.62581
O 3.37443 -0.18904 1.08038
C 5.60199 -1.01922 0.97675
H 5.28920 -1.92614 0.44637
H 6.64784 -0.81174 0.72497
H 5.53374 -1.21105 2.05289
C 5.15132 1.41162 1.33304
H 4.41497 2.21310 1.22817
H 5.28363 1.20931 2.40154
H 6.11036 1.75846 0.92950
C 3.98119 1.69485 -1.29715
H 3.64010 1.67458 -2.33749
H 3.13149 1.99015 -0.66928
H 4.76879 2.45017 -1.20096
C 5.66110 -0.09577 -1.77041
H 5.92150 -1.14722 -1.62074
H 5.41711 0.04641 -2.82869
H 6.53690 0.52131 -1.53559
H 1.41078 -2.94292 1.47863
C -1.26750 2.33298 -0.19777
C -2.14028 3.44277 -0.28429
C -2.17084 1.53113 -2.32223
C -3.00091 3.57805 -1.34422
C -3.01722 2.61075 -2.37261
H -2.18165 0.77949 -3.10984
H -3.67487 4.43111 -1.39641
H -3.70326 2.72671 -3.20979
H 1.01573 -1.87380 -0.73760
H -2.12727 4.18159 0.51571

**13’**

C -0.65230 3.07995 0.11426
H -0.24056 3.62723 -0.74785
H -0.23071 3.56069 1.01037
C -0.31627 1.62163 0.05739
C 1.05480 1.21887 0.03806
C 1.43837 -0.12949 -0.01382
C 2.11157 2.19234 0.06927
C 2.78942 -0.52608 -0.03420
C 3.42523 1.82971 0.05021
H 0.67133 -0.89755 -0.03798
H 1.85822 3.24814 0.10915
H 4.20250 2.59251 0.07495
B -1.51535 0.65229 0.02571
O -1.48167 -0.72056 -0.04354
C -2.81109 -1.20106 0.20988
C -3.68110 0.02397 -0.21832
O -2.80588 1.12246 0.06870
C -3.95478 0.05029 -1.71552
H -3.02890 -0.10364 -2.28192
H -4.67696 -0.71918 -2.01019
H -4.36079 1.03125 -1.98444
C -4.96702 0.19538 0.56294
H -4.76890 0.34569 1.62775

```
H  -5.51044  1.07163  0.19273
H  -5.61337 -0.68283  0.44433
C  -2.90443 -1.48920  1.70133
H  -2.11281 -2.19344  1.97849
H  -2.76461 -0.57066  2.28305
H  -3.87183 -1.92829  1.96927
C  -3.04402 -2.46240 -0.59462
H  -2.80852 -2.31022 -1.65150
H  -2.40312 -3.26686 -0.21714
H  -4.08745 -2.78938 -0.50865
H  -1.73392  3.23592  0.12561
C   3.81534  0.46386 -0.00183
C   5.16522  0.05819 -0.02222
C   3.16538 -1.89275 -0.08619
C   5.49866 -1.27600 -0.07258
C   4.48823 -2.25914 -0.10483
H   2.38110 -2.64779 -0.11124
H   6.54450 -1.57666 -0.08776
H   4.76214 -3.31171 -0.14480
H   5.94315  0.82008  0.00276
```

**13”**

```
C  -0.94485  3.35189 -0.56999
H  -0.23774  3.97546 -0.02537
C  -0.58463  1.98975 -1.02177
C   0.68504  1.42376 -0.42714
C   1.55947  0.67281 -1.17983
C   0.92670  1.55742  0.96551
C   2.69064  0.04885 -0.60357
C   2.01347  0.96706  1.55434
H   1.37850  0.54291 -2.24646
H   0.21604  2.12326  1.56568
H   2.18444  1.07754  2.62436
B  -1.60576  0.86208 -0.59611
O  -1.80750 -0.27051 -1.32888
C  -2.31861 -1.25728 -0.41144
C  -3.02228 -0.36342  0.66213
O  -2.22929  0.83753  0.61437
C  -4.43963  0.02171  0.26802
H  -4.46942  0.39555 -0.76205
H  -5.12677 -0.82768  0.35056
H  -4.78897  0.81936  0.93224
C  -2.98614 -0.91795  2.07045
H  -1.95790 -1.03191  2.42458
H  -3.50585 -0.23460  2.75094
H  -3.48647 -1.89295  2.11642
C  -1.10840 -1.99380  0.14614
H  -0.53607 -2.41666 -0.68641
H  -0.44939 -1.30523  0.69030
H  -1.40172 -2.80690  0.81915
C  -3.23603 -2.20273 -1.15488
H  -4.01633 -1.65919 -1.69486
H  -2.65835 -2.78176 -1.88350
H  -3.71197 -2.90534 -0.46003
H  -1.91162  3.77821 -0.83040
C   2.92757  0.19757  0.79401
C   4.05792 -0.42972  1.37009
C   3.59686 -0.72469 -1.36997
C   4.91755 -1.17301  0.60113
C   4.68356 -1.32177 -0.78296
H   3.41446 -0.83745 -2.43771
H   5.78344 -1.65049  1.05575
H   5.37123 -1.91341 -1.38439
H  -0.50968  1.93746 -2.12223
H   4.23394 -0.31159  2.43843
```

## 10. References


1. Vu, J.; Haug, G. C.; Li, Y.; Zhao, B.; Chang, C. J.; Paton, R. S.; Dong, Y., Enantioconvergent Cross-Nucleophile Coupling: Copper-Catalyzed Deborylative Cyanation. *Angew. Chem. Int. Ed.* **2024**, *63*, e202408745.
2. Vu, J.; Haug, G. C.; Schubert, T. J.; Head, J. F.; Paton, R. S.; Dong, Y., Enantioconvergent Chan–Lam Coupling: Synthesis of Chiral Benzylic Amides via Cu-Catalyzed Deborylative Amidation. *J. Am. Chem. Soc.* **2025**, *147*, 25527-25535.
3. Xiao, H.; Cao, P.; Xiong, W.; Yang, T., Overcoming limitations in Chan-Lam amination with alkylboronic esters via aminyl radical substitution. *Nat. Commun.* **2025**, *16*, 9121.
4. Huninik, P.; Im, H.; Szyling, J.; Baik, M.-H.; Walkowiak, J., Markovnikov-Selective Hydroboration of Aryl Alkenes Catalyzed by Quaternary Ammonium Salts. *J. Am. Chem. Soc.* **2025**, *147*, 44152-44161.
5. Jin, H.; Han, J.; Liu, X.; Feng, C.; Zhan, M., Access to Diverse Organoborons by α-Deprotonation and Functionalization of Benzylboronates. *Org. Lett.* **2023**, *25*, 4168-4172.
6. Bagutski, V.; Ros, A.; Aggarwal, V. K., Improved method for the conversion of pinacolboronic esters into trifluoroborate salts: facile synthesis of chiral secondary and tertiary trifluoroborates. *Tetrahedron* **2009**, *65*, 9956-9960.
7. Zhou, Q.; Srinivas, H. D.; Zhang, S.; Watson, M. P., Accessing Both Retention and Inversion Pathways in Stereospecific, Nickel-Catalyzed Miyaura Borylations of Allylic Pivalates. *J. Am. Chem. Soc.* **2016**, *138*, 11989-11995.
8. Bull, S. D.; Davies, S. G.; Garner, A. C.; Kruchinin, D.; Key, M.-S.; Roberts, P. M.; Savory, E. D.; Smith, A. D.; Thomson, J. E., SuperQuat 5,5-dimethyl-4-iso-propyloxazolidin-2-one as a mimic of Evans 4-tert-butyloxazolidin-2-one. *Org. Biomol. Chem.* **2006**, *4*, 2945-2964.
9. Ha, M. W.; Hong, S.; Park, C.; Park, Y.; Lee, J.; Kim, M.-h.; Lee, J.; Park, H.-g., Enantioselective phase-transfer catalytic *α*-alkylation of 2-methylbenzyl *tert*-butyl malonates. *Org. Biomol. Chem.* **2013**, *11*, 4030-4039.
10. Dakarapu, R.; Falck, J. R., Stereospecific Stille Cross-Couplings Using Mn(II)$Cl_2$. *J. Org. Chem.* **2018**, *83*, 1241-1251.
11. Holt, D. A.; Luengo, J. I.; Yamashita, D. S.; Oh, H. J.; Konialian, A. L.; Yen, H. K.; Rozamus, L. W.; Brandt, M.; Bossard, M. J.; Levy, M. A.; Eggleston, D. S.; Liang, J.; Schultz, L. W.; Stout, T. J.; Clardy, J., Design, synthesis, and kinetic evaluation of high-affinity FKBP ligands and the X-ray crystal structures of their complexes with FKBP12. *J. Am. Chem. Soc.* **1993**, *115*, 9925-9938.
12. Holt, D. A.; Konialian-Beck, A. L.; Oh, H.-J.; Yen, H.-K.; Rozamus, L. W.; Krog, A. J.; Erhard, K. F.; Ortiz, E.; Levy, M. A.; Brandt, M.; Bossard, M. J.; Luengo, J. I., Structure-activity studies of synthetic FKBP ligands as peptidyl-prolyl isomerase inhibitors. *Bioorg. Med. Chem. Lett.* **1994**, *4*, 315-320.
13. Bruker *APEX2*, Bruker AXS Inc.: Madison, Wisconsin, USA, 2012.
14. Sheldrick, G. M., A short history of *SHELX*. *Acta Cryst.* **2008**, *A64*, 112-122.
15. Wang, L.; Li, T.; Perveen, S.; Zhang, S.; Wang, X.; Ouyang, Y.; Li, P., Nickel-Catalyzed Enantioconvergent Carboxylation Enabled by a Chiral 2,2′-Bipyridine Ligand. *Angew. Chem. Int. Ed.* **2022**, *61*, e202213943.
16. Ryan, M. C.; Martinelli, J. R.; Stahl, S. S., Cu-Catalyzed Aerobic Oxidative N–N Coupling of Carbazoles and Diarylamines Including Selective Cross-Coupling. *J. Am. Chem. Soc.* **2018**, *140*, 9074-9077.

17. Hong, W.; Chun, M. S.; Jeong, K. S.; Kim, J. J.; Song, O. K.; Yoon, H. S. Compound and organic light emitting display (OLED) comprising the same. US11502261B2 2022.
18. Heurich, T.; Nesterov, V.; Schnakenburg, G.; Qu, Z.-W.; Grimme, S.; Hazin, K.; Gates, D. P.; Engeser, M.; Streubel, R., Strong Evidence of a Phosphanoxyl Complex: Formation, Bonding, and Reactivity of Ligated Phosphorus Analogues of Nitroxides. *Angew. Chem. Int. Ed.* **2016**, *55*, 14439-14443.
19. Maltese, M.; Vergari, M. C.; Donzello, M. P., Zinc chloride homogeneous catalysis in the tritylation of hydroxyl- and amide-bearing molecules. *Tetrahedron Lett.* **2011**, *52*, 483-487.
20. Yamataka, H.; Fujimura, N.; Kawafuji, Y.; Hanafusa, T., Electron transfer in reactions of ketones with organolithium reagents. A carbon-14 kinetic isotope effect probe. *J. Am. Chem. Soc.* **1987**, *109*, 4305-4308.
21. Desai, L. V.; Stowers, K. J.; Sanford, M. S., Insights into Directing Group Ability in Palladium-Catalyzed C−H Bond Functionalization. *J. Am. Chem. Soc.* **2008**, *130*, 13285-13293.
22. Mullins, R. J.; Vedernikov, A.; Viswanathan, R., Competition Experiments as a Means of Evaluating Linear Free Energy Relationships. An Experiment for the Advanced Undergraduate Organic Chemistry Lab. *J. Chem. Educ.* **2004**, *81*, 1357.
23. Huang, H.; Kang, J. Y., Mitsunobu Reaction Using Basic Amines as Pronucleophiles. *J. Org. Chem.* **2017**, *82*, 6604-6614.
24. Dasgupta, A.; Stefkova, K.; Babaahmadi, R.; Yates, B. F.; Buurma, N. J.; Ariafard, A.; Richards, E.; Melen, R. L., Site-Selective $Csp^3$–Csp/$Csp^3$–$Csp^2$ Cross-Coupling Reactions Using Frustrated Lewis Pairs. *J. Am. Chem. Soc.* **2021**, *143*, 4451-4464.
25. Jia, H.; He, M.; Yang, S.; Yu, X.; Bao, M., Visible-Light-Driven di-*t*-Butyl Peroxide-Promoted the Oxidative Homo- and Cross-Coupling of Phenols. *European Journal of Organic Chemistry* **2022**, *2022*, e202101469.
26. Kumari, R.; Wadepalli, S.; Mondal, S.; Hazarika, A.; Santra, A., Visible Light-Induced Di-*tert*-Butyl Peroxide (DTBP)-Mediated Controlled Oxidation of Alcohols. *ChemPhotoChem* **2026**, *10*, e202500382.
27. Scaiano, J. C.; Wubbels, G. G., Photosensitized dissociation of di-tert-butyl peroxide. Energy transfer to a repulsive excited state. *J. Am. Chem. Soc.* **1981**, *103*, 640-645.
28. Liu, X.-M.; Li, F.; Xue, L.-Y.; Dai, L.; Liu, J.-Y.; Xiao, L.-J.; Zhou, Q.-L., Copper-Catalyzed Enantioselective Oxidative Coupling Between Benzylic C($sp^3$)–H Bond and Carboxylic Acids. *CCS Chemistry* **2025**, *7*, 2970-2977.
29. Pracht, P.; Bohle, F.; Grimme, S., Automated exploration of the low-energy chemical space with fast quantum chemical methods. *Phys. Chem. Chem. Phys.* **2020**, *22*, 7169-7192.
30. Bannwarth, C.; Caldeweyher, E.; Ehlert, S.; Hansen, A.; Pracht, P.; Seibert, J.; Spicher, S.; Grimme, S., Extended tight-binding quantum chemistry methods. *WIREs Comput Mol Sci.* **2021**, *11*, e1493.
31. Neese, F., Software Update: The ORCA Program System—Version 6.0. *WIREs Comput Mol Sci.* **2025**, *15*, e70019.
32. Frisch, M. J.; Trucks, G. W.; Schlegel, H. B.; Scuseria, G. E.; Robb, M. A.; Cheeseman, J. R.; Scalmani, G.; Barone, V.; Petersson, G. A.; Nakatsuji, H.; Li, X.; Caricato, M.; Marenich, A. V.; Bloino, J.; Janesko, B. G.; Gomperts, R.; Mennucci, B.; Hratchian, H. P.; Ortiz, J. V.; Izmaylov, A. F.; Sonnenberg, J. L.; Williams; Ding, F.; Lipparini, F.; Egidi, F.; Goings, J.; Peng, B.; Petrone, A.; Henderson, T.; Ranasinghe, D.; Zakrzewski, V. G.; Gao, J.; Rega, N.; Zheng, G.; Liang, W.; Hada, M.; Ehara, M.; Toyota, K.; Fukuda, R.; Hasegawa, J.; Ishida, M.; Nakajima, T.; Honda, Y.; Kitao, O.; Nakai, H.; Vreven, T.; Throssell, K.; Montgomery Jr., J.

A.; Peralta, J. E.; Ogliaro, F.; Bearpark, M. J.; Heyd, J. J.; Brothers, E. N.; Kudin, K. N.; Staroverov, V. N.; Keith, T. A.; Kobayashi, R.; Normand, J.; Raghavachari, K.; Rendell, A. P.; Burant, J. C.; Iyengar, S. S.; Tomasi, J.; Cossi, M.; Millam, J. M.; Klene, M.; Adamo, C.; Cammi, R.; Ochterski, J. W.; Martin, R. L.; Morokuma, K.; Farkas, O.; Foresman, J. B.; Fox, D. J. *Gaussian 16 Rev. A.03*, Wallingford, CT, 2016.
33. Zhao, Y.; Truhlar, D. G., Design of Density Functionals That Are Broadly Accurate for Thermochemistry, Thermochemical Kinetics, and Nonbonded Interactions. *J. Phys. Chem. A* **2005**, *109*, 5656-5667.
34. Grimme, S.; Ehrlich, S.; Goerigk, L., Effect of the damping function in dispersion corrected density functional theory. *J. Comput. Chem.* **2011**, *32*, 1456-1465.
35. Grimme, S.; Antony, J.; Ehrlich, S.; Krieg, H., A consistent and accurate ab initio parametrization of density functional dispersion correction (DFT-D) for the 94 elements H-Pu. *J. Chem. Phys.* **2010**, *132*.
36. Weigend, F.; Ahlrichs, R., Balanced basis sets of split valence, triple zeta valence and quadruple zeta valence quality for H to Rn: Design and assessment of accuracy. *Phys. Chem. Chem. Phys.* **2005**, *7*, 3297-3305.
37. Marenich, A. V.; Cramer, C. J.; Truhlar, D. G., Universal Solvation Model Based on Solute Electron Density and on a Continuum Model of the Solvent Defined by the Bulk Dielectric Constant and Atomic Surface Tensions. *J. Phys. Chem. B* **2009**, *113*, 6378-6396.
38. Luchini, G.; Alegre-Requena, J.; Funes-Ardoiz, I.; Paton, R., GoodVibes: automated thermochemistry for heterogeneous computational chemistry data [version 1; peer review: 2 approved with reservations]. *F1000Research* **2020**, *9*.
39. Funes-Ardoiz, I.; Paton, R. S., GoodVibes: GoodVibes v1.0.1. *Zenodo* **2016**.
40. Legault, C., *CYLview, 1.0 b*. Sherbrooke, QC: http://www.cylview.org: Université de Sherbrooke, 2009.
41. Humphrey, W.; Dalke, A.; Schulten, K., VMD: Visual molecular dynamics. *J. Mol. Graph. Model.* **1996**, *14*, 33-38.
42. Boto, R. A.; Peccati, F.; Laplaza, R.; Quan, C.; Carbone, A.; Piquemal, J.-P.; Maday, Y.; Contreras-García, J., NCIPLOT4: Fast, Robust, and Quantitative Analysis of Noncovalent Interactions. *J. Chem. Theory Comput.* **2020**, *16*, 4150-4158.
43. Wu, P.; Chaudret, R.; Hu, X.; Yang, W., Noncovalent Interaction Analysis in Fluctuating Environments. *J. Chem. Theory Comput.* **2013**, *9*, 2226-2234.
44. Horn, P. R.; Mao, Y.; Head-Gordon, M., Probing non-covalent interactions with a second generation energy decomposition analysis using absolutely localized molecular orbitals. *Phys. Chem. Chem. Phys.* **2016**, *18*, 23067-23079.
45. Epifanovsky, E.; Gilbert, A. T. B.; Feng, X.; Lee, J.; Mao, Y.; Mardirossian, N.; Pokhilko, P.; White, A. F.; Coons, M. P.; Dempwolff, A. L.; Gan, Z.; Hait, D.; Horn, P. R.; Jacobson, L. D.; Kaliman, I.; Kussmann, J.; Lange, A. W.; Lao, K. U.; Levine, D. S.; Liu, J.; McKenzie, S. C.; Morrison, A. F.; Nanda, K. D.; Plasser, F.; Rehn, D. R.; Vidal, M. L.; You, Z.-Q.; Zhu, Y.; Alam, B.; Albrecht, B. J.; Aldossary, A.; Alguire, E.; Andersen, J. H.; Athavale, V.; Barton, D.; Begam, K.; Behn, A.; Bellonzi, N.; Bernard, Y. A.; Berquist, E. J.; Burton, H. G. A.; Carreras, A.; Carter-Fenk, K.; Chakraborty, R.; Chien, A. D.; Closser, K. D.; Cofer-Shabica, V.; Dasgupta, S.; de Wergifosse, M.; Deng, J.; Diedenhofen, M.; Do, H.; Ehlert, S.; Fang, P.-T.; Fatehi, S.; Feng, Q.; Friedhoff, T.; Gayvert, J.; Ge, Q.; Gidofalvi, G.; Goldey, M.; Gomes, J.; González-Espinoza, C. E.; Gulania, S.; Gunina, A. O.; Hanson-Heine, M. W. D.; Harbach, P. H. P.; Hauser, A.; Herbst, M. F.; Hernández Vera, M.; Hodecker, M.; Holden, Z. C.;

Houck, S.; Huang, X.; Hui, K.; Huynh, B. C.; Ivanov, M.; Jász, Á.; Ji, H.; Jiang, H.; Kaduk, B.; Kähler, S.; Khistyaev, K.; Kim, J.; Kis, G.; Klunzinger, P.; Koczor-Benda, Z.; Koh, J. H.; Kosenkov, D.; Koulias, L.; Kowalczyk, T.; Krauter, C. M.; Kue, K.; Kunitsa, A.; Kus, T.; Ladjánszki, I.; Landau, A.; Lawler, K. V.; Lefrancois, D.; Lehtola, S.; Li, R. R.; Li, Y.-P.; Liang, J.; Liebenthal, M.; Lin, H.-H.; Lin, Y.-S.; Liu, F.; Liu, K.-Y.; Loipersberger, M.; Luenser, A.; Manjanath, A.; Manohar, P.; Mansoor, E.; Manzer, S. F.; Mao, S.-P.; Marenich, A. V.; Markovich, T.; Mason, S.; Maurer, S. A.; McLaughlin, P. F.; Menger, M. F. S. J.; Mewes, J.-M.; Mewes, S. A.; Morgante, P.; Mullinax, J. W.; Oosterbaan, K. J.; Paran, G.; Paul, A. C.; Paul, S. K.; Pavošević, F.; Pei, Z.; Prager, S.; Proynov, E. I.; Rák, Á.; Ramos-Cordoba, E.; Rana, B.; Rask, A. E.; Rettig, A.; Richard, R. M.; Rob, F.; Rossomme, E.; Scheele, T.; Scheurer, M.; Schneider, M.; Sergueev, N.; Sharada, S. M.; Skomorowski, W.; Small, D. W.; Stein, C. J.; Su, Y.-C.; Sundstrom, E. J.; Tao, Z.; Thirman, J.; Tornai, G. J.; Tsuchimochi, T.; Tubman, N. M.; Veccham, S. P.; Vydrov, O.; Wenzel, J.; Witte, J.; Yamada, A.; Yao, K.; Yeganeh, S.; Yost, S. R.; Zech, A.; Zhang, I. Y.; Zhang, X.; Zhang, Y.; Zuev, D.; Aspuru-Guzik, A.; Bell, A. T.; Besley, N. A.; Bravaya, K. B.; Brooks, B. R.; Casanova, D.; Chai, J.-D.; Coriani, S.; Cramer, C. J.; Cserey, G.; DePrince, A. E., III; DiStasio, R. A., Jr.; Dreuw, A.; Dunietz, B. D.; Furlani, T. R.; Goddard, W. A., III; Hammes-Schiffer, S.; Head-Gordon, T.; Hehre, W. J.; Hsu, C.-P.; Jagau, T.-C.; Jung, Y.; Klamt, A.; Kong, J.; Lambrecht, D. S.; Liang, W.; Mayhall, N. J.; McCurdy, C. W.; Neaton, J. B.; Ochsenfeld, C.; Parkhill, J. A.; Peverati, R.; Rassolov, V. A.; Shao, Y.; Slipchenko, L. V.; Stauch, T.; Steele, R. P.; Subotnik, J. E.; Thom, A. J. W.; Tkatchenko, A.; Truhlar, D. G.; Van Voorhis, T.; Wesolowski, T. A.; Whaley, K. B.; Woodcock, H. L., III; Zimmerman, P. M.; Faraji, S.; Gill, P. M. W.; Head-Gordon, M.; Herbert, J. M.; Krylov, A. I., Software for the frontiers of quantum chemistry: An overview of developments in the Q-Chem 5 package. *J. Chem. Phys.* **2021**, *155*.

46. Liu, X.-M.; Li, F.; Wang, T.; Dai, L.; Yang, Y.; Jiang, N.-Q.; Xue, L.-Y.; Liu, J.-Y.; Xue, X.-S.; Xiao, L.-J.; Zhou, Q.-L., Catalytic Asymmetric Oxidative Coupling between C($sp^3$)–H Bonds and Carboxylic Acids. *J. Am. Chem. Soc.* **2025**, *147*, 627-635.

47. Fan, L.-W.; Tang, J.-B.; Wang, L.-L.; Gao, Z.; Liu, J.-R.; Zhang, Y.-S.; Yuan, D.-L.; Qin, L.; Tian, Y.; Chen, Z.-C.; Liu, F.; Xiang, J.-M.; Huang, P.-J.; Liu, W.-L.; Xiao, C.-Y.; Luan, C.; Li, Z.-L.; Hong, X.; Dong, Z.; Gu, Q.-S.; Liu, X.-Y., Copper-catalysed asymmetric cross-coupling reactions tolerant of highly reactive radicals. *Nature Chem.* **2026**, *18*, 142-151.

48. Cordero, B.; Gómez, V.; Platero-Prats, A. E.; Revés, M.; Echeverría, J.; Cremades, E.; Barragán, F.; Alvarez, S., Covalent radii revisited. *Dalton Trans.* **2008**, 2832-2838.

49. Zhou, Y.; Chen, P.; Liu, G., Copper-Catalyzed Highly Efficient and Asymmetric Allylic C–H Oxidation of 3-Aryl-Substituted Terminal Alkenes. *J. Am. Chem. Soc.* **2025**, *147*, 22873-22882.

50. Wang, P.-Z.; Liang, Y.-J.; Wu, X.; Guan, W.; Xiao, W.-J.; Chen, J.-R., Copper-Catalyzed Three-Component Photo-ATRA-Type Reaction for Asymmetric Intermolecular C–O Coupling. *ACS Catal.* **2022**, *12*, 10925-10937.

51. Li, F.; Liu, X.-M.; Pan, J.-B.; Dai, L.; Yang, Y.; Xiao, L.-J.; Fan, C.; Zhou, Q.-L., Photo- and Copper-Catalyzed Enantioselective Oxidation of Benzylic C($sp^3$)–H Bonds. *J. Am. Chem. Soc.* **2025**, *147*, 19093-19100.

52. Tang, S.; Xu, H.; Dang, Y.; Yu, S., Photoexcited Copper-Catalyzed Enantioselective Allylic C($sp^3$)–H Acyloxylation of Acyclic Internal Alkenes. *J. Am. Chem. Soc.* **2024**, *146*, 27196-27203.

53. Silverstein, T. P., Marcus Theory: Thermodynamics CAN Control the Kinetics of Electron Transfer Reactions. *J. Chem. Educ.* **2012**, *89*, 1159-1167.
54. Marcus, R. A., On the Theory of Oxidation-Reduction Reactions Involving Electron Transfer. I. *J. Chem. Phys.* **1956**, *24*, 966-978.
55. Nelsen, S. F.; Blackstock, S. C.; Kim, Y., Estimation of inner shell Marcus terms for amino nitrogen compounds by molecular orbital calculations. *J. Am. Chem. Soc.* **1987**, *109*, 677-682.
56. Bernasconi, C. F., The principle of nonperfect synchronization: more than a qualitative concept? *Acc. Chem. Res.* **1992**, *25*, 9-16.
57. Salamone, M.; Galeotti, M.; Romero-Montalvo, E.; van Santen, J. A.; Groff, B. D.; Mayer, J. M.; DiLabio, G. A.; Bietti, M., Bimodal Evans–Polanyi Relationships in Hydrogen Atom Transfer from C($sp^3$)–H Bonds to the Cumyloxyl Radical. A Combined Time-Resolved Kinetic and Computational Study. *J. Am. Chem. Soc.* **2021**, *143*, 11759-11776.
